# Asymmetric Intramolecular α‐Arylation of Polar Amino Acids Bearing β‐Leaving Groups

**DOI:** 10.1002/anie.202507713

**Published:** 2025-05-24

**Authors:** Ömer Taspinar, Daniel J. Leonard, Nathan Picois, Cornelia Göcke, Matej Žabka, Hazel A. Sparkes, Jonathan Clayden

**Affiliations:** ^1^ School of Chemistry University of Bristol Cantock's Close Bristol BS8 1TS UK; ^2^ CY Cergy Paris Université, CNRS, BioCIS Cergy Pontoise 95000 France

**Keywords:** Amino acids, Arylation, Hydantoins, Rearrangement, Ureas

## Abstract

The α‐arylation of amino acids may be achieved by intramolecular nucleophilic aromatic substitution (S_N_Ar) reactions of amino‐acid derived enolates, but for amino acids bearing β‐leaving groups, such reactions are complicated by competing E1cB elimination of the β‐substituent. In this paper we report an approach to the arylation of the polar amino acids serine, cysteine, diaminopropionic acid, and allothreonine by inducing intramolecular S_N_Ar reactions of heterocycles, which the heteroatom substituent is stereoelectronically protected from elimination by incorporating it into the ring system of *N*‐carbamoyl oxazolidines, thiazolidines, or imidazolidines. The sequence comprises the diastereoselective formation of a heterocyclic urea followed by an intramolecular N‐to‐C aryl migration, yielding bicyclic hydantoins that can be further hydrolysed to afford quaternary α‐aryl amino acids. The method is practical and scalable, avoids the use of transition metals or chiral auxiliaries, and provides the opportunity to access a variety of α‐arylated products bearing electronically diverse benzenoid or heterocyclic substituents (35 examples).

## Graphical abstract

Use of enolate chemistry to functionalise amino acids with β‐heteroatom side‐chains (serine, cysteine, threonine, etc.) is hampered by β‐elimination. We show that α‐arylation can be achieved by incorporating the side chain into a saturated heterocycle, making the unwanted elimination a disfavoured 5‐*endo‐trig* reaction. An alternative, now favoured, unactivated intramolecular S_N_Ar reaction provides a range of functionalised amino acid derivatives.

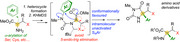

Quaternary (or Cα,α‐disubstituted) amino acids (QAAs) occur in non‐ribosomal peptide natural products and are important tools for governing conformation and controlling metabolic degradation in synthetic peptidomimetic structures.^[^ ^1^, ^2^, ^3^, ^4^, ^5^, ^6^, ^7^, ^8^ ^]^ They are also of value as building blocks for the synthesis of pharmaceuticals, agrochemicals, and natural products.^[^ ^9^, ^10^, ^11^, ^12^, ^13^, ^14^, ^15^, ^16^, ^17^, ^18^, ^19^, ^20^, ^21^ ^]^ Notably, heterocyclic derivatives of QAAs show promising biological properties as androgen receptor modulators and NK_1_ antagonists (Scheme 1a).^[^ ^22^, ^23^, ^24^ ^]^ They have also been used as precursors for imidazolidinone organocatalyts.^[^ ^25^ ^]^

**Scheme 1:**
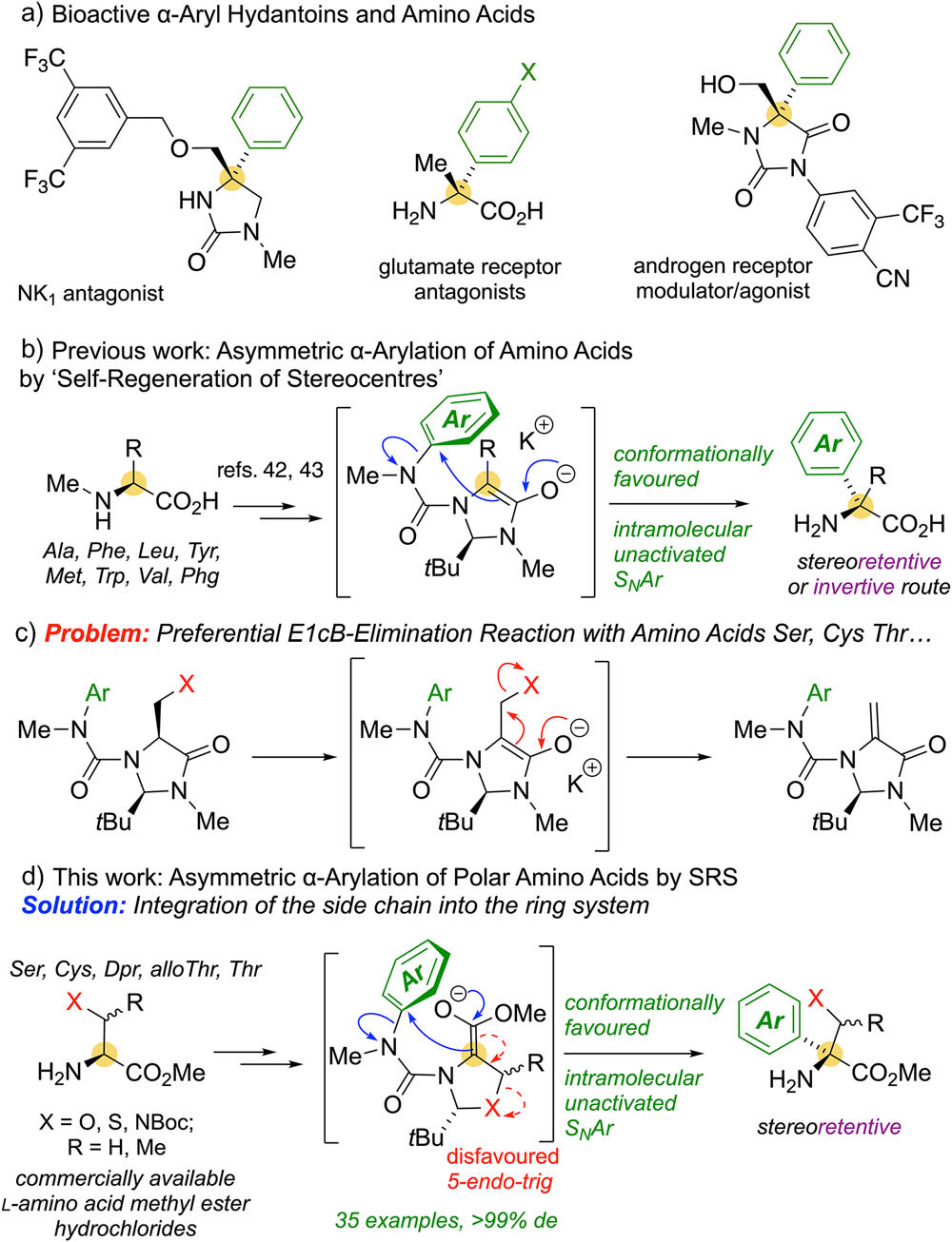
Concept of the work: a) Examples of relevant bioactive molecules; b) Previous asymmetric α‐arylation based on SRS; c) Problematic elimination reactions with amino acids bearing β‐leaving groups; d) Extension of the previous methodology to polar amino acids by using an alternative template.

QAAs are typically prepared by α‐functionalisation of their naturally occurring tertiary precursors.^[^ ^26^, ^27^ ^]^ Although α‐alkylation is a well‐established method,^[^ ^28^, ^29^, ^30^, ^31^, ^32^, ^33^ ^]^ few approaches to the stereoselective preparation of α‐arylated variants have been reported.^[^ ^34^, ^35^, ^36^, ^37^, ^38^, ^39^, ^40^, ^41^ ^]^ We showed that Seebach's “self‐regeneration of stereocentres” (SRS) strategy, in which the configurational purity of the starting material is conserved by the use of a second, temporary stereogenic centre, can be modified to induce stereoselective intramolecular nucleophilic aromatic substitution (S_N_Ar) reactions in conformationally preorganised *N*‐carbamoylimidazolidinone derivatives of a selection of typically hydrophobic amino acids (Scheme 1b), even with electronically unactivated substrates.^[^ ^42^, ^43^ ^]^ However, the method failed with polar amino acids containing β‐leaving groups (Ser, Cys, Thr), owing to preferential E1cB elimination reactions (Scheme 1c).^[^ ^44^, ^45^ ^]^ We now present a solution to this limitation that now allows the enantioselective arylation of functionalised, polar amino acids, including serine and cysteine, both essential polar amino acids with significant roles in biochemical processes.^[^ ^46^, ^47^, ^48^, ^49^, ^50^ ^]^

Key to avoiding the competitive elimination was the incorporation of the amino acid heteroatom side chain into a ring (Scheme 1d). The potential for elimination is diminished by the resulting 5‐*endo*‐*trig* stereoelectronics, disfavoured according to Baldwin's rules,^[^ ^51^, ^52^, ^53^ ^]^ of the consequent ring opening. Seebach and co‐workers utilised related heterocycles to minimise a similar side‐reaction in alkylations of serine and cysteine.^[^ ^54^, ^55^, ^56^, ^57^, ^58^ ^]^

L‐Serine methyl ester hydrochloride (**1**) was converted into heterocyclic carbamoyl chloride **3** in a two‐step sequence (Scheme 2a).^[^ ^59^, ^60^ ^]^ Cyclisation of **1** using triethylamine and pivaldehyde in CH_2_Cl_2_ in the presence of MgSO_4_ gave a 2:1 mixture of the diastereoisomers of oxazolidine **2**. Treatment of the crude diastereomeric mixture in dry CH_2_Cl_2_ with phosgene (15 wt.% in toluene) and triethylamine resulted in a marked enhancement of the diastereomeric ratio, affording, after 3 h, the thermodynamically favoured *cis* carbamoyl chloride as a single diastereomer **3** in 99% yield.^[^ ^61^ ^]^ Carbamoyl chloride **3** reacted with *N*‐methylaniline in refluxing dichloromethane in the presence of triethylamine^[^ ^42^, ^43^ ^]^ to yield the diastereoisomerically pure urea **4a** in 98% yield. The structures **3** and **4a** were confirmed by X‐ray crystallography.^[^ ^62^ ^]^

**Scheme 2:**
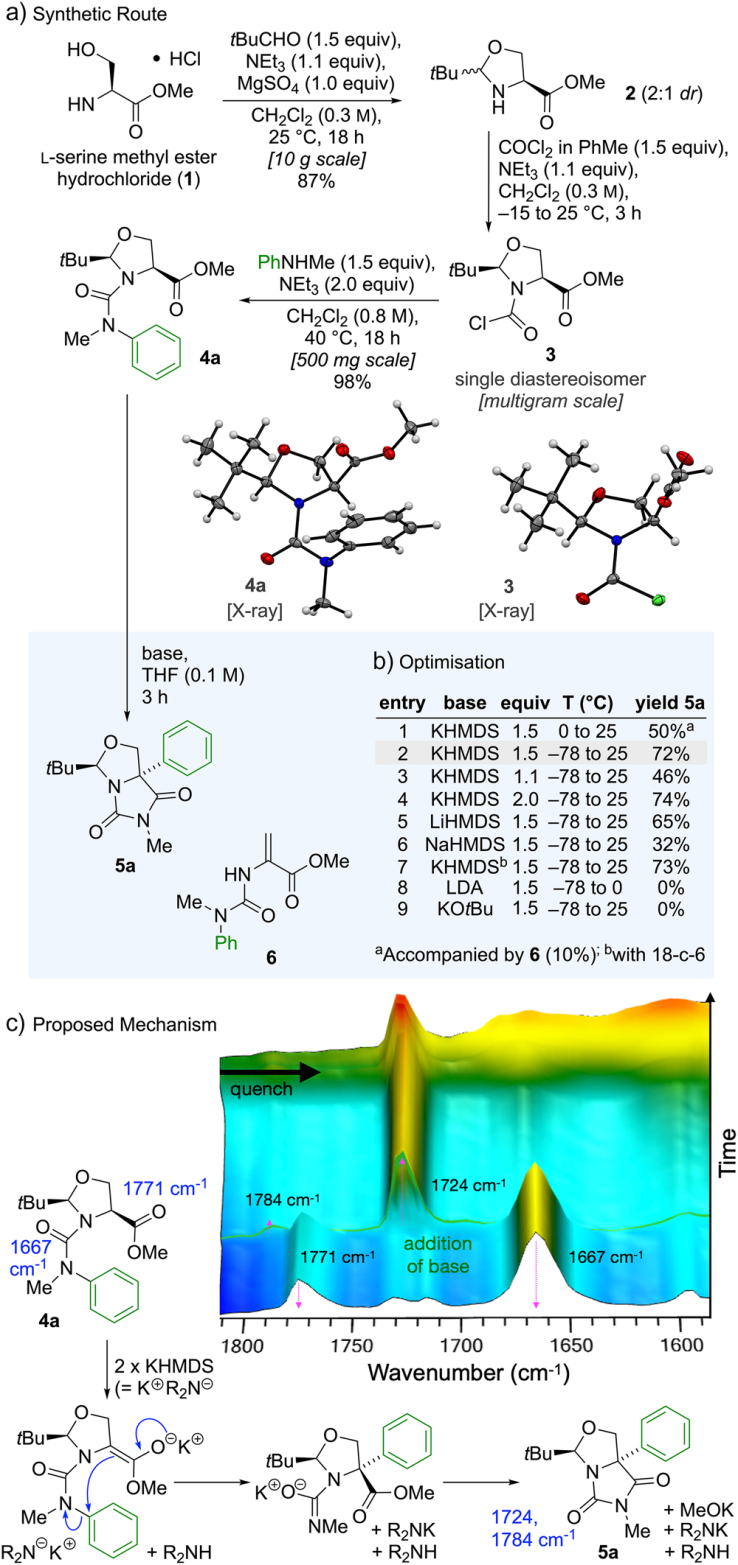
a) Synthesis of urea **4a** from L‐serine methyl ester hydrochloride **1** and rearrangement to the bicyclic hydantoin **5**. b) Optimisation of the reaction of **4a** to give **5a**, with **6** as a by‐product. c) In situ infrared study of the rearrangement of **4a**, with carbonyl stretching frequencies and proposed mechanism of the rearrangement

We first reported the conformationally‐driven Truce‐miles‐like rearrangement of anionic derivatives of *N*’‐aryl ureas in 2007,^[^ ^63^ ^]^ and have employed variants of the reaction to make target molecules including α‐aryl and α‐vinyl amino acids and their hydantoin analogues.^[^ ^41^, ^42^, ^43^, ^64^, ^65^, ^66^, ^67^ ^]^ *N*‐Carbamoyl oxazolidine **4a** was treated under conditions similar to those employed previously to rearrange *N*‐carbamoyl imidazolidinones. The reaction employing potassium bis(trimethylsilyl)amide (KHMDS, 1.5 equiv.) at room temperature resulted in a complex mixture of compounds, with a single diastereoisomer of the hydantoin **5a** (arising from cyclisation of the urea onto the pendent ester after rearrangement) as the major product^[^ ^68^ ^]^ (Scheme 2b, entry 1) and the unwanted elimination product **6** as a minor component (10%). Adding the base at −78 °C (entry 2) before allowing the mixture to warm slowly to room temperature over 3 h significantly improved the yield of the single diastereoisomer of **5a** to 72%. Reducing the amount of KHMDS to 1.1 equivalents gave a lower yield of 46% (entry 3), whereas increasing the amount to 2.0 equiv. yielded 74% (entry 4). Substituting KHMDS with LiHMDS or NaHMDS reduced the yield (entries 5 and 6) and alternative bases (LDA or KO*t*Bu, entries 8 and 9) gave no product. 18‐Crown‐6^[^ ^66^ ^]^ had only a marginal effect (entry 7).

The rearrangement of **4a** under the conditions of entry 2 was monitored at −78 °C by in situ infrared spectroscopy (React‐IR), as depicted in Scheme 2b. The transformation of the starting material **4a** (urea C═O stretch 1667 cm^−1^, ester C═O stretch 1771 cm^−1^) occurs directly and almost instantaneously to yield the bicyclic product **5a** (1724 cm^−1^, 1784 cm^−1^). The reaction sequence of deprotonation, aryl migration and cyclisation proceeded without detectable intermediates, indicating that the rearrangement, despite the lack of an electron‐withdrawing substituent, is faster than enolate formation, even at low temperatures. Computational studies (see below) suggested that two solvated potassium cations are involved in the mechanism, consistent with the positive effect of excess KHMDS (Scheme 2b).

We applied the optimised method to the α‐arylation of other polar amino acids bearing β‐heteroatom substituents, namely cysteine **7** and 2,3‐diaminopropionic acid (Dpr) **8**, threonine **9**, and allothreonine **10**, each used as their methyl ester hydrochloride salts (Scheme 3). The formation of the oxazolidine, thiazolidine, and imidazolidine derivatives of these amino acids gave diastereomeric mixtures of compounds **11**–**14**. In each case, treatment of the mixture with phosgene and triethylamine converted these mixtures into a single diastereoisomer of the corresponding heterocyclic carbamoyl chloride. Both steps could be performed on a multigram scale.^[^ ^69^ ^]^

**Scheme 3:**
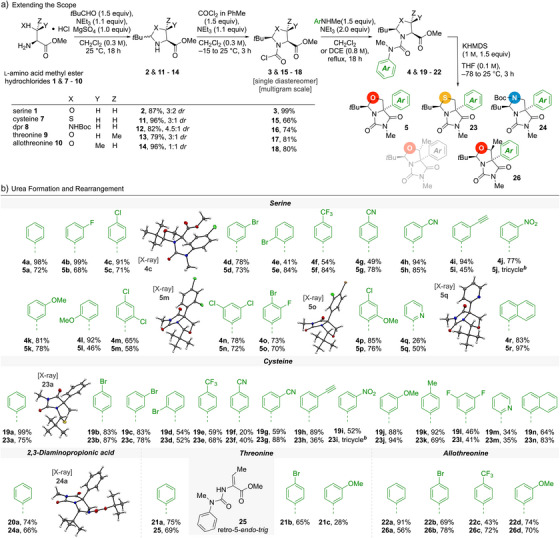
a) Stereoselective synthesis of the *N*‐carbamoyl chlorides from polar L‐amino acid methyl ester hydrochlorides; b) Scope of the urea formation and rearrangement steps including X‐ray crystal structures of selected compounds. [a] All rearrangement reactions were performed on a 100‐mg scale in 0.1 M THF. [b] See Supporting Information for details. [c] 0.8 M DCE was used as the solvent for the urea formation.

Carbamoyl chlorides **3** and **15**–**18** were treated overnight at either 40 °C in CH_2_Cl_2_ or (for the cysteine derivative **15**) at 84 °C in refluxing DCE with a range of *N*‐methylanilines to give a series of urea derivatives **4** and **19 – 22** bearing functionalised *N*‐aryl substituents that included electron‐rich and electron‐deficient phenyl rings, halo‐ and polyhaloarenes, pyridines, and naphthalenes. These ureas were treated with base to explore the scope of the rearrangement reaction leading to the α‐arylation of their parent amino acid (Scheme 3b).

The serine‐derived ureas **4**, cysteine‐derived ureas **19**, and Dpr‐derived ureas **20** generally underwent stereoselective rearrangement and ring closure to give single diastereoisomers of the bicyclic hydantoins **5**, **23** and **24**. Thus, substituted phenyl rings carrying electronegative substituents, such as fluoro (**5b**), chloro (**5c**), bromo (for example, **5d**, **5e**, **23b** or **23c**), trifluoromethyl (**5f** or **23e**), or cyano (**5g** and **5h**) rearranged to give high yields regardless of the position of the substituent. All halogenated rings, including those bearing bromo‐substituents, rearranged without evidence of dehalogenation or benzyne formation, but nitro‐substituted **5j** and **23i** gave tricyclic structures, which are further detailed in the Supporting Information. Alkynyl‐substituted **5i** and **23h** formed cleanly, providing versatile intermediates for potential cross‐coupling or cycloaddition reactions.

Similarly, despite the key step being an intramolecular nucleophilic aromatic substitution reaction, electron‐rich rings rearranged successfully, with high yields for *meta*‐ or *para*‐substituted methoxy and methyl derivatives **5k**, **23j** or **23k**. Some *ortho*‐substituted aryl groups gave more moderate yields (for example, **5l** and **23d)**.

2‐Pyridyl rings (**5q** and **23m**) rearranged in low yield; disubstituted rings (for example **5o** or **23l**) rearranged regardless of the position of the substituents and their electron density, and 2‐naphthyl substituents (**5r** and **23n**) also gave good yields of both serine and cysteine derivatives.

Boc‐protected Dpr‐derived urea **20a** was successfully converted into the corresponding triazabicyclic structure **24a**, the structure of which was confirmed by X‐ray crystallography.^[^ ^62^ ^]^ The rearrangement shows remarkable tolerance to variations in the aryl migrating group, with fused, heterocyclic, electron‐deficient, and electron‐rich rings all participating in the reaction. Representative X‐ray crystal structures are depicted in Scheme 3b.^[^ ^62^ ^]^

Threonine and allothreonine‐derived ureas **21** and **22** showed divergent reactivity. Allothreonine‐derived ureas **22** gave the bicyclic compounds (**26a‐d**) in a manner similar to their serine counterparts. However, threonine‐derived ureas **21** (in which the aryl ring would need to migrate to the oxazolidine *syn* to the methyl substituent) failed to rearrange and instead gave elimination products (exemplified by **25**) as a consequence of the retro 5‐*endo*‐*trig* ring opening we had hoped to disfavour. Computational investigations (see Supporting information) of the rearrangement of **22a** suggested that inclusion of two solvated potassium cations (Scheme 2c) results in a reasonable modelled barrier for the S_N_Ar step of ∼30 kJ mol^−1^. The corresponding transition state structures for **21a** would not converge. Instead, migration of K^+^ led to a low‐energy pathway for elimination to give **25** with a barrier of ∼33 kJ mol^−1^.

The bicyclic products of the rearrangement **5**, **23**, **24** and **26** are α,α‐disubstituted amino acid derivatives, and they were transformed either to monocyclic hydantoins (a class of compounds with known biological activity), or to amino acids, by methods illustrated in Scheme 4. Hydrolysis under acidic conditions of a representative selection of 10 bicycles yielded the hydantoins **27–30** in moderate to high yields. The optimal reaction conditions were determined to be aqueous HCl in ethanol (6 M, 10:1) at 120 °C for 90 min, with the Boc‐protecting group of **24a** (Scheme 4) being removed to yield **29a**, and a cyano function being hydrolysed to the carboxyl derivative **27f**. The structure of the α‐phenylserine‐derived hydantoin **27a** was confirmed by X‐ray crystallography.^[^ ^62^, ^70^ ^]^

**Scheme 4:**
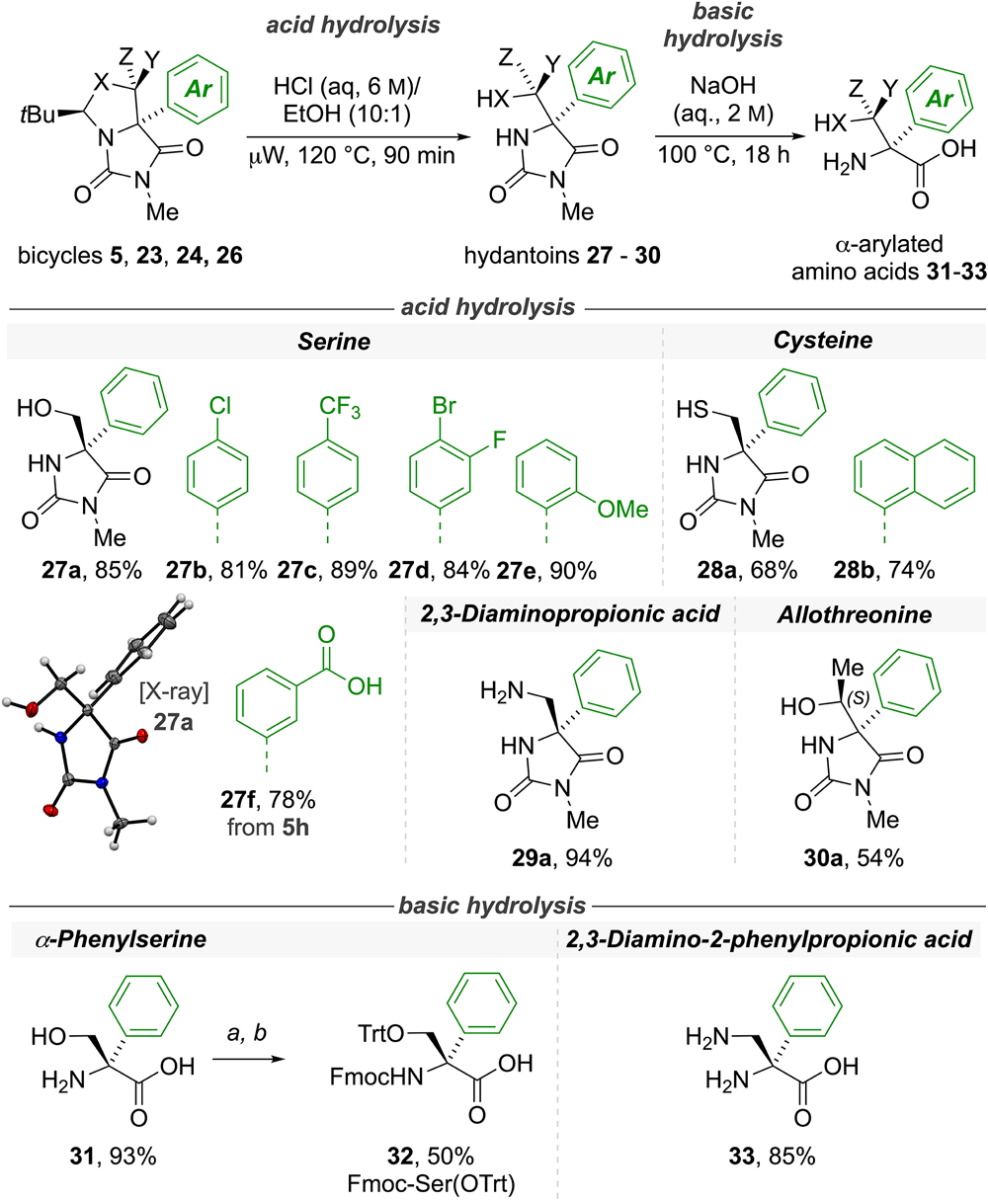
Acidic and basic hydrolysis of α‐arylated bicyclic ureas. The stereochemistry of the serine‐derived hydantoin **27a** was confirmed by X‐ray crystallography. Derivatisation of representative quaternary α‐arylated serine with *N*‐Fmoc and *O*‐Trt protecting groups for solid‐phase peptide synthesis (SPPS). i. Fmoc‐OSu (2 equiv), Na_2_CO_3_ (10 equiv). H_2_O/dioxane (1:1 v/v), 16 h, 25 °C; ii. TrtCl (2 equiv), DIPEA (1.5 equiv), CH_2_Cl_2_, 16 h, 25 °C.

A small selection of hydantoins were converted into α‐arylated amino acids through a second hydrolysis step under basic conditions, using aqueous sodium hydroxide (2 M) at 100 °C for 18 h, affording the desired quaternary α‐amino acids. Phenylserine was protected at the *N*‐terminus to give the corresponding fluorenylmethyloxycarbonyl (Fmoc) carbamate using standard conditions (Fmoc‐OSu and sodium carbonate), and the hydroxy side chain was protected as a triphenylmethyl (trityl) group (TrtCl and DIPEA).^[^ ^71^, ^72^ ^]^ The protected residue thus formed provides a suitable α‐arylated amino acid building block for use in solid‐phase peptide synthesis (SPPS)^[^ ^67^, ^73^ ^]^ with orthogonal protection allowing side chain modifications and selective deprotection.

In conclusion, we have demonstrated a practical method for introducing an aryl substituent to the α‐C atom of the polar amino acids serine, cysteine, diaminopropionic acid, and allothreonine, and their derived hydantoins. Competing elimination of their β‐hydroxyl, thiol, or amino substituents was disfavoured stereoelectronically through the use of a five‐membered ring. Such arylated amino acids do not occur naturally, but are valuable synthetic building blocks, and their incorporation into peptide structures could allow the exploration of new chemical space. The key step of the sequence is a KHMDS‐mediated intramolecular N‐to‐C aryl migration entailing an electronically unactivated S_N_Ar reaction, which facilitates the cyclisation of *N*‐aryl urea derivatives to bicyclic hydantoins in good yields, with a broad substrate scope and functional group tolerance (35 examples). The method is transition‐metal‐free and avoids complex catalysts or auxiliaries, making it practical and economical.

## Supporting Information

The data that support the findings of this study such as full experimental details, characterisation data and additional references are available in the Supporting Information of this article.

## Conflict of Interests

The authors declare no conflict of interest.

## Supporting information



Supporting Information

## Data Availability

The data that support the findings of this study are available in the Supporting Information of this article.
