# Supplementary material for: Asymmetric Intramolecular α‐Arylation of Polar Amino Acids Bearing β‐Leaving Groups
Source: Angew Chem Int Ed Engl. 2025 May 24;64(29):e202507713. doi: 10.1002/anie.202507713 (PMC12258666; doi:10.1002/anie.202507713)
Supplement: Supplementary file 1 — Supporting Information [file ANIE-64-e202507713-s001.pdf]

Supporting Information

Asymmetric Intramolecular α-Arylation of Polar Amino Acids Bearing β-Leaving Groups

Ömer Taşpınar, Daniel J. Leonard, Nathan Picois, Cornelia Göcke, Matej Žabka, Hazel A. Sparkes, and Jonathan Clayden*

School of Chemistry, University of Bristol
Cantock's Close, Bristol BS8 1TS, United Kingdom
E-mail: j.clayden@bristol.ac.uk

Table of Contents

1. Overview Schemes (Synthetic Route) S3

2. General Information S4

2.1 Analytical Methods S4

2.2 Chromatographic Methods S5

2.3 Miscellaneous Information S5

3. General Procedures S6

4. Experimental Procedures and Characterisation Data S9

4.1 Formation of the Heterocyclic Rings according to GP1 .... S9

4.2 Formation of the Carbamoyl Chlorides according to GP2 .... S18

4.3 Formation of the *N*-Aryl Ureas according to GP3 .... S28

4.4 N to C Rearrangement (Aryl Migration) according to GP4 .... S110

4.5 Acidic Hydrolysis according to GP5 .... S186

4.6 Basic Hydrolysis according to GP6 .... S206

5. In situ IR Spectroscopy S212

6. X-Ray Crystallographic Data S213

7. Computational Studies S230

7.1 Computational Details S230

7.2 Investigated Reactions S231

7.3 Energy Profiles S232

7.4 Computed Energies S236

7.5 Coordinates of Computed Structures S238

8. References S281

1. Overview Schemes (Synthetic Route)

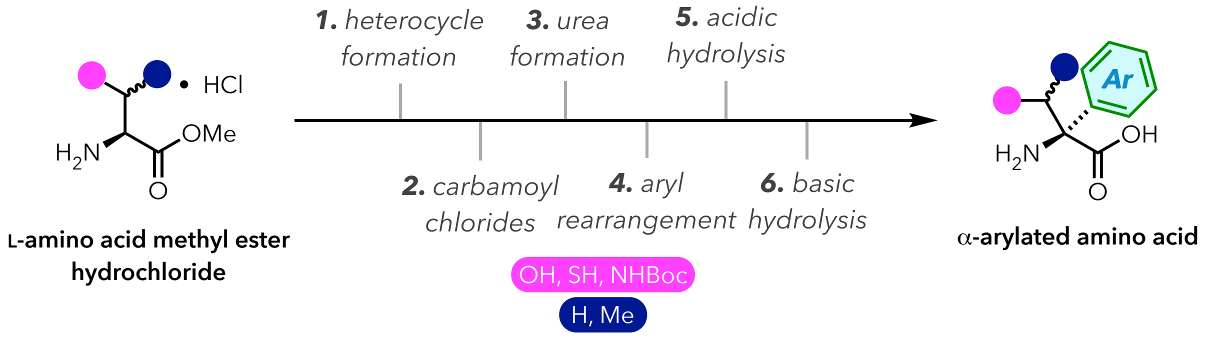

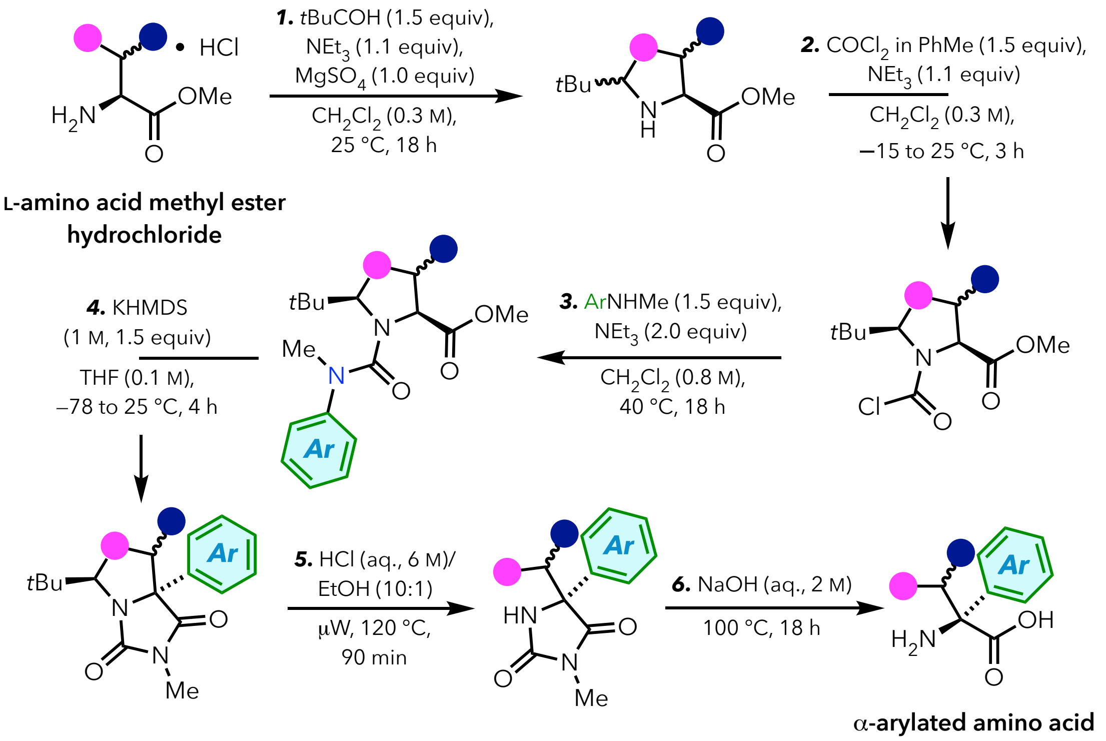

Scheme S1. Overview of experiments **GP1** to **GP6**.

2. General Information

All reactions were performed under anhydrous conditions (unless otherwise stated) under a dry nitrogen or argon atmospheres in glassware that was dried using either a combination of vacuum and heat-gun, oven, or flame drying. Reaction mixtures were stirred magnetically. Air- and moisture-sensitive liquids and solutions were transferred via syringe into the reaction vessels through rubber septa. All reagents were purchased at highest commercial quality and used as received. Non-anhydrous solvents were purchased (unless specified) at the highest commercial quality and used as received. Anhydrous CH_2Cl_2 and THF were obtained from the University of Bristol's dry solvent system and were purified by filtration over a column of activated alumina. PE refers to the fractions of petroleum ether fraction that boils in the range 40 to 60 °C. Reactions below 0 °C were cooled using an ice water/NaCl bath for reactions at –20 °C and an acetone/dry ice bath for reactions at –78 °C.

2.1 Analytical Methods

2.1.1 Nuclear magnetic resonance spectroscopy (NMR) ^{1}H and ^{13}C NMR spectra were obtained in $CDCl_3$, CD_3OD or D_2O at ambient temperature on a *Bruker* Ascend (400 MHz), *Bruker* Avance III HD (500 MHz) or *Varian* Cryo (600 MHz) spectrometer. Chemical shifts for the ^{1}H, ^{13}C and ^{19}F NMR spectra were recorded in parts per million (ppm) on δ scale quoted to the nearest 0.01 ppm. Signals are reported in relative to the residual signal of the non-deuterated solvent (^{1}H: 7.26 ppm and ^{13}C: 77.0 for $CDCl_3$; ^{1}H: 3.31 ppm and ^{13}C: 49.0 ppm for CD_3OD; ^{1}H: 4.79 ppm for D_2O). Abbreviations of fine structure of ^{1}H NMR spectra used: s = singlet, d = doublet, dd = doublet of doublets, ddd = doublet of doublets of doublets, td = triplet of doublets, t = triplet, m = multiplet, br = broad. Scalar coupling constants (*J*) are given in Hertz (Hz). ^{13}C NMR spectra were recorded using either APT or standard sequence with complete proton decoupling quoted to the nearest 0.1 ppm. Abbreviations of fine structure of ^{13}C NMR spectra used: s for quaternary C, d for CH, t for CH_2 and q for CH_3. ^{19}F chemical shifts are quoted to the nearest 0.1 ppm, relative to hexafluorobenzene (-164.9 ppm) as an internal standard. The non-trivial assignments were determined by ^{1}H,^{1}H COSY, ^{1}H,^{1}H NOESY, ^{1}H,^{13}C HSQCed and ^{1}H,^{13}C HMBC spectra.

2.1.2 Fourier transform infrared spectroscopy (FT-IR) Infrared (IR) spectra were obtained on neat compounds in 4000 – 500 cm^{-1} range using a *PerkinElmer* Spectrum Two FT-IR spectrometer using ATR technique. Selected absorption bands are reported in cm^{-1}. Abbreviations of fine structure of spectra used: w = weak, m = middle strong, s = strong, vs = very strong, bw = broad weak.

2.1.3 High-resolution electrospray ionisation mass spectrometry (HR-MS) High-resolution mass spectra were recorded by the technical staff at the University of Bristol on a *Thermo Scientific* Orbitrap Elite (ESI or APCI) or on a *Bruker* ultrafleXtreme 2 spectrometers (MALDI or TOF), with only molecular ions of interest reported.

2.1.4 Specific optical rotation ($[a]_{\lambda}^{T}$) Optical rotation values were determined with a *Bellingham + Stanley Ltd.* ADP220 polarimeter using a cell with a pathlength of 2.5 cm. The measurements were performered at ambient temperature in chloroform. The concentration c of the measured solution (g/100 mL) are preceded in squared brackets with optical rotation values. Letter D represents the Fraunhofer d-lines at 589 nm.

2.2. Chromatographic Methods

2.2.1 Thin-layer chromatography (TLC) Reactions were monitored on TLC on silica gel 60 F_{254} (layer thickness 0.25 mm, *Merck*) visualized with UV fluorescence (254 and 366 nm) and further developed using a potassium permanganate ($KMnO_4$) stain (1.5 g of $KMnO_4$, 10 g of K_2CO_3 and 1.25 mL NaOH (aq. 10%) in 200 mL of H_2O) or ninhydrin stain (0.3 g of ninydrin in 100 mL of *n*-butanol and 3 mL AcOH), and subsequent heating.

2.2.2 Column chromatography Flash column chromatography was performed on an automated *Biotage* Isolera Spektra Four on pre-packed silica gel *Biotage* Sfär D Duo columns (100 Å pore size, 60 mm particle size) or *Biotage* Sfär C18 Duo reverse phase columns (100 Å pore size, 30 mm particle size).

2.3 Miscellaneous Information

2.3.1 Melting-point apparatus Melting points were determined with a *Stuart Scientific* SMP10 melting point apparatus in open capillary tubes and are uncorrected.

2.3.2 X-ray crystallography Crystals for X-ray crystallography were grown by slow solvent evaporation and analysed by the technical staff at the University of Bristol on a *Bruker* D8 Venture single-crystal diffractometer. Details about X-ray crystallographic data and structure refinement is presented in the respective section.

2.3.3 Microwave reactor Microwave reactions were carried out in a *Biotage* Initiator+ microwave system with robot Eight.

3. General Procedures

General Procedure 1 (GP1): Formation of the heterocyclic rings

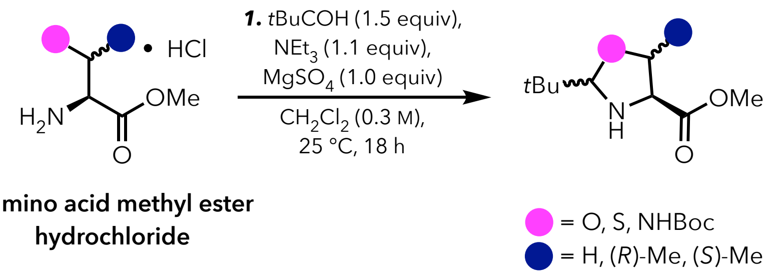

To a solution of L-amino acid methyl ester hydrochloride (1.0 equiv.) in dichloromethane (0.3 M, HPLC grade) was added magnesium sulfate (1.0 equiv.), triethylamine (1.1 equiv.) and pivaldeyhde (1.5 equiv.). The reaction mixture was stirred for 20 h at ambient temperature. The magnesium sulfate was removed by filtration, and triethylamine as well as CH_2Cl_2 were removed under reduced pressure. The crude product (as a white residue) was then dissolved in EtOAc, $NaHCO_3$ (sat. aq.) was added and the layers were separated. The aqueous phase was further extracted with EtOAc and the combined organic layers were washed with brine, dried over $MgSO_4$ and concentrated under reduced pressure to give a crude diastereomeric heterocycle (as a colourless oil), which was used in the next step without further purification. *For analytical purposes, a small sample was purified by column chromatography.*

General Procedure 2 (GP2): Formation of the carbamoyl chlorides

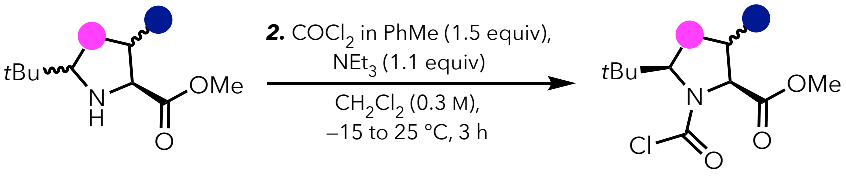

To a stirred solution of the crude heterocycle (1.0 equiv.) in dry dichloromethane (0.3 M) was added a phosgene solution (1.5 equiv., 15 wt. % in toluene) at –15 °C and triethylamine (1.3 equiv.). The reaction mixture was allowed to warm to room temperature over an hour and (the colour changes to beige) left for a further 2 h. The yellow reaction mixture was quenched with HCl (1 M, aq.) and the layers were separated. The aqueous layer was extracted with CH_2Cl_2 and the combined organic layers were washed with HCl (1 M, aq.), brine and dried over $MgSO_4$. The solvent was removed under reduced pressure to give an orange oil, which was purified by silica gel column chromatography to yield the desired carbamoyl chloride (single diastereomer) as a colourless oil. *Some compounds as colourless oils became solid after storage in the freezer.*

General Procedure 3 (GP3): *N*-Aryl urea formation from carbamoyl chlorides

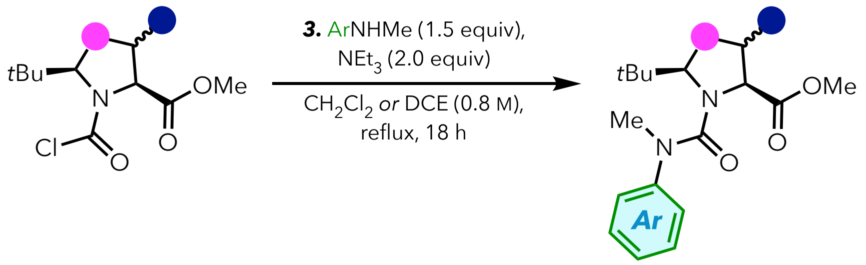

To a solution carbamoyl chloride (500-mg or 250-mg scale; 1.0 equiv.) in dichloromethane or 1,2-dichloroethane (0.8 M, HPLC grade) was added triethylamine (2.0 equiv.) and *N*-methylaniline (1.5 equiv.). The reaction mixture was heated to reflux (40 °C or 85 °C) for 18 h. The reaction mixture was cooled to ambient temperature and quenched with HCl (1 M, aq.), the organic layer was separated and the aqueous layer was extracted with CH_2Cl_2. The combined organic layers were washed with brine, dried over $MgSO_4$ and the solvent was removed under reduced pressure to give the crude mixture as an oil, which was purified by silica gel column chromatography to yield the urea.

General Procedure 4 (GP4): Rearrangement of *N*-aryl ureas

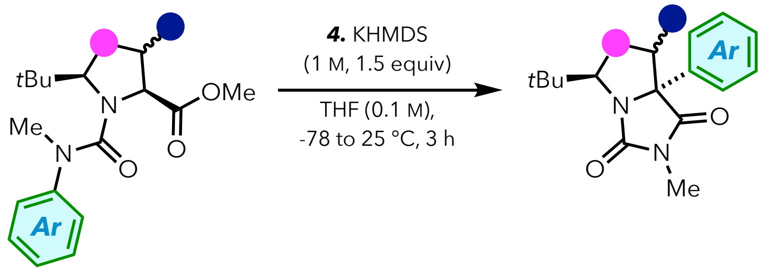

To a solution of *N*-aryl urea (100-mg scale; 1.0 equiv.) in dry tetrahydrofuran (0.1 M) at –78 °C was added a potassium bis(trimethylsilyl)amide solution (1 M in THF, 1.5 equiv.) dropwise upon which the solution change colour (formation of the enolate). The reaction mixture was left at –78 °C for 1 h and warmed to ambient temperature over 2 h. The reaction was quenched with HCl (1 M, aq.) and extracted with EtOAc. The combined organic layers were washed with brine, dried over $MgSO_4$ and the solvent was removed under reduced pressure. The crude product was purified by silica gel column chromatography to yield the rearranged product as a bicyclic hydantoin.

General Procedure 5 (GP5): Acidic hydrolysis of bicyclic hydantoins

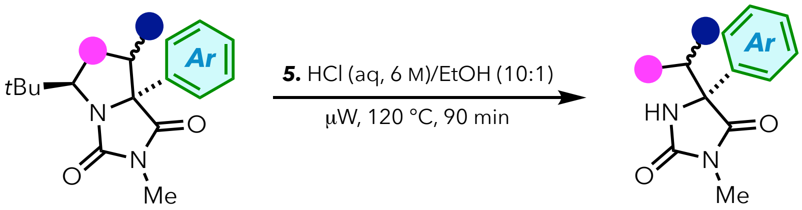

A solution of bicyclic hydantoin was suspended in a 6 M aqueous hydrochloric acid/ethanol-solution mixture (10:1 v/v) in a microwave vial and heated in a microwave reactor at 120 °C for 90 mins. After the reaction, the contents were transferred to a round-bottom flask and concentrated under reduced pressure. The crude product was then purified by silica gel column chromatography to afford the desired hydantoin.

General Procedure 6 (GP6): Basic hydrolysis of hydantoins to α-arylated amino acids

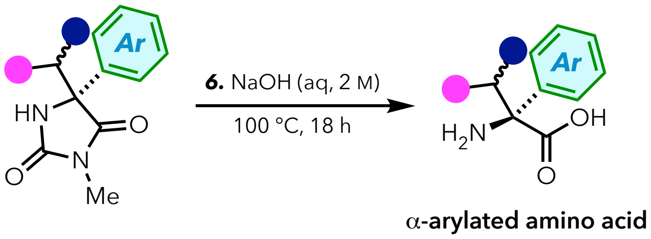

Hydantoin was dissolved in a water/dioxane mixture (5:1 v/v), and a 2 M aqueous sodium hydroxide solution was added. The reaction mixture was heated to reflux overnight, forming a homogenous solution. After completion, the mixture was acidified to pH 7 using concentrated aqueous HCl and extracted with EtOAc. The aqueous layer was concentrated under reduced pressure, yielding a white solid. The crude product was purified by silica gel column chromatography to afford the α-arylated amino acid.

4. Experimental Procedures and Data

4.1 Formation of the Heterocyclic Rings according to General Procedure 1 (GP1)

Methyl (4*S*)-2-(*tert*-butyl)oxazolidine-4-carboxylate (2)[1]

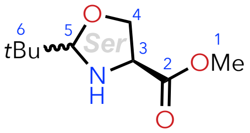

2

Following **GP1**, to a solution of L-serine methyl ester hydrochloride **1** (10.5 g, 64.3 mmol, 1.0 equiv.) and magnesium sulfate (7.75 g, 64.3 mmol, 1.0 equiv.) in CH_2Cl_2 (200 mL, 0.3 M) was added triethylamine (9.8 mL, 70.7 mmol, 1.1 equiv.) and pivaldehyde (10.5 mL, 96.5 mmol, 1.5 equiv.). The reaction mixture was stirred at ambient temperature for 18 h. The product **2** was obtained as a colourless oil (10.5 g, 56.1 mmol, 87%) as a mixture of diastereomers (3:2).

Formula: $C_9H_{17}NO_3$, **MW:** 187.24 g/mol. **TLC:** R_f = 0.54 (EA/CH_2Cl_2 6:1), $KMnO_4$ stain. **^{1}H NMR – Major diastereomer** (500 MHz, $CDCl_3$): δ [ppm] = 4.09 (s, 1H, H-5), 3.97 – 3.91 (m, 2H, H-3, H-4a), 3.77 (s, 3H, H-1, OMe), 3.74 – 3.70 (m, 1H, H-4b), 1.00 (s, 9H, *t*Bu). **^{1}H NMR – Minor diastereomer** (500 MHz, $CDCl_3$): δ [ppm] = 4.34 (s, 1H, H-5), 4.12 (dd, *J* 7.9, 7.4 Hz, 1H, H-3), 3.75 (s, 3H, H-1, OMe), 3.97 – 3.91 (m, 1H, H-4a), 3.74 – 3.70 (m, 1H, H-4b), 0.92 (s, 9H, *t*Bu). **^{13}C NMR – Major diastereomer** (125 MHz, $CDCl_3$): δ [ppm] = 173.0 (s, C-2, ester), 100.1 (d, C-5), 68.4 (t, C-4), 59.7 (d, C-3), 52.7 (q, C-1, OMe), 33.3 (s, C-6, *t*Bu), 25.4 (q, *t*Bu, 3Me). **^{13}C NMR – Major diastereomer** (125 MHz, $CDCl_3$): δ [ppm] = 173.2 (s, C-2, ester), 99.4 (d, C-5), 69.1 (t, C-4), 59.5 (d, C-3), 52.5 (q, C-1, OMe), 34.7 (s, C-6, *t*Bu), 25.0 (q, *t*Bu, 3Me). **FT-IR (ATR):** $\tilde{\nu}$ [cm^{-1}] = 2956 (br w), 1739 (vs), 1482 (w), 1435 (m), 1365 (w), 1332 (w), 1303 (w), 1205 (s), 1141 (w), 1032 (m), 913 (m), 935 (m), 838 (w), 674 (w), 474 (w), 405 (w). **HR-MS:** (ESI) = *m*/*z* calcd. for: $C_9H_{18}NO_3$ $[M+H]^+$ 188.1287 u, found: 188.1284 u.

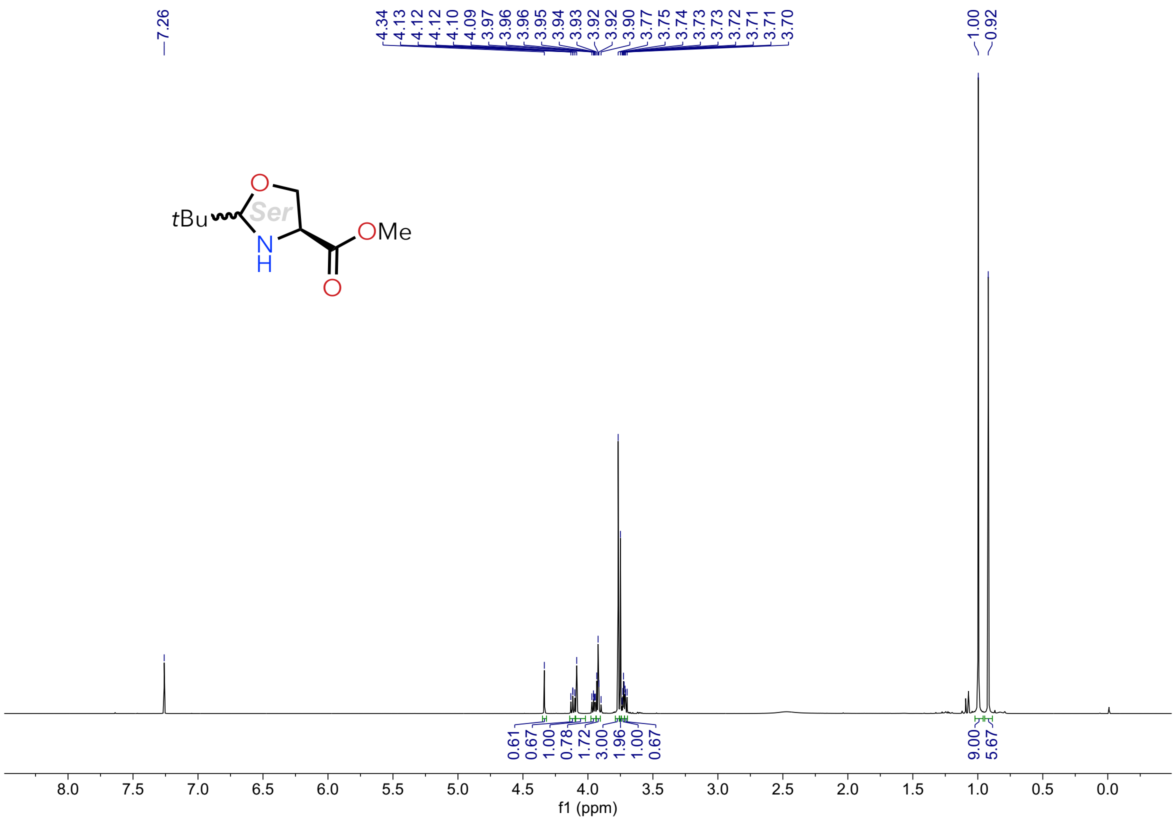
tBu
O
Ser
N
H
OMe
O
7.26
4.34
4.13
4.12
4.12
4.10
4.09
3.97
3.96
3.96
3.95
3.94
3.93
3.92
3.92
3.90
3.77
3.75
3.74
3.73
3.73
3.72
3.71
3.71
3.70
1.00
0.92
0.61
0.67
1.00
0.78
1.72
3.00
1.96
1.00
0.67
9.00
5.67
f1 (ppm)

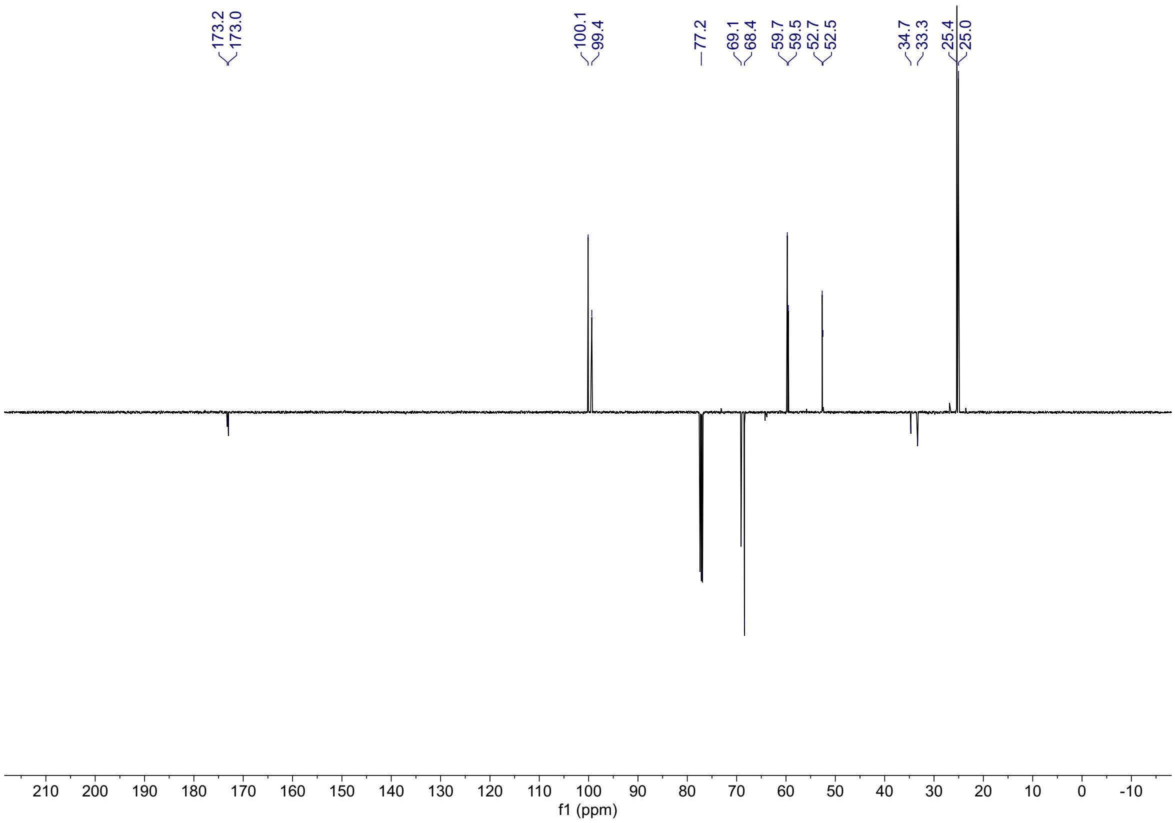
173.2
173.0
100.1
99.4
77.2
69.1
68.4
59.7
59.5
52.7
52.5
34.7
33.3
25.4
25.0
f1 (ppm)

Methyl (4*R*)-2-(*tert*-butyl)thiazolidine-4-carboxylate (11)

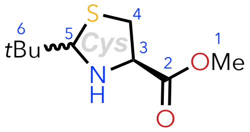

11

Following **GP1**, to a solution of L-cysteine methyl ester hydrochloride **7** (12 g, 69.9 mmol, 1.0 equiv.) and magnesium sulfate (8.41 g, 69.9 mmol, 1.0 equiv.) in CH_2Cl_2 (200 mL, 0.3 M) was added triethylamine (10.7 mL, 76.9 mmol, 1.1 equiv.) and pivaldehyde (11.4 mL, 104.9 mmol, 1.5 equiv.). The reaction mixture was stirred at ambient temperature for 18 h. The product **11** was obtained as a slightly yellow oil (13.7 g, 67.2 mmol, 96%) as a mixture of diastereomers (3:1).

Formula: $C_9H_{17}NO_2S$, **MW:** 203.30 g/mol. **TLC:** R_f = 0.63 (EA/CH_2Cl_2 6:1), $KMnO_4$ stain. **^{1}H NMR – Major diastereomer** (600 MHz, $CDCl_3$): δ [ppm] = 4.45 (s, 1H, H-5), 3.81 (dd, *J* 9.6, 6.7 Hz, 1H, H-3), 3.77 (s, 3H, H-1, OMe), 3.25 (dd, *J* 9.6, 6.7 Hz, 1H, H-4a), 2.68 (t, *J* 9.9 Hz, 1H, H-4b), 1.06 (s, 9H, *t*Bu). **^{1}H NMR – Minor diastereomer** (600 MHz, $CDCl_3$): δ [ppm] = 4.52 (s, 1H, H-5), 4.14 (t, *J* 6.0 Hz, 1H, H-3), 3.75 (s, 3H, H-1, OMe), 3.11 (dd, *J* 10.6, 6.4 Hz, 1H, H-4a), 3.02 (dd, *J* 10.6, 5.6 Hz, 1H, H-4b), 0.97 (s, 9H, *t*Bu). **^{13}C NMR – Major diastereomer** (150 MHz, $CDCl_3$): δ [ppm] = 171.9 (s, C-2, ester), 82.0 (d, C-5), 65.6 (d, C-3), 52.6 (q, C-1, OMe), 37.6 (t, C-4), 34.1 (s, C-6, *t*Bu), 27.1 (q, *t*Bu, 3Me). **^{13}C NMR – Major diastereomer** (150 MHz, $CDCl_3$): δ [ppm] = 172.6 (s, C-2, ester), 79.9 (d, C-5), 65.2 (d, C-3), 52.6 (q, C-1, OMe), 37.1 (t, C-4), 36.0 (s, C-6, *t*Bu), 26.7 (q, *t*Bu, 3Me). **FT-IR (ATR):** $\tilde{\nu}$ [cm^{-1}] = 2953 (br m), 1741 (vs), 1477 (w), 1435 (m), 1396 (w), 1364 (m), 1313 (m), 1258 (w), 1199 (m), 1179 (m), 1156 (m), 1111 (w), 1026 (w), 825 (m), 792 (m), 714 (w). **HR-MS:** (ESI) = *m*/*z* calcd. for: $C_9H_{18}NO_2S$ [M+H]$^+$ 204.1058 u, found: 204.1047 u.

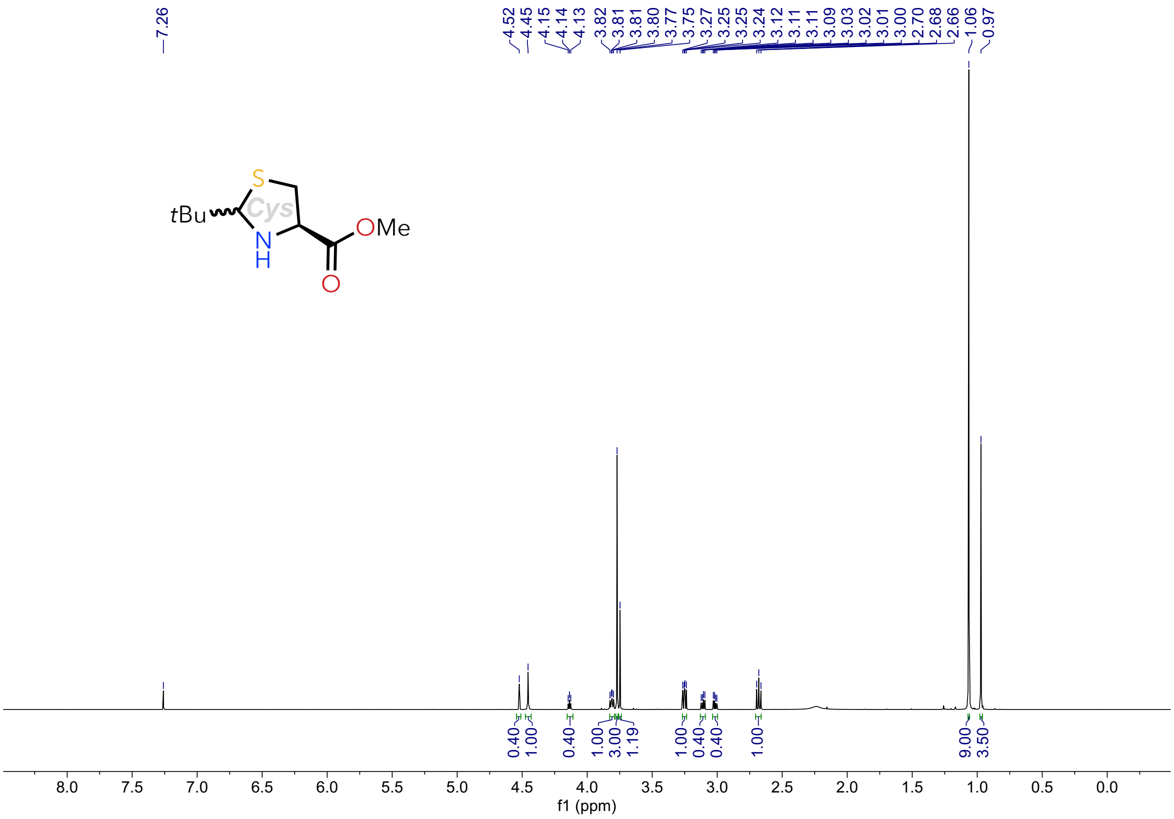
7.26
4.52
4.45
4.15
4.14
4.13
3.82
3.81
3.81
3.80
3.77
3.75
3.27
3.25
3.25
3.24
3.12
3.11
3.11
3.09
3.03
3.02
3.01
3.00
2.70
2.68
2.66
1.06
0.97
tBu
S
Cys
N
H
OMe
O
0.40
1.00
0.40
1.00
3.00
1.19
1.00
0.40
0.40
1.00
9.00
3.50
8.0 7.5 7.0 6.5 6.0 5.5 5.0 4.5 4.0 3.5 3.0 2.5 2.0 1.5 1.0 0.5 0.0
f1 (ppm)

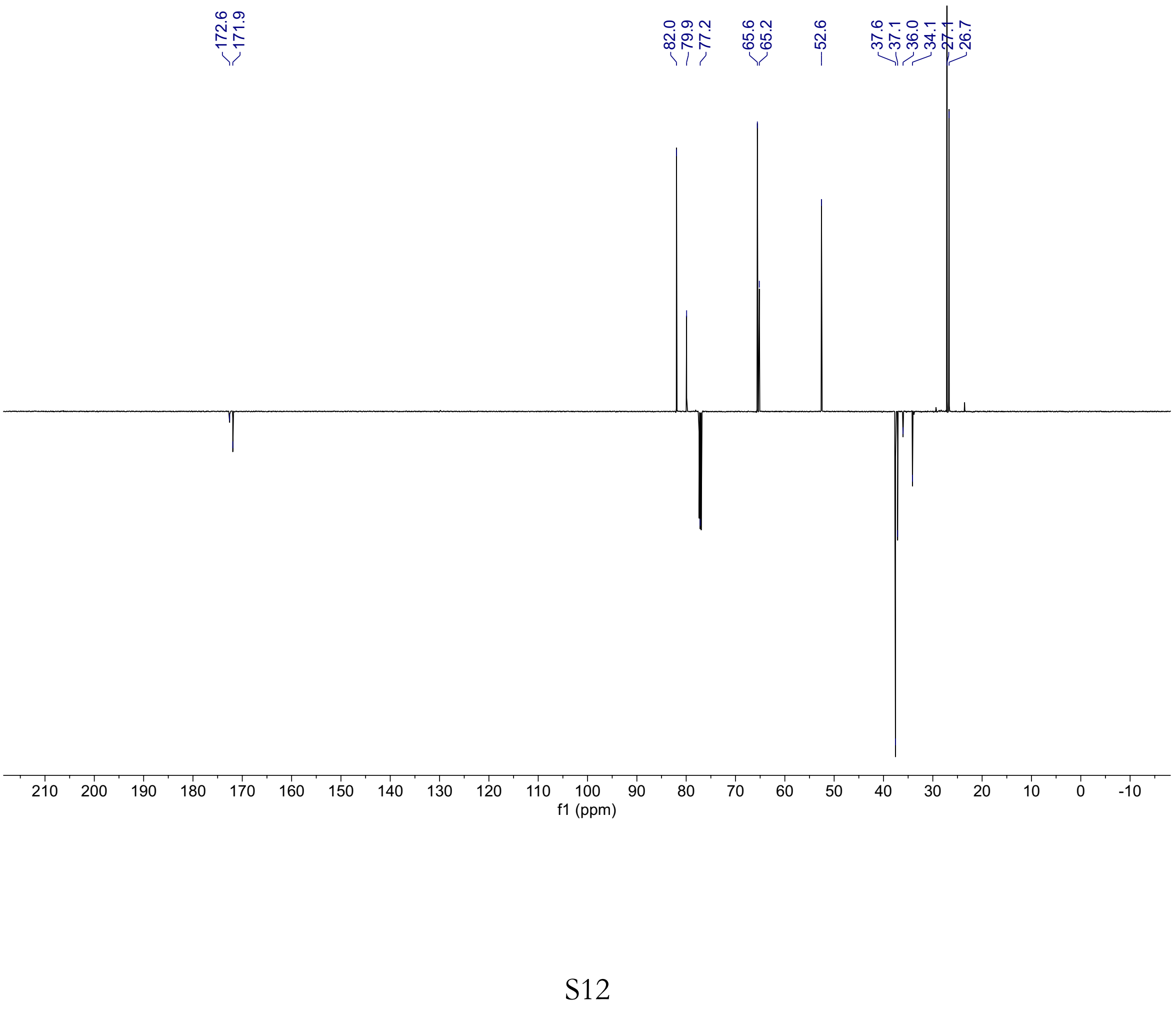
172.6
171.9
82.0
79.9
77.2
65.6
65.2
52.6
37.6
37.1
36.0
34.1
27.1
26.7
210 200 190 180 170 160 150 140 130 120 110 100 90 80 70 60 50 40 30 20 10 0 -10
f1 (ppm)

1-(*tert*-Butyl) 4-methyl (4*S*)-2-(*tert*-butyl)imidazolidine-1,4-dicarboxylatecarboxylate (12)

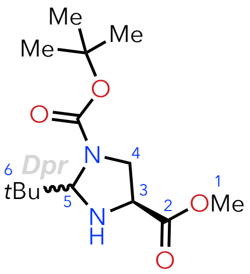

12

Following **GP1**, to a solution of *N*-beta-(*t*-Butyloxycarbonyl)-L-2,3-diaminopropionic acid methyl ester hydrochloride **8** (1.5 g, 5.89 mmol, 1.0 equiv.) and magnesium sulfate (0.71 g, 5.89 mmol, 1.0 equiv.) in CH_2Cl_2 (20 mL, 0.3 M) was added triethylamine (0.90 mL, 6.48 mmol, 1.1 equiv.) and pivaldehyde (0.96 mL, 8.83 mmol, 1.5 equiv.). The reaction mixture was stirred at ambient temperature for 18 h. A mixture of diastereomers (4.5:1) of the product **12** was obtained as a colourless oil (1.39 g, 4.85 mmol, 82%), which was used without further purification.

Formula: $C_{14}H_{26}N_2O_4$, **MW:** 286.37 g/mol. **TLC:** R_f = 0.30 (PE/EA 7:3), $KMnO_4$ stain. **FT-IR (ATR):** $\tilde{\nu}$ [cm^{-1}] = 2956 (br w), 1741 (w), 1694 (s), 1515 (w), 1365 (s), 1249 (m), 1160 (vs), 1021 (w), 909 (w), 858 (w), 776 (w). **HR-MS:** (ESI) = *m*/*z* calcd. for: $C_{14}H_{27}N_2O_4$ $[M+H]^+$ 287.1971 u, found: 287.1957 u.

Methyl (4*S*,5*R*)-2-(*tert*-butyl)-5-methyloxazolidine-4-carboxylate (13)

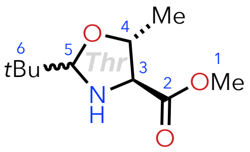

13

Following **GP1**, to a solution of L-threonine methyl ester hydrochloride **9** (5 g, 29.5 mmol, 1.0 equiv.) and magnesium sulfate (3.55 g, 29.5 mmol, 1.0 equiv.) in CH_2Cl_2 (100 mL, 0.3 M) was added triethylamine (4.5 mL, 32.4 mmol, 1.1 equiv.) and pivaldehyde (4.8 mL, 44.2 mmol, 1.5 equiv.). The reaction mixture was stirred at ambient temperature for 18 h. The product **13** was obtained as a colourless oil (4.68 g, 23.2 mmol, 79%) as a mixture of diastereomers (3:1).

Formula: $C_{10}H_{19}NO_3$, **MW:** 201.27 g/mol. **TLC:** R_f = 0.62 (EA/CH_2Cl_2 6:1), $KMnO_4$ stain. **^{1}H NMR – Major diastereomer** (600 MHz, $CDCl_3$): δ [ppm] = 4.21 (dq, *J* 8.2, 6.3 Hz, 1H, H-3), 4.07 (s, 1H, H-5), 3.94 (d, *J* 8.2 Hz, 1H, H-4), 3.74 (s, 3H, H-1, OMe), 1.06 (d, *J* 6.3 Hz, 3H, Me), 0.99 (s, 9H, *t*Bu). **^{1}H NMR – Minor diastereomer** (600 MHz, $CDCl_3$): δ [ppm] = 4.59 (s, 1H, H-5), 4.39 (p, *J* 6.4 Hz, 1H, H-3), 3.96 (d, *J* 6.6 Hz, 1H, H-4), 3.73 (s, 3H, H-1, OMe), 1.05 (d, *J* 10.1 Hz, 3H, Me), 0.87 (s, 9H, *t*Bu). **^{13}C NMR – Major diastereomer** (150 MHz, $CDCl_3$): δ [ppm] = 171.8 (s, C-2, ester), 98.7 (d, C-5), 73.5 (d, C-4), 63.5 (d, C-3), 52.2 (q, C-1, OMe), 33.0 (s, C-6, *t*Bu), 25.4 (q, *t*Bu, 3Me), 16.9 (q, Me). **^{13}C NMR – Major diastereomer** (150 MHz, $CDCl_3$): δ [ppm] = 171.9 (s, C-2, ester), 98.3 (d, C-5), 74.7 (d, C-4), 63.4 (d, C-3), 52.1 (q, C-1, OMe), 35.8 (s, C-6, *t*Bu), 24.9 (q, *t*Bu, 3Me), 16.1 (q, Me). **FT-IR (ATR):** $\tilde{\nu}$ [cm^{-1}] = 2956 (br w), 1740 (vs), 1482 (w), 1435 (w), 1364 (w), 1324 (m), 1304 (m), 1204 (s), 1142 (s), 1034 (m), 981 (m), 926 (m), 841 (w), 763 (w), 674 (w), 451 (w). **HR-MS:** (ESI) = *m/z* calcd. for: $C_{10}H_{20}NO_3$ $[M+H]^+$ 202.1443 u, found: 202.1434 u.

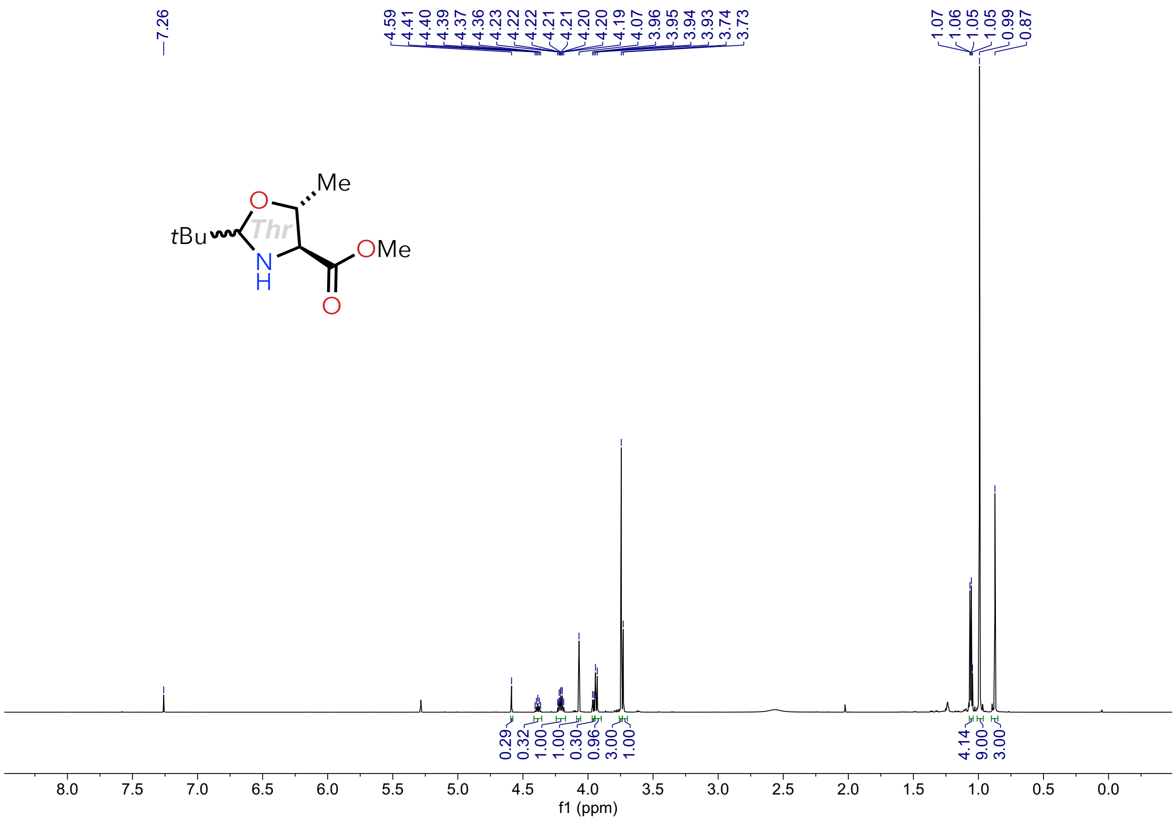
7.26
4.59 4.41 4.40 4.39 4.37 4.36 4.23 4.22 4.22 4.21 4.21 4.20 4.20 4.19 4.07 3.96 3.95 3.94 3.93 3.74 3.73
1.07 1.06 1.05 1.05 0.99 0.87
Me
O
tBu
Thr
N
H
OMe
O
0.29 0.32 1.00 1.00 0.30 0.96 3.00 1.00
4.14 9.00 3.00
f1 (ppm)

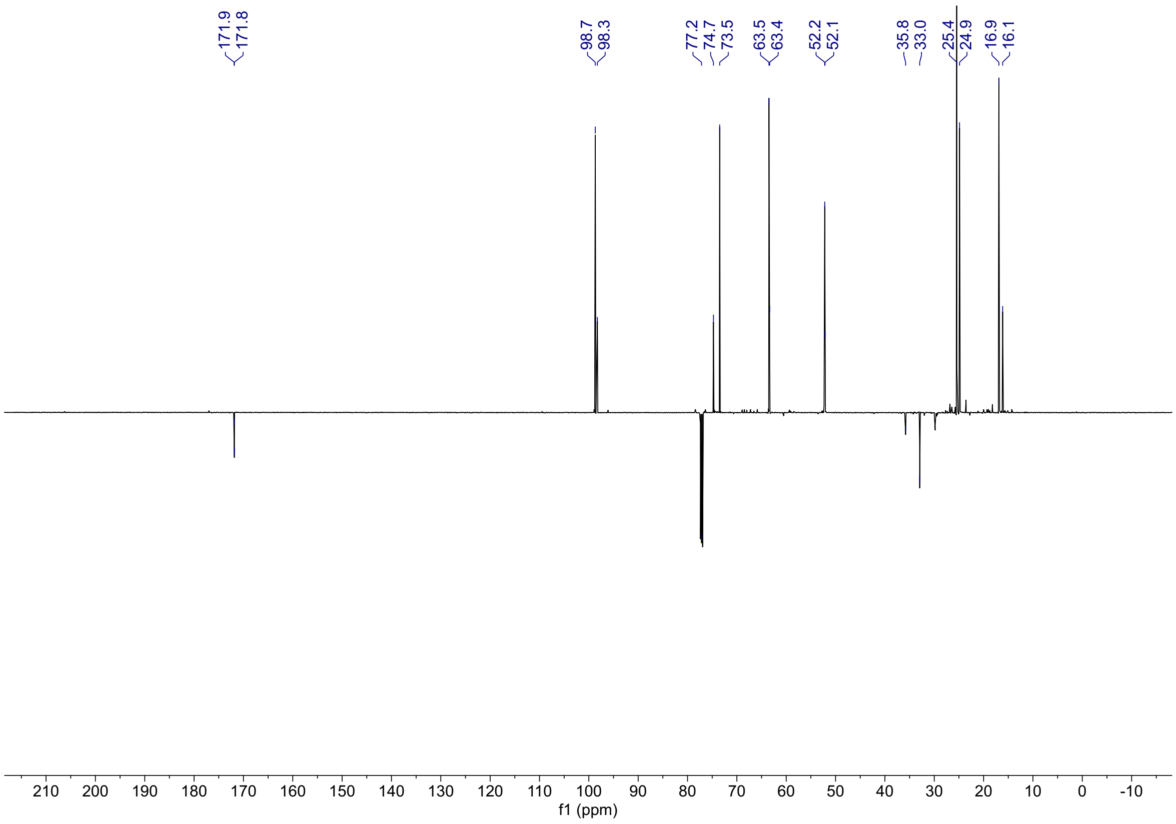
171.9 171.8
98.7 98.3
77.2 74.7 73.5
63.5 63.4
52.2 52.1
35.8 33.0
25.4 24.9
16.9 16.1
f1 (ppm)

Methyl (4*S*,5*S*)-2-(*tert*-butyl)-5-methyloxazolidine-4-carboxylate (14)

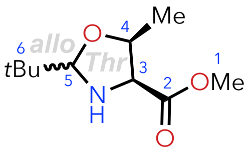

14

Following **GP1**, to a solution of L-allo-threonine methyl ester hydrochloride **10** (1.04 g, 6.11 mmol, 1.0 equiv.) and magnesium sulfate (0.74 g, 6.11 mmol, 1.0 equiv.) in CH_2Cl_2 (20 mL, 0.3 M) was added triethylamine (0.94 mL, 6.72 mmol, 1.1 equiv.) and pivaldehyde (0.79 mL, 9.16 mmol, 1.5 equiv.). The reaction mixture was stirred at ambient temperature for 18 h. The product **14** was obtained as a colourless oil (1.18 g, 5.86 mmol, 96%) as a mixture of diastereomers (1:1).

Formula: $C_{10}H_{19}NO_3$, **MW:** 201.27 g/mol. **TLC:** R_f = 0.52 (EA/CH_2Cl_2 6:1), $KMnO_4$ stain. **^{1}H NMR – Diastereomer 1** (600 MHz, $CDCl_3$): δ [ppm] = 4.38 (s, 1H, H-5), 3.84 – 3.77 (m, 1H, H-3), 3.75 (s, 3H, H-1, OMe), 3.36 (d, *J* 7.3 Hz, 1H, H-4), 1.37 (d, *J* 6.0 Hz, 3H, Me), 0.90 (s, 9H, *t*Bu). **^{1}H NMR – Diastereomer 2** (600 MHz, $CDCl_3$): δ [ppm] = 4.29 (s, 1H, H-5), 3.85 (dd, *J* 7.3, 6.0 Hz, 1H, H-3), 3.77 (s, 3H, H-1, OMe), 3.46 (d, *J* 7.2 Hz, 1H, H-4), 1.33 (d, *J* 6.1 Hz, 3H, Me), 0.97 (s, 9H, *t*Bu). **^{13}C NMR – Diastereomer 1** (150 MHz, $CDCl_3$): δ [ppm] = 172.8 (s, C-2, ester), 98.3 (d, C-5), 77.4 (d, C-4), 67.2 (d, C-3), 52.6 (q, C-1, OMe), 33.7 (s, C-6, *t*Bu), 25.3 (q, *t*Bu, 3Me), 19.0 (q, Me). **^{13}C NMR – Diastereomer 2** (150 MHz, $CDCl_3$): δ [ppm] = 172.1 (s, C-2, ester), 99.0 (d, C-5), 76.4 (d, C-4), 65.9 (d, C-3), 52.5 (q, C-1, OMe), 34.7 (s, C-6, *t*Bu), 24.9 (q, *t*Bu, 3Me), 20.0 (q, Me). **HR-MS:** (ESI) = *m/z* calcd. for: $C_{10}H_{20}NO_3$ $[M+H]^+$ 202.1443 u, found: 202.1434 u.

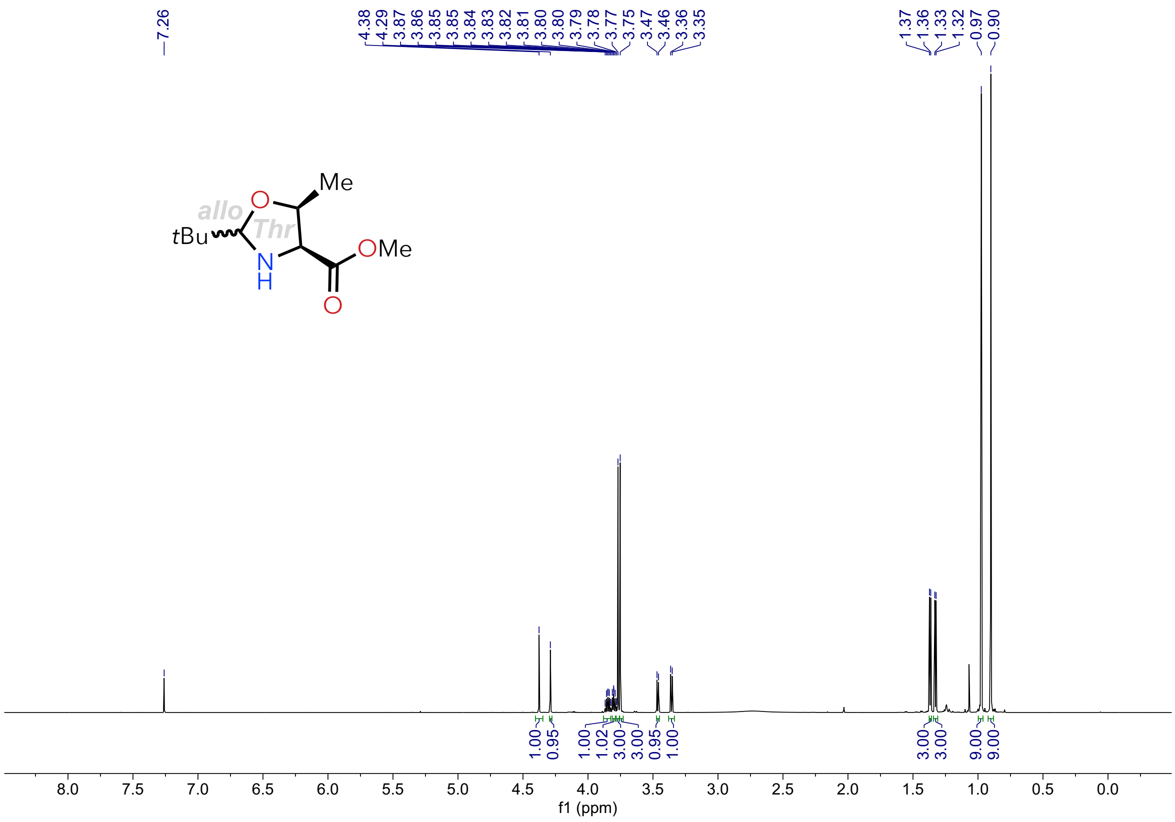

7.26
4.38
4.29
3.87
3.86
3.85
3.85
3.84
3.83
3.82
3.81
3.80
3.80
3.79
3.78
3.77
3.75
3.47
3.46
3.36
3.35
1.37
1.36
1.33
1.32
0.97
0.90
Me
allo
O
Thr
tBu
N
H
OMe
O
1.00
0.95
1.00
1.02
3.00
3.00
0.95
1.00
3.00
3.00
9.00
9.00
f1 (ppm)

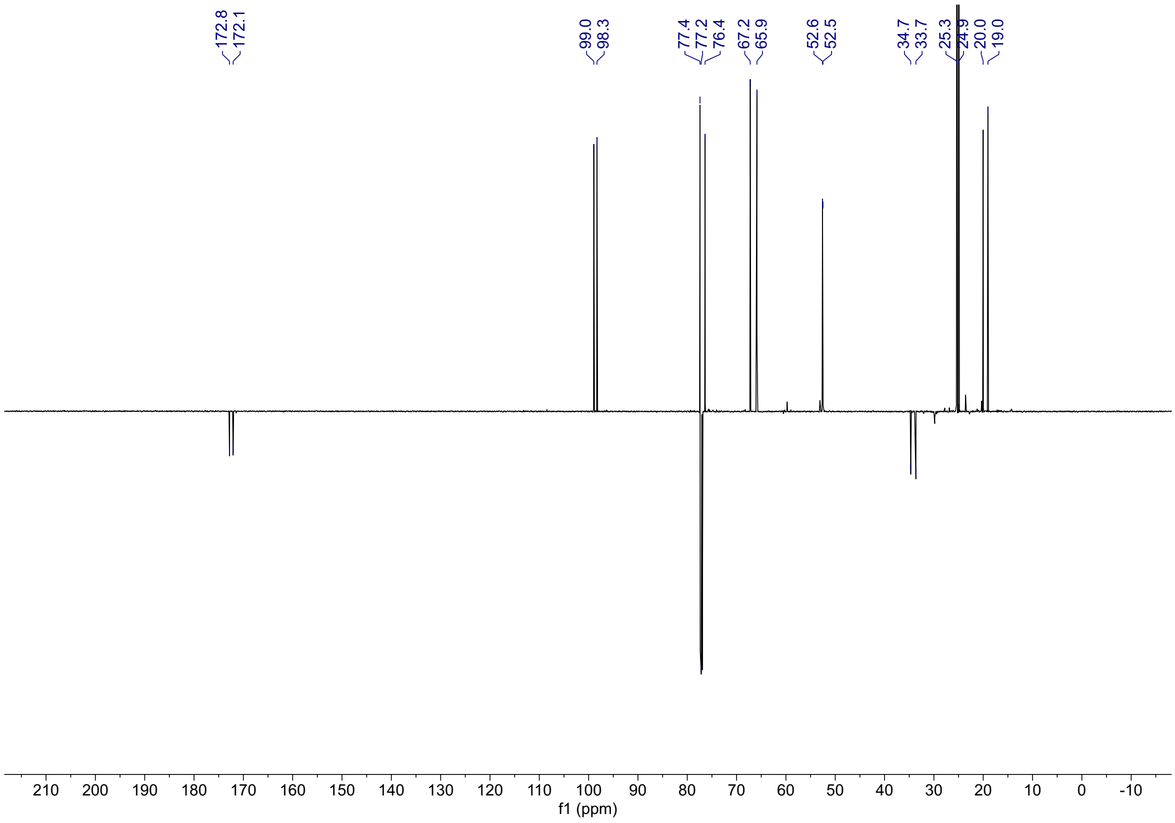

172.8
172.1
99.0
98.3
77.4
77.2
76.4
67.2
65.9
52.6
52.5
34.7
33.7
25.3
24.9
20.0
19.0
f1 (ppm)

4.2 Formation of the Carbamoyl Chlorides according to General Procedure 2 (GP2)

Methyl (2*R*,4*S*)-2-(*tert*-butyl)-3-(chlorocarbonyl)oxazolidine-4-carboxylate (3)[1]

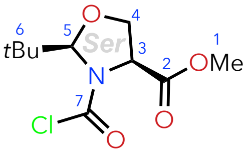

3

Following **GP2**, to a solution of crude oxazolidine **2** (10.5 g, 56.1 mmol, 1.0 equiv.) in dry CH_2Cl_2 (180 mL, 0.3 M) was added a phosgene solution (60 mL, 84.1 mmol, 1.5 equiv., 15 wt. % in toluene) and triethylamine (10.1 mL, 72.9 mmol, 1.3 equiv.) at –15 °C. The reaction mixture was stirred and allowed to warm to ambient temperature over 1 h and left for a further 2 h. The product **3** was obtained as a white solid (13.8 g, 55.3 mmol, 99%, single diastereomer) after purification by silica gel column chromatography.

Formula: $C_{10}H_{16}ClNO_4$, **MW:** 249.69 g/mol, **m.p.:** 77 – 81 °C. **TLC:** R_f = 0.57 (PE/EA 7:3), $KMnO_4$ stain. **^{1}H NMR** (400 MHz, $CDCl_3$): δ [ppm] = 5.18 (br s, 1H, H-5), 4.90 (dd, *J* 8.0, 4.5 Hz, 1H, H-3), 4.40 (dd, *J* 8.9, 4.5 Hz, 1H, H-4a), 4.21 (t, *J* 8.4 Hz, 1H, H-4b), 3.81 (s, 3H, H-1, OMe), 0.97 (s, 9H, *t*Bu). **^{13}C NMR** (100 MHz, $CDCl_3$): δ [ppm] = 169.2 (s, C-2, ester), 149.4 (s, C-7, carbamoyl), 99.6 (d, C-5), 68.4 (t, C-4), 62.3 (d, C-3), 53.0 (q, C-1, OMe), 38.1 (s, C-6, *t*Bu), 25.8 (q, *t*Bu, 3Me). **FT-IR (ATR):** $\tilde{\nu}$ [cm^{-1}] = 2962 (br w), 1726 (vs), 1483 (w), 1436 (m), 1364 (m), 1265 (s), 1217 (m), 1162 (s), 1103 (m), 1024 (m), 980 (m), 963 (m), 837 (m), 751 (s), 671 (m), 496 (m), 476 (w). **$[a]_{\lambda}^{T}$:** (c = 1.05 g/100 mL, $CHCl_3$) = $[a]_{D}^{20}$: –3.43°.

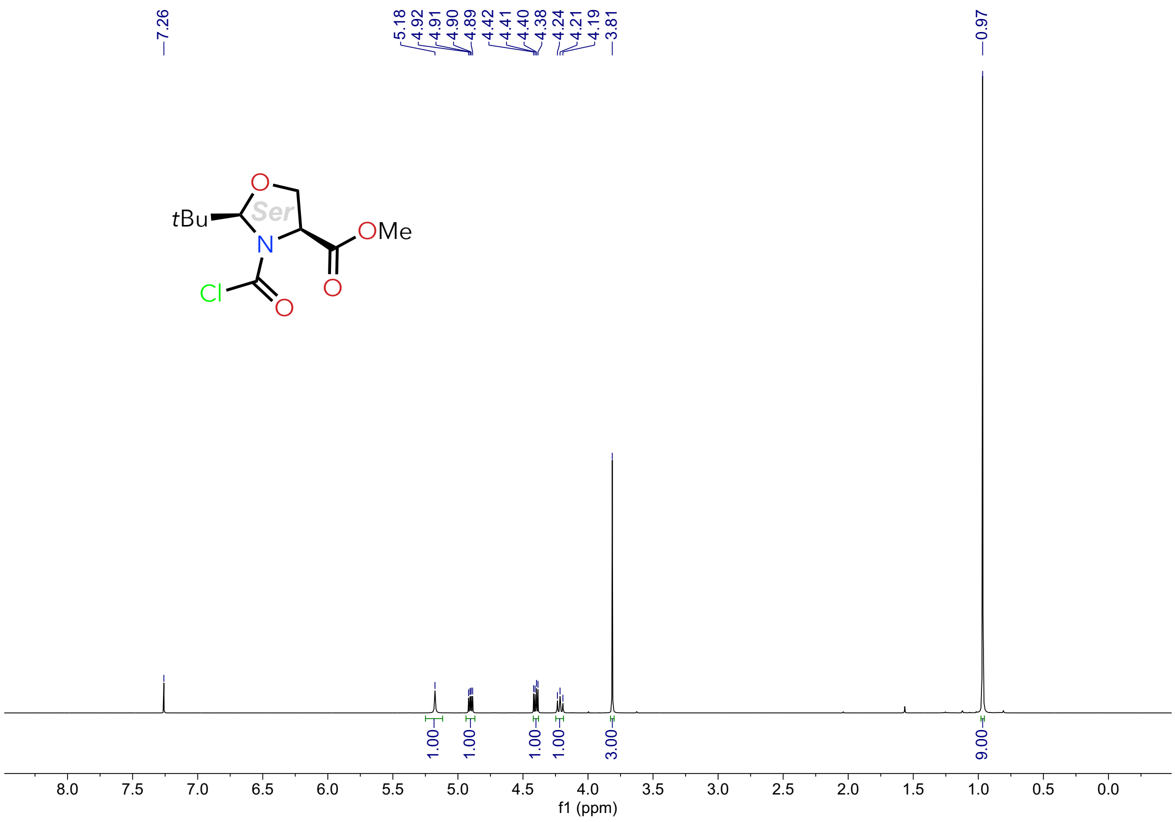
7.26
5.18
4.92
4.91
4.90
4.89
4.42
4.41
4.40
4.38
4.24
4.21
4.19
3.81
0.97
tBu
O
N
Ser
OMe
O
Cl
O
1.00
1.00
1.00
1.00
3.00
9.00
8.0 7.5 7.0 6.5 6.0 5.5 5.0 4.5 4.0 3.5 3.0 2.5 2.0 1.5 1.0 0.5 0.0
f1 (ppm)

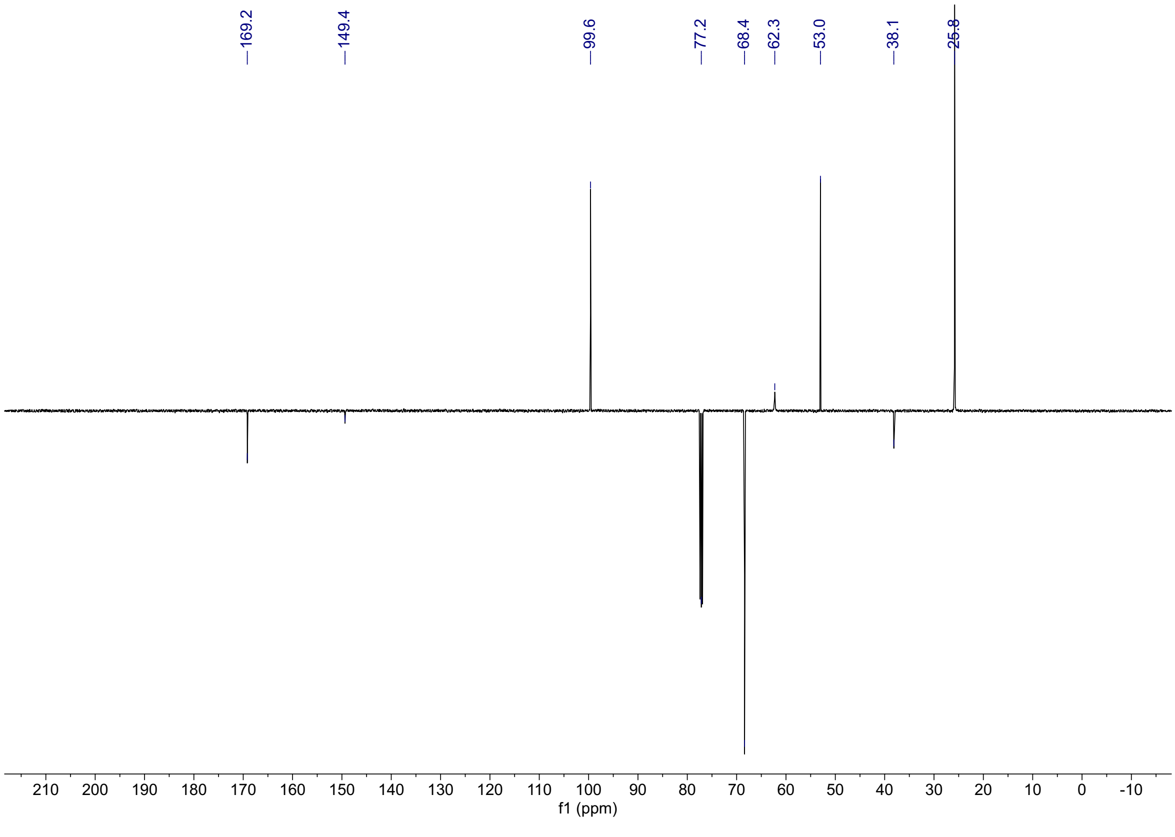
169.2
149.4
99.6
77.2
68.4
62.3
53.0
38.1
25.8
210 200 190 180 170 160 150 140 130 120 110 100 90 80 70 60 50 40 30 20 10 0 -10
f1 (ppm)

Methyl (2*R*,4*R*)-2-(*tert*-butyl)-3-(chlorocarbonyl)thiazolidine-4-carboxylate (15)

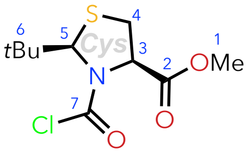

15

Following **GP2**, to a solution of crude thiazolidine **11** (13.5 g, 66.3 mmol, 1.0 equiv.) in dry CH_2Cl_2 (200 mL, 0.3 M) was added a phosgene solution (71.3 mL, 99.5 mmol, 1.5 equiv., 15 wt. % in toluene) and triethylamine (10.1 mL, 72.9 mmol, 1.3 equiv.) at –15 °C. The title compound **15** was obtained as a white solid (11.7 g, 44.0 mmol, 66%, single diastereomer) after purification by silica gel column chromatography (PE/EA, gradient elution).

Formula: $C_{10}H_{16}ClNO_3S$, **MW:** 265.75 g/mol, **m.p.:** 54 – 57 °C. **TLC:** R_f = 0.62 (PE/EA 7:3), $KMnO_4$ stain. **^{1}H NMR** (600 MHz, $CDCl_3$): δ [ppm] = 5.19 (br s, 1H, H-5), 5.00 (dd, *J* 9.0 Hz, 1H, H-3), 3.82 (s, 3H, H-1, OMe), 3.44 – 3.36 (m, 1H, H-4a), 3.33 – 3.25 (m, 1H, H-4b), 1.06 (s, 9H, *t*Bu). **^{13}C NMR** (150 MHz, $CDCl_3$): δ [ppm] = 170.2 (s, C-2, ester), 149.8 (s, C-7, carbamoyl), 76.6 (d, C-5), 67.4 (d, C-3), 53.1 (q, C-1, OMe), 68.4 (t, C-4), 40.3 (s, C-6, *t*Bu), 33.8 (t, C-4), 26.9 (q, *t*Bu, 3Me). **FT-IR (ATR):** $\tilde{\nu}$ [cm^{-1}] = 2963 (br w), 1722 (vs), 1430 (m), 1361 (m), 1245 (s), 1211 (s), 1172 (vs), 1126 (m), 999 (m), 873 (m), 769 (m), 699 (m), 669 (w), 608 (w), 488 (m). **$[a]_\lambda^T$:** (c = 1.03 g/100 mL, $CHCl_3$) = $[a]_D^{20}$: –10.49°.

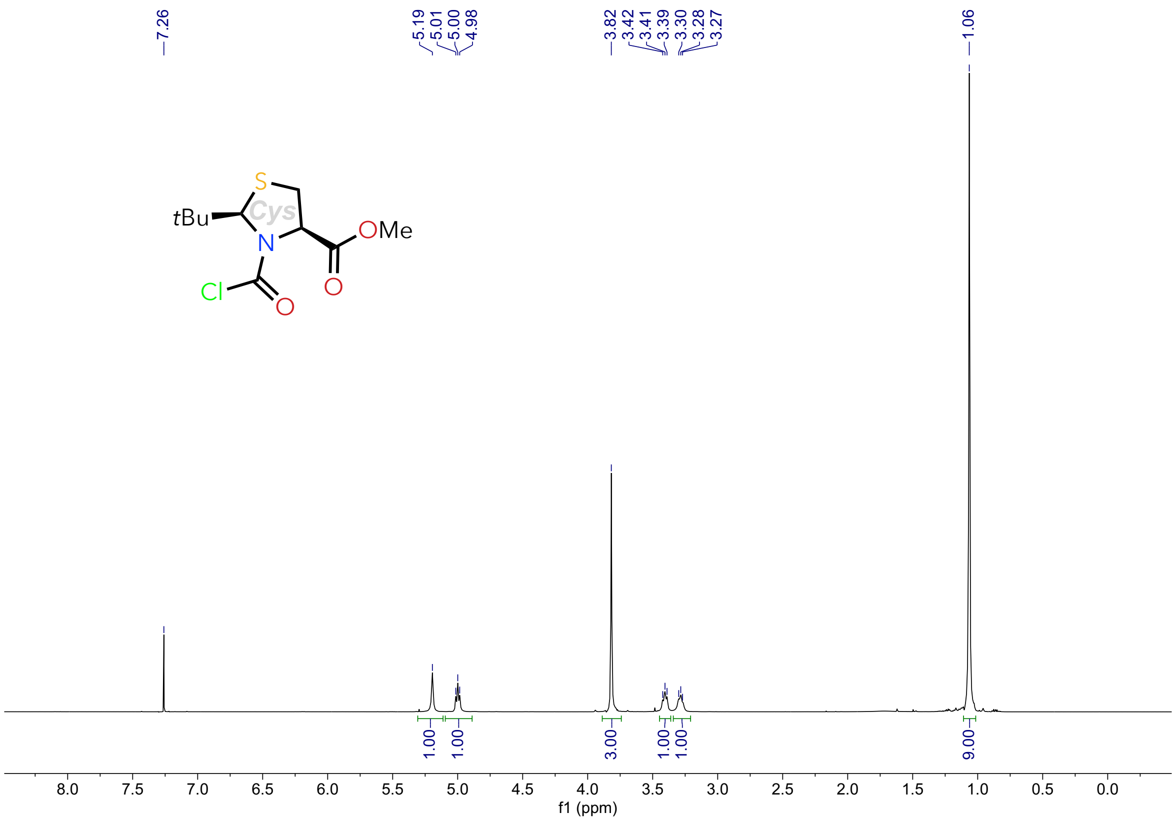
7.26
5.19
5.01
5.00
4.98
3.82
3.42
3.41
3.39
3.30
3.28
3.27
1.06
S
tBu
Cys
N
OMe
O
Cl
O
1.00
1.00
3.00
1.00
1.00
9.00
8.0
7.5
7.0
6.5
6.0
5.5
5.0
4.5
4.0
3.5
3.0
2.5
2.0
1.5
1.0
0.5
0.0
f1 (ppm)

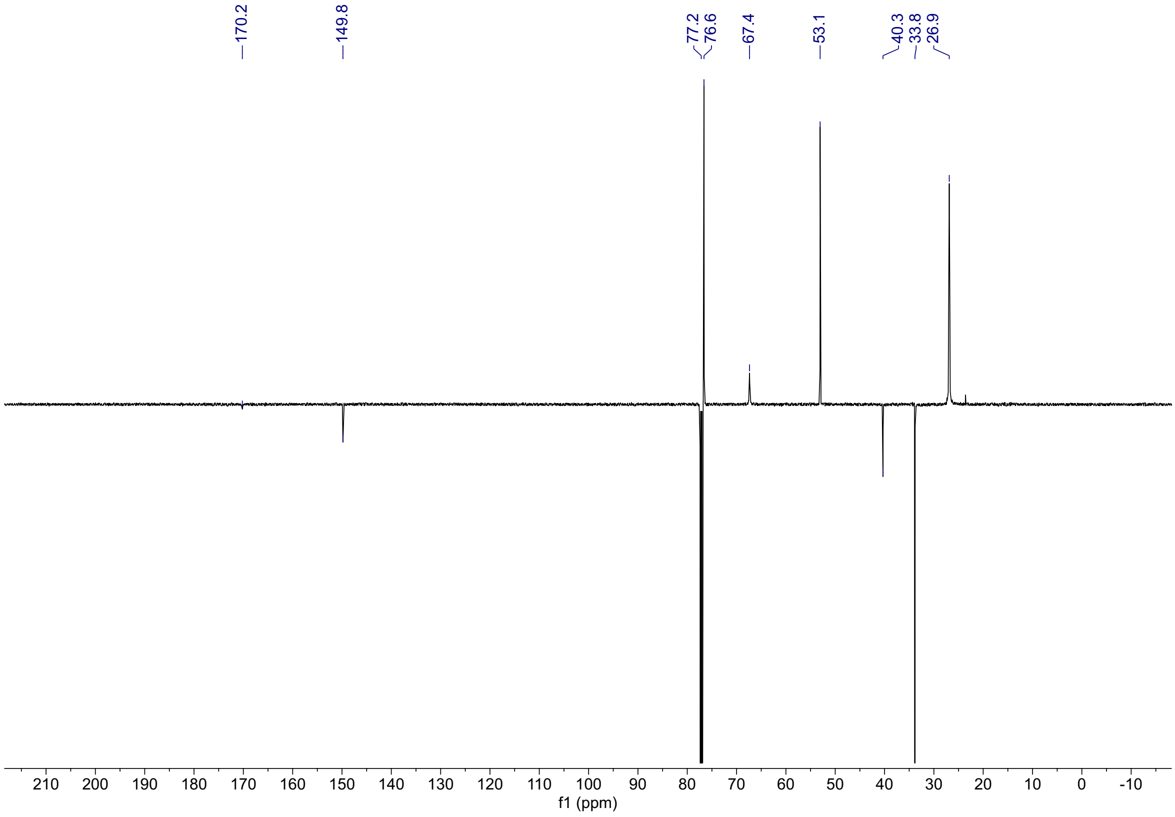
170.2
149.8
77.2
76.6
67.4
53.1
40.3
33.8
26.9
210
200
190
180
170
160
150
140
130
120
110
100
90
80
70
60
50
40
30
20
10
0
-10
f1 (ppm)

1-(*tert*-Butyl) 4-methyl (2*S*,4*S*)-2-(*tert*-butyl)-3-(chlorocarbonyl)imidazolidine-1,4-dicarboxylate (16)

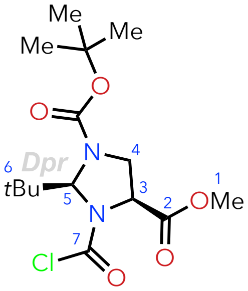

16

Following **GP2**, to a solution of crude imidazolidine **12** (1.39 g, 4.85 mmol, 1.0 equiv.) in dry CH_2Cl_2 (24 mL, 0.3 M) was added a phosgene solution (4.8 mL, 7.28 mmol, 1.5 equiv., 15 wt. % in toluene) and triethylamine (0.87 mL, 6.31 mmol, 1.3 equiv.) at –15 °C. The title compound **16** was obtained as a colourless oil (1.26 g, 3.61 mmol, 74%, single diastereomer) after purification by silica gel column chromatography (PE/EA, gradient elution).

Formula: $C_{15}H_{25}ClN_2O_5$, **MW:** 348.82 g/mol. **TLC:** R_f = 0.49 (EA/CH_2Cl_2 6:1), $KMnO_4$ stain. **^{1}H NMR** (600 MHz, $CDCl_3$): δ [ppm] = 5.58 (br s, 1H, H-5), 4.81 (dd, *J* 10.3, 8.1 Hz, 1H, H-3), 4.59 (br s, 1H, H-4a), 3.82 (s, 3H, H-1, OMe), 3.46 (br s, 1H, H-4b), 1.48 (s, 9H, Boc, *t*Bu), 1.01 (s, 9H, *t*Bu). **^{13}C NMR** (150 MHz, $CDCl_3$): δ [ppm] = 168.3 (s, C-2, ester), 153.6 (s, Boc), 149.6 (s, C-7, carbamoyl), 82.1 (s, Boc, *t*Bu), 81.8 (d, C-5), 61.4 (d, C-3), 53.0 (q, C-1, OMe), 47.3 (d, C-4), 39.8 (s, C-6, *t*Bu), 28.4 (q, Boc, *t*Bu, 3Me), 26.7 (q, *t*Bu, 3Me). **FT-IR (ATR):** $\tilde{\nu}$ [cm^{-1}] = 2958 (br w), 1753 (s), 1738 (s), 1705 (vs), 1366 (s), 1265 (m), 1212 (m), 1156 (vs), 1107 (m), 1033 (m), 990 (w), 912 (m), 869 (w), 812 (w), 759 (m), 674 (w), 627 (w), 577 (w), 460 (w). **HR-MS:** (ESI) = *m*/*z* calcd. for: $C_{15}H_{25}{}^{35}ClN_2O_5Na$ $[M+Na]^+$ 371.1350 u, found: 371.1341 u. **$[a]_\lambda^T$:** (c = 1.02 g/100 mL, $CHCl_3$) = $[a]_D^{20}$: –4.71°.

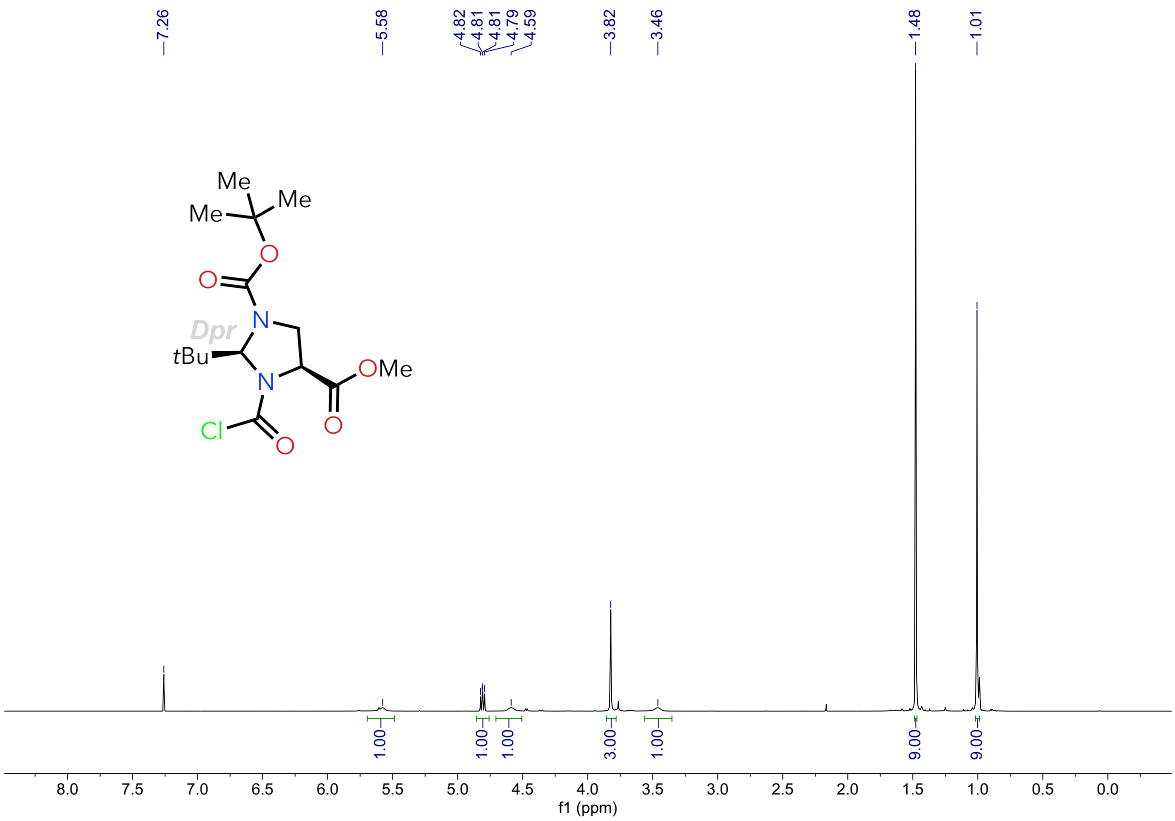

7.26
5.58
4.82
4.81
4.81
4.79
4.59
3.82
3.46
1.48
1.01
Me
Me
Me
O
O
N
Dpr
tBu
N
OMe
O
Cl
O
1.00
1.00
1.00
3.00
1.00
9.00
9.00
8.0
7.5
7.0
6.5
6.0
5.5
5.0
4.5
4.0
3.5
3.0
2.5
2.0
1.5
1.0
0.5
0.0
f1 (ppm)

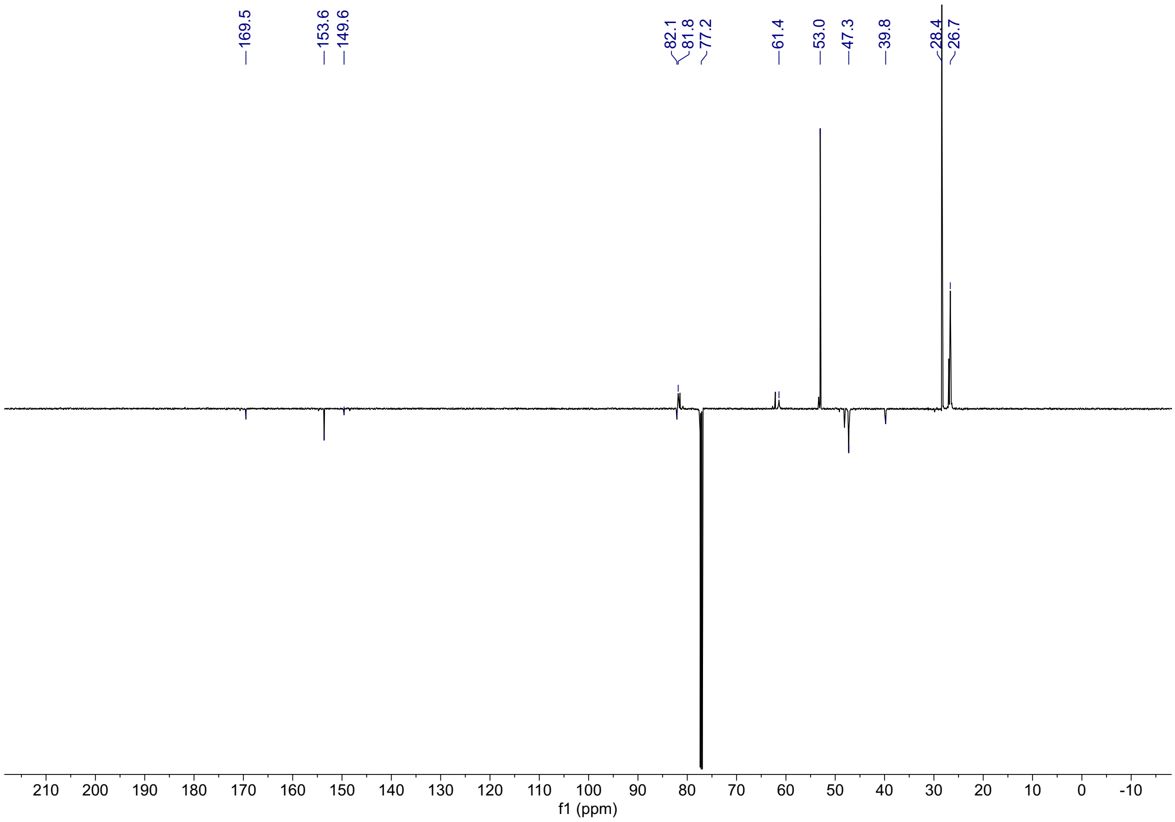

169.5
153.6
149.6
82.1
81.8
77.2
61.4
53.0
47.3
39.8
28.4
26.7
210
200
190
180
170
160
150
140
130
120
110
100
90
80
70
60
50
40
30
20
10
0
-10
f1 (ppm)

Methyl (2*R*,4*S*,5*R*)-2-(*tert*-butyl)-3-(chlorocarbonyl)-5-methyloxazolidine-4-carboxylate (17)

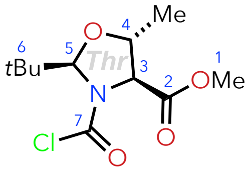

17

Following **GP2**, to a solution of crude oxazolidine **13** (4.68 g, 23.2 mmol, 1.0 equiv.) in dry CH_2Cl_2 (75 mL, 0.3 M) was added a phosgene solution (25 mL, 34.9 mmol, 1.5 equiv., 15 wt. % in toluene) and triethylamine (4.19 mL, 30.2 mmol, 1.3 equiv.) at –15 °C. The title compound **17** was obtained as a sticky white solid (4.97 g, 18.9 mmol, 81%, single diastereomer) after purification by silica gel column chromatography (PE/EA, gradient elution).

Formula: $C_{11}H_{18}ClNO_4$, **MW:** 263.72 g/mol, **m.p.:** 45 – 48 °C. **TLC:** R_f = 0.55 (PE/EA 7:3), $KMnO_4$ stain. **^{1}H NMR** (500 MHz, $CDCl_3$): δ [ppm] = 5.24 (br s, 1H, H-5), 4.61 – 4.54 (m, 1H, H-3), 4.34 (d, *J* 6.7 Hz, 1H, H-4), 3.82 (s, 3H, H-1, OMe), 1.44 (d, *J* 6.1 Hz, 3H, Me), 0.99 (s, 9H, *t*Bu). **^{13}C NMR** (125 MHz, $CDCl_3$): δ [ppm] = 169.1 (s, C-2, ester), 149.3 (s, C-7, carbamoyl), 99.4 (d, C-5), 76.8 (d, C-3), 68.8 (d, C-4), 53.0 (q, C-1, OMe), 38.8 (s, C-6, *t*Bu), 26.1 (q, *t*Bu, 3Me), 20.3 (q, Me). **FT-IR (ATR):** $\tilde{\nu}$ [cm^{-1}] = 2960 (br w), 1744 (vs), 1484 (w), 1438 (w), 1364 (m), 1325 (w), 1281 (m), 1209 (m), 1175 (s), 1114 (m), 1059 (m), 1003 (m), 980 (w), 890 (w), 810 (m), 754 (m), 671 (w), 548 (w). **HR-MS:** (ESI) = *m/z* calcd. for: $C_{11}H_{18}{}^{35}ClNO_4Na$ $[M+Na]^+$ 286.0822 u, found: 286.0818 u. **$[a]_\lambda^T$:** (c = 1.05 g/100 mL, $CHCl_3$) = $[a]_D^{20}$: –1.90°.

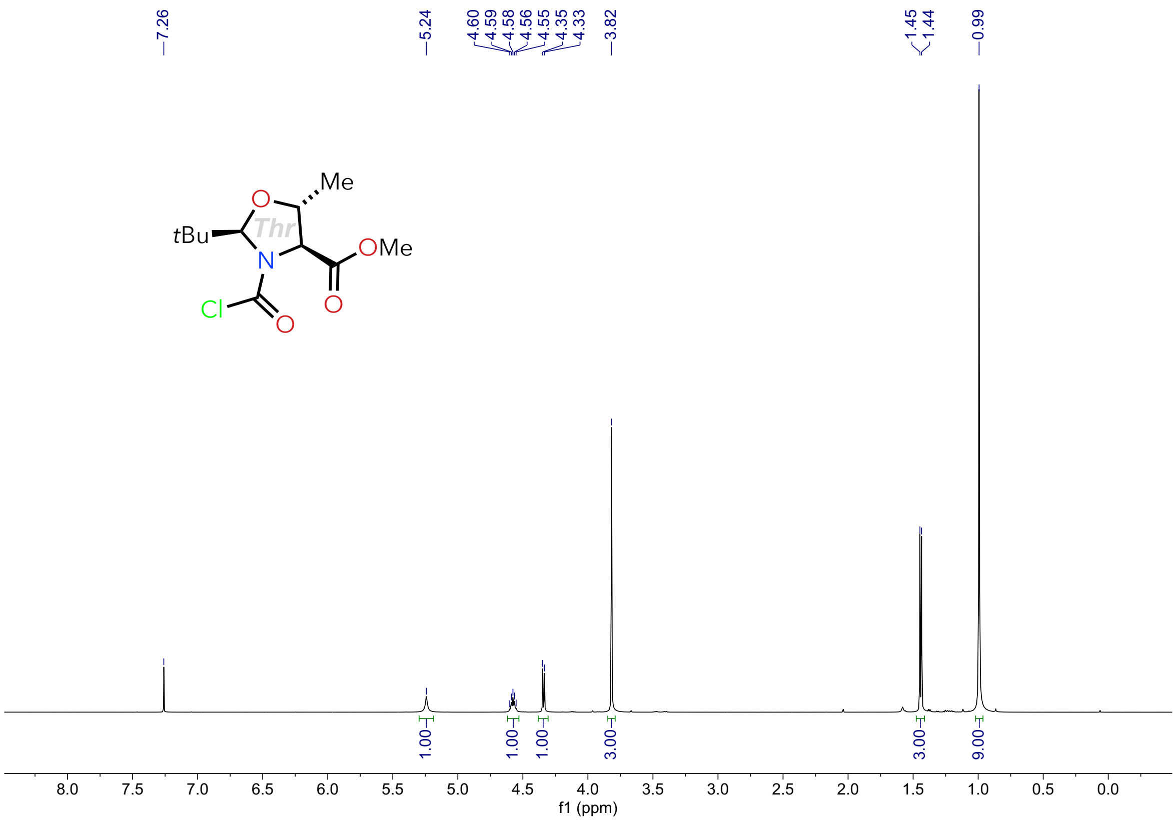
7.26
5.24
4.60
4.59
4.58
4.56
4.55
4.35
4.33
3.82
1.45
1.44
0.99
Me
O
tBu
Thr
N
OMe
O
Cl
O
1.00
1.00
1.00
3.00
3.00
9.00
8.0 7.5 7.0 6.5 6.0 5.5 5.0 4.5 4.0 3.5 3.0 2.5 2.0 1.5 1.0 0.5 0.0
f1 (ppm)

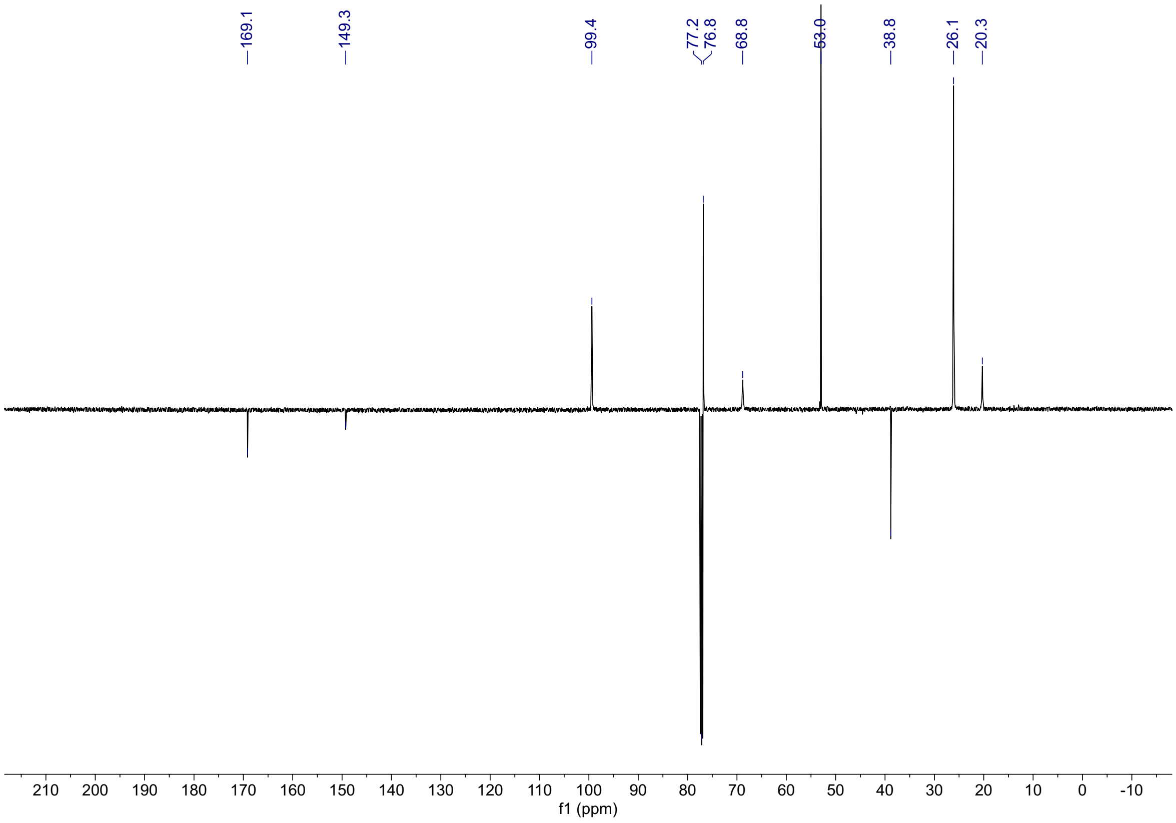
169.1
149.3
99.4
77.2
76.8
68.8
53.0
38.8
26.1
20.3
210 200 190 180 170 160 150 140 130 120 110 100 90 80 70 60 50 40 30 20 10 0 -10
f1 (ppm)

Methyl (2*R*,4*S*,5*S*)-2-(*tert*-butyl)-3-(chlorocarbonyl)-5-methyloxazolidine-4-carboxylate (18)

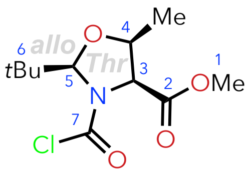

18

Following **GP2**, to a solution of crude oxazolidine **14** (1.07 g, 5.32 mmol, 1.0 equiv.) in dry CH_2Cl_2 (18 mL, 0.3 M) was added a phosgene solution (5.69 mL, 7.97 mmol, 1.5 equiv., 15 wt. % in toluene) and triethylamine (0.96 mL, 6.91 mmol, 1.3 equiv.) at –15 °C. The title compound **18** was obtained as a colourless oil (1.12 g, 4.25 mmol, 80%, single diastereomer) after purification by silica gel column chromatography (PE/EA, gradient elution).

Formula: $C_{11}H_{18}ClNO_4$, **MW:** 263.72 g/mol. **TLC:** R_f = 0.57 (PE/EA 7:3), $KMnO_4$ stain. **^{1}H NMR** (600 MHz, $CDCl_3$): δ [ppm] = 5.01 (s, 1H, H-5), 4.72 (d, *J* 7.1 Hz, 1H, H-3), 4.32 (p, *J* 6.4 Hz, 1H, H-4), 3.79 (s, 3H, H-1, OMe), 1.34 (d, *J* 6.3 Hz, 3H, Me), 1.04 (s, 9H, *t*Bu). **^{13}C NMR** (150 MHz, $CDCl_3$): δ [ppm] = 168.3 (s, C-2, ester), 148.8 (s, C-7, carbamoyl), 99.0 (d, C-5), 74.7 (d, C-4), 66.5 (d, C-3), 52.3 (q, C-1, OMe), 37.2 (s, C-6, *t*Bu), 26.4 (q, *t*Bu, 3Me), 15.7 (q, Me). **FT-IR (ATR):** $\tilde{\nu}$ [cm^{-1}] = 2958 (br w), 1750 (vs), 1482 (w), 1439 (w), 1341 (m), 1280 (m), 1207 (m), 1162 (s), 1119 (m), 1074 (m), 995 (m), 802 (m), 780 (m), 691 (w), 670 (w), 526 (w). **HR-MS:** (ESI) = *m/z* calcd. for: $C_{11}H_{19}{}^{35}ClNO_4$ $[M+H]^+$ 264.1003 u, found: 264.0990 u. **$[a]_\lambda^T$:** (c = 1.07 g/100 mL, $CHCl_3$) = $[a]_D^{20}$: +1.12°.

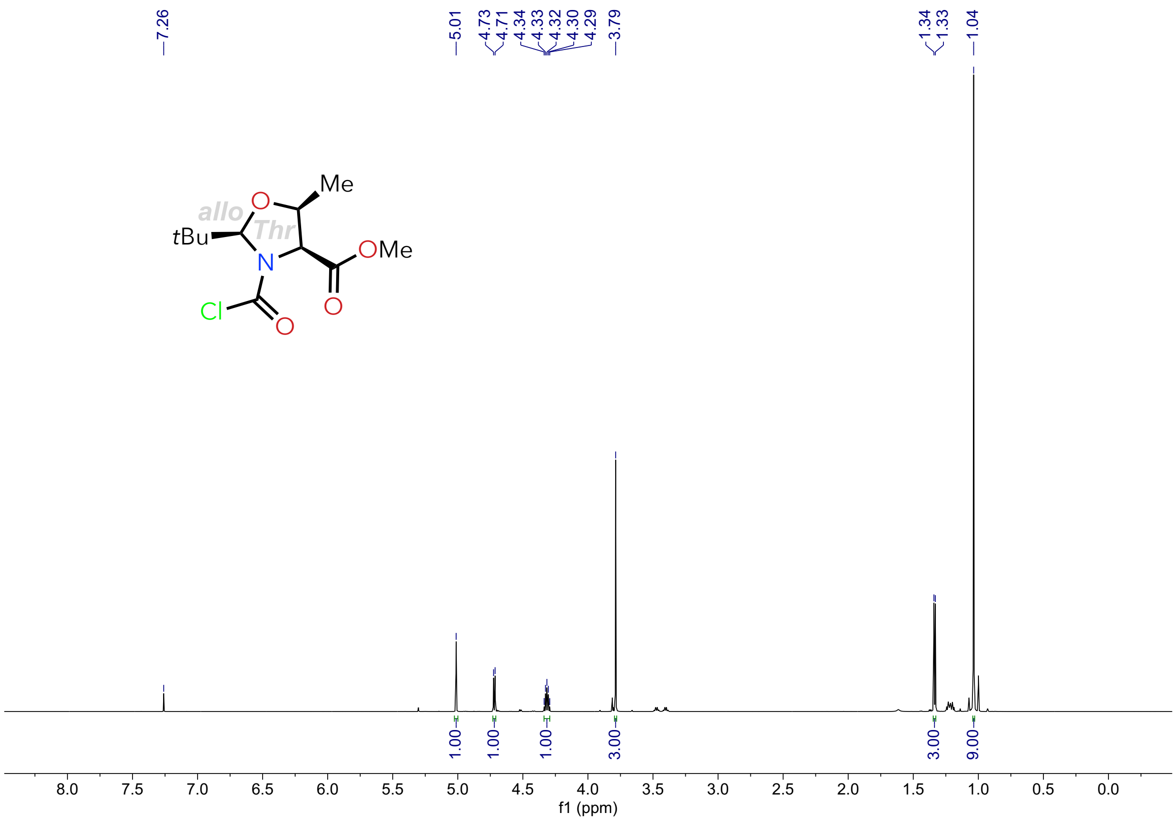
7.26
5.01
4.73
4.71
4.34
4.33
4.32
4.30
4.29
3.79
1.34
1.33
1.04
allo
Thr
Me
tBu
O
N
OMe
Cl
O
O
1.00
1.00
1.00
3.00
3.00
9.00
8.0 7.5 7.0 6.5 6.0 5.5 5.0 4.5 4.0 3.5 3.0 2.5 2.0 1.5 1.0 0.5 0.0
f1 (ppm)

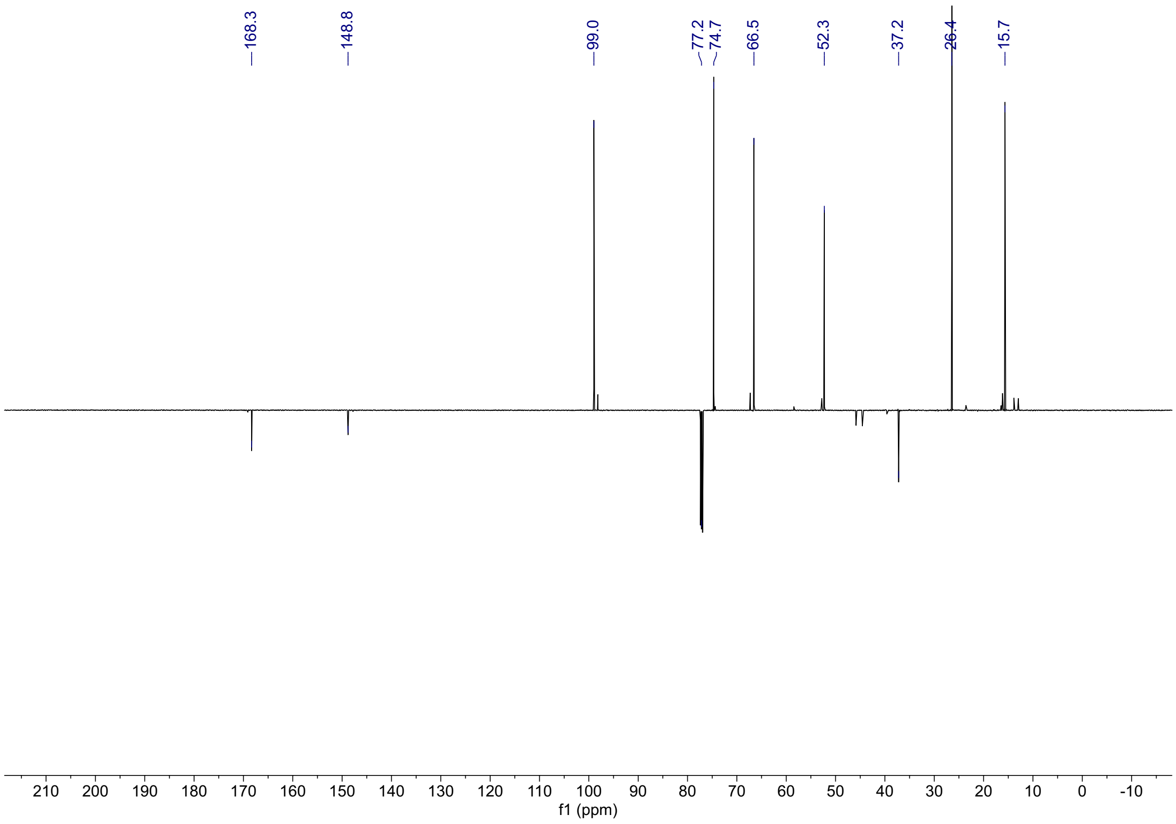
168.3
148.8
99.0
77.2
74.7
66.5
52.3
37.2
26.4
15.7
210 200 190 180 170 160 150 140 130 120 110 100 90 80 70 60 50 40 30 20 10 0 -10
f1 (ppm)

4.3 Formation of the *N*-Aryl Ureas according to General Procedure 3 (GP3)

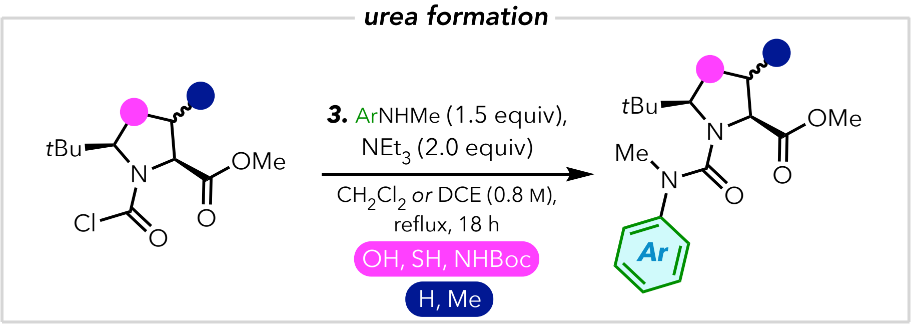

Methyl (2*R*,4*S*)-2-(*tert*-butyl)-3-(methyl(phenyl)carbamoyl)oxazolidine-4-carboxylate (4a)

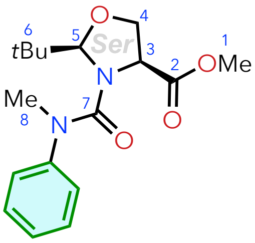

4a

Following **GP3**, to a solution of *N*-chloroformyloxazolidine **3** (500 mg, 2.0 mmol, 1.0 equiv.) in CH_2Cl_2 (2.5 mL, 0.8 M) was added triethylamine (0.55 mL, 4.0 mmol, 2.0 equiv.) and *N*-methylaniline (0.33 mL, 3.0 mmol, 1.5 equiv.). The reaction mixture was heated to reflux (40 °C) for 18 h. The product **4a** was obtained as a white solid (630 mg, 1.97 mmol, 98%) after purification by silica gel column chromatography (gradient elution, PE/EA).

Formula: $C_{17}H_{24}N_2O_4$, **MW:** 320.39 g/mol, **m.p.:** 96 – 99 °C. **TLC:** R_f = 0.50 (PE/EA 2:1), $KMnO_4$ stain. **^{1}H NMR** (500 MHz, $CDCl_3$): δ [ppm] = 7.35 – 7.32 (m, 2H, Ar), 7.22 – 7.19 (m, 1H, Ar), 7.16 – 7.15 (m, 2H, Ar), 5.36 (s, 1H, H-5), 4.29 (d, *J* 6.4 Hz, 1H, H-3), 4.01 (d, *J* 8.9 Hz, 1H, H-4a), 3.83 (dd, *J* 8.9, 6.9 Hz, 1H, H-4b), 3.22 (s, 3H, H-1, OMe), 3.19 (s, 3H, H-8, NMe), 1.00 (s, 9H, *t*Bu). **^{13}C NMR** (125 MHz, $CDCl_3$): δ [ppm] = 170.1 (s, C-2, ester), 163.0 (s, C-7, urea), 144.5 (s, Ar), 129.7 (d, Ar), 128.1 (d, Ar), 126.7 (d, Ar), 98.4 (d, C-5), 70.2 (t, C-4), 63.2 (d, C-3), 51.6 (q, C-1, OMe), 40.3 (q, C-8, NMe), 36.0 (s, C-6, *t*Bu), 26.4 (q, *t*Bu, 3Me). **FT-IR (ATR):** $\tilde{\nu}$ [cm^{-1}] = 2954 (br w), 2869 (w), 1765 (s), 1656 (vs), 1596 (m), 1495 (m), 1366 (m), 1336 (s), 1291 (m), 1196 (s), 1180 (m), 1150 (vs), 1056 (m), 1043 (m), 983 (s), 930 (m), 879 (m), 767 (m), 698 (s), 554 (w). **HR-MS:** (ESI) = *m*/*z* calcd. for: $C_{17}H_{25}N_2O_4$ $[M+H]^+$ 321.1814 u, found: 321.1817 u. **$[a]_\lambda^T$:** (c = 1.05 g/100 mL, $CHCl_3$) = $[a]_D^{20}$: –22.10°.

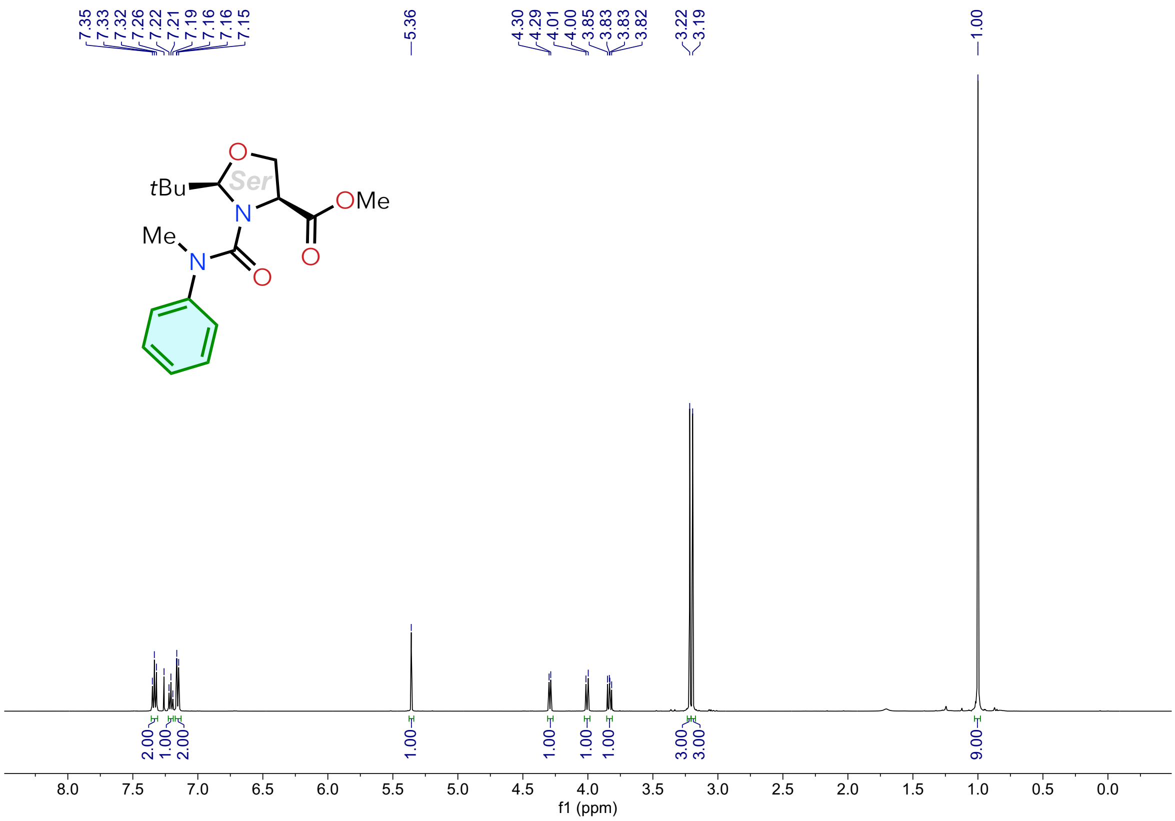
7.35 7.33 7.32 7.26 7.22 7.21 7.19 7.16 7.16 7.15
5.36
4.30 4.29 4.01 4.00 3.85 3.83 3.83 3.82
3.22 3.19
1.00
O
Ser
tBu
N
OMe
Me
N
O
O
2.00 1.00 2.00
1.00
1.00 1.00 1.00
3.00 3.00
9.00
8.0 7.5 7.0 6.5 6.0 5.5 5.0 4.5 4.0 3.5 3.0 2.5 2.0 1.5 1.0 0.5 0.0
f1 (ppm)

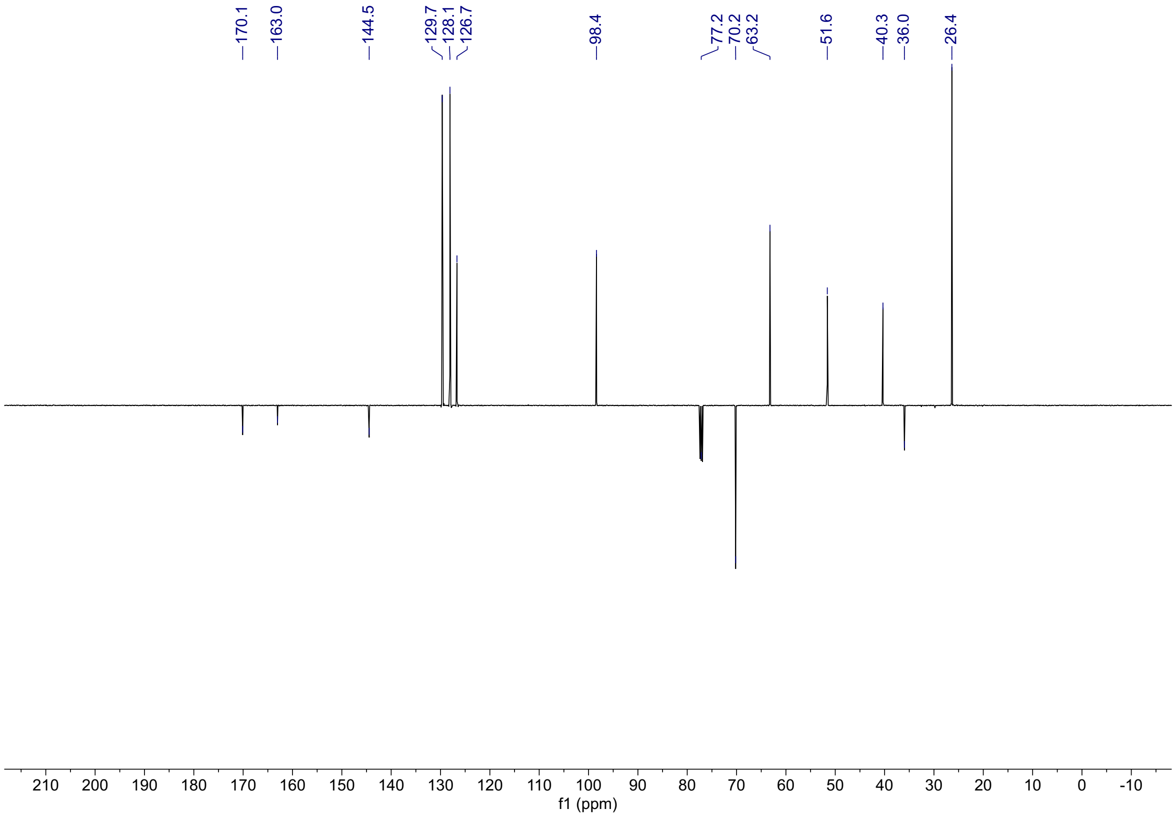
170.1
163.0
144.5
129.7 128.1 126.7
98.4
77.2 70.2 63.2
51.6
40.3 36.0
26.4
210 200 190 180 170 160 150 140 130 120 110 100 90 80 70 60 50 40 30 20 10 0 -10
f1 (ppm)

Methyl (2*R*,4*S*)-2-(*tert*-butyl)-3-((3-fluorophenyl)(methyl)carbamoyl)oxazolidine-4-carboxylate (4b)

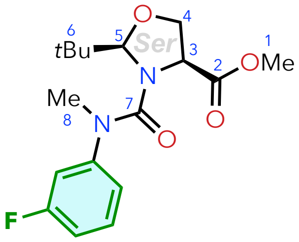

4b

Following **GP3**, *N*-chloroformyloxazolidine **3** (500-mg scale) was used as the carbamoyl chloride and 3-fluoro-*N*-methylaniline (0.34 mL, 3.0 mmol, 1.5 equiv.) as the aniline in CH_2Cl_2. The title compound **4b** was obtained as a colourless oil (668 mg, 2.70 mmol, 99%) after purification by silica gel column chromatography (PE/EA, gradient elution).

Formula: $C_{17}H_{23}FN_2O_4$, **MW:** 338.38 g/mol. **TLC:** R_f = 0.30 (PE/EA 2:1), $KMnO_4$ stain. **^{1}H NMR** (600 MHz, $CDCl_3$): δ [ppm] = 7.30 (q, *J* 7.9 Hz, 1H, Ar), 6.96 (d, *J* 7.9 Hz, 1H, Ar), 6.94 – 6.87 (m, 2H, Ar), 5.35 (s, 1H, H-5), 4.27 (d, *J* 6.9 Hz, 1H, H-3), 4.04 (d, *J* 8.9 Hz, 1H, H-4), 3.84 (dd, *J* 8.7, 7.1 Hz, 1H, H-4), 3.32 (s, 3H, H-1, OMe), 3.20 (s, 3H, H-8, NMe), 1.01 (s, 9H, *t*Bu). **^{13}C NMR** (150 MHz, $CDCl_3$): δ [ppm] = 170.0 (s, C-2), 163.9 (s, Ar), 162.7 (s, C-7), 162.3 (s, Ar), 146.0 (s, *d J* 9.5 Hz, C-9), 130.8 (d, *d J* 9.2 Hz, Ar), 123.4 (d, *d J* 3.1 Hz, Ar), 115.2 (d, *d J* 22.7 Hz, Ar), 113.6 (d, *d J* 20.9 Hz, Ar), 98.5 (d, C-5), 70.2 (t, C-4), 63.2 (d, C-3), 51.7 (q, C-1, OMe), 40.1 (q, C-8, NMe), 36.0 (s, C-6, *t*Bu), 26.4 (q, *t*Bu, 3Me). **^{19}F NMR** (565 MHz, $CDCl_3$, C_6F_6 ref.): δ [ppm] = –114.36 (dd, *J* 15.8, 8.9 Hz, F). **FT-IR (ATR):** $\tilde{\nu}$ [cm^{-1}] = 2954 (br w), 1765 (m), 1729 (w), 1658 (m), 1607 (m), 1587 (m), 1481 (m), 1335 (m), 1301 (m), 1196 (m), 1144 (vs), 1095 (m), 1057 (m), 984 (m), 896 (m), 951 (m), 860 (w), 781 (m), 698 (s), 520 (w). **HR-MS:** (ESI) = *m*/*z* calcd. for: $C_{17}H_{24}FN_2O_4$ $[M+H]^+$ 339.1715 u, found: 339.1711 u. **$[a]_\lambda^T$:** (c = 1.05 g/100 mL, $CHCl_3$) = $[a]_D^{20}$: –20.95°.

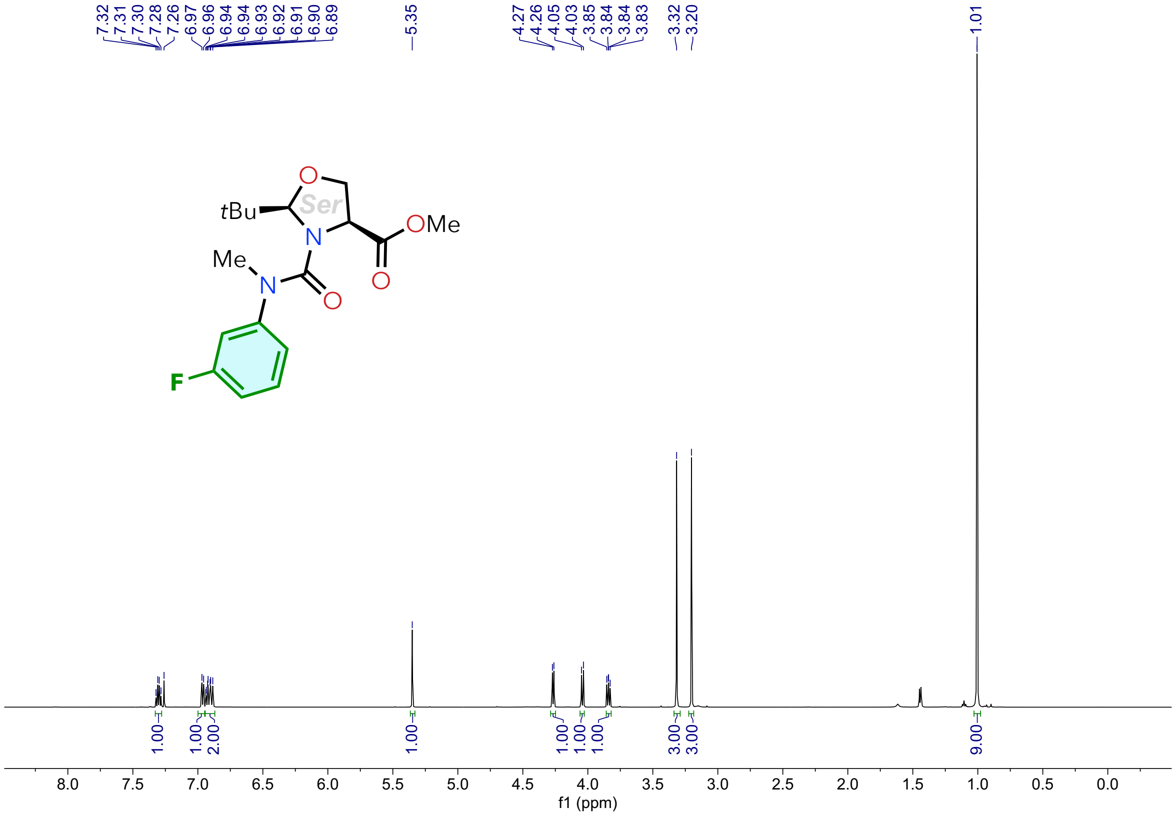
7.32
7.31
7.30
7.28
7.26
6.97
6.96
6.94
6.94
6.93
6.92
6.91
6.90
6.89
5.35
4.27
4.26
4.05
4.03
3.85
3.84
3.84
3.83
3.32
3.20
1.01
O
tBu
Ser
N
OMe
O
Me
N
O
F
1.00
1.00
2.00
1.00
1.00
1.00
1.00
3.00
3.00
9.00
f1 (ppm)

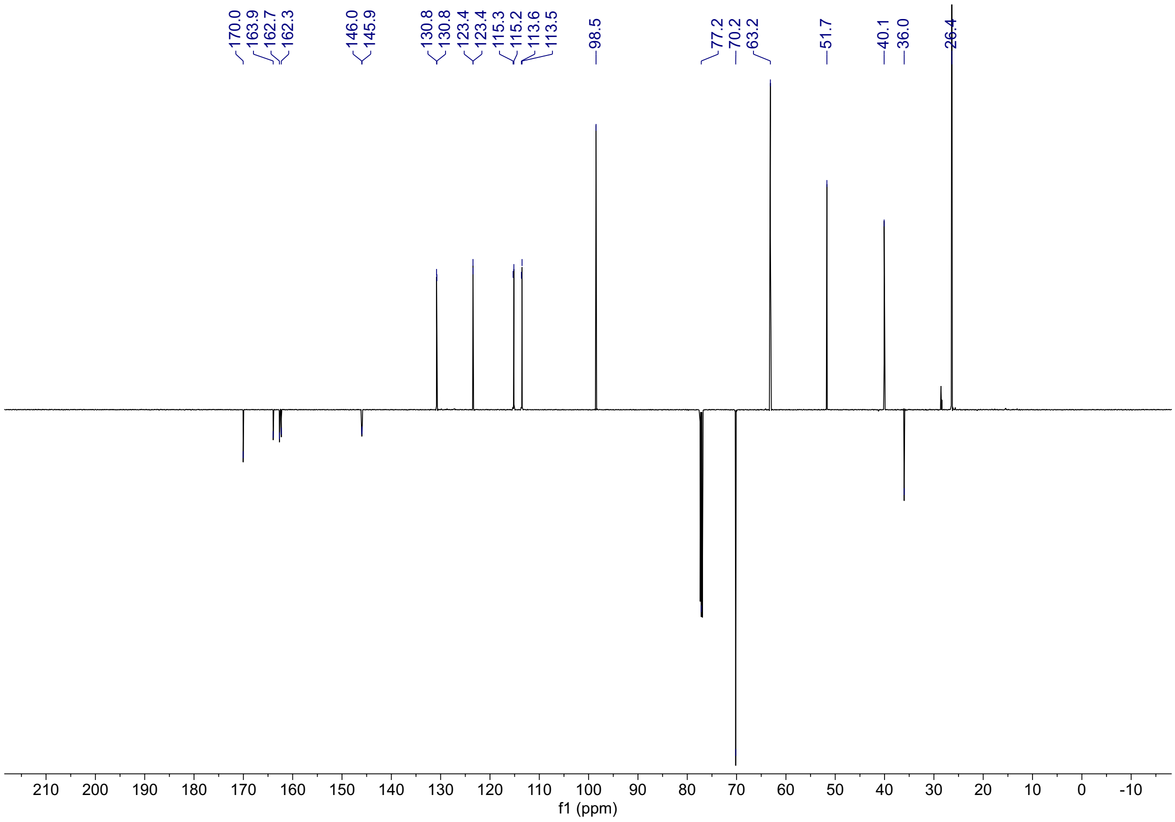
170.0
163.9
162.7
162.3
146.0
145.9
130.8
130.8
123.4
123.4
115.3
115.2
113.6
113.5
98.5
77.2
70.2
63.2
51.7
40.1
36.0
26.4
f1 (ppm)

Methyl (2*R*,4*S*)-2-(*tert*-butyl)-3-((4-chlorophenyl)(methyl)carbamoyl)oxazolidine-4-carboxylate (4c)

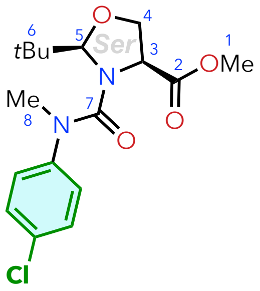

4c

Following **GP3**, *N*-chloroformyloxazolidine **3** (500-mg scale) was used as the carbamoyl chloride and 4-chloro-*N*-methylaniline (0.36 mL, 3.0 mmol, 1.5 equiv.) as the aniline in CH_2Cl_2. The title compound **4c** was obtained as a white solid (643 mg, 1.81 mmol, 91%) after purification by silica gel column chromatography (PE/EA, gradient elution).

Formula: $C_{17}H_{23}ClN_2O_4$, **MW:** 354.83 g/mol, **m.p.:** 115 – 118 °C. **TLC:** R_f = 0.35 (PE/EA 2:1), $KMnO_4$ stain. **^{1}H NMR** (500 MHz, $CDCl_3$): δ [ppm] = 7.30 (d, *J* 8.7 Hz, 2H, Ar), 7.09 (d, *J* 8.7 Hz, 2H, Ar), 5.34 (s, 1H, H-5), 4.24 (d, *J* 6.3 Hz, 1H, H-3), 4.03 (d, *J* 8.9 Hz, 1H, H-4a), 3.83 (dd, *J* 8.9, 6.9 Hz, 1H, H-4b), 3.29 (s, 3H, H-1, OMe), 3.18 (s, 3H, H-8, NMe), 0.99 (s, 9H, *t*Bu). **^{13}C NMR** (125 MHz, $CDCl_3$): δ [ppm] = 170.1 (s, C-2, ester), 162.8 (s, C-7, urea), 142.9 (s, Ar), 132.4 (s, Ar), 129.8 (d, Ar), 129.4 (d, Ar), 98.4 (d, C-5), 70.2 (t, C-4), 63.1 (d, C-3), 51.8 (q, C-1, OMe), 40.2 (q, C-8, NMe), 36.0 (s, C-6, *t*Bu), 26.4 (q, *t*Bu, 3Me). **FT-IR (ATR):** $\tilde{\nu}$ [cm^{-1}] = 2971 (br w), 2896 (br w), 1755 (s), 1667 (vs), 1493 (s), 1478 (m), 1432 (m), 1366 (m), 1335 (s), 1296 (m), 1201 (s), 1181 (s), 1154 (s), 1090 (m), 1041 (m), 1016 (m), 928 (m), 877 (m), 833 (m), 788 (m), 715 (m), 670 (m), 607 (m), 561 (m), 438 (m). **HR-MS:** (ESI) = *m*/*z* calcd. for: $C_{17}H_{24}{}^{35}ClN_2O_4$ $[M+H]^+$ 355.1425 u, found: 355.1430 u. **$[a]_\lambda^T$:** (c = 1.08 g/100 mL, $CHCl_3$) = $[a]_D^{20}$: –21.85°.

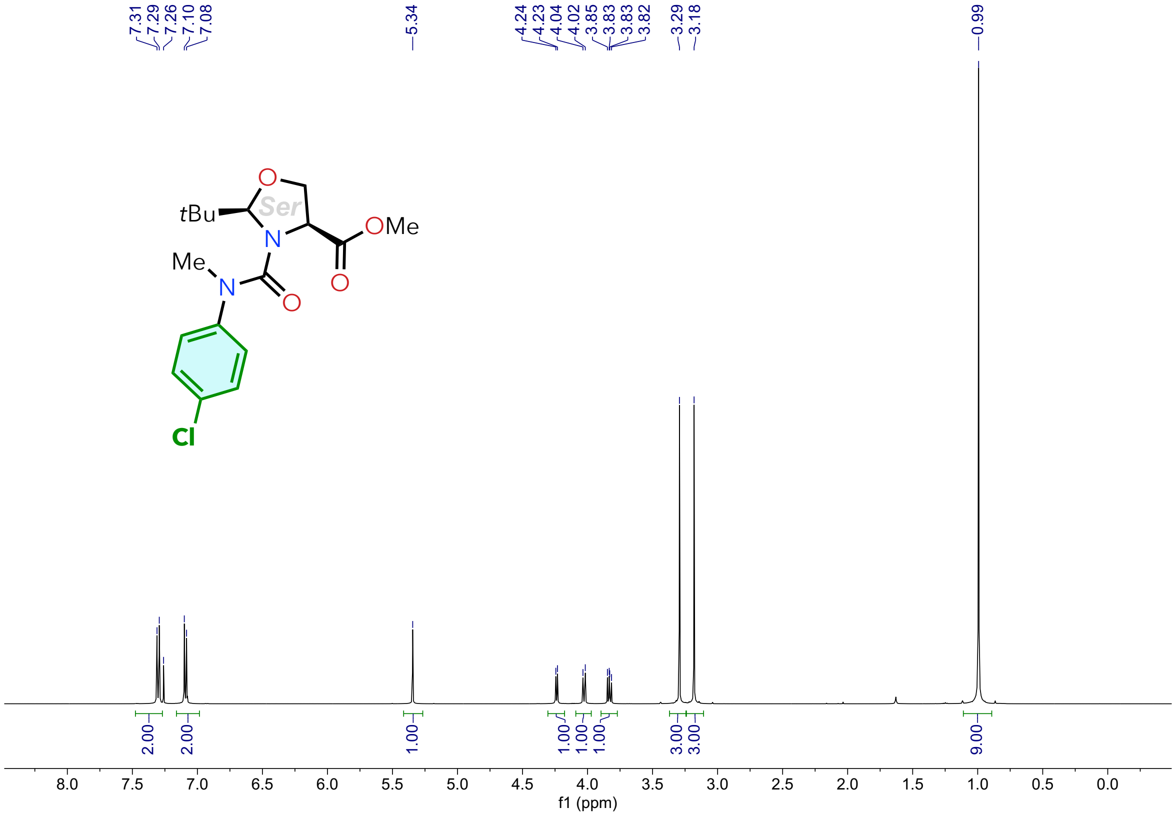
7.31
7.29
7.26
7.10
7.08
5.34
4.24
4.23
4.04
4.02
3.85
3.83
3.83
3.82
3.29
3.18
0.99
tBu
Ser
O
N
OMe
Me
Cl
2.00
2.00
1.00
1.00
1.00
1.00
3.00
3.00
9.00
8.0
7.5
7.0
6.5
6.0
5.5
5.0
4.5
4.0
3.5
3.0
2.5
2.0
1.5
1.0
0.5
0.0
f1 (ppm)

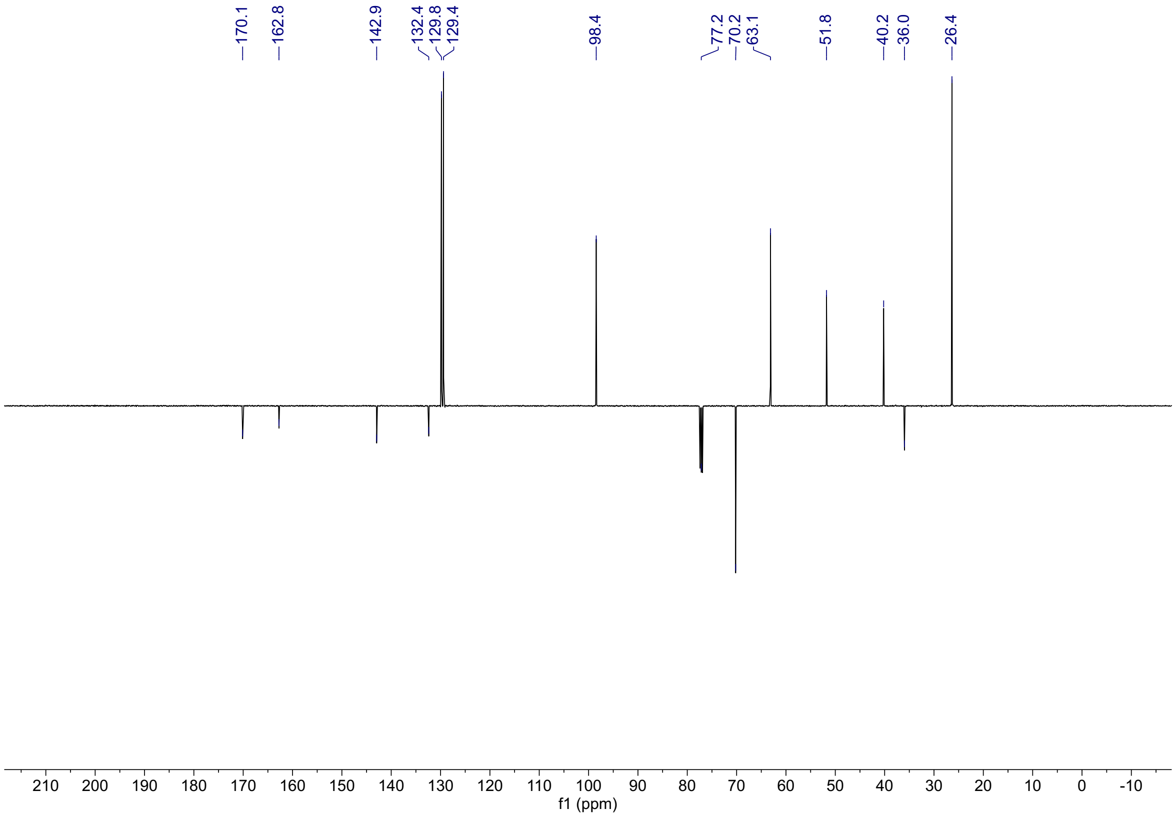
170.1
162.8
142.9
132.4
129.8
129.4
98.4
77.2
70.2
63.1
51.8
40.2
36.0
26.4
210
200
190
180
170
160
150
140
130
120
110
100
90
80
70
60
50
40
30
20
10
0
-10
f1 (ppm)

Methyl (2*R*,4*S*)-3-((3-bromophenyl)(methyl)carbamoyl)-2-(*tert*-butyl)oxazolidine-4-carboxylate (4d)

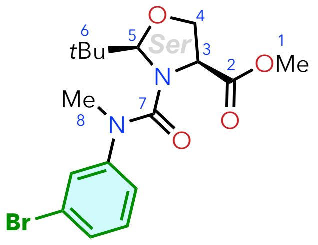

4d

Following **GP3**, *N*-chloroformyloxazolidine **3** (500-mg scale) was used as the carbamoyl chloride and 3-bromo-*N*-methylaniline (0.38 mL, 3.0 mmol, 1.5 equiv.) as the aniline in CH_2Cl_2. The title compound **4d** was obtained as a beige solid (626 mg, 1.57 mmol, 78%) after purification by silica gel column chromatography (PE/EA, gradient elution).

Formula: $C_{17}H_{23}BrN_2O_4$, **MW:** 399.29 g/mol, **m.p.:** 87 – 90 °C. **TLC:** R_f = 0.42 (PE/EA 2:1), $KMnO_4$ stain. ^{1}**H NMR** (500 MHz, $CDCl_3$): δ [ppm] = 7.36 – 7.33 (m, 2H, Ar), 7.24 – 7.21 (d, *J* 8.0 Hz, 1H, Ar), 7.11 (d, *J* 9.2 Hz, 1H, Ar), 5.34 (s, 1H, H-5), 4.25 (d, *J* 6.7 Hz, 1H, H-3), 4.04 (d, *J* 8.9 Hz, 1H, H-4a), 3.84 (dd, *J* 8.9, 6.9 Hz, 1H, H-4b), 3.34 (s, 3H, H-1, OMe), 3.19 (s, 3H, H-8, NMe), 1.00 (s, 9H, *t*Bu). ^{13}**C NMR** (125 MHz, $CDCl_3$): δ [ppm] = 170.0 (s, C-2, ester), 162.7 (s, C-7, urea), 145.8 (s, Ar), 131.2 (d, Ar), 131.1 (d, Ar), 129.7 (d, Ar), 126.6 (d, Ar), 122.7 (s, Ar), 98.4 (d, C-5), 70.2 (t, C-4), 63.2 (d, C-3), 51.9 (q, C-1, OMe), 40.1 (q, C-8, NMe), 36.0 (s, C-6, *t*Bu), 26.4 (q, *t*Bu, 3Me). **FT-IR (ATR):** $\tilde{\nu}$ [cm^{-1}] = 2957 (br w), 1758 (s), 1661 (vs), 1588 (m), 1569 (m), 1477 (m), 1427 (m), 1374 (m), 1335 (s), 1245 (m), 1201 (s), 1178 (s), 1151 (vs), 1042 (m), 985 (m), 931 (m), 878 (m), 837 (w), 780 (s), 718 (m), 692 (vs), 662 (w), 609 (w), 525 (w). **HR-MS:** (ESI) = *m*/*z* calcd. for: $C_{17}H_{24}{}^{79}BrN_2O_4$ $[M+H]^+$ 399.0919 u, found: 399.0902 u. $\mathbf{[a]_\lambda^T}$**:** (c = 1.00 g/100 mL, $CHCl_3$) = $[a]_D^{20}$: –18.80°.

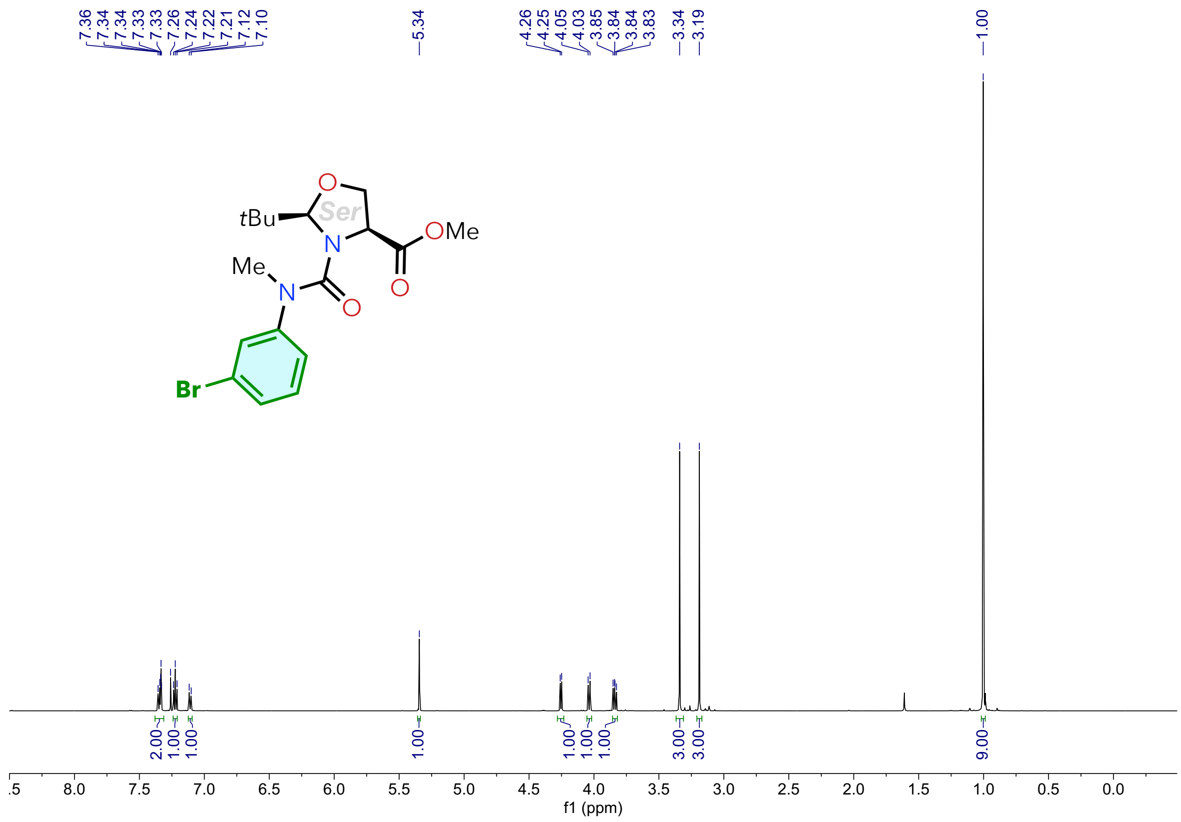
7.36
7.34
7.34
7.33
7.33
7.26
7.24
7.22
7.21
7.12
7.10
5.34
4.26
4.25
4.05
4.03
3.85
3.84
3.84
3.83
3.34
3.19
1.00
O
tBu
Ser
N
OMe
Me
N
O
O
Br
2.00
1.00
1.00
1.00
1.00
1.00
1.00
3.00
3.00
9.00
8.0
7.5
7.0
6.5
6.0
5.5
5.0
4.5
4.0
3.5
3.0
2.5
2.0
1.5
1.0
0.5
0.0
f1 (ppm)

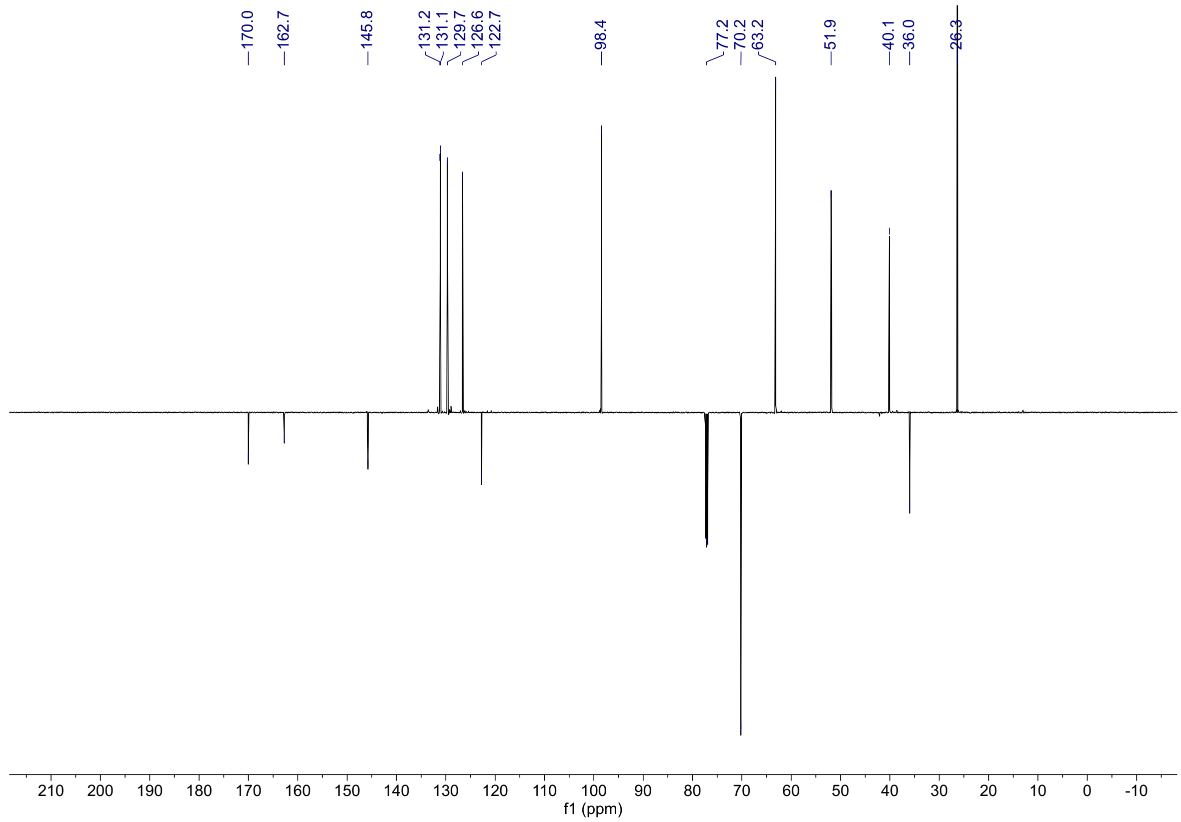
170.0
162.7
145.8
131.2
131.1
129.7
126.6
122.7
98.4
77.2
70.2
63.2
51.9
40.1
36.0
26.3
210
200
190
180
170
160
150
140
130
120
110
100
90
80
70
60
50
40
30
20
10
0
-10
f1 (ppm)

Methyl (2*R*,4*S*)-3-((2-bromophenyl)(methyl)carbamoyl)-2-(*tert*-butyl)oxazolidine-4-carboxylate (4e)

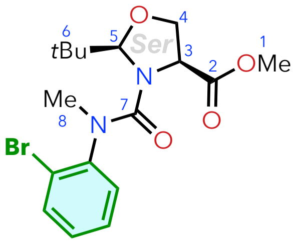

4e

Following **GP3**, *N*-chloroformyloxazolidine **3** (500-mg scale) was used as the carbamoyl chloride and 2-bromo-*N*-methylaniline (0.38 mL, 3.0 mmol, 1.5 equiv.) as the aniline in CH_2Cl_2. The title compound **4e** was obtained as a white solid (324 mg, 0.81 mmol, 41%) after purification by silica gel column chromatography (PE/EA, gradient elution).

Formula: $C_{17}H_{23}BrN_2O_4$, **MW:** 399.29 g/mol, **m.p.:** 74 – 77 °C. **TLC:** R_f = 0.45 (PE/EA 2:1), $KMnO_4$ stain. **^{1}H NMR** (500 MHz, $CDCl_3$): δ [ppm] = 7.57 (dd, *J* 8.1, 1.4 Hz, 1H, Ar), 7.36 (td, *J* 7.7, 1.4 Hz, 1H, Ar), 7.24 (dd, *J* 8.0, 1.6 Hz, 1H, Ar), 7.14 (ddd, *J* 8.0, 7.4, 1.6 Hz, 1H, Ar), 5.35 (s, 1H, H-5), 4.39 (d, *J* 6.7 Hz, 1H, H-3), 4.09 (d, *J* 8.9 Hz, 1H, H-4a), 3.87 (dd, *J* 8.9, 6.9 Hz, 1H, H-4b), 3.26 (s, 3H, H-1, OMe), 3.11 (s, 3H, H-8, NMe), 0.99 (s, 9H, *t*Bu). **^{13}C NMR** (125 MHz, $CDCl_3$): δ [ppm] = 169.6 (s, C-2, ester), 162.7 (s, C-7, urea), 142.9 (s, Ar), 133.6 (d, Ar), 131.6 (d, Ar), 129.2 (d, Ar), 129.0 (d, C-12), 123.4 (s, Ar), 98.6 (d, C-5), 70.3 (t, C-4), 62.0 (d, C-3), 51.7 (q, C-1, OMe), 38.6 (q, C-8, NMe), 36.0 (s, C-6, *t*Bu), 26.3 (q, *t*Bu, 3Me). **FT-IR (ATR):** $\tilde{\nu}$ [cm^{-1}] = 2954 (br w), 1757 (m), 1658 (vs), 1478 (m), 1423 (m), 1378 (m), 1341 (s), 1321 (m), 1201 (s), 1151 (vs), 1029 (m), 971 (m), 876 (w), 762 (m), 726 (m), 665 (m), 607 (m). **HR-MS:** (ESI) = *m*/*z* calcd. for: $C_{17}H_{24}{}^{79}BrN_2O_4$ [M+H]$^+$ 399.0919 u, found: 399.0914 u. **$[a]_\lambda^T$:** (c = 1.01 g/100 mL, $CHCl_3$) = $[a]_D^{20}$: –20.59°.

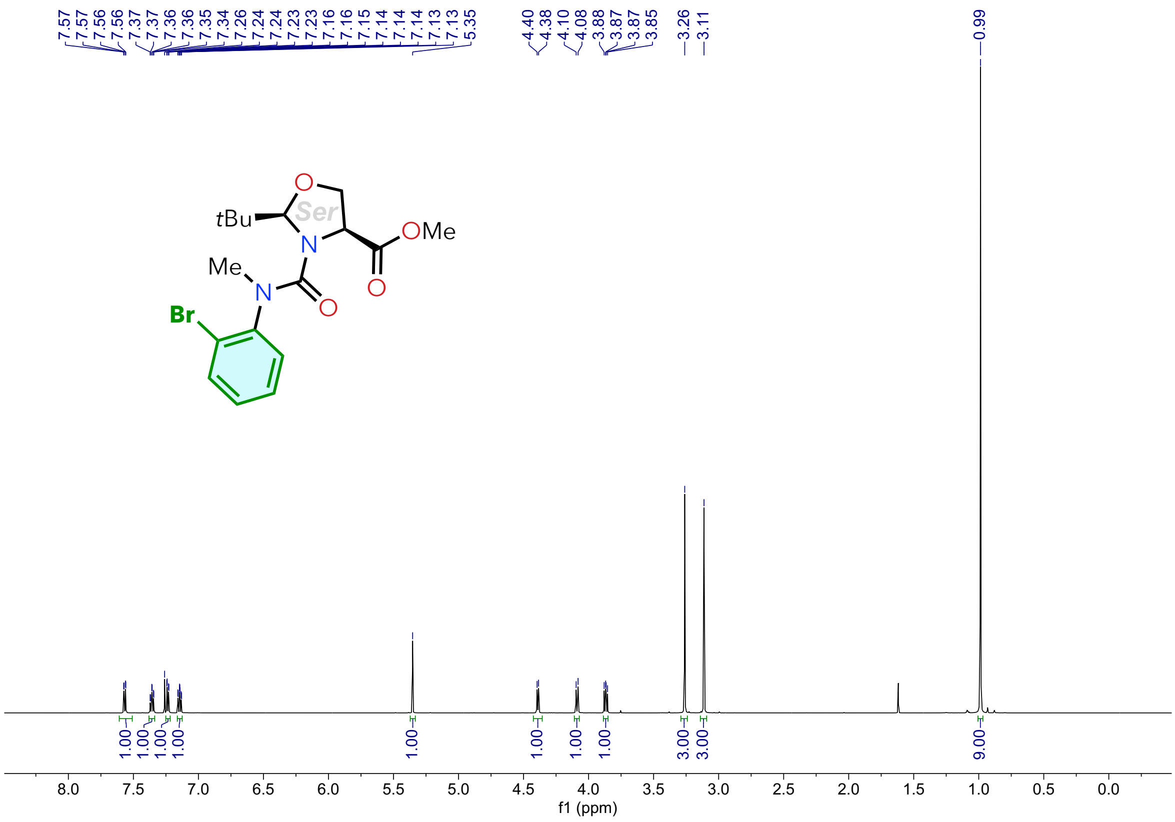

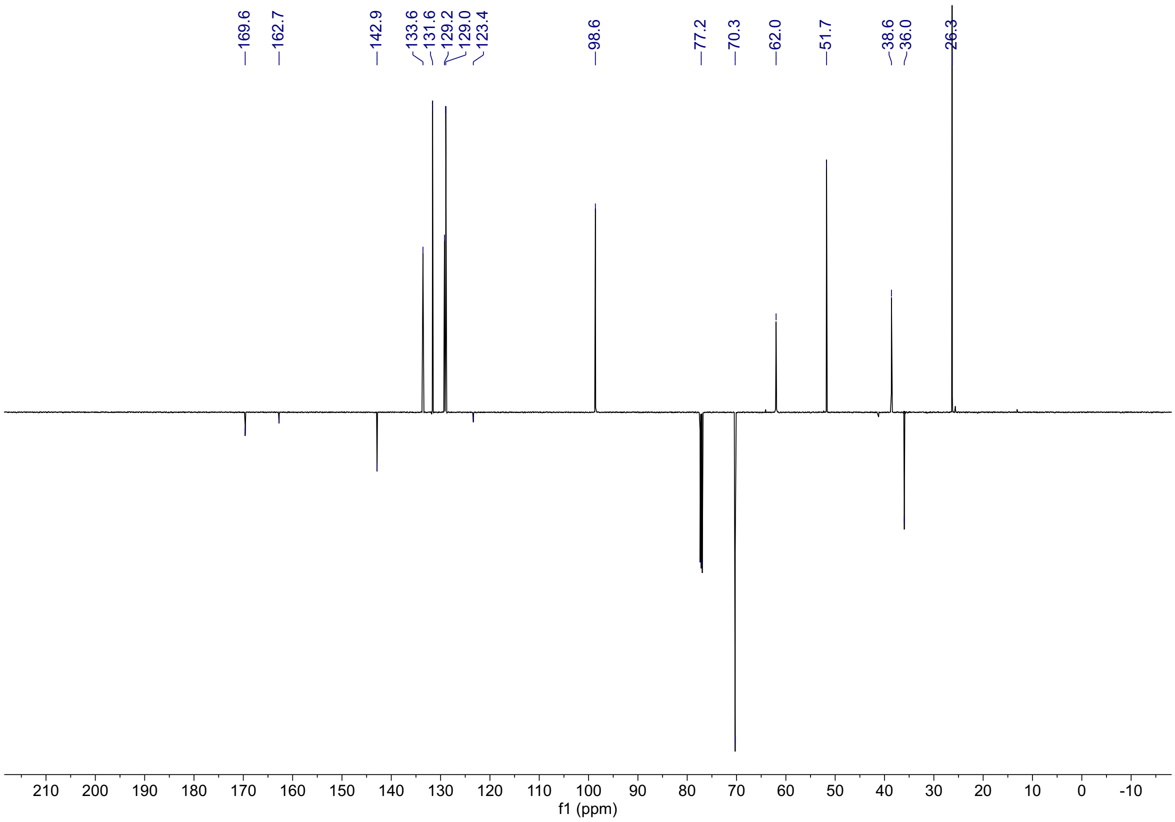

Methyl (2*R*,4*S*)-2-(*tert*-butyl)-3-(methyl(4-(trifluoromethyl)phenyl)carbamoyl)oxazolidine-4-carboxylate (4f)

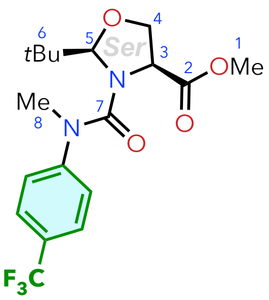

4f

Following **GP3**, *N*-chloroformyloxazolidine **3** (500-mg scale) was used as the carbamoyl chloride and *N*-methyl-4-(trifluoromethyl)aniline (0.39 mL, 3.0 mmol, 1.5 equiv.) as the aniline in CH_2Cl_2. The title compound **4f** was obtained as a white solid (416 mg, 1.07 mmol, 54%) after purification by silica gel column chromatography (PE/EA, gradient elution).

Formula: $C_{18}H_{23}F_3N_2O_4$, **MW:** 388.39 g/mol, **m.p.:** 93 – 97 °C. **TLC:** R_f = 0.33 (PE/EA 2:1), $KMnO_4$ stain. **^{1}H NMR** (500 MHz, $CDCl_3$): δ [ppm] = 7.60 (d, *J* 8.4 Hz, 2H, Ar), 7.29 (d, *J* 8.4 Hz, 2H, Ar), 5.36 (s, 1H, H-5), 4.25 (d, *J* 6.5 Hz, 1H, H-3), 4.04 (d, *J* 9.0 Hz, 1H, H-4a), 3.84 (dd, *J* 8.9, 6.9 Hz, 1H, H-4b), 3.23 (d, *J* 3.5 Hz, 6H, H-1, OMe, H-8, NMe), 1.01 (s, 9H, *t*Bu). **^{13}C NMR** (125 MHz, $CDCl_3$): δ [ppm] = 169.9 (s, C-2, ester), 162.6 (s, C-7, urea), 147.6 (s, Ar), 128.5 (s, *q J* 32.8 Hz, Ar), 127.9 (d, Ar), 126.8 (d, *q J* 3.7 Hz, Ar), 124.0 (s, *q J* 272.0 Hz, Ar), 98.6 (d, C-5), 70.2 (t, C-4), 63.1 (d, C-3), 51.7 (q, C-1, OMe), 40.0 (q, C-8, NMe), 36.0 (s, C-6, *t*Bu), 26.4 (q, *t*Bu, 3Me). **^{19}F NMR** (471 MHz, $CDCl_3$, C_6F_6 ref.): δ [ppm] = –65.60 (s, 3F). **FT-IR (ATR):** $\tilde{\nu}$ [cm^{-1}] = 2960 (br w), 1759 (m), 1666 (s), 1614 (m), 1518 (w), 1402 (w), 1428 (m), 1324 (vs), 1192 (m), 1153 (vs), 1107 (vs), 1065 (s), 1013 (m), 969 (m), 930 (w), 849 (m), 787 (w), 667 (w), 615 (m), 543 (w). **HR-MS:** (ESI) = *m/z* calcd. for: $C_{18}H_{24}F_3N_2O_4$ $[M+H]^+$ 389.1688, found: 389.1670 u. **$[a]_\lambda^T$:** (c = 0.95 g/100 mL, $CHCl_3$) = $[a]_D^{20}$: –23.58°.

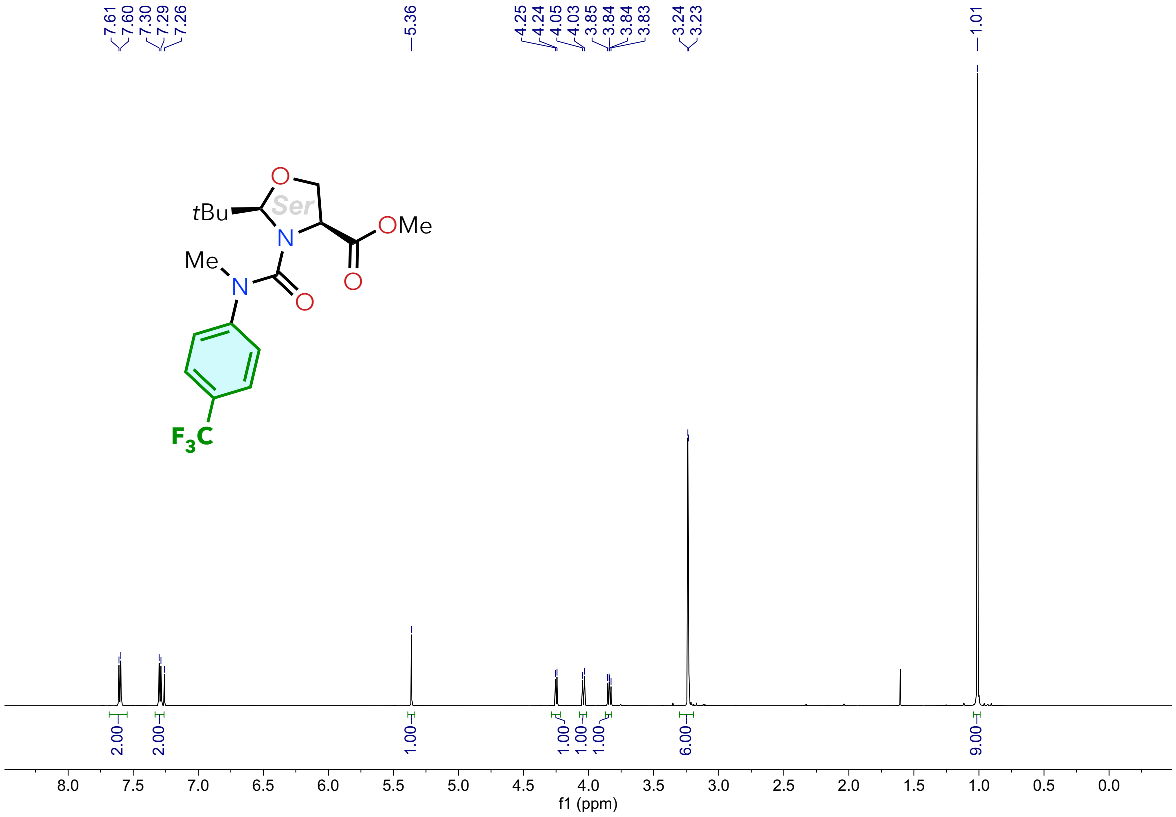
7.61
7.60
7.30
7.29
7.26
5.36
4.25
4.24
4.05
4.03
3.85
3.84
3.84
3.83
3.24
3.23
1.01
tBu
O
Ser
N
OMe
O
Me
N
O
F3C
2.00
2.00
1.00
1.00
1.00
1.00
6.00
9.00
8.0 7.5 7.0 6.5 6.0 5.5 5.0 4.5 4.0 3.5 3.0 2.5 2.0 1.5 1.0 0.5 0.0
f1 (ppm)

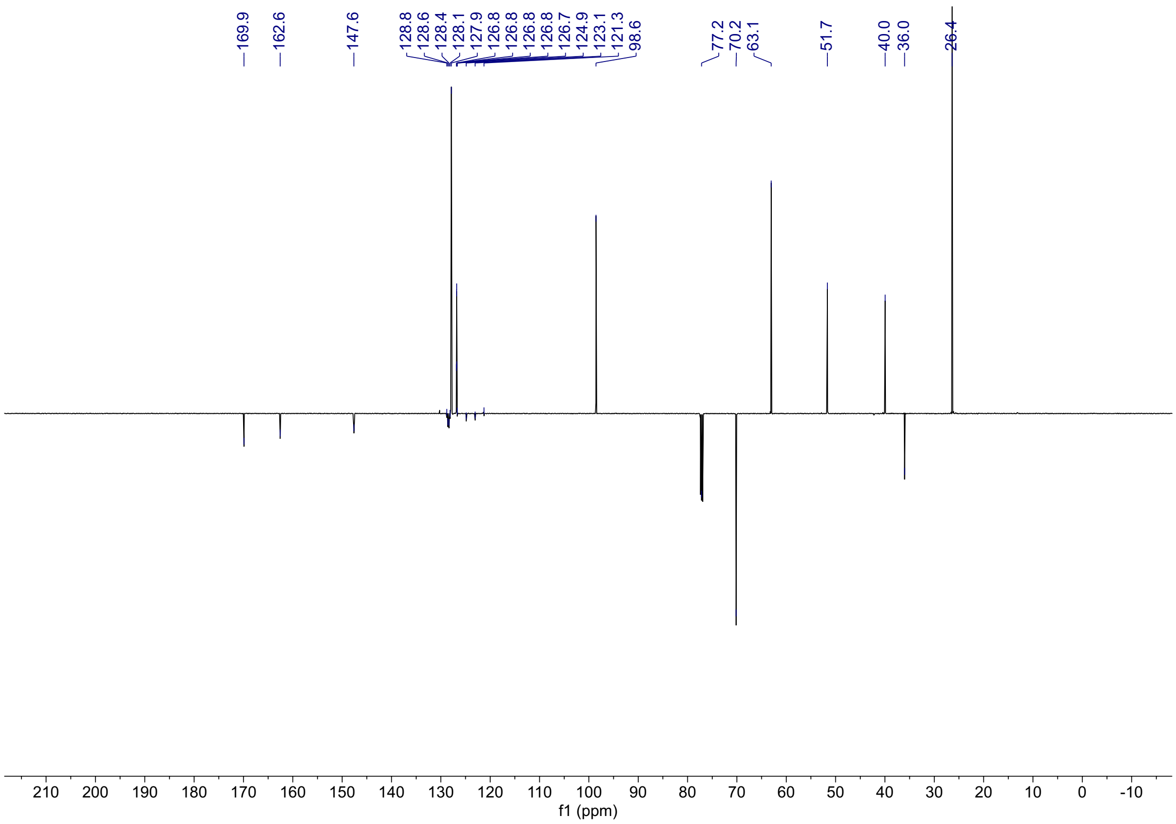
169.9
162.6
147.6
128.8
128.6
128.4
128.1
127.9
126.8
126.8
126.8
126.8
126.7
124.9
123.1
121.3
98.6
77.2
70.2
63.1
51.7
40.0
36.0
26.4
210 200 190 180 170 160 150 140 130 120 110 100 90 80 70 60 50 40 30 20 10 0 -10
f1 (ppm)

Methyl (2*R*,4*S*)-2-(*tert*-butyl)-3-((4-cyanophenyl)(methyl)carbamoyl)oxazolidine-4-carboxylate (4g)

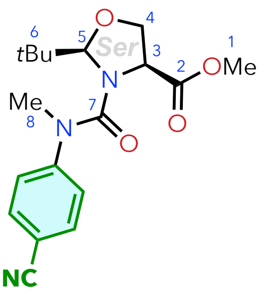

4g

Following **GP3**, *N*-chloroformyloxazolidine **3** (500-mg scale) was used as the carbamoyl chloride and 4-(methylamino)benzonitrile (396 mg, 3.0 mmol, 1.5 equiv.) as the aniline in CH_2Cl_2. The title compound **4g** was obtained as a beige solid (338 mg, 0.98 mmol, 49%) after purification by silica gel column chromatography (PE/EA, gradient elution).

Formula: $C_{18}H_{23}N_3O_4$, **MW:** 345.40 g/mol, **m.p.:** 137 – 140 °C. **TLC:** R_f = 0.33 (PE/EA 2:1), $KMnO_4$ stain. **^{1}H NMR** (500 MHz, $CDCl_3$): δ [ppm] = 7.62 (d, *J* 8.6 Hz, 2H, Ar), 7.28 (d, *J* 8.6 Hz, 2H, Ar), 5.34 (s, 1H, H-5), 4.24 (d, *J* 6.3 Hz, 1H, H-3), 4.08 (d, *J* 9.0 Hz, 1H, H-4a), 3.85 (dd, *J* 9.0, 6.8 Hz, 1H, H-4b), 3.34 (s, 3H, H-1, OMe), 3.25 (s, 3H, H-8, NMe), 1.01 (s, 9H, *t*Bu). **^{13}C NMR** (125 MHz, $CDCl_3$): δ [ppm] = 169.8 (s, C-2, ester), 162.3 (s, C-7, urea), 148.5 (s, Ar), 133.9 (d, Ar), 133.5 (d, Ar), 127.3 (d, Ar), 126.7 (d, Ar), 118.3 (s, Ar), 109.4 (s, Ar), 98.6 (d, C-5), 70.1 (t, C-4), 62.9 (d, C-3), 52.0 (q, C-1, OMe), 39.5 (q, C-8, NMe), 36.0 (s, C-6, *t*Bu), 26.3 (q, *t*Bu, 3Me). **FT-IR (ATR):** $\tilde{\nu}$ [cm^{-1}] = 2960 (br w), 2232 (w), 1755 (s), 1670 (vs), 1602 (m), 1505 (m), 1458 (w), 1433 (w), 1367 (m), 1333 (s), 1256 (w), 1199 (s), 1178 (s), 1153 (vs), 1105 (m), 1041 (w), 985 (m), 928 (w), 871 (w), 850 (m), 788 (w), 732 (w), 670 (w), 587 (s). **HR-MS:** (ESI) = *m*/*z* calcd. for: $C_{18}H_{24}N_3O_4$ $[M+H]^+$ 346.1767 u, found: 346.1761 u. **$[a]_\lambda^T$:** (c = 0.94 g/100 mL, $CHCl_3$) = $[a]_D^{20}$: –31.49°.

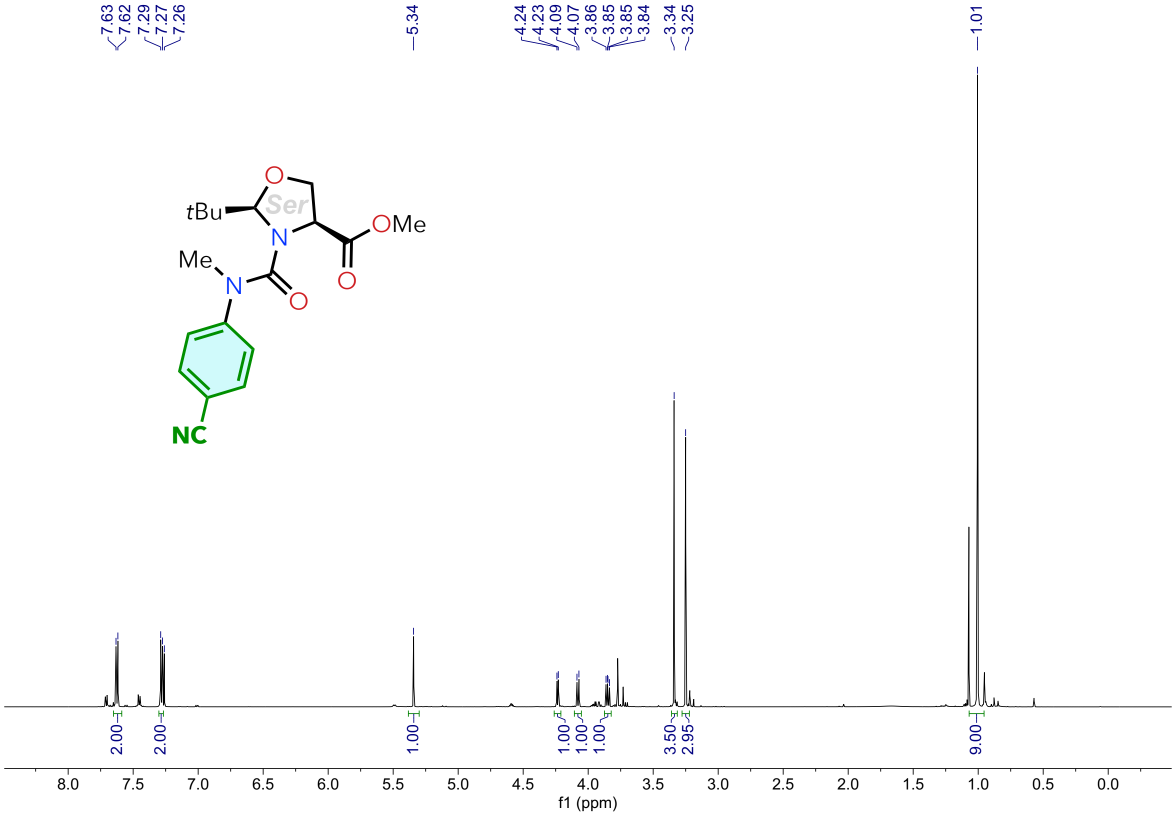
7.63
7.62
7.29
7.27
7.26
5.34
4.24
4.23
4.09
4.07
3.86
3.85
3.85
3.84
3.34
3.25
1.01
tBu
Ser
OMe
Me
NC
2.00
2.00
1.00
1.00
1.00
1.00
3.50
2.95
9.00
f1 (ppm)

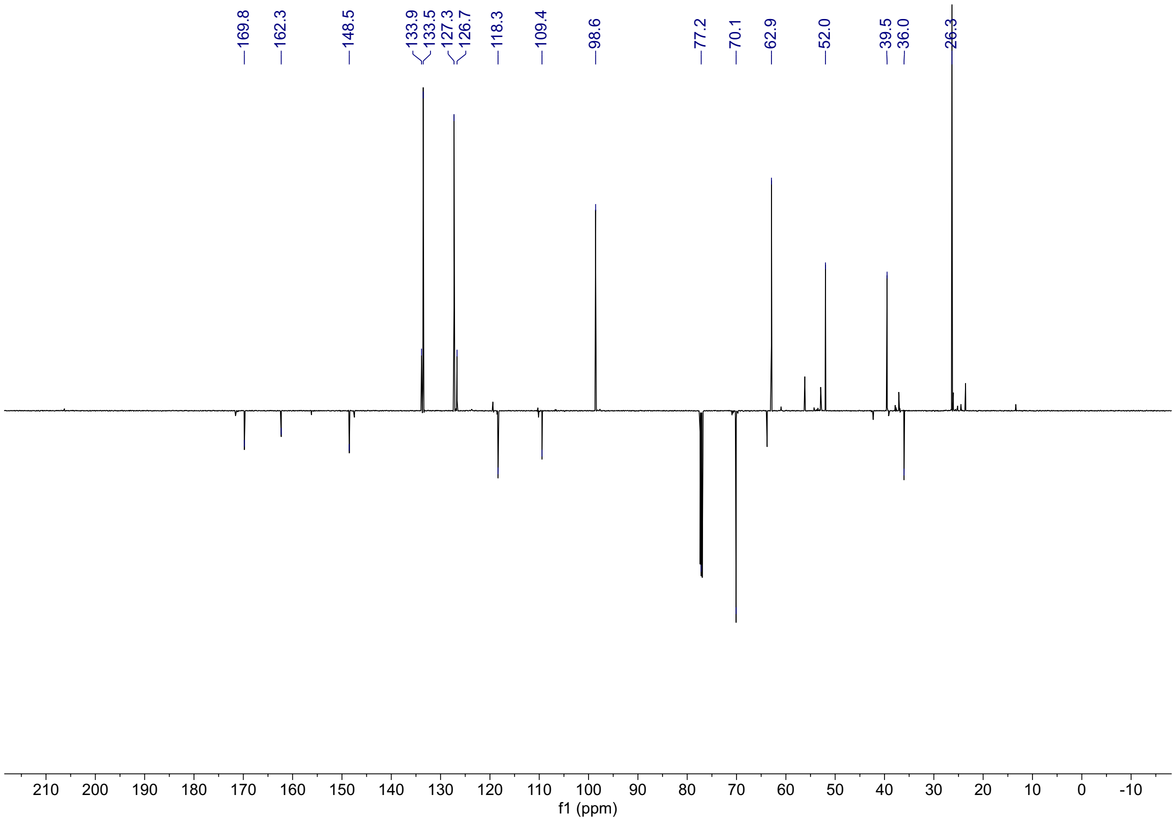
169.8
162.3
148.5
133.9
133.5
127.3
126.7
118.3
109.4
98.6
77.2
70.1
62.9
52.0
39.5
36.0
26.3
f1 (ppm)

Methyl (2*R*,4*S*)-2-(*tert*-butyl)-3-((3-isocyanophenyl)(methyl)carbamoyl)oxazolidine-4-carboxylate (4h)

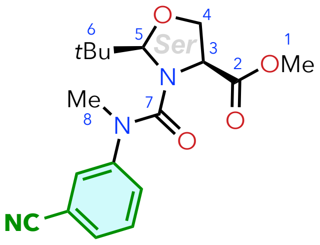

4h

Following **GP3**, *N*-chloroformyloxazolidine **3** (500-mg scale) was used as the carbamoyl chloride and 3-(methylamino)benzonitrile (396 mg, 3.0 mmol, 1.5 equiv.) as the aniline in CH_2Cl_2. The title compound **4h** was obtained as an off-white solid (649 mg, 1.88 mmol, 94%) after purification by silica gel column chromatography (PE/EA, gradient elution).

Formula: $C_{18}H_{23}N_3O_4$, **MW:** 345.40 g/mol, **m.p.:** 127 – 130 °C. **TLC:** R_f = 0.28 (PE/EA 2:1), $KMnO_4$ stain. **^{1}H NMR** (500 MHz, $CDCl_3$): δ [ppm] = 7.50 – 7.41 (m, 4H, Ar), 5.34 (s, 1H, H-5), 4.24 (d, *J* 6.3 Hz, 1H, H-3), 4.06 (d, *J* 9.0 Hz, 1H, H-4a), 3.85 (dd, *J* 9.0, 6.9 Hz, 1H, H-4b), 3.33 (s, 3H, H-1, OMe), 3.21 (s, 3H, H-8, NMe), 0.99 (s, 9H, *t*Bu). **^{13}C NMR** (125 MHz, $CDCl_3$): δ [ppm] = 169.8 (s, C-2, ester), 162.5 (s, C-7, urea), 145.4 (s, Ar), 132.3 (d, Ar), 131.0 (d, Ar), 130.8 (d, Ar), 129.9 (d, Ar), 117.9 (s, Ar), 113.6 (s, Ar), 98.5 (d, C-5), 70.1 (t, C-4), 63.0 (d, C-3), 51.9 (q, C-1, OMe), 39.9 (q, C-8, NMe), 35.9 (s, C-6, *t*Bu), 26.3 (q, *t*Bu, 3Me). **FT-IR (ATR):** $\tilde{\nu}$ [cm^{-1}] = 2968 (br w), 2227 (w), 1765 (m), 1657 (vs), 1595 (w), 1484 (w), 1428 (m), 1369 (w), 1325 (s), 1274 (w), 1288 (w), 1193 (m), 1169 (m), 1146 (vs), 1100 (w), 1040 (w), 985 (m), 946 (w), 857 (w), 787 (w), 693 (s), 677 (w), 658 (w), 587 (w), 533 (w). **HR-MS:** (ESI) = *m*/*z* calcd. for: $C_{18}H_{24}N_3O_4$ $[M+H]^+$ 346.1767 u, found: 346.1761 u. **$[a]_\lambda^T$:** (c = 1.09 g/100 mL, $CHCl_3$) = $[a]_D^{20}$: –23.49°.

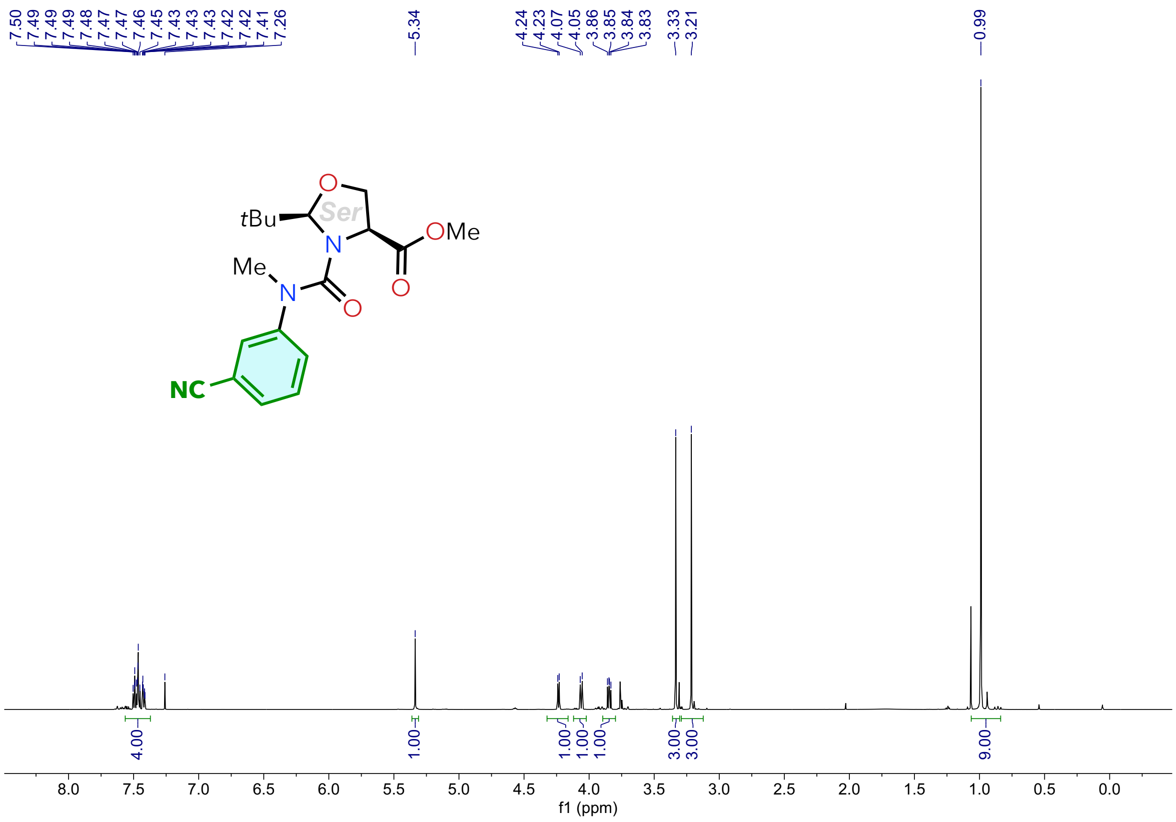

7.50
7.49
7.49
7.49
7.48
7.47
7.47
7.46
7.45
7.43
7.43
7.43
7.42
7.42
7.41
7.26
5.34
4.24
4.23
4.07
4.05
3.86
3.85
3.84
3.83
3.33
3.21
0.99
tBu
Ser
O
N
OMe
O
Me
N
O
NC
4.00
1.00
1.00
1.00
1.00
3.00
3.00
9.00
f1 (ppm)

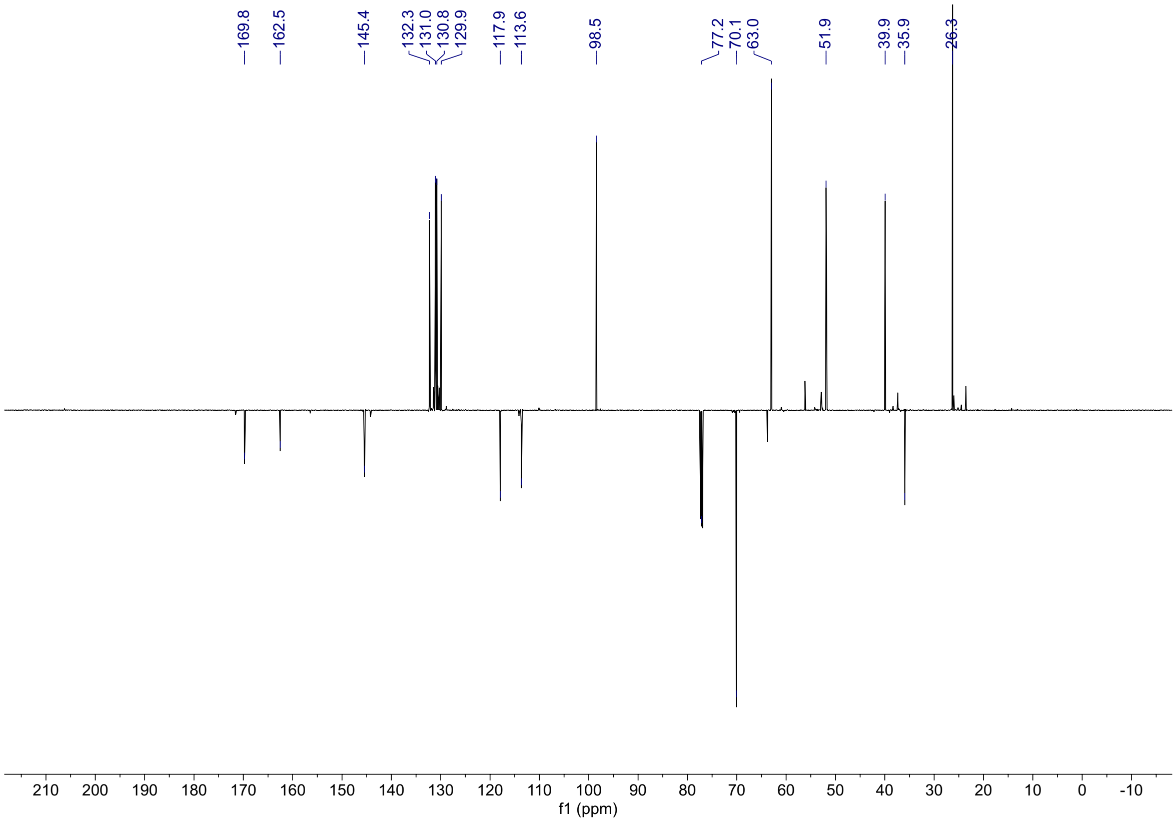

169.8
162.5
145.4
132.3
131.0
130.8
129.9
117.9
113.6
98.5
77.2
70.1
63.0
51.9
39.9
35.9
26.3
f1 (ppm)

Methyl (2*R*,4*S*)-2-(*tert*-butyl)-3-((3-ethynylphenyl)(methyl)carbamoyl)oxazolidine-4-carboxylate (4i)

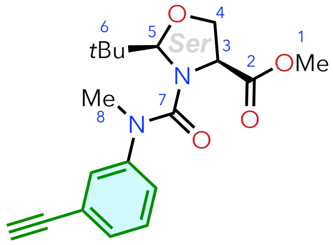

4i

Following **GP3**, *N*-chloroformyloxazolidine **3** (500-mg scale) was used as the carbamoyl chloride and 3-ethynyl-*N*-methylaniline (0.40 mL, 3.0 mmol, 1.5 equiv.) as the aniline in CH_2Cl_2. The title compound **4i** was obtained as a yellow oil (646 mg, 1.88 mmol, 94%) after purification by silica gel column chromatography (PE/EA, gradient elution).

Formula: $C_{19}H_{24}N_2O_4$, **MW:** 344.41 g/mol. **TLC:** R_f = 0.35 (PE/EA 2:1), $KMnO_4$ stain. **^{1}H NMR** (600 MHz, $CDCl_3$): δ [ppm] = 7.35 – 7.29 (m, 3H, Ar), 7.16 (dt, *J* 7.7, 1.8 Hz, 1H, Ar), 5.35 (s, 1H, H-5), 4.26 (d, *J* 6.3 Hz, 1H, H-5), 4.03 (d, *J* 8.9 Hz, 1H, H-4a), 3.84 (dd, *J* 8.9, 6.9 Hz, 1H, H-4b), 3.31 (s, 3H, H-1, OMe), 3.19 (s, 3H, H-8, NMe), 3.11 (s, 1H, Ar, ≡CH), 1.00 (s, 9H, *t*Bu). **^{13}C NMR** (150 MHz, $CDCl_3$): δ [ppm] = 170.0 (s, C-2, ester), 162.8 (s, C-7, urea), 142.5 (s, Ar), 131.6 (d, Ar), 130.3 (d, Ar), 129.9 (d, Ar), 128.7 (d, Ar), 123.6 (s, Ar), 98.4 (d, C-5), 82.6 (s, Ar, C≡), 78.3 (s, Ar, ≡CH), 70.2 (t, C-4), 63.2 (d, C-3), 51.8 (q, C-1, OMe), 40.1 (q, C-8, NMe), 36.0 (s, C-6, *t*Bu), 26.3 (q, *t*Bu, 3Me). **FT-IR (ATR):** $\tilde{\nu}$ [cm^{-1}] = 3259 (br w), 2955 (br w), 1767 (m), 1731 (m), 1662 (vs), 1597 (m), 1577 (m), 1481 (m), 1425 (m), 1337 (vs), 1234 (m), 1193 (m), 1149 (vs), 1042 (m), 879 (w), 786 (m), 696 (m), 540 (w). **HR-MS:** (ESI) = *m/z* calcd. for: $C_{19}H_{24}N_2O_4$ $[M+H]^+$ 345.1814 u, found: 345.1803 u. **$[a]_\lambda^T$:** (c = 1.20 g/100 mL, $CHCl_3$) = $[a]_D^{20}$: –20.00°.

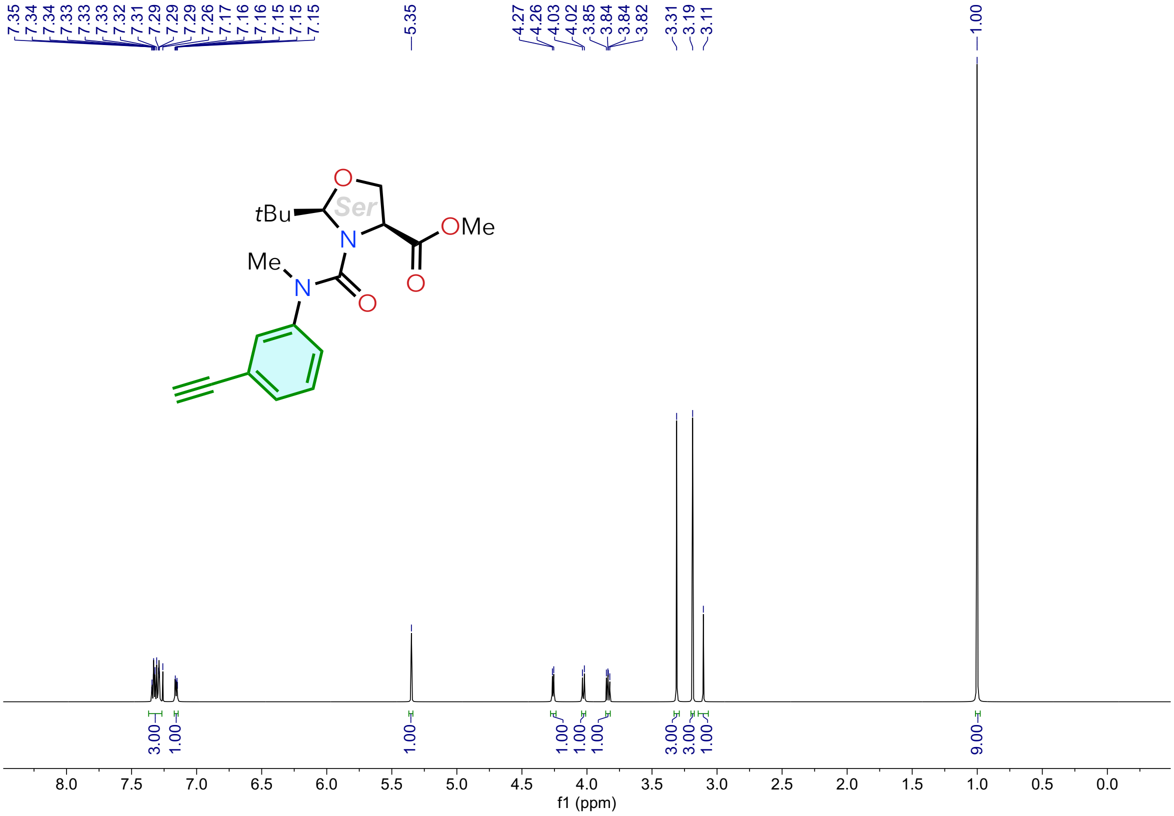

7.35
7.34
7.34
7.33
7.33
7.33
7.32
7.31
7.29
7.29
7.29
7.26
7.17
7.16
7.16
7.15
7.15
7.15
5.35
4.27
4.26
4.03
4.02
3.85
3.84
3.84
3.82
3.31
3.19
3.11
1.00
3.00
1.00
1.00
1.00
1.00
1.00
3.00
3.00
1.00
9.00
8.0 7.5 7.0 6.5 6.0 5.5 5.0 4.5 4.0 3.5 3.0 2.5 2.0 1.5 1.0 0.5 0.0
f1 (ppm)
O
tBu
Ser
N
OMe
O
Me
N
O

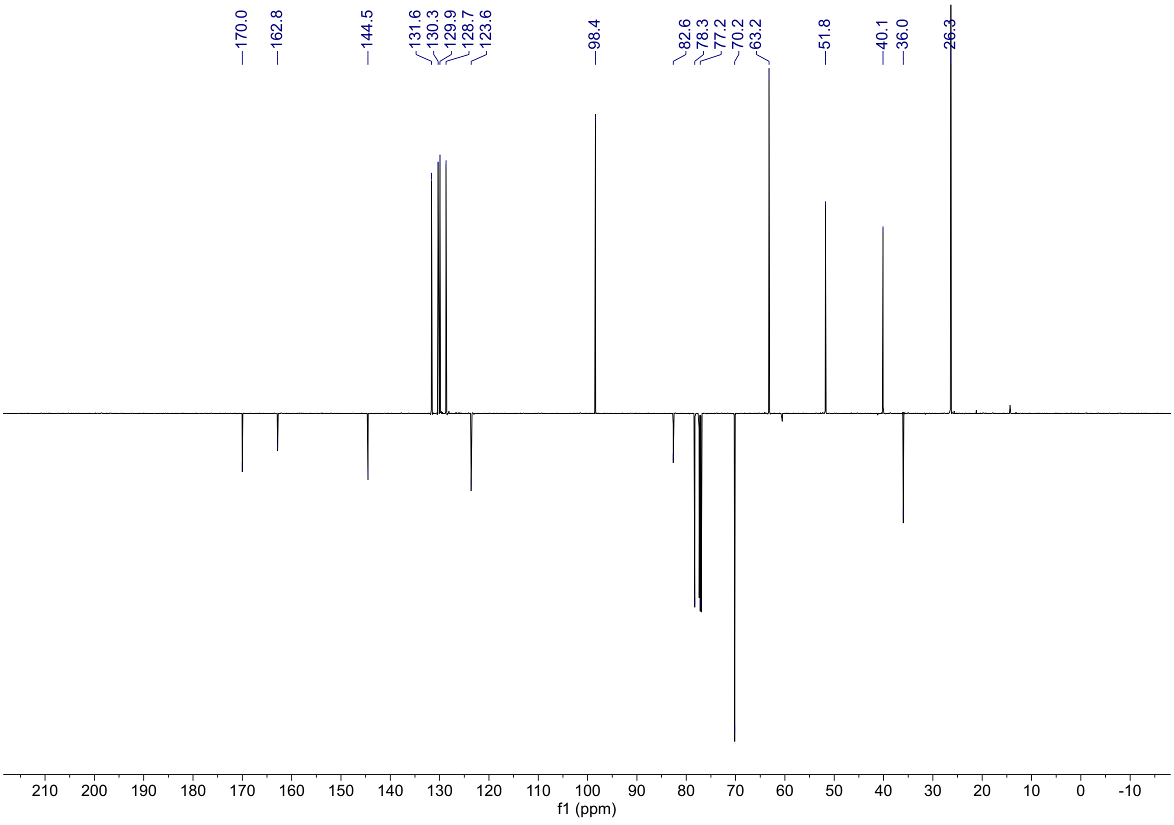

170.0
162.8
144.5
131.6
130.3
129.9
128.7
123.6
98.4
82.6
78.3
77.2
70.2
63.2
51.8
40.1
36.0
26.3
210 200 190 180 170 160 150 140 130 120 110 100 90 80 70 60 50 40 30 20 10 0 -10
f1 (ppm)

Methyl (2*R*,4*S*)-2-(*tert*-butyl)-3-(methyl(3-nitrophenyl)carbamoyl)oxazolidine-4-carboxylate (4j)

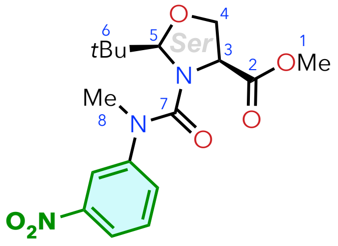

4j

Following **GP3**, *N*-chloroformyloxazolidine **3** (500-mg scale) was used as the carbamoyl chloride and *N*-methyl-4-nitroaniline (456 mg, 3.0 mmol, 1.5 equiv.) as the aniline in CH_2Cl_2. The title compound **4j** was obtained as a yellow solid (560 mg, 1.53 mmol, 77%) after purification by silica gel column chromatography (PE/EA, gradient elution).

Formula: $C_{17}H_{23}N_3O_6$, **MW:** 365.39 g/mol, **m.p.:** 142 – 145 °C. **TLC:** R_f = 0.20 (PE/EA 2:1), $KMnO_4$ stain. **^{1}H NMR** (500 MHz, $CDCl_3$): δ [ppm] = 8.08 – 8.06 (m, 2H, Ar), 7.53 – 7.52 (m, 2H, Ar), 5.36 (s, 1H, H-5), 4.26 (d, *J* 5.9 Hz, 1H, H-3), 4.07 (d, *J* 9.0 Hz, 1H, H-4a), 3.86 (dd, *J* 9.0, 6.8 Hz, 1H, H-4b), 3.26 (d, *J* 7.2 Hz, 6H, H-1, OMe, H-8, NMe), 1.01 (s, 9H, *t*Bu). **^{13}C NMR** (125 MHz, $CDCl_3$): δ [ppm] = 169.8 (s, C-2, ester), 162.6 (s, C-7, urea), 149.0 (s, Ar), 145.7 (s, Ar), 133.4 (d, Ar), 130.5 (d, Ar), 122.6 (d, Ar), 121.1 (d, Ar), 98.5 (d, C-5), 70.1 (t, C-4), 63.0 (d, C-3), 51.8 (q, C-1, OMe), 39.8 (q, C-8, NMe), 36.0 (s, C-6, *t*Bu), 26.3 (q, *t*Bu, 3Me). **FT-IR (ATR):** $\tilde{\nu}$ [cm^{-1}] = 2956 (br w), 1758 (m), 1665 (s), 1524 (s), 1478 (w), 1441 (w), 1375 (w), 1338 (vs), 1274 (w), 1255 (w), 1199 (m), 1179 (m), 1149 (s), 1041 (w), 1016 (w), 985 (m), 890 (w), 821 (w), 788 (w), 740 (m), 721 (m), 684 (s), 602 (w). **HR-MS:** (ESI) = *m*/*z* calcd. for: $C_{17}H_{24}N_3O_6$ $[M+H]^+$ 366.1665 u, found: 366.1668 u. **$[a]_\lambda^T$:** (c = 1.12 g/100 mL, $CHCl_3$) = $[a]_D^{20}$: –21.43°.

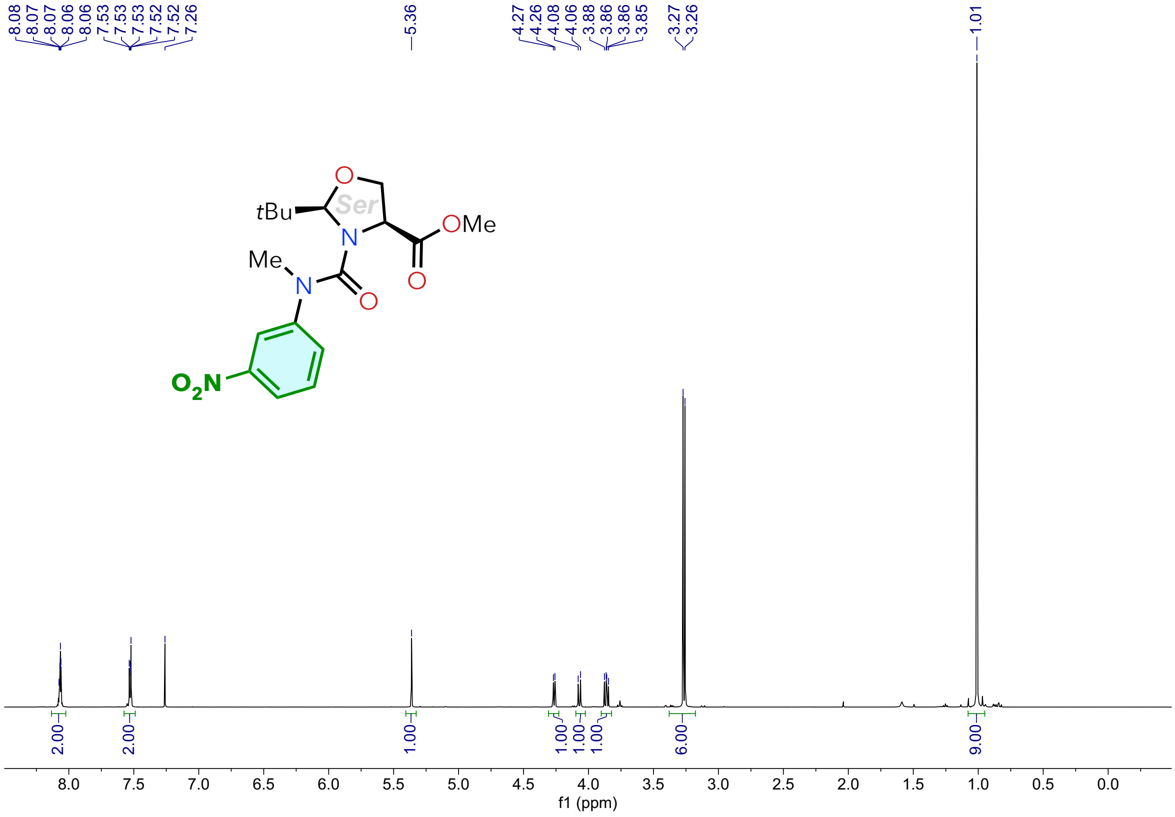
8.08
8.07
8.07
8.06
8.06
7.53
7.53
7.53
7.52
7.52
7.26
5.36
4.27
4.26
4.08
4.06
3.88
3.86
3.86
3.85
3.27
3.26
1.01
tBu
Ser
O
N
OMe
O
Me
N
O
O2N
2.00
2.00
1.00
1.00
1.00
1.00
6.00
9.00
f1 (ppm)

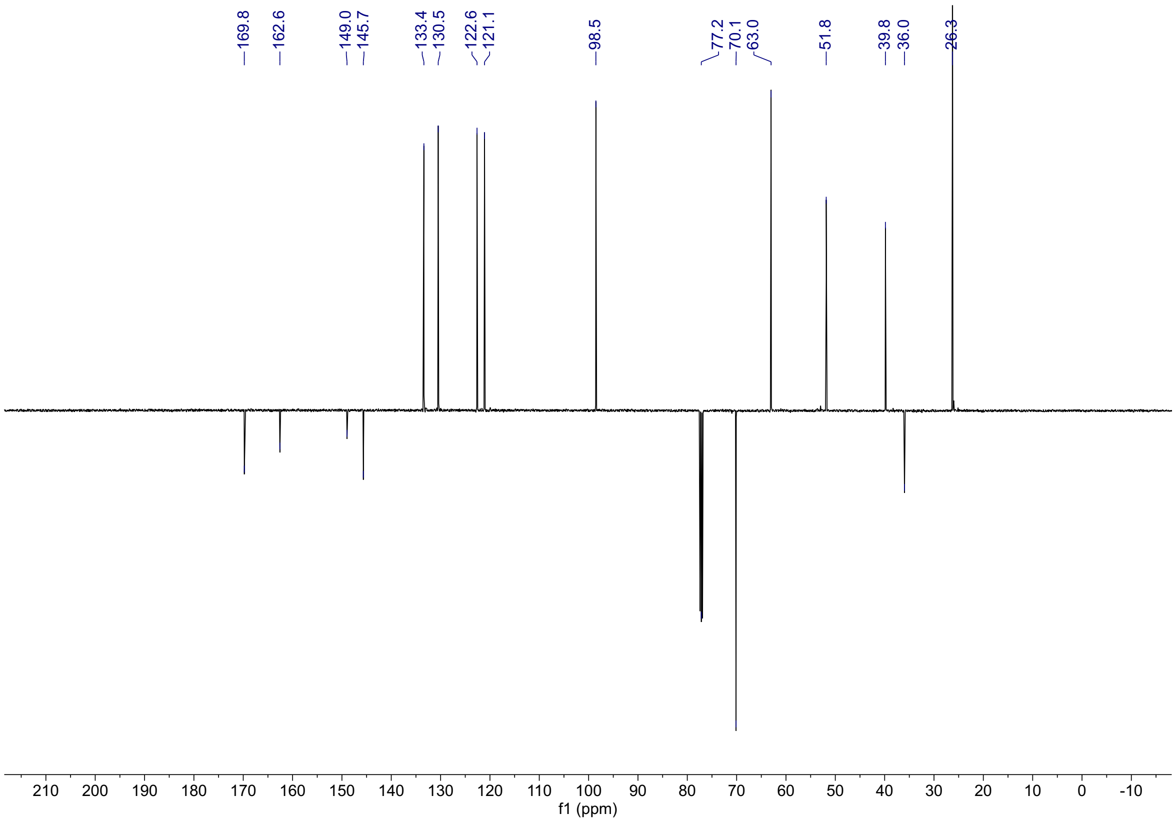
169.8
162.6
149.0
145.7
133.4
130.5
122.6
121.1
98.5
77.2
70.1
63.0
51.8
39.8
36.0
26.3
f1 (ppm)

Methyl (2*R*,4*S*)-2-(*tert*-butyl)-3-((3-methoxyphenyl)(methyl)carbamoyl)oxazolidine-4-carboxylate (4k)

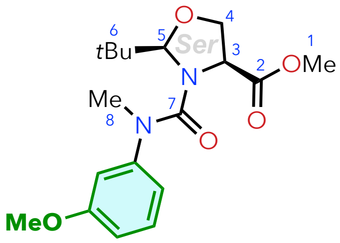

4k

Following **GP3**, *N*-chloroformyloxazolidine **3** (500-mg scale) was used as the carbamoyl chloride and 3-methoxy-*N*-methylaniline (0.39 mL, 3.0 mmol, 1.5 equiv.) as the aniline in CH_2Cl_2. The title compound **4k** was obtained as a burgundy solid (570 mg, 1.63 mmol, 81%) after purification by silica gel column chromatography (PE/EA, gradient elution).

Formula: $C_{18}H_{26}N_2O_5$, **MW:** 350.42 g/mol, **m.p.:** 80 – 84 °C. **TLC:** R_f = 0.23 (PE/EA 2:1), $KMnO_4$ stain. **^{1}H NMR** (500 MHz, $CDCl_3$): δ [ppm] = 7.23 (t, *J* 8.1 Hz, 1H, Ar), 6.75 (dddd, *J* 7.2, 6.2, 2.3, 0.9 Hz, 2H, Ar), 6.69 (t, *J* 2.2 Hz, 1H, Ar), 5.36 (s, 1H, H-5), 4.30 (dd, *J* 6.8 Hz, 1H, H-3), 4.01 (d, *J* 8.9 Hz, 1H, H-4a), 3.83 (dd, *J* 8.9, 7.0 Hz, 1H, H-4b), 3.80 (s, 3H, ArOMe), 3.27 (s, 3H, H-1, OMe), 3.20 (s, 3H, H-8, NMe), 1.01 (s, 9H, *t*Bu). **^{13}C NMR** (125 MHz, $CDCl_3$): δ [ppm] = 170.1 (s, C-2, ester), 162.9 (s, C-7, urea), 160.5 (s, Ar), 145.5 (s, Ar), 130.3 (d, Ar), 120.0 (d, Ar), 113.4 (d, Ar), 112.8 (d, Ar), 98.4 (d, C-5), 70.2 (t, C-4), 63.2 (d, C-3), 55.4 (q, Ar, OMe), 51.6 (q, C-1, OMe), 40.2 (q, C-8, NMe), 36.0 (s, C-6, *t*Bu), 26.4 (q, *t*Bu, 3Me). **FT-IR (ATR):** $\tilde{\nu}$ [cm^{-1}] = 2954 (br w), 1767 (m), 1728 (w), 1660 (s), 1597 (m), 1488 (m), 1340 (s), 1230 (m), 1149 (vs), 1099 (m), 1041 (m), 985 (w), 949 (w), 855 (w), 781 (w), 699 (m), 527 (w). **HR-MS:** (ESI) = *m/z* calcd. for: $C_{18}H_{27}N_2O_5$ $[M+H]^+$ 351.1920 u, found: 351.1903 u. **$[a]_\lambda^T$:** (c = 1.06 g/100 mL, $CHCl_3$) = $[a]_D^{20}$: –19.62°.

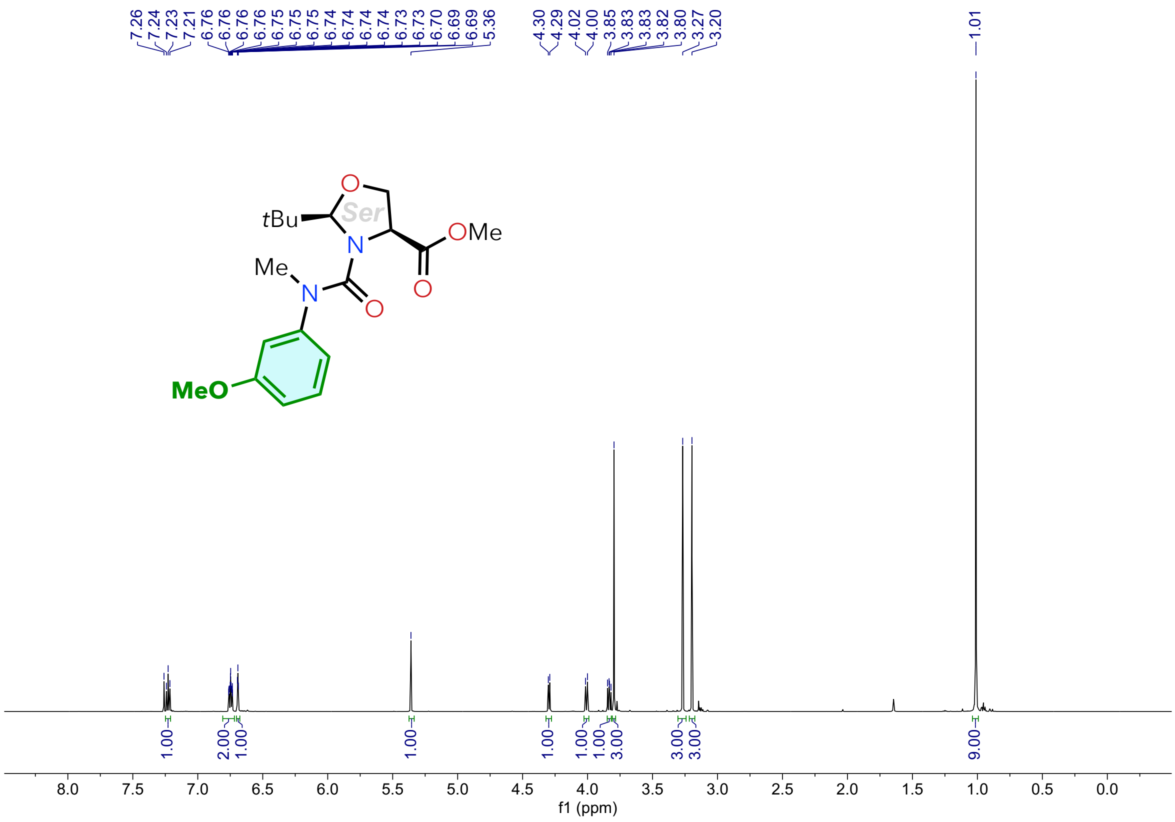

7.26
7.24
7.23
7.21
6.76
6.76
6.76
6.75
6.75
6.74
6.74
6.74
6.74
6.73
6.73
6.70
6.69
6.69
5.36
4.30
4.29
4.02
4.00
3.85
3.83
3.83
3.82
3.80
3.27
3.20
1.01
tBu
O
Ser
N
OMe
O
Me
N
O
MeO
1.00
2.00
1.00
1.00
1.00
1.00
1.00
3.00
3.00
3.00
9.00
f1 (ppm)

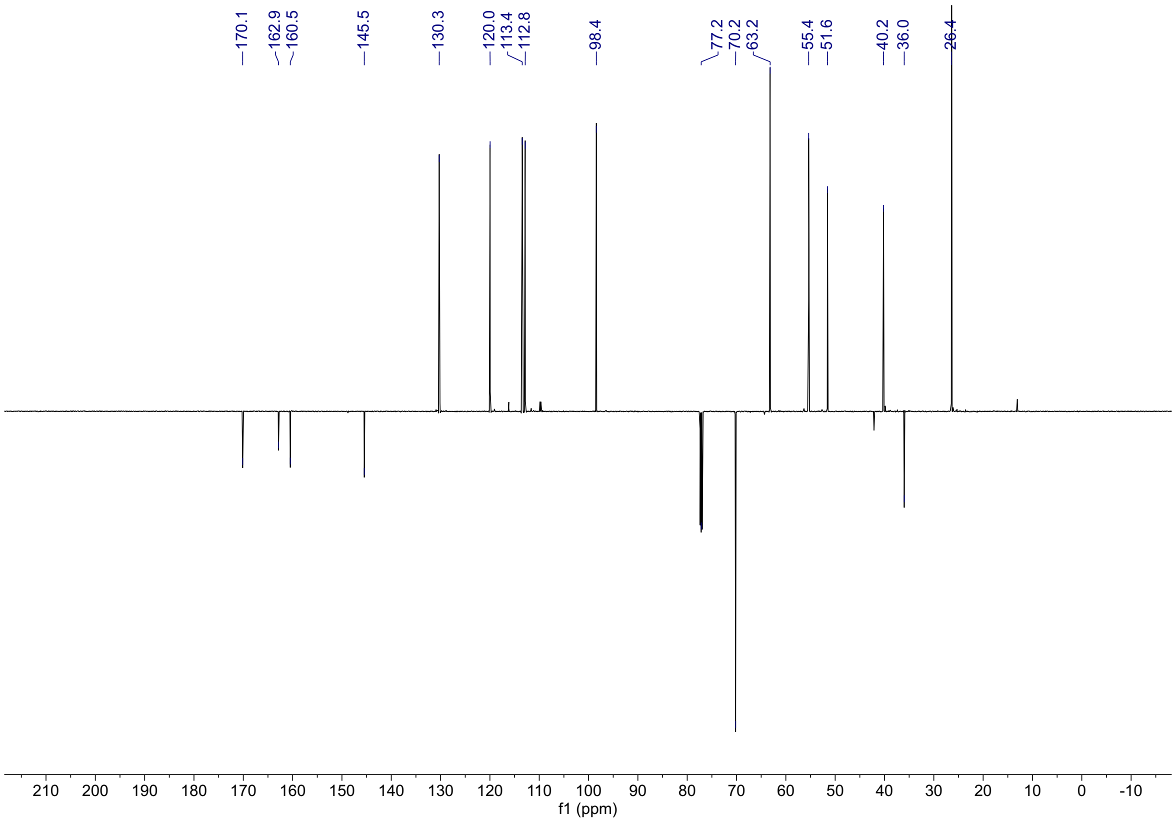

170.1
162.9
160.5
145.5
130.3
120.0
113.4
112.8
98.4
77.2
70.2
63.2
55.4
51.6
40.2
36.0
26.4
f1 (ppm)

Methyl (2*R*,4*S*)-2-(*tert*-butyl)-3-((2-methoxyphenyl)(methyl)carbamoyl)oxazolidine-4-carboxylate (4l)

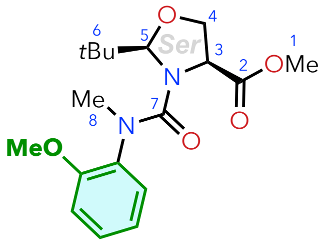

4l

Following **GP3**, *N*-chloroformyloxazolidine **3** (500-mg scale) was used as the carbamoyl chloride and 2-methoxy-*N*-methylaniline (412 mg, 3.0 mmol, 1.5 equiv.) as the aniline in CH_2Cl_2. The title compound **4l** was obtained as a yellow oil (642 mg, 1.83 mmol, 92%) after purification by silica gel column chromatography (PE/EA, gradient elution).

Formula: $C_{18}H_{26}N_2O_5$, **MW:** 350.42 g/mol. **TLC:** R_f = 0.32 (PE/EA 2:1), $KMnO_4$ stain. **^{1}H NMR** (600 MHz, $CDCl_3$): δ [ppm] = 7.24 (td, *J* 8.1, 1.6 Hz, 1H, Ar), 7.10 (dd, *J* 7.7, 1.6 Hz, 1H, Ar), 6.91 (t, *J* 7.2 Hz, 1H, Ar), 6.87 (d, *J* 8.3 Hz, 1H, Ar), 5.34 (s, 1H, H-5), 4.31 (dd, *J* 6.8 Hz, 1H, H-3), 4.01 (d, *J* 8.8 Hz, 1H, H-4a), 3.84 (s, 3H, ArOMe), 3.82 (d, *J* 8.8 Hz, 1H, H-4b), 3.22 (s, 3H, H-1, OMe), 3.07 (s, 3H, H-8, NMe), 0.97 (s, 9H, *t*Bu). **^{13}C NMR** (150 MHz, $CDCl_3$): δ [ppm] = 170.3 (s, C-2, ester), 163.5 (s, C-7, urea), 155.2 (s, Ar), 132.3 (s, Ar), 130.7 (d, Ar), 128.9 (d, Ar), 121.1 (d, Ar), 111.7 (d, Ar), 98.4 (d, C-5), 70.2 (t, C-4), 62.6 (d, C-3), 55.4 (q, Ar, OMe), 51.5 (q, C-1, OMe), 38.5 (q, C-8, NMe), 35.9 (s, C-6, *t*Bu), 26.1 (q, *t*Bu, 3Me). **FT-IR (ATR):** $\tilde{\nu}$ [cm^{-1}] = 2958 (br w), 1768 (m), 1727 (w), 1659 (s), 1501 (m), 1340 (m), 1277 (m), 1236 (m), 1149 (vs), 1044 (m), 1027 (m), 984 (m), 931 (w), 879 (w), 748 (m), 681 (w), 609 (w). **HR-MS:** (ESI) = *m*/*z* calcd. for: $C_{18}H_{27}N_2O_5$ $[M+H]^+$ 351.1920 u, found: 351.1904 u. **$[a]_\lambda^T$:** (c = 0.99 g/100 mL, $CHCl_3$) = $[a]_D^{20}$: –19.39°.

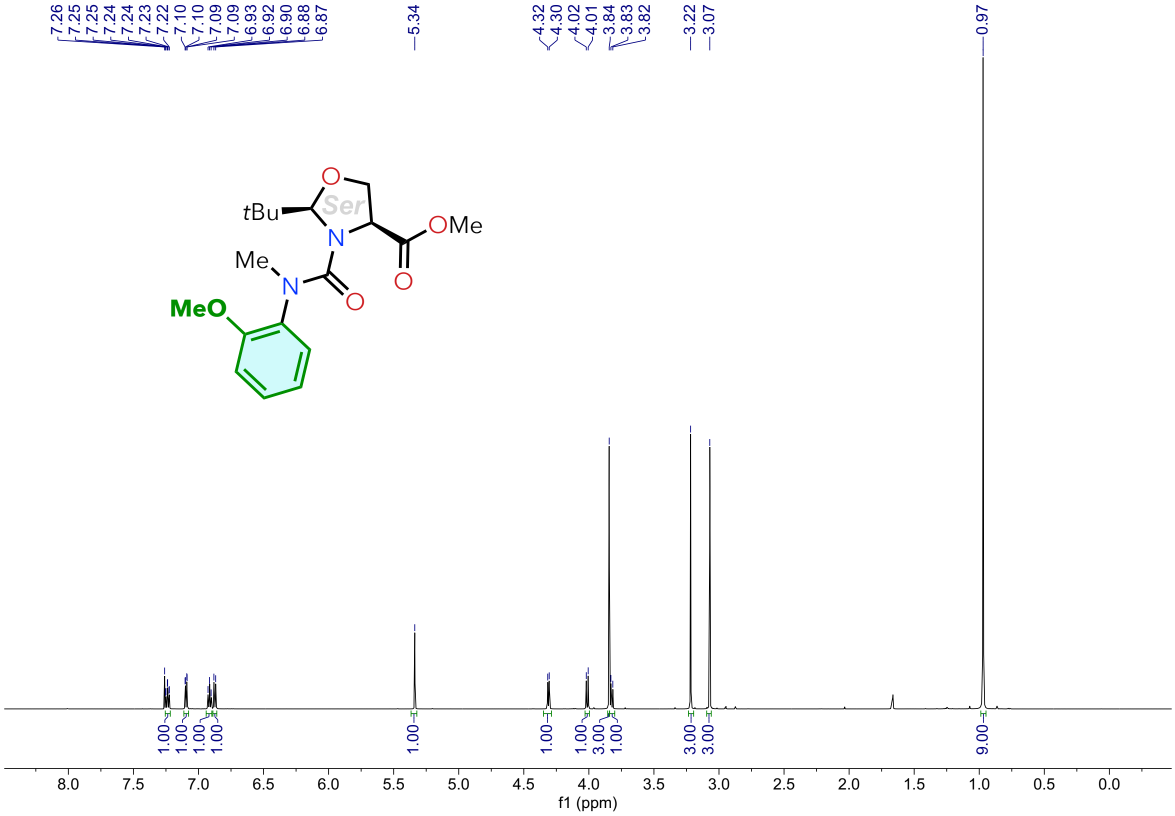
7.26 7.25 7.25 7.24 7.24 7.23 7.22 7.10 7.10 7.09 7.09 6.93 6.92 6.90 6.88 6.87
5.34
4.32 4.30 4.02 4.01 3.84 3.83 3.82
3.22 3.07
0.97
tBu
Ser
OMe
Me
MeO
1.00 1.00 1.00 1.00
1.00
1.00 1.00 3.00 1.00
3.00 3.00
9.00
f1 (ppm)

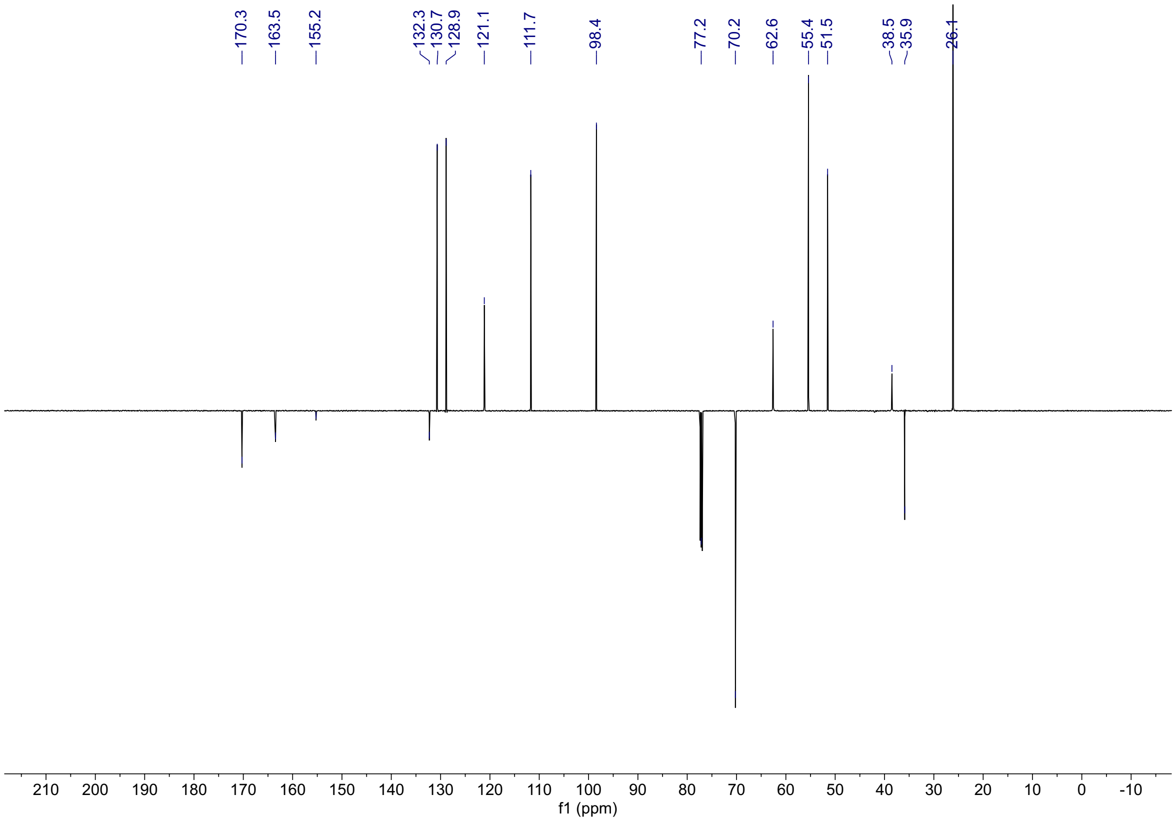
170.3 163.5 155.2
132.3 130.7 128.9 121.1
111.7
98.4
77.2 70.2 62.6 55.4 51.5
38.5 35.9 26.1
f1 (ppm)

Methyl (2*R*,4*S*)-2-(*tert*-butyl)-3-((2,4-dichlorophenyl)(methyl)carbamoyl)oxazolidine-4-carboxylate (4m)

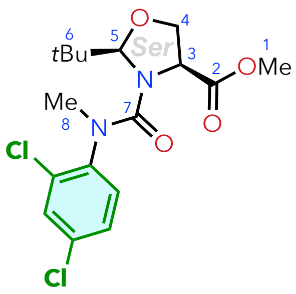

4m

Following **GP3**, *N*-chloroformyloxazolidine **3** (500-mg scale) was used as the carbamoyl chloride and 2,4-dichloro-*N*-methylaniline (0.53 mL, 3.0 mmol, 1.5 equiv.) as the aniline in CH_2Cl_2. The title compound **4m** was obtained as a white solid (509 mg, 1.31 mmol, 65%) after purification by silica gel column chromatography (PE/EA, gradient elution).

Formula: $C_{17}H_{22}Cl_2N_2O_4$, **MW:** 389.27 g/mol, **m.p.:** 93 – 96 °C. **TLC:** R_f = 0.47 (PE/EA 2:1), $KMnO_4$ stain. **^{1}H NMR** (500 MHz, $CDCl_3$): δ [ppm] = 7.41 (d, *J* 2.3 Hz, 1H, Ar), 7.28 (dd, *J* 8.5, 2.3 Hz, 1H, Ar), 7.15 (d, *J* 8.5 Hz, 1H, Ar), 5.34 (s, 1H, H-5), 4.27 (d, *J* 6.7 Hz, 1H, H-3), 4.09 (d, *J* 8.9 Hz, 1H, H-4a), 3.87 (dd, *J* 8.9, 6.9 Hz, 1H, H-4b), 3.33 (s, 3H, H-1, OMe), 3.10 (s, 3H, H-8, NMe), 0.98 (s, 9H, *t*Bu). **^{13}C NMR** (125 MHz, $CDCl_3$): δ [ppm] = 169.7 (s, C-2, ester), 162.5 (s, C-7, urea), 140.0 (s, Ar), 134.0 (s, Ar), 133.8 (s, Ar), 132.2 (d, Ar), 130.2 (d, Ar), 128.8 (d, Ar), 98.7 (d, C-5), 70.2 (t, C-4), 62.0 (d, C-3), 51.9 (q, C-1, OMe), 38.3 (q, C-8, NMe), 36.0 (s, C-6, *t*Bu), 26.2 (q, *t*Bu, 3Me). **FT-IR (ATR):** $\tilde{\nu}$ [cm^{-1}] = 2955 (br w), 1766 (m), 1731 (w), 1667 (vs), 1480 (s), 1336 (s), 1303 (m9, 1195 (m9, 1147 (vs), 1099 (m), 1057 (m), 1041 (m), 983 (m), 930 (w), 868 8w), 837 (w), 804 (w), 786 (w), 669 (w), 581 (m), 508 (w). **HR-MS:** (ESI) = *m*/*z* calcd. for: $C_{17}H_{23}{}^{35}Cl_2N_2O_4$ $[M+H]^+$ 389.1035 u, found: 389.1029 u. **$[a]_{\lambda}^{T}$:** (c = 0.94 g/100 mL, $CHCl_3$) = $[a]_D^{20}$: –20.43°.

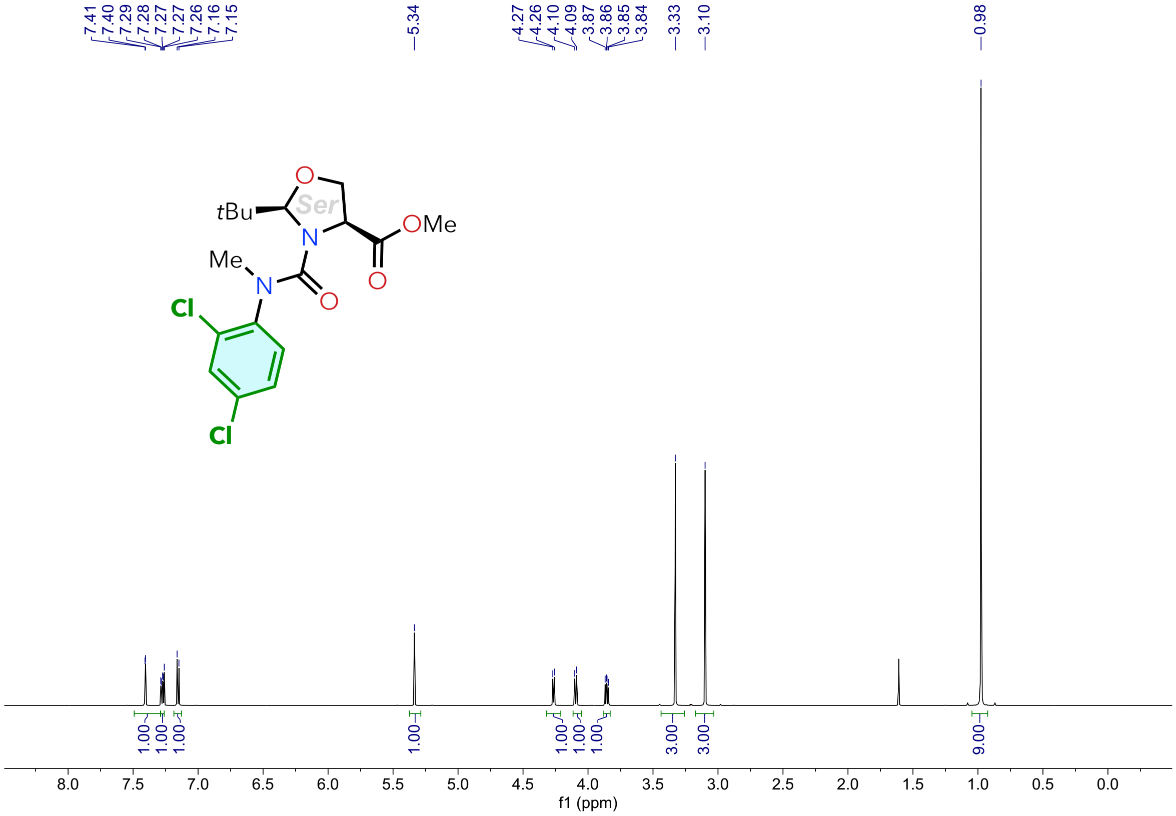
7.41
7.40
7.29
7.28
7.27
7.27
7.26
7.16
7.15
5.34
4.27
4.26
4.10
4.09
3.87
3.86
3.85
3.84
3.33
3.10
0.98
tBu
O
Ser
N
OMe
O
Me
N
O
Cl
Cl
1.00
1.00
1.00
1.00
1.00
1.00
1.00
3.00
3.00
9.00
8.0 7.5 7.0 6.5 6.0 5.5 5.0 4.5 4.0 3.5 3.0 2.5 2.0 1.5 1.0 0.5 0.0
f1 (ppm)

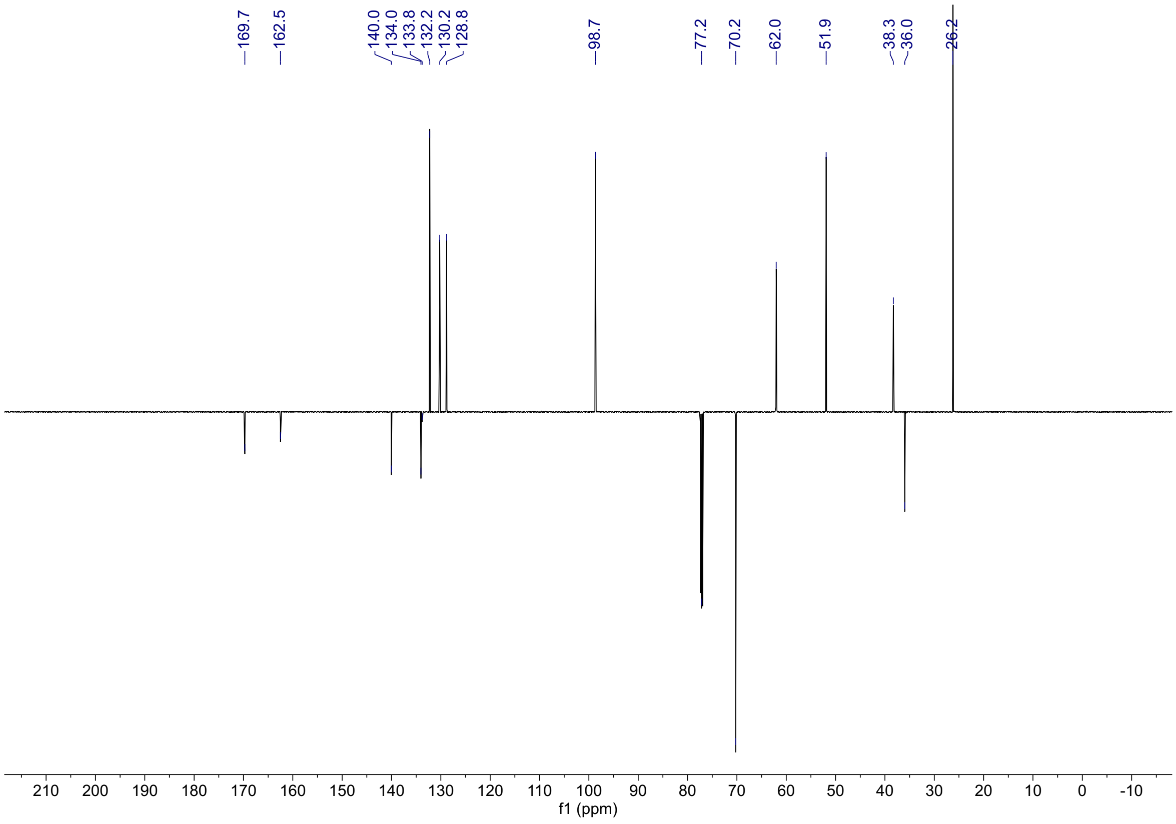
169.7
162.5
140.0
134.0
133.8
132.2
130.2
128.8
98.7
77.2
70.2
62.0
51.9
38.3
36.0
26.2
210 200 190 180 170 160 150 140 130 120 110 100 90 80 70 60 50 40 30 20 10 0 -10
f1 (ppm)

Methyl (2*R*,4*S*)-2-(*tert*-butyl)-3-((3,5-dichlorophenyl)(methyl)carbamoyl)oxazolidine-4-carboxylate (4n)

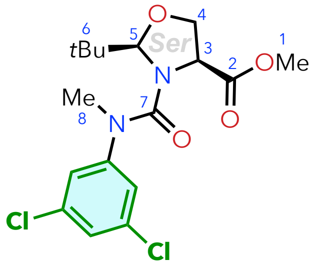

4n

Following **GP3**, *N*-chloroformyloxazolidine **3** (500-mg scale) was used as the carbamoyl chloride and 3,5-dichloro-*N*-methylaniline (0.53 mg, 3.0 mmol, 1.5 equiv.) as the aniline in CH_2Cl_2. The title compound **4n** was obtained as a white solid (610 mg, 1.57 mmol, 78%) after purification by silica gel column chromatography (PE/EA, gradient elution).

Formula: $C_{17}H_{22}Cl_2N_2O_4$, **MW:** 389.27 g/mol, **m.p.:** 85 – 88 °C. **TLC:** R_f = 0.27 (PE/EA 2:1), $KMnO_4$ stain. **^{1}H NMR** (600 MHz, $CDCl_3$): δ [ppm] = 7.21 (t, *J* 1.8 Hz, 1H, Ar), 7.08 (d, *J* 1.8 Hz, 1H, Ar), 5.33 (s, 1H, H-5), 4.22 (d, *J* 6.3 Hz, 1H, H-3), 4.07 (d, *J* 9.0 Hz, 1H, H-4a), 3.84 (dd, *J* 9.0, 6.9 Hz, 1H, H-4b), 3.41 (s, 3H, H-1, OMe), 3.19 (s, 3H, H-8, NMe), 1.00 (s, 9H, *t*Bu). **^{13}C NMR** (150 MHz, $CDCl_3$): δ [ppm] = 169.9 (s, C-2, ester), 162.4 (s, C-7, urea), 146.3 (s, Ar), 135.7 (s, Ar), 126.6 (d, Ar), 126.3 (d, 2Ar), 128.8 (d, Ar), 98.5 (d, C-5), 70.2 (t, C-4), 63.1 (d, C-3), 52.0 (q, C-1, OMe), 39.8 (q, C-8, NMe), 36.0 (s, C-6, *t*Bu), 26.3 (q, *t*Bu, 3Me). **FT-IR (ATR):** $\tilde{\nu}$ [cm^{-1}] = 2956 (br w), 1760 (m), 1650 (s), 1507 (vs), 1430 (m), 1375 (m), 1343 (m), 1217 (s), 1198 (s), 1147 (s), 985 (m), 933 (w), 842 (s), 810 (w), 786 (w), 722 (w), 667 (m), 593 (m), 535 (w). **HR-MS:** (ESI) = *m*/*z* calcd. for: $C_{17}H_{23}{}^{35}Cl_2N_2O_4$ $[M+H]^+$ 389.1035 u, found: 389.1034 u. **$[a]_\lambda^T$:** (c = 1.15 g/100 mL, $CHCl_3$) = $[a]_D^{20}$: –18.09°.

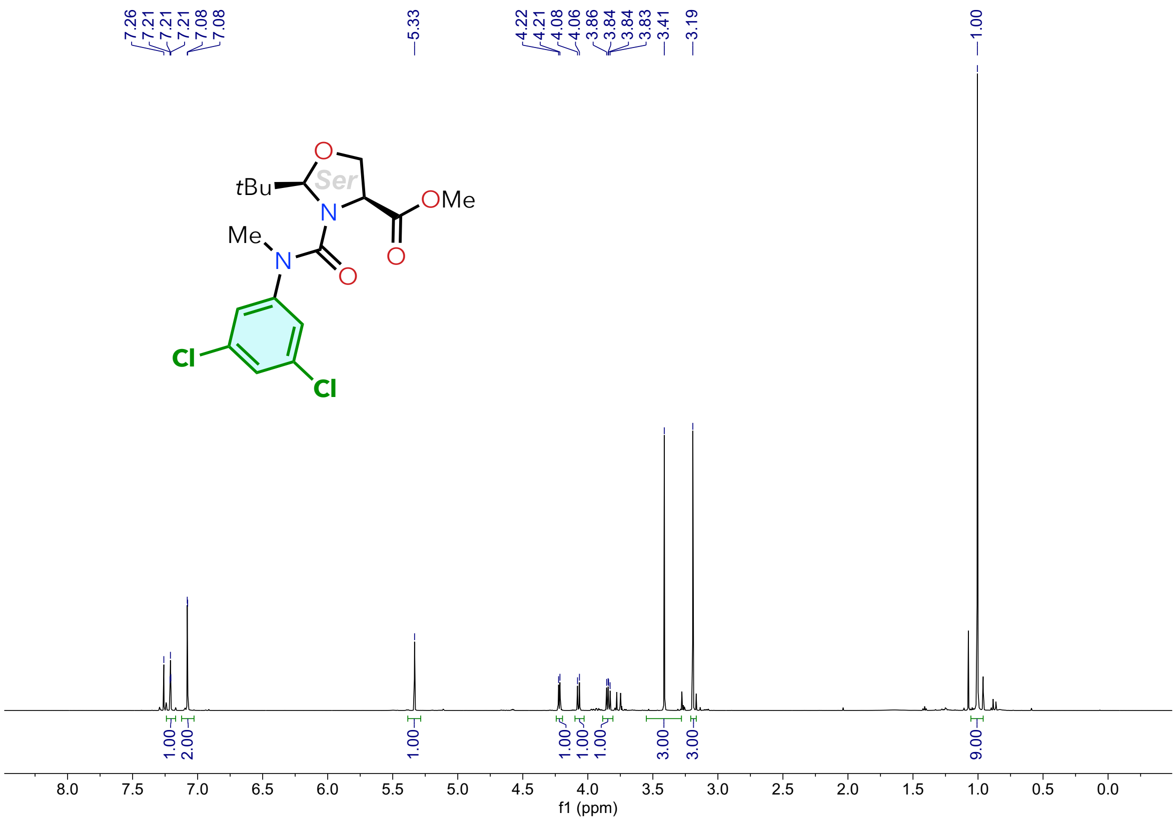

7.26
7.21
7.21
7.21
7.08
7.08
5.33
4.22
4.21
4.08
4.06
3.86
3.84
3.84
3.83
3.41
3.19
1.00
tBu
Ser
O
N
OMe
Me
N
Cl
Cl
1.00
2.00
1.00
1.00
1.00
1.00
3.00
3.00
9.00
8.0
7.5
7.0
6.5
6.0
5.5
5.0
4.5
4.0
3.5
3.0
2.5
2.0
1.5
1.0
0.5
0.0
f1 (ppm)

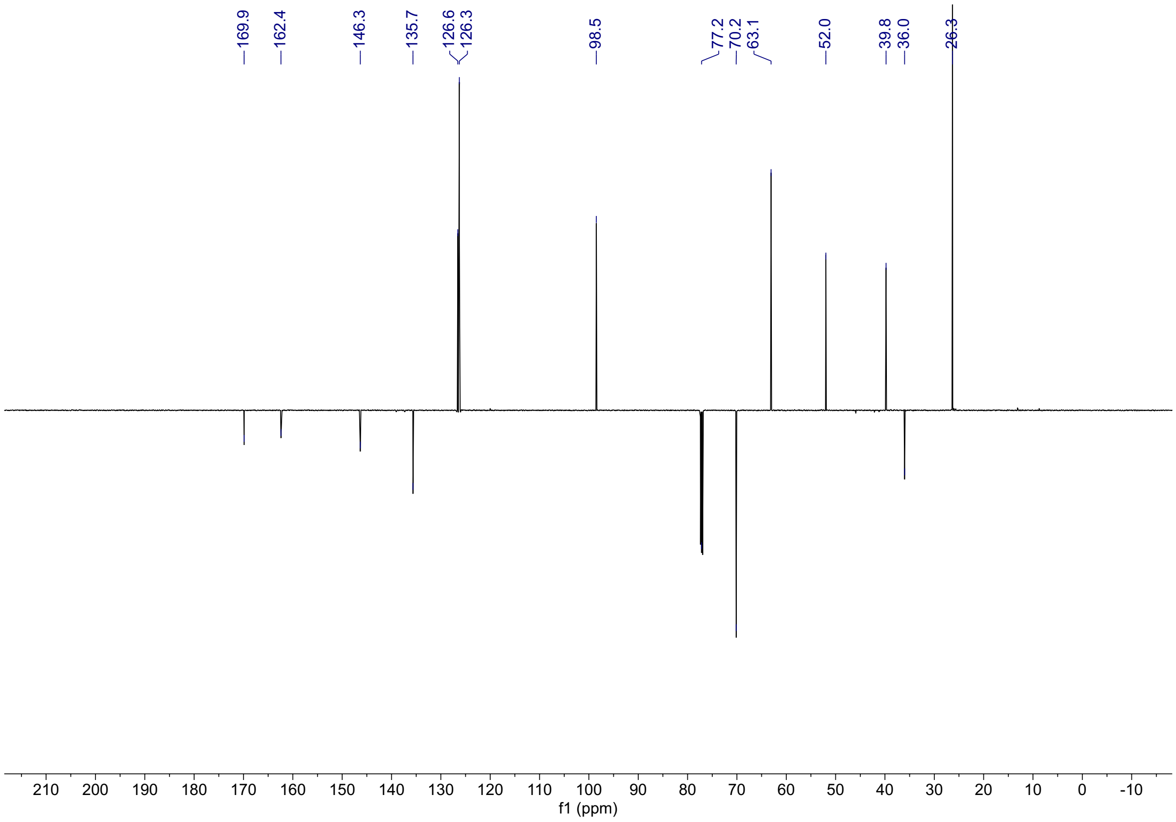

169.9
162.4
146.3
135.7
126.6
126.3
98.5
77.2
70.2
63.1
52.0
39.8
36.0
26.3
210
200
190
180
170
160
150
140
130
120
110
100
90
80
70
60
50
40
30
20
10
0
-10
f1 (ppm)

Methyl (2*R*,4*S*)-3-((4-bromo-3-fluorophenyl)(methyl)carbamoyl)-2-(*tert*-butyl)oxazolidine-4-carboxylate (4o)

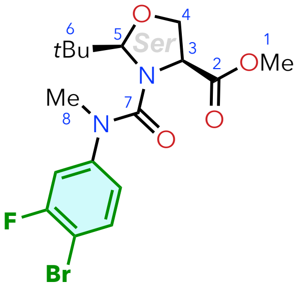

4o

Following **GP3**, *N*-chloroformyloxazolidine **3** (500-mg scale) was used as the carbamoyl chloride and 4-bromo-3-fluoro-*N*-methylaniline (0.39 mL, 3.0 mmol, 1.5 equiv.) as the aniline in CH_2Cl_2. The title compound **4o** was obtained as a beige solid (607 mg, 1.45 mmol, 73%) after purification by silica gel column chromatography (PE/EA, gradient elution).

Formula: $C_{17}H_{22}BrFN_2O_4$, **MW:** 417.28 g/mol, **m.p.:** 94 – 97 °C. **TLC:** R_f = 0.30 (PE/EA 2:1), $KMnO_4$ stain. **^{1}H NMR** (500 MHz, $CDCl_3$): δ [ppm] = 7.50 (t, *J* 8.1 Hz, 1H, Ar), 6.96 (dd, *J* 9.4, 2.4 Hz, 1H, Ar), 6.87 (dd, *J* 8.5, 2.3 Hz, 1H, Ar), 5.34 (s, 1H, H-5), 4.22 (d, *J* 6.7 Hz, 1H, H-3), 4.05 (d, *J* 9.0 Hz, 1H, H-4a), 3.84 (dd, *J* 8.9, 6.9 Hz, 1H, H-4b), 3.35 (s, 3H, H-1, OMe), 3.20 (s, 3H, H-8, NMe), 1.00 (s, 9H, *t*Bu). **^{13}C NMR** (125 MHz, $CDCl_3$): δ [ppm] = 170.0 (s, C-2, ester), 162.4 (s, C-7, urea), 160.1 (s, Ar), 158.5 (s, Ar), 145.0 (s, *d J* 8.5 Hz, Ar), 134.2 (d, *d J* 1.0 Hz, Ar), 124.5 (d, *d J* 3.4 Hz, Ar), 116.3 (d, *d J* 23.5 Hz, Ar), 106.8 (s, *d J* 20.9 Hz, Ar), 98.5 (d, C-5), 70.2 (t, C-4), 63.0 (d, C-3), 51.9 (q, C-1, OMe), 39.9 (q, C-8, NMe), 36.0 (s, C-6, *t*Bu), 26.3 (q, *t*Bu, 3Me). **^{19}F NMR** (471 MHz, $CDCl_3$, C_6F_6 ref.): δ [ppm] = –107.7 (t, *J* 8.5 Hz, F). **FT-IR (ATR):** $\tilde{\nu}$ [cm^{-1}] = 2968 (br w), 1755 (s), 1667 (vs), 1582 (w), 1485 (m), 1367 (m), 1216 (m), 1197 (s), 1172 (m), 1151 (vs), 1104 (m), 1042 (m), 954 (m), 866 (m), 787 (w), 701 (w), 668 (m), 637 (m), 526 (w). **HR-MS:** (ESI) = *m/z* calcd. for: $C_{17}H_{23}{}^{79}BrFN_2O_4$ $[M+H]^+$ 417.0825 u, found: 417.0806 u. **$[a]_\lambda^T$:** (c = 1.15 g/100 mL, $CHCl_3$) = $[a]_D^{20}$: –20.52 °.

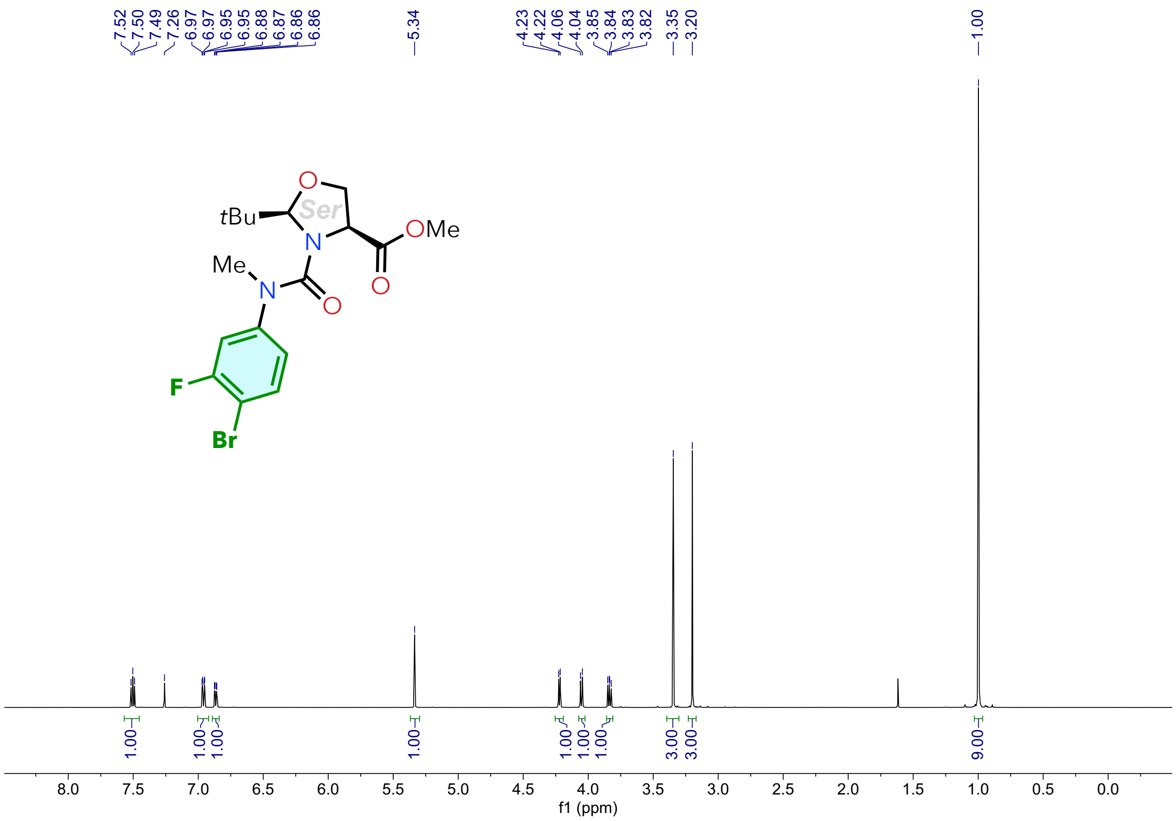

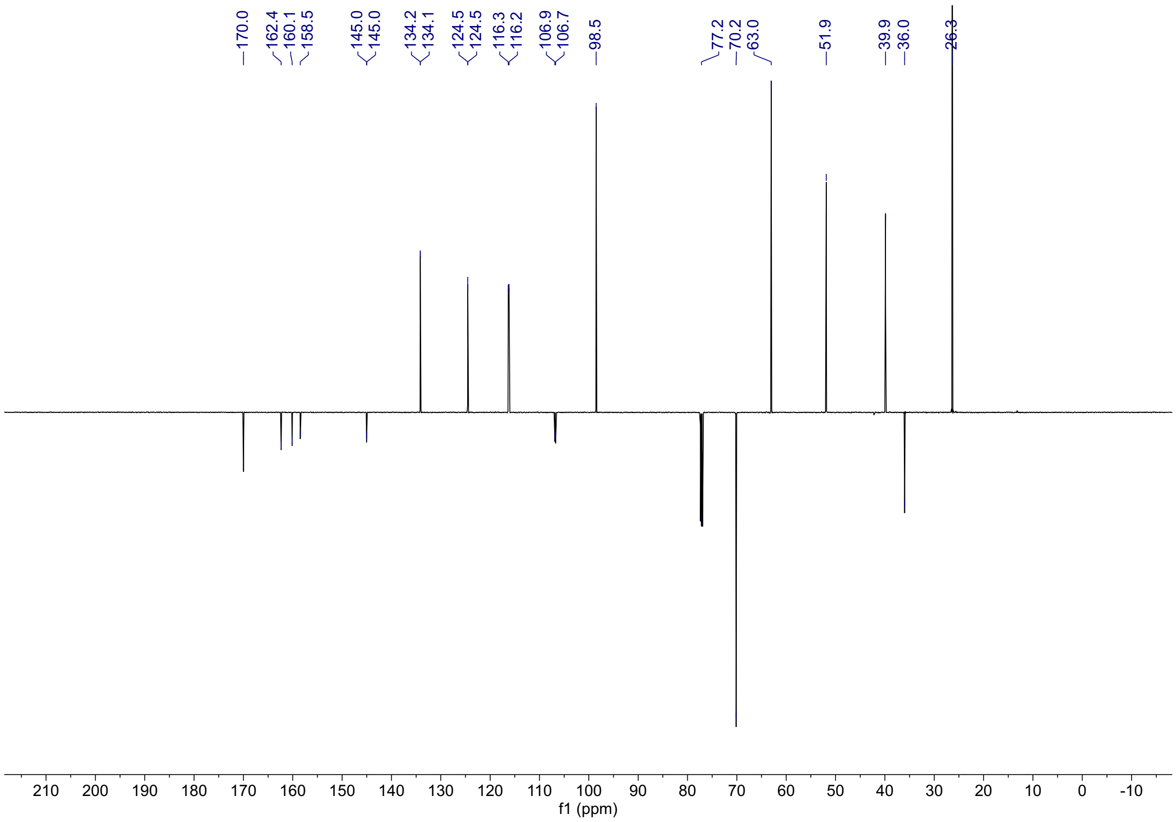

Methyl (2*R*,4*S*)-2-(*tert*-butyl)-3-((4-chloro-2-methoxyphenyl)(methyl)carbamoyl)-oxazolidine-4-carboxylate (4p)

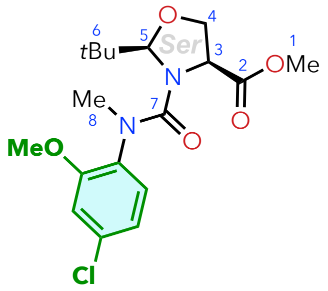

4p

Following **GP3**, *N*-chloroformyloxazolidine **3** (500-mg scale) was used as the carbamoyl chloride and 4-chloro-2-methoxy-*N*-methylaniline (0.44 mL, 3.0 mmol, 1.5 equiv.) as the aniline in CH_2Cl_2. The title compound **4p** was obtained as a white solid (658 mg, 1.71 mmol, 85%) after purification by silica gel column chromatography (PE/EA, gradient elution).

Formula: $C_{18}H_{25}ClN_2O_5$, **MW:** 384.86 g/mol, **m.p.:** 107 – 110 °C. **TLC:** R_f = 0.48 (PE/EA 2:1), $KMnO_4$ stain. **^{1}H NMR** (500 MHz, $CDCl_3$): δ [ppm] = 7.02 (d, *J* 8.3 Hz, 1H, Ar), 6.90 (dd, *J* 8.3, 2.2 Hz, 1H, Ar), 6.86 (d, *J* 2.2 Hz, 1H, Ar), 5.32 (s, 1H, H-5), 4.24 (d, *J* 6.9 Hz, 1H, H-3), 4.03 (d, *J* 9.2 Hz, 1H, H-4a), 3.84 – 3.81 (m, 4H, ArOMe, H-4b), 3.30 (s, 3H, H-1, OMe), 3.05 (s, 3H, H-8, NMe), 0.96 (s, 9H, *t*Bu). **^{13}C NMR** (125 MHz, $CDCl_3$): δ [ppm] = 170.3 (s, C-2, ester), 163.2 (s, C-7, urea), 155.8 (s, Ar), 134.2 (s, Ar), 131.5 (d, Ar), 131.0 (s, Ar), 121.2 (d, Ar), 112.7 (d, Ar), 98.5 (d, C-5), 70.2 (t, C-4), 62.5 (d, C-3), 55.8 (q, MeOAr), 51.7 (q, C-1, OMe), 38.4 (q, C-8, NMe), 35.9 (s, C-6, *t*Bu), 26.1 (q, *t*Bu, 3Me). **FT-IR (ATR):** $\tilde{\nu}$ [cm^{-1}] = 2967 (br w), 1768 (m), 1664 (vs), 1496 (m), 1418 (w), 1365 (m), 1338 (s), 1248 (m), 1195 (m), 1175 (m), 1152 (vs), 1093 (w), 1029 (m), 983 (m), 930 (w), 876 (m), 844 (s), 789 (w), 664 (w), 606 (m), 567 (w). **HR-MS:** (ESI) = *m*/*z* calcd. for: $C_{18}H_{26}{}^{35}ClN_2O_5$ $[M+H]^+$ 385.1530 u, found: 385.1525 u. **$[a]_\lambda^T$:** (c = 1.08 g/100 mL, $CHCl_3$) = $[a]_D^{20}$: –19.63°.

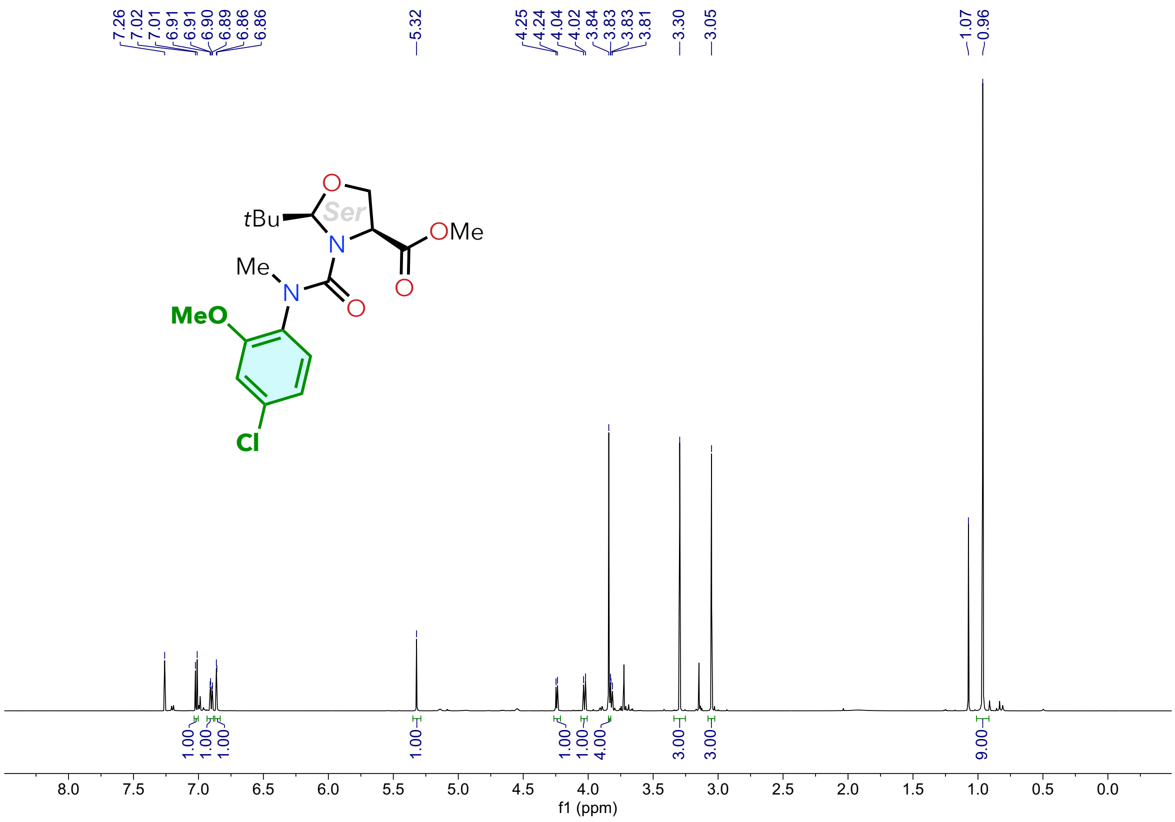
7.26
7.02
7.01
6.91
6.91
6.90
6.89
6.86
6.86
5.32
4.25
4.24
4.04
4.02
3.84
3.83
3.83
3.81
3.30
3.05
1.07
0.96
tBu
Ser
O
N
OMe
O
Me
N
O
MeO
Cl
1.00
1.00
1.00
1.00
1.00
1.00
4.00
3.00
3.00
9.00
8.0
7.5
7.0
6.5
6.0
5.5
5.0
4.5
4.0
3.5
3.0
2.5
2.0
1.5
1.0
0.5
0.0
f1 (ppm)

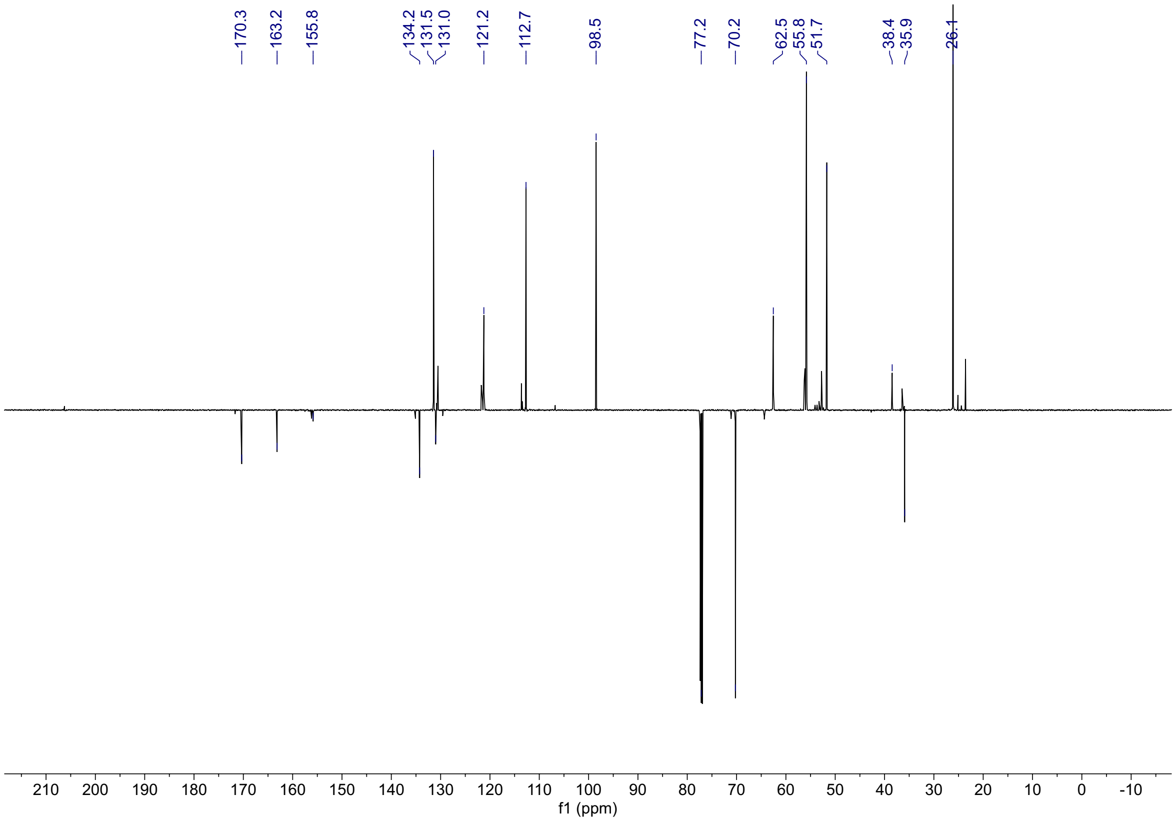
170.3
163.2
155.8
134.2
131.5
131.0
121.2
112.7
98.5
77.2
70.2
62.5
55.8
51.7
38.4
35.9
26.1
210
200
190
180
170
160
150
140
130
120
110
100
90
80
70
60
50
40
30
20
10
0
-10
f1 (ppm)

Methyl (2*R*,4*S*)-2-(*tert*-butyl)-3-(methyl(pyridin-2-yl)carbamoyl)oxazolidine-4-carboxylate (4q)

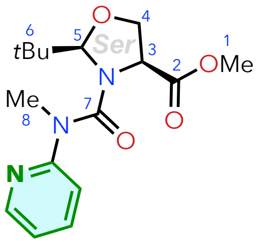

4q

Following **GP3**, *N*-chloroformyloxazolidine **3** (500-mg scale) was used as the carbamoyl chloride and *N*-methylpyridin-2-amine (0.31 mL, 3.0 mmol, 1.5 equiv.) as the aniline in CH_2Cl_2. The title compound **4q** was obtained as a beige solid (167 mg, 0.52 mmol, 26%) after purification by silica gel column chromatography (PE/EA, gradient elution).

Formula: $C_{16}H_{23}N_3O_4$, **MW:** 321.38 g/mol, **m.p.:** 58 – 62 °C. **TLC:** R_f = 0.29 (PE/EA 2:1), $KMnO_4$ stain. ^{1}**H NMR** (500 MHz, $CDCl_3$): δ [ppm] = 8.39 (ddd, *J* 5.0, 2.0, 0.9 Hz, 1H, Ar), 7.67 (td, *J* 8.1, 2.0 Hz, 1H, Ar), 7.21 (d, *J* 8.2 Hz, 1H, Ar), 7.06 (ddd, *J* 7.4, 4.9, 1.0 Hz, 1H, Ar), 5.36 (s, 1H, H-5), 4.45 (dd, *J* 6.3 Hz, 1H, H-3), 4.16 (d, *J* 8.9 Hz, 1H, H-4a), 3.89 (dd, *J* 8.9, 6.7 Hz, 1H, H-4b), 3.43 (s, 3H, H-1, OMe), 3.33 (s, 3H, H-8, NMe), 1.01 (s, 9H, *t*Bu). ^{13}**C NMR** (125 MHz, $CDCl_3$): δ [ppm] = 170.3 (s, C-2, ester), 162.5 (s, C-7, urea), 156.4 (s, Ar), 148.6 (d, Ar), 138.5 (d, Ar), 120.2 (d, Ar), 120.1 (d, Ar), 98.3 (d, C-5), 69.9 (t, C-4), 62.9 (d, C-3), 52.0 (q, C-1, OMe), 36.9 (q, C-8, NMe), 36.1 (s, C-6, *t*Bu), 26.2 (q, *t*Bu, 3Me). **FT-IR (ATR):** $\tilde{\nu}$ [cm^{-1}] = 2955 (br w), 1761 (m), 1668 (s), 1587 (m), 1472 (s), 1438 (m), 1421 (m), 1338 (vs), 1299 (s), 1197 (m), 1178 (m), 1148 (vs), 1097 (m), 1042 (m), 985 (m), 929 (m), 879 (w), 781 (m), 742 (w), 704 (w), 662 (w). **HR-MS:** (ESI) = *m*/*z* calcd. for: $C_{16}H_{24}N_3O_4$ $[M+H]^+$ 322.1767 u, found: 322.1761 u. $\mathbf{[a]_{\lambda}^{T}}$**:** (c = 13.2 g/100 mL, $CHCl_3$) = $[a]_D^{20}$: –21.21°.

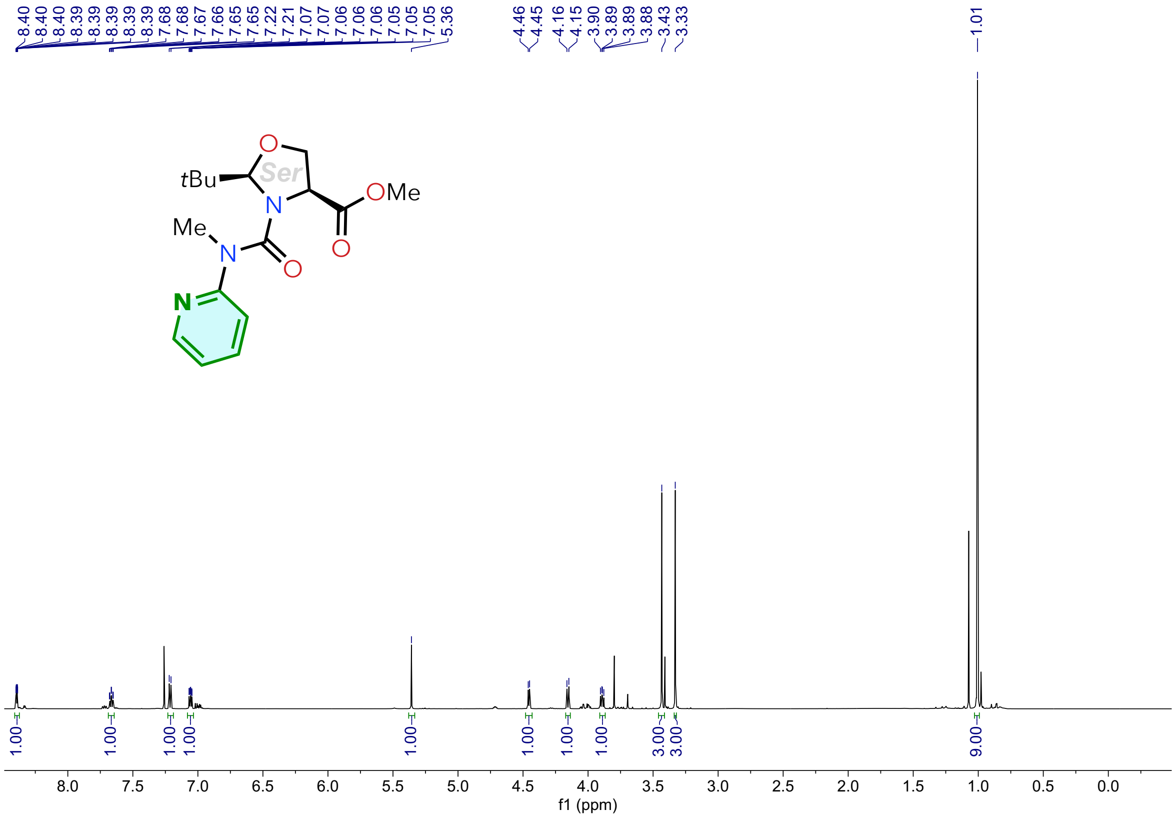
8.40
8.40
8.40
8.39
8.39
8.39
8.39
7.68
7.68
7.67
7.66
7.65
7.65
7.22
7.21
7.07
7.07
7.06
7.06
7.06
7.05
7.05
7.05
5.36
4.46
4.45
4.16
4.15
3.90
3.89
3.89
3.88
3.43
3.33
1.01
tBu
O
Ser
N
OMe
O
Me
N
O
N
1.00
1.00
1.00
1.00
1.00
1.00
1.00
1.00
3.00
3.00
9.00
8.0
7.5
7.0
6.5
6.0
5.5
5.0
4.5
4.0
3.5
3.0
2.5
2.0
1.5
1.0
0.5
0.0
f1 (ppm)

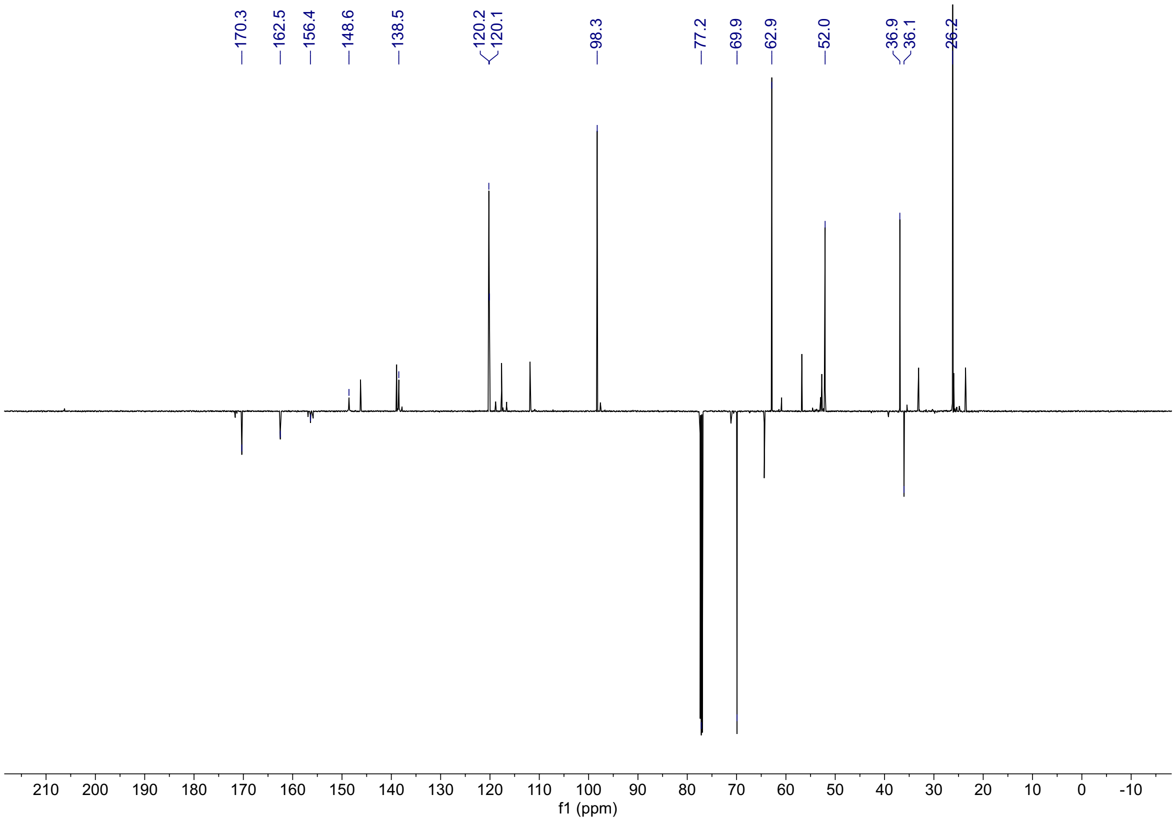
170.3
162.5
156.4
148.6
138.5
120.2
120.1
98.3
77.2
69.9
62.9
52.0
36.9
36.1
26.2
210
200
190
180
170
160
150
140
130
120
110
100
90
80
70
60
50
40
30
20
10
0
-10
f1 (ppm)

Methyl (2*R*,4*S*)-2-(*tert*-butyl)-3-(methyl(naphthalen-2-yl)carbamoyl)oxazolidine-4-carboxylate (4r)

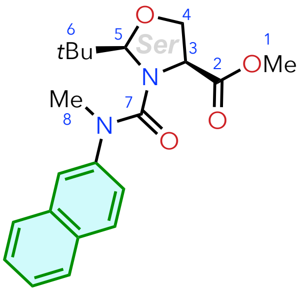

4r

Following **GP3**, *N*-chloroformyloxazolidine **3** (500-mg scale) was used as the carbamoyl chloride and *N*-methylnaphthalen-2-amine hydrochloride (581 mg, 3.0 mmol, 1.5 equiv.) as the aniline in CH_2Cl_2. A slight excess of triethylamine (0.88 mL, 6.0 mmol, 3.0 equiv.) was used in this reaction. The title compound **4r** was obtained as a dark beige solid (615 mg, 1.66 mmol, 83%) after purification by silica gel column chromatography (PE/EA, gradient elution).

Formula: $C_{21}H_{26}N_2O_4$, **MW:** 370.45 g/mol, **m.p.:** 109 – 113 °C. **TLC:** R_f = 0.45 (PE/EA 2:1), $KMnO_4$ stain. **^{1}H NMR** (500 MHz, $CDCl_3$): δ [ppm] = 7.88 (d, *J* 8.1 Hz, 2H, Ar), 7.80 (d, *J* 8.3 Hz, 1H, Ar), 7.59 (t, *J* 7.5 Hz, 1H, Ar), 7.53 (ddd, *J* 8.0, 6.9, 1.1 Hz, 1H, Ar), 7.48 (t, *J* 7.5 Hz, 1H, Ar), 7.34 (dd, *J* 7.3, 0.9 Hz, 1H, Ar), 5.40 (s, 1H, H-5), 4.15 (br s, 1H, H-3), 3.91 (br s, 1H, H-4a), 3.81 (dd, *J* 8.8, 7.0 Hz, 1H, H-4b), 3.27 (s, 3H, H-1, OMe), 2.77 (s, 3H, H-8, NMe), 0.97 (br s, 9H, *t*Bu). **^{13}C NMR** (125 MHz, $CDCl_3$): δ [ppm] = 171.6 (s, Ar), 171.3 (s, Ar), 169.3 (s, C-2, ester), 163.8 (s, C-7, urea), 134.8 (s, Ar), 128.7 (d, Ar), 128.1 (d, Ar), 127.4 (d, Ar), 127.3 (d, Ar), 126.5 (d, Ar), 126.4 (d, Ar), 98.6 (d, C-5), 70.1 (t, C-4), 62.7 (d, C-3), 51.1 (q, C-1, OMe), 39.9 (q, C-8, NMe), 36.1 (s, C-6, *t*Bu), 26.3 (q, *t*Bu, 3Me). **FT-IR (ATR):** $\tilde{\nu}$ [cm^{-1}] = 2960 (br w), 1762 (m), 1652 (s), 1423 (w), 1401 (m), 1334 (s), 1197 (m), 1180 (m), 1151 (m), 1057 (m), 976 (m), 925 (w), 872 (w), 782 (vs), 674 (w), 664 (w), 559 (w), 519 (w). **HR-MS:** (ESI) = *m*/*z* calcd. for: $C_{21}H_{27}N_2O_4$ $[M+H]^+$ 371.1971 u, found: 371.1952 u. **$[a]_\lambda^T$:** (c = 1.05 g/100 mL, $CHCl_3$) = $[a]_D^{20}$: –21.33°.

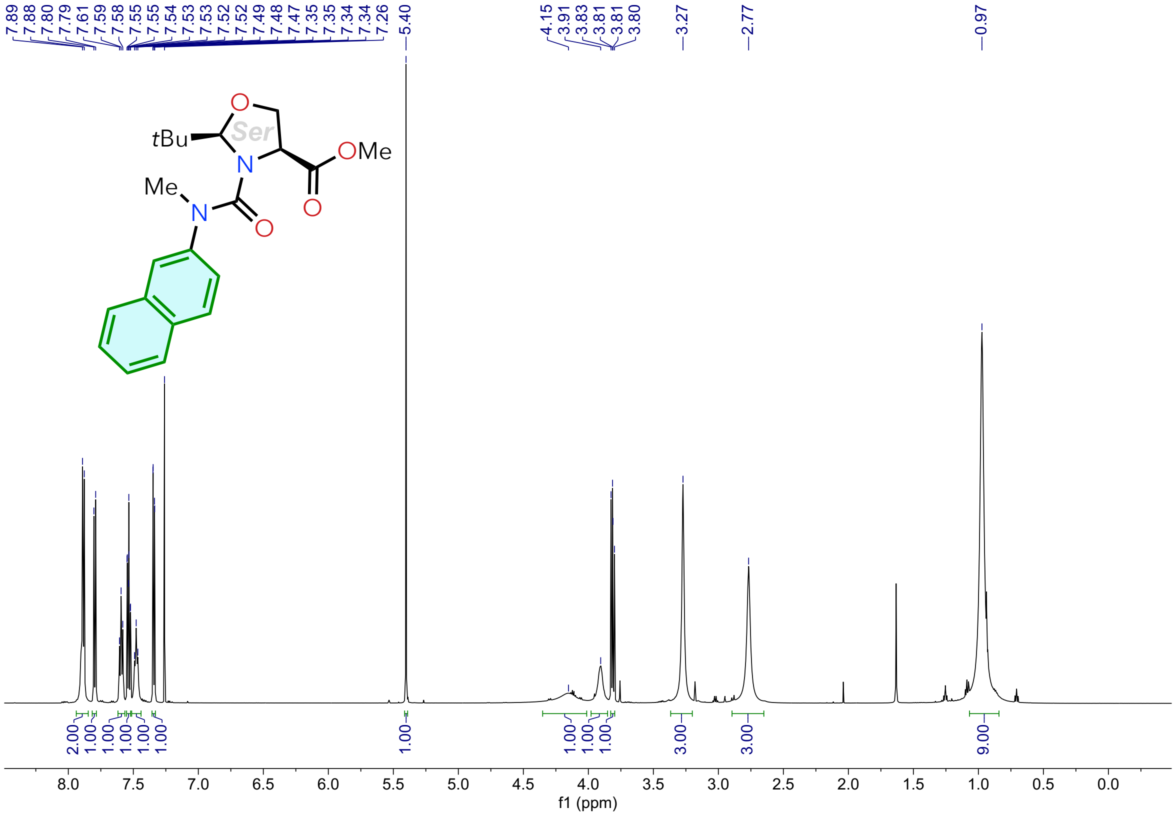
7.89
7.88
7.80
7.79
7.61
7.59
7.58
7.55
7.55
7.54
7.53
7.53
7.52
7.52
7.49
7.48
7.47
7.35
7.35
7.34
7.34
7.26
5.40
4.15
3.91
3.83
3.81
3.81
3.80
3.27
2.77
0.97
tBu
Ser
O
N
OMe
Me
N
O
O
2.00
1.00
1.00
1.00
1.00
1.00
1.00
1.00
1.00
1.00
3.00
3.00
9.00
8.0 7.5 7.0 6.5 6.0 5.5 5.0 4.5 4.0 3.5 3.0 2.5 2.0 1.5 1.0 0.5 0.0
f1 (ppm)

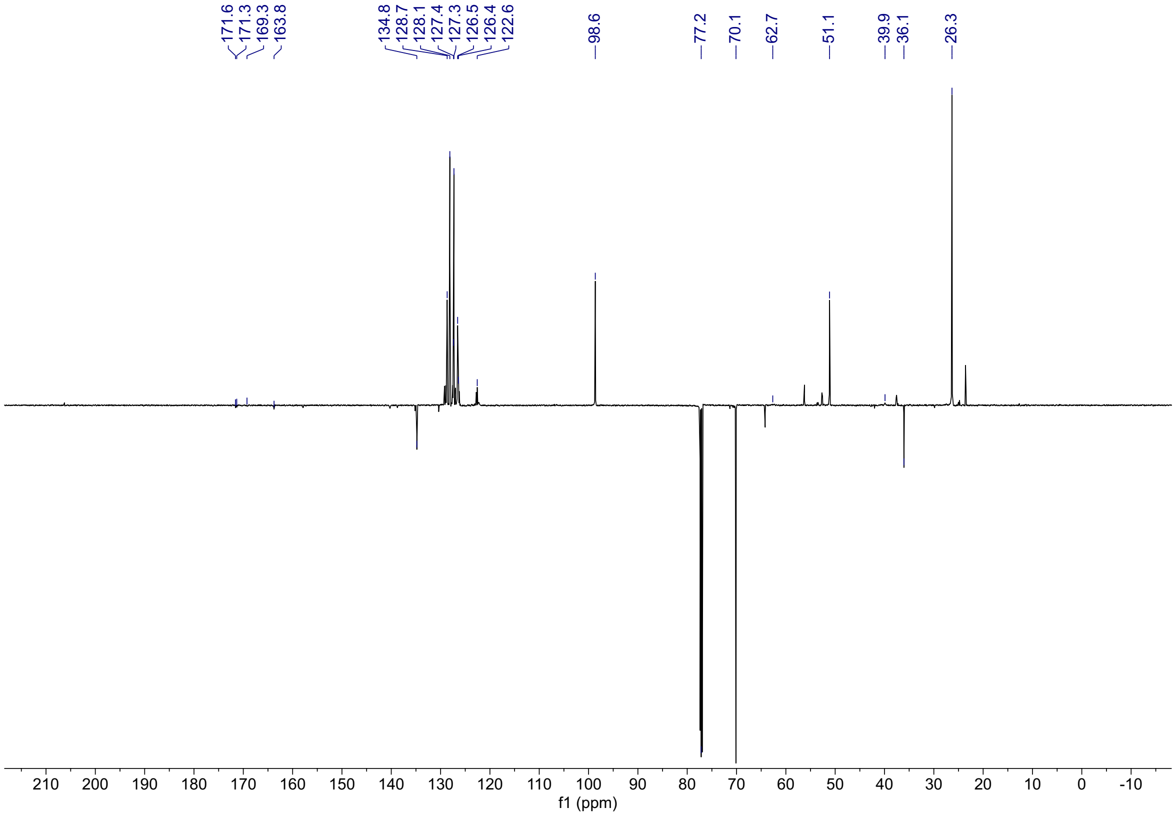
171.6
171.3
169.3
163.8
134.8
128.7
128.1
127.4
127.3
126.5
126.4
122.6
98.6
77.2
70.1
62.7
51.1
39.9
36.1
26.3
210 200 190 180 170 160 150 140 130 120 110 100 90 80 70 60 50 40 30 20 10 0 -10
f1 (ppm)

Methyl (2*R*,4*R*)-2-(*tert*-butyl)-3-(methyl(phenyl)carbamoyl)thiazolidine-4-carboxylate (19a)

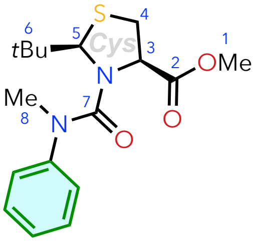

19a

Following **GP3**, to a solution of *N*-chloroformylthiozolidine **15** (500 mg, 1.88 mmol, 1.0 equiv.) in DCE (2.35 mL, 0.8 M) was added triethylamine (0.52 mL, 3.76 mmol, 2.0 equiv.) and *N*-methyl-aniline (0.31 mL, 2.82 mmol, 1.5 equiv.). The reaction mixture was heated to reflux (85 °C) for 18 h. The product **19a** was obtained as a yellow oil (659 mg, 1.96 mmol, 99%) after purification by silica gel column chromatography (PE/EA, gradient elution).

Formula: $C_{17}H_{24}N_2O_3S$, **MW:** 336.45 g/mol. **TLC:** R_f = 0.43 (PE/EA 2:1), $KMnO_4$ stain. **^{1}H NMR** (600 MHz, $CDCl_3$): δ [ppm] = 7.35 (t, *J* 7.9 Hz, 2H, Ar), 7.19 (t, *J* 7.4 Hz, 1H, Ar), 7.07 (d, *J* 9.4 Hz, 2H, Ar), 5.08 (s, 1H, H-5), 4.76 (dd, *J* 7.4, 6.0 Hz, 1H, H-3), 3.47 (s, 3H, H-1, OMe), 3.17 (s, 3H, H-8, NMe), 3.12 – 3.06 (m, 2H, H-4), 0.98 (s, 9H, *t*Bu). **^{13}C NMR** (150 MHz, $CDCl_3$): δ [ppm] = 171.0 (s, C-2, ester), 162.1 (s, C-7, urea), 145.2 (s, Ar), 129.8 (d, 2Ar), 126.0 (d, Ar), 125.6 (d, 2Ar), 74.3 (d, C-5), 67.5 (d, C-3), 52.1 (q, C-1, OMe), 40.8 (q, C-8, NMe), 38.5 (s, C-6, *t*Bu), 34.1 (s, C-4), 27.4 (q, *t*Bu, 3Me). **FT-IR (ATR):** $\tilde{\nu}$ [cm^{-1}] = 2953 (br w), 1757 (m), 1653 (vs), 1597 (m), 1495 (m), 1436 (m), 1363 (s), 1340 (s), 1297 (m), 1200 (w), 1168 (s), 1004 (w), 962 (w), 879 (w), 749 (m), 700 (m), 542 (w). **HR-MS:** (ESI) = *m/z* calcd. for: $C_{17}H_{25}N_2O_3S$ $[M+H]^+$ 337.1586 u, found: 337.1580 u. **$[a]_\lambda^T$:** (c = 1.04 g/100 mL, $CHCl_3$) = $[a]_D^{20}$: –26.15°.

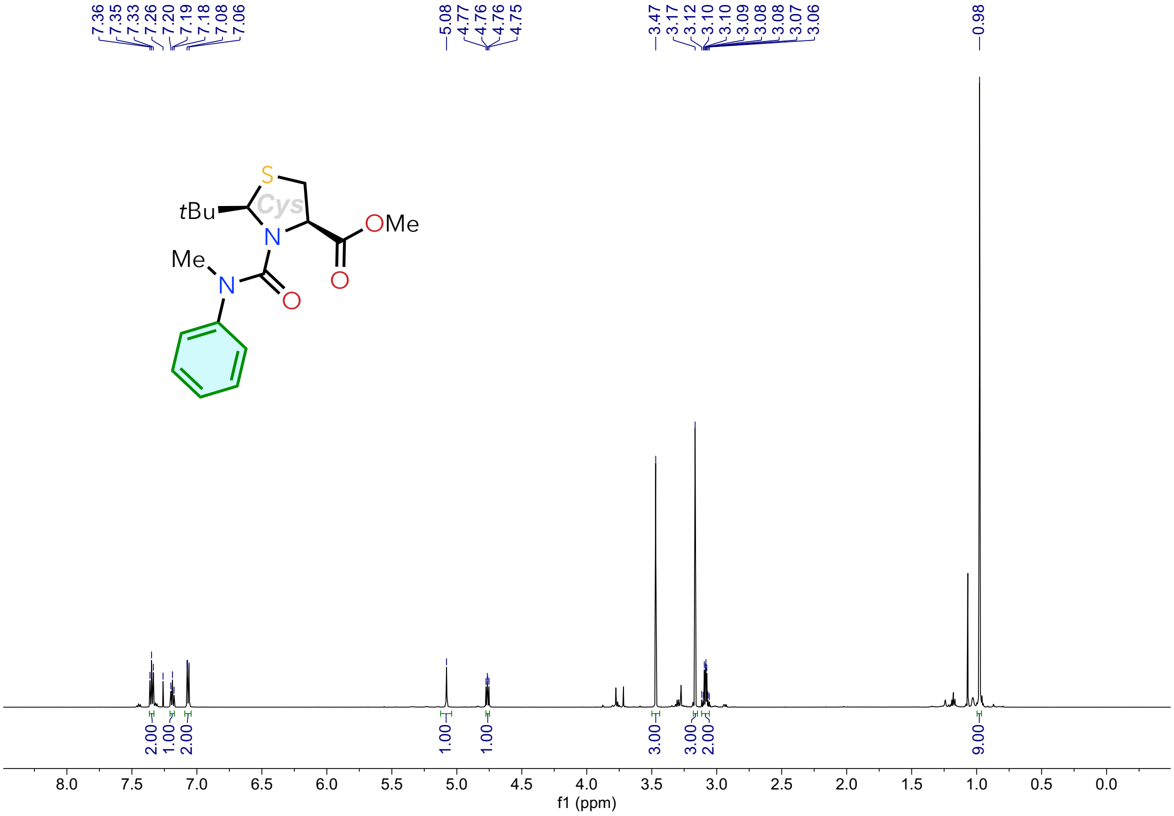
7.36
7.35
7.33
7.26
7.20
7.19
7.18
7.08
7.06
5.08
4.77
4.76
4.76
4.75
3.47
3.17
3.12
3.10
3.10
3.09
3.08
3.08
3.07
3.06
0.98
S
*t*Bu
Cys
N
OMe
O
Me
N
O
2.00
1.00
2.00
1.00
1.00
3.00
3.00
2.00
9.00
8.0 7.5 7.0 6.5 6.0 5.5 5.0 4.5 4.0 3.5 3.0 2.5 2.0 1.5 1.0 0.5 0.0
f1 (ppm)

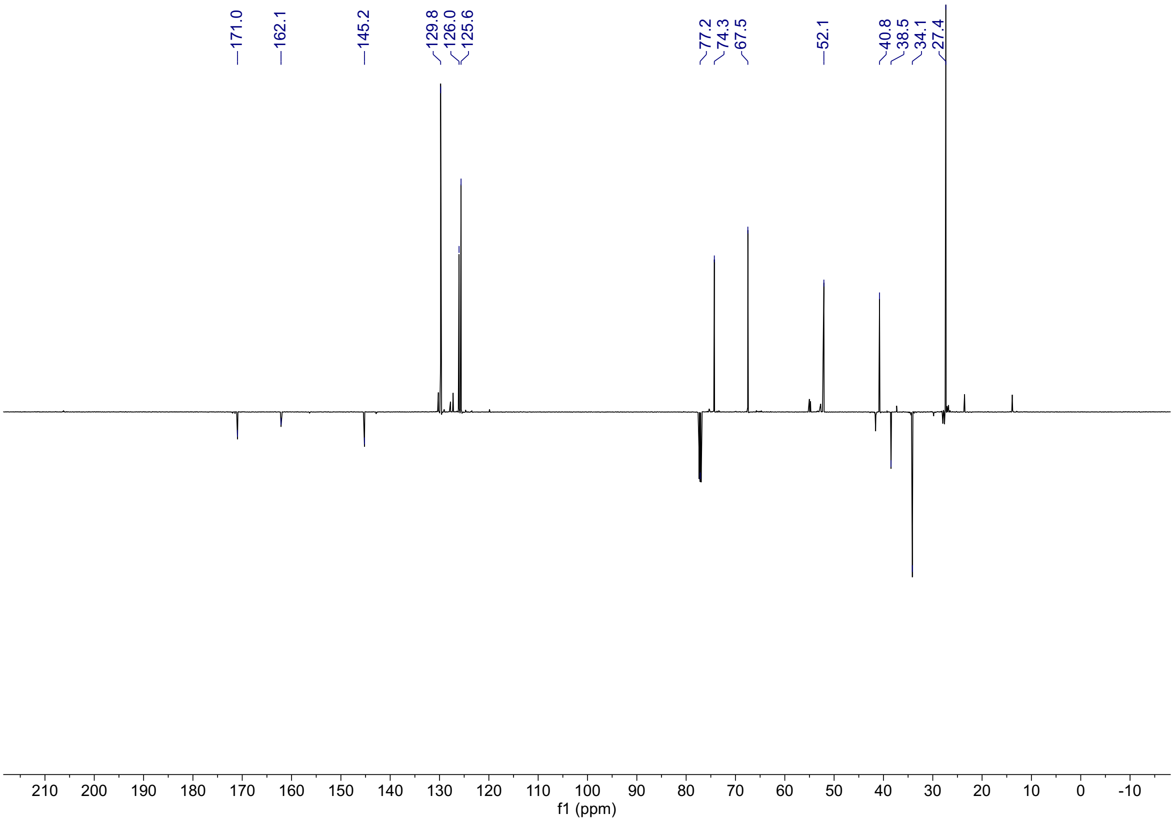
171.0
162.1
145.2
129.8
126.0
125.6
77.2
74.3
67.5
52.1
40.8
38.5
34.1
27.4
210 200 190 180 170 160 150 140 130 120 110 100 90 80 70 60 50 40 30 20 10 0 -10
f1 (ppm)

Methyl (2*R*,4*R*)-3-((4-bromophenyl)(methyl)carbamoyl)-2-(*tert*-butyl)thiazolidine-4-carboxylate (19b)

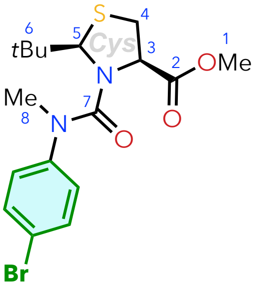

19b

Following **GP3**, *N*-chloroformylthiozolidine **15** (500-mg scale) was used as the carbamoyl chloride and 4-bromo-*N*-methylaniline (0.35 mL, 2.82 mmol, 1.5 equiv.) as the aniline in DCE. The title compound **19b** was obtained as a brown oil (646 mg, 1.56 mmol, 83%) after purification by silica gel column chromatography (PE/EA, gradient elution).

Formula: $C_{17}H_{23}BrN_2O_3S$, **MW:** 415.35 g/mol. **TLC:** R_f = 0.54 (PE/EA 2:1), $KMnO_4$ stain. **^{1}H NMR** (600 MHz, $CDCl_3$): δ [ppm] = 7.46 (d, *J* 8.6 Hz, 2H, Ar), 6.95 (d, *J* 8.6 Hz, 2H, Ar), 5.18 (s, 1H, H-5), 4.73 (t, *J* 6.5 Hz, 1H, H-3), 3.50 (s, 3H, H-1, OMe), 3.15 (s, 3H, H-8, NMe), 3.10 (d, *J* 6.5 Hz, 2H, H-4), 0.99 (s, 9H, *t*Bu). **^{13}C NMR** (150 MHz, $CDCl_3$): δ [ppm] = 170.8 (s, C-2, ester), 162.0 (s, C-7, urea), 144.3 (s, Ar), 132.9 (d, 2Ar), 127.2 (d, 2Ar), 119.3 (s, Ar), 74.6 (d, C-5), 67.4 (d, C-3), 52.5 (q, C-1, OMe), 40.6 (q, C-8, NMe), 38.4 (s, C-6, *t*Bu), 34.3 (t, C-4), 27.4 (q, *t*Bu, 3Me). **FT-IR (ATR):** $\tilde{\nu}$ [cm^{-1}] = 2952 (br w), 1758 (m), 1731 (w), 1652 (s), 1488 (m), 1434 (m), 1362 (m), 1333 (vs), 1292 (m), 1157 (s), 1010 (m), 831 (m), 795 (w), 755 (m), 715 (w), 540 (m), 453 (w). **HR-MS:** (ESI) = *m*/*z* calcd. for: $C_{17}H_{24}{}^{79}BrN_2O_3S$ $[M+H]^+$ 415.0691 u, found: 415.0686 u. **$[a]_\lambda^T$:** (c = 1.12 g/100 mL, $CHCl_3$) = $[a]_D^{20}$: –15.36°.

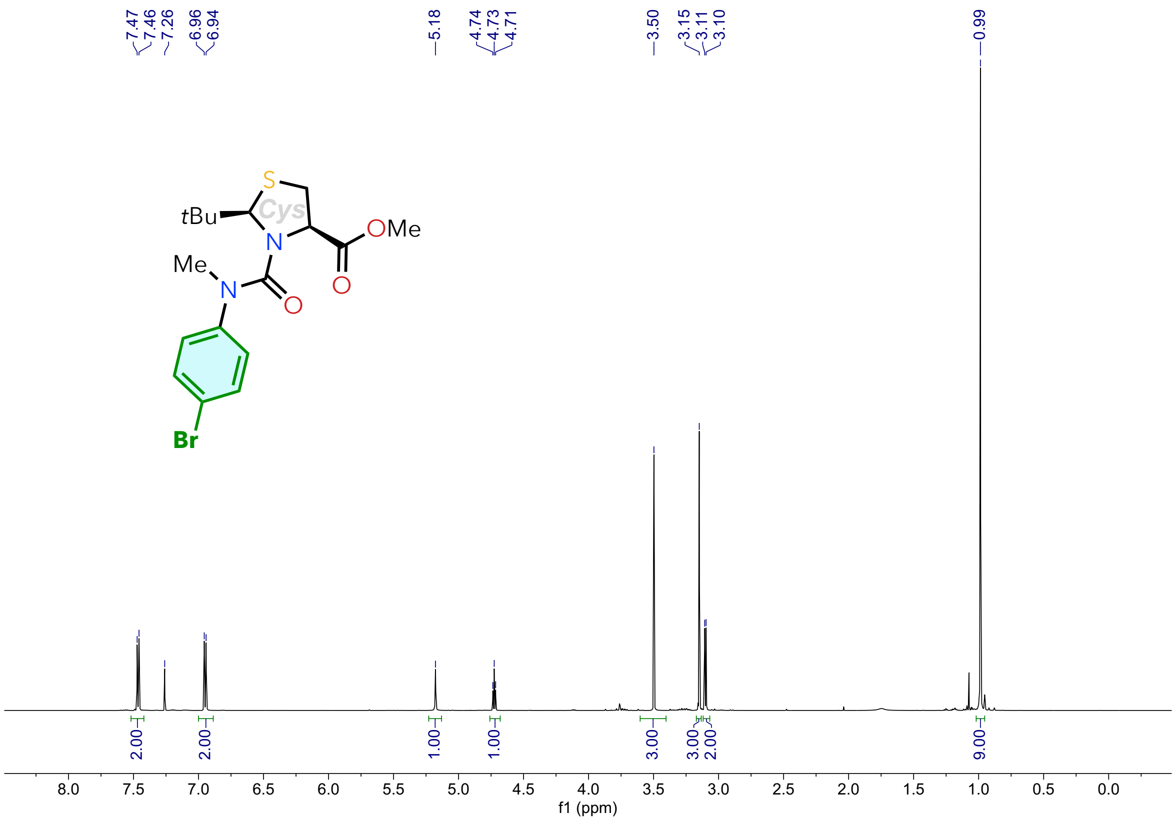

7.47
7.46
7.26
6.96
6.94
5.18
4.74
4.73
4.71
3.50
3.15
3.11
3.10
0.99
tBu
S
Cys
N
OMe
O
Me
N
O
Br
2.00
2.00
1.00
1.00
3.00
3.00
2.00
9.00
8.0
7.5
7.0
6.5
6.0
5.5
5.0
4.5
4.0
3.5
3.0
2.5
2.0
1.5
1.0
0.5
0.0
f1 (ppm)

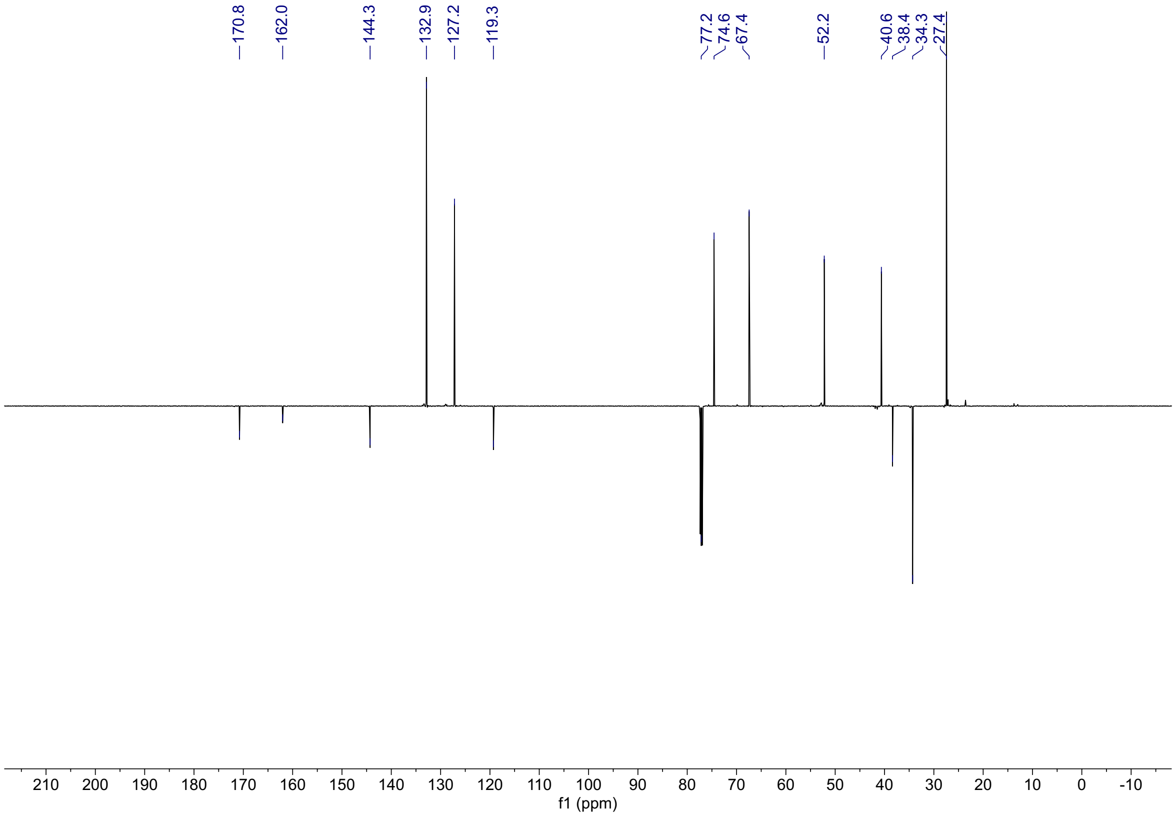

170.8
162.0
144.3
132.9
127.2
119.3
77.2
74.6
67.4
52.2
40.6
38.4
34.3
27.4
210
200
190
180
170
160
150
140
130
120
110
100
90
80
70
60
50
40
30
20
10
0
-10
f1 (ppm)

Methyl (2*R*,4*R*)-3-((3-bromophenyl)(methyl)carbamoyl)-2-(*tert*-butyl)thiazolidine-4-carboxylate (19c)

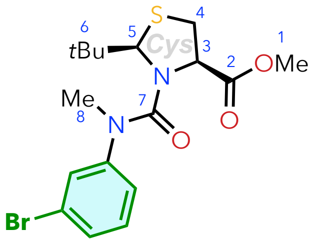

19c

Following **GP3**, *N*-chloroformylthiozolidine **15** (500-mg scale) was used as the carbamoyl chloride and 3-bromo-*N*-methylaniline (0.36 mL, 2.82 mmol, 1.5 equiv.) as the aniline in DCE. The title compound **19c** was obtained as a yellow oil (647 mg, 1.56 mmol, 83%) after purification by silica gel column chromatography (PE/EA, gradient elution).

Formula: $C_{17}H_{23}BrN_2O_3S$, **MW:** 415.35 g/mol. **TLC:** R_f = 0.42 (PE/EA 2:1), $KMnO_4$ stain. **^{1}H NMR** (600 MHz, $CDCl_3$): δ [ppm] = 7.32 (ddd, *J* 8.0, 1.9, 1.0 Hz, 1H, Ar), 7.24 – 7.21 (m, 2H, Ar), 7.00 (ddd, *J* 8.0, 2.2, 1.0 Hz, 1H, Ar), 5.15 (s, 1H, H-5), 4.74 (t, *J* 6.6 Hz, 1H, H-3), 3.53 (s, 3H, H-1, OMe), 3.16 (s, 3H, H-8, NMe), 3.12 (d, *J* 6.8 Hz, 2H, H-4), 0.99 (s, 9H, *t*Bu). **^{13}C NMR** (150 MHz, $CDCl_3$): δ [ppm] = 170.7 (s, C-2, ester), 161.9 (s, C-7, urea), 146.5 (s, Ar), 131.0 (d, Ar), 128.8 (d, Ar), 128.5 (d, Ar), 123.8 (d, Ar), 123.0 (s, Ar), 74.6 (d, C-5), 67.4 (d, C-3), 52.3 (q, C-1, OMe), 40.5 (q, C-8, NMe), 38.4 (s, C-6, *t*Bu), 34.3 (t, C-4), 27.4 (q, *t*Bu, 3Me). **FT-IR (ATR):** $\tilde{\nu}$ [cm^{-1}] = 2954 (br w), 1757 (m), 1659 (s), 1587 (m), 1566 (m), 1477 (m), 1434 (m), 1363 (m), 1333 (vs), 1299 (m), 1199 (m), 1165 (s), 999 (m), 966 (w), 881 (w), 861 (m), 780 (m), 765 (m), 695 (s). **HR-MS:** (ESI) = *m*/*z* calcd. for: $C_{17}H_{24}{}^{79}BrN_2O_3S$ $[M+H]^+$ 415.0691 u, found: 415.0676 u. **$[a]_\lambda^T$:** (c = 1.01 g/100 mL, $CHCl_3$) = $[a]_D^{20}$: –19.80°.

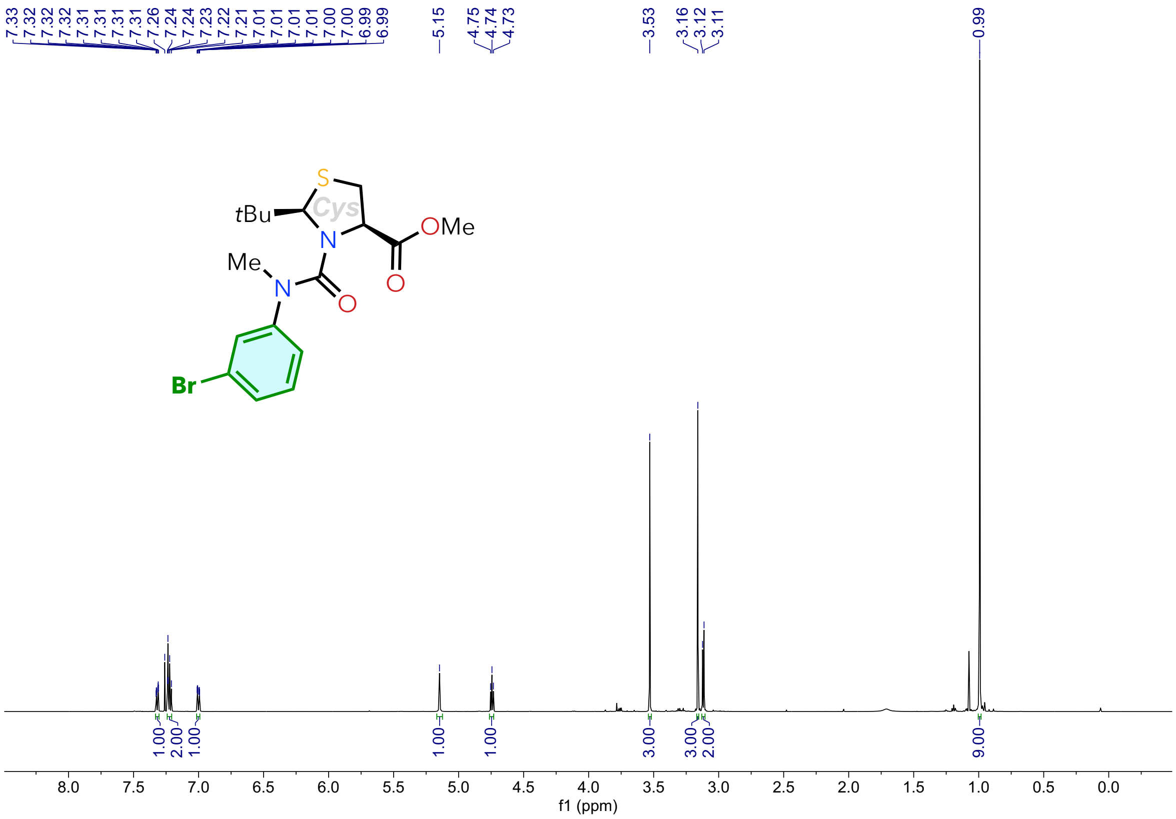
7.33 7.32 7.32 7.32 7.31 7.31 7.31 7.31 7.26 7.24 7.24 7.23 7.22 7.21 7.01 7.01 7.01 7.01 7.00 7.00 6.99 6.99
5.15
4.75 4.74 4.74 4.73
3.53
3.16 3.12 3.11
0.99
S
Cys
tBu
N
OMe
O
Me
N
O
Br
1.00 2.00 1.00
1.00
1.00
3.00
3.00 2.00
9.00
8.0 7.5 7.0 6.5 6.0 5.5 5.0 4.5 4.0 3.5 3.0 2.5 2.0 1.5 1.0 0.5 0.0
f1 (ppm)

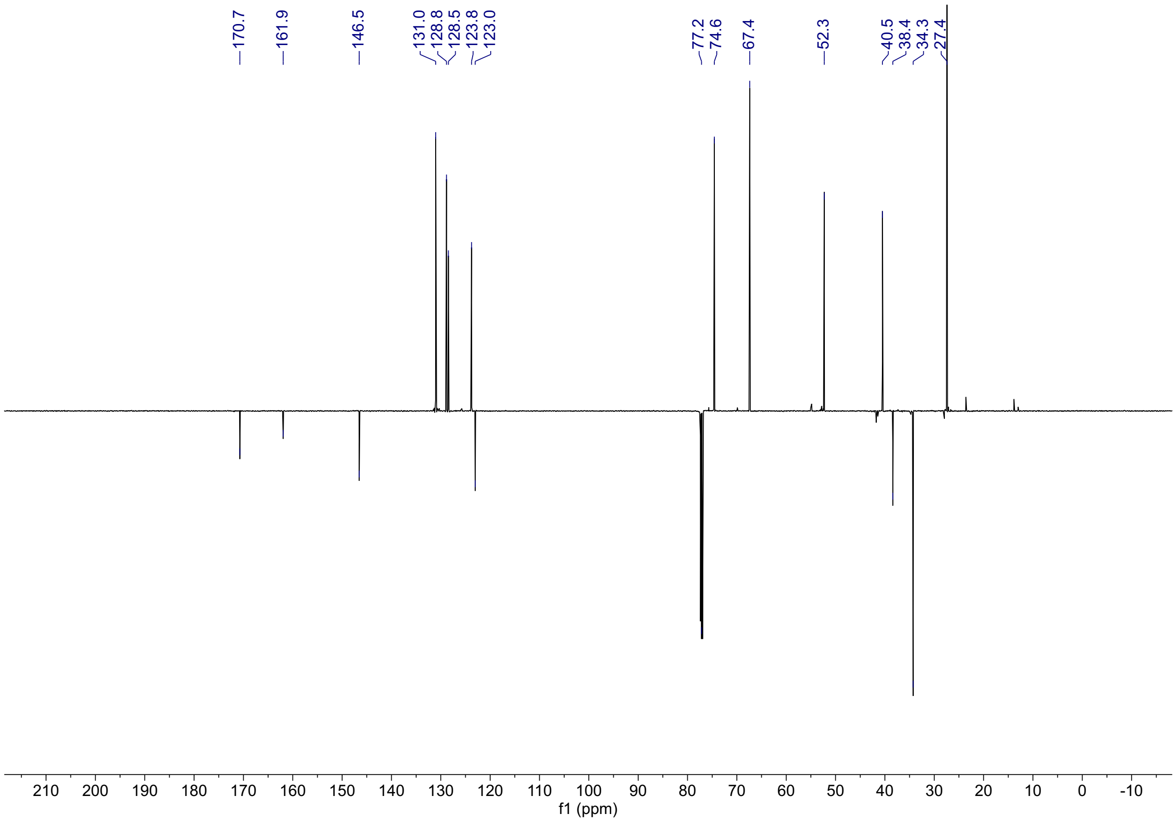
170.7
161.9
146.5
131.0 128.8 128.5 123.8 123.0
77.2 74.6
67.4
52.3
40.5 38.4 34.3 27.4
210 200 190 180 170 160 150 140 130 120 110 100 90 80 70 60 50 40 30 20 10 0 -10
f1 (ppm)

Methyl (2*R*,4*R*)-3-((2-bromophenyl)(methyl)carbamoyl)-2-(*tert*-butyl)thiazolidine-4-carboxylate (19d)

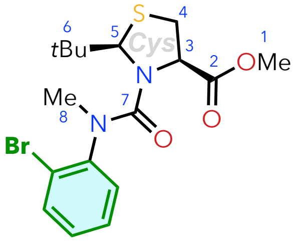

19d

Following **GP3**, *N*-chloroformylthiozolidine **15** (500-mg scale) was used as the carbamoyl chloride and 2-bromo-*N*-methylaniline (0.33 mL, 2.82 mmol, 1.5 equiv.) as the aniline in DCE. The title compound **19d** was obtained as a beige solid (420 mg, 1.01 mmol, 54%) after purification by silica gel column chromatography (PE/EA, gradient elution).

Formula: $C_{17}H_{23}BrN_2O_3S$, **MW:** 415.35 g/mol, **m.p.:** 112 – 116 °C. **TLC:** R_f = 0.53 (PE/EA 2:1), $KMnO_4$ stain. **^{1}H NMR** (600 MHz, $CDCl_3$): δ [ppm] = 7.58 (dd, *J* 8.0, 1.4 Hz, 1H, Ar), 7.37 (td, *J* 7.7, 1.5 Hz, 1H, Ar), 7.22 (dd, *J* 7.9, 1.6 Hz, 1H, Ar), 7.15 (td, *J* 7.7, 1.6 Hz, 1H, Ar), 5.58 (s, 1H, H-5), 4.85 (dd, *J* 6.9, 3.9 Hz, 1H, H-3), 3.35 (s, 3H, H-1, OMe), 3.15 – 3.09 (m, 5H, H-4, H-8, NMe), 1.00 (s, 9H, *t*Bu). **^{13}C NMR** (150 MHz, $CDCl_3$): δ [ppm] = 170.1 (s, C-2, ester), 163.1 (s, C-7, urea), 143.5 (s, Ar), 133.7 (d, Ar), 130.8 (d, Ar), 129.2 (d, Ar), 128.8 (d, Ar), 122.6 (s, Ar), 75.3 (d, C-5), 67.0 (d, C-3), 52.1 (q, C-1, OMe), 39.4 (q, C-8, NMe), 37.7 (s, C-6, *t*Bu), 34.9 (t, C-4), 27.7 (q, *t*Bu, 3Me). **FT-IR (ATR):** $\tilde{\nu}$ [cm^{-1}] = 2950 (br w), 1759 (w), 1731 (m), 1654 (vs), 1477 (m), 1433 (m), 1334 (vs), 1299 (vs), 1198 (m), 1148 (m), 1027 (m), 876 (w), 869 (w), 768 (m), 725 (m), 627 (w), 489 (w), 457 (w). **HR-MS:** (ESI) = *m*/*z* calcd. for: $C_{17}H_{23}{}^{79}BrN_2O_3SNa$ $[M+Na]^+$ 437.0510 u, found: 437.0487 u. **$[a]_\lambda^T$:** (c = 1.00 g/100 mL, $CHCl_3$) = $[a]_D^{20}$: –21.20°.

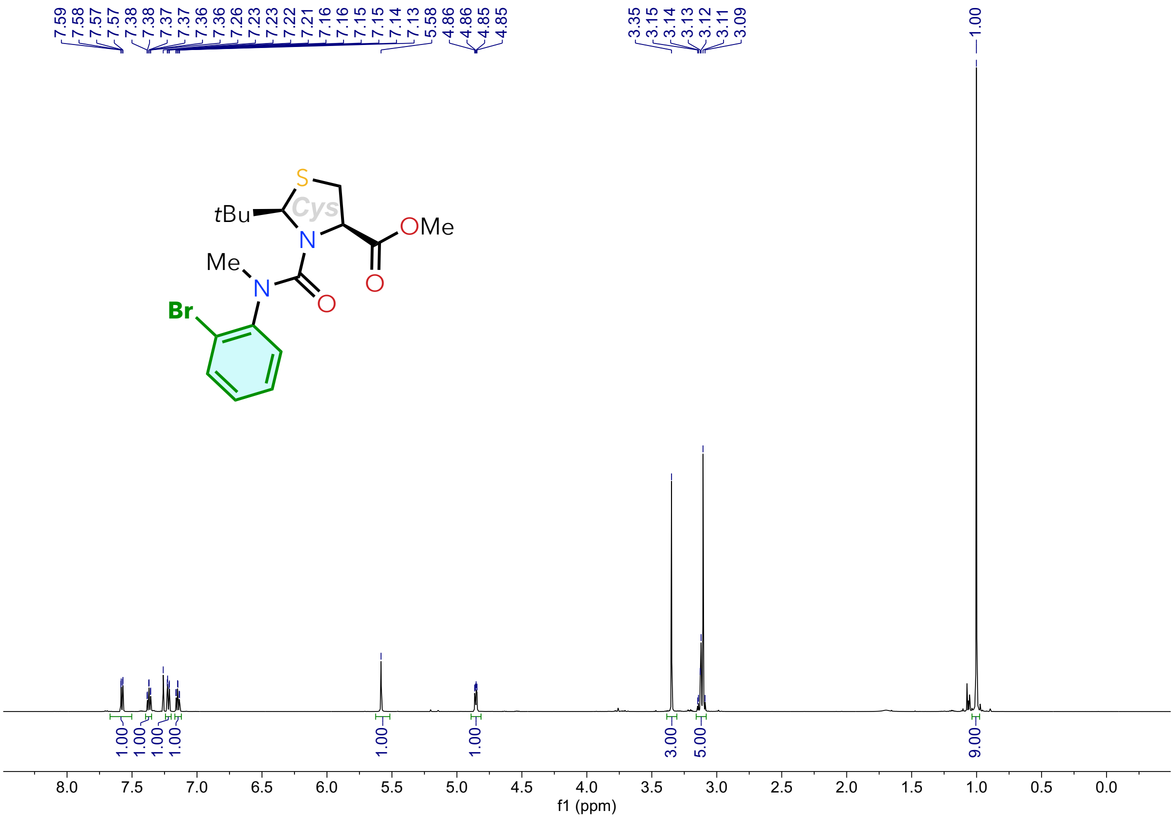
7.59
7.58
7.57
7.57
7.38
7.38
7.37
7.37
7.36
7.36
7.26
7.23
7.23
7.22
7.21
7.16
7.16
7.15
7.15
7.14
7.13
5.58
4.86
4.86
4.85
4.85
3.35
3.15
3.14
3.13
3.12
3.11
3.09
1.00
S
Cys
tBu
N
OMe
O
Me
N
O
Br
1.00
1.00
1.00
1.00
1.00
1.00
3.00
5.00
9.00
8.0
7.5
7.0
6.5
6.0
5.5
5.0
4.5
4.0
3.5
3.0
2.5
2.0
1.5
1.0
0.5
0.0
f1 (ppm)

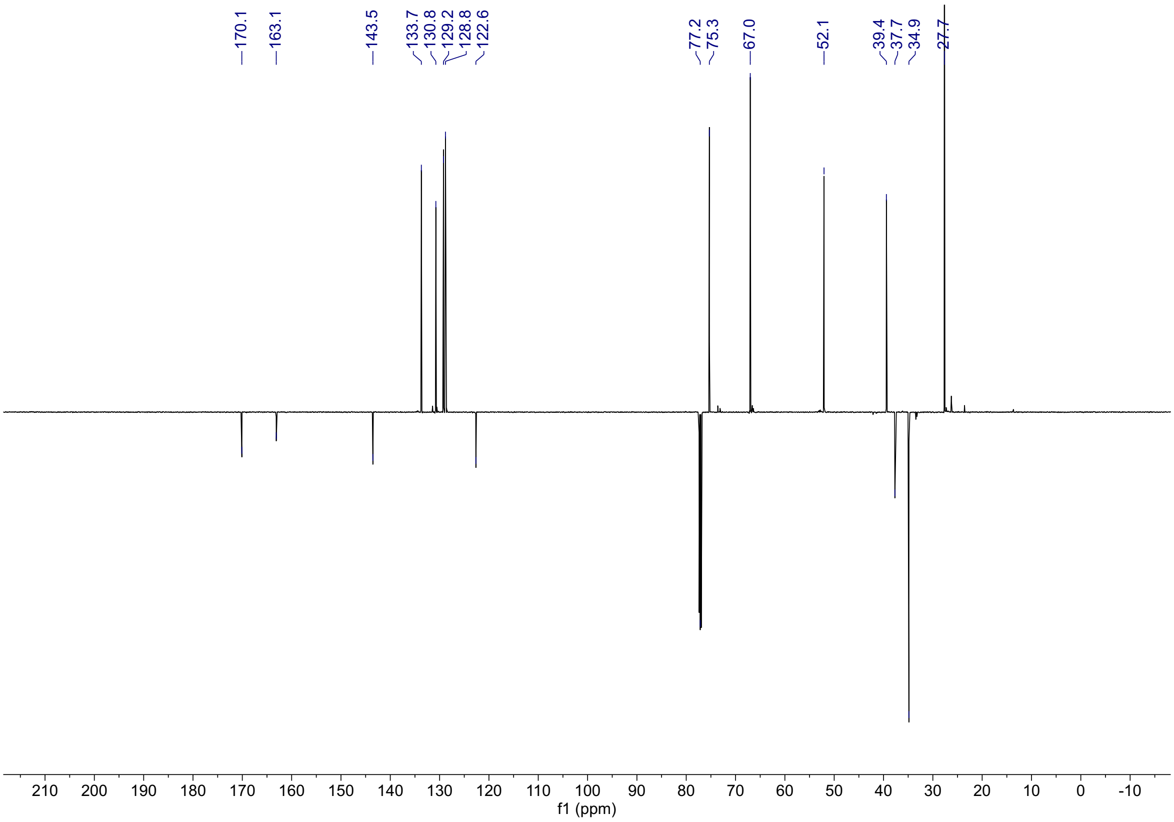
170.1
163.1
143.5
133.7
130.8
129.2
128.8
122.6
77.2
75.3
67.0
52.1
39.4
37.7
34.9
27.7
210
200
190
180
170
160
150
140
130
120
110
100
90
80
70
60
50
40
30
20
10
0
-10
f1 (ppm)

Methyl (2*R*,4*R*)-2-(*tert*-butyl)-3-(methyl(4-(trifluoromethyl)phenyl)carbamoyl)-thiazolidine-4-carboxylate (19e)

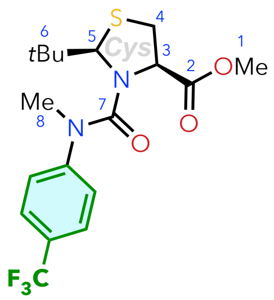

19e

Following **GP3**, *N*-chloroformylthiozolidine **15** (500-mg scale) was used as the carbamoyl chloride and *N*-methyl-4-(trifluoromethyl)aniline (0.40 mL, 2.82 mmol, 1.5 equiv.) as the aniline in DCE. The title compound **19e** was obtained as a yellow oil (452 mg, 1.12 mmol, 59%) after purification by silica gel column chromatography (PE/EA, gradient elution).

Formula: $C_{18}H_{23}F_3N_2O_3S$, **MW:** 404.45 g/mol. **TLC:** R_f = 0.51 (PE/EA 2:1), $KMnO_4$ stain. **^{1}H NMR** (500 MHz, $CDCl_3$): δ [ppm] = 7.60 (d, *J* 8.4 Hz, 2H, Ar), 7.13 (d, *J* 8.4 Hz, 2H, Ar), 5.09 (s, 1H, H-5), 4.75 (t, *J* 6.9 Hz, 1H, H-3), 3.51 (s, 3H, H-1, OMe), 3.20 (s, 3H, H-8, NMe), 3.14 (d, *J* 7.2 Hz, 2H, H-4), 1.00 (s, 9H, *t*Bu). **^{13}C NMR** (125 MHz, $CDCl_3$): δ [ppm] = 170.6 (s, C-2, ester), 161.6 (s, Ar), 161.1 (s, C-7, urea), 127.2 (s, Ar), 126.9 (d, *q J* 3.7 Hz, 2Ar), 124.1 (s, *q J* 271.2 Hz, Ar), 123.8 (d, 2Ar), 74.6 (d, C-5), 67.0 (d, C-3), 52.2 (q, C-1, OMe), 39.9 (q, C-8, NMe), 38.6 (s, C-6, *t*Bu), 34.1 (t, C-4), 27.4 (q, *t*Bu, 3Me). **^{19}F NMR** (470 MHz, $CDCl_3$, C_6F_6 ref.): δ [ppm] = –65.41 (s, 3F). **FT-IR (ATR):** $\tilde{\nu}$ [cm^{-1}] = 2956 (br w), 1757 (w), 1660 (m), 1614 (m), 1519 (w), 1436 (w), 1323 (vs), 1164 (s), 1116 (s), 1068 (s), 1015 (w), 846 (w); 747 (w). **HR-MS:** (ESI) = *m/z* calcd. for: $C_{18}H_{24}F_3N_2O_3S$ $[M+H]^+$ 405.1460 u, found: 405.1442 u. **$[a]_\lambda^T$:** (c = 1.00 g/100 mL, $CHCl_3$) = $[a]_D^{20}$: –21.60°.

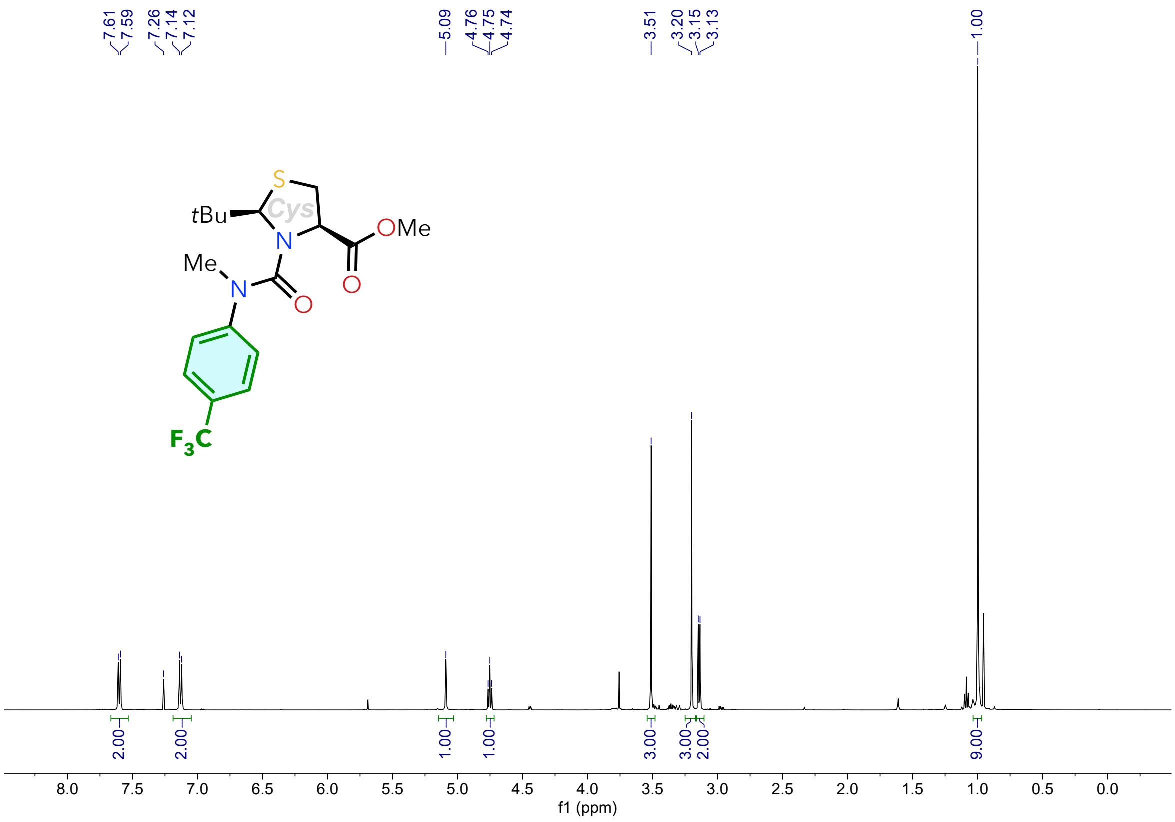
7.61
7.59
7.26
7.14
7.12
5.09
4.76
4.75
4.74
3.51
3.20
3.15
3.13
1.00
tBu
S
Cys
N
OMe
O
Me
N
O
F3C
2.00
2.00
1.00
1.00
3.00
3.00
2.00
9.00
f1 (ppm)

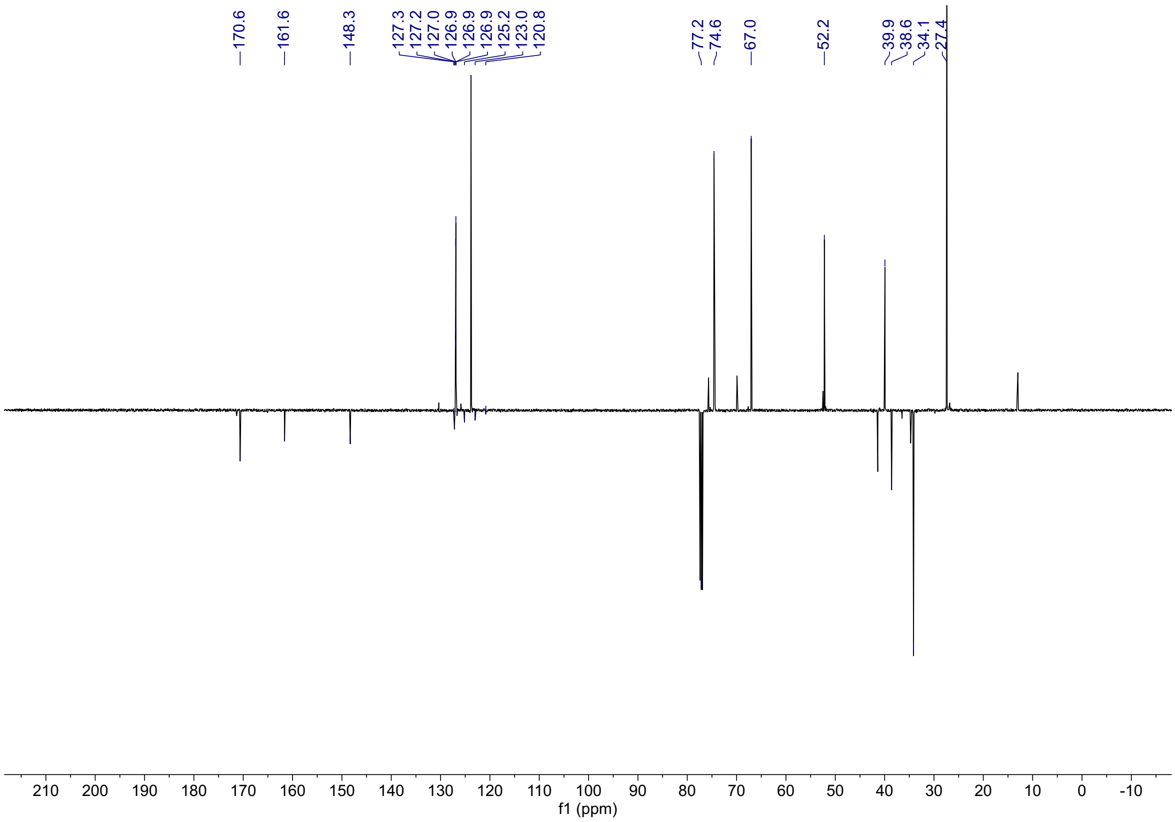
170.6
161.6
148.3
127.3
127.2
127.0
126.9
126.9
126.9
125.2
123.0
120.8
77.2
74.6
67.0
52.2
39.9
38.6
34.1
27.4
f1 (ppm)

Methyl (2*R*,4*R*)-2-(*tert*-butyl)-3-((4-cyanophenyl)(methyl)carbamoyl)thiazolidine-4-carboxylate (19f)

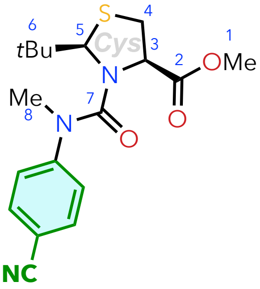

19f

Following **GP3**, *N*-chloroformylthiozolidine **15** (500-mg scale) was used as the carbamoyl chloride and 4-(methylamino)benzonitrile (373 mg, 2.82 mmol, 1.5 equiv.) as the aniline in DCE. The title compound **19f** was obtained as a yellow oil (133 mg, 0.37 mmol, 20%) after purification by silica gel column chromatography (PE/EA, gradient elution).

Formula: $C_{18}H_{23}N_3O_3S$, **MW:** 361.46 g/mol. **TLC:** R_f = 0.33 (PE/EA 2:1), $KMnO_4$ stain. **^{1}H NMR** (500 MHz, $CDCl_3$): δ [ppm] = 7.61 (d, *J* 8.8 Hz, 2H, Ar), 7.01 (d, *J* 8.7 Hz, 2H, Ar), 4.84 (s, 1H, H-5), 4.74 (t, *J* 7.7 Hz, 1H, H-3), 3.61 (s, 3H, H-1, OMe), 3.21 (d, *J* 1.3 Hz, 1H, H-4a), 2.30 (s, 4H, H-4b, H-8, NMe), 0.99 (s, 9H, *t*Bu). **^{13}C NMR** (125 MHz, $CDCl_3$): δ [ppm] = 170.6 (s, C-2, ester), 160.6 (s, C-7, urea), 149.0 (s, Ar), 133.8 (d, 2Ar), 121.2 (d, 2Ar), 118.8 (s, Ar), 107.0 (s, Ar), 74.4 (d, C-5), 66.4 (d, C-3), 52.5 (q, C-1, OMe), 39.0 (s, C-6, *t*Bu), 38.8 (q, C-8, NMe), 33.8 (t, C-4), 27.2 (q, *t*Bu, 3Me). **FT-IR (ATR):** $\tilde{\nu}$ [cm^{-1}] = 2955 (br w), 2224 (w), 1752 (m), 1662 (s), 1604 (s), 1508 (m), 1435 (m), 1336 (vs), 1173 (s), 1016 (w), 834 (w), 734 (w), 546 (w). **HR-MS:** (ESI) = *m*/*z* calcd. for: $C_{18}H_{24}N_3O_3S$ $[M+H]^+$ 362.1538 u, found: 362.1522 u. **$[a]_{\lambda}^{T}$:** (c = 1.00 g/100 mL, $CHCl_3$) = $[a]_{D}^{20}$: –24.80°.

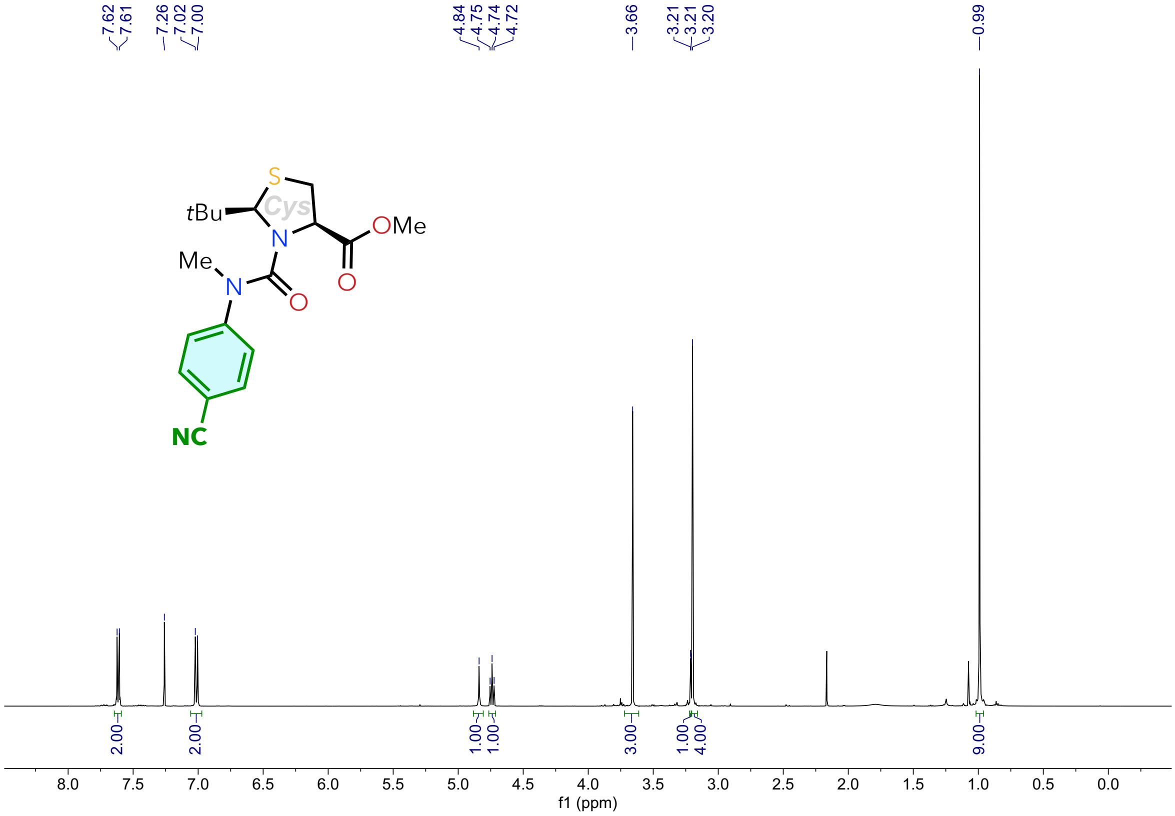
7.62
7.61
7.26
7.02
7.00
4.84
4.75
4.74
4.72
3.66
3.21
3.21
3.20
0.99
S
tBu
Cys
N
OMe
O
Me
N
O
NC
2.00
2.00
1.00
1.00
3.00
1.00
4.00
9.00
8.0 7.5 7.0 6.5 6.0 5.5 5.0 4.5 4.0 3.5 3.0 2.5 2.0 1.5 1.0 0.5 0.0
f1 (ppm)

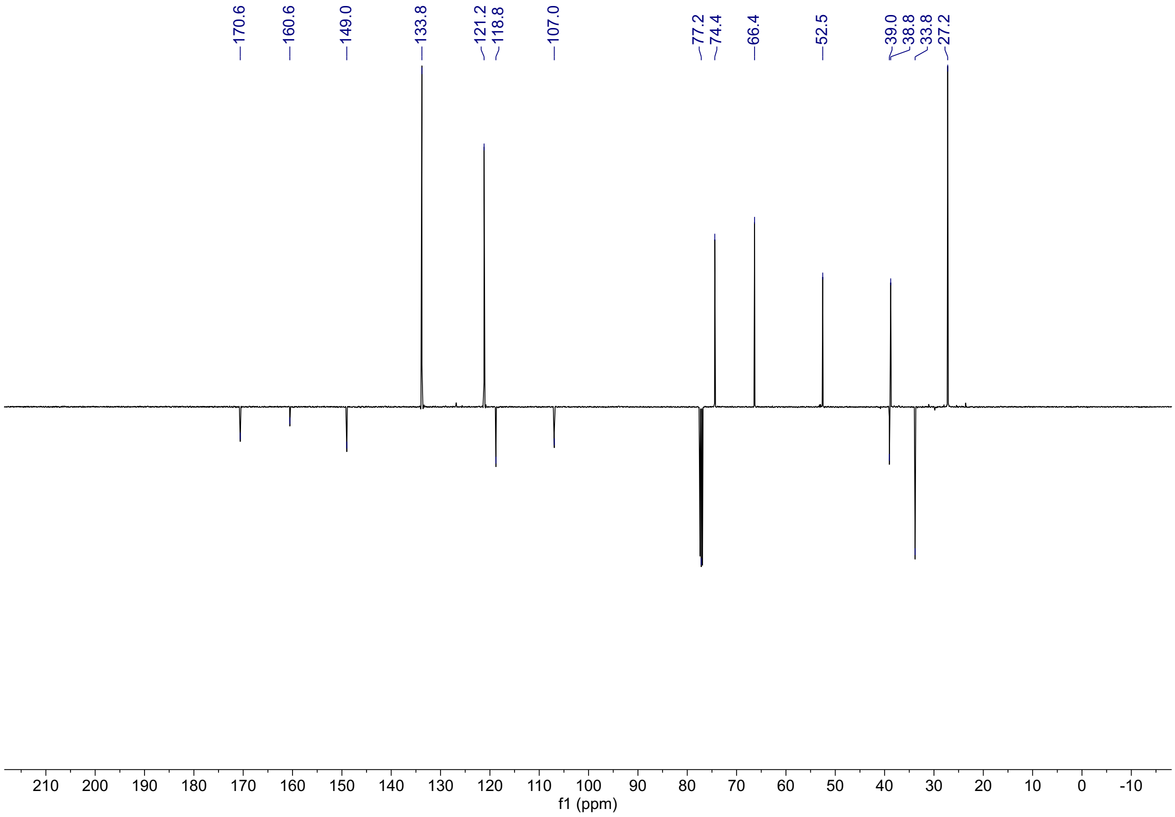
170.6
160.6
149.0
133.8
121.2
118.8
107.0
77.2
74.4
66.4
52.5
39.0
38.8
33.8
27.2
210 200 190 180 170 160 150 140 130 120 110 100 90 80 70 60 50 40 30 20 10 0 -10
f1 (ppm)

Methyl (2*R*,4*R*)-2-(*tert*-butyl)-3-((3-cyanophenyl)(methyl)carbamoyl)thiazolidine-4-carboxylate (19g)

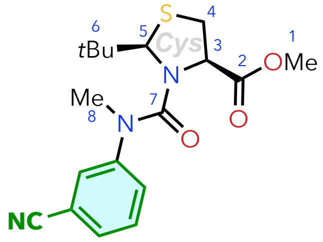

19g

Following **GP3**, *N*-chloroformylthiozolidine **15** (500-mg scale) was used as the carbamoyl chloride and 3-(methylamino)benzonitrile (373 mg, 2.82 mmol, 1.5 equiv.) as the aniline in DCE. The title compound **19g** was obtained as a white solid (403 mg, 1.11 mmol, 59%) after purification by silica gel column chromatography (PE/EA, gradient elution).

Formula: $C_{18}H_{23}N_3O_3S$, **MW:** 361.46 g/mol, **m.p.:** 124 – 127 °C. **TLC:** R_f = 0.40 (PE/EA 2:1), $KMnO_4$ stain. **^{1}H NMR** (600 MHz, $CDCl_3$): δ [ppm] = 7.46 – 7.44 (m, 2H, Ar), 7.33 (m, 1H, Ar), 7.28 (dt, *J* 7.2, 2.2 Hz, 1H, Ar), 5.09 (s, 1H, H-5), 4.73 (t, *J* 6.7 Hz, 1H, H-3), 3.56 (s, 3H, H-1, OMe), 3.19 (s, 3H, H-8, NMe), 3.15 (d, *J* 6.7 Hz, 2H, H-4), 0.99 (s, 9H, *t*Bu). **^{13}C NMR** (150 MHz, $CDCl_3$): δ [ppm] = 170.4 (s, C-2, ester), 161.6 (s, C-7, urea), 146.1 (s, Ar), 130.7 (d, Ar), 128.7 (d, Ar), 128.5 (d, Ar), 127.3 (d, Ar), 118.1 (s, Ar), 113.8 (s, Ar), 74.6 (d, C-5), 67.1 (d, C-3), 52.4 (q, C-1, OMe), 40.0 (q, C-8, NMe), 38.5 (s, C-6, *t*Bu), 34.2 (t, C-4), 27.4 (q, *t*Bu, 3Me). **FT-IR (ATR):** $\tilde{\nu}$ [cm^{-1}] = 2960 (br w), 2227 (w), 1757 (m), 1656 (s), 1597 (w), 1478 (m), 1428 (m), 1361 (m), 1322 (vs), 1208 (m), 1186 (m), 1164 (m), 1141 (m), 1015 (w), 929 (w), 879 (w), 806 (m), 691 (s), 767 (w), 735 (w). **HR-MS:** (ESI) = *m*/*z* calcd. for: $C_{18}H_{24}N_3O_3S$ $[M+H]^+$ 362.1538 u, found: 362.1529 u. **$[a]_\lambda^T$:** (c = 1.00 g/100 mL, $CHCl_3$) = $[a]_D^{20}$: –23.60°.

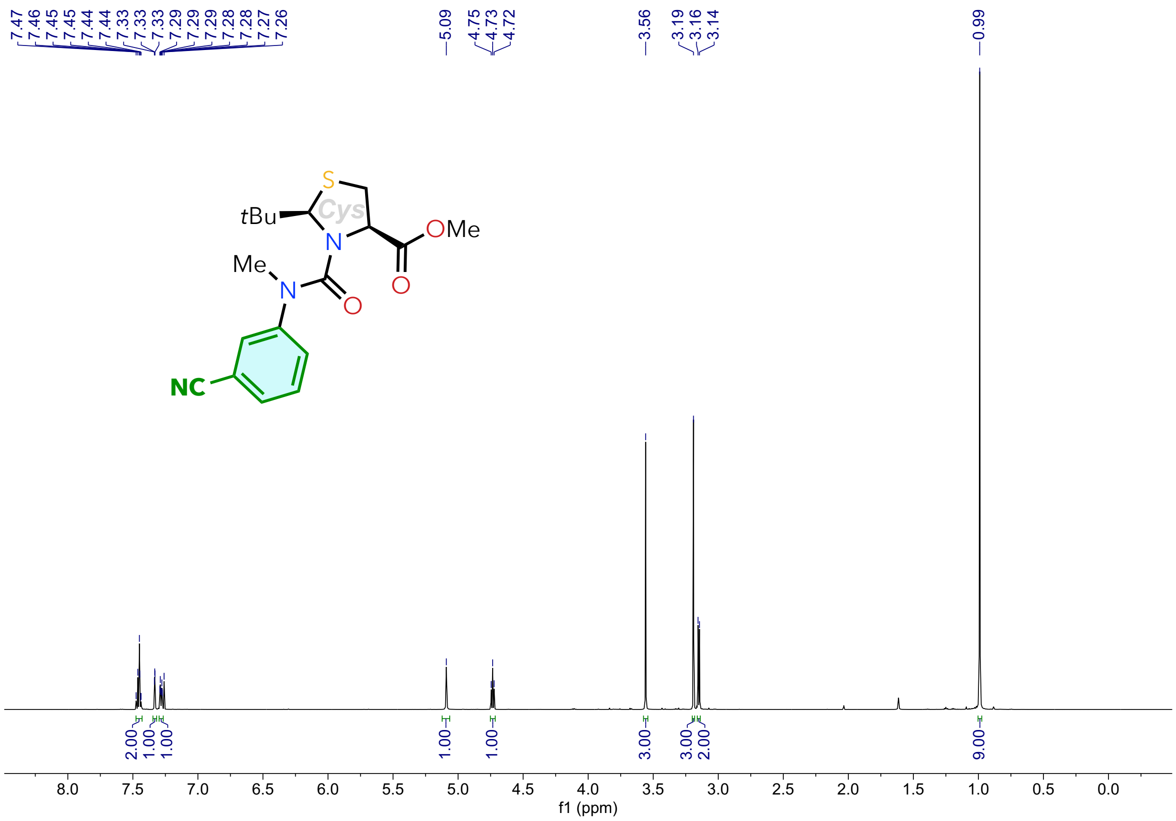
7.47
7.46
7.45
7.45
7.44
7.44
7.33
7.33
7.33
7.29
7.29
7.29
7.28
7.28
7.27
7.26
5.09
4.75
4.73
4.73
4.72
3.56
3.19
3.16
3.14
0.99
S
tBu
Cys
N
OMe
O
Me
N
O
NC
2.00
1.00
1.00
1.00
1.00
3.00
3.00
2.00
9.00
f1 (ppm)

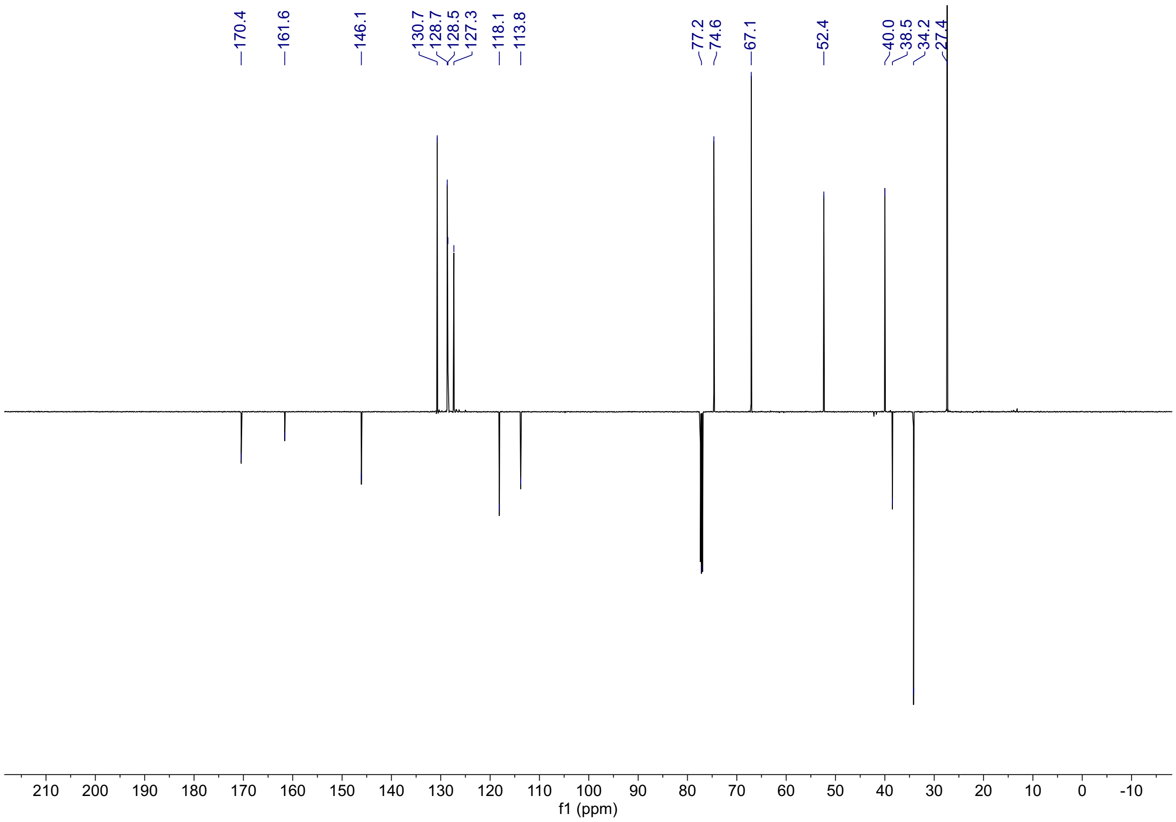
170.4
161.6
146.1
130.7
128.7
128.5
127.3
118.1
113.8
77.2
74.6
67.1
52.4
40.0
38.5
34.2
27.4
f1 (ppm)

Methyl (2*R*,4*R*)-2-(*tert*-butyl)-3-((3-ethynylphenyl)(methyl)carbamoyl)thiazolidine-4-carboxylate (19h)

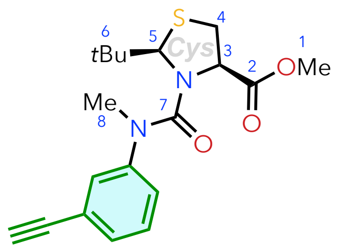

19h

Following **GP3**, *N*-chloroformylthiozolidine **15** (500-mg scale) was used as the carbamoyl chloride and 3-ethynyl-*N*-methylaniline (0.37 mL, 2.82 mmol, 1.5 equiv.) as the aniline in DCE. The title compound **19h** was obtained as a yellow oil (604 mg, 1.68 mmol, 89%) after purification by silica gel column chromatography (PE/EA, gradient elution).

Formula: $C_{19}H_{24}N_2O_3S$, **MW:** 360.47 g/mol. **TLC:** R_f = 0.44 (PE/EA 2:1), $KMnO_4$ stain. **^{1}H NMR** (600 MHz, $CDCl_3$): δ [ppm] = 7.32 – 7.31 (m, 2H, Ar), 7.21 (m, 1H, Ar), 7.07 – 7.05 (m, 1H, Ar), 5.18 (s, 1H, H-5), 4.75 (t, *J* 6.2 Hz, 1H, H-5), 3.50 (s, 3H, H-1, OMe), 3.16 (s, 3H, H-8, NMe), 3.11 (dd, *J* 4.9, 2.3 Hz, 3H, Ar, ≡CH, H-4), 0.99 (s, 9H, *t*Bu). **^{13}C NMR** (150 MHz, $CDCl_3$): δ [ppm] = 170.7 (s, C-2, ester), 162.1 (s, C-7, urea), 145.3 (s, Ar), 129.9 (d, Ar), 129.6 (d, Ar), 129.1 (d, Ar), 126.1 (d, Ar), 123.7 (s, Ar), 82.7 (s, Ar, C≡), 78.2 (s, Ar, ≡CH), 74.6 (d, C-5), 67.5 (d, C-3), 52.2 (q, C-1, OMe), 40.6 (q, C-8, NMe), 38.4 (s, C-6, *t*Bu), 34.3 (t, C-4), 27.4 (q, *t*Bu, 3Me). **FT-IR (ATR):** $\tilde{\nu}$ [cm^{-1}] = 3262 (br w), 2953 (br w), 1757 (m), 1732 (m), 1655 (vs), 1596 (m), 1576 (m), 1481 (m), 1435 (m), 1336 (vs), 1155 (s), 1017 (w), 793 (w), 695 (m), 649 (m). **HR-MS:** (ESI) = *m*/*z* calcd. for: $C_{19}H_{25}N_2O_3S$ $[M+H]^+$ 361.1586 u, found: 361.1573 u. **$[a]_\lambda^T$:** (c = 1.00 g/100 mL, $CHCl_3$) = $[a]_D^{20}$: –22.31°.

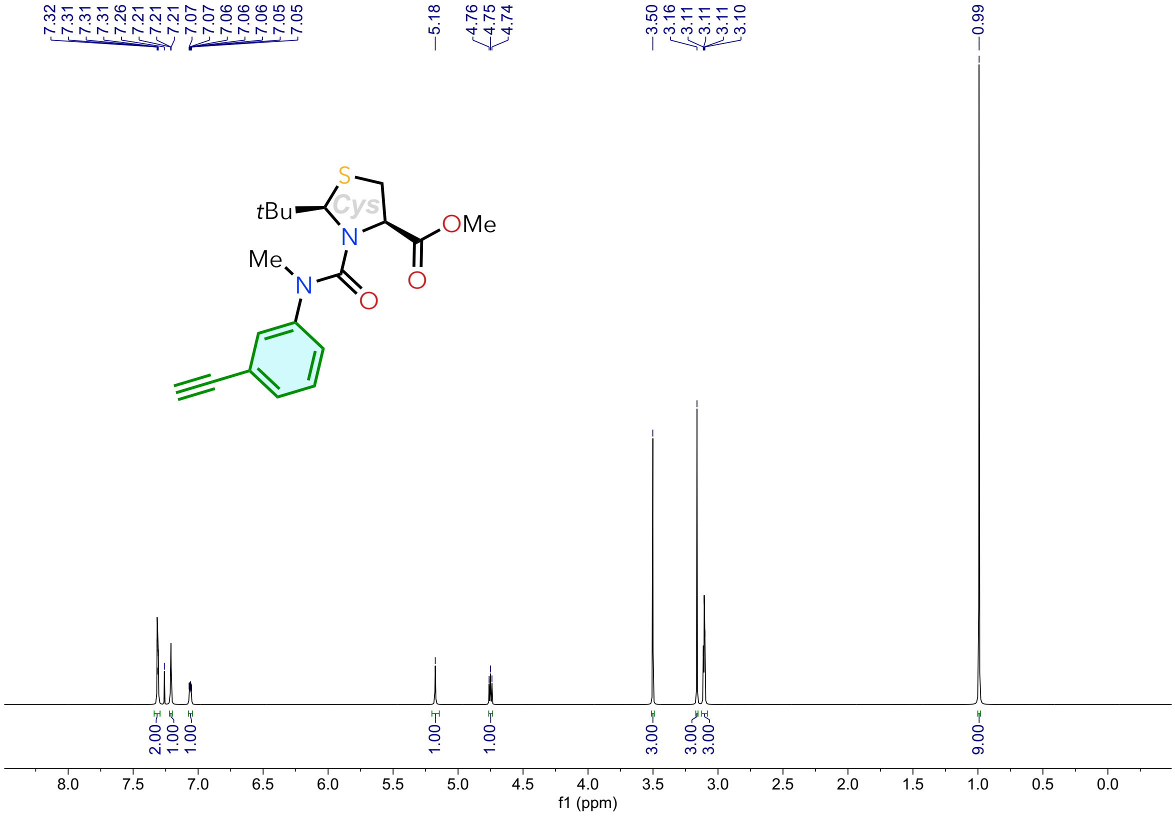
7.32 7.31 7.31 7.31 7.26 7.21 7.21 7.21 7.07 7.07 7.06 7.06 7.06 7.05 7.05
5.18
4.76 4.75 4.74
3.50 3.16 3.11 3.11 3.11 3.10
0.99
S
Cys
tBu
N
OMe
O
Me
N
O
2.00 1.00 1.00 1.00 1.00 3.00 3.00 3.00 9.00
8.0 7.5 7.0 6.5 6.0 5.5 5.0 4.5 4.0 3.5 3.0 2.5 2.0 1.5 1.0 0.5 0.0
f1 (ppm)

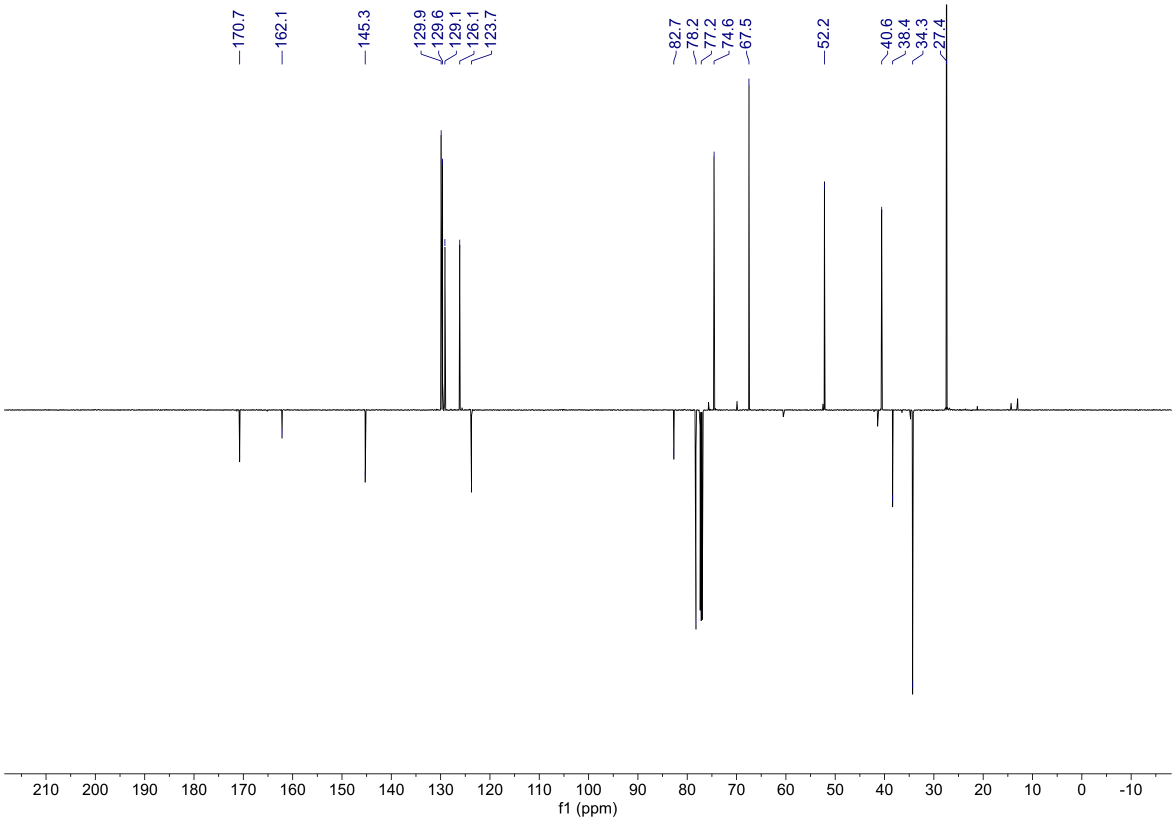
170.7
162.1
145.3
129.9 129.6 129.1 126.1 123.7
82.7 78.2 77.2 74.6 67.5
52.2
40.6 38.4 34.3 27.4
210 200 190 180 170 160 150 140 130 120 110 100 90 80 70 60 50 40 30 20 10 0 -10
f1 (ppm)

Methyl (2*R*,4*R*)-2-(*tert*-butyl)-3-(methyl(3-nitrophenyl)carbamoyl)thiazolidine-4-carboxylate (19i)

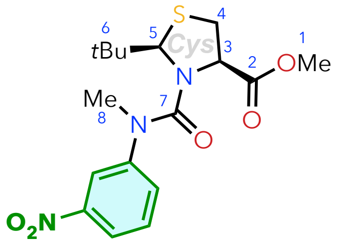

19i

Following **GP3**, *N*-chloroformylthiozolidine **15** (500-mg scale) was used as the carbamoyl chloride and 3-nitro-*N*-methylaniline (429 mg, 2.82 mmol, 1.5 equiv.) as the aniline in DCE. The title compound **19i** was obtained as a yellow oil (374 mg, 0.98 mmol, 52%) after purification by silica gel column chromatography (PE/EA, gradient elution).

Formula: $C_{17}H_{23}N_3O_5S$, **MW:** 381.44 g/mol, **m.p.:** 105 –109 °C. **TLC:** R_f = 0.44 (PE/EA 2:1), $KMnO_4$ stain. **^{1}H NMR** (500 MHz, $CDCl_3$): δ [ppm] = 8.01 (ddd, *J* 8.2, 2.2, 0.9 Hz, 1H, Ar), 7.93 (t, *J* 2.2 Hz, 1H, Ar), 7.52 (t, *J* 8.1 Hz, 1H, Ar), 7.37 (ddd, *J* 8.1, 2.3, 0.9 Hz, 1H, Ar), 5.15 (s, 1H, H-5), 4.77 (t, *J* 6.6 Hz, 1H, H-3), 3.52 (s, 3H, H-1, OMe), 3.24 (s, 3H, H-8, NMe), 3.16 (d, *J* 6.7 Hz, 1H, H-4), 1.00 (s, 9H, *t*Bu). **^{13}C NMR** (125 MHz, $CDCl_3$): δ [ppm] = 170.4 (s, C-2, ester), 161.7 (s, C-7, urea), 149.1 (s, Ar), 146.4 (s, Ar), 130.5 (d, Ar), 129.7 (d, Ar), 119.8 (d, Ar), 118.8 (d, Ar), 74.7 (d, C-5), 67.1 (d, C-3), 52.3 (q, C-1, OMe), 39.9 (q, C-8, NMe), 38.4 (s, C-6, *t*Bu), 34.2 (t, C-4), 26.3 (q, *t*Bu, 3Me). **FT-IR (ATR):** $\tilde{\nu}$ [cm^{-1}] = 2957 (br w), 1755 (m), 1658 (m), 1525 (vs), 1479 (w), 1428 (m), 1321 (vs), 1258 (m), 1207 (m), 1170 (m), 1141 (m), 1033 (s), 1014 (m), 529 (w), 876 (m), 791 (m), 729 (w), 686 (s), 486 (w), 404 (w). **HR-MS:** (ESI) = *m*/*z* calcd. for: $C_{17}H_{24}N_3O_5S$ [M+H]$^+$ 382.1437 u, found: 382.1424 u. **$[a]_\lambda^T$:** (c = 1.02 g/100 mL, $CHCl_3$) = $[a]_D^{20}$: –20.00°.

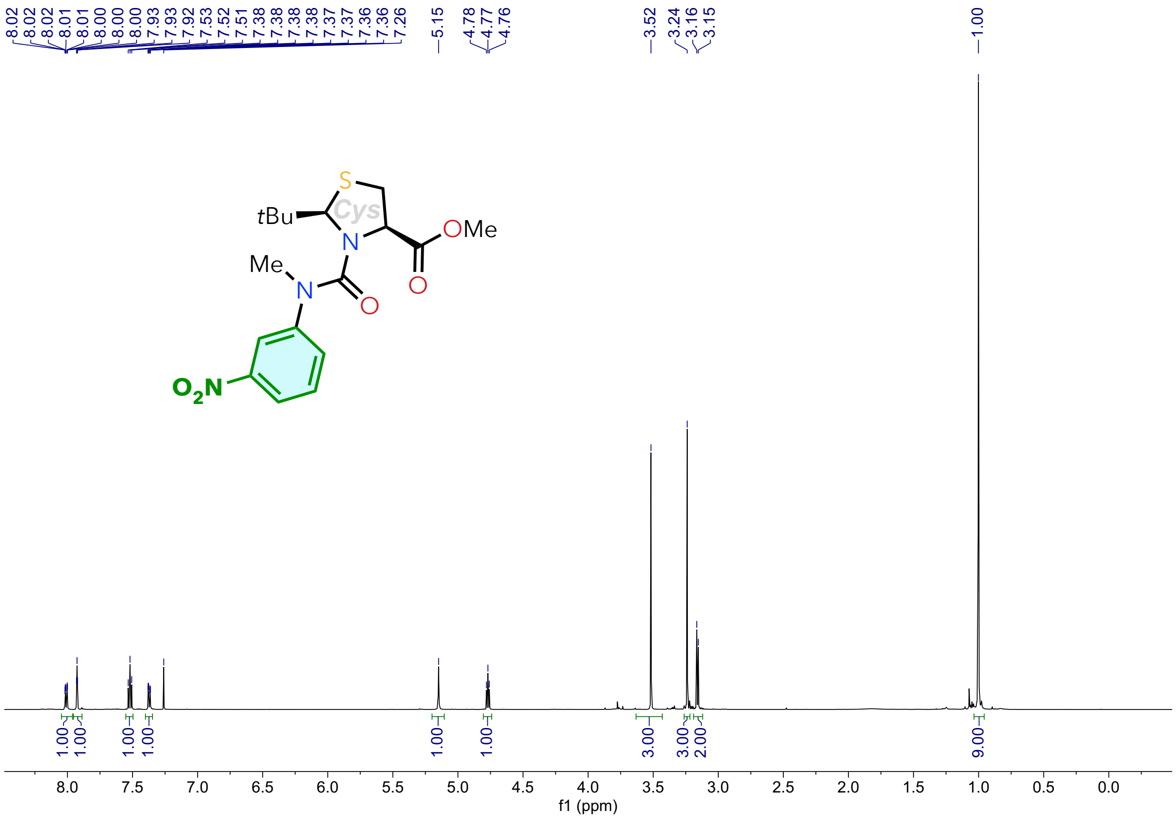
8.02 8.02 8.01 8.01 8.00 8.00 8.00 7.93 7.93 7.92 7.53 7.52 7.51 7.38 7.38 7.38 7.38 7.37 7.37 7.36 7.36 7.26
5.15
4.78 4.77 4.76
3.52
3.24 3.16 3.15
1.00
tBu
S
Cys
N
OMe
O
Me
N
O
O2N
1.00 1.00 1.00 1.00 1.00 1.00 3.00 3.00 2.00 9.00
f1 (ppm)

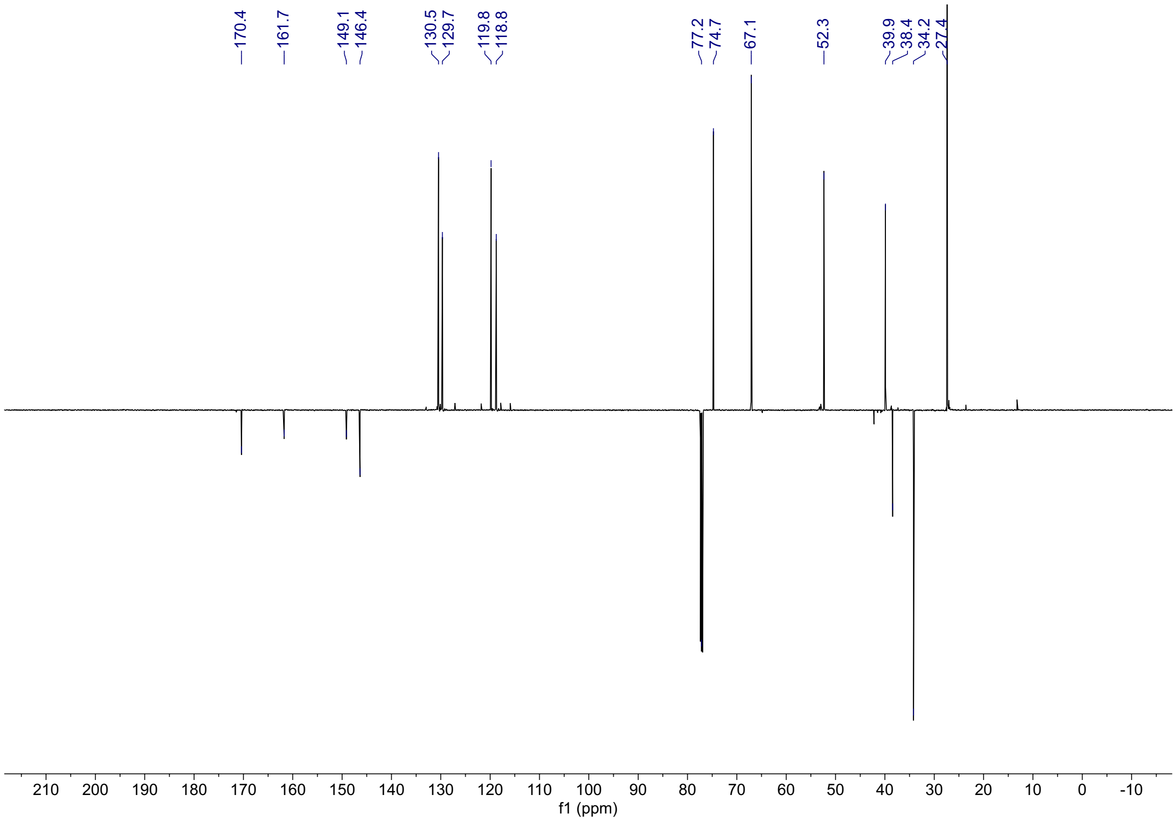
170.4
161.7
149.1 146.4
130.5 129.7
119.8 118.8
77.2 74.7
67.1
52.3
39.9 38.4 34.2 27.4
f1 (ppm)

Methyl (2*R*,4*R*)-2-(*tert*-butyl)-3-((3-methoxyphenyl)(methyl)carbamoyl)thiazolidine-4-carboxylate (19j)

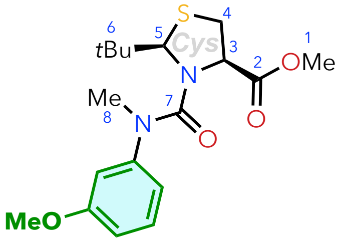

19j

Following **GP3**, *N*-chloroformylthiozolidine **15** (500-mg scale) was used as the carbamoyl chloride and 3-methoxy-*N*-methylaniline (0.37 mL, 2.82 mmol, 1.5 equiv.) as the aniline in DCE. The title compound **19j** was obtained as a red oil (604 mg, 1.65 mmol, 88%) after purification by silica gel column chromatography (PE/EA, gradient elution).

Formula: $C_{18}H_{26}N_2O_4S$, **MW:** 366.48 g/mol. **TLC:** R_f = 0.39 (PE/EA 2:1), $KMnO_4$ stain. **^{1}H NMR** (500 MHz, $CDCl_3$): δ [ppm] = 7.25 (t, *J* 8.1 Hz, 1H, Ar), 6.73 (ddd, *J* 8.3, 2.5, 0.8 Hz, 1H, Ar), 6.65 (ddd, *J* 7.9, 2.0, 0.8 Hz, 1H, Ar), 6.58 (t, *J* 2.2 Hz, 1H, Ar), 5.05 (s, 1H, H-5), 4.77 (dd, *J* 7.5, 6.3 Hz, 1H, H-3), 3.80 (s, 3H, ArOMe), 3.52 (s, 3H, H-1, OMe), 3.17 (s, 3H, H-8, NMe), 3.10 (dd, *J* 6.9, 5.1 Hz, 1H, H-4), 0.99 (s, 9H, *t*Bu). **^{13}C NMR** (125 MHz, $CDCl_3$): δ [ppm] = 171.0 (s, C-2, ester), 161.9 (s, Ar), 160.7 (s, C-7, urea), 158.2 (s, Ar), 146.3 (s, Ar), 130.4 (d, Ar), 117.4 (d, Ar), 111.8 (d, Ar), 110.9 (d, Ar), 74.3 (d, C-5), 67.4 (d, C-3), 55.5 (q, Ar, OMe), 52.1 (q, C-1, OMe), 40.6 (q, C-8, NMe), 38.6 (s, C-6, *t*Bu), 34.1 (t, C-4), 27.3 (q, *t*Bu, 3Me). **FT-IR (ATR):** $\tilde{\nu}$ [cm^{-1}] = 2953 (br w), 1757 (w), 1655 (m), 1597 (s), 1488 (m), 1435 (m), 1362 (s), 1338 (vs), 1297 (m), 1231 (m), 1154 (vs), 1042 (m), 978 (w), 864 (w), 778 (m), 738 (m), 698 (m). **HR-MS:** (ESI) = *m*/*z* calcd. for: $C_{18}H_{27}N_2O_4S$ $[M+H]^+$ 367.1692 u, found: 367.1674 u. **$[a]_\lambda^T$:** (c = 1.00 g/100 mL, $CHCl_3$) = $[a]_D^{20}$: –21.60°.

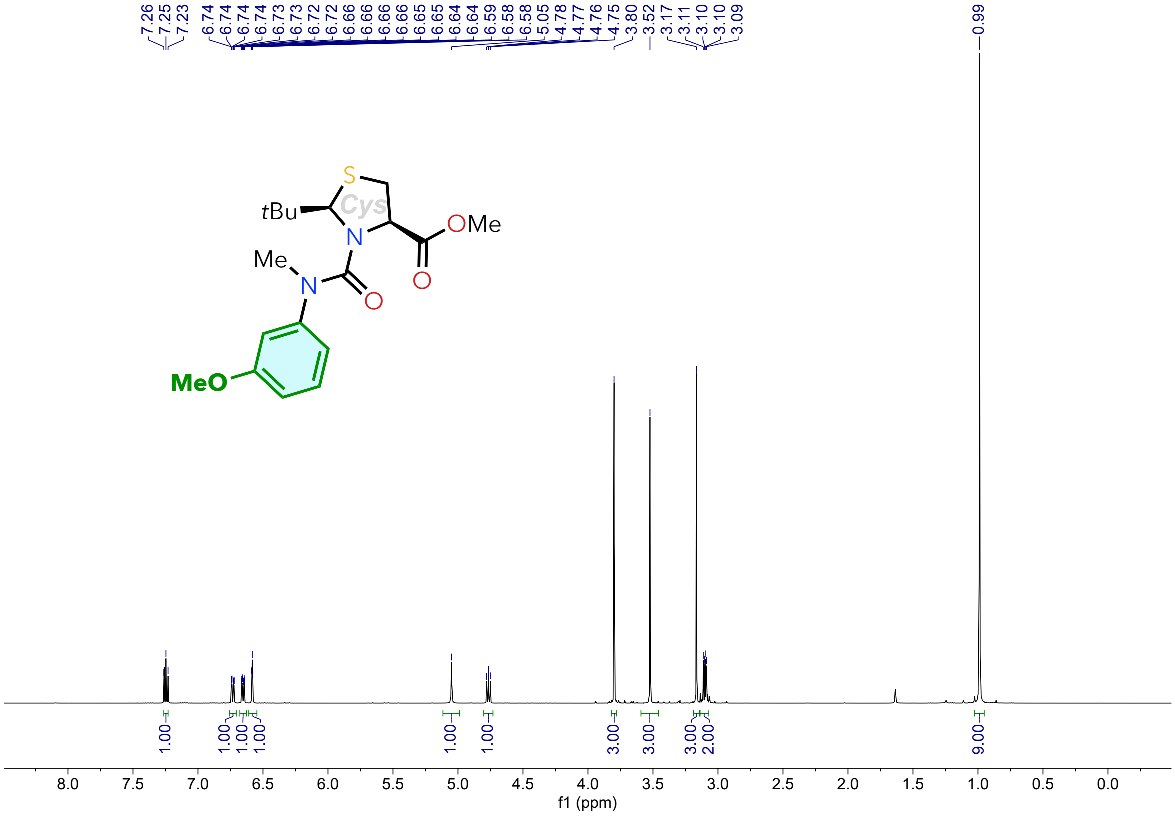
7.26
7.25
7.23
6.74
6.74
6.74
6.74
6.73
6.73
6.72
6.72
6.66
6.66
6.66
6.66
6.65
6.65
6.64
6.64
6.59
6.58
6.58
5.05
4.78
4.77
4.76
4.75
3.80
3.52
3.17
3.11
3.10
3.10
3.09
0.99
S
Cys
tBu
N
OMe
O
Me
N
O
MeO
1.00
1.00
1.00
1.00
1.00
1.00
3.00
3.00
3.00
2.00
9.00
8.0
7.5
7.0
6.5
6.0
5.5
5.0
4.5
4.0
3.5
3.0
2.5
2.0
1.5
1.0
0.5
0.0
f1 (ppm)

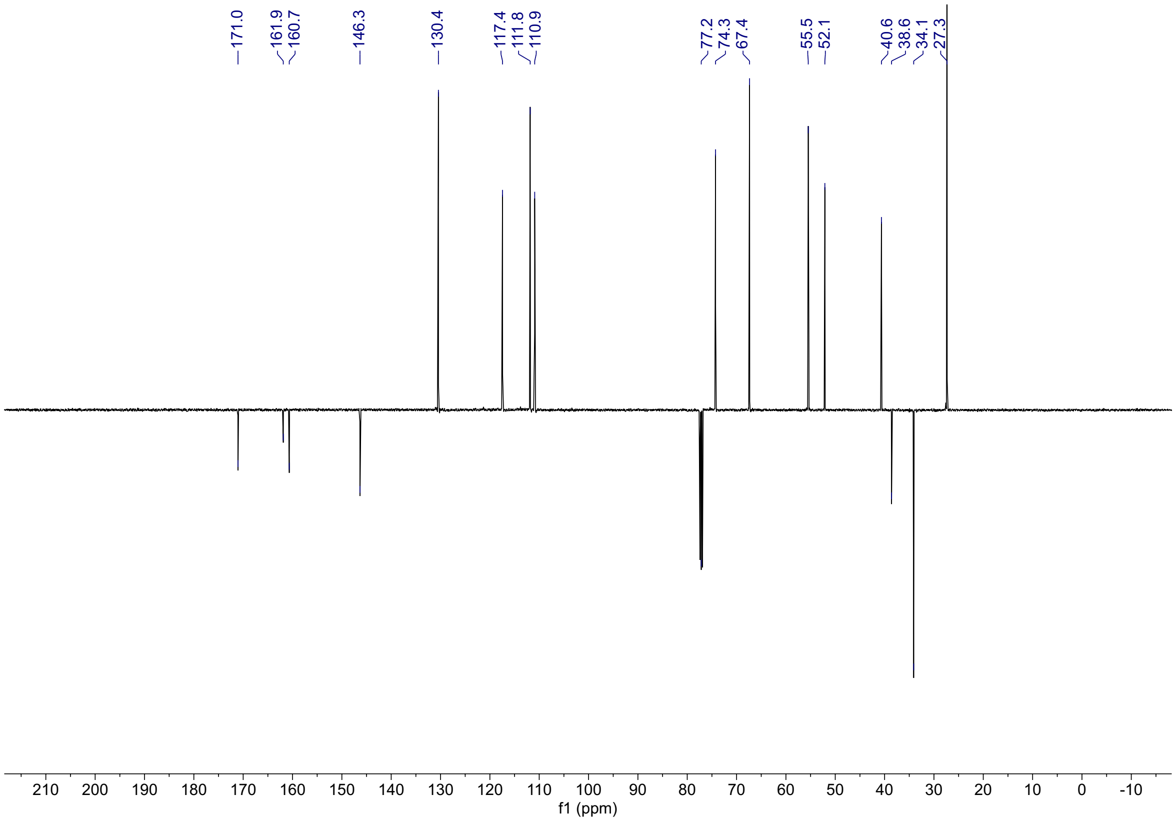
171.0
161.9
160.7
146.3
130.4
117.4
111.8
110.9
77.2
74.3
67.4
55.5
52.1
40.6
38.6
34.1
27.3
210
200
190
180
170
160
150
140
130
120
110
100
90
80
70
60
50
40
30
20
10
0
-10
f1 (ppm)

Methyl (2*R*,4*R*)-2-(*tert*-butyl)-3-(methyl(*p*-tolyl)carbamoyl)thiazolidine-4-carboxylate (19k)

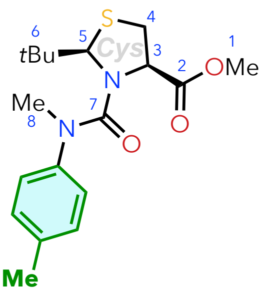

19k

Following **GP3**, *N*-chloroformylthiozolidine **15** (500-mg scale) was used as the carbamoyl chloride and *N*-methyl-*p*-toluidine (0.36 mL, 2.82 mmol, 1.5 equiv.) as the aniline in DCE. The title compound **19k** was obtained as a yellow oil (604 mg, 1.72 mmol, 92%) after purification by silica gel column chromatography (PE/EA, gradient elution).

Formula: $C_{18}H_{26}N_2O_3S$, **MW:** 350.48 g/mol. **TLC:** R_f = 0.43 (PE/EA 2:1), $KMnO_4$ stain. **^{1}H NMR** (500 MHz, $CDCl_3$): δ [ppm] = 7.17 (d, *J* 8.0 Hz, 2H, Ar), 6.98 (d, *J* 8.3 Hz, 2H, Ar), 5.18 (s, 1H, H-5), 4.78 (dd, *J* 7.4, 5.8 Hz, 1H, H-3), 3.47 (s, 3H, H-1, OMe), 3.16 (s, 3H, H-8, NMe), 3.14 – 3.07 (m, 2H, H-4), 2.36 (s, 3H, ArOMe), 1.01 (s, 9H, *t*Bu). **^{13}C NMR** (125 MHz, $CDCl_3$): δ [ppm] = 171.0 (s, C-2, ester), 162.3 (s, C-7, urea), 142.6 (s, Ar), 136.0 (s, Ar), 130.4 (d, 2Ar), 125.9 (d, 2Ar), 74.3 (d, C-5), 67.6 (d, C-3), 51.9 (q, C-1, OMe), 41.0 (q, C-8, NMe), 38.4 (s, C-6, *t*Bu), 34.3 (t, C-4), 27.4 (q, *t*Bu, 3Me), 21.0 (q, Ar, Me). **FT-IR (ATR):** $\tilde{\nu}$ [cm^{-1}] = 2953 (br w), 1759 (m), 1730 (w), 1652 (s), 1512 (s), 1435 (m), 1362 (m), 1338 (vs), 1296 (m), 1196 (m), 1157 (s), 1019 (w), 964 (w), 863 (w), 879 (w), 742 (m), 612 (w), 552 (w). **HR-MS:** (ESI) = *m*/*z* calcd. for: $C_{18}H_{27}N_2O_3S$ $[M+H]^+$ 351.1742 u, found: 351.1726 u. **$[a]_\lambda^T$:** (c = 1.00 g/100 mL, $CHCl_3$) = $[a]_D^{20}$: –24.00°.

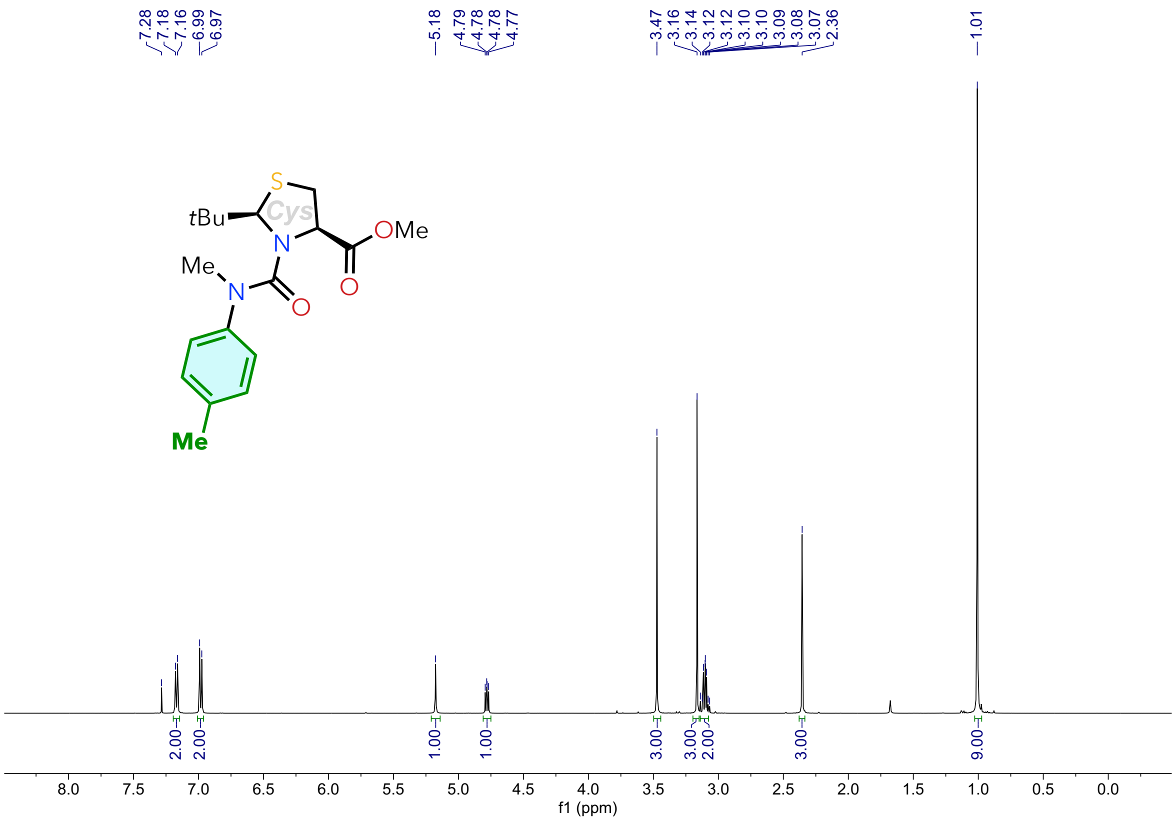
7.28
7.18
7.16
6.99
6.97
5.18
4.79
4.78
4.78
4.77
3.47
3.16
3.14
3.12
3.12
3.10
3.10
3.09
3.08
3.07
2.36
1.01
tBu
S
Cys
N
OMe
O
Me
N
O
Me
2.00
2.00
1.00
1.00
3.00
3.00
2.00
3.00
9.00
f1 (ppm)

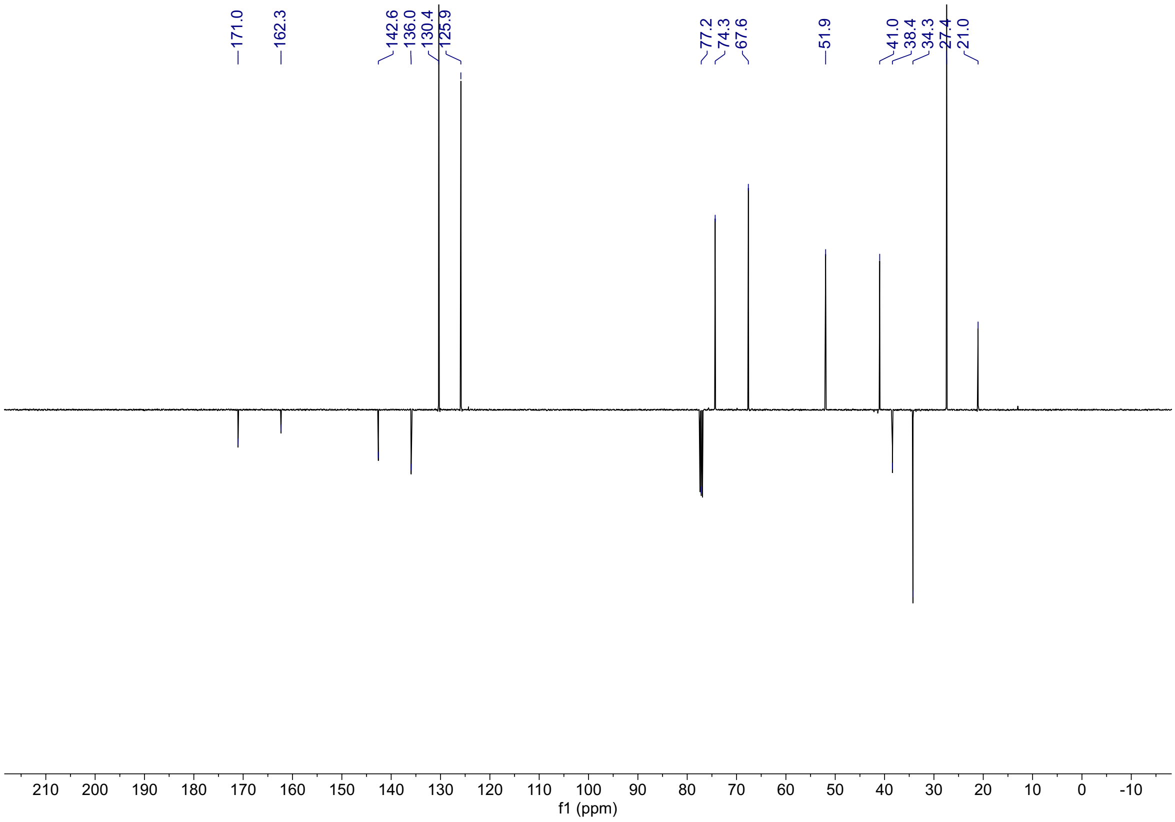
171.0
162.3
142.6
136.0
130.4
125.9
77.2
74.3
67.6
51.9
41.0
38.4
34.3
27.4
21.0
f1 (ppm)

Methyl (2*R*,4*R*)-2-(*tert*-butyl)-3-((3,5-difluorophenyl)(methyl)carbamoyl)thiazolidine-4-carboxylate (191)

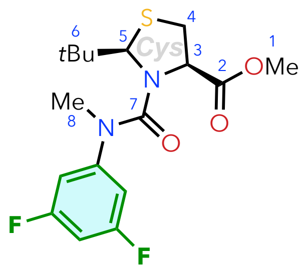

191

Following **GP3**, *N*-chloroformylthiozolidine **15** (500-mg scale) was used as the carbamoyl chloride and 3,5-difluoro-*N*-methylaniline (0.33 mL, 2.82 mmol, 1.5 equiv.) as the aniline in DCE. The title compound **191** was obtained as a yellow oil (321 mg, 0.86 mmol, 46%) after purification by silica gel column chromatography (PE/EA, gradient elution).

Formula: $C_{17}H_{22}F_2N_2O_3S$, **MW:** 372.43 g/mol. **TLC:** R_f = 0.52 (PE/EA 2:1), $KMnO_4$ stain. **^{1}H NMR** (600 MHz, $CDCl_3$): δ [ppm] = 6.59 (tt, *J* 8.8, 2.2 Hz, 1H, Ar), 6.54 (td, *J* 8.5, 2.1 Hz, 2H, Ar), 5.00 (s, 1H, H-5), 4.73 (t, *J* 7.2 Hz, 1H, H-3), 3.64 (s, 3H, H-1, OMe), 3.17 (d, *J* 7.2 Hz, 2H, H-4), 3.15 (s, 3H, H-8, NMe), 1.00 (s, 9H, *t*Bu). **^{13}C NMR** (150 MHz, $CDCl_3$): δ [ppm] = 170.7 (s, C-2, ester), 164.4 (s, *d J* 14.8 Hz, Ar), 162.8 (s, *d J* 14.8 Hz, Ar), 161.1 (s, C-7, urea), 147.5 (s, *t J* 12.2 Hz, Ar), 106.3 (d, *d J* 6.3 Hz, Ar), 106.1 (d, *d J* 6.2 Hz, Ar), 100.3 (d, *t J* 25.5 Hz, Ar), 74.5 (d, C-5), 66.8 (d, C-3), 52.4 (q, C-1, OMe), 39.6 (q, C-8, NMe), 38.8 (s, C-6, *t*Bu), 34.0 (t, C-4), 27.3 (q, *t*Bu, 3Me). **^{19}F NMR** (565 MHz, $CDCl_3$, C_6F_6 ref.): δ [ppm] = –111.33 (t, *J* 8.3 Hz, 2F). **FT-IR (ATR):** $\tilde{\nu}$ [cm^{-1}] = 2956 (br w), 1756 (m), 1662 (m), 1617 (s), 1593 (s), 1479 (m), 1460 (m), 1436 (m), 1365 (m), 1319 (s), 1234 (w), 1196 (m), 1169 (m), 1153 (m), 1118 (vs), 990 (s), 954 (w), 840 (m), 786 (w), 723 (w), 693 (w), 607 (w). **HR-MS:** (ESI) = *m/z* calcd. for: $C_{17}H_{23}F_2N_2O_3S$ $[M+H]^+$ 373.1397 u, found: 373.1380 u. **$[a]^{T}_{\lambda}$:** (c = 1.21 g/100 mL, $CHCl_3$) = $[a]^{20}_{D}$: –24.13°.

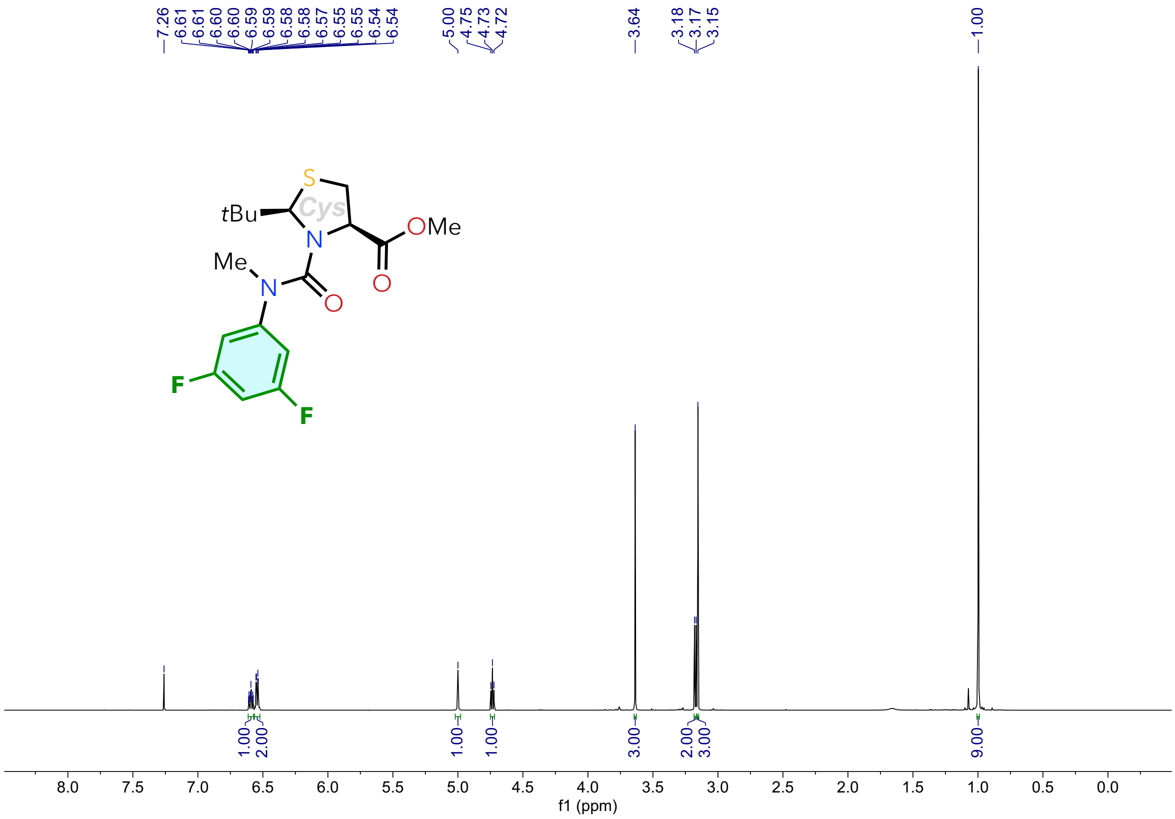
7.26
6.61
6.61
6.60
6.60
6.59
6.59
6.58
6.58
6.57
6.55
6.55
6.54
6.54
5.00
4.75
4.73
4.72
3.64
3.18
3.17
3.15
1.00
S
tBu
Cys
N
OMe
O
Me
N
O
F
F
1.00
2.00
1.00
1.00
3.00
2.00
3.00
9.00
8.0 7.5 7.0 6.5 6.0 5.5 5.0 4.5 4.0 3.5 3.0 2.5 2.0 1.5 1.0 0.5 0.0
f1 (ppm)

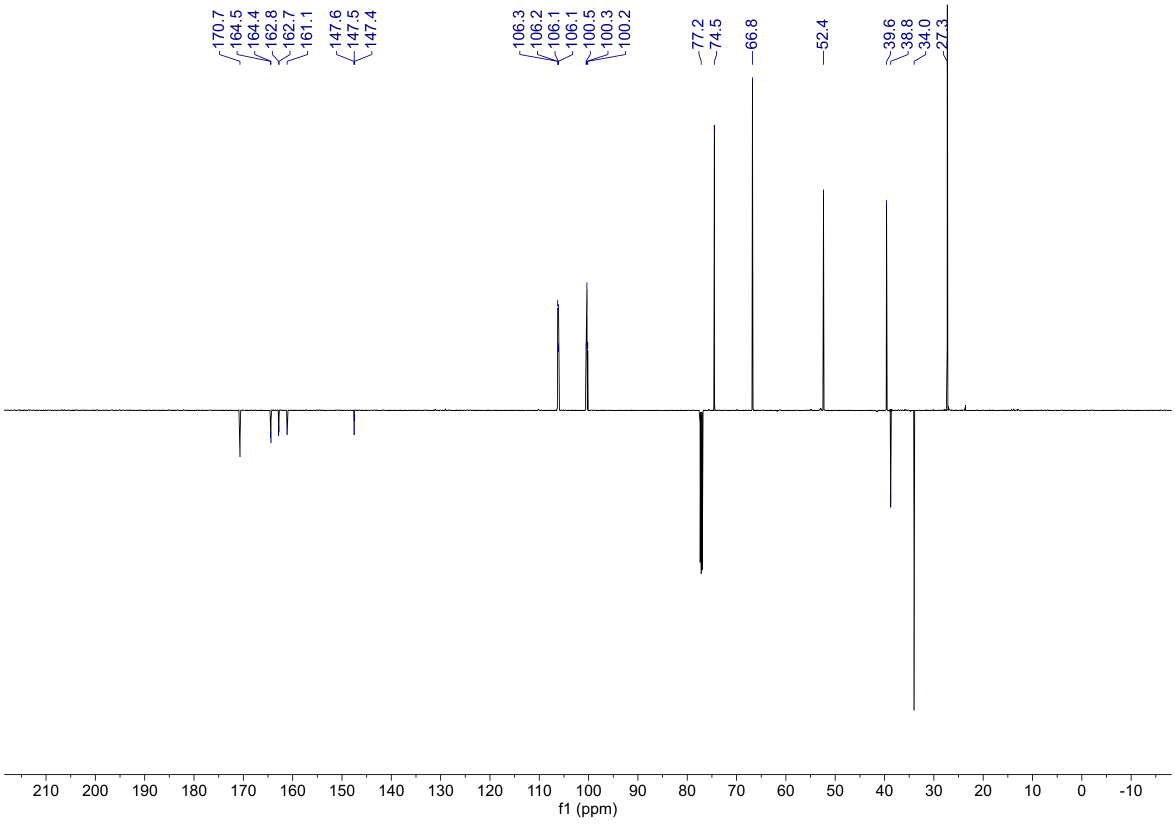
170.7
164.5
164.4
162.8
162.7
161.1
147.6
147.5
147.4
106.3
106.2
106.1
106.1
100.5
100.3
100.2
77.2
74.5
66.8
52.4
39.6
38.8
34.0
27.3
210 200 190 180 170 160 150 140 130 120 110 100 90 80 70 60 50 40 30 20 10 0 -10
f1 (ppm)

Methyl (2*R*,4*R*)-2-(*tert*-butyl)-3-(methyl(pyridin-2-yl)carbamoyl)thiazolidine-4-carboxylate (19m)

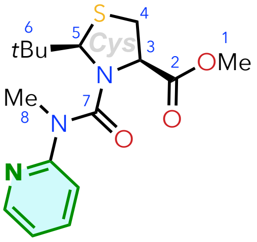

19m

Following **GP3**, *N*-chloroformylthiozolidine **15** (500-mg scale) was used as the carbamoyl chloride and *N*-methylpyridin-2-amine (0.29 mL, 2.82 mmol, 1.5 equiv.) as the aniline in DCE. The title compound **19m** was obtained as a red oil (216 mg, 0.64 mmol, 34%) after purification by silica gel column chromatography (PE/EA, gradient elution).

Formula: $C_{16}H_{23}N_3O_3S$, **MW:** 337.44 g/mol. **TLC:** R_f = 0.27 (PE/EA 2:1), $KMnO_4$ stain. **^{1}H NMR** (500 MHz, $CDCl_3$): δ [ppm] = 8.31 (ddd, *J* 4.9, 1.9, 0.8 Hz, 1H, Ar), 7.61 (ddd, *J* 8.3, 7.3, 2.0 Hz, 1H, Ar), 6.92 (ddd, *J* 7.3, 4.9, 0.9 Hz, 1H, Ar), 6.82 (dt, *J* 8.3, 0.7 Hz, 1H, Ar), 4.90 (d, *J* 5.1 Hz, 1H, H-5), 4.88 (d, *J* 7.7 Hz, 1H, H-3), 3.68 (s, 3H, H-1, OMe), 3.22 (d, *J* 0.8 Hz, 1H, H-4a), 3.21 (s, 3H, H-8, NMe), 3.21 (d, *J* 1.2 Hz, 1H, H-4b), 1.00 (s, 9H, *t*Bu). **^{13}C NMR** (125 MHz, $CDCl_3$): δ [ppm] = 171.0 (s, C-2, ester), 161.5 (s, C-7, urea), 157.0 (s, Ar), 148.7 (d, Ar), 138.2 (d, Ar), 118.1 (d, Ar), 113.5 (d, Ar), 74.4 (d, C-5), 66.3 (d, C-3), 52.4 (q, C-1, OMe), 39.0 (s, C-6, *t*Bu), 36.4 (q, C-8, NMe), 33.7 (t, C-4), 27.2 (q, *t*Bu, 3Me). **FT-IR (ATR):** $\tilde{\nu}$ [cm^{-1}] = 2956 (br w), 1754 (m), 1664 (s), 1591 (m), 1569 (m), 1478 (s), 1438 (m), 1422 (m), 1363 (vs), 1337 (vs), 1300 (s), 1266 (m), 1172 (vs), 987 (w), 965 (w), 914 (w), 733 (s), 647 (w). **HR-MS:** (ESI) = *m*/*z* calcd. for: $C_{16}H_{24}N_3O_3S$ $[M+H]^+$ 338.1538 u, found: 338.1521 u. **$[a]_\lambda^T$:** (c = 1.00 g/100 mL, $CHCl_3$) = $[a]_D^{20}$: –21.20°.

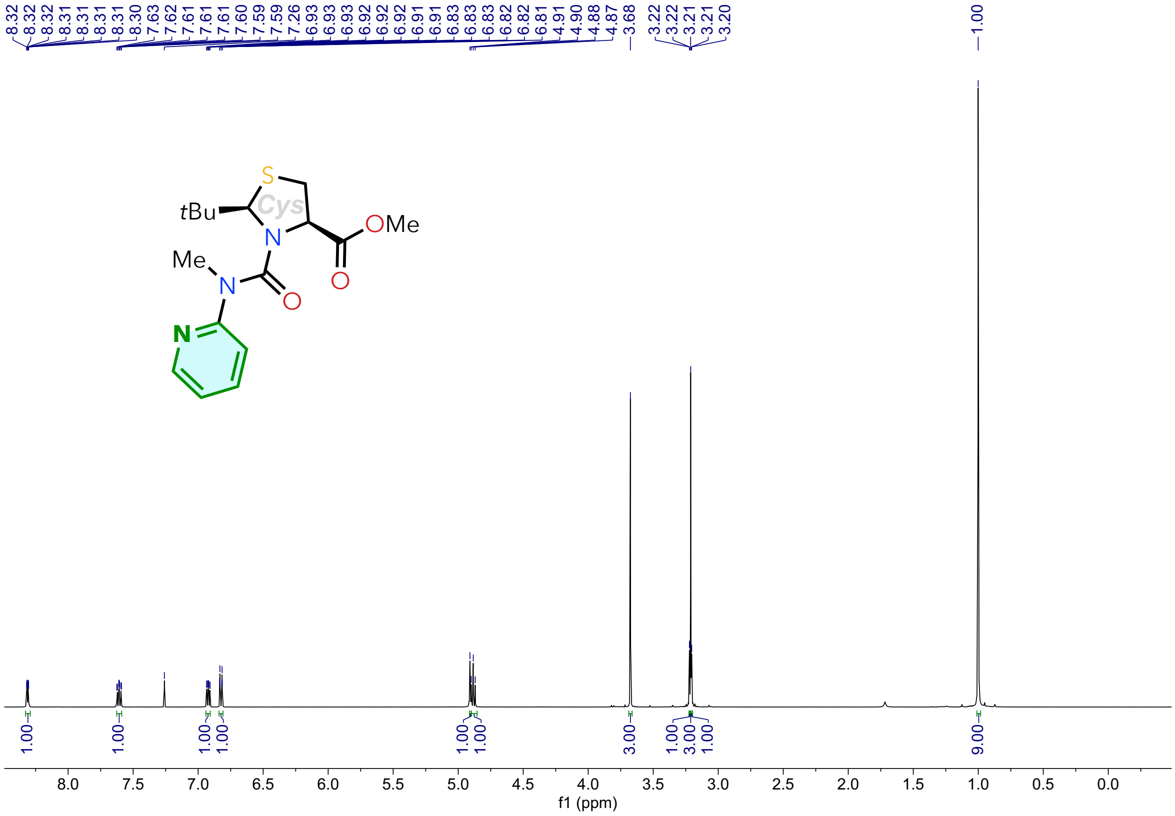
8.32 8.32 8.32 8.31 8.31 8.31 8.30 7.63 7.62 7.61 7.61 7.60 7.59 7.59 7.26 6.93 6.93 6.93 6.92 6.92 6.92 6.91 6.91 6.83 6.83 6.83 6.82 6.82 6.81 4.91 4.90 4.88 4.87
3.68
3.22 3.22 3.21 3.21 3.20
1.00
S
Cys
tBu
N
OMe
O
Me
N
O
N
1.00
1.00
1.00
1.00
1.00
1.00
3.00
1.00
3.00
1.00
9.00
8.0 7.5 7.0 6.5 6.0 5.5 5.0 4.5 4.0 3.5 3.0 2.5 2.0 1.5 1.0 0.5 0.0
f1 (ppm)

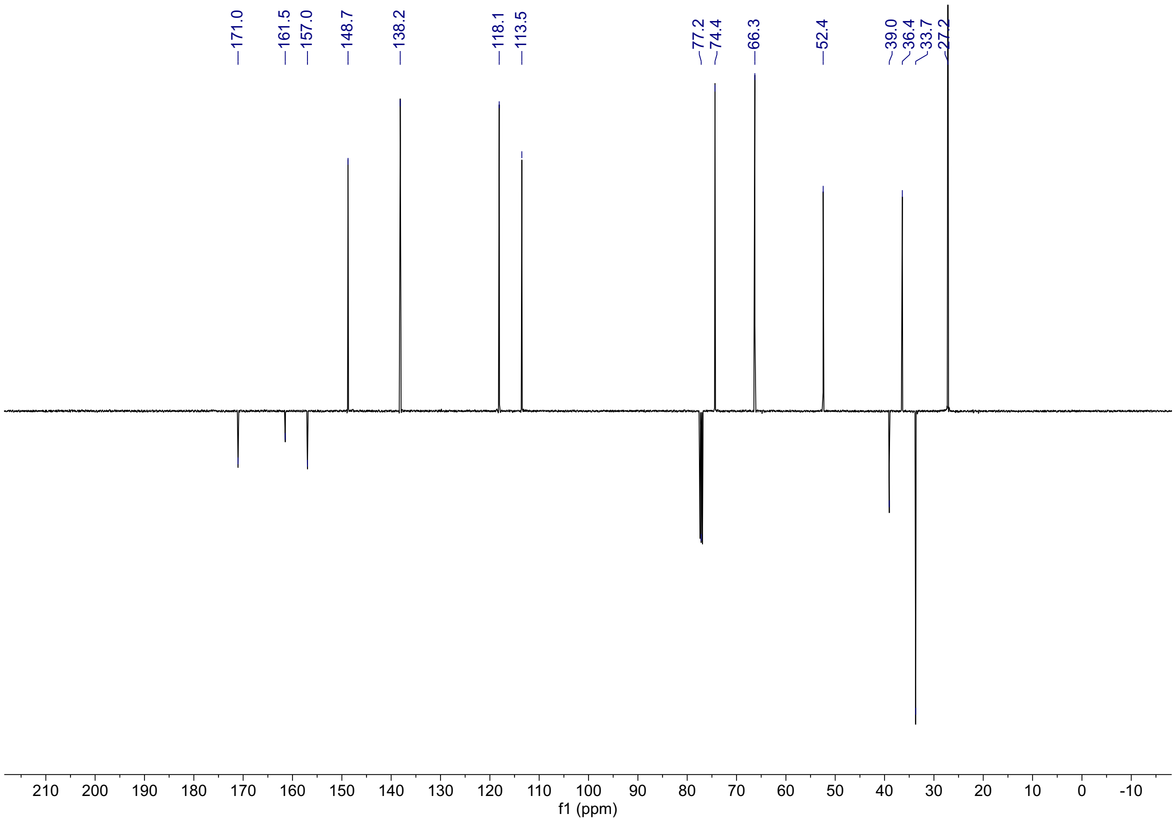
171.0
161.5
157.0
148.7
138.2
118.1
113.5
77.2
74.4
66.3
52.4
39.0
36.4
33.7
27.2
210 200 190 180 170 160 150 140 130 120 110 100 90 80 70 60 50 40 30 20 10 0 -10
f1 (ppm)

Methyl (2*R*,4*R*)-2-(*tert*-butyl)-3-(methyl(naphthalen-2-yl)carbamoyl)thiazolidine-4-carboxylate (19n)

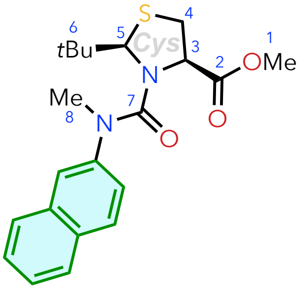

19n

Following **GP3**, *N*-chloroformylthiozolidine **15** (500-mg scale) was used as the carbamoyl chloride and *N*-methylnaphthalen-2-amine hydrochloride (546 mg, 2.82 mmol, 1.5 equiv.) as the aniline in DCE. A slight excess of triethylamine (0.78 mL, 5.64 mmol, 3.0 equiv.) was used in this reaction. The title compound **19n** was obtained as a pink solid (468 mg, 1.21 mmol, 64%) after purification by silica gel column chromatography (PE/EA, gradient elution).

Formula: $C_{21}H_{26}N_2O_3S$, **MW:** 386.51 g/mol, **m.p.:** 139 – 142 °C. **TLC:** R_f = 0.53 (PE/EA 2:1), $KMnO_4$ stain. **^{1}H NMR** (500 MHz, $CDCl_3$): δ [ppm] = 7.96 (d, *J* 8.4 Hz, 1H, Ar), 7.91 (d, *J* 8.1 Hz, 1H, Ar), 7.82 (d, *J* 8.3 Hz, 1H, Ar), 7.61 (ddd, *J* 8.3, 6.9, 1.3 Hz, 1H, Ar), 7.56 (ddd, *J* 8.0, 6.9, 1.2 Hz, 1H, Ar), 7.53 – 7.47 (m, 1H, Ar), 7.31 (dd, *J* 7.3, 0.9 Hz, 1H, Ar) 5.57 (s, 1H, H-5), 4.74 (dd, *J* 7.7, 4.4 Hz, 1H, H-3), 3.27 (s, 3H, H-1, OMe), 3.10 (dd, *J* 11.7, 7.8 Hz, 1H, H-4a), 2.99 (d, *J* 4.4 Hz, 1H, H-4b), 2.96 (s, 3H, H-8, NMe), 1.00 (s, 9H, *t*Bu). **^{13}C NMR** (125 MHz, $CDCl_3$): δ [ppm] = 170.0 (s, C-2, ester), 163.9 (s, C-7, urea), 141.0 (s, Ar), 134.9 (s, Ar), 129.8 (s, Ar), 128.0 (d, Ar), 127.3 (d, Ar), 126.6 (d, Ar), 126.4 (d, Ar), 126.2 (d, Ar), 122.4 (d, Ar), 75.3 (d, C-5), 67.3 (d, C-3), 51.5 (q, C-1, OMe), 40.9 (q, C-8, NMe), 38.0 (s, C-6, *t*Bu), 34.9 (t, C-4), 27.5 (q, *t*Bu, 3Me). **FT-IR (ATR):** $\tilde{\nu}$ [cm^{-1}] = 2949 (br w), 1758 (m), 1640 (vs), 1433 (w), 1421 (m), 1391 (w), 1334 (vs), 1295 (m), 1206 (m), 1166 (s), 1160 (s), 1139 (m), 1030 (w), 1009 (m), 878 (w), 779 (vs), 735 (w), 690 (w), 660 (w). **HR-MS:** (ESI) = *m/z* calcd. for: $C_{21}H_{27}N_2O_3S$ $[M+H]^+$ 387.1742 u, found: 387.1725 u. **$[a]_\lambda^T$:** (c = 1.00 g/100 mL, $CHCl_3$) = $[a]_D^{20}$: –22.80°.

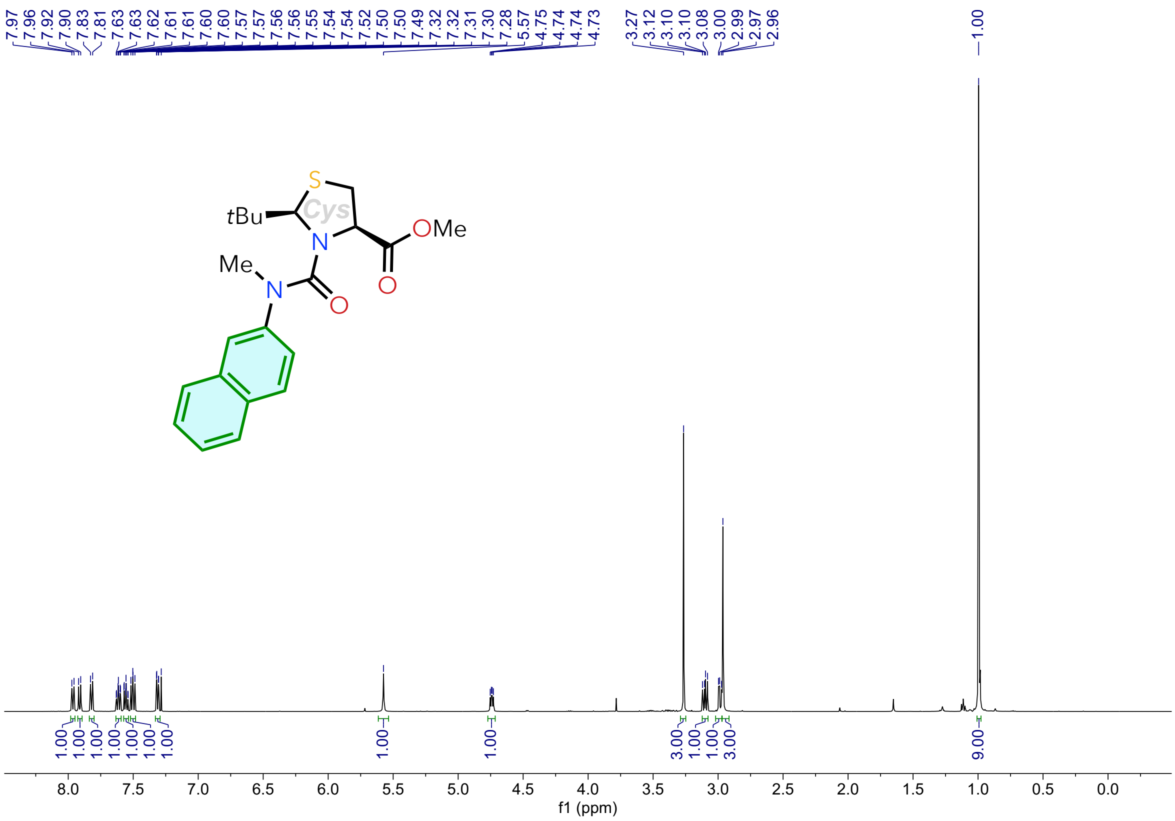

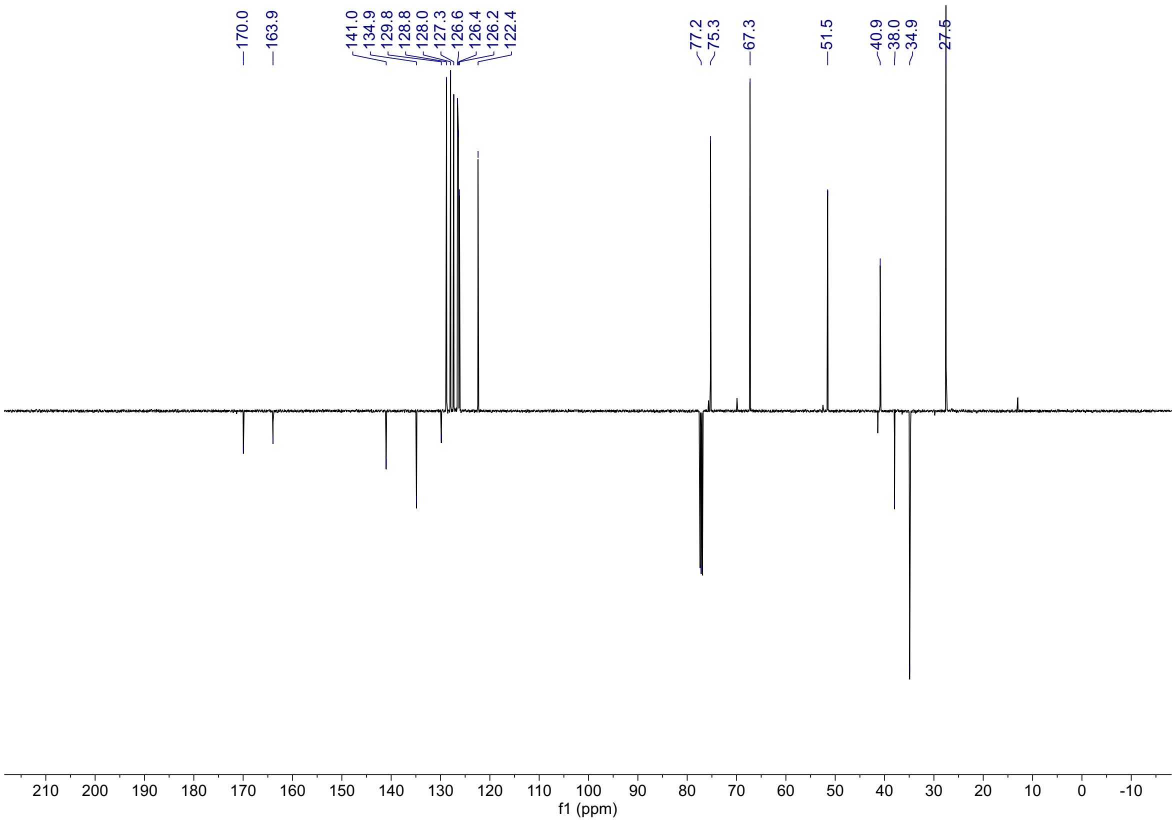

1-(*tert*-Butyl) 4-methyl (2*R*,4*S*)-2-(*tert*-butyl)-3-(methyl(phenyl)carbamoyl)imidazolidine-1,4-dicarboxylate (20a)

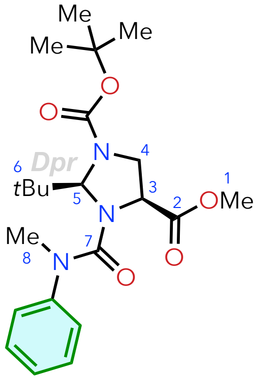

20a

Following **GP3**, to a solution of *N*-chloroformylimidazolidine **16** (1.23 g, 3.52 mmol, 1.0 equiv.) in DCE (4.4 mL, 0.8 M) was added triethylamine (0.98 mL, 7.04 mmol, 2.0 equiv.) and *N*-methylaniline (0.57 mL, 5.28 mmol, 1.5 equiv.). The reaction mixture was heated to reflux (85 °C) for 18 h. The product **20a** was obtained as a colourless oil (1.09 g, 2.60 mmol, 74%) after purification by silica gel column chromatography (gradient elution, PE/EA).

Formula: $C_{22}H_{33}N_3O_5$, **MW:** 419.52 g/mol. **TLC:** R_f = 0.40 (PE/EA 2:1), $KMnO_4$ stain. **^{1}H NMR** (600 MHz, $CDCl_3$): δ [ppm] = 7.33 (t, *J* 7.8 Hz, 2H, Ar), 7.20 (t, *J* 7.4 Hz, 2H, Ar), 7.00 (d, *J* 7.6 Hz, 2H, Ar), 5.43 (s, 1H, H-5), 4.50 (dd, *J* 10.0, 8.1 Hz, 1H, H-3), 4.41 (br s, 1H, H-4a), 3.54 (s, 3H, H-1, OMe), 3.22 – 3.19 (m, 1H, H-4b), 3.11 (s, 3H, H-8, NMe), 1.36 (s, 9H, Boc, *t*Bu), 0.96 (s, 9H, *t*Bu). **^{13}C NMR** (150 MHz, $CDCl_3$): δ [ppm] = 170.9 (s, C-2, ester), 161.3 (s, C-7, urea), 153.8 (s, Boc), 145.7 (s, Ar), 130.0 (d, 2Ar), 126.1 (d, Ar), 125.1 (d, 2Ar), 81.0 (s, Boc, *t*Bu), 79.5 (d, C-5), 60.5 (d, C-3), 52.2 (q, C-1, OMe), 47.9 (t, C-4), 41.3 (q, C-8, NMe), 39.1 (s, C-6, *t*Bu), 28.2 (q, Boc, *t*Bu, 3Me), 26.8 (q, *t*Bu, 3Me). **FT-IR (ATR):** $\tilde{\nu}$ [cm^{-1}] = 2967 (br w), 1762 (w), 1738 (w), 1697 (m), 1657 (m), 1596 (w), 1495 (w), 1393 (m), 1364 (m), 1344 (m), 1301 (m), 1282 (m), 1241 (w), 1157 (vs), 1107 (m), 1032 (w), 904 (m), 880 (m), 761 (m), 699 (m), 547 (w). **HR-MS:** (ESI) = *m*/*z* calcd. for: $C_{22}H_{34}N_3O_5$ $[M+H]^+$ 420.2498 u, found: 420.2482 u. **$[a]_\lambda^T$:** (c = 1.15 g/100 mL, $CHCl_3$) = $[a]_D^{20}$: –17.39°.

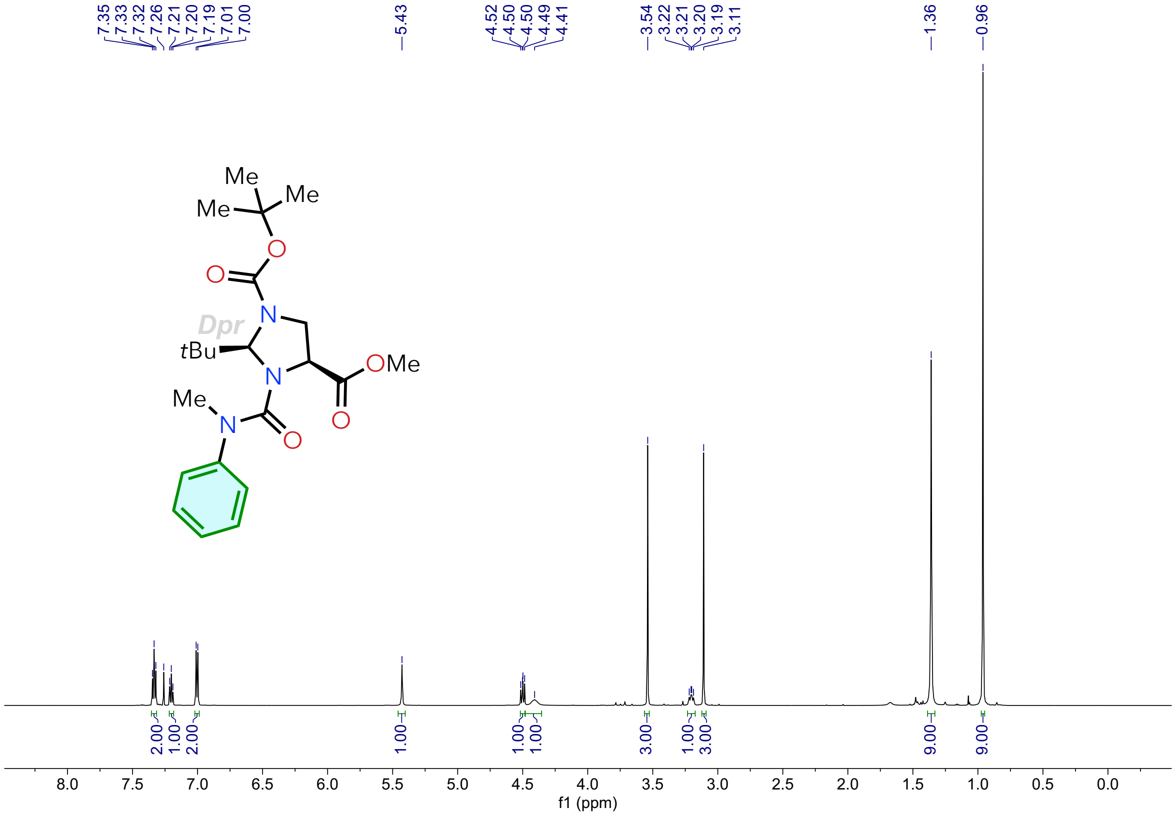
7.35
7.33
7.32
7.26
7.21
7.20
7.19
7.01
7.00
5.43
4.52
4.50
4.50
4.49
4.41
3.54
3.22
3.21
3.20
3.19
3.11
1.36
0.96
Me
Me
Me
O
O
N
Dpr
tBu
N
OMe
O
Me
N
O
2.00
1.00
2.00
1.00
1.00
1.00
3.00
1.00
3.00
9.00
9.00
8.0
7.5
7.0
6.5
6.0
5.5
5.0
4.5
4.0
3.5
3.0
2.5
2.0
1.5
1.0
0.5
0.0
f1 (ppm)

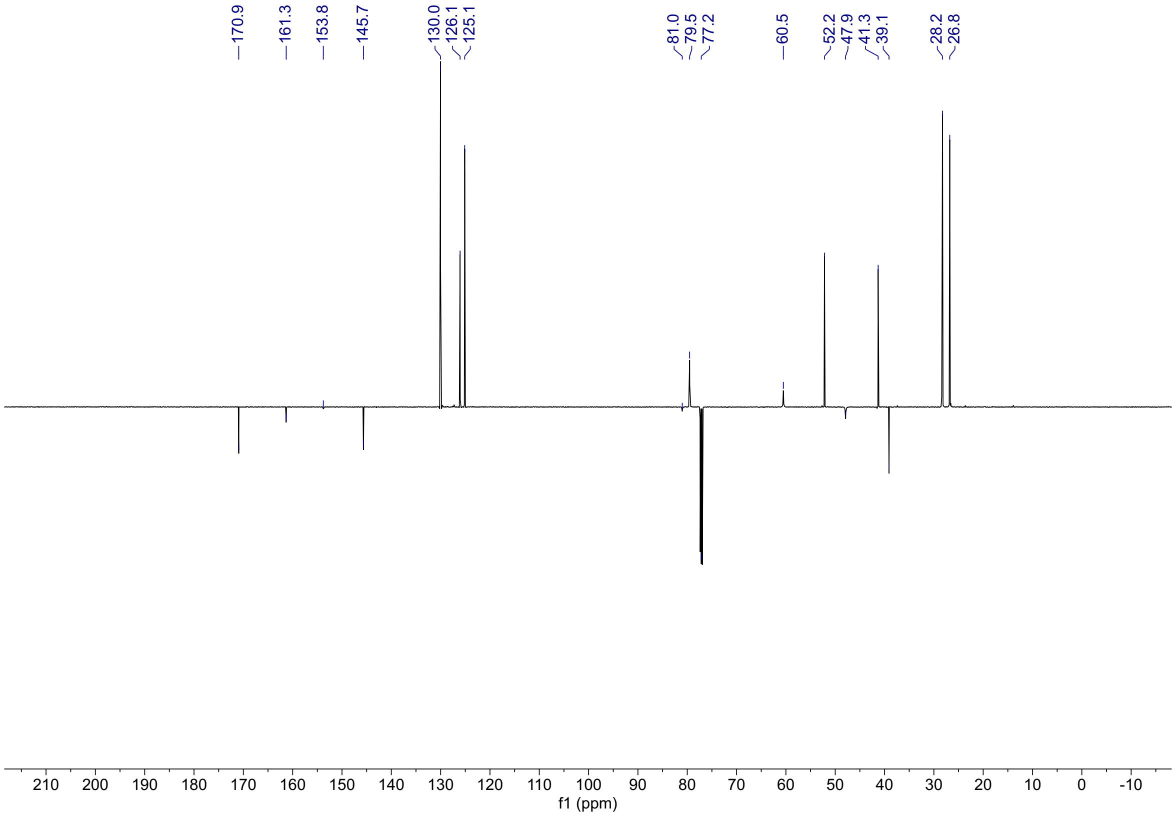
170.9
161.3
153.8
145.7
130.0
126.1
125.1
81.0
79.5
77.2
60.5
52.2
47.9
41.3
39.1
28.2
26.8
210
200
190
180
170
160
150
140
130
120
110
100
90
80
70
60
50
40
30
20
10
0
-10
f1 (ppm)

Methyl (2*R*,4*S*,5*R*)-2-(*tert*-butyl)-5-methyl-3-(methyl(phenyl)carbamoyl)oxazolidine-4-carboxylate (21a)

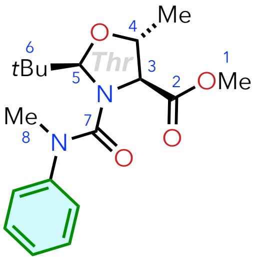

21a

Following **GP3**, to a solution of *N*-chloroformyloxazolidine **17** (500 mg, 1.90 mmol, 1.0 equiv.) in CH_2Cl_2 (2.4 mL, 0.8 M) was added triethylamine (0.53 mL, 3.79 mmol, 2.0 equiv.) and *N*-methylaniline (0.31 mL, 2.84 mmol, 1.5 equiv.). The reaction mixture was heated to reflux (85 °C) for 18 h. The product **21a** was obtained as a colourless oil (474 mg, 1.42 mmol, 75%) after purification by silica gel column chromatography (PE/EA, gradient elution).

Formula: $C_{18}H_{26}N_2O_4$, **MW:** 334.42 g/mol. **TLC:** R_f = 0.38 (PE/EA 2:1), $KMnO_4$ stain. **^{1}H NMR** (600 MHz, $CDCl_3$): δ [ppm] = 7.33 (t, *J* 7.8 Hz, 2H, Ar), 7.20 (t, *J* 7.4 Hz, 1H, Ar), 7.15 (d, *J* 7.5 Hz, 1H, Ar), 5.50 (s, 1H, H-5), 4.26 (q, *J* 6.4 Hz, 1H, H-4), 3.98 (s, 1H, H-3), 3.22 (s, 3H, H-1, OMe), 3.19 (s, 3H, H-8, NMe), 1.23 (d, *J* 6.4 Hz, 3H, Me), 0.99 (s, 9H, *t*Bu). **^{13}C NMR** (150 MHz, $CDCl_3$): δ [ppm] = 169.9 (s, C-2, ester), 163.4 (s, C-7, urea), 144.6 (s, Ar), 129.6 (d, 2Ar), 128.0 (d, 2Ar), 126.6 (d, Ar), 96.1 (d, C-5), 77.5 (d, C-4) 68.7 (d, C-3), 51.5 (q, C-1, OMe), 40.5 (q, C-8, NMe), 36.1 (s, C-6, *t*Bu), 26.4 (q, *t*Bu, 3Me), 19.6 (q, Me). **FT-IR (ATR):** $\tilde{\nu}$ [cm^{-1}] = 2976 (br w), 1745 (m), 1647 (vs), 1596 (m), 1495 (s), 1437 (w), 1365 (m), 1276 (s), 1177 (m), 1154 (m), 993 (w), 648 (s), 515 (w), 464 (w). **HR-MS:** (ESI) = *m*/*z* calcd. for: $C_{18}H_{27}N_2O_4$ $[M+H]^+$ 335.1971 u, found: 335.1951 u. **$[a]_\lambda^T$:** (c = 1.00 g/100 mL, $CHCl_3$) = $[a]_D^{20}$: –20.40°.

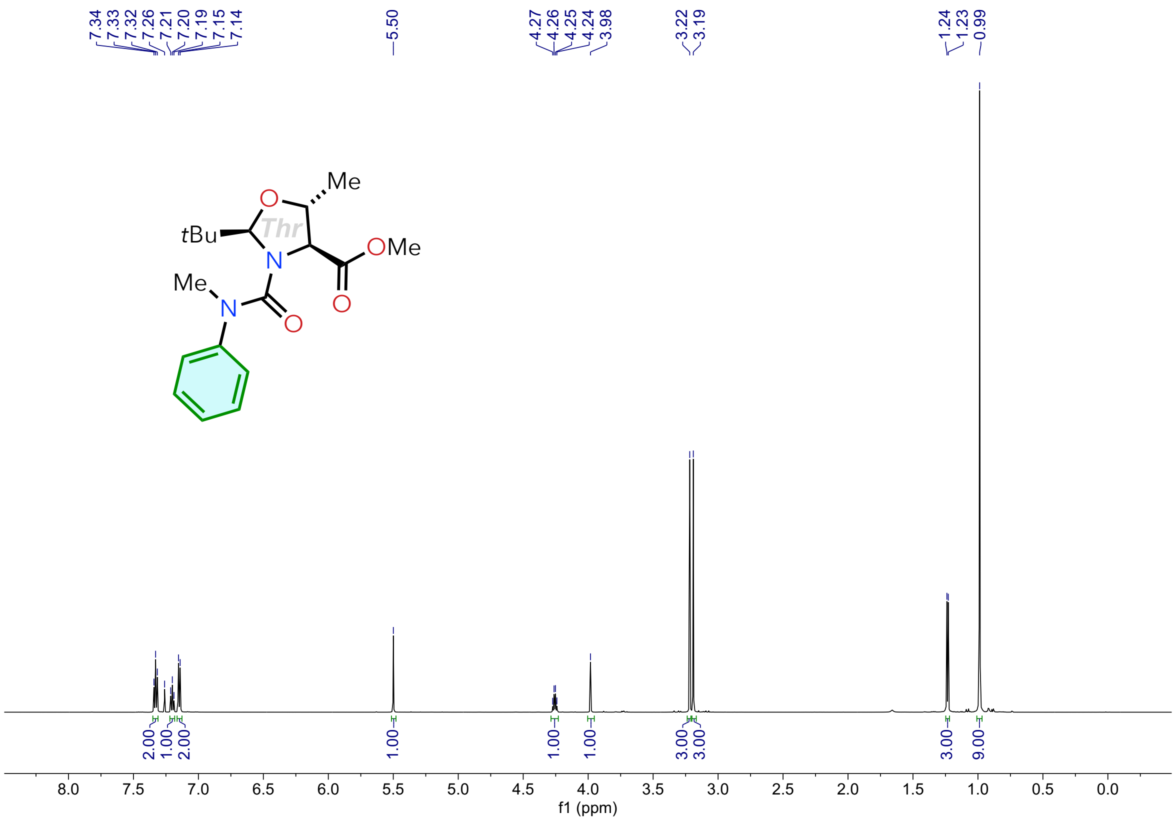

7.34
7.33
7.32
7.26
7.21
7.20
7.19
7.15
7.14
5.50
4.27
4.26
4.25
4.24
3.98
3.22
3.19
1.24
1.23
0.99
Me
O
tBu
Thr
N
OMe
O
Me
N
O
2.00
1.00
2.00
1.00
1.00
1.00
3.00
3.00
3.00
9.00
8.0
7.5
7.0
6.5
6.0
5.5
5.0
4.5
4.0
3.5
3.0
2.5
2.0
1.5
1.0
0.5
0.0
f1 (ppm)

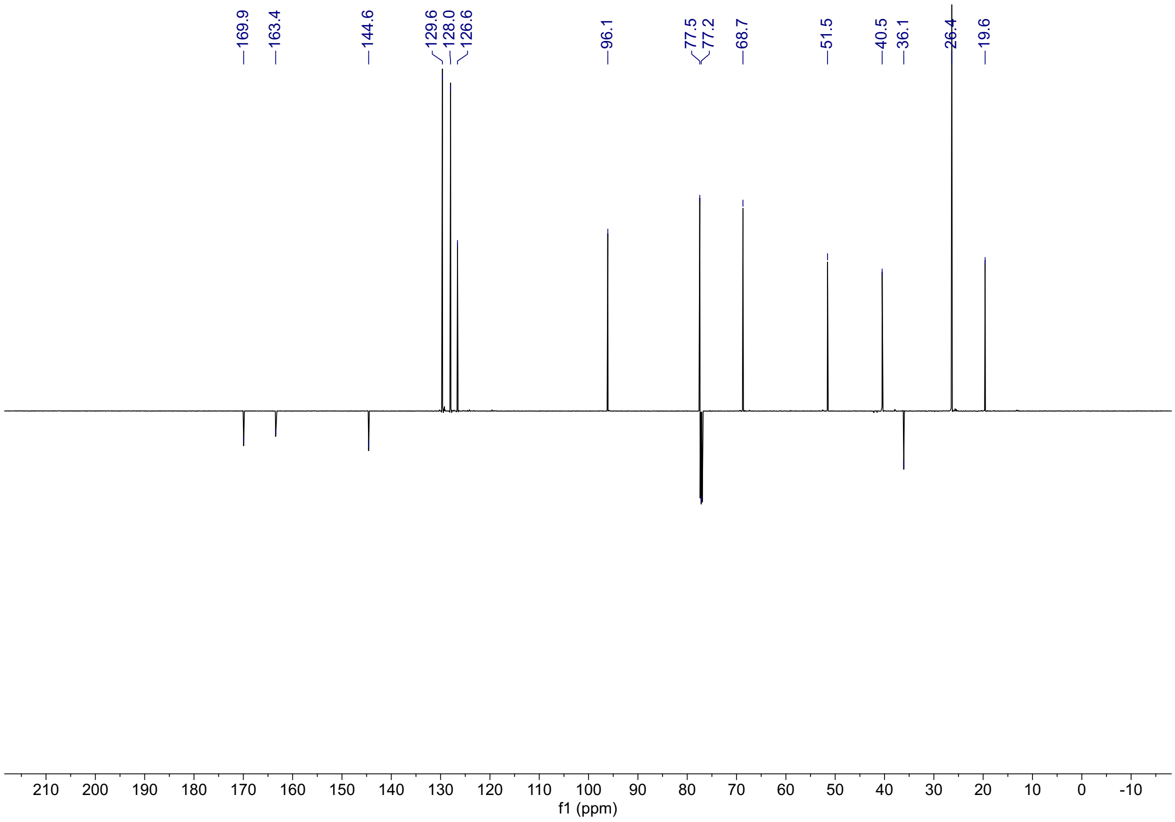

169.9
163.4
144.6
129.6
128.0
126.6
96.1
77.5
77.2
68.7
51.5
40.5
36.1
26.4
19.6
210
200
190
180
170
160
150
140
130
120
110
100
90
80
70
60
50
40
30
20
10
0
-10
f1 (ppm)

Methyl (2*R*,4*S*,5*R*)-3-((4-bromophenyl)(methyl)carbamoyl)-2-(*tert*-butyl)-5-methyl-oxazolidine-4-carboxylate (21b)

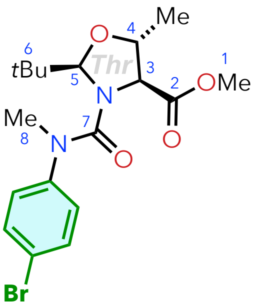

21b

Following **GP3**, *N*-chloroformyloxazolidine **17** (500-mg scale) was used as the carbamoyl chloride and 4-bromo-*N*-methylaniline (0.36 mL, 2.84 mmol, 1.5 equiv.) as the aniline in DCE. The title compound **21b** was obtained as a brown oil (508 mg, 1.23 mmol, 65%) after purification by silica gel column chromatography (PE/EA, gradient elution).

Formula: $C_{18}H_{25}BrN_2O_4$, **MW:** 413.31 g/mol. **TLC:** R_f = 0.54 (PE/EA 2:1), $KMnO_4$ stain. **^{1}H NMR** (600 MHz, $CDCl_3$): δ [ppm] = 7.45 (d, *J* 8.5 Hz, 2H, Ar), 7.03 (d, *J* 8.5 Hz, 2H, Ar), 5.49 (s, 1H, H-5), 4.28 (q, *J* 6.4 Hz, 1H, H-4), 3.92 (s, 1H, H-3), 3.30 (s, 3H, H-1, OMe), 3.18 (s, 3H, H-8, NMe), 1.23 (d, *J* 6.4 Hz, 1H, Me), 0.98 (s, 9H, *t*Bu). **^{13}C NMR** (150 MHz, $CDCl_3$): δ [ppm] = 170.0 (s, C-2, ester), 163.1 (s, C-7, urea), 143.6 (s, Ar), 132.8 (d, 2Ar), 129.6 (d, 2Ar), 120.2 (d, Ar), 96.2 (d, C-5), 77.5 (d, C-4) 68.6 (d, C-3), 51.8 (q, C-1, OMe), 40.3 (q, C-8, NMe), 36.1 (s, C-6, *t*Bu), 26.4 (q, *t*Bu, 3Me), 19.6 (q, Me). **FT-IR (ATR):** $\tilde{\nu}$ [cm^{-1}] = 2981 (vs), 2889 (m), 1462 (w), 1473 (w), 1382 (m), 1251 (m), 1152 (m), 1072 (m), 954 (m), 815 (w). **HR-MS:** (ESI) = *m*/*z* calcd. for: $C_{18}H_{26}{}^{79}BrN_2O_4$ $[M+H]^+$ 413.1076 u, found: 413.1052 u. **$[a]_\lambda^T$:** (c = 1.00 g/100 mL, $CHCl_3$) = $[a]_D^{20}$: –16.00°.

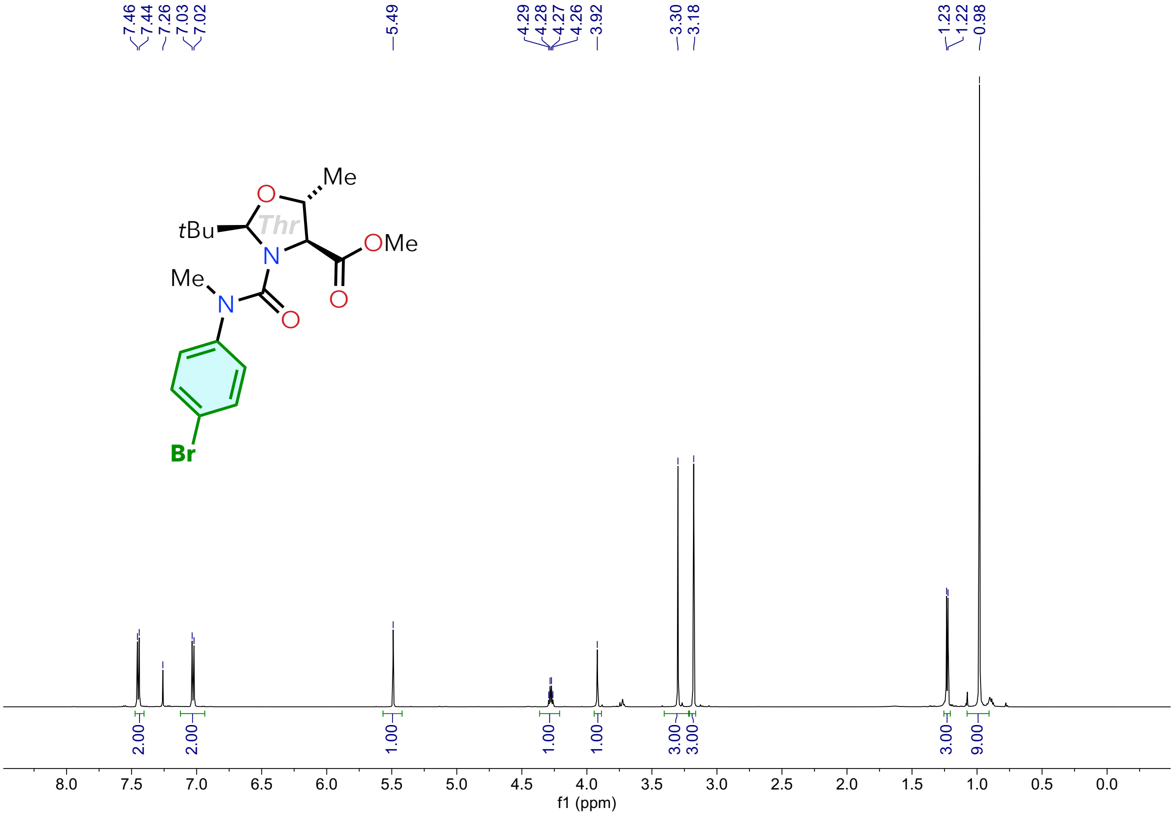

7.46
7.44
7.26
7.03
7.02
5.49
4.29
4.28
4.27
4.26
3.92
3.30
3.18
1.23
1.22
0.98
Me
O
tBu
Thr
N
OMe
Me
N
O
O
Br
2.00
2.00
1.00
1.00
1.00
3.00
3.00
3.00
9.00
8.0
7.5
7.0
6.5
6.0
5.5
5.0
4.5
4.0
3.5
3.0
2.5
2.0
1.5
1.0
0.5
0.0
f1 (ppm)

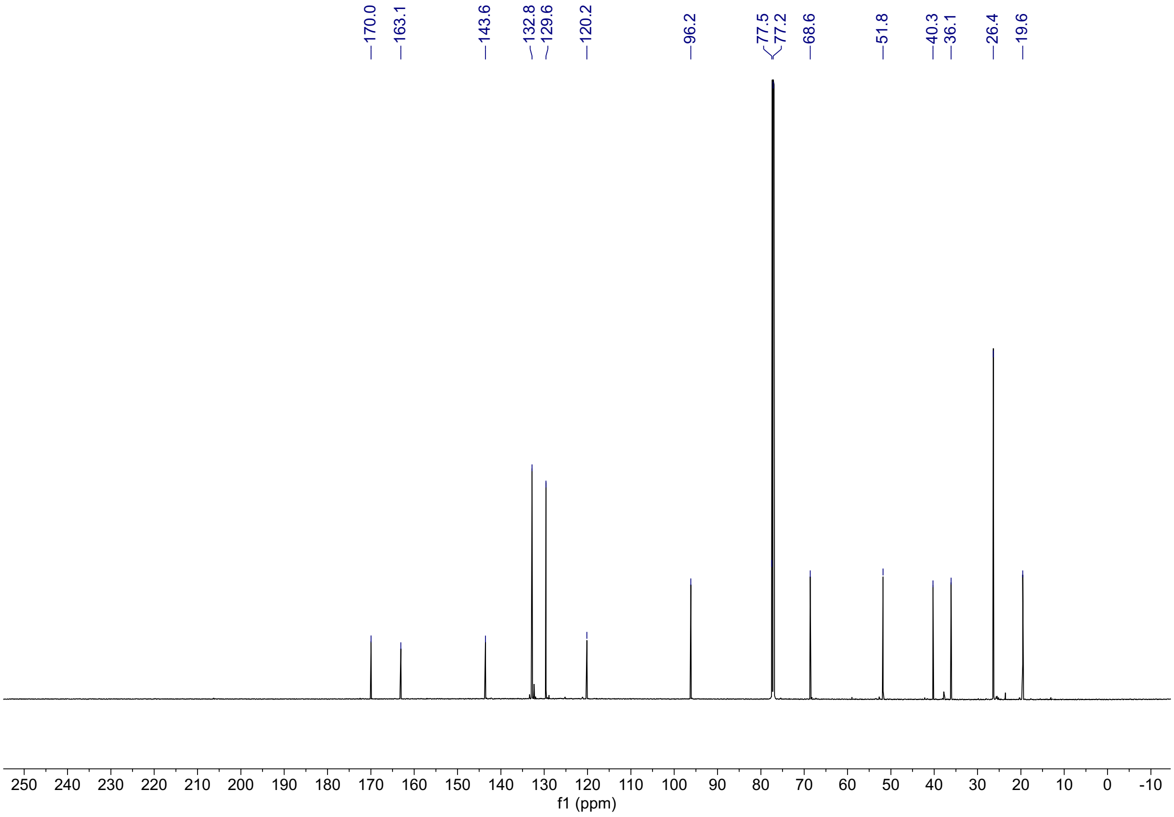

170.0
163.1
143.6
132.8
129.6
120.2
96.2
77.5
77.2
68.6
51.8
40.3
36.1
26.4
19.6
250
240
230
220
210
200
190
180
170
160
150
140
130
120
110
100
90
80
70
60
50
40
30
20
10
0
-10
f1 (ppm)

Methyl (2*R*,4*S*,5*R*)-2-(*tert*-butyl)-3-((3-methoxyphenyl)(methyl)carbamoyl)-5-methyl-oxazolidine-4-carboxylate (21c)

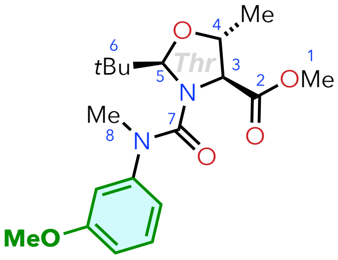

21c

Following **GP3**, *N*-chloroformyloxazolidine **17** (250-mg scale) was used as the carbamoyl chloride and 3-methoxy-*N*-methylaniline (0.19 mL, 1.42 mmol, 1.5 equiv.) as the aniline in DCE. The title compound **21c** was obtained as a red oil (96 mg, 0.26 mmol, 28%) after purification by silica gel column chromatography (PE/EA, gradient elution).

Formula: $C_{19}H_{28}N_2O_5$, **MW:** 364.44 g/mol. **TLC:** R_f = 0.39 (PE/EA 2:1), $KMnO_4$ stain. **^{1}H NMR** (600 MHz, $CDCl_3$): δ [ppm] = 7.23 (t, *J* 8.1 Hz, 1H, Ar), 6.74 (td, *J* 7.8, 7.3, 2.1 Hz, 2H, Ar), 6.69 (t, *J* 2.2 Hz, 1H, Ar), 5.50 (s, 1H, H-5), 4.26 (q, *J* 6.4 Hz, 1H, H-4), 3.99 (s, 1H, H-3), 3.80 (s, 3H, H-1, OMe), 3.27 (s, 3H, H-8, NMe), 3.19 (s, 3H, ArOMe), 1.23 (d, *J* 6.4 Hz, 1H, Me), 1.00 (s, 9H, H-6, *t*Bu). **^{13}C NMR** (150 MHz, $CDCl_3$): δ [ppm] = 170.0 (s, C-2, ester), 163.3 (s, C-7, urea), 160.5 (s, Ar), 145.6 (s, Ar), 130.3 (d, Ar), 119.9 (d, Ar), 113.3 (d, Ar), 112.8 (d, Ar), 96.2 (d, C-5), 77.5 (d, C-4) 68.7 (d, C-3), 55.4 (q, Ar, OMe), 51.5 (q, C-1, OMe), 40.4 (q, C-8, NMe), 36.1 (s, C-6, *t*Bu), 26.4 (q, *t*Bu, 3Me), 19.6 (q, Me). **FT-IR (ATR):** $\tilde{\nu}$ [cm^{-1}] = 2980 (vs), 2889 (m), 1768 (w), 1726 (w), 1664 (m), 1599 (w), 1489 (m), 1381 (m), 1235 (m), 1153 (s), 1071 (m), 954 (m), 787 (w), 698 (w). **HR-MS:** (ESI) = *m*/*z* calcd. for: $C_{19}H_{29}N_2O_5$ $[M+H]^+$ 365.2076 u, found: 365.2053 u. **$[a]_\lambda^T$:** (c = 1.00 g/100 mL, $CHCl_3$) = $[a]_D^{20}$: –8.40°.

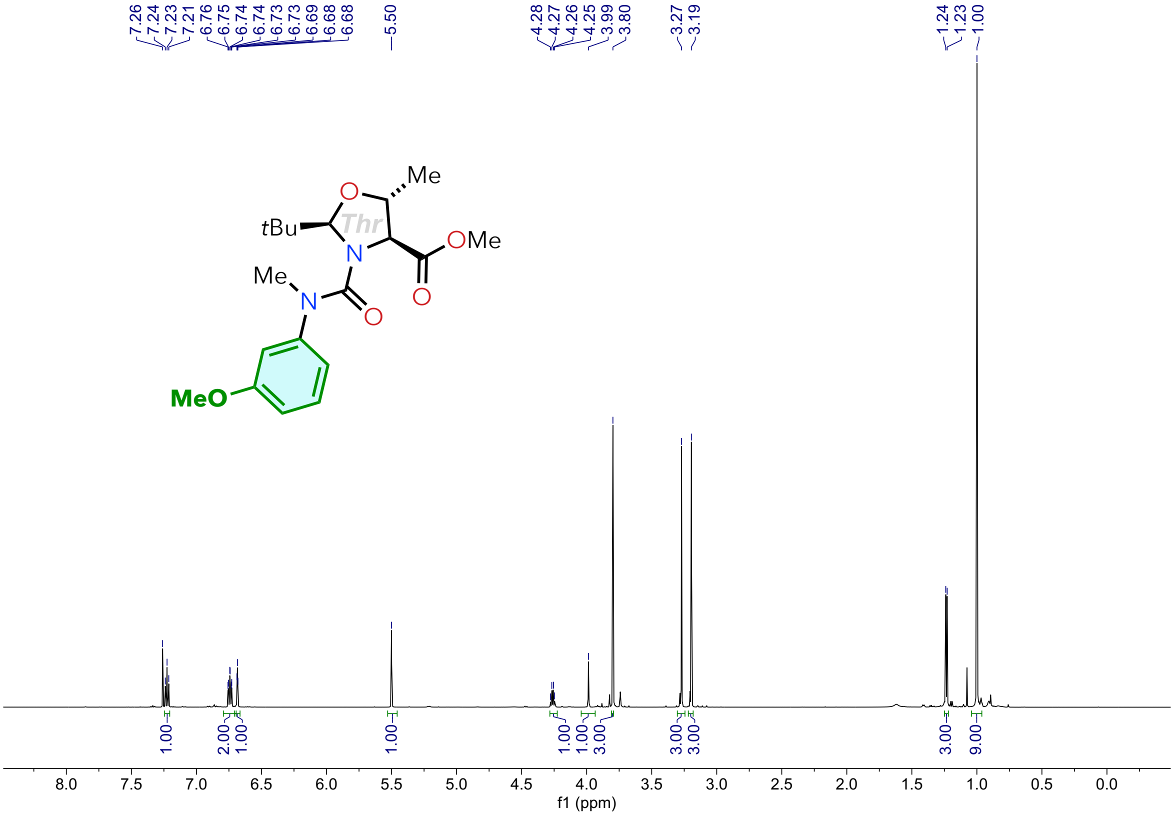
7.26
7.24
7.23
7.21
6.76
6.75
6.74
6.74
6.73
6.73
6.69
6.68
6.68
5.50
4.28
4.27
4.26
4.25
3.99
3.80
3.27
3.19
1.24
1.23
1.00
Me
O
tBu
Thr
N
OMe
O
Me
N
O
MeO
1.00
2.00
1.00
1.00
1.00
1.00
3.00
3.00
3.00
3.00
9.00
8.0 7.5 7.0 6.5 6.0 5.5 5.0 4.5 4.0 3.5 3.0 2.5 2.0 1.5 1.0 0.5 0.0
f1 (ppm)

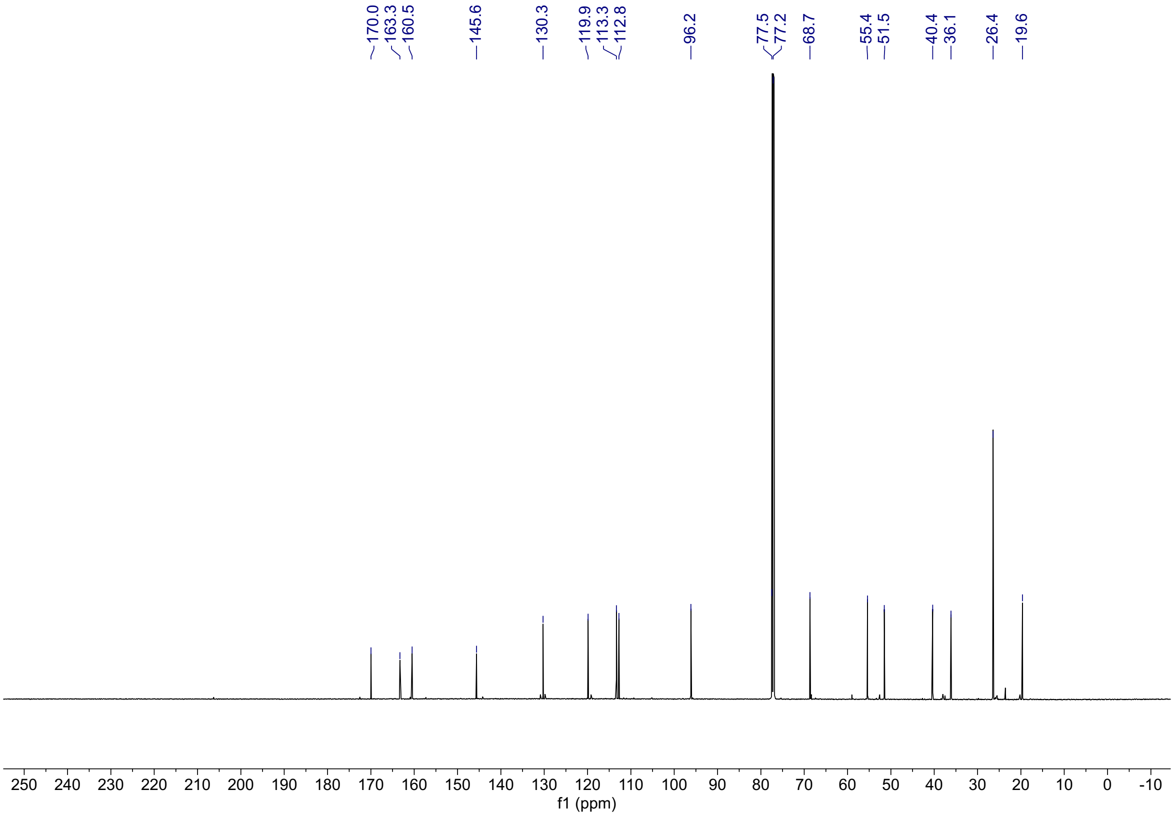
170.0
163.3
160.5
145.6
130.3
119.9
113.3
112.8
96.2
77.5
77.2
68.7
55.4
51.5
40.4
36.1
26.4
19.6
250 240 230 220 210 200 190 180 170 160 150 140 130 120 110 100 90 80 70 60 50 40 30 20 10 0 -10
f1 (ppm)

Methyl (2*R*,4*S*,5*S*)-2-(*tert*-butyl)-5-methyl-3-(methyl(phenyl)carbamoyl)oxazolidine-4-carboxylate (22a)

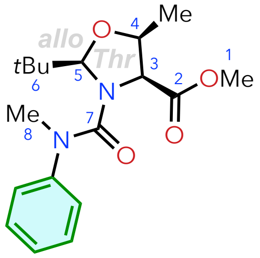

22a

Following **GP3**, to a solution of *N*-chloroformyloxazolidine **18** (500 mg, 0.95 mmol, 1.0 equiv.) in DCE (1.2 mL, 0.8 M) was added triethylamine (0.27 mL, 1.90 mmol, 2.0 equiv.) and *N*-methylaniline (0.15 mL, 1.42 mmol, 1.5 equiv.). The reaction mixture was heated to reflux (85 °C) for 18 h. The product **22a** was obtained as a beige solid (580 mg, 1.73 mmol, 91%) after purification by silica gel column chromatography (PE/EA, gradient elution).

Formula: $C_{18}H_{26}N_2O_4$, **MW:** 334.42 g/mol. **TLC:** R_f = 0.34 (PE/EA 2:1), $KMnO_4$ stain. ^{1}**H NMR** (600 MHz, $CDCl_3$): δ [ppm] = 7.31 (t, *J* 7.8 Hz, 2H, Ar), 7.18 (dd, *J* 17.4, 7.7 Hz, 3H, Ar), 5.36 (s, 1H, H-5), 4.12 (d, *J* 6.5 Hz, 1H, H-4), 4.06 (s, 1H, H-3), 3.21 (s, 3H, H-1, OMe), 3.19 (s, 3H, H-8, NMe), 1.06 (d, *J* 6.3 Hz, 3H, Me), 1.04 (s, 9H, *t*Bu). ^{13}**C NMR** (150 MHz, $CDCl_3$): δ [ppm] = 168.7 (s, C-2, ester), 162.8 (s, C-7, urea), 144.3 (s, Ar), 129.5 (d, 2Ar), 127.8 (d, 2Ar), 126.4 (d, Ar), 97.4 (d, C-5), 75.4 (d, C-4), 67.2 (d, C-3), 50.9 (q, C-1, OMe), 40.1 (q, C-8, NMe), 36.1 (s, C-6, *t*Bu), 26.7 (q, *t*Bu, 3Me), 15.0 (q, Me). **FT-IR (ATR):** $\tilde{\nu}$ [cm^{-1}] = 2957 (br w), 1757 (m), 1664 (s), 1594 (w), 1494 (m), 1457 (w), 1444 (w), 1363 (m), 1336 (m), 1301 (m), 1197 (m), 1152 (vs), 1076 (m), 998 (m), 924 (w), 904 (w), 876 (w), 792 (w), 767 (m), 693 (s), 654 (w), 590 (m), 546 (w), 530 (w). **HR-MS:** (ESI) = *m/z* calcd. for: $C_{18}H_{27}N_2O_4$ $[M+H]^+$ 335.1971 u, found: 335.1949 u. $\mathbf{[a]_\lambda^T}$**:** (c = 1.00 g/100 mL, $CHCl_3$) = $[a]_D^{20}$: –17.20°.

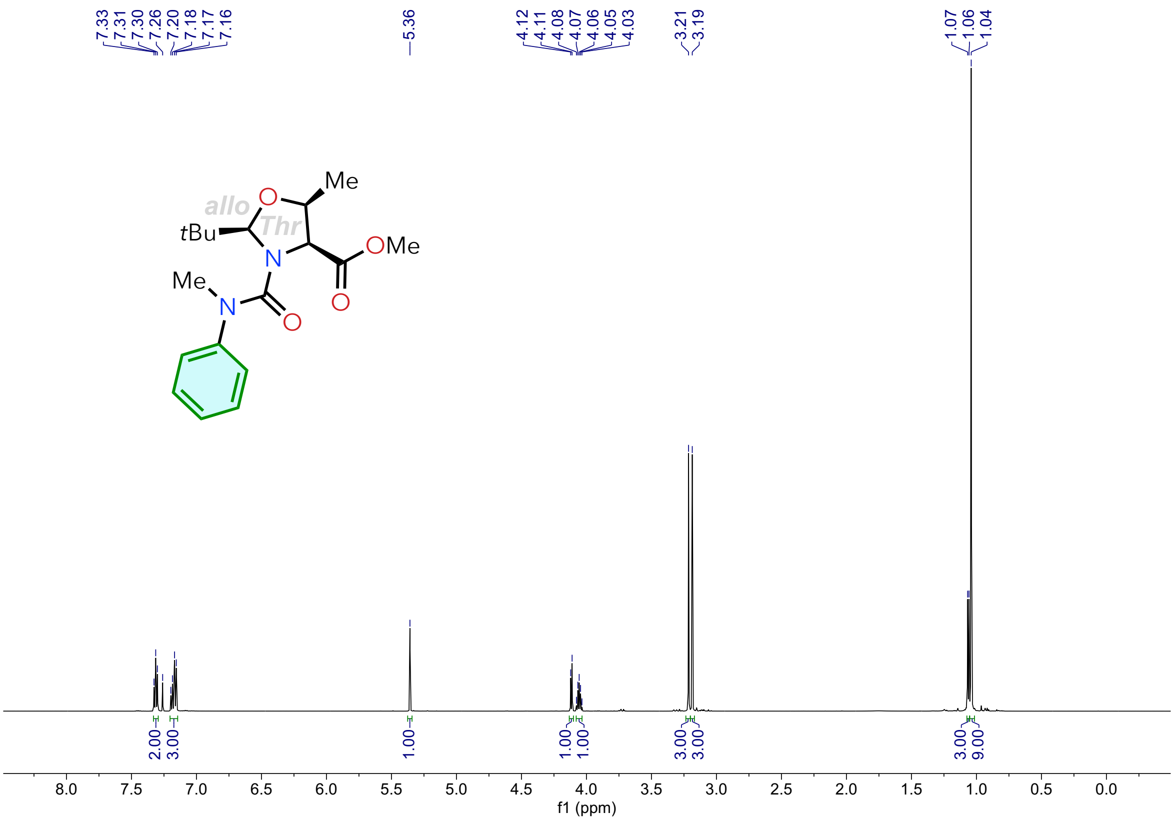
7.33
7.31
7.30
7.26
7.20
7.18
7.17
7.16
5.36
4.12
4.11
4.08
4.07
4.06
4.05
4.03
3.21
3.19
1.07
1.06
1.04
allo Thr
Me
tBu
OMe
2.00
3.00
1.00
1.00
1.00
3.00
3.00
3.00
9.00
f1 (ppm)

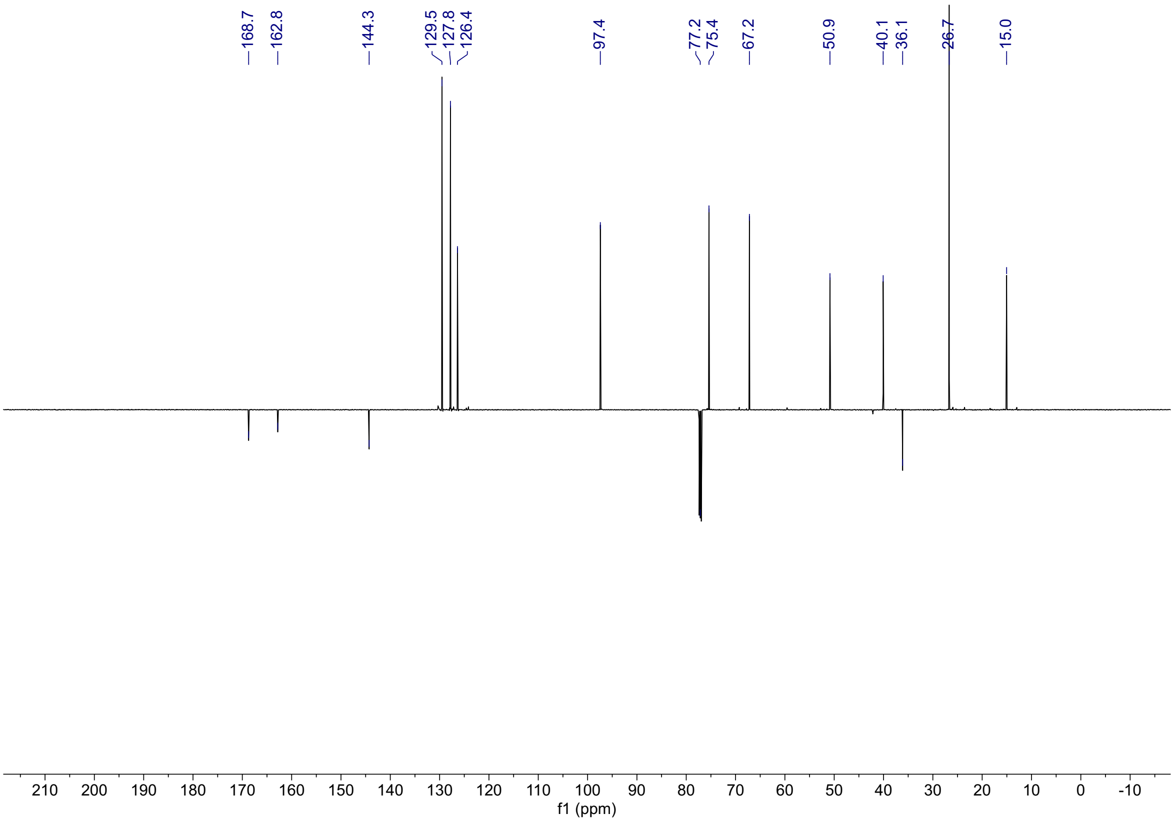
168.7
162.8
144.3
129.5
127.8
126.4
97.4
77.2
75.4
67.2
50.9
40.1
36.1
26.7
15.0
f1 (ppm)

Methyl (2*R*,4*S*,5*S*)-3-((4-bromophenyl)(methyl)carbamoyl)-2-(*tert*-butyl)-5-methyl-oxazolidine-4-carboxylate (22b)

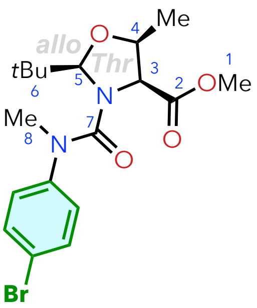

22b

Following **GP3**, *N*-chloroformyloxazolidine **18** (250-mg scale) was used as the carbamoyl chloride and 4-bromo-*N*-methylaniline (0.18 mL, 1.43 mmol, 1.5 equiv.) as the aniline in DCE. The title compound **22b** was obtained as a red solid (272 mg, 0.66 mmol, 69%) after purification by silica gel column chromatography (PE/EA, gradient elution).

Formula: $C_{18}H_{25}BrN_2O_4$, **MW:** 413.31 g/mol, **m.p.:** 101 – 104 °C. **TLC:** R_f = 0.35 (PE/EA 2:1), $KMnO_4$ stain. **^{1}H NMR** (600 MHz, $CDCl_3$): δ [ppm] = 7.43 (d, *J* 8.6 Hz, 2H, Ar), 7.04 (d, *J* 8.6 Hz, 2H, Ar), 5.34 (s, 1H, H-5), 4.05 (q, *J* 6.1, 5.3 Hz, 2H, H-3, H-4), 3.27 (s, 3H, H-1, OMe), 3.20 (s, 3H, H-8, NMe), 1.07 (d, *J* 6.3 Hz, 3H, Me), 1.03 (s, 9H, *t*Bu). **^{13}C NMR** (150 MHz, $CDCl_3$): δ [ppm] = 168.7 (s, C-2, ester), 162.4 (s, C-7, urea), 143.3 (s, Ar), 132.7 (d, 2Ar), 129.4 (d, 2Ar), 119.9 (s, Ar), 97.5 (d, C-5), 75.5 (d, C-4), 67.1 (d, C-3), 51.2 (q, C-1, OMe), 39.9 (q, C-8, NMe), 36.1 (s, C-6, *t*Bu), 26.7 (q, *t*Bu, 3Me), 15.0 (q, Me). **FT-IR (ATR):** $\tilde{\nu}$ [cm^{-1}] = 2981 (br w), 1753 (m), 1664 (s), 1586 (w), 1489 (m), 1364 (m), 1333 (s), 1311 (m), 1293 (m), 1245 (w), 1195 (m), 1152 (vs), 1120 (m), 1072 (m), 995 (m), 922 (w), 903 (w), 875 (w), 834 (w), 786 (w), 699 (w), 662 (w), 588 (m), 527 (m). **HR-MS:** (ESI) = *m*/*z* calcd. for: $C_{18}H_{26}{}^{79}BrN_2O_4$ $[M+H]^+$ 413.1076 u, found: 413.1063 u. **$[a]_\lambda^T$:** (c = 1.00 g/100 mL, $CHCl_3$) = $[a]_D^{20}$: –15.60°.

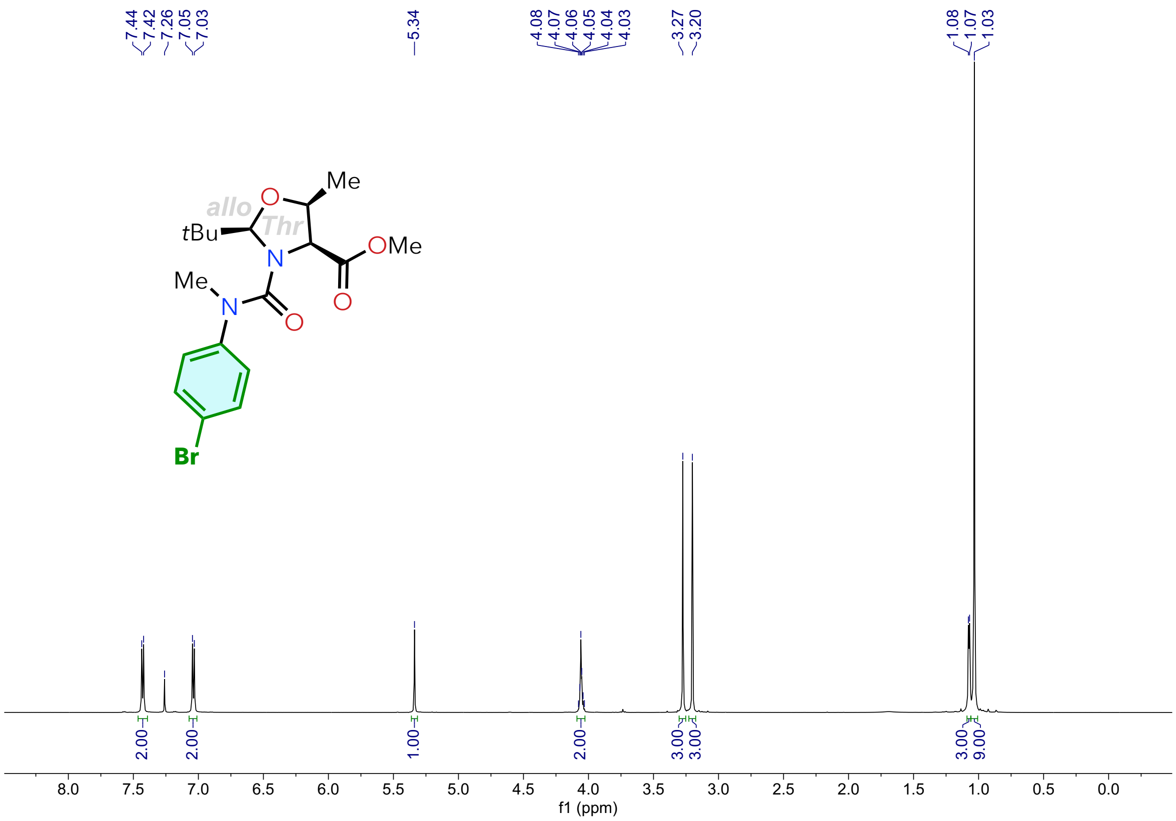
7.44
7.42
7.26
7.05
7.03
5.34
4.08
4.07
4.06
4.05
4.04
4.03
3.27
3.20
1.08
1.07
1.03
Me
O
allo
Thr
tBu
N
OMe
Me
N
O
O
Br
2.00
2.00
1.00
2.00
3.00
3.00
3.00
9.00
8.0 7.5 7.0 6.5 6.0 5.5 5.0 4.5 4.0 3.5 3.0 2.5 2.0 1.5 1.0 0.5 0.0
f1 (ppm)

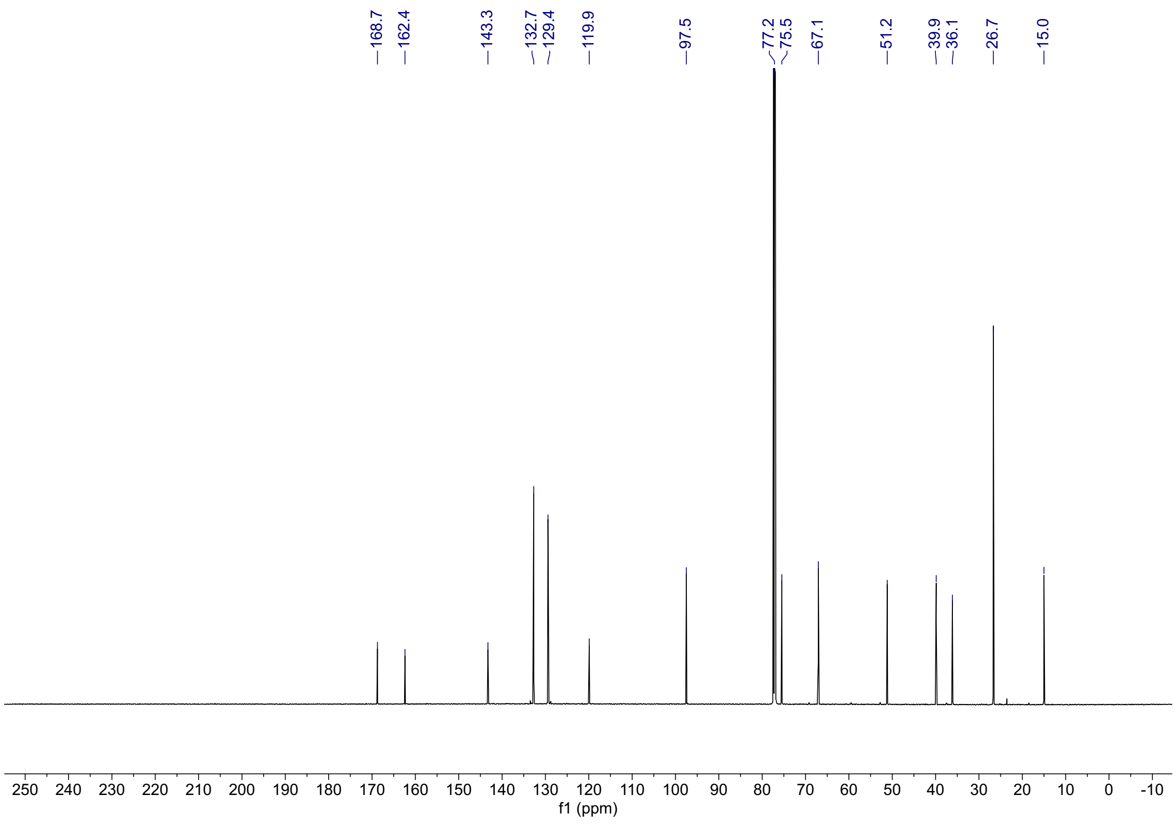
168.7
162.4
143.3
132.7
129.4
119.9
97.5
77.2
75.5
67.1
51.2
39.9
36.1
26.7
15.0
250 240 230 220 210 200 190 180 170 160 150 140 130 120 110 100 90 80 70 60 50 40 30 20 10 0 -10
f1 (ppm)

Methyl (2*R*,4*S*,5*S*)-2-(*tert*-butyl)-5-methyl-3-(methyl(4-(trifluoromethyl)phenyl)-carbamoyl)oxazolidine-4-carboxylate (22c)

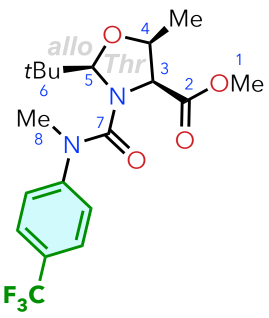

22c

Following **GP3**, *N*-chloroformyloxazolidine **18** (250-mg scale) was used as the carbamoyl chloride and *N*-methyl-4-(trifluoromethyl)aniline (0.20 mL, 1.42 mmol, 1.5 equiv.) as the aniline in DCE. The title compound **22c** was obtained as a white solid (163 mg, 0.41 mmol, 43%) after purification by silica gel column chromatography (PE/EA, gradient elution).

Formula: $C_{19}H_{25}F_3N_2O_4$, **MW:** 402.41 g/mol, **m.p.:** 107 – 110 °C. **TLC:** R_f = 0.52 (PE/EA 2:1), $KMnO_4$ stain. ^{1}**H NMR** (600 MHz, $CDCl_3$): δ [ppm] = 7.58 (d, *J* 8.5 Hz, 2H, Ar), 7.30 (d, *J* 8.4 Hz, 3H, Ar), 5.35 (s, 1H, H-5), 4.07 – 4.04 (m, 2H, H-3, H-4), 3.25 (s, 3H, H-1, OMe), 3.19 (s, 3H, H-8, NMe), 1.07 (d, *J* 5.4 Hz, 3H, Me), 1.05 (s, 9H, *t*Bu). ^{13}**C NMR** (150 MHz, $CDCl_3$): δ [ppm] = 168.7 (s, C-2, ester), 162.3 (s, C-7, urea), 147.5 (s, Ar), 128.1 (s, *q* 32.8 Hz, Ar), 127.5 (d, 2Ar), 126.7 (d, *q J* 3.7 Hz, 2Ar), 123.9 (s, *q* 271.5 Hz, Ar), 97.6 (d, C-5), 75.5 (d, C-4), 67.0 (d, C-3), 50.9 (q, C-1, OMe), 39.7 (q, C-8, NMe), 36.2 (s, C-6, *t*Bu), 26.7 (q, *t*Bu, 3Me), 14.9 (q, Me). ^{19}**F NMR** (565 MHz, $CDCl_3$, C_6F_6 ref.): δ [ppm] = –65.61 (s, 3F). **FT-IR (ATR):** $\tilde{\nu}$ [cm^{-1}] = 2980 (br w), 1754 (m), 1665 (m), 1613 (w), 1462 (w), 1364 (w), 1321 (vs), 1199 (w), 1159 (s), 1122 (s), 1067 (s), 1016 (m), 997 (w), 926 (w), 877 (w), 845 (m), 790 (m), 662 (w), 610 (w). **HR-MS:** (ESI) = *m*/*z* calcd. for: $C_{19}H_{26}F_3N_2O_4$ $[M+H]^+$ 403.1845 u, found: 403.1837 u. **$[a]_\lambda^T$:** (c = 1.00 g/100 mL, $CHCl_3$) = $[a]_D^{20}$: –16.40°.

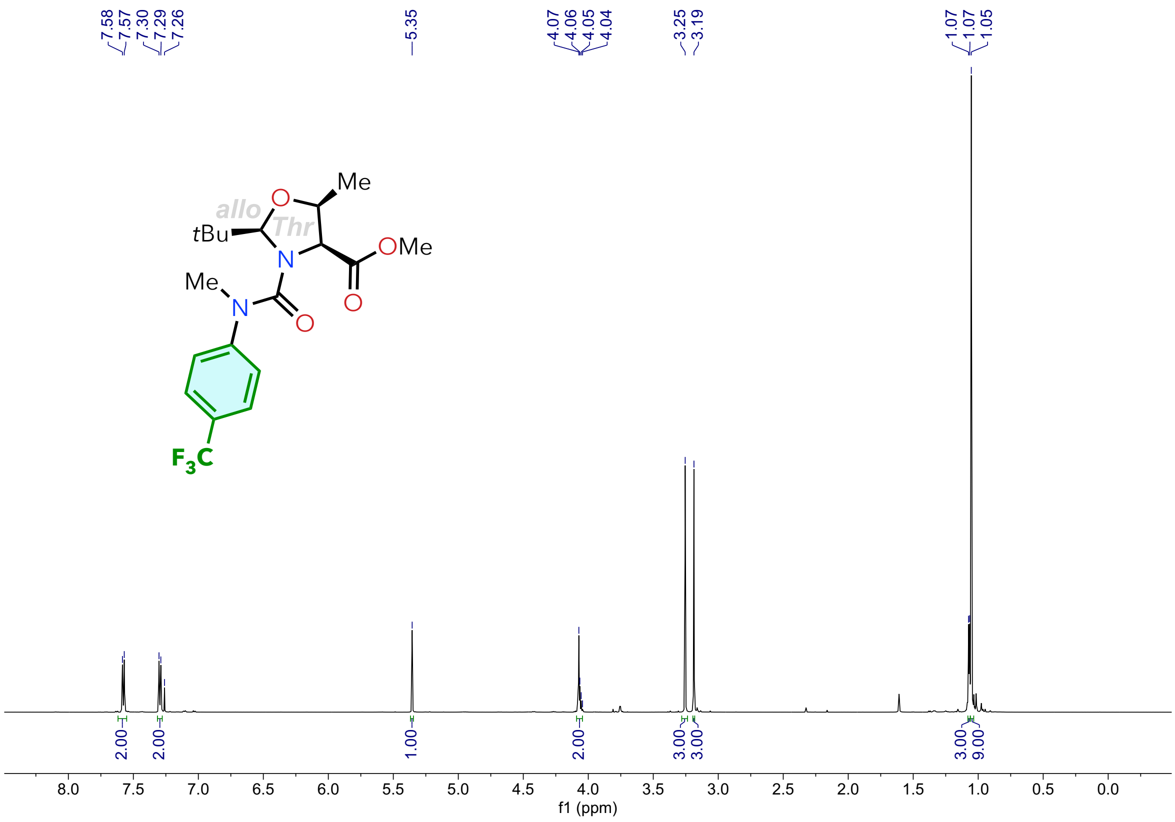

allo Thr
O
Me
tBu
N
OMe
O
Me
N
O
F3C
7.58
7.57
7.30
7.29
7.26
5.35
4.07
4.06
4.05
4.04
3.25
3.19
1.07
1.07
1.05
2.00
2.00
1.00
2.00
3.00
3.00
3.00
9.00
8.0 7.5 7.0 6.5 6.0 5.5 5.0 4.5 4.0 3.5 3.0 2.5 2.0 1.5 1.0 0.5 0.0
f1 (ppm)

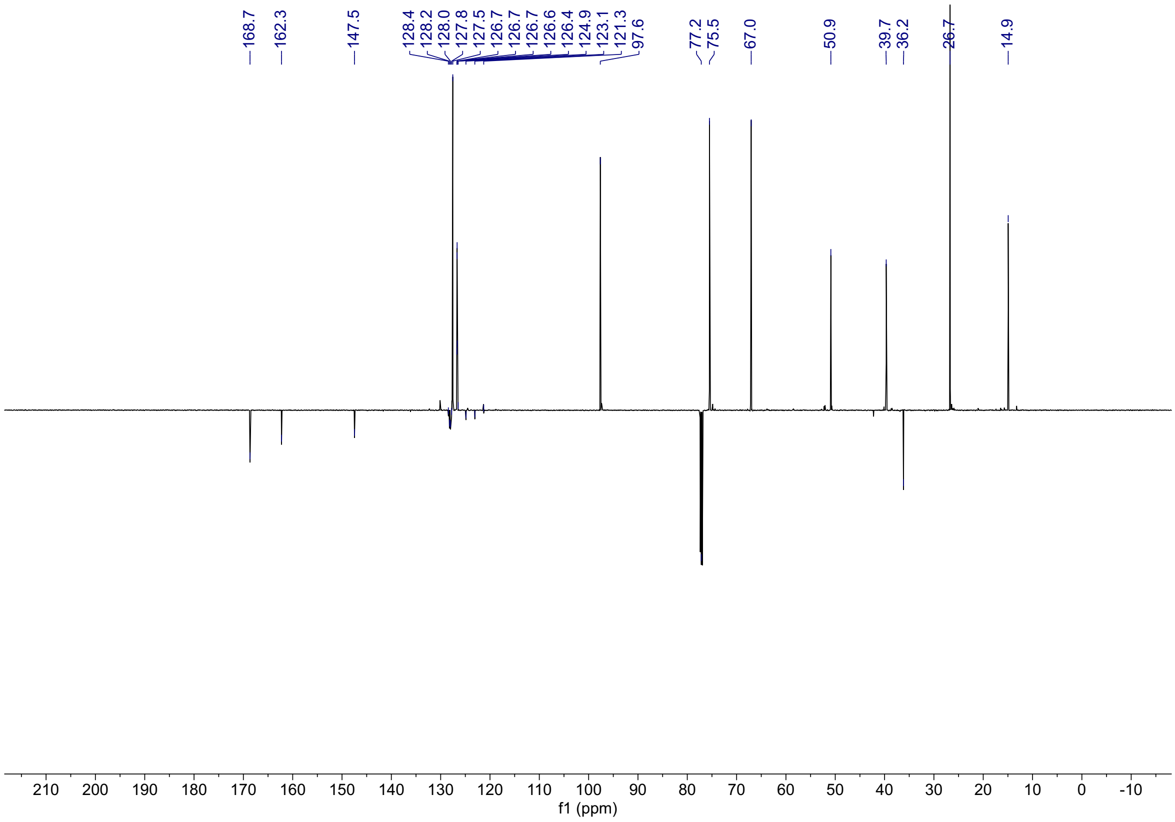

168.7
162.3
147.5
128.4
128.2
128.0
127.8
127.5
126.7
126.7
126.6
126.4
124.9
123.1
121.3
97.6
77.2
75.5
67.0
50.9
39.7
36.2
26.7
14.9
210 200 190 180 170 160 150 140 130 120 110 100 90 80 70 60 50 40 30 20 10 0 -10
f1 (ppm)

Methyl (2*R*,4*S*,5*S*)-2-(*tert*-butyl)-3-((3-methoxyphenyl)(methyl)carbamoyl)-5-methyl-oxazolidine-4-carboxylate (22d)

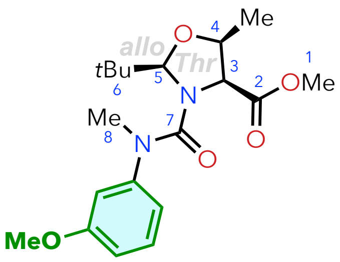

22d

Following **GP3**, *N*-chloroformyloxazolidine **18** (250-mg scale) was used as the carbamoyl chloride and 3-methoxy-*N*-methylaniline (0.19 mL, 1.42 mmol, 1.5 equiv.) as the aniline in DCE. The title compound **22d** was obtained as a red solid (255 mg, 0.70 mmol, 74%) after purification by silica gel column chromatography (PE/EA, gradient elution).

Formula: $C_{19}H_{28}N_2O_5$, **MW:** 364.44 g/mol. **TLC:** R_f = 0.40 (PE/EA 2:1), $KMnO_4$ stain. **^{1}H NMR** (500 MHz, $CDCl_3$): δ [ppm] = 7.22 (t, *J* 8.1 Hz, 1H, Ar), 6.78 – 6.65 (m, 3H, Ar), 5.36 (s, 1H, H-5), 4.12 (d, *J* 6.6 Hz, 1H, H-3), 4.05 (p, *J* 6.3 Hz, 1H, H-4), 3.80 (s, 3H, Ar, MeO) 3.24 (s, 3H, H-1, OMe), 3.22 (s, 3H, H-8, NMe), 1.07 (d, *J* 6.2 Hz, 3H, Me), 1.05 (s, 9H, *t*Bu). **^{13}C NMR** (125 MHz, $CDCl_3$): δ [ppm] = 168.8 (s, C-2, ester), 162.6 (s, C-7, urea), 160.3 (s, Ar), 145.3 (s, Ar), 130.2 (d, Ar), 119.7 (d, Ar), 113.1 (d, Ar), 112.6 (d, Ar), 97.5 (d, C-5), 75.4 (d, C-4), 67.1 (d, C-3), 55.3 (q, Ar, OMe), 50.8 (q, C-1, OMe), 40.0 (q, C-8, NMe), 36.1 (s, C-6, *t*Bu), 26.7 (q, *t*Bu, 3Me), 15.1 (q, Me). **FT-IR (ATR):** $\tilde{\nu}$ [cm^{-1}] = 2954 (br w), 1763 (m), 1731 (w), 1660 (s), 1599 (m), 1489 (w), 1439 (w), 1364 (m), 1333 (m), 1283 (w), 1152 (vs), 1120 (m), 1074 (w), 1041 (w), 998 (w), 955 (w), 926 (w), 903 (w), 876 (w), 781 (w), 699 (m). **HR-MS:** (ESI) = *m*/*z* calcd. for: $C_{19}H_{29}N_2O_5$ $[M+H]^+$ 365.2076 u, found: 365.2066 u. **$[a]_\lambda^T$:** (c = 1.00 g/100 mL, $CHCl_3$) = $[a]_D^{20}$: –10.80°.

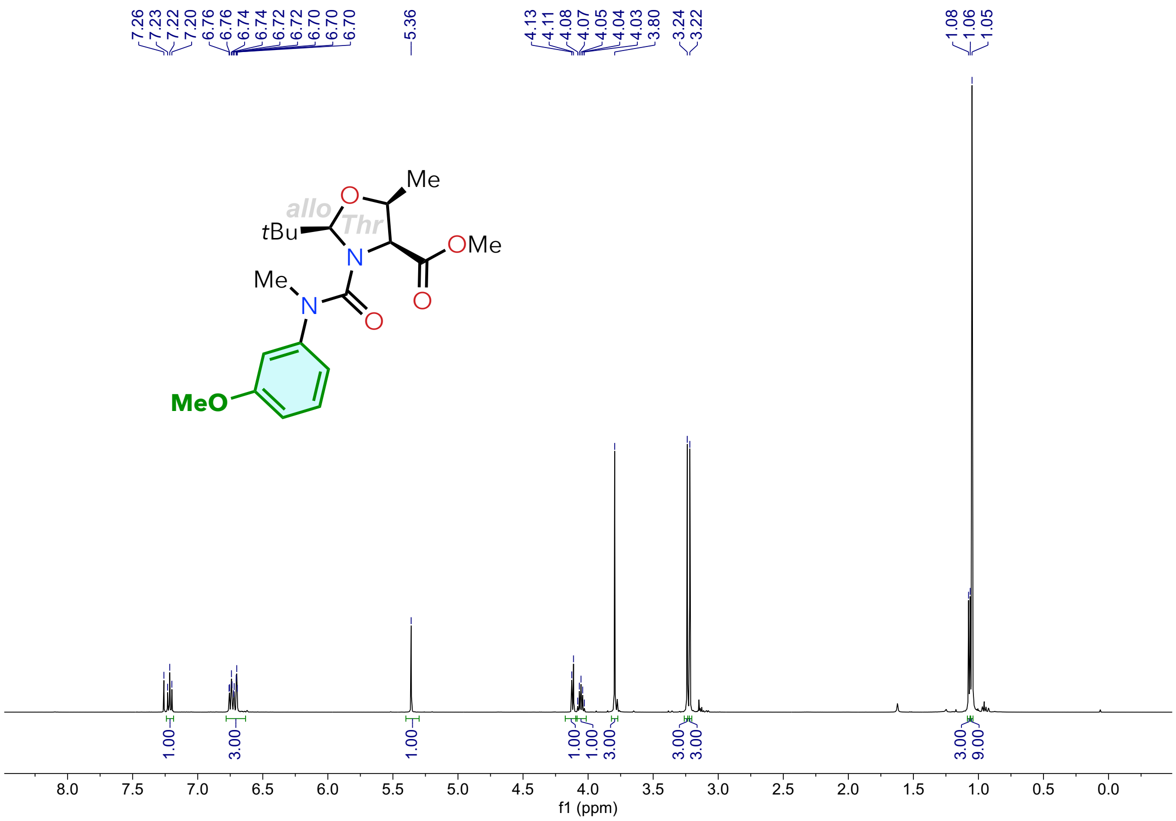

7.26
7.23
7.22
7.20
6.76
6.76
6.74
6.74
6.72
6.72
6.70
6.70
6.70
5.36
4.13
4.11
4.08
4.07
4.05
4.04
4.03
3.80
3.24
3.22
1.08
1.06
1.05
Me
O
allo
Thr
tBu
N
OMe
O
Me
N
O
MeO
1.00
3.00
1.00
1.00
1.00
3.00
3.00
3.00
3.00
9.00
f1 (ppm)

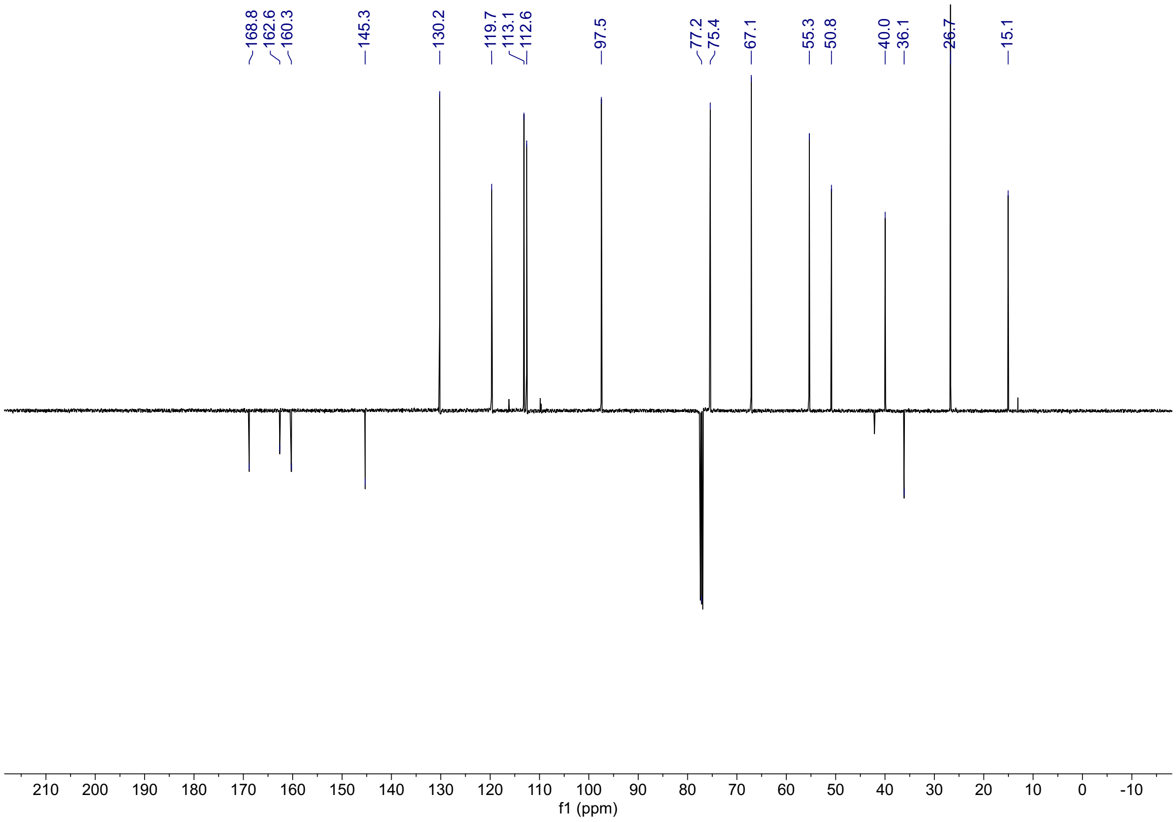

168.8
162.6
160.3
145.3
130.2
119.7
113.1
112.6
97.5
77.2
75.4
67.1
55.3
50.8
40.0
36.1
26.7
15.1
f1 (ppm)

Methyl (2*R*,4*S*,5*S*)-2-(*tert*-butyl)-5-methyl-3-(methyl(*p*-tolyl)carbamoyl)oxazolidine-4-carboxylate (22e)

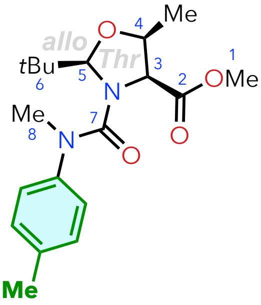

22e

Following **GP3**, *N*-chloroformyloxazolidine **18** (250-mg scale) was used as the carbamoyl chloride and *N*-methyl-*p*-toluidine (0.18 mL, 1.42 mmol, 1.5 equiv.) as the aniline in DCE. The title compound **22e** was obtained as a white solid (317 mg, 0.91 mmol, 96%) after purification by silica gel column chromatography (PE/EA, gradient elution).

Formula: $C_{19}H_{28}N_2O_4$, **MW:** 348.44 g/mol, **m.p.:** 108 – 111 °C. **TLC:** R_f = 0.51 (PE/EA 2:1), $KMnO_4$ stain. **^{1}H NMR** (600 MHz, $CDCl_3$): δ [ppm] = 7.11 (d, *J* 8.2 Hz, 2H, Ar), 7.03 (d, *J* 8.3 Hz, 3H, Ar), 5.35 (s, 1H, H-5), 4.10 (d, *J* 6.6 Hz, 1H, H-3), 4.05 (p, *J* 6.3 Hz, 1H, H-4), 3.18 (d, *J* 3.7 Hz, 3H, H-1, OMe, H-8, NMe), 2.32 (s, 3H, Ar, Me), 1.06 (d, *J* 6.2 Hz, 3H, Me), 1.03 (s, 9H, *t*Bu). **^{13}C NMR** (150 MHz, $CDCl_3$): δ [ppm] = 168.8 (s, C-2, ester), 162.9 (s, C-7, urea), 141.6 (s, Ar), 136.1 (s, Ar), 130.1 (d, 2Ar), 127.7 (d, 2Ar), 97.4 (d, C-5), 75.4 (d, C-4), 67.2 (d, C-3), 50.7 (q, C-1, OMe), 40.1 (q, C-8, NMe), 36.1 (s, C-6, *t*Bu), 26.7 (q, *t*Bu, 3Me), 21.0 (q, Ar, Me), 15.0 (q, Me). **FT-IR (ATR):** $\tilde{\nu}$ [cm^{-1}] = 2979 (br w), 1757 (m), 1663 (vs), 1512 (m), 1442 (w), 1417 (m), 1364 (m), 1335 (s), 1301 (m), 1198 (s), 1159 (s), 1119 (m), 1080 (m), 1061 (w), 1002 (m), 924 (w), 878 (w), 832 (m), 789 (m), 721 (w), 674 (w), 591 (m), 542 (w). **HR-MS:** (ESI) = *m*/*z* calcd. for: $C_{19}H_{29}N_2O_4$ $[M+H]^+$ 349.2127 u, found: 349.2117 u. **$[a]_\lambda^T$:** (c = 1.00 g/100 mL, $CHCl_3$) = $[a]_D^{20}$: –16.40°.

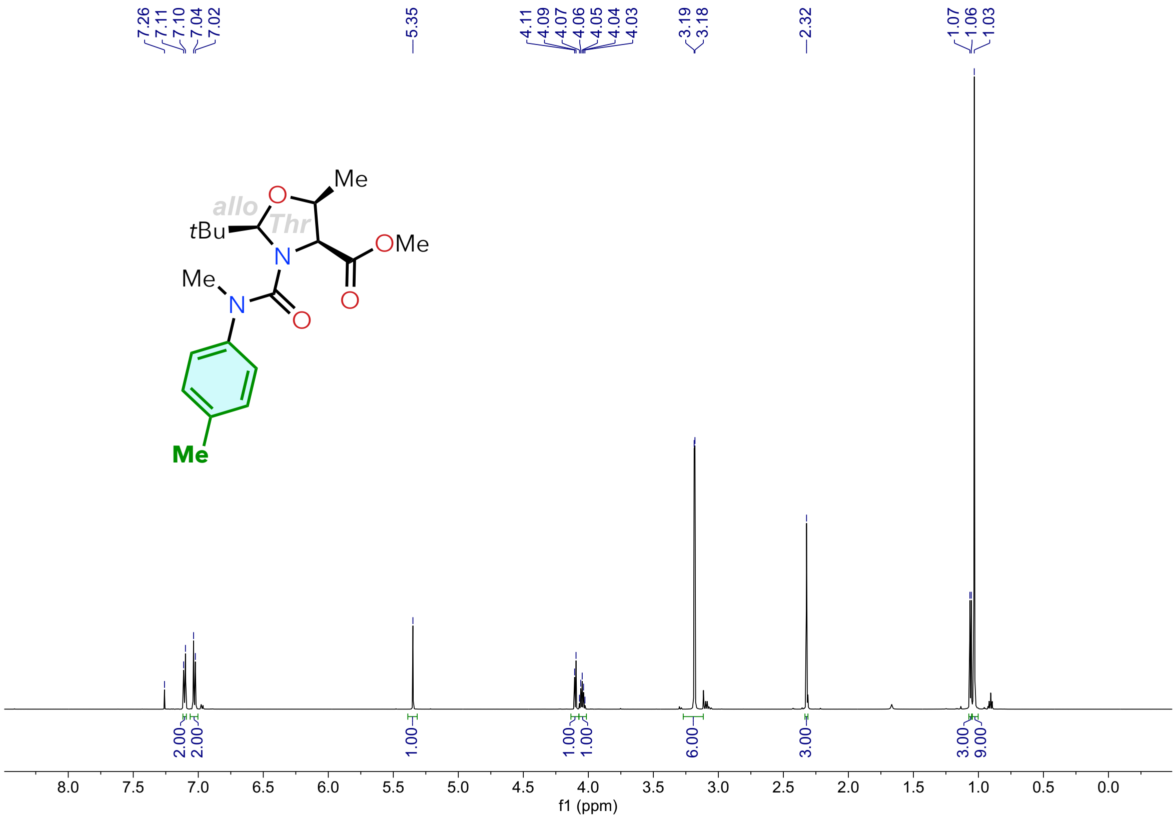
7.26
7.11
7.10
7.04
7.02
5.35
4.11
4.09
4.07
4.06
4.05
4.04
4.03
3.19
3.18
2.32
1.07
1.06
1.03
allo Thr
tBu
O
N
Me
OMe
O
Me
N
O
Me
2.00
2.00
1.00
1.00
1.00
6.00
3.00
3.00
9.00
f1 (ppm)

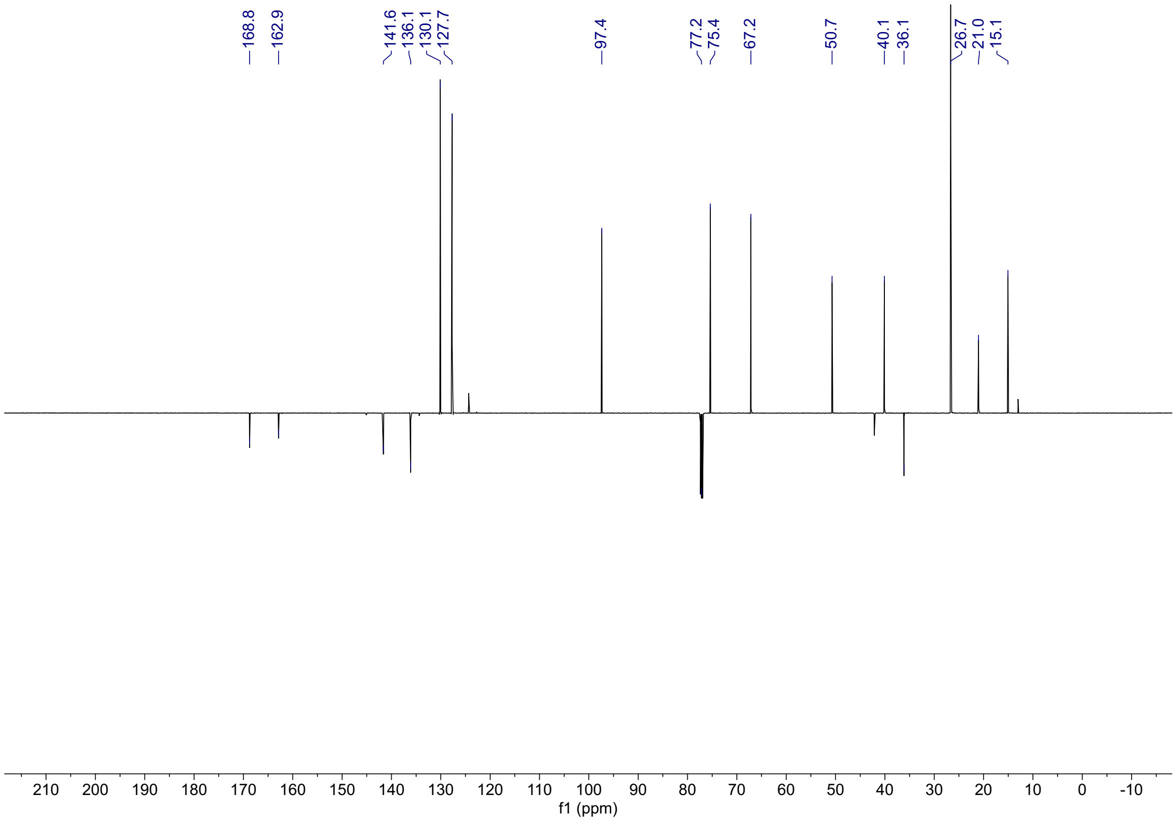
168.8
162.9
141.6
136.1
130.1
127.7
97.4
77.2
75.4
67.2
50.7
40.1
36.1
26.7
21.0
15.1
f1 (ppm)

4.4 N to C Rearrangement (Aryl Migration) according to General Procedure 4 (GP4)

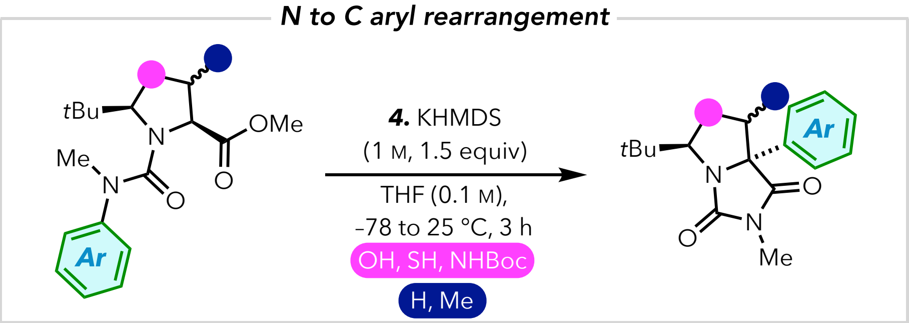

(3*R*,7a*S*)-3-(*tert*-Butyl)-6-methyl-7a-phenyldihydro-3*H*,5*H*-imidazo[1,5-*c*]oxazole-5,7(6*H*)-dione (5a)

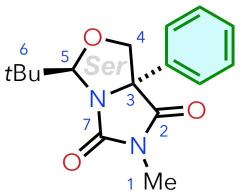

5a

Following **GP4**, to a solution of *N*-aryl urea **4a** (500 mg, 1.56 mmol, 1.0 equiv.) in dry THF (15.6 mL, 0.1 M) at –78 °C was added potassium bis(trimethylsilyl)amide solution (2.34 mL, 2.34 mmol, 1 M in THF, 1.5 equiv.) dropwise. The title compound **5a** was obtained as a white solid (740 mg, 2.57 mmol, 72%) after purification by silica gel column chromatography (gradient elution, PE/EA).

Formula: $C_{16}H_{20}N_2O_3$, **MW:** 288.35 g/mol, **m.p.:** 179 – 182 °C. **TLC:** R_f = 0.62 (PE/EA 2:1), $KMnO_4$ stain. **^{1}H NMR** (500 MHz, $CHCl_3$): δ [ppm] = 7.52 (d, *J* 7.1 Hz, 2H, Ar), 7.42 (t, *J* 7.4 Hz, 2H, Ar), 7.37 (d, *J* 7.3 Hz, 1H, Ar), 4.51 (d, *J* 8.6 Hz, 1H, H-4a), 4.25 (s, 1H, H-5), 4.01 (d, *J* 8.6 Hz, 1H, H-4b), 2.97 (s, 3H, H-1, Me), 1.25 (s, 9H, H-6, *t*Bu). **^{13}C NMR** (125 MHz, $CHCl_3$): δ [ppm] = 173.2 (s, C-2), 157.7 (s, C-7), 137.3 (s, Ar), 129.2 (d, 2Ar), 128.9 (d, Ar), 125.4 (d, 2Ar), 99.0 (d, C-5), 73.5 (s, C-3), 73.4 (t, C-4), 33.6 (s, *t*Bu), 25.7 (q, C-1, Me). **FT-IR (ATR):** $\tilde{\nu}$ [cm^{-1}] = 2959 (br w), 1784 (w), 1715 (vs), 1439 (m), 1390 (m), 1366 (w), 1283 (m), 1196 (w), 1145 (w), 1067 (m), 1049 (m), 942 (w), 887 (w), 767 (m), 729 (m), 696 (m), 629 (w), 572 (w), 503 (m). **HR-MS:** (APCI) = *m*/*z* calcd. for: $C_{16}H_{21}N_2O_3$ $[M+H]^+$ 289.1547 u, found: 289.1548 u. **$[a]_\lambda^T$:** (c = 0.92 g/100 mL, MeOH) = $[a]_D^{20}$: –10.43°.

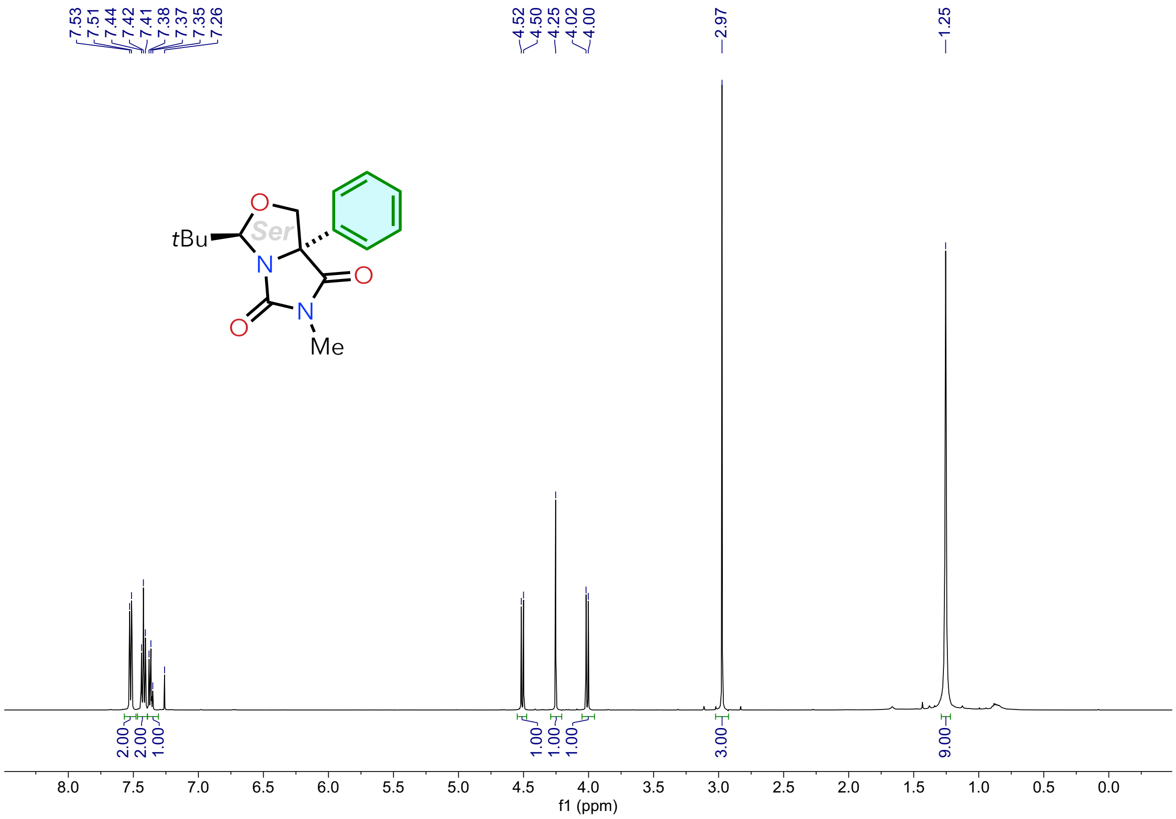
7.53
7.51
7.44
7.42
7.41
7.38
7.37
7.35
7.26
4.52
4.50
4.25
4.02
4.00
2.97
1.25
tBu
Ser
O
N
O
N
O
Me
2.00
2.00
1.00
1.00
1.00
1.00
3.00
9.00
8.0 7.5 7.0 6.5 6.0 5.5 5.0 4.5 4.0 3.5 3.0 2.5 2.0 1.5 1.0 0.5 0.0
f1 (ppm)

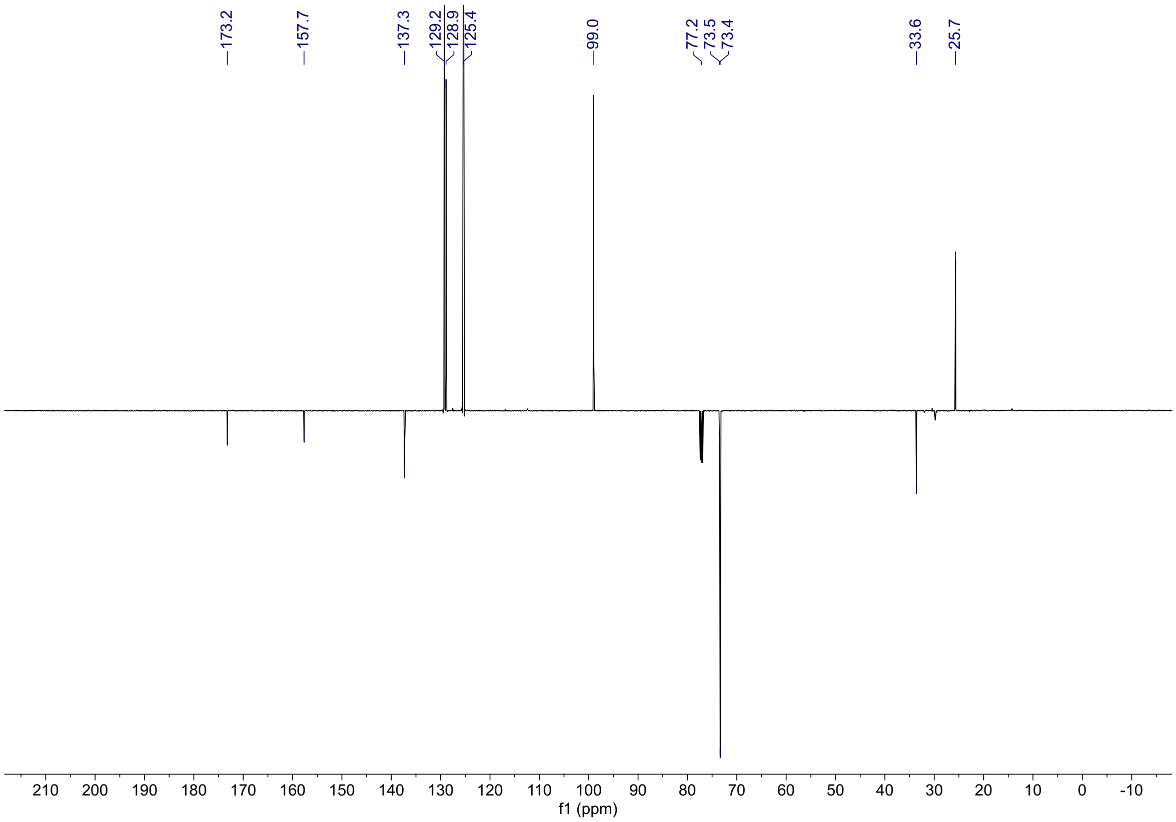
173.2
157.7
137.3
129.2
128.9
125.4
99.0
77.2
73.5
73.4
33.6
25.7
210 200 190 180 170 160 150 140 130 120 110 100 90 80 70 60 50 40 30 20 10 0 -10
f1 (ppm)

(3*R*,7a*S*)-3-(*tert*-Butyl)-7a-(3-fluorophenyl)-6-methyldihydro-3*H*,5*H*-imidazo[1,5-*c*]oxazole-5,7(6*H*)-dione (5b)

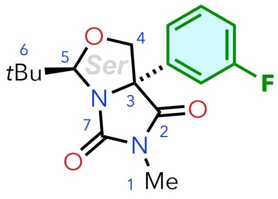

5b

Following **GP4**, to a solution of *N*-aryl urea **4b** (100 mg, 0.30 mmol, 1.0 equiv.) in dry THF (3.0 mL, 0.1 M) at –78 °C was added potassium bis(trimethylsilyl)amide solution (0.44 mL, 0.44 mmol, 1 M in THF, 1.5 equiv.) dropwise. The title compound **5b** was obtained as a colourless oil (62 mg, 0.20 mmol, 68%) after purification by silica gel column chromatography (gradient elution, PE/EA).

Formula: $C_{16}H_{19}FN_2O_3$, **MW:** 306.34 g/mol. **TLC:** R_f = 0.61 (PE/EA 2:1), $KMnO_4$ stain. **^{1}H NMR** (600 MHz, $CDCl_3$): δ [ppm] = 7.43 -7.36 (m, 1H, Ar), 7.31 (d, *J* 7.8 Hz, 1H, Ar), 7.24 (d, *J* 9.7 Hz, 1H, Ar), 7.06 (td, *J* 8.3, 2.4 Hz, 1H, Ar), 4.50 (d, *J* 8.7 Hz, 1H, H-4a), 4.23 (s, 1H, H-5), 3.96 (d, *J* 8.7 Hz, 1H, H-4b), 2.98 (s, 3H, H-1, Me), 1.25 (s, 9H, H-6, *t*Bu). **^{13}C NMR** (150 MHz, $CDCl_3$): δ [ppm] = 172.7 (s, C-2), 164.1 (s, Ar), 162.4 (s, Ar), 157.6 (s, C-7), 139.8 (s, *d J* 7.2 Hz, Ar), 131.0 (d, *d J* 8.2 Hz, Ar), 121.2 (d, *d J* 3.1 Hz, Ar), 116.0 (d, *d* 21.1 Hz, Ar), 112.7 (d, *d J* 23.5 Hz, Ar), 99.3 (d, C-5), 73.4 (t, C-4), 73.2 (s, *d J* 1.9 Hz, C-3), 33.7 (s, *t*Bu), 25.8 (q, C-1, Me). **^{19}F NMR** (565 MHz, $CDCl_3$, C_6F_6 ref.): δ [ppm] = –114.27 (td, *J* 9.0, 5.7 Hz, F). **FT-IR (ATR):** $\tilde{\nu}$ [cm^{-1}] = 2961 (br w), 1786 (w), 1716 (vs), 1613 (w), 1591 (w), 1487 (w), 1439 (m), 1390 (m), 1356 (w), 1270 (m), 1196 (w), 1144 (w), 1067 (m), 1049 (m), 933 (m), 832 (w), 781 (m), 687 (m), 635 8w), 568 (w), 521 (w), 456 (w). **HR-MS:** (ESI) = *m*/*z* calcd. for: $C_{16}H_{20}FN_2O_3$ [M+H]$^+$ 307.1458 u, found: 307.1462 u. **$[a]_{\lambda}^{T}$:** (c = 1.04 g/100 mL, $CHCl_3$) = $[a]_D^{20}$: –13.85°.

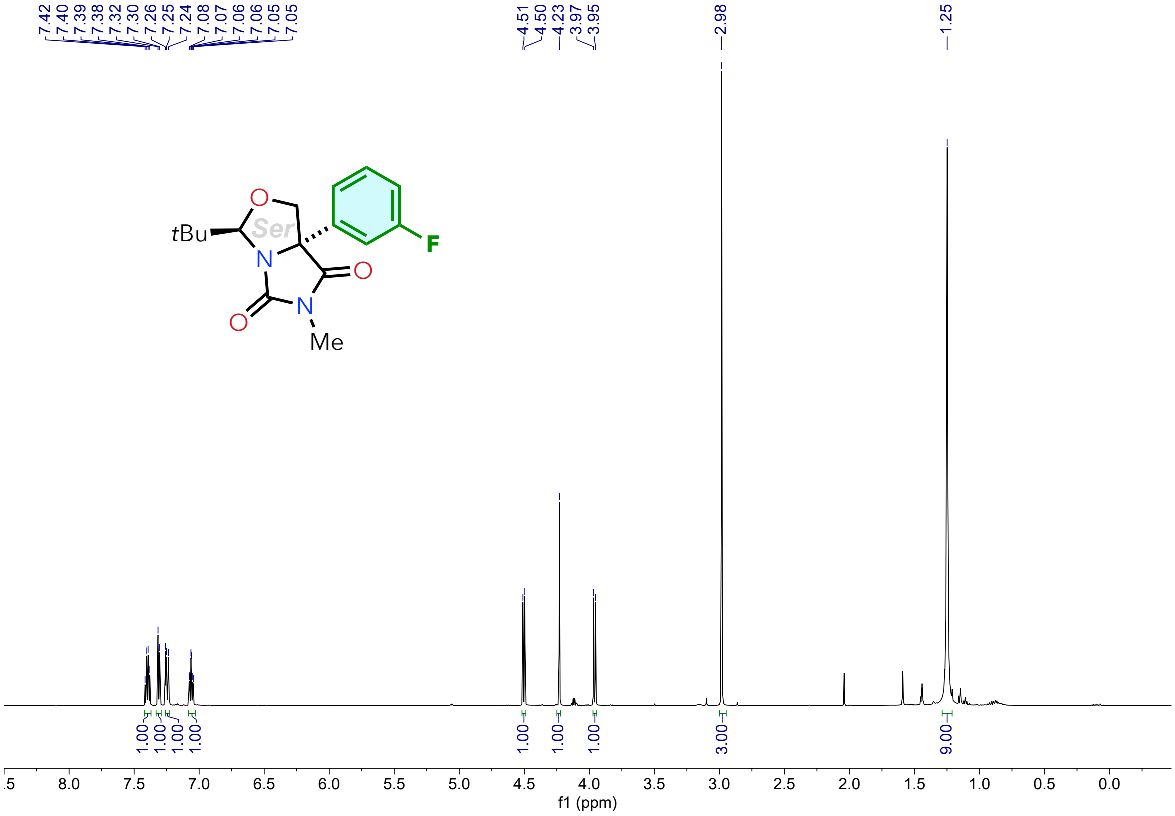

7.42
7.40
7.39
7.38
7.32
7.30
7.26
7.25
7.24
7.08
7.07
7.06
7.06
7.05
7.05
4.51
4.50
4.23
3.97
3.95
2.98
1.25
tBu
O
Ser
N
O
N
O
Me
F
1.00
1.00
1.00
1.00
1.00
1.00
1.00
3.00
9.00
f1 (ppm)

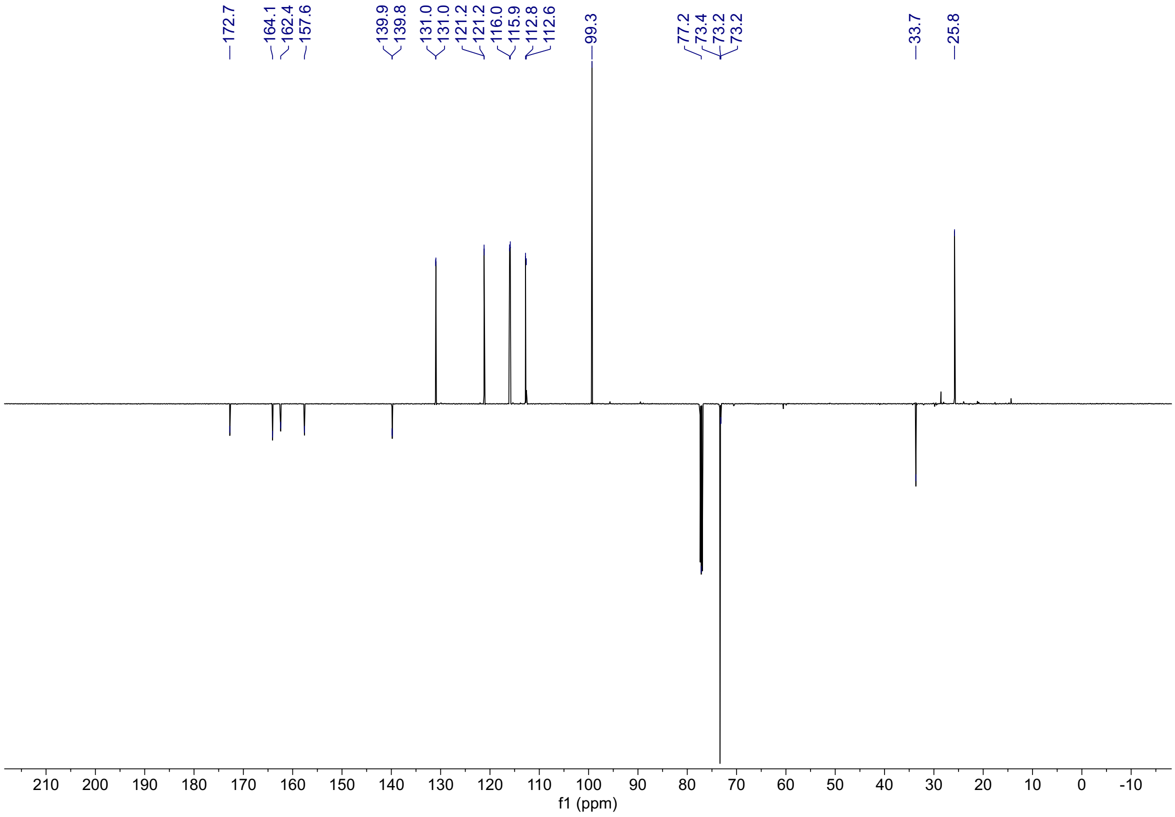

172.7
164.1
162.4
157.6
139.9
139.8
131.0
131.0
121.2
121.2
116.0
115.9
112.8
112.6
99.3
77.2
73.4
73.2
73.2
33.7
25.8
f1 (ppm)

(3*R*,7a*S*)-3-(*tert*-Butyl)-7a-(4-chlorophenyl)-6-methyldihydro-3*H*,5*H*-imidazo[1,5-*c*]oxazole-5,7(6*H*)-dione (5c)

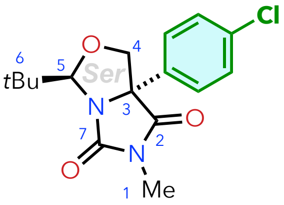

5c

Following **GP4**, to a solution of *N*-aryl urea **4c** (100 mg, 0.30 mmol, 1.0 equiv.) in dry THF (2.8 mL, 0.1 M) at –78 °C was added potassium bis(trimethylsilyl)amide solution (0.42 mL, 0.42 mmol, 1 M in THF, 1.5 equiv.) dropwise. The title compound **5c** was obtained as a white solid (64 mg, 0.20 mmol, 71%) after purification by silica gel column chromatography (gradient elution, PE/EA).

Formula: $C_{16}H_{19}ClN_2O_3$, **MW:** 322.79 g/mol, **m.p.:** 147 – 150 °C. **TLC:** R_f = 0.68 (PE/EA 2:1), $KMnO_4$ stain. **^{1}H NMR** (500 MHz, $CHCl_3$): δ [ppm] = 7.46 (d, *J* 8.7 Hz, 2H, Ar), 7.39 (d, *J* 8.7 Hz, 2H, Ar), 4.50 (d, *J* 8.6 Hz, 1H, H-4a), 4.21 (s, 1H, H-5), 3.94 (d, *J* 8.6 Hz, 1H, H-4b), 2.98 (s, 3H, H-1, Me), 1.24 (s, 9H, H-6, *t*Bu). **^{13}C NMR** (125 MHz, $CHCl_3$): δ [ppm] = 172.8 (s, C-2), 157.8 (s, C-7), 135.7 (s, Ar), 135.0 (s, Ar), 129.4 (d, 2Ar), 126.7 (d, 2Ar), 99.1 (d, C-5), 73.3 (t, C-4), 73.0 (s, C-3), 33.5 (s, *t*Bu), 25.7 (q, C-1, Me). **FT-IR (ATR):** $\tilde{\nu}$ [cm^{-1}] = 2950 (br w), 1784 (m), 1716 (vs), 1439 (m), 1394 (m), 1289 (m), 1199 (m), 1092 (m), 1066 (m), 1054 (m), 1014 (m), 963 (w), 940 (m), 930 (m), 889 (w), 835 (m), 807 (m), 786 (m), 740 (w), 706 (w), 686 (w), 575 (m), 512 (m), 483 (m). **HR-MS:** (ESI) = *m/z* calcd. for: $C_{15}H_{16}{}^{35}ClN_2O_3$ $[M–Me]^+$ 307.0849 u, found: 307.0844 u. **$[a]_\lambda^T$:** (c = 1.05 g/100 mL, $CHCl_3$) = $[a]_D^{20}$: –15.62°.

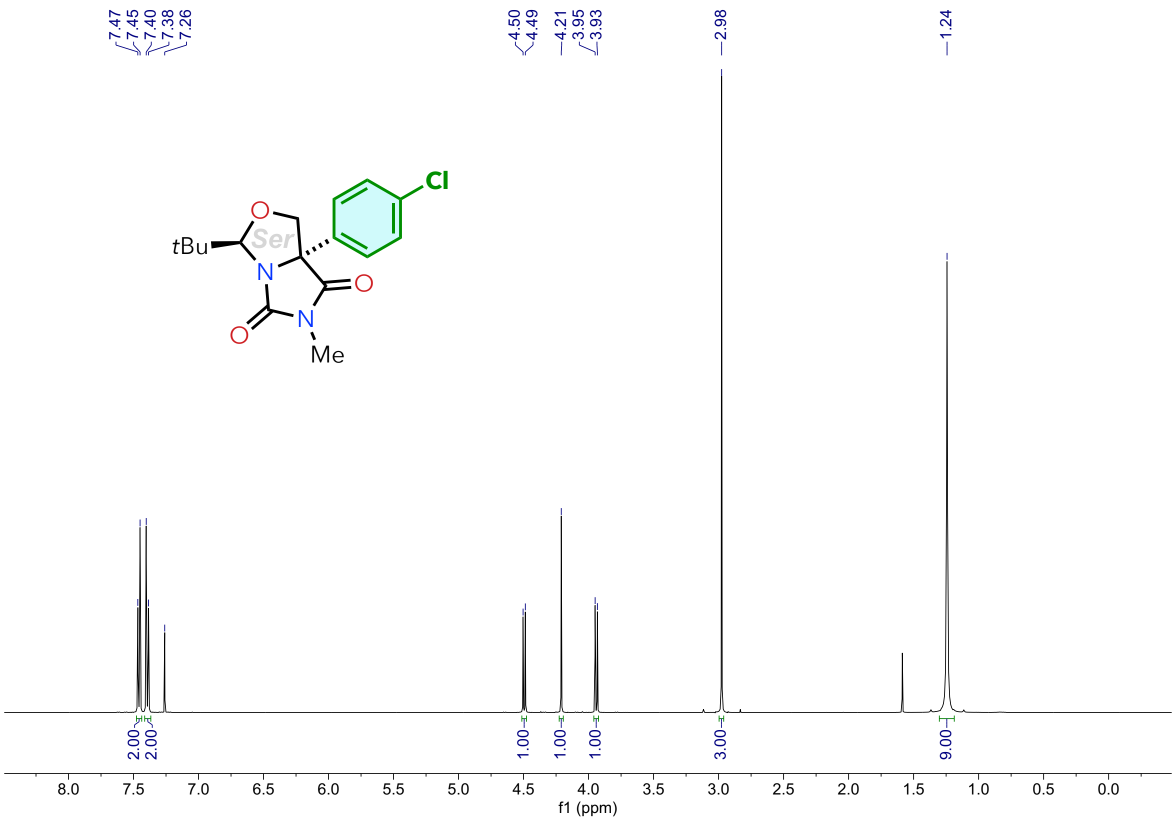
7.47
7.45
7.40
7.38
7.26
4.50
4.49
4.21
3.95
3.93
2.98
1.24
Cl
O
tBu
Ser
N
O
N
O
Me
2.00
2.00
1.00
1.00
1.00
3.00
9.00
8.0 7.5 7.0 6.5 6.0 5.5 5.0 4.5 4.0 3.5 3.0 2.5 2.0 1.5 1.0 0.5 0.0
f1 (ppm)

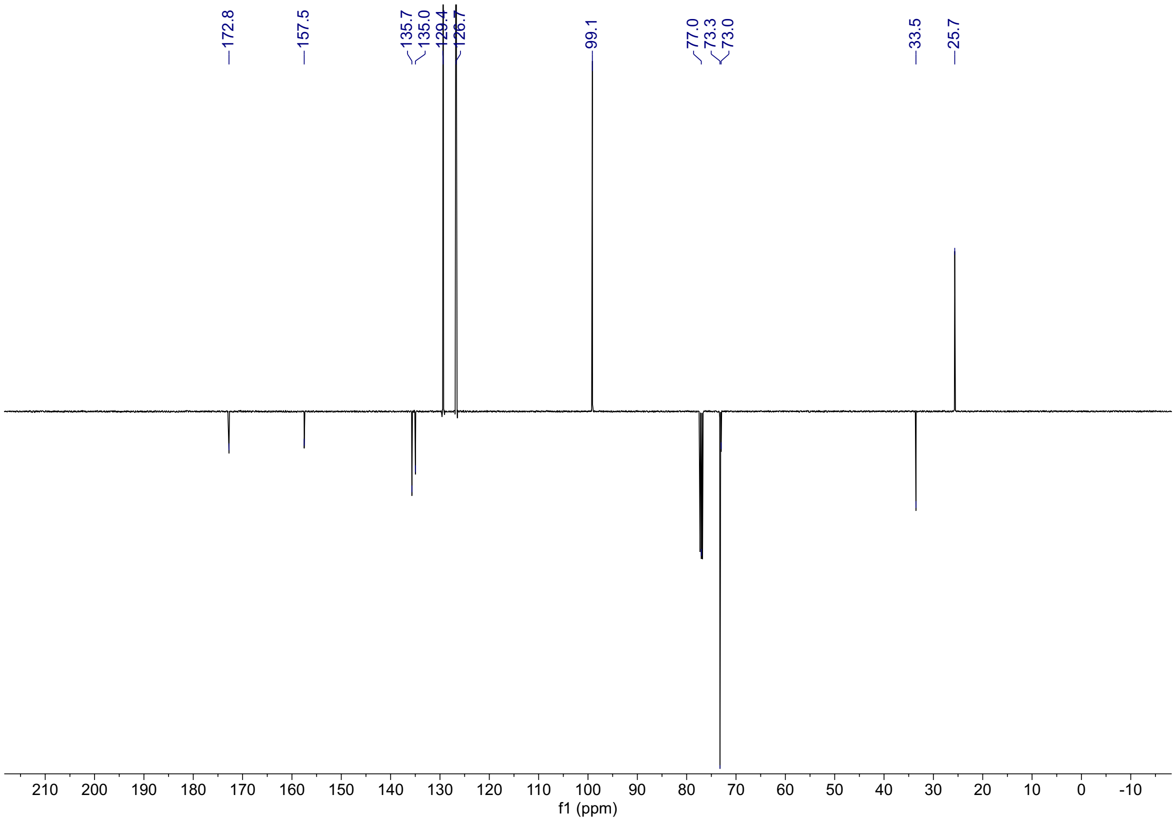
172.8
157.5
135.7
135.0
129.4
126.7
99.1
77.0
73.3
73.0
33.5
25.7
210 200 190 180 170 160 150 140 130 120 110 100 90 80 70 60 50 40 30 20 10 0 -10
f1 (ppm)

(3*R*,7a*S*)-7a-(4-Bromophenyl)-3-(*tert*-butyl)-6-methyldihydro-3*H*,5*H*-imidazo[1,5-*c*]oxazole-5,7(6*H*)-dione (5d)

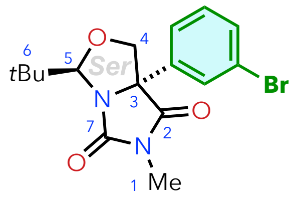

5d

Following **GP4**, to a solution of *N*-aryl urea **4d** (100 mg, 0.25 mmol, 1.0 equiv.) in dry THF (2.5 mL, 0.1 M) at –78 °C was added potassium bis(trimethylsilyl)amide solution (0.38 mL, 0.38 mmol, 1 M in THF, 1.5 equiv.) dropwise. The title compound **5d** was obtained as a white solid (67 mg, 0.18 mmol, 73%) after purification by silica gel column chromatography (gradient elution, PE/EA).

Formula: $C_{16}H_{19}BrN_2O_3$, **MW:** 367.24 g/mol, **m.p.:** 104 – 107 °C. **TLC:** R_f = 0.65 (PE/EA 2:1), $KMnO_4$ stain. **^{1}H NMR** (600 MHz, $CHCl_3$): δ [ppm] = 7.66 (t, *J* 1.8 Hz, 1H, Ar), 7.50 (ddd, *J* 7.9, 2.0, 1.0 Hz, 1H, Ar), 7.45 (ddd, *J* 7.9, 1.8, 1.0 Hz, 1H, Ar), 7.30 (t, *J* 7.9 Hz, 1H, Ar), 4.50 (d, *J* 8.7 Hz, 1H, H-4a), 4.22 (s, 1H, H-5), 3.95 (d, *J* 8.7 Hz, 1H, H-4b), 2.98 (s, 3H, H-1, Me), 1.25 (s, 9H, H-6, *t*Bu). **^{13}C NMR** (150 MHz, $CHCl_3$): δ [ppm] = 172.7 (s, C-2), 157.6 (s, C-7), 139.6 (s, Ar), 132.1 (d, Ar), 130.9 (d, Ar), 128.4 (d, Ar), 124.3 (d, Ar), 123.4 (s, Ar), 99.4 (d, C-5), 73.3 (t, C-4), 73.1 (s, C-3), 33.7 (s, *t*Bu), 25.8 (q, C-1, Me). **FT-IR (ATR):** $\tilde{\nu}$ [cm^{-1}] = 2963 (br w), 1786 (m), 1719 (vs), 1567 (w), 1438 (m), 1383 (m), 1367 (m), 1286 (m), 1199 (m), 1142 (m), 1048 (m), 942 (m), 885 (m), 820 (w), 783 (s), 761 (m), 705 (m), 680 (m), 634 (m), 579 (m), 543 (w), 433 (m). **HR-MS:** (ESI) = *m/z* calcd. for: $C_{15}H_{16}{}^{79}BrN_2O_3$ $[M–Me]^+$ 351.0344 u, found: 351.0333 u. **$[a]_\lambda^T$:** (c = 1.00 g/100 mL, $CHCl_3$) = $[a]_D^{20}$: –15.60°.

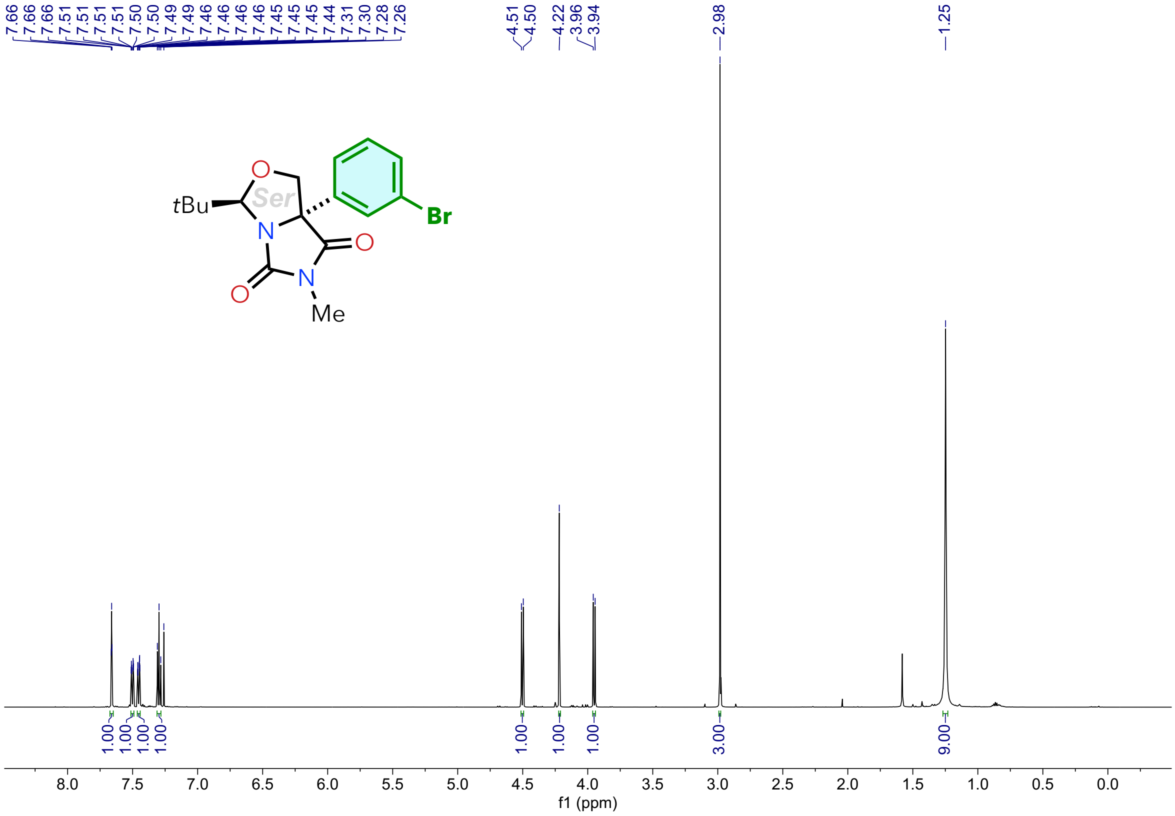
7.66
7.66
7.66
7.51
7.51
7.51
7.50
7.50
7.49
7.46
7.46
7.46
7.45
7.45
7.45
7.44
7.31
7.30
7.28
7.26
4.51
4.50
4.22
3.96
3.94
2.98
1.25
tBu
Ser
O
N
N
O
O
Me
Br
1.00
1.00
1.00
1.00
1.00
1.00
1.00
3.00
9.00
8.0 7.5 7.0 6.5 6.0 5.5 5.0 4.5 4.0 3.5 3.0 2.5 2.0 1.5 1.0 0.5 0.0
f1 (ppm)

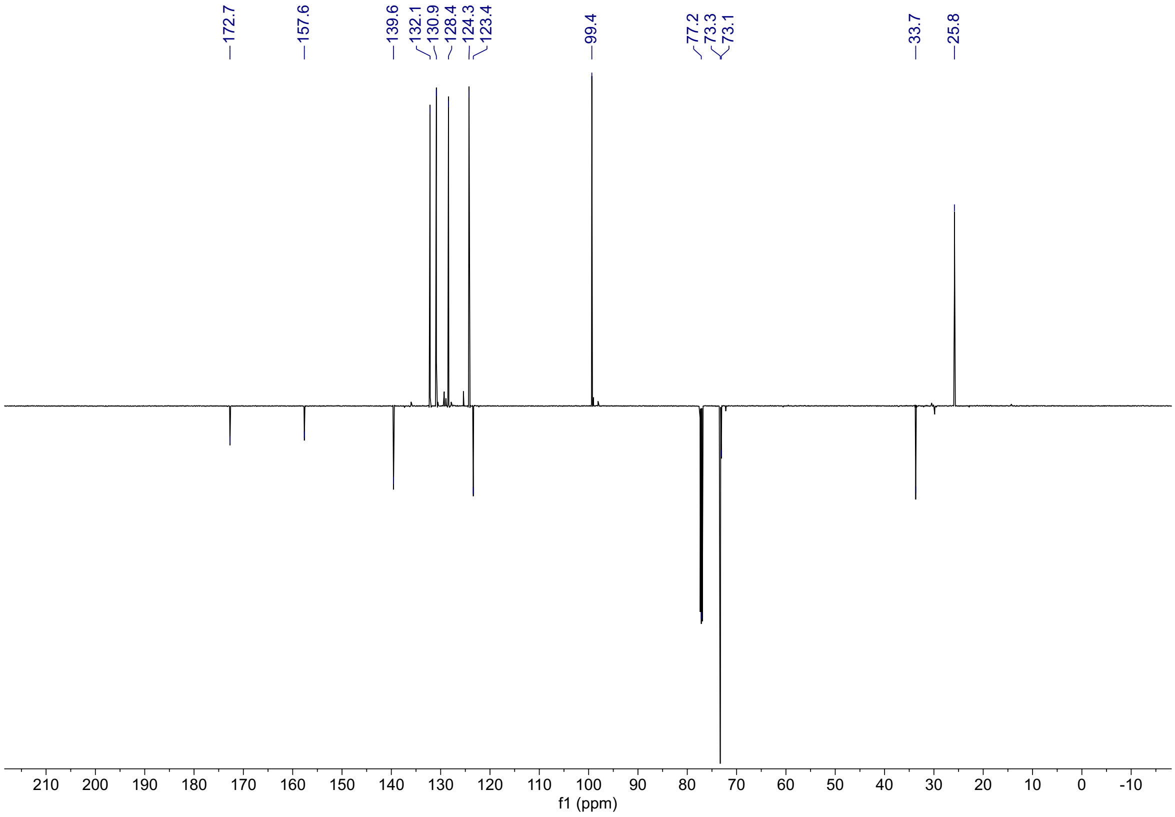
172.7
157.6
139.6
132.1
130.9
128.4
124.3
123.4
99.4
77.2
73.3
73.1
33.7
25.8
210 200 190 180 170 160 150 140 130 120 110 100 90 80 70 60 50 40 30 20 10 0 -10
f1 (ppm)

(3*R*,7a*S*)-7a-(2-Bromophenyl)-3-(*tert*-butyl)-6-methyldihydro-3*H*,5*H*-imidazo[1,5-*c*]oxazole-5,7(6*H*)-dione (5e)

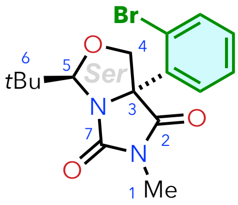

5e

Following **GP4**, to a solution of *N*-aryl urea **4e** (100 mg, 0.25 mmol, 1.0 equiv.) in dry THF (2.5 mL, 0.1 M) at –78 °C was added potassium bis(trimethylsilyl)amide solution (0.38 mL, 0.38 mmol, 1 M in THF, 1.5 equiv.) dropwise. The title compound **5e** was obtained as a white solid (77 mg, 0.21 mmol, 84%) after purification by silica gel column chromatography (gradient elution, PE/EA).

Formula: $C_{16}H_{19}BrN_2O_3$, **MW:** 367.24 g/mol. **TLC:** R_f = 0.60 (PE/EA 2:1), $KMnO_4$ stain. **^{1}H NMR** (600 MHz, $CHCl_3$): δ [ppm] = 7.69 (dd, *J* 7.9, 1.2 Hz, 1H, Ar), 7.62 (dd, *J* 7.9, 1.6 Hz, 1H, Ar), 7.34 (td, *J* 7.7, 1.2 Hz, 1H, Ar), 7.22 (td, *J* 7.6, 1.7 Hz, 1H, Ar), 4.68 (d, *J* 9.5 Hz, 1H, H-4a), 4.40 (d, *J* 9.5 Hz, 1H, H-4b), 4.03 (s, 1H, H-5), 2.97 (s, 3H, H-1, Me), 1.24 (s, 9H, H-6, *t*Bu). **^{13}C NMR** (150 MHz, $CHCl_3$): δ [ppm] = 171.6 (s, C-2), 157.4 (s, C-7), 136.2 (s, Ar), 135.9 (d, Ar), 130.6 (d, Ar), 127.8 (d, Ar), 127.7 (d, Ar), 122.2 (s, Ar), 98.0 (d, C-5), 74.2 (s, C-3), 72.2 (t, C-4), 33.4 (s, *t*Bu), 25.6 (q, C-1, Me). **FT-IR (ATR):** $\tilde{\nu}$ [cm^{-1}] = 2959 (br w), 1786 (w), 1717 (vs), 1438 (m), 1390 (m), 1366 (w), 1284 (m), 1199 (m), 1136 (w), 1070 (m), 1049 (m), 1023 (m), 948 (w), 888 (w), 764 (m), 748 (m), 702 (w), 675 (w), 624 (w), 576 (w), 520 (w), 449 (w). **HR-MS:** (ESI) = *m/z* calcd. for: $C_{16}H_{20}{}^{79}BrN_2O_3$ $[M+H]^+$ 367.0657 u, found: 367.0656 u. **$[a]_\lambda^T$:** (c = 0.71 g/100 mL, $CHCl_3$) = $[a]_D^{20}$: –16.34°.

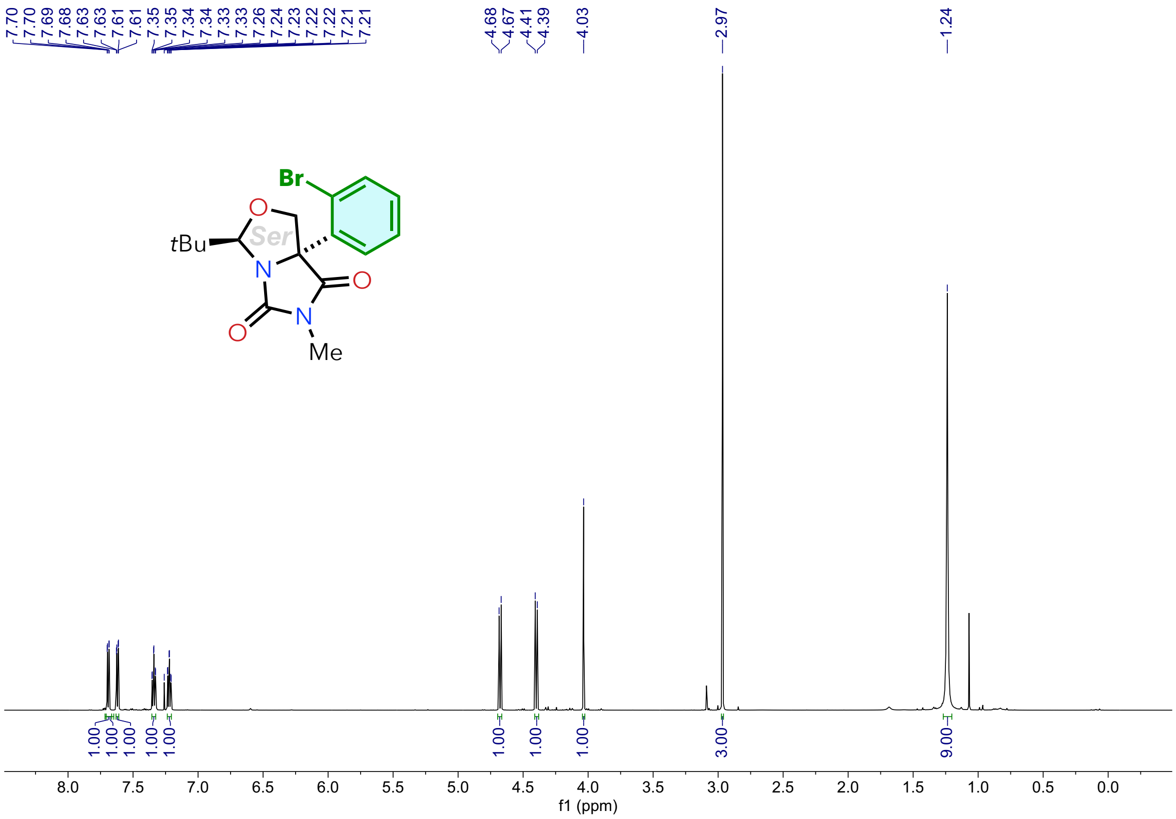
7.70 7.70 7.69 7.68 7.63 7.63 7.61 7.61 7.35 7.35 7.34 7.34 7.33 7.33 7.26 7.24 7.23 7.22 7.22 7.21 7.21
4.68 4.67 4.41 4.39
4.03
2.97
1.24
Br
O
tBu
Ser
N
O
O
N
Me
1.00 1.00 1.00 1.00 1.00
1.00 1.00 1.00
3.00
9.00
8.0 7.5 7.0 6.5 6.0 5.5 5.0 4.5 4.0 3.5 3.0 2.5 2.0 1.5 1.0 0.5 0.0
f1 (ppm)

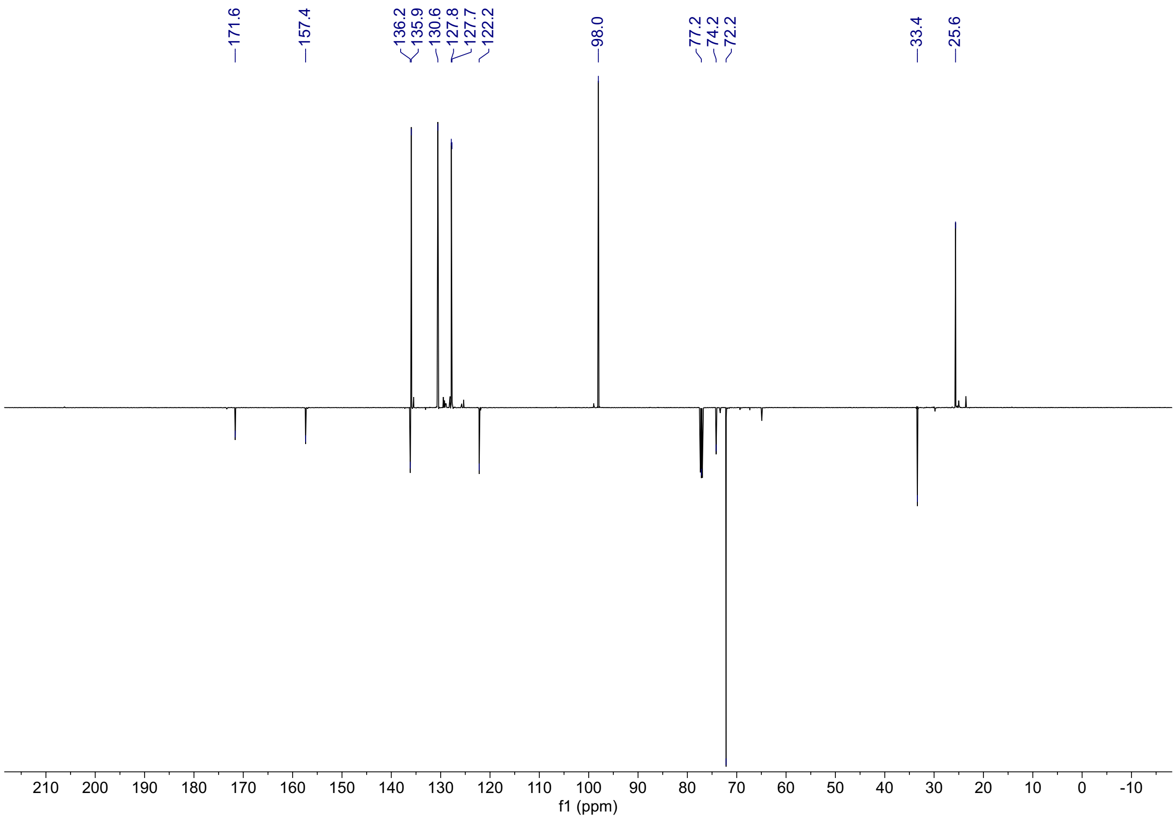
171.6
157.4
136.2 135.9 130.6 127.8 127.7 122.2
98.0
77.2 74.2 72.2
33.4
25.6
210 200 190 180 170 160 150 140 130 120 110 100 90 80 70 60 50 40 30 20 10 0 -10
f1 (ppm)

(3*R*,7a*S*)-3-(*tert*-Butyl)-6-methyl-7a-(4-(trifluoromethyl)phenyl)dihydro-3*H*,5*H*-imidazo[1,5-*c*]oxazole-5,7(6*H*)-dione (5f)

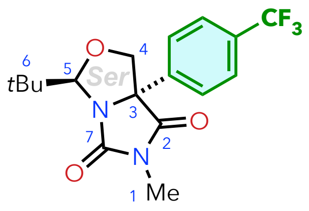

5f

Following **GP4**, to a solution of *N*-aryl urea **4f** (100 mg, 0.26 mmol, 1.0 equiv.) in dry THF (2.6 mL, 0.1 M) at –78 °C was added potassium bis(trimethylsilyl)amide solution (0.39 mL, 0.39 mmol, 1 M in THF, 1.5 equiv.) dropwise. The title compound **5f** was obtained as a white solid (78 mg, 0.22 mmol, 84%) after purification by silica gel column chromatography (gradient elution, PE/EA).

Formula: $C_{17}H_{19}F_3N_2O_3$, **MW:** 356.35 g/mol, **m.p.:** 103 – 106 °C. **TLC:** R_f = 0.74 (PE/EA 2:1), $KMnO_4$ stain. **^{1}H NMR** (600 MHz, $CDCl_3$): δ [ppm] = 7.68 (q, *J* 8.5 Hz, 4H, Ar), 4.55 (d, *J* 8.7 Hz, 1H, H-4a), 4.22 (s, 1H, H-5), 3.96 (d, *J* 9.0 Hz, 1H, H-4b), 2.99 (s, 3H, H-1, Me), 1.25 (s, 9H, *t*Bu). **^{13}C NMR** (150 MHz, $CDCl_3$): δ [ppm] = 172.6 (s, C-2), 157.6 (s, C-7), 141.3 (s, Ar), 131.3 (s, *q J* 32.8 Hz, Ar), 126.3 (d, *q J* 3.7 Hz, Ar), 126.0 (d, 2Ar), 124.0 (s, *q J* 272.0 Hz, Ar), 99.5 (d, C-5), 73.4 (t, C-4), 73.4 (s, C-3), 33.7 (s, *t*Bu), 25.9 (q, C-1, Me). **^{19}F NMR** (565 MHz, $CDCl_3$, C_6F_6 ref.): δ [ppm] = –66.00 (s, 3F). **FT-IR (ATR):** $\tilde{\nu}$ [cm^{-1}] = 2965 (br w), 1785 (m), 1717 (vs), 1618 (w), 1439 (m), 1412 (w), 1392 (m), 1369 (w), 1356 (w), 1327 (s), 1318 (s), 1284 (m), 1156 (m), 1135 (vs), 1117 (s), 1053 (s), 1015 (m), 843 (m), 891 (w), 848 (m), 809 (m), 790 (w), 758 (w), 698 (m), 641 (w), 596 (m), 574 (m), 508 (w). **HR-MS:** (ESI) = *m*/*z* calcd. for: $C_{17}H_{20}F_3N_2O_3$ $[M+H]^+$ 357.1426 u, found: 357.1429 u. **$[a]_\lambda^T$:** (c = 1.01 g/100 mL, $CHCl_3$) = $[a]_D^{20}$: –15.05°.

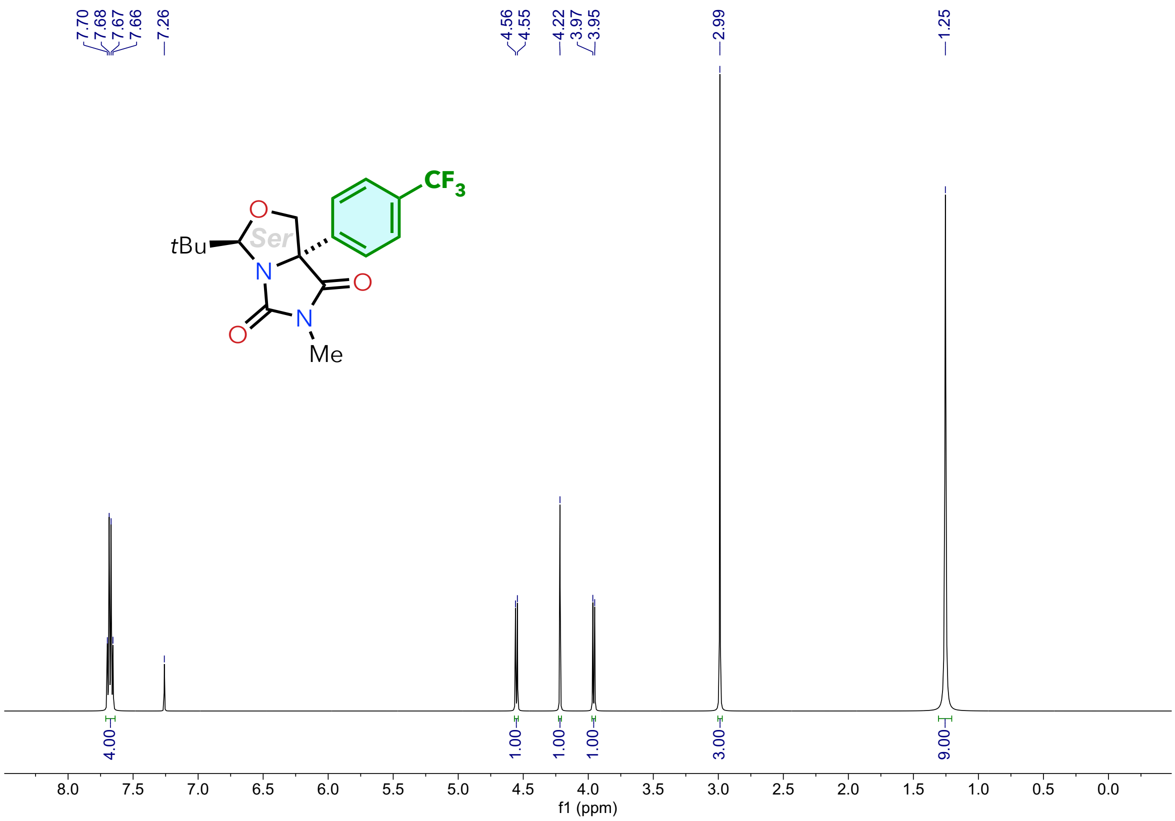
7.70
7.68
7.67
7.66
7.26
4.56
4.55
4.22
3.97
3.95
2.99
1.25
tBu
Ser
CF3
Me
4.00
1.00
1.00
1.00
3.00
9.00
f1 (ppm)

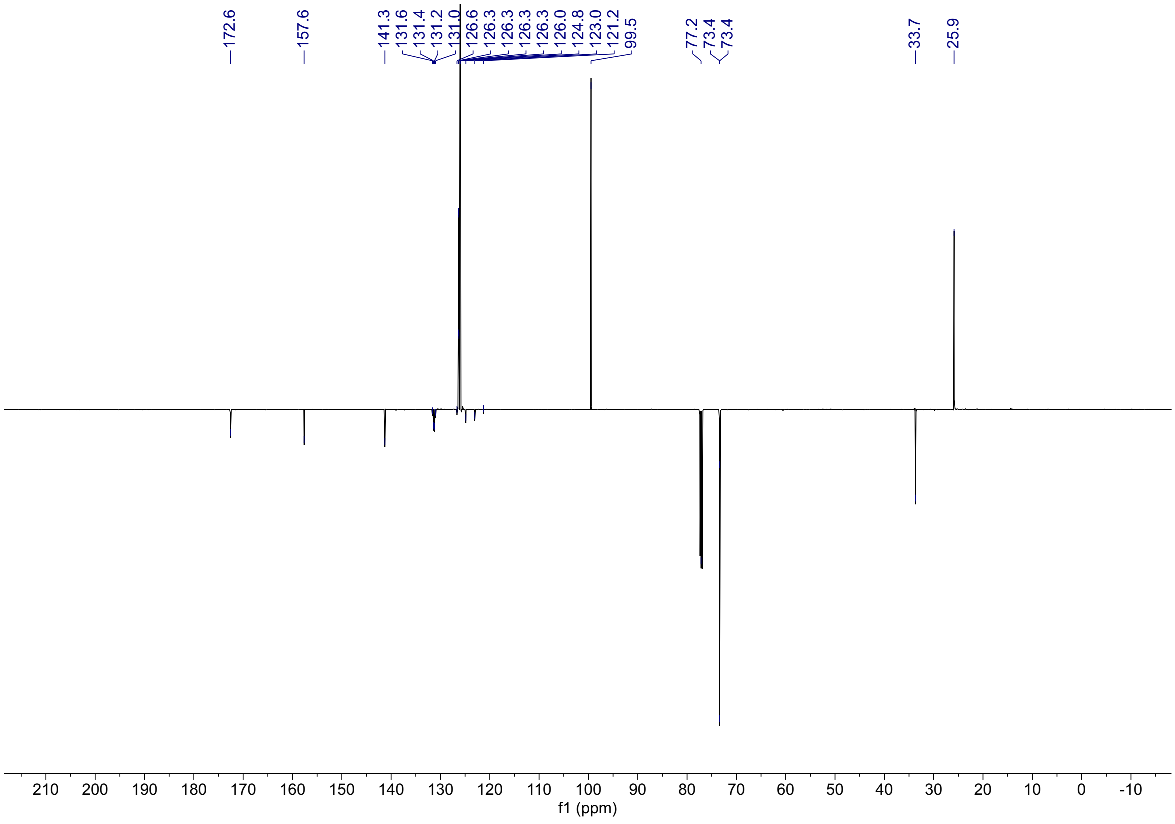
172.6
157.6
141.3
131.6
131.4
131.2
131.0
126.6
126.3
126.3
126.3
126.3
126.0
124.8
123.0
121.2
99.5
77.2
73.4
73.4
33.7
25.9
f1 (ppm)

4-((3*R*,7a*S*)-3-(*tert*-Butyl)-6-methyl-5,7-dioxodihydro-1*H*,3*H*-imidazo[1,5-*c*]oxazol-7a(5*H*)-yl)benzonitrile (5g)

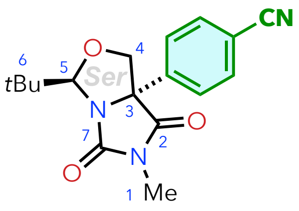

5g

Following **GP4**, to a solution of *N*-aryl urea **4g** (100 mg, 0.29 mmol, 1.0 equiv.) in dry THF (2.9 mL, 0.1 M) at –78 °C was added potassium bis(trimethylsilyl)amide solution (0.43 mL, 0.43 mmol, 1 M in THF, 1.5 equiv.) dropwise. The title compound **5g** was obtained as a white solid (72 mg, 0.23 mmol, 78%) after purification by silica gel column chromatography (gradient elution, PE/EA).

Formula: $C_{17}H_{19}N_3O_3$, **MW:** 313.36 g/mol, **m.p.:** 99 – 102 °C. **TLC:** R_f = 0.61 (PE/EA 2:1), $KMnO_4$ stain. **^{1}H NMR** (600 MHz, $CDCl_3$): δ [ppm] = 7.72 (d, *J* 8.5 Hz, 2H, Ar), 7.66 (d, *J* 8.5 Hz, 2H, Ar), 4.55 (d, *J* 8.7 Hz, 1H, H-4a), 4.19 (s, 1H, H-5), 3.91 (d, *J* 8.7 Hz, 1H, H-4b), 2.98 (s, 3H, H-1, Me), 1.25 (s, 9H, *t*Bu). **^{13}C NMR** (150 MHz, $CDCl_3$): δ [ppm] = 172.2 (s, C-2), 157.6 (s, C-7), 142.4 (s, Ar), 133.0 (d, 2Ar), 126.4 (d, 2Ar), 118.3 (s, Ar), 113.1 (s, Ar), 99.7 (d, C-5), 73.3 (t, C-4), 33.7 (s, C-6, *t*Bu), 25.9 (q, Me). **FT-IR (ATR):** $\tilde{\nu}$ [cm^{-1}] = 2961 (br w), 2230 (w), 1786 (w), 1716 (vs), 1440 (m), 1390 (m), 1281 (m), 1195 (m), 1144 (m), 1050 (m), 942 (m), 947 (w), 847 (w), 810 (w), 691 (w), 558 (m). **HR-MS:** (ESI) = *m*/*z* calcd. for: $C_{17}H_{20}N_3O_3$ [M+H]$^+$ 314.1499 u, found: 314.1500 u. **$[a]_\lambda^T$:** (c = 1.12 g/100 mL, $CHCl_3$) = $[a]_D^{20}$: –15.36°.

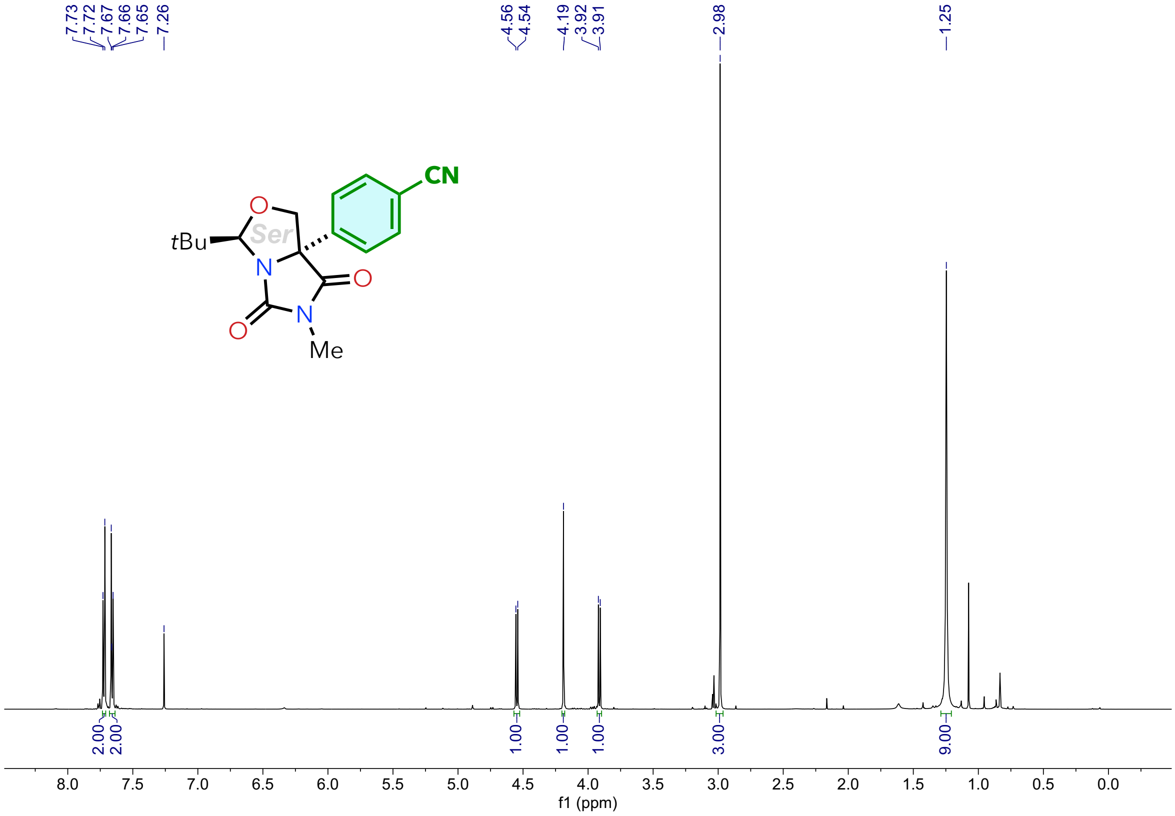
7.73
7.72
7.67
7.66
7.65
7.26
4.56
4.54
4.19
3.92
3.91
2.98
1.25
O
Ser
tBu
N
O
N
O
Me
CN
2.00
2.00
1.00
1.00
1.00
3.00
9.00
f1 (ppm)

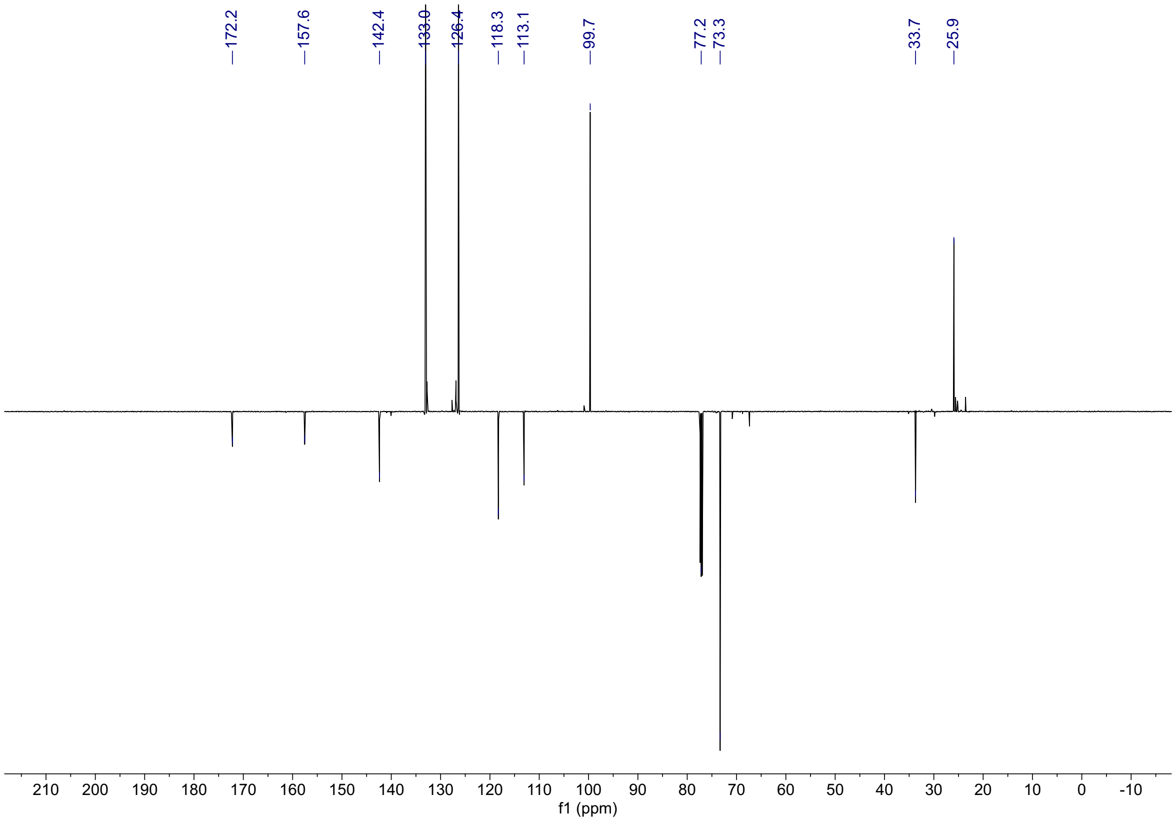
172.2
157.6
142.4
133.0
126.4
118.3
113.1
99.7
77.2
73.3
33.7
25.9
f1 (ppm)

3-((3*R*,7a*S*)-3-(*tert*-Butyl)-6-methyl-5,7-dioxodihydro-1*H*,3*H*-imidazo[1,5-*c*]oxazol-7a(5*H*)-yl)benzonitrile (5h)

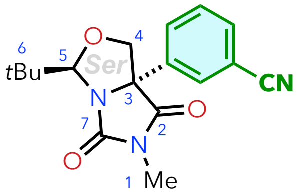

5h

Following **GP4**, to a solution of *N*-aryl urea **4h** (100 mg, 0.29 mmol, 1.0 equiv.) in dry THF (2.9 mL, 0.1 M) at –78 °C was added potassium bis(trimethylsilyl)amide solution (0.43 mL, 0.43 mmol, 1 M in THF, 1.5 equiv.) dropwise. The title compound **5h** was obtained as a white solid (77 mg, 0.25 mmol, 85%) after purification by silica gel column chromatography (gradient elution, PE/EA).

Formula: $C_{17}H_{19}N_3O_3$, **MW:** 313.36 g/mol, **m.p.:** 57 – 60 °C. **TLC:** R_f = 0.58 (PE/EA 2:1), $KMnO_4$ stain. **^{1}H NMR** (600 MHz, $CDCl_3$): δ [ppm] = 7.82 (t, *J* 1.5 Hz, 1H, Ar), 7.78 (dt, *J* 8.0, 1.5 Hz, 1H, Ar), 7.67 (dt, *J* 7.7, 1.3 Hz, 1H, Ar), 7.56 (t, *J* 7.8 Hz, 1H, Ar), 4.54 (d, *J* 8.7 Hz, 1H, H-4a), 4.20 (s, 1H, H-5), 3.92 (d, *J* 8.7 Hz, 1H, H-4b), 2.99 (s, 3H, H-1, Me), 1.25 (s, 9H, *t*Bu). **^{13}C NMR** (150 MHz, $CDCl_3$): δ [ppm] = 172.3 (s, C-2), 157.6 (s, C-7), 139.0 (s, Ar), 132.6 (d, Ar), 130.2 (d, Ar), 130.1 (d, Ar), 129.0 (d, Ar), 118.3 (s, Ar), 113.6 (s, Ar), 99.7 (d, C-5), 73.4 (t, C-4), 33.8 (s, C-6, *t*Bu), 25.9 (q, Me). **FT-IR (ATR):** $\tilde{\nu}$ [cm^{-1}] = 2960 (br w), 2231 (w), 1785 (w), 1713 (vs), 1440 (m), 1389 (m), 1282 (m), 1196 (w), 1144 (w), 1050 (m), 932 (w), 801 (w), 782 (w), 688 (m), 636 (w), 572 (w), 473 (m). **HR-MS:** (ESI) = *m/z* calcd. for: $C_{17}H_{20}N_3O_3$ $[M+H]^+$ 314.1505 u, found: 314.1499 u. **$[a]_{\lambda}^{T}$:** (c = 1.10 g/100 mL, $CHCl_3$) = $[a]_D^{20}$: –17.45°.

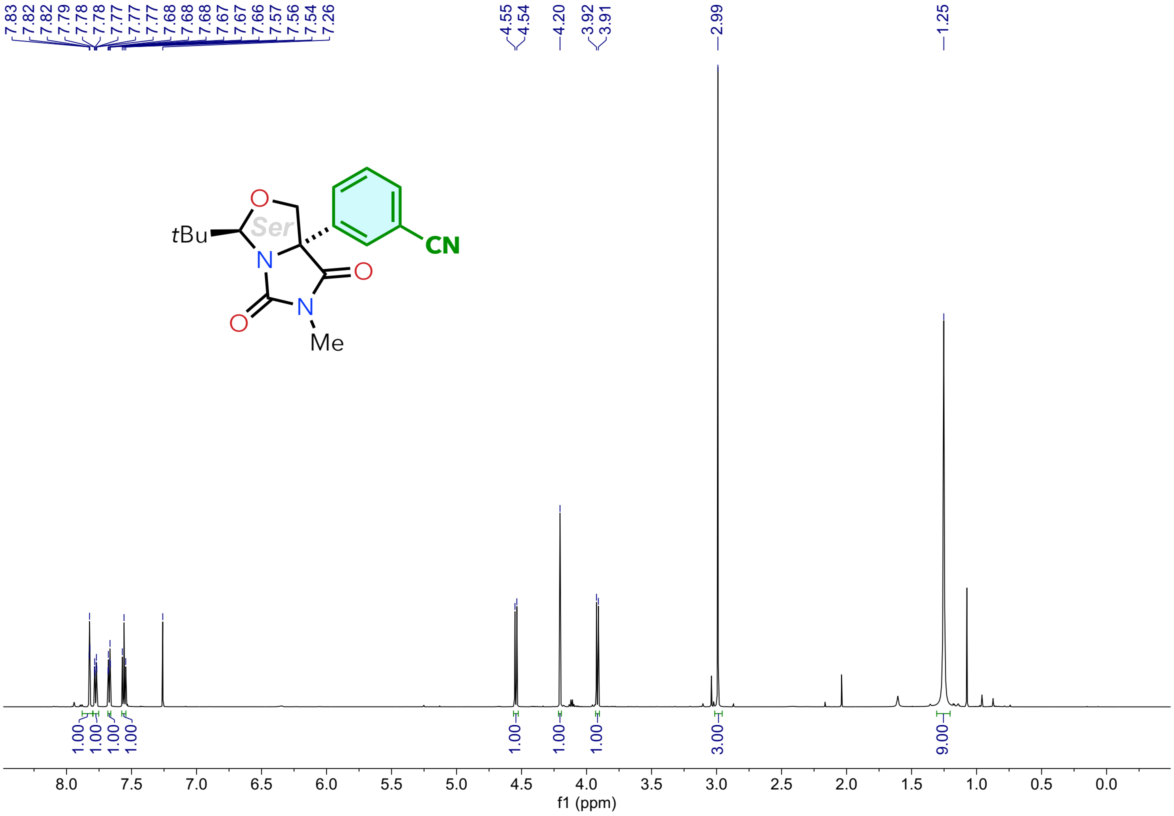
7.83
7.82
7.82
7.79
7.78
7.78
7.77
7.77
7.77
7.68
7.68
7.68
7.67
7.67
7.66
7.57
7.56
7.54
7.26
4.55
4.54
4.20
3.92
3.91
2.99
1.25
tBu
O
Ser
N
O
N
O
Me
CN
1.00
1.00
1.00
1.00
1.00
1.00
1.00
3.00
9.00
8.0 7.5 7.0 6.5 6.0 5.5 5.0 4.5 4.0 3.5 3.0 2.5 2.0 1.5 1.0 0.5 0.0
f1 (ppm)

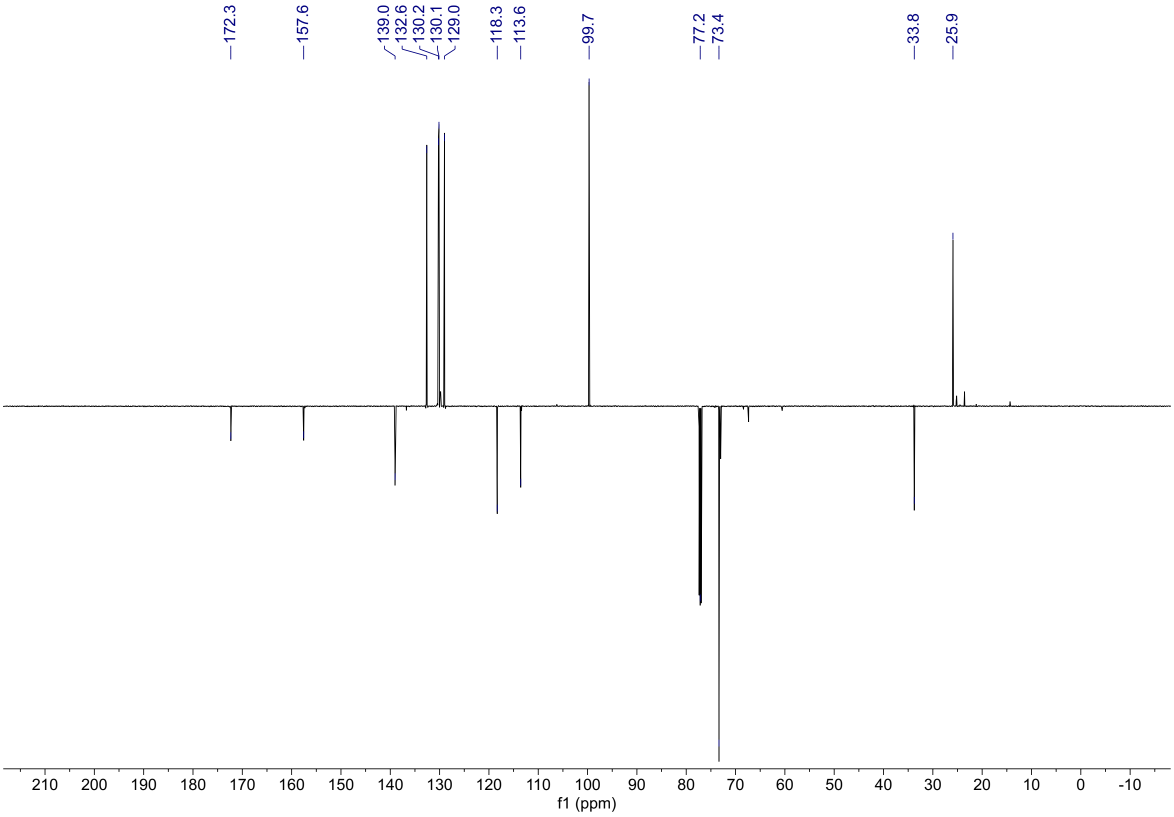
172.3
157.6
139.0
132.6
130.2
130.1
129.0
118.3
113.6
99.7
77.2
73.4
33.8
25.9
210 200 190 180 170 160 150 140 130 120 110 100 90 80 70 60 50 40 30 20 10 0 -10
f1 (ppm)

(3*R*,7a*S*)-3-(*tert*-butyl)-7a-(3-ethynylphenyl)-6-methyldihydro-3*H*,5*H*-imidazo[1,5-*c*]oxazole-5,7(6*H*)-dione (5i)

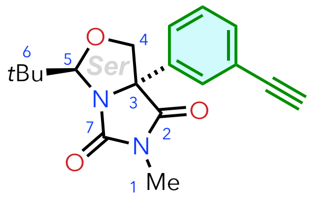

5i

Following **GP4**, to a solution of *N*-aryl urea **4i** (100 mg, 0.29 mmol, 1.0 equiv.) in dry THF (2.9 mL, 0.1 M) at –78 °C was added potassium bis(trimethylsilyl)amide solution (0.44 mL, 0.44 mmol, 1 M in THF, 1.5 equiv.) dropwise. The title compound **5i** was obtained as a white solid (41 mg, 0.13 mmol, 45%) after purification by silica gel column chromatography (gradient elution, PE/EA).

Formula: $C_{18}H_{20}N_2O_3$, **MW:** 312.37 g/mol, **m.p.:** 126 – 129 °C. **TLC:** R_f = 0.58 (PE/EA 2:1), $KMnO_4$ stain. **^{1}H NMR** (600 MHz, $CHCl_3$): δ [ppm] = 7.64 (s, 1H, Ar), 7.50 (t, *J* 8.2 Hz, 2H, Ar), 7.39 (t, *J* 7.8 Hz, 1H, Ar), 4.51 (d, *J* 8.7 Hz, 1H, H-4a), 4.22 (s, 1H, H-5), 3.97 (d, *J* 8.7 Hz, 1H, H-4b), 3.12 (s, 1H, Ar, ≡CH), 2.98 (s, 3H, H-1, Me), 1.25 (s, 9H, *t*Bu). **^{13}C NMR** (150 MHz, $CHCl_3$): δ [ppm] = 172.9 (s, C-2), 157.7 (s, Ar), 137.7 (s, Ar), 132.6 (d, Ar), 129.4 (d, Ar), 128.9 (d, Ar), 126.0 (d, Ar), 123.2 (s, Ar), 99.2 (d, C-5), 83.0 (s, Ar, C≡), 78.3 (s, Ar, ≡CH), 73.3 (t, C-4), 33.7 (s, C-6, *t*Bu), 25.8 (q, C-1, Me). **FT-IR (ATR):** $\tilde{\nu}$ [cm^{-1}] = 3286 (w), 2963 (br w), 1786 (m), 1721 (vs), 1440 (m), 1384 (m), 1277 (m), 1237 (w), 1201 (m), 1144 (w), 1049 (m), 1027 (m), 943 (m), 905 (w), 802 (w), 786 (m), 690 (m), 666 (m), 636 (m), 577 (w), 461 (w). **HR-MS:** (ESI) = *m*/*z* calcd. for: $C_{18}H_{20}N_2O_3$ $[M+H]^+$ 313.1552 u, found: 313.1552 u. **$[a]_\lambda^T$:** (c = 1.03 g/100 mL, $CHCl_3$) = $[a]_D^{20}$: –18.64°.

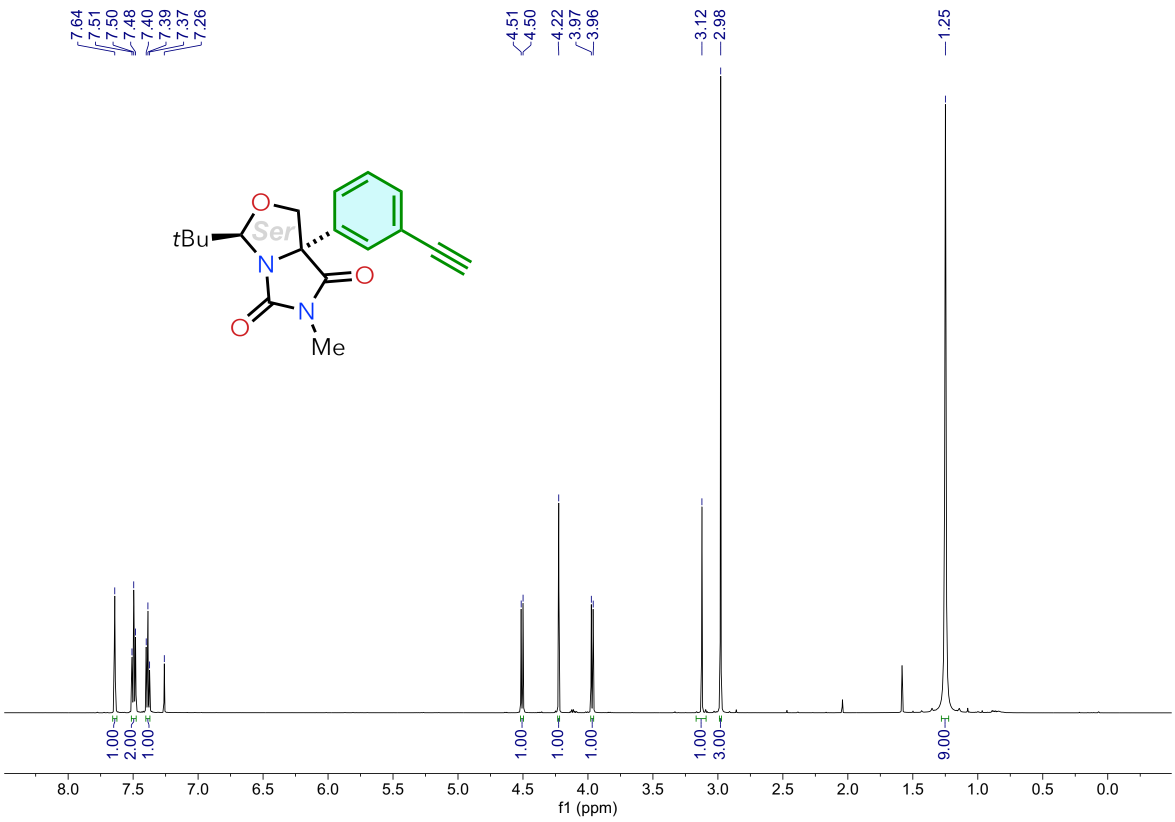
7.64
7.51
7.50
7.48
7.40
7.39
7.37
7.26
4.51
4.50
4.22
3.97
3.96
3.12
2.98
1.25
tBu
Ser
O
N
N
O
O
Me
1.00
2.00
1.00
1.00
1.00
1.00
1.00
3.00
9.00
8.0
7.5
7.0
6.5
6.0
5.5
5.0
4.5
4.0
3.5
3.0
2.5
2.0
1.5
1.0
0.5
0.0
f1 (ppm)

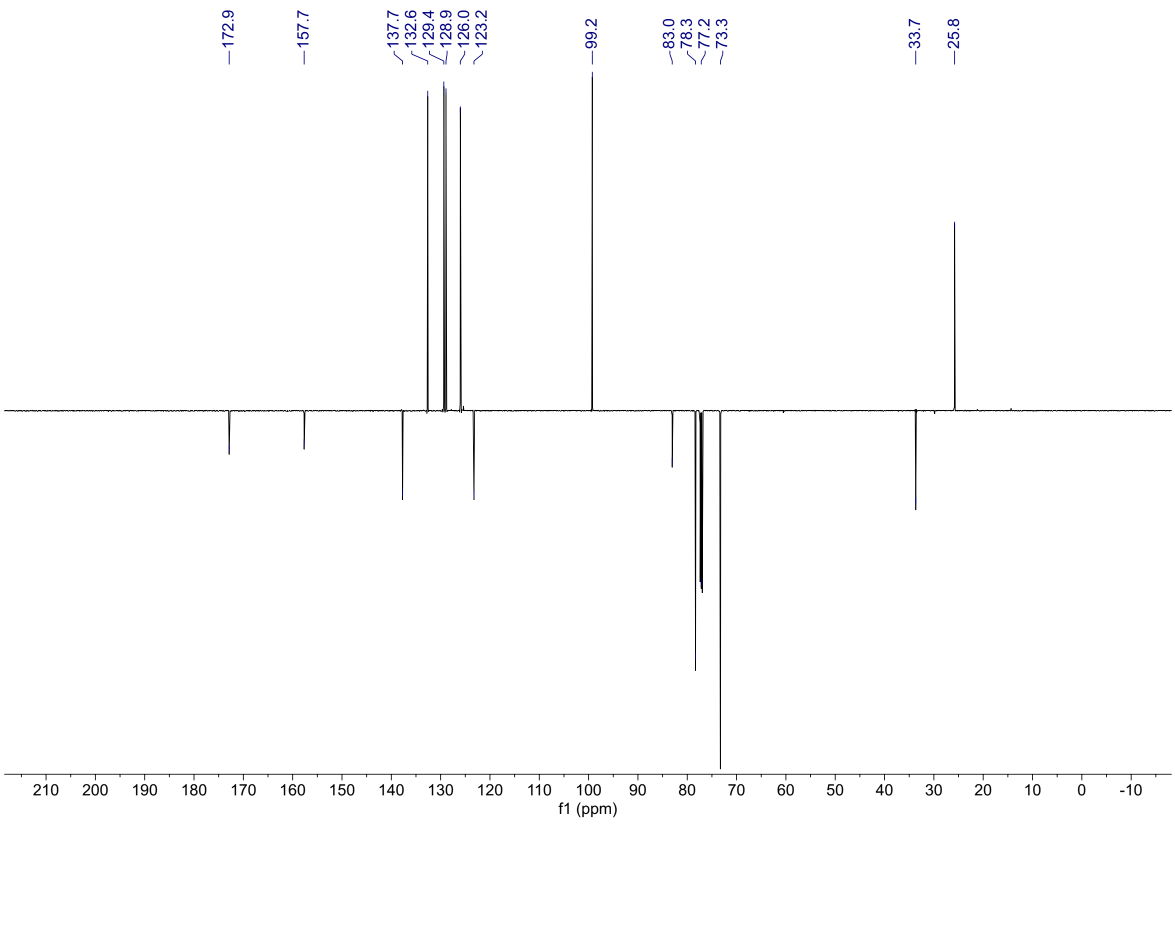
172.9
157.7
137.7
132.6
129.4
128.9
126.0
123.2
99.2
83.0
78.3
77.2
73.3
33.7
25.8
210
200
190
180
170
160
150
140
130
120
110
100
90
80
70
60
50
40
30
20
10
0
-10
f1 (ppm)

Methyl (3*R*,10b*S*)-3-(*tert*-butyl)-6-methyl-7-nitro-5-oxo-5,6-dihydro-3*H*-oxazolo[3,4-*c*]quinazoline-10b(1*H*)-carboxylate (5j)

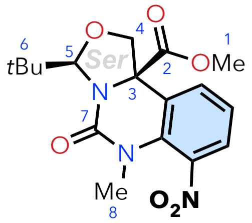

5j

Following **GP4**, to a solution of *N*-aryl urea **4j** (100 mg, 0.30 mmol, 1.0 equiv.) in dry THF (3.0 mL, 0.1 M) at –78 °C was added potassium bis(trimethylsilyl)amide solution (0.45 mL, 0.45 mmol, 1 M in THF, 1.5 equiv.) dropwise. The title compound **5j** was obtained as an orange solid (20 mg, 0.05 mmol, 18%) after purification by silica gel column chromatography (gradient elution, PE/EA).

Formula: $C_{17}H_{21}N_3O_6$, **MW:** 363.37 g/mol, **m.p.:** 196 – 199 °C. **TLC:** R_f = 0.70 (PE/EA 2:1), $KMnO_4$ stain. **^{1}H NMR** (600 MHz, $CDCl_3$): δ [ppm] = 7.83 (d, *J* 8.4 Hz, 1H, Ar), 7.71 (s, 1H, Ar), 7.22 (d, *J* 8.4 Hz, 1H, Ar), 5.28 (s, 1H, H-5), 5.22 (d, *J* 8.2 Hz, 1H, H-4a), 3.82 (d, *J* 8.2 Hz, 1H, H-4b), 3.69 (s, 3H, H-1, OMe), 3.39 (s, 3H, H-8, NMe), 0.99 (s, 9H, H-6, *t*Bu). **^{13}C NMR** (150 MHz, $CDCl_3$): δ [ppm] = 170.6 (s, C-2, ester), 151.1 (s, C-7, urea), 149.3 (s, Ar), 139.2 (s, C-Ar), 126.4 (d, Ar), 124.3 (s, Ar), 117.0 (d, Ar), 108.2 (d, Ar), 100.5 (d, C-5), 74.6 (t, C-4), 68.7 (s, C-3), 53.6 (q, C-1, OMe), 37.5 (s, C-6, *t*Bu), 31.1 (q, C-8, NMe), 26.0 (q, C-6, *t*Bu). **FT-IR (ATR):** $\tilde{\nu}$ [cm^{-1}] = 2957 (br w), 1743 (s), 1674 (vs), 1530 (vs), 1463 (w), 1436 (w), 1352 (vs), 1309 (m), 1270 (m), 1229 (s), 1207 (m), 1160 (s), 1115 (m), 1058 (m), 1041 (w), 975 (m), 937 (m), 833 (w), 768 (m), 738 (m), 729 (m), 692 (w), 633 (w). **HR-MS:** (ESI) = *m*/*z* calcd. for: $C_{17}H_{22}N_3O_6$ $[M+H]^+$ 364.1509 u, found: 364.1508 u. **$[a]^T_\lambda$:** (c = 0.98 g/100 mL, $CHCl_3$) = $[a]^{20}_D$: +12.65°.

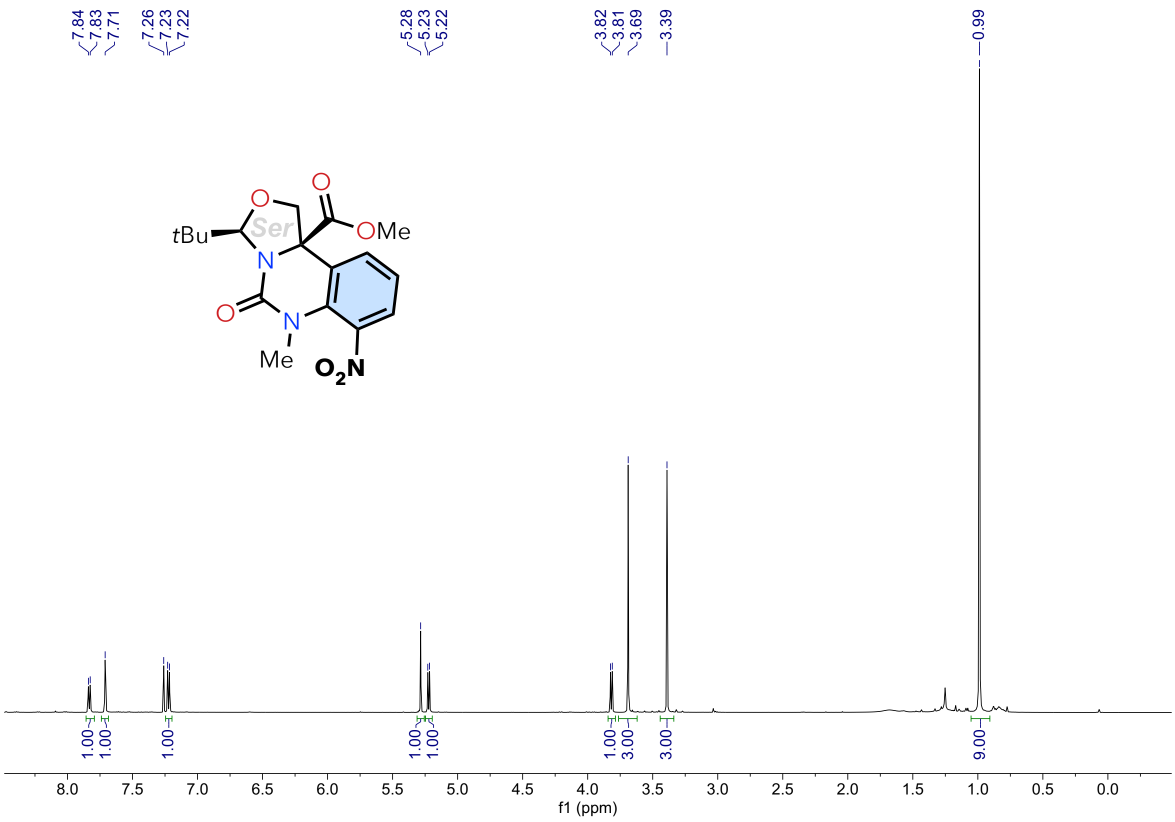
7.84
7.83
7.71
7.26
7.23
7.22
5.28
5.23
5.22
3.82
3.81
3.69
3.39
0.99
tBu
Ser
O
N
OMe
Me
O_2N
1.00
1.00
1.00
1.00
1.00
1.00
3.00
3.00
9.00
8.0
7.5
7.0
6.5
6.0
5.5
5.0
4.5
4.0
3.5
3.0
2.5
2.0
1.5
1.0
0.5
0.0
f1 (ppm)

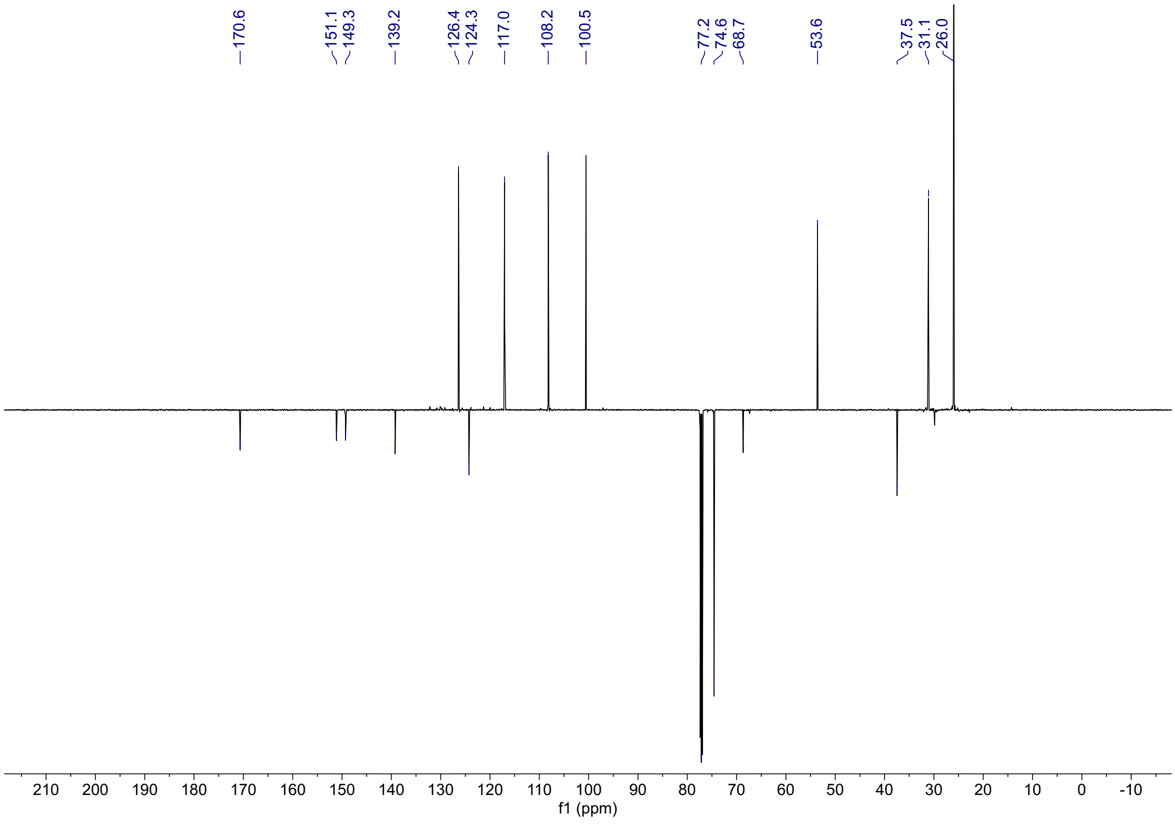
170.6
151.1
149.3
139.2
126.4
124.3
117.0
108.2
100.5
77.2
74.6
68.7
53.6
37.5
31.1
26.0
210
200
190
180
170
160
150
140
130
120
110
100
90
80
70
60
50
40
30
20
10
0
-10
f1 (ppm)

(3*R*,7a*S*)-3-(*tert*-Butyl)-7a-(3-methoxyphenyl)-6-methyldihydro-3*H*,5*H*-imidazo[1,5-*c*]oxazole-5,7(6*H*)-dione (5k)

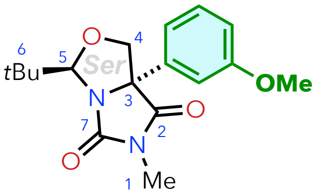

5k

Following **GP4**, to a solution of *N*-aryl urea **4k** (100 mg, 0.29 mmol, 1.0 equiv.) in dry THF (2.9 mL, 0.1 M) at –78 °C was added potassium bis(trimethylsilyl)amide solution (0.43 mL, 0.43 mmol, 1 M in THF, 1.5 equiv.) dropwise. The title compound **5k** was obtained as a colourless oil (72 mg, 0.23 mmol, 78%) after purification by silica gel column chromatography (gradient elution, PE/EA).

Formula: $C_{17}H_{22}N_2O_4$, **MW:** 318.37 g/mol. **TLC:** R_f = 0.65 (PE/EA 2:1), $KMnO_4$ stain. ^{1}**H NMR** (600 MHz, $CDCl_3$): δ [ppm] = 7.34 (t, *J* 8.0 Hz, 1H, Ar), 7.10 (d, *J* 7.8 Hz, 1H, Ar), 7.06 (s, 1H, Ar), 6.89 (dd, *J* 8.2, 2.5 Hz, 1H, Ar), 4.49 (d, *J* 8.6 Hz, 1H, H-4a), 4.25 (s, 1H, H-5), 4.00 (d, *J* 8.6 Hz, 1H, H-4b), 3.83 (s, 3H, H-1, Me), 2.97 (s, 3H, Ar, OMe), 1.25 (s, 9H, *t*Bu). ^{13}**C NMR** (150 MHz, $CDCl_3$): δ [ppm] = 173.1 (s, C-2, amide), 160.3 (s, Ar), 157.7 (s, C-7, urea), 138.9 (s, Ar), 130.4 (d, Ar), 117.6 (d, Ar), 114.1 (d, Ar), 111.3 (d, Ar), 99.0 (d, C-5), 73.5 (s, C-3), 73.3 (t, C-4), 55.5 (q, Ar, OMe), 33.6 (s, *t*Bu), 25.7 (q, C-1, Me). **FT-IR (ATR):** $\tilde{\nu}$ [cm^{-1}] = 3244 (br w), 2959 (br w), 1783 (w), 1713 (vs), 1601 (w), 1437 (m), 1390 (m), 1287 (m), 1270 (m), 1198 (m), 1170 (w), 1145 (w), 1046 (m), 1031 (m), 943 (m), 779 (m), 692 (m), 637 (w), 567 (w), 459 (w). **HR-MS:** (ESI) = *m*/*z* calcd. for: $C_{17}H_{23}N_2O_4$ $[M+H]^+$ 319.1658 u, found: 319.1647 u. $[\mathbf{a}]_{\lambda}^{\mathbf{T}}$**:** (c = 1.10 g/100 mL, $CHCl_3$) = $[a]_D^{20}$: –10.91°.

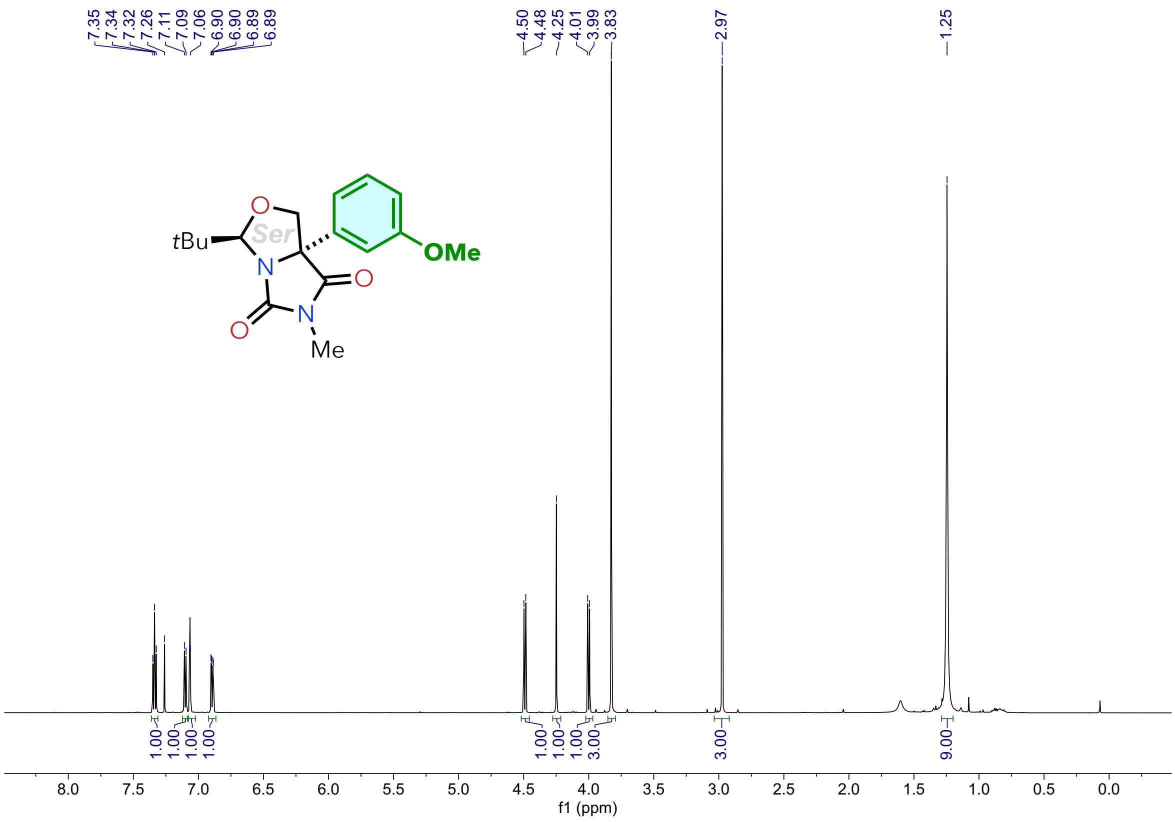
7.35
7.34
7.32
7.26
7.11
7.09
7.06
6.90
6.90
6.89
6.89
4.50
4.48
4.25
4.01
3.99
3.83
2.97
1.25
tBu
O
Ser
N
O
N
Me
O
OMe
1.00
1.00
1.00
1.00
1.00
1.00
1.00
3.00
3.00
9.00
8.0
7.5
7.0
6.5
6.0
5.5
5.0
4.5
4.0
3.5
3.0
2.5
2.0
1.5
1.0
0.5
0.0
f1 (ppm)

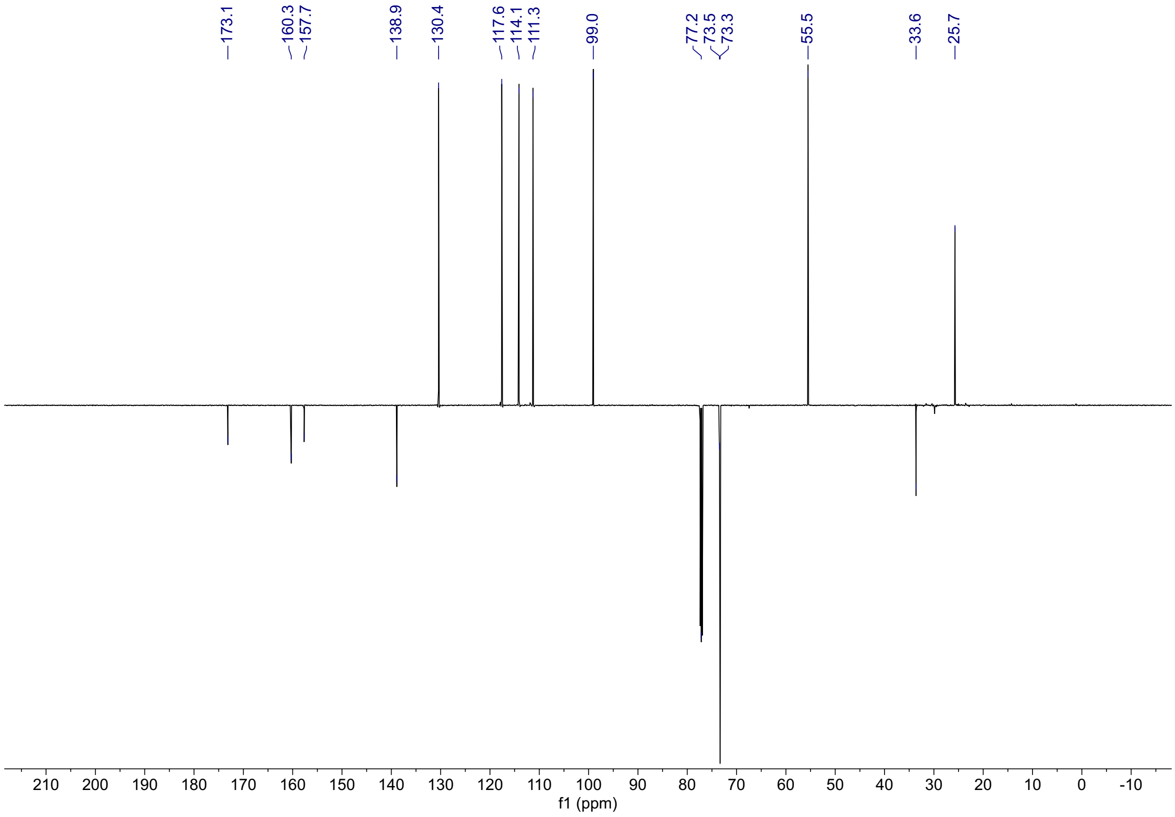
173.1
160.3
157.7
138.9
130.4
117.6
114.1
111.3
99.0
77.2
73.5
73.3
55.5
33.6
25.7
210
200
190
180
170
160
150
140
130
120
110
100
90
80
70
60
50
40
30
20
10
0
-10
f1 (ppm)

(3*R*,7a*S*)-3-(*tert*-Butyl)-7a-(2-methoxyphenyl)-6-methyldihydro-3*H*,5*H*-imidazo[1,5-*c*]oxazole-5,7(6*H*)-dione (51)

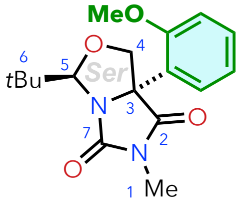

51

Following **GP4**, to a solution of *N*-aryl urea **41** (100 mg, 0.29 mmol, 1.0 equiv.) in dry THF (2.9 mL, 0.1 M) at –78 °C was added potassium bis(trimethylsilyl)amide solution (0.43 mL, 0.43 mmol, 1 M in THF, 1.5 equiv.) dropwise. The title compound **51** was obtained as a colourless oil (42 mg, 0.13 mmol, 46%) after purification by silica gel column chromatography (gradient elution, PE/EA).

Formula: $C_{17}H_{22}N_2O_4$, **MW:** 318.37 g/mol. **TLC:** R_f = 0.50 (PE/EA 2:1), $KMnO_4$ stain. **^{1}H NMR** (500 MHz, $CDCl_3$): δ [ppm] = 7.47 (dd, *J* 8.0, 1.7 Hz, 1H, Ar), 7.40 – 7.31 (m, 1H, Ar), 6.99 (dd, *J* 7.8 Hz, 2H, Ar), 4.40 (d, *J* 9.6 Hz, 1H, H-4a), 4.28 (d, *J* 9.6 Hz, 1H, H-4b), 4.09 (s, 1H, H-5), 3.88 (s, 3H, H-1, Me), 2.98 (s, 3H, Ar, OMe), 1.22 (s, 9H, *t*Bu). **^{13}C NMR** (125 MHz, $CDCl_3$): δ [ppm] = 172.9 (s, C-2, amide), 158.2 (s, Ar), 157.7 (s, C-7, urea), 130.6 (d, Ar), 126.4 (d, Ar), 125.0 (s, Ar), 112.9 (d, Ar), 97.8 (d, C-5), 72.8 (s, C-3), 71.1 (t, C-4), 56.4 (q, Ar, OMe), 33.5 (s, *t*Bu), 25.5 (q, C-1, Me). **FT-IR (ATR):** $\tilde{\nu}$ [cm^{-1}] = 3424 (br w), 2958 (br w), 1783 (w), 1714 (vs), 1599 (w), 1489 (m), 1440 (m), 1391 (m), 1365 (w), 1283 (m), 1249 (w), 1197 (w), 1141 (w), 1100 (w), 1025 (s), 947 (w), 890 (w), 755 (m), 626 (w), 471 (w). **HR-MS:** (ESI) = *m/z* calcd. for: $C_{17}H_{23}N_2O_4$ $[M+H]^+$ 319.1658 u, found: 319.1647 u. **$[a]_\lambda^T$:** (c = 1.04 g/100 mL, $CHCl_3$) = $[a]_D^{20}$: –12.31°.

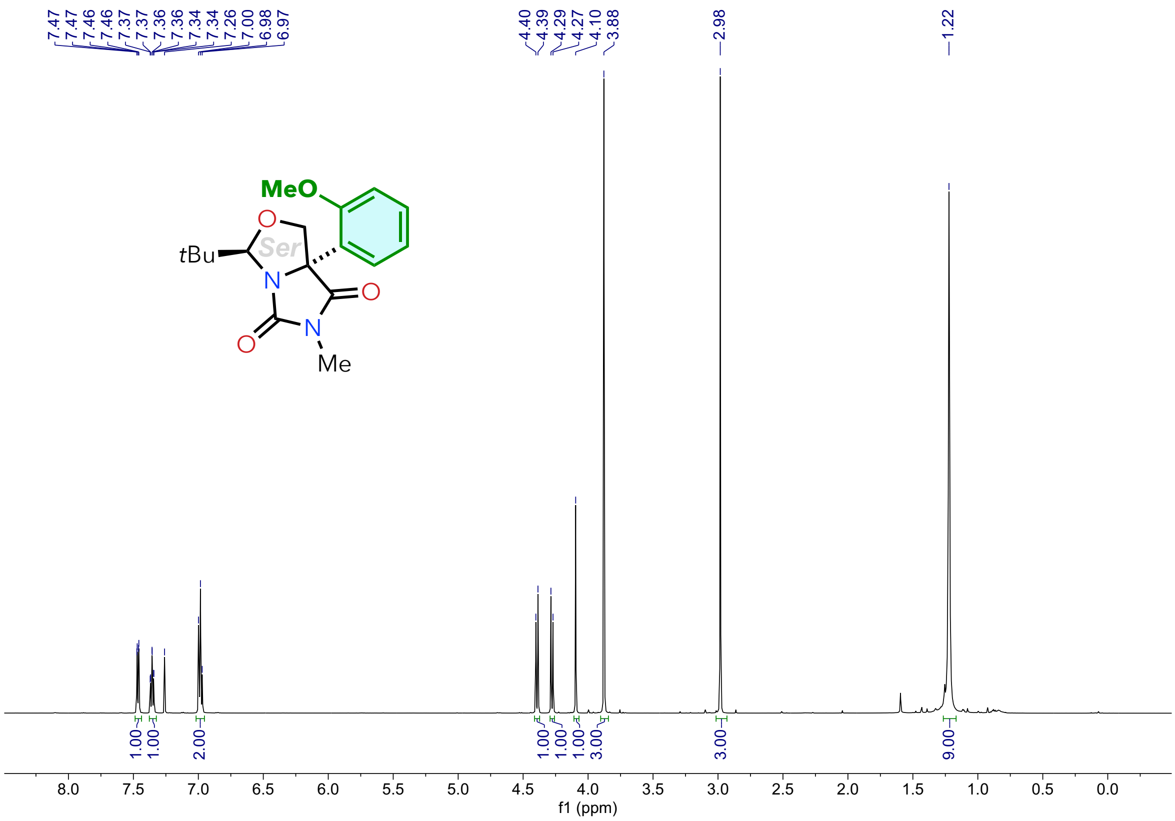

7.47
7.47
7.46
7.46
7.37
7.37
7.36
7.36
7.34
7.34
7.26
7.00
6.98
6.97
4.40
4.39
4.29
4.27
4.10
3.88
2.98
1.22
MeO
O
tBu
Ser
N
O
N
O
Me
1.00
1.00
2.00
1.00
1.00
1.00
3.00
3.00
9.00
f1 (ppm)

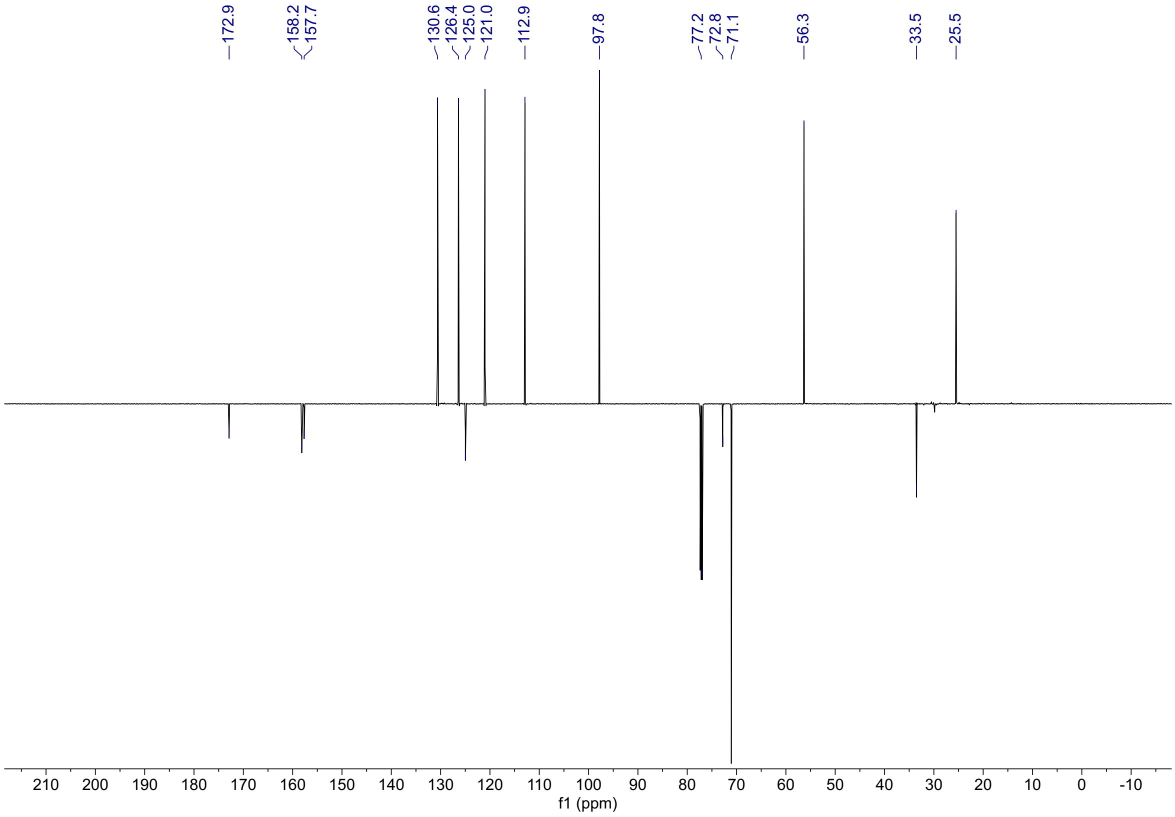

172.9
158.2
157.7
130.6
126.4
125.0
121.0
112.9
97.8
77.2
72.8
71.1
56.3
33.5
25.5
f1 (ppm)

(3*R*,7a*S*)-3-(*tert*-Butyl)-7a-(2,4-dichlorophenyl)-6-methyldihydro-3*H*,5*H*-imidazo[1,5-*c*]oxazole-5,7(6*H*)-dione (5m)

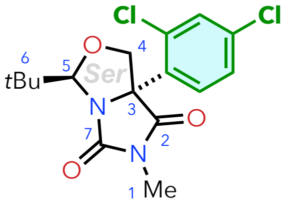

5m

Following **GP4**, to a solution of *N*-aryl urea **4m** (100 mg, 0.26 mmol, 1.0 equiv.) in dry THF (2.6 mL, 0.1 M) at –78 °C was added potassium bis(trimethylsilyl)amide solution (0.39 mL, 0.39 mmol, 1 M in THF, 1.5 equiv.) dropwise. The title compound **5m** was obtained as a white solid (54 mg, 0.15 mmol, 58%) after purification by silica gel column chromatography (gradient elution, PE/EA).

Formula: $C_{16}H_{18}Cl_2N_2O_3$, **MW:** 357.23 g/mol, **m.p.:** 147 – 150 °C. **TLC:** R_f = 0.66 (PE/EA 2:1), $KMnO_4$ stain. **^{1}H NMR** (600 MHz, $CHCl_3$): δ [ppm] = 7.56 (d, *J* 8.5 Hz, 1H, Ar), 7.50 (d, *J* 2.1 Hz, 1H, Ar), 7.21 (dd, *J* 8.5, 2.1 Hz, 1H, Ar), 4.64 (d, *J* 9.6 Hz, 1H, H-4a), 4.28 (d, *J* 9.6 Hz, 1H, H-4b), 4.00 (s, 1H, H-5), 2.97 (s, 3H, H-1, Me), 1.23 (s, 9H, H-6, *t*Bu). **^{13}C NMR** (150 MHz, $CHCl_3$): δ [ppm] = 171.5 (s, C-2), 157.5 (s, C-7), 135.8 (s, Ar), 134.4 (s, Ar), 133.4 (s, Ar), 132.0 (d, Ar), 128.2 (d, Ar), 127.6 (d, Ar), 98.4 (d, C-5), 73.5 (s, C-3), 71.9 (t, C-4), 33.4 (s, *t*Bu), 25.7 (q, C-1, Me). **FT-IR (ATR):** $\tilde{\nu}$ [cm^{-1}] = 2923 (br w), 1783 (w), 1718 (vs), 1585 (w), 1557 (w), 1440 (s), 1380 (m), 1287 (m), 1193 (m), 1136 (w), 1102 (m), 1069 (s), 1047 (s), 948 (m), 897 (w), 881 (m), 828 (w), 809 (m), 792 (m), 754 (m), 688 (m), 589 (m), 560 (m), 500 (w), 460 (m). **HR-MS:** (ESI) = *m*/*z* calcd. for: $C_{16}H_{19}{}^{35}Cl_2N_2O_3$ $[M+H]^+$ 357.0773 u, found: 357.0773 u. **$[a]_\lambda^T$:** (c = 1.02 g/100 mL, $CHCl_3$) = $[a]_D^{20}$: –17.25°.

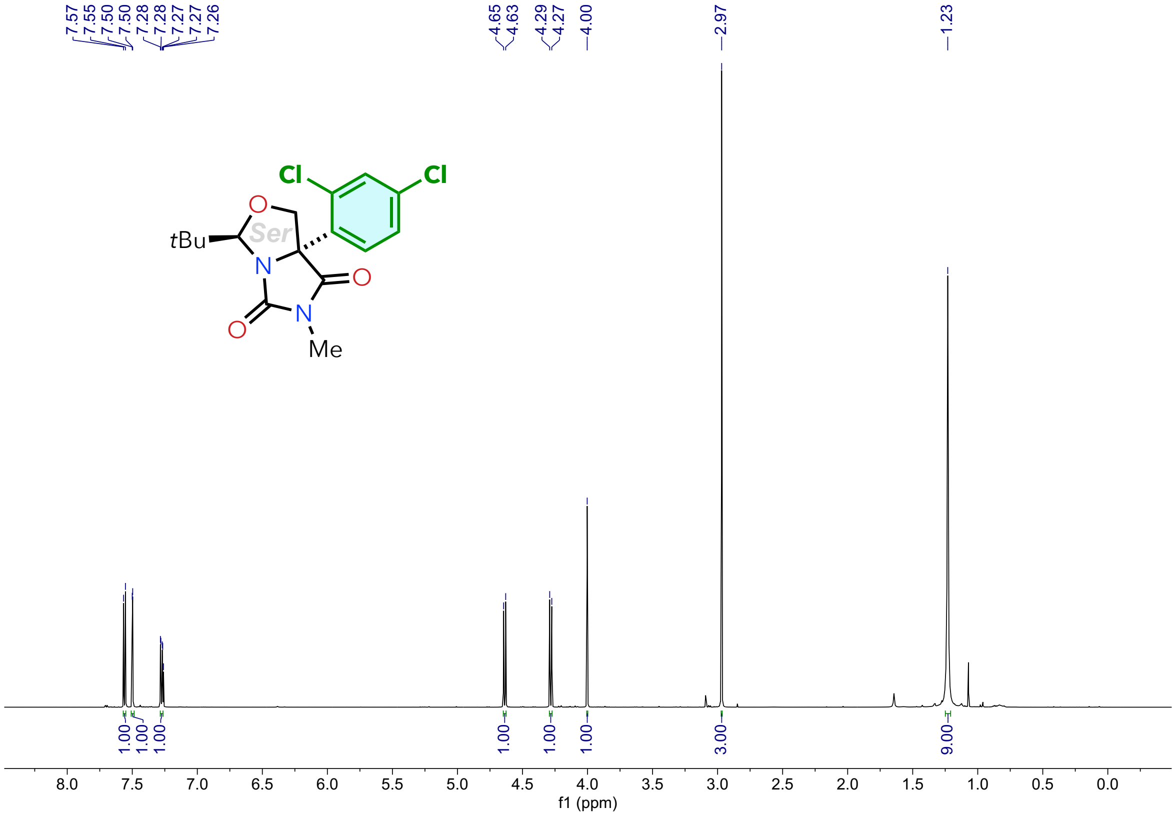
7.57
7.55
7.50
7.50
7.28
7.28
7.27
7.27
7.26
4.65
4.63
4.29
4.27
4.00
2.97
1.23
Cl
Cl
O
tBu
Ser
N
O
N
O
Me
1.00
1.00
1.00
1.00
1.00
1.00
3.00
9.00
8.0 7.5 7.0 6.5 6.0 5.5 5.0 4.5 4.0 3.5 3.0 2.5 2.0 1.5 1.0 0.5 0.0
f1 (ppm)

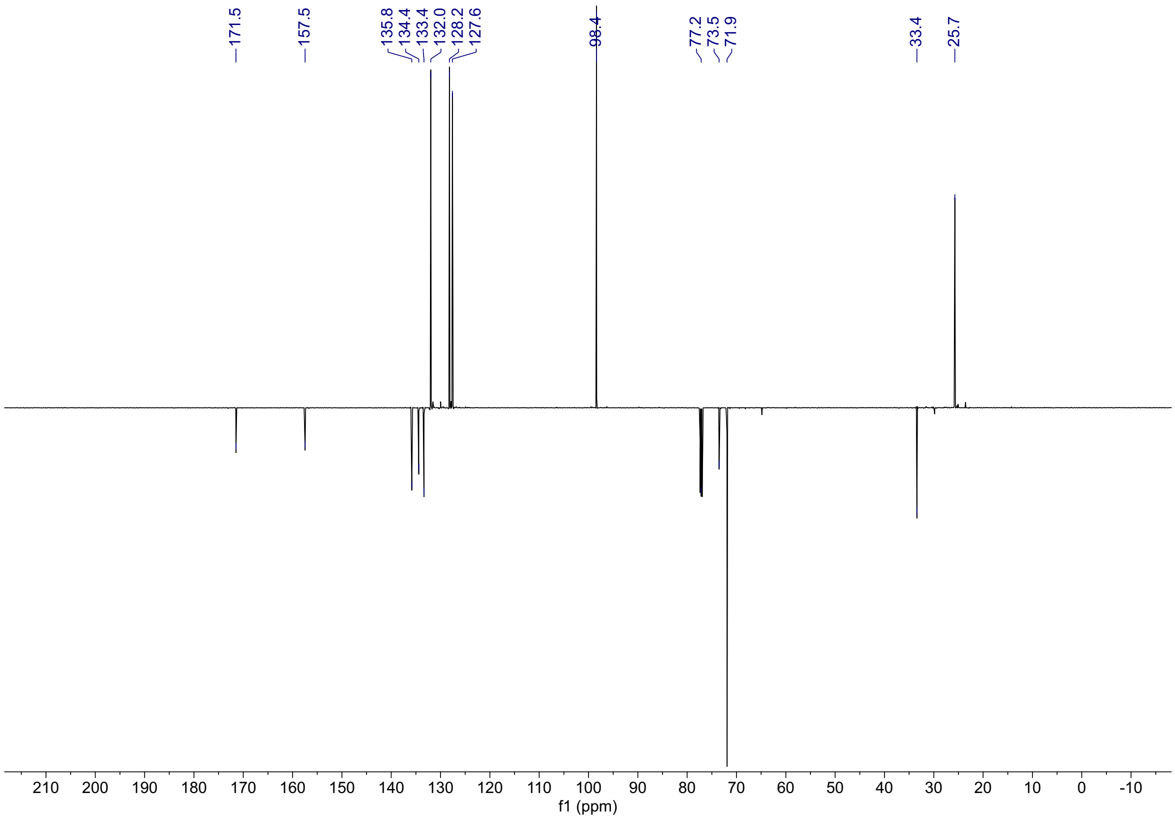
171.5
157.5
135.8
134.4
133.4
132.0
128.2
127.6
98.4
77.2
73.5
71.9
33.4
25.7
210 200 190 180 170 160 150 140 130 120 110 100 90 80 70 60 50 40 30 20 10 0 -10
f1 (ppm)

(3*R*,7a*S*)-3-(*tert*-Butyl)-7a-(3,5-dichlorophenyl)-6-methyldihydro-3*H*,5*H*-imidazo[1,5-*c*]oxazole-5,7(6*H*)-dione (5n)

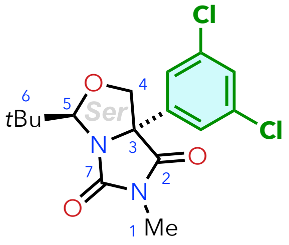

5n

Following **GP4**, to a solution of *N*-aryl urea **4n** (100 mg, 0.26 mmol, 1.0 equiv.) in dry THF (2.6 mL, 0.1 M) at –78 °C was added potassium bis(trimethylsilyl)amide solution (0.39 mL, 0.39 mmol, 1 M in THF, 1.5 equiv.) dropwise. The title compound **5n** was obtained as a colourless oil (67 mg, 0.19 mmol, 72%) after purification by silica gel column chromatography (gradient elution, PE/EA).

Formula: $C_{16}H_{18}Cl_2N_2O_3$, **MW:** 357.23 g/mol. **TLC:** R_f = 0.70 (PE/EA 2:1), $KMnO_4$ stain. **^{1}H NMR** (600 MHz, $CHCl_3$): δ [ppm] = 7.40 (d, *J* 1.9 Hz, 2H, Ar), 7.35 (t, *J* 1.9 Hz, 1H, Ar), 4.50 (d, *J* 8.8 Hz, 1H, H-4a), 4.19 (s, 1H, H-5), 3.90 (d, *J* 8.8 Hz, 1H, H-4b), 2.98 (s, 3H, H-1, Me), 1.24 (s, 9H, H-6, *t*Bu). **^{13}C NMR** (150 MHz, $CHCl_3$): δ [ppm] = 172.2 (s, C-2), 157.6 (s, C-7), 140.7 (s, Ar), 136.1 (s, Ar), 129.3 (d, Ar), 124.1 (d, 2Ar), 99.7 (d, C-5), 73.2 (t, C-4), 72.9 (s, C-3), 33.7 (s, *t*Bu), 25.9 (q, C-1, Me). **FT-IR (ATR):** $\tilde{\nu}$ [cm^{-1}] = 2959 (br w), 1788 (w), 1718 (vs), 1588 (w), 1568 (m), 1441 (m), 1390 (m), 1367 (w), 1283 (m), 1194 (w), 1143 (w), 1069 (m), 1055 (m), 943 (w), 862 (w), 799 (m), 769 (w), 707 (w), 672 (m), 578 (w). **HR-MS:** (ESI) = *m*/*z* calcd. for: $C_{15}H_{15}{}^{35}Cl_2N_2O_3$ [M–Me]$^+$ 341.0460 u, found: 341.0448 u. **$[a]_\lambda^T$:** (c = 1.00 g/100 mL, $CHCl_3$) = $[a]_D^{20}$: –18.40°.

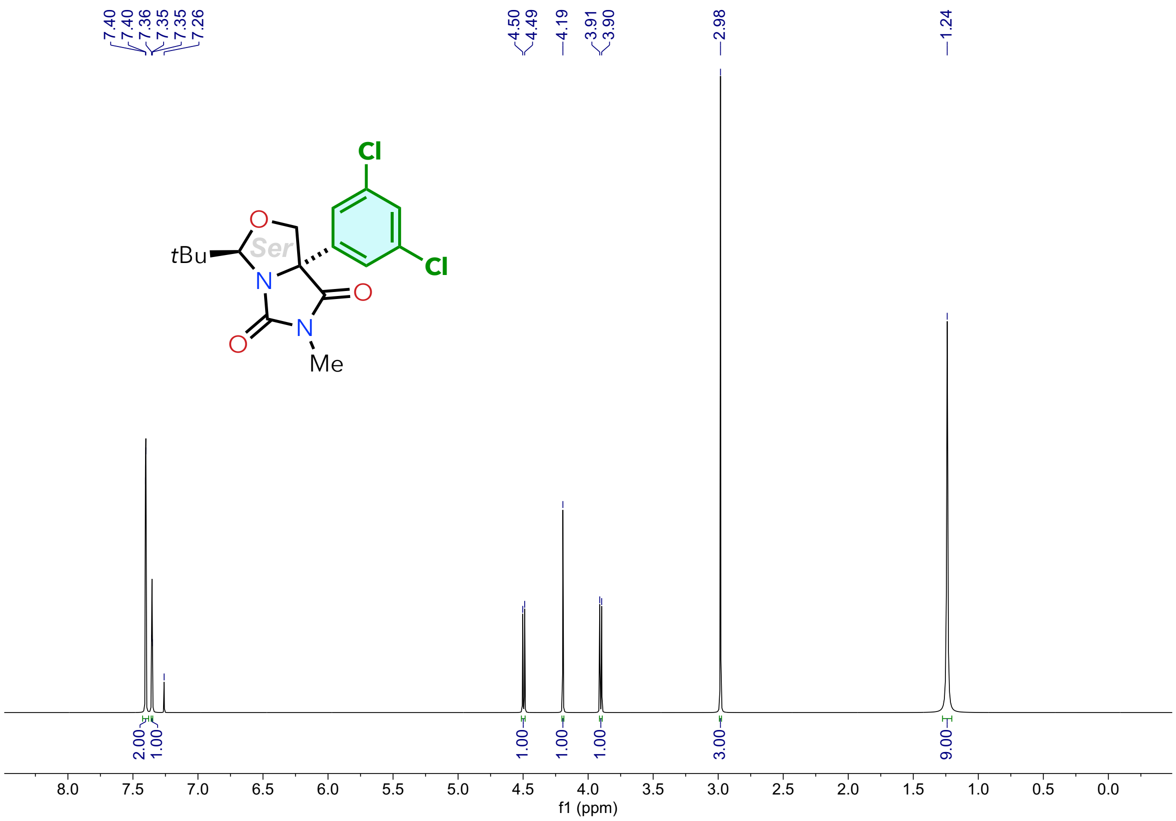
7.40
7.40
7.36
7.35
7.35
7.26
4.50
4.49
4.19
3.91
3.90
2.98
1.24
Cl
Cl
O
tBu
Ser
N
O
N
O
Me
2.00
1.00
1.00
1.00
1.00
3.00
9.00
8.0 7.5 7.0 6.5 6.0 5.5 5.0 4.5 4.0 3.5 3.0 2.5 2.0 1.5 1.0 0.5 0.0
f1 (ppm)

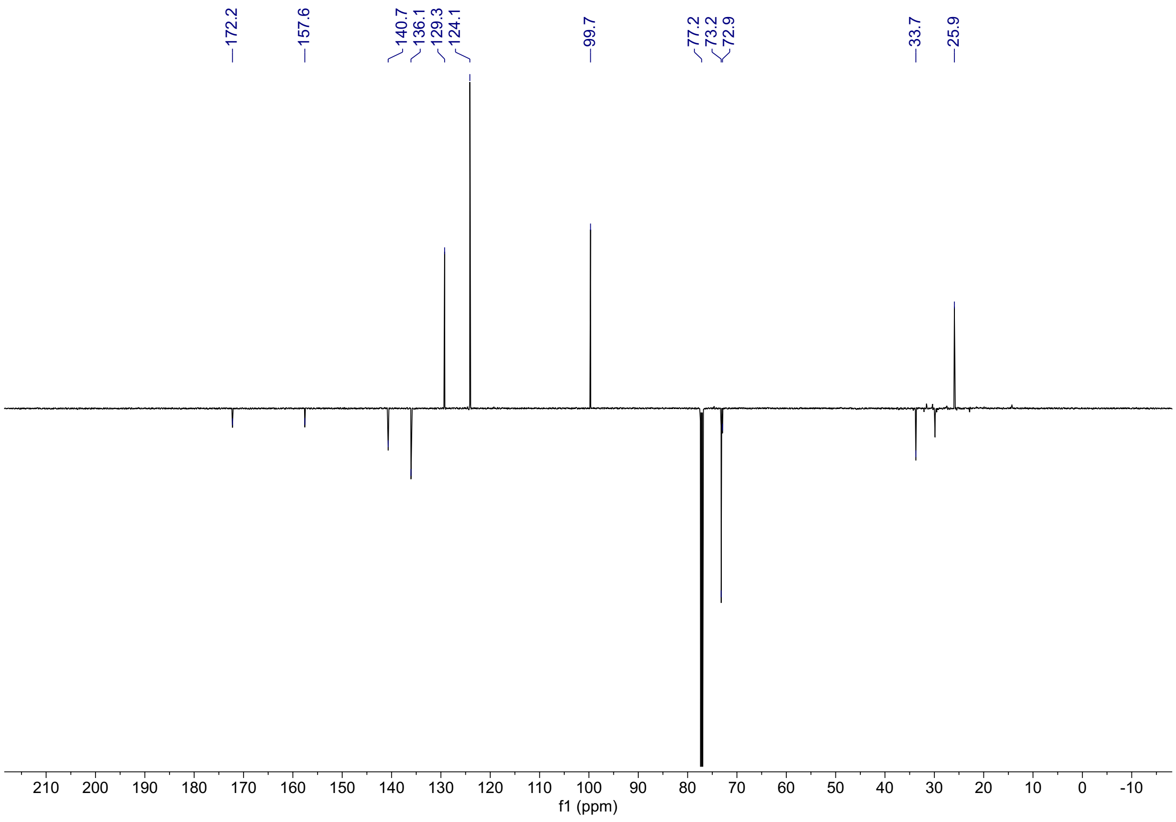
172.2
157.6
140.7
136.1
129.3
124.1
99.7
77.2
73.2
72.9
33.7
25.9
210 200 190 180 170 160 150 140 130 120 110 100 90 80 70 60 50 40 30 20 10 0 -10
f1 (ppm)

(3*R*,7a*S*)-7a-(4-Bromo-3-fluorophenyl)-3-(*tert*-butyl)-6-methyldihydro-3*H*,5*H*-imidazo[1,5-*c*]oxazole-5,7(6*H*)-dione (5o)

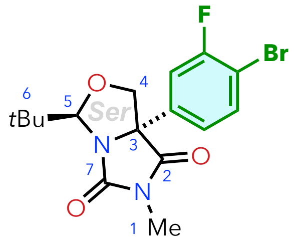

5o

Following **GP4**, to a solution of *N*-aryl urea **4o** (100 mg, 0.24 mmol, 1.0 equiv.) in dry THF (2.4 mL, 0.1 M) at –78 °C was added potassium bis(trimethylsilyl)amide solution (0.36 mL, 0.36 mmol, 1 M in THF, 1.5 equiv.) dropwise. The title compound **5o** was obtained as a white solid (65 mg, 0.17 mmol, 70%) after purification by silica gel column chromatography (gradient elution, PE/EA).

Formula: $C_{16}H_{18}BrFN_2O_3$, **MW:** 385.23 g/mol, **m.p.:** 142 – 145 °C. **TLC:** R_f = 0.64 (PE/EA 2:1), $KMnO_4$ stain. **^{1}H NMR** (600 MHz, $CHCl_3$): δ [ppm] = 7.58 (t, *J* 7.5 Hz, 1H, Ar), 7.29 (dd, *J* 9.3, 2.1 Hz, 1H, Ar), 7.20 (dd, *J* 8.4, 2.1 Hz, 1H, Ar), 4.48 (d, *J* 8.7 Hz, 1H, H-4a), 4.19 (s, 1H, H-5), 3.90 (d, *J* 8.7 Hz, 1H, H-4b), 2.96 (s, 3H, H-1, Me), 1.22 (s, 9H, H-6, *t*Bu). **^{13}C NMR** (150 MHz, $CHCl_3$): δ [ppm] = 172.2 (s, C-2), 160.2 (s, Ar), 158.6 (s, Ar), 157.5 (s, C-7), 139.0 (s, *d J* 6.4 Hz, Ar), 134.4 (d, Ar), 122.3 (d, *d J* 3.5 Hz, Ar), 113.8 (d, *d J* 24.4 Hz, Ar), 109.7 (d, *d J* 21.0 Hz, Ar), 99.4 (d, C-5), 73.2 (t, C-4), 72.8 (s, C-3), 33.6 (s, *t*Bu), 25.8 (q, C-1, Me). **^{19}F NMR** (565 MHz, $CDCl_3$, C_6F_6 ref.): δ [ppm] = –108.17 (m, F). **FT-IR (ATR):** $\tilde{\nu}$ [cm^{-1}] = 2957 (br w), 1783 (m), 1712 (vs), 1577 (w), 1438 (m), 1415 (m), 1393 (m), 1286 (m), 1197 (m), 1177 (m), 1138 (w), 1054 (s), 934 (s), 887 (w), 877 (w), 819 (m), 782 (m), 732 (w), 671 (w), 636 (w), 603 (w), 572 (m), 547 (m), 508 (w), 456 (m), 422 (w). **HR-MS:** (ESI) = *m/z* calcd. for: $C_{16}H_{19}{}^{79}BrFN_2O_3$ $[M+H]^+$ 385.0563 u, found: 385.0561 u. **$[a]_{\lambda}^{T}$:** (c = 1.01 g/100 mL, $CHCl_3$) = $[a]_{D}^{20}$: –13.07°.

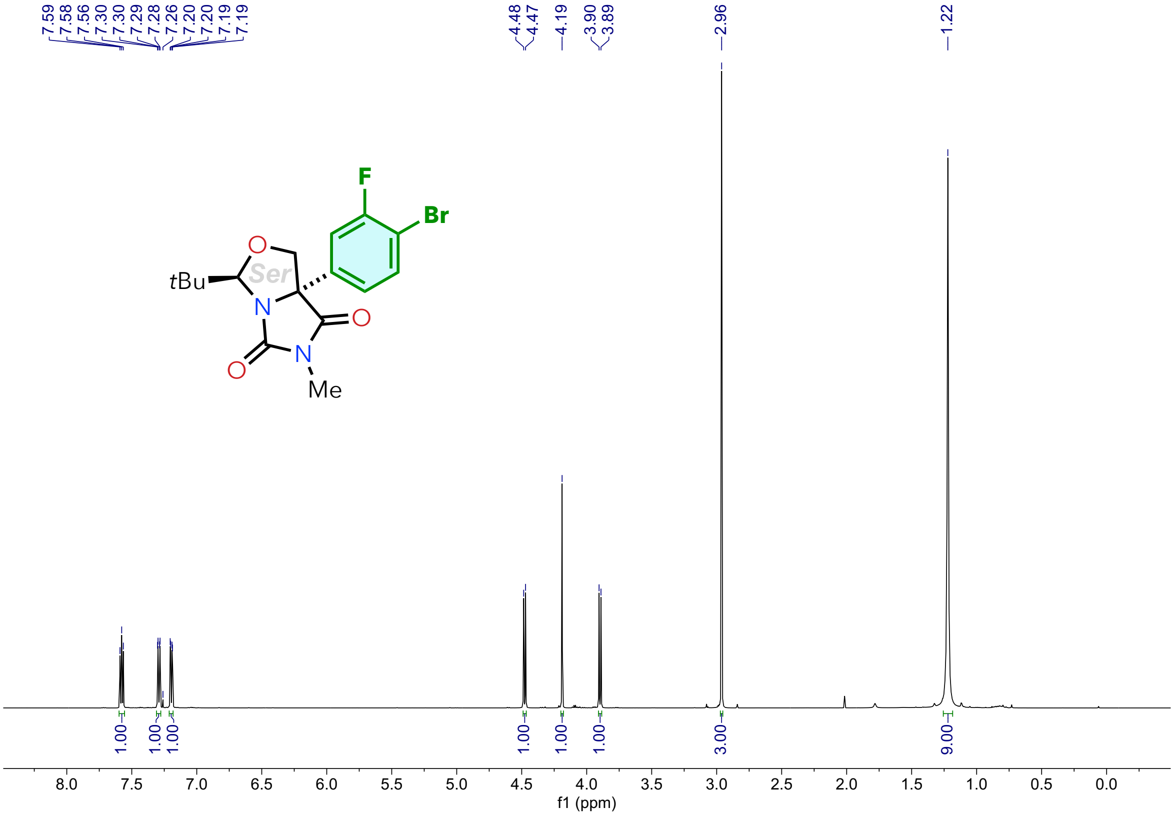
7.59
7.58
7.56
7.30
7.30
7.29
7.28
7.26
7.20
7.20
7.19
7.19
4.48
4.47
4.19
3.90
3.89
2.96
1.22
F
Br
O
tBu
Ser
N
N
O
O
Me
1.00
1.00
1.00
1.00
1.00
1.00
3.00
9.00
8.0 7.5 7.0 6.5 6.0 5.5 5.0 4.5 4.0 3.5 3.0 2.5 2.0 1.5 1.0 0.5 0.0
f1 (ppm)

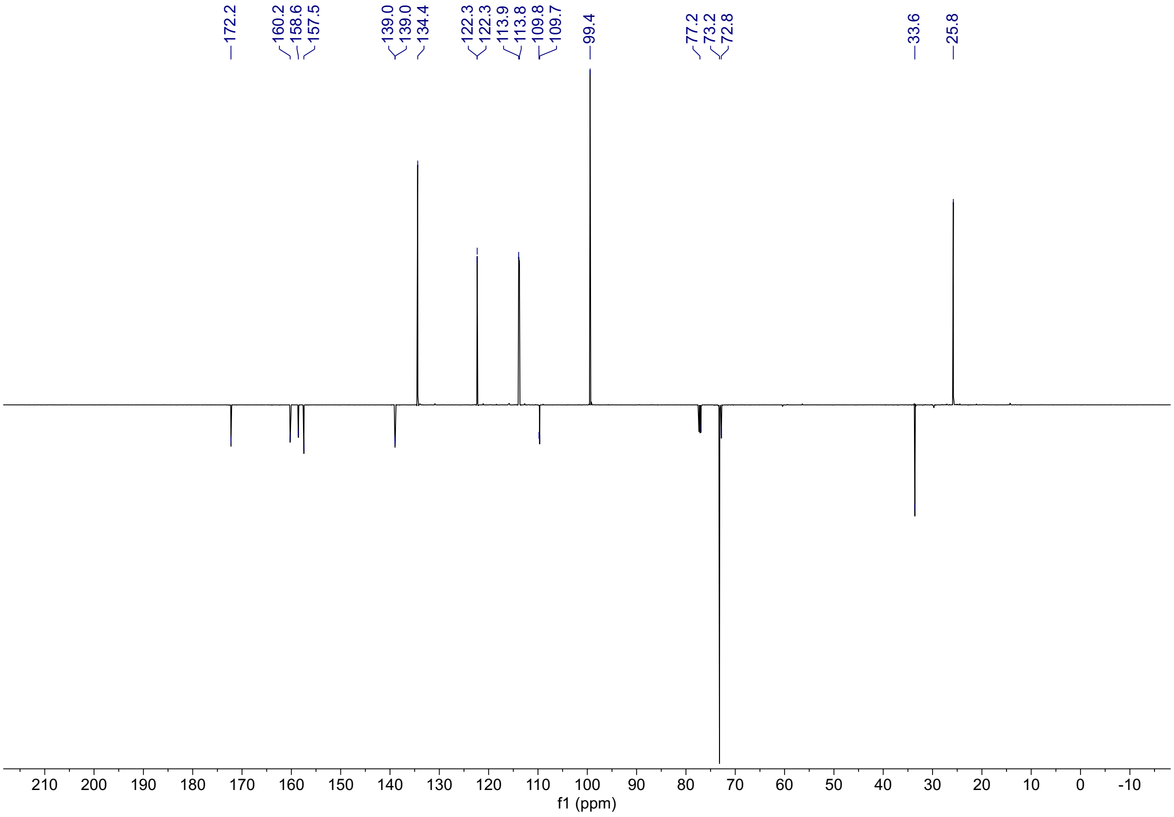
172.2
160.2
158.6
157.5
139.0
139.0
134.4
122.3
122.3
113.9
113.8
109.8
109.7
99.4
77.2
73.2
72.8
33.6
25.8
210 200 190 180 170 160 150 140 130 120 110 100 90 80 70 60 50 40 30 20 10 0 -10
f1 (ppm)

(3*R*,7a*S*)-3-(*tert*-Butyl)-7a-(4-chloro-2-methoxyphenyl)-6-methyldihydro-3*H*,5*H*-imidazo[1,5-*c*]oxazole-5,7(6*H*)-dione (5p)

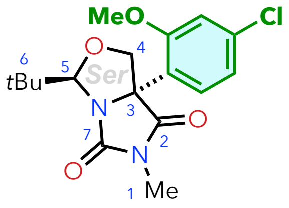

5p

Following **GP4**, to a solution of *N*-aryl urea **4p** (100 mg, 0.26 mmol, 1.0 equiv.) in dry THF (2.6 mL, 0.1 M) at –78 °C was added potassium bis(trimethylsilyl)amide solution (0.39 mL, 0.39 mmol, 1 M in THF, 1.5 equiv.) dropwise. The title compound **5p** was obtained as a white solid (70 mg, 0.20 mmol, 76%) after purification by silica gel column chromatography (gradient elution, PE/EA).

Formula: $C_{17}H_{21}ClN_2O_4$, **MW:** 352.82 g/mol, **m.p.:** 136 – 139 °C. **TLC:** R_f = 0.60 (PE/EA 2:1), $KMnO_4$ stain. **^{1}H NMR** (600 MHz, $CDCl_3$): δ [ppm] = 7.38 (d, *J* 8.8 Hz, 1H, Ar), 6.99 – 6.94 (m, 2H, Ar), 4.38 (d, *J* 9.7 Hz, 1H, H-4a), 4.21 (d, *J* 8.7 Hz, 1H, H-4b), 4.05 (s, 1H, H-5), 3.88 (s, 3H, Ar, OMe), 2.98 (s, 3H, H-1, Me), 1.21 (s, 9H, *t*Bu). **^{13}C NMR** (150 MHz, $CDCl_3$): δ [ppm] = 172.5 (s, C-2), 158.7 (s, Ar), 157.6 (s, C-7), 136.2 (s, Ar), 127.4 (d, Ar), 123.7 (s, Ar), 121.1 (d, Ar), 113.6 (d, Ar), 98.0 (d, C-5), 71.1 (t, C-4), 56.6 (q, Ar, OMe), 33.5 (s, C-6, *t*Bu), 25.6 (q, Me). **FT-IR (ATR):** $\tilde{\nu}$ [cm^{-1}] = 2956 (br w), 1774 (w), 1709 (vs), 1595 (w), 1501 (w), 1442 (m), 1403 (w), 1378 (w), 1287 (w), 1253 (m), 1204 (w), 1190 (w), 1119 (w), 1069 (w), 1036 (m), 1013 (m), 919 (w), 878 (m), 799 (m), 753 (m), 545 (w). **HR-MS:** (ESI) = *m*/*z* calcd. for: $C_{17}H_{22}{}^{35}ClN_2O_4$ $[M+H]^+$ 353.1268 u, found: 353.1251 u. **$[a]_\lambda^T$:** (c = 0.98 g/100 mL, $CDCl_3$) = $[a]_D^{20}$: –19.59°.

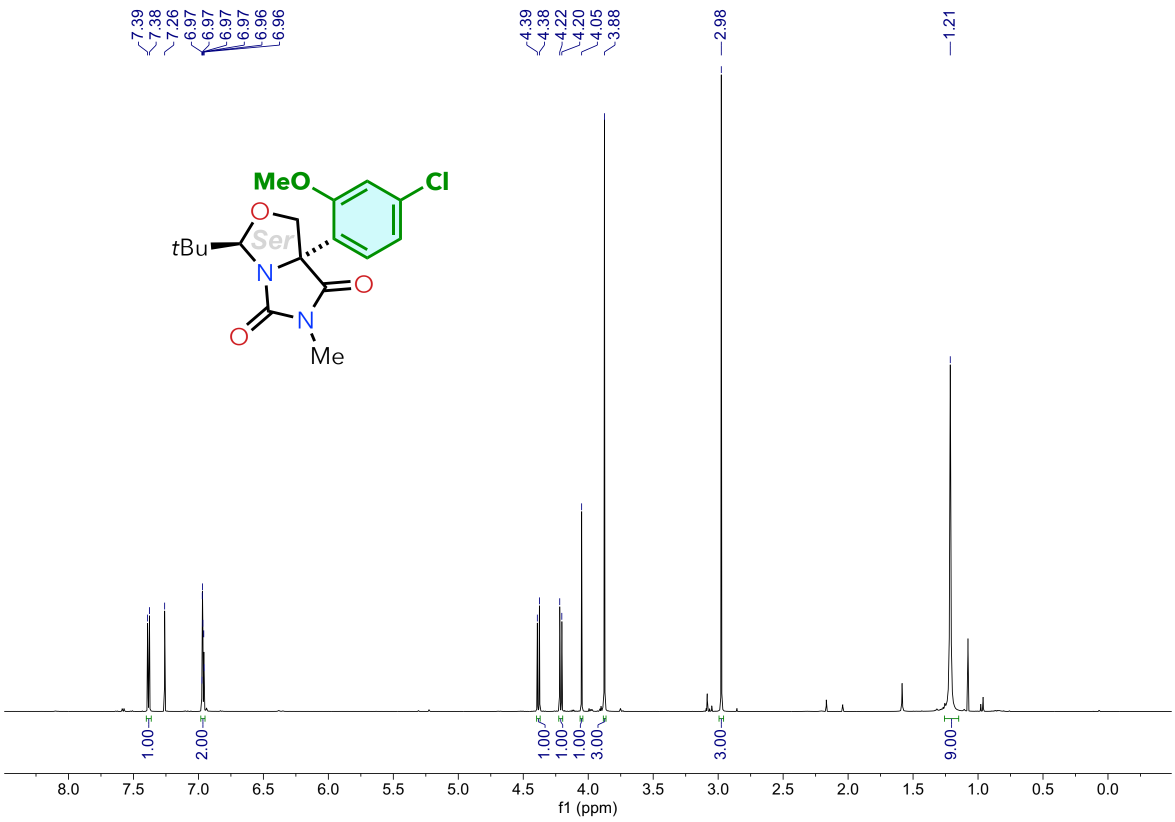
7.39
7.38
7.26
6.97
6.97
6.97
6.97
6.96
6.96
4.39
4.38
4.22
4.20
4.05
3.88
2.98
1.21
MeO
Cl
O
tBu
Ser
N
O
N
O
Me
1.00
2.00
1.00
1.00
1.00
3.00
3.00
9.00
f1 (ppm)

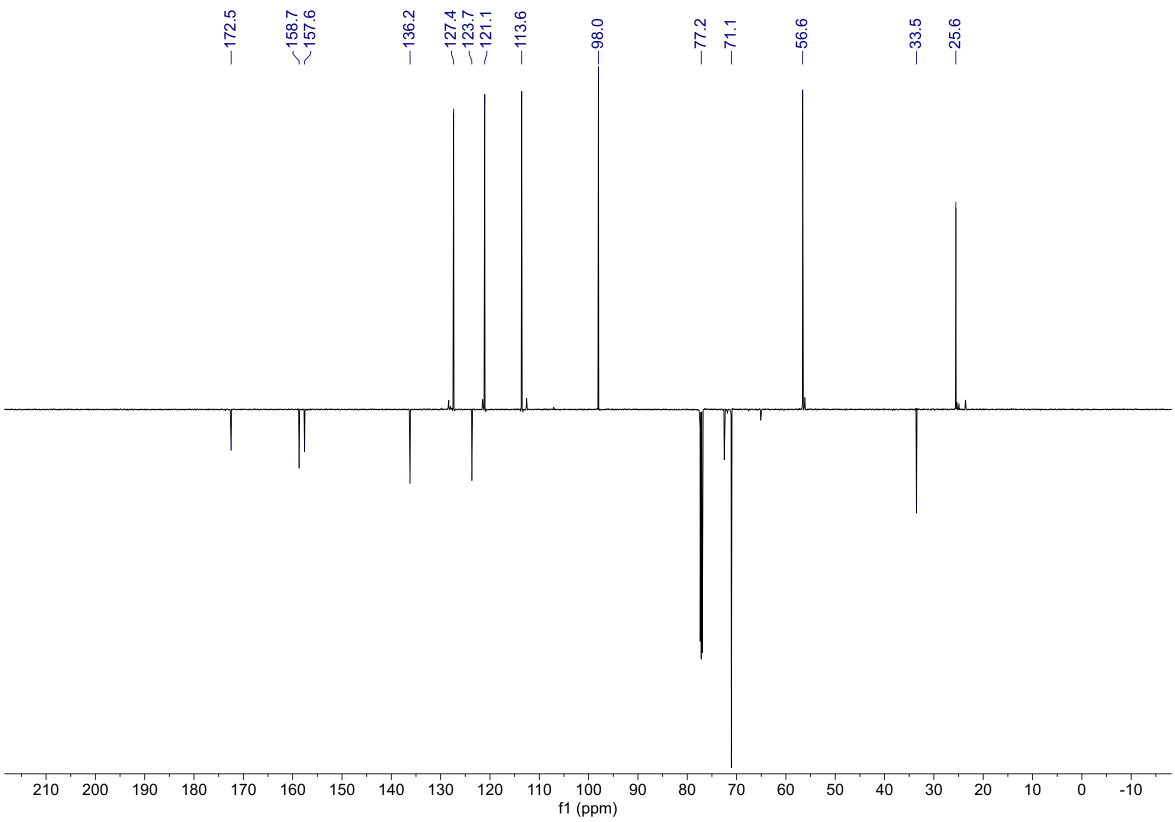
172.5
158.7
157.6
136.2
127.4
123.7
121.1
113.6
98.0
77.2
71.1
56.6
33.5
25.6
f1 (ppm)

(3*R*,7a*S*)-3-(*tert*-Butyl)-6-methyl-7a-(pyridin-2-yl)dihydro-3*H*,5*H*-imidazo[1,5-*c*]oxazole-5,7(6*H*)-dione (5q)

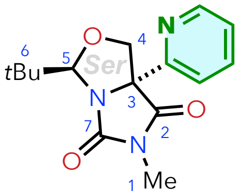

5q

Following **GP4**, to a solution of *N*-aryl urea **4q** (70 mg, 0.22 mmol, 1.0 equiv.) in dry THF (2.2 mL, 0.1 M) at –78 °C was added potassium bis(trimethylsilyl)amide solution (0.33 mL, 0.33 mmol, 1 M in THF, 1.5 equiv.) dropwise. The title compound **4q** was obtained as a white solid (32 mg, 0.11 mmol, 50%) after purification by silica gel column chromatography (gradient elution, PE/EA).

Formula: $C_{15}H_{19}N_3O_3$, **MW:** 289.34 g/mol, **m.p.:** 155 – 158 °C. **TLC:** R_f = 0.73 (PE/EA 2:1), $KMnO_4$ stain. **^{1}H NMR** (600 MHz, $CDCl_3$): δ [ppm] = 8.73 (d, *J* 4.7 Hz, 1H, Ar), 7.74 (td, *J* 7.8, 1.5 Hz, 1H, Ar), 7.53 (d, *J* 7.9 Hz, 1H, Ar), 7.30 (dd, *J* 7.5, 4.8, 1.1 Hz, 1H, Ar), 4.51 (d, *J* 9.0 Hz, 1H, H-4a), 4.46 (d, *J* 9.0 Hz, 1H, H-4b), 4.22 (s, 1H, H-5), 3.02 (s, 3H, H-1, Me), 1.23 (s, 9H, *t*Bu). **^{13}C NMR** (150 MHz, $CDCl_3$): δ [ppm] = 171.8 (s, C-2), 157.7 (s, C-7), 155.5 (s, Ar), 150.6 (d, Ar), 137.4 (d, Ar), 123.8 (d, Ar), 119.8 (d, Ar), 99.0 (d, C-5), 74.8 (s, C-3), 71.2 (t, C-4), 33.6 (s, C-6, *t*Bu), 25.8 (q, Me). **FT-IR (ATR):** $\tilde{\nu}$ [cm^{-1}] = 2970 (br w), 1780 (w), 1717 (vs), 1589 (w), 1435 (m), 1389 (m), 1363 (w), 1287 (w), 1232 (w), 1194 (w), 1052 (s), 947 (w), 887 (w), 780 (m), 761 (m), 698 (w), 614 (w), 504 (w). **HR-MS:** (ESI) = *m/z* calcd. for: $C_{15}H_{20}N_3O_3$ [M+H]$^+$ 290.1505 u, found: 290.1492 u. **$[a]_\lambda^T$:** (c = 1.17 g/100 mL, $CHCl_3$) = $[a]_D^{20}$: –13.33°.

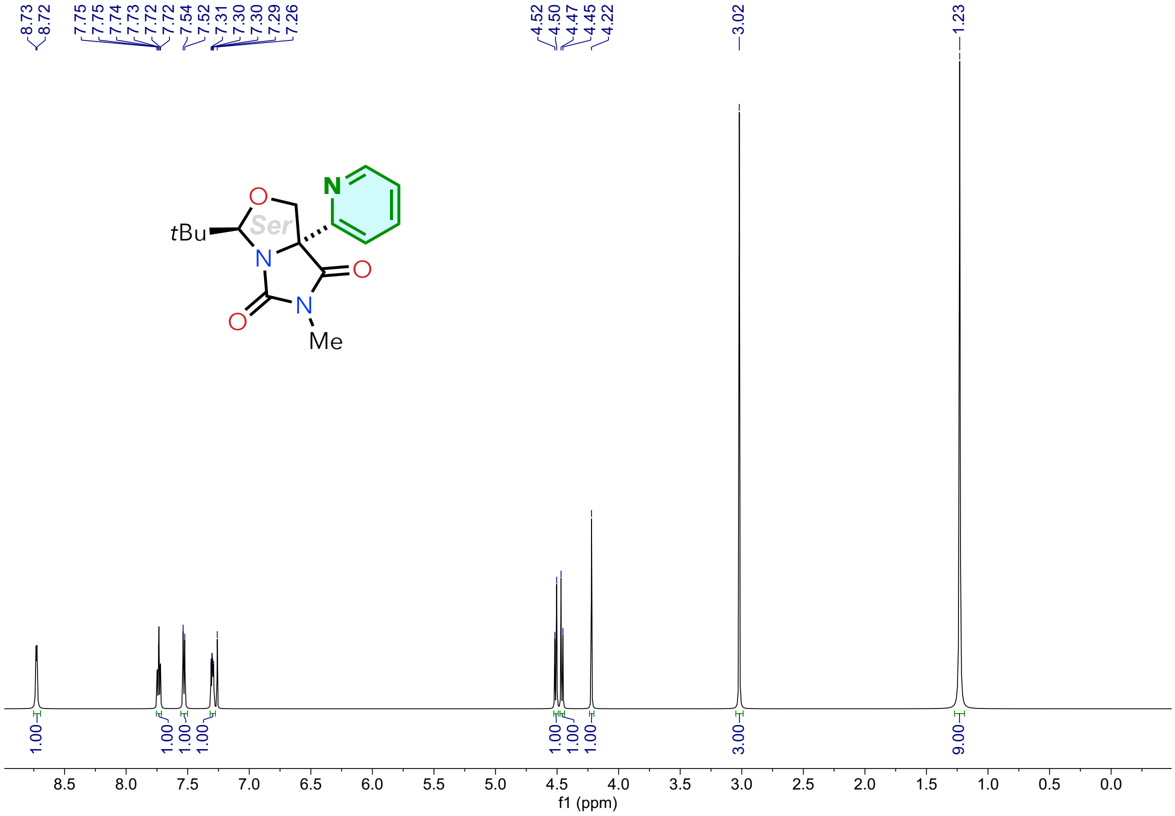
8.73
8.72
7.75
7.75
7.74
7.73
7.72
7.72
7.54
7.52
7.31
7.30
7.30
7.29
7.26
4.52
4.50
4.47
4.45
4.22
3.02
1.23
tBu
O
Ser
N
N
O
O
N
Me
1.00
1.00
1.00
1.00
1.00
1.00
1.00
3.00
9.00
f1 (ppm)

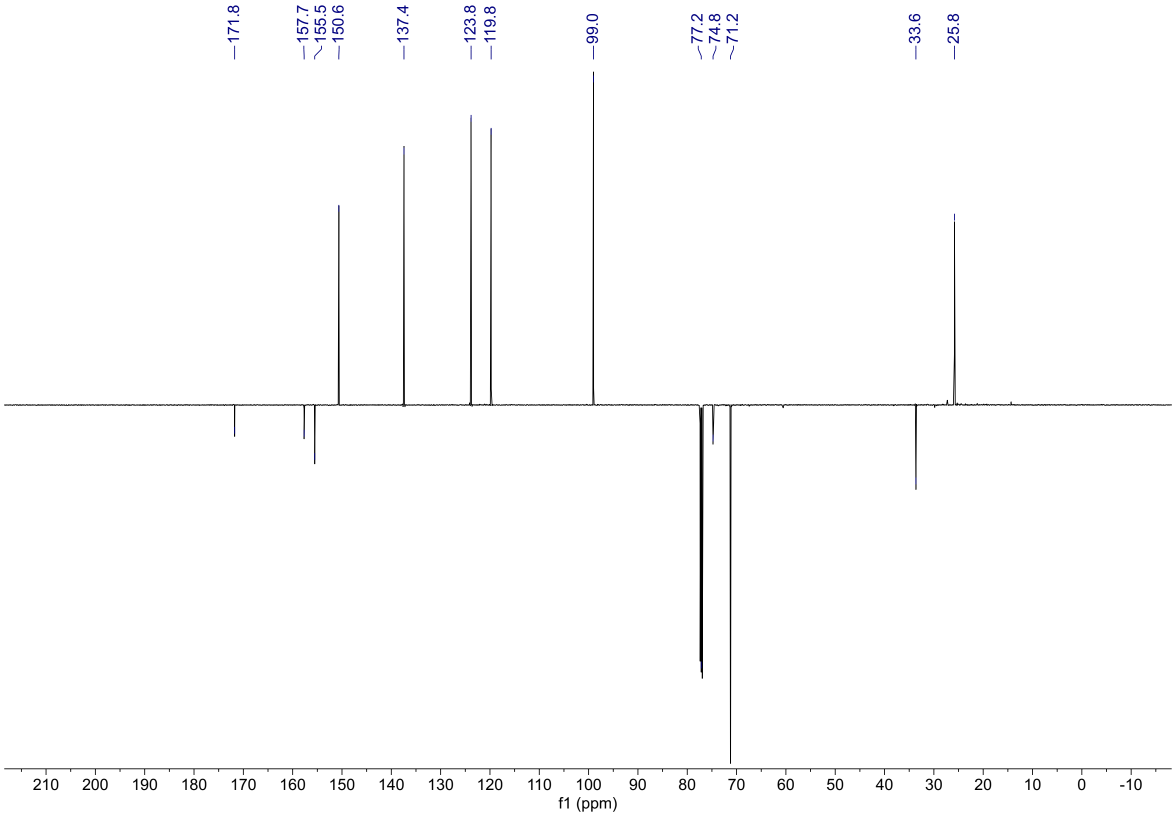
171.8
157.7
155.5
150.6
137.4
123.8
119.8
99.0
77.2
74.8
71.2
33.6
25.8
f1 (ppm)

(3*R*,7a*S*)-3-(*tert*-Butyl)-6-methyl-7a-(naphthalen-2-yl)dihydro-3*H*,5*H*-imidazo[1,5-*c*]oxazole-5,7(6*H*)-dione (5r)

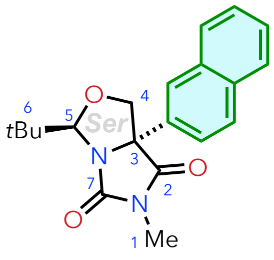

5r

Following **GP4**, to a solution of *N*-aryl urea **4r** (100 mg, 0.27 mmol, 1.0 equiv.) in dry THF (2.7 mL, 0.1 M) at –78 °C was added potassium bis(trimethylsilyl)amide solution (0.40 mL, 0.40 mmol, 1 M in THF, 1.5 equiv.) dropwise. The title compound **5r** was obtained as a beige solid (89 mg, 0.26 mmol, 97%) after purification by silica gel column chromatography (gradient elution, PE/EA).

Formula: $C_{20}H_{22}N_2O_3$, **MW:** 338.41 g/mol, **m.p.:** 171 – 174 °C. **TLC:** R_f = 0.73 (PE/EA 2:1), $KMnO_4$ stain. **^{1}H NMR** (600 MHz, $CDCl_3$): δ [ppm] = 8.26 (d, *J* 8.6 Hz, 1H, Ar), 7.88 (q, *J* 8.0 Hz, 3H, Ar), 7.63 (ddd, *J* 8.4, 6.9, 1.3 Hz, 1H, Ar), 7.54 (t, *J* 7.5 Hz, 1H, Ar), 7.46 (t, *J* 7.4 Hz, 1H, Ar), 4.91 (d, *J* 8.8 Hz, 1H, H-4a), 4.27 (d, *J* 8.8 Hz, 1H, H-4b), 4.22 (s, 1H, H-5), 3.00 (s, 3H, H-1, Me), 1.34 (s, 9H, *t*Bu). **^{13}C NMR** (150 MHz, $CDCl_3$): δ [ppm] = 172.7 (s, C-2, amide), 157.1 (s, C-7, urea), 135.1 (s, Ar), 133.9 (s, Ar), 130.2 (s, Ar), 130.0 (d, Ar), 128.9 (d, Ar), 126.5 (d, Ar), 126.3 (d, Ar), 126.1 (d, Ar), 125.1 (d, Ar), 122.3 (d, Ar), 97.4 (d, C-5), 74.7 (s, C-3), 72.8 (t, C-4), 33.2 (s, *t*Bu), 25.5 (q, C-1, Me). **FT-IR (ATR):** $\tilde{\nu}$ [cm^{-1}] = 2956 (br w), 1776 (m), 1715 (vs), 1509 (w), 1441 (m), 1380 (m), 1235 (w), 1283 (m), 1202 (m), 1142 (w), 1079 (m), 1056 (m), 1043 (m), 950 (w), 775 (s), 746 (m), 706 (m), 662 (w), 618 (w), 569 (w), 430 (m). **HR-MS:** (ESI) = *m*/*z* calcd. for: $C_{20}H_{23}N_2O_3$ $[M+H]^+$ 339.1709 u, found: 339.1707 u. **$[a]_\lambda^T$:** (c = 1.16 g/100 mL, $CHCl_3$) = $[a]_D^{20}$: –51.03°.

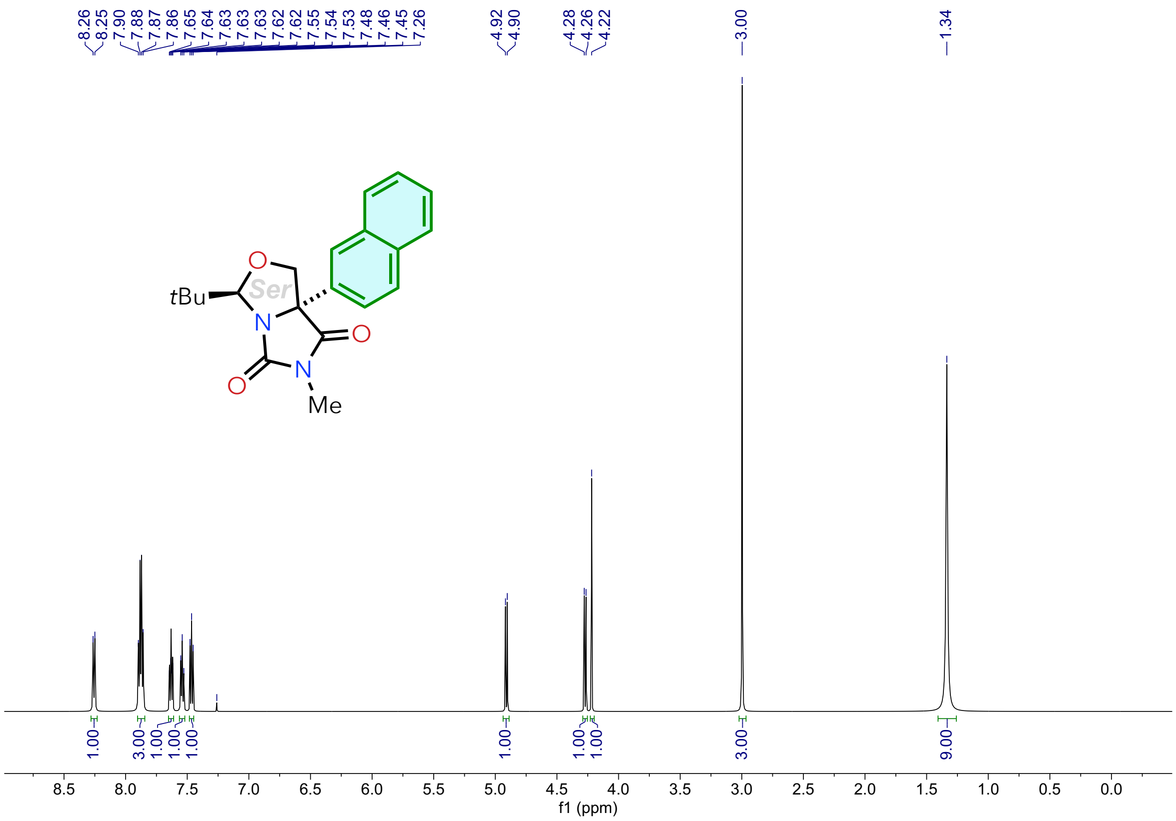
8.26
8.25
7.90
7.88
7.87
7.86
7.65
7.64
7.63
7.63
7.63
7.62
7.62
7.62
7.55
7.54
7.53
7.48
7.46
7.45
7.26
4.92
4.90
4.28
4.26
4.22
3.00
1.34
tBu
Ser
O
N
O
N
O
Me
1.00
3.00
1.00
1.00
1.00
1.00
1.00
1.00
3.00
9.00
8.5
8.0
7.5
7.0
6.5
6.0
5.5
5.0
4.5
4.0
3.5
3.0
2.5
2.0
1.5
1.0
0.5
0.0
f1 (ppm)

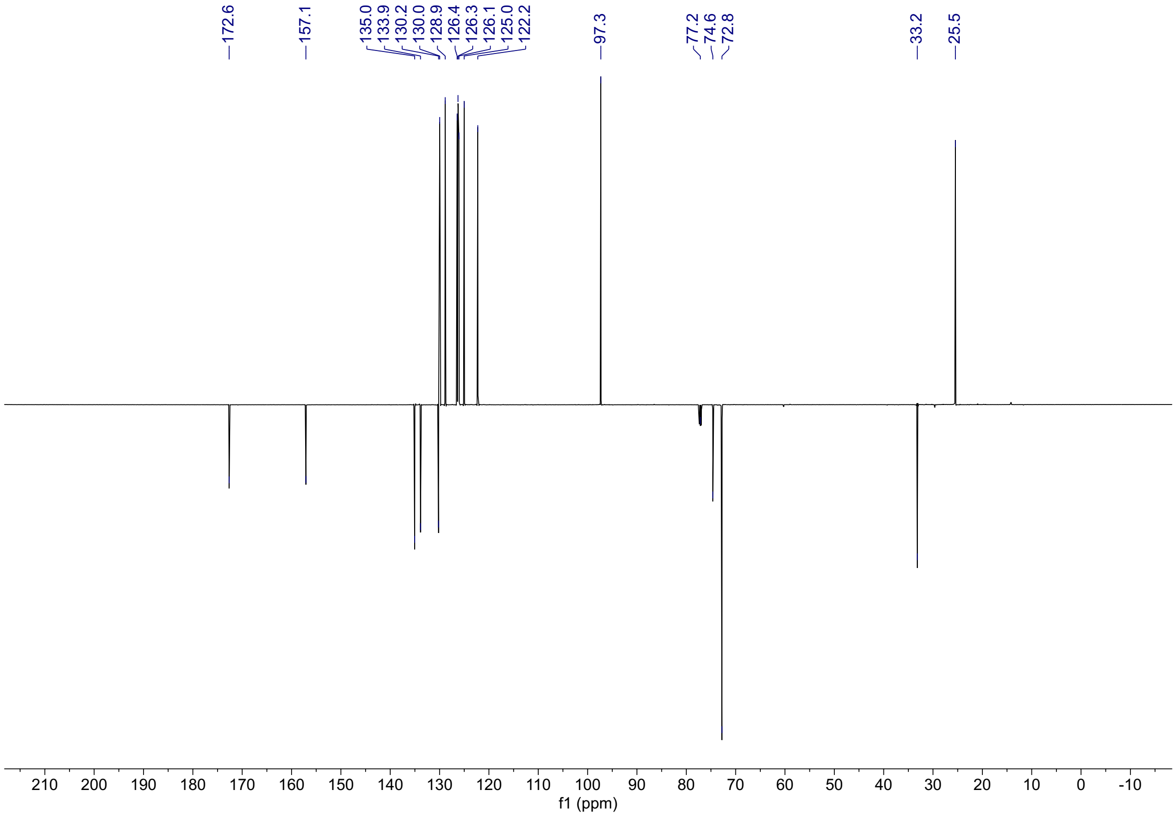
172.6
157.1
135.0
133.9
130.2
130.0
128.9
126.4
126.3
126.1
125.0
122.2
97.3
77.2
74.6
72.8
33.2
25.5
210
200
190
180
170
160
150
140
130
120
110
100
90
80
70
60
50
40
30
20
10
0
-10
f1 (ppm)

(3*R*,7a*R*)-3-(*tert*-butyl)-6-methyl-7a-phenyldihydro-3*H*,5*H*-imidazo[1,5-*c*]thiazole-5,7(6*H*)-dione (23a)

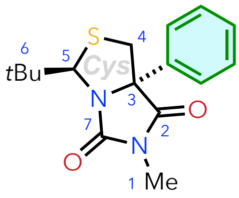

23a

Following **GP4**, to a solution of *N*-aryl urea **19a** (100 mg, 0.30 mmol, 1.0 equiv.) in dry THF (3.0 mL, 0.1 M) at –78 °C was added potassium bis(trimethylsilyl)amide solution (0.45 mL, 0.45 mmol, 1 M in THF, 1.5 equiv.) dropwise. The title compound **23a** was obtained as a colourless oil (68 mg, 0.22 mmol, 75%) after purification by silica gel column chromatography (gradient elution, PE/EA).

Formula: $C_{16}H_{20}N_2O_2S$, **MW:** 304.41 g/mol, **m.p.:** 125 – 128 °C. **TLC:** R_f = 0.64 (PE/EA 2:1), $KMnO_4$ stain. **^{1}H NMR** (600 MHz, $CDCl_3$): δ [ppm] = 7.52 (d, *J* 7.8 Hz, 2H, Ar), 7.44 (t, *J* 7.6 Hz, 2H, Ar), 7.39 (t, *J* 7.2 Hz, 1H, Ar), 4.50 (s, 1H, H-5), 3.58 (d, *J* 11.5 Hz, 1H, H-4a), 3.53 (d, *J* 11.5 Hz, 1H, H-4b), 2.99 (s, 3H, H-1, Me), 1.35 (s, 9H, *t*Bu). **^{13}C NMR** (150 MHz, $CDCl_3$): δ [ppm] = 172.4 (s, C-2), 154.9 (s, C-7), 136.8 (s, Ar), 139.4 (d, 2Ar), 129.1 (d, Ar), 125.5 (d, 2Ar), 77.6 (d, C-5), 76.9 (s, C-3), 38.6 (t, C-4), 34.4 (s, *t*Bu), 28.5 (q, C-6, *t*Bu), 25.6 (q, C-1, Me). **FT-IR (ATR):** $\tilde{\nu}$ [cm^{-1}] = 2969 (br w), 1776 (w), 1712 (vs), 1444 (s), 1388 (m), 1293 (w), 1206 (w), 1181 (w), 1158 (w), 1043 (w), 997 (w), 886 (w), 762 (w), 726 (m), 695 (m), 639 (w), 506 (m), 419 (w). **HR-MS:** (ESI) = *m*/*z* calcd. for: $C_{16}H_{21}N_2O_2S$ $[M+H]^+$ 305.1324 u, found: 305.1320 u. **$[a]_\lambda^T$:** (c = 1.11 g/100 mL, MeOH) = $[a]_D^{20}$: –12.97°.

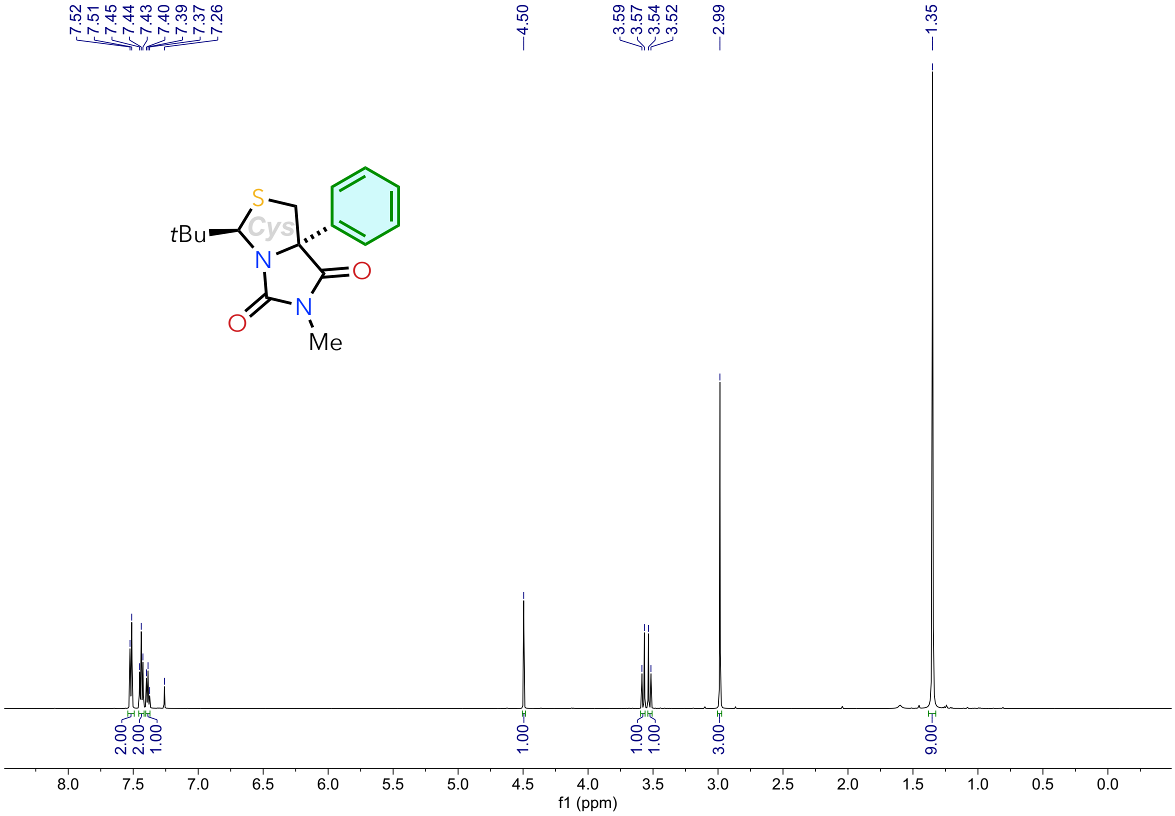
7.52
7.51
7.45
7.44
7.43
7.40
7.39
7.37
7.26
4.50
3.59
3.57
3.54
3.52
2.99
1.35
S
Cys
tBu
N
O
N
O
Me
2.00
2.00
1.00
1.00
1.00
1.00
3.00
9.00
8.0 7.5 7.0 6.5 6.0 5.5 5.0 4.5 4.0 3.5 3.0 2.5 2.0 1.5 1.0 0.5 0.0
f1 (ppm)

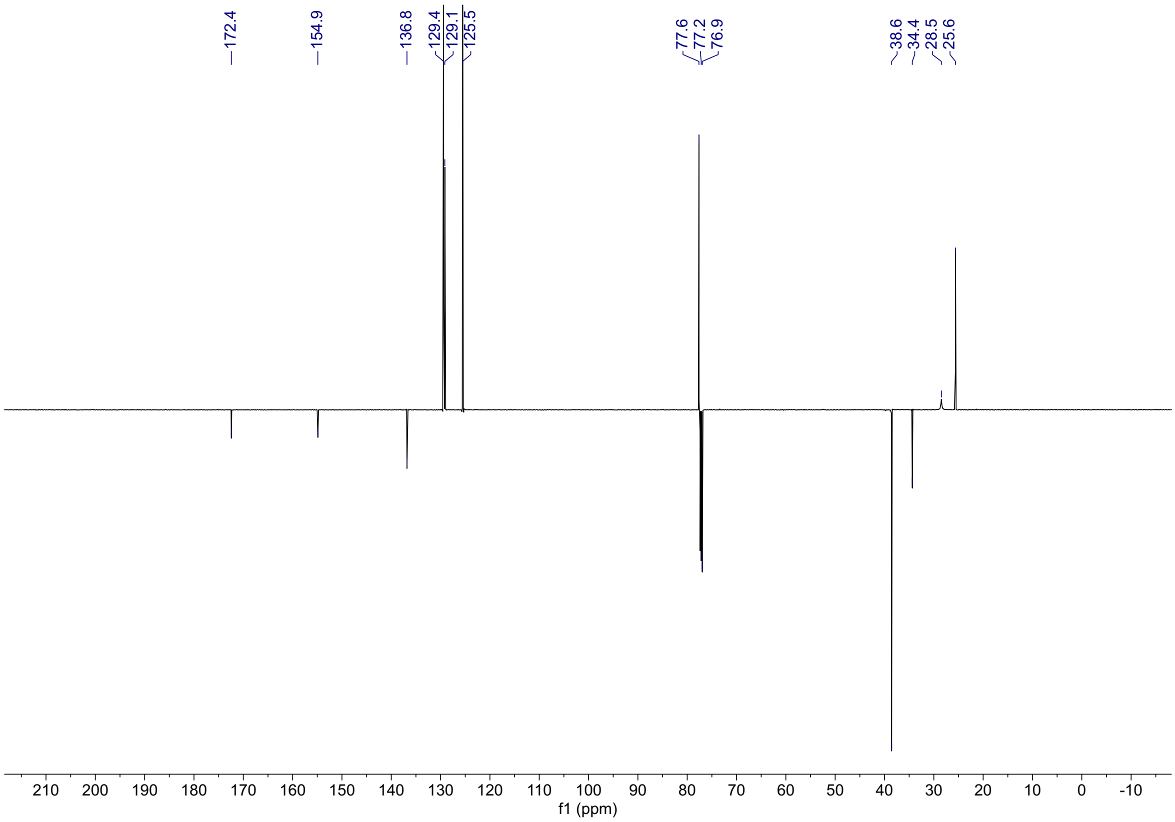
172.4
154.9
136.8
129.4
129.1
125.5
77.6
77.2
76.9
38.6
34.4
28.5
25.6
210 200 190 180 170 160 150 140 130 120 110 100 90 80 70 60 50 40 30 20 10 0 -10
f1 (ppm)

(3*R*,7a*R*)-7a-(4-Bromophenyl)-3-(*tert*-butyl)-6-methyldihydro-3*H*,5*H*-imidazo[1,5-*c*]thiazole-5,7(6*H*)-dione (23b)

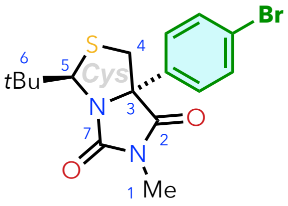

23b

Following **GP4**, to a solution of *N*-aryl urea **19b** (100 mg, 0.24 mmol, 1.0 equiv.) in dry THF (2.4 mL, 0.1 M) at –78 °C was added potassium bis(trimethylsilyl)amide solution (0.36 mL, 0.36 mmol, 1 M in THF, 1.5 equiv.) dropwise. The title compound **23b** was obtained as a beige solid (80 mg, 0.21 mmol, 87%) after purification by silica gel column chromatography (gradient elution, PE/EA).

Formula: $C_{16}H_{19}BrN_2O_2S$, **MW:** 383.30 g/mol, **m.p.:** 51 – 54 °C. **TLC:** R_f = 0.74 (PE/EA 2:1), $KMnO_4$ stain. ^{1}**H NMR** (600 MHz, $CDCl_3$): δ [ppm] = 7.56 (d, *J* 8.7 Hz, 2H, Ar), 7.39 (d, *J* 8.7 Hz, 2H, Ar), 4.44 (s, 1H, H-5), 3.56 (d, *J* 11.6 Hz, 1H, H-4a), 3.45 (d, *J* 11.6 Hz, 1H, H-4b), 2.98 (s, 3H, H-1, Me), 1.34 (s, 9H, *t*Bu). ^{13}**C NMR** (150 MHz, $CDCl_3$): δ [ppm] = 172.0 (s, C-2), 154.8 (s, C-7), 135.9 (s, Ar), 132.5 (d, 2Ar), 127.3 (d, 2Ar), 123.5 (s, Ar), 77.9 (d, C-5), 76.6 (s, C-3), 38.6 (t, C-4), 34.4 (s, *t*Bu), 28.5 (q, C-6, *t*Bu), 25.7 (q, C-1, Me). **FT-IR (ATR):** $\tilde{\nu}$ [cm^{-1}] = 2957 (br w), 1778 (w), 1713 (vs), 1486 (w), 1438 (m), 1388 (m), 1292 (w), 1074 (w), 1044 (w), 1009 (m), 829 (w), 769 (w), 714 (w), 652 (w), 582 (w), 506 (m). **HR-MS:** (ESI) = *m*/*z* calcd. for: $C_{16}H_{20}{}^{79}BrN_2O_2S$ $[M+H]^+$ 383.0429 u, found: 383.0432 u. $\mathbf{[a]_{\lambda}^{T}}$**:** (c = 1.00 g/100 mL, $CHCl_3$) = $[a]_D^{20}$: –9.20°.

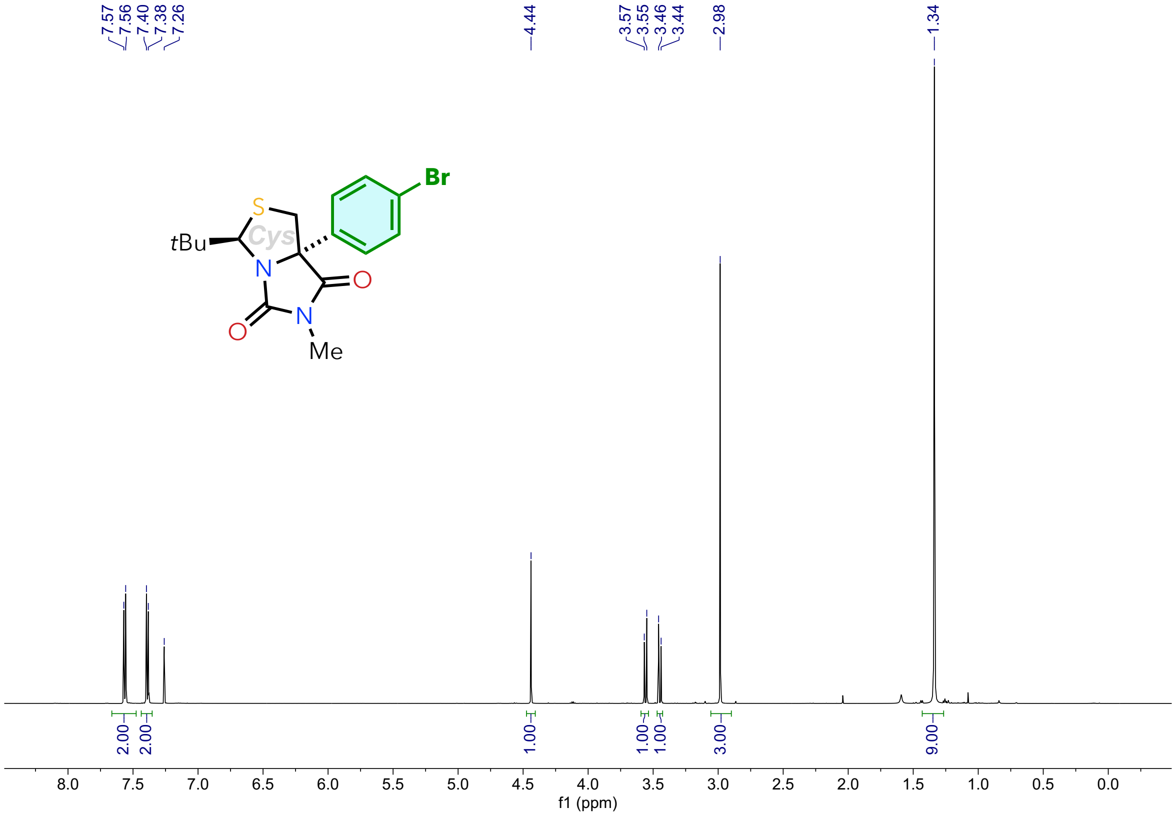
7.57
7.56
7.40
7.38
7.26
4.44
3.57
3.55
3.46
3.44
2.98
1.34
Br
S
Cys
tBu
N
O
N
O
Me
2.00
2.00
1.00
1.00
1.00
3.00
9.00
8.0 7.5 7.0 6.5 6.0 5.5 5.0 4.5 4.0 3.5 3.0 2.5 2.0 1.5 1.0 0.5 0.0
f1 (ppm)

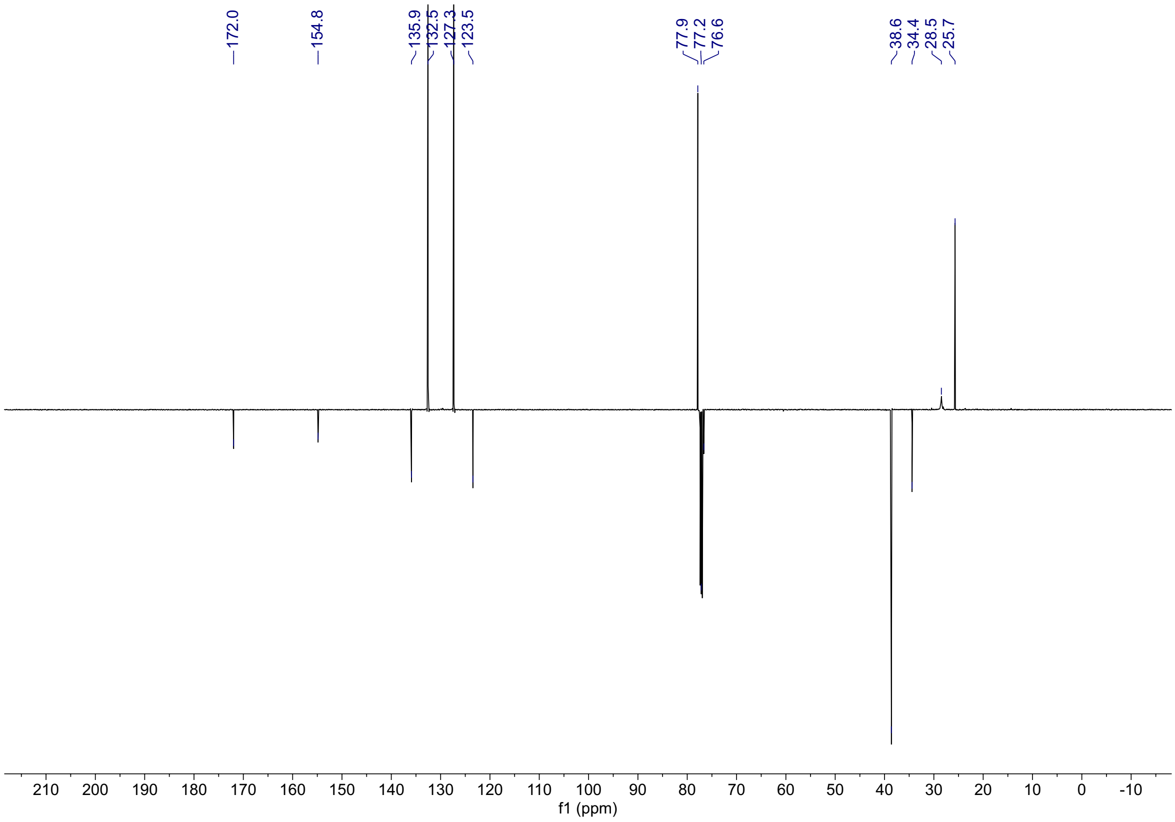
172.0
154.8
135.9
132.5
127.3
123.5
77.9
77.2
76.6
38.6
34.4
28.5
25.7
210 200 190 180 170 160 150 140 130 120 110 100 90 80 70 60 50 40 30 20 10 0 -10
f1 (ppm)

(3*R*,7a*R*)-7a-(3-bromophenyl)-3-(*tert*-butyl)-6-methyldihydro-3*H*,5*H*-imidazo[1,5-*c*]thiazole-5,7(6*H*)-dione (23c)

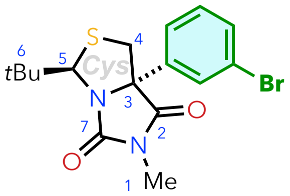

23c

Following **GP4**, to a solution of *N*-aryl urea **19c** (100 mg, 0.24 mmol, 1.0 equiv.) in dry THF (2.4 mL, 0.1 M) at –78 °C was added potassium bis(trimethylsilyl)amide solution (0.36 mL, 0.36 mol, 1 M in THF, 1.5 equiv.) dropwise. The title compound **23c** was obtained as a beige solid (72 mg, 0.19 mmol, 78%) after purification by silica gel column chromatography (gradient elution, PE/EA).

Formula: $C_{16}H_{19}BrN_2O_2S$, **MW:** 383.30 g/mol, **m.p.:** 52 – 55 °C. **TLC:** R_f = 0.51 (PE/EA 2:1), $KMnO_4$ stain. **^{1}H NMR** (600 MHz, $CDCl_3$): δ [ppm] = 7.65 (t, *J* 1.9 Hz, 1H, Ar), 7.52 (d, *J* 7.9 Hz, 1H, Ar), 7.46 (d, *J* 7.9 Hz, 1H, Ar), 7.31 (t, *J* 7.9 Hz, 1H, Ar), 4.45 (s, 1H, H-5), 3.57 (d, *J* 11.6 Hz, 1H, H-4a), 3.46 (d, *J* 11.6 Hz, 1H, H-4b), 2.99 (s, 3H, H-1, Me), 1.35 (s, 9H, *t*Bu). **^{13}C NMR** (150 MHz, $CDCl_3$): δ [ppm] = 171.9 (s, C-2), 154.9 (s, C-7), 139.2 (s, Ar), 132.3 (d, Ar), 130.9 (d, Ar), 128.7 (d, Ar), 124.4 (d, Ar), 123.5 (s, Ar), 78.1 (d, C-5), 76.5 (s, C-3), 38.8 (t, C-4), 34.4 (s, *t*Bu), 28.5 (q, C-6, *t*Bu), 25.7 (q, C-1, Me). **FT-IR (ATR):** $\tilde{\nu}$ [cm^{-1}] = 2958 (br w), 1780 (w), 1713 (vs), 1567 (w), 1439 (m), 1387 (m), 1291 (w), 1205 (w), 1182 (w), 1045 (w), 995 (w), 889 (w), 789 (w), 683 (m), 660 (m), 437 (w). **HR-MS:** (ESI) = *m*/*z* calcd. for: $C_{15}H_{16}{}^{79}BrN_2O_2S$ [M–Me]$^+$ 367.0116 u, found: 367.0101 u. **$[a]_\lambda^T$:** (c = 1.10 g/100 mL, $CHCl_3$) = $[a]_D^{20}$: –13.09°.

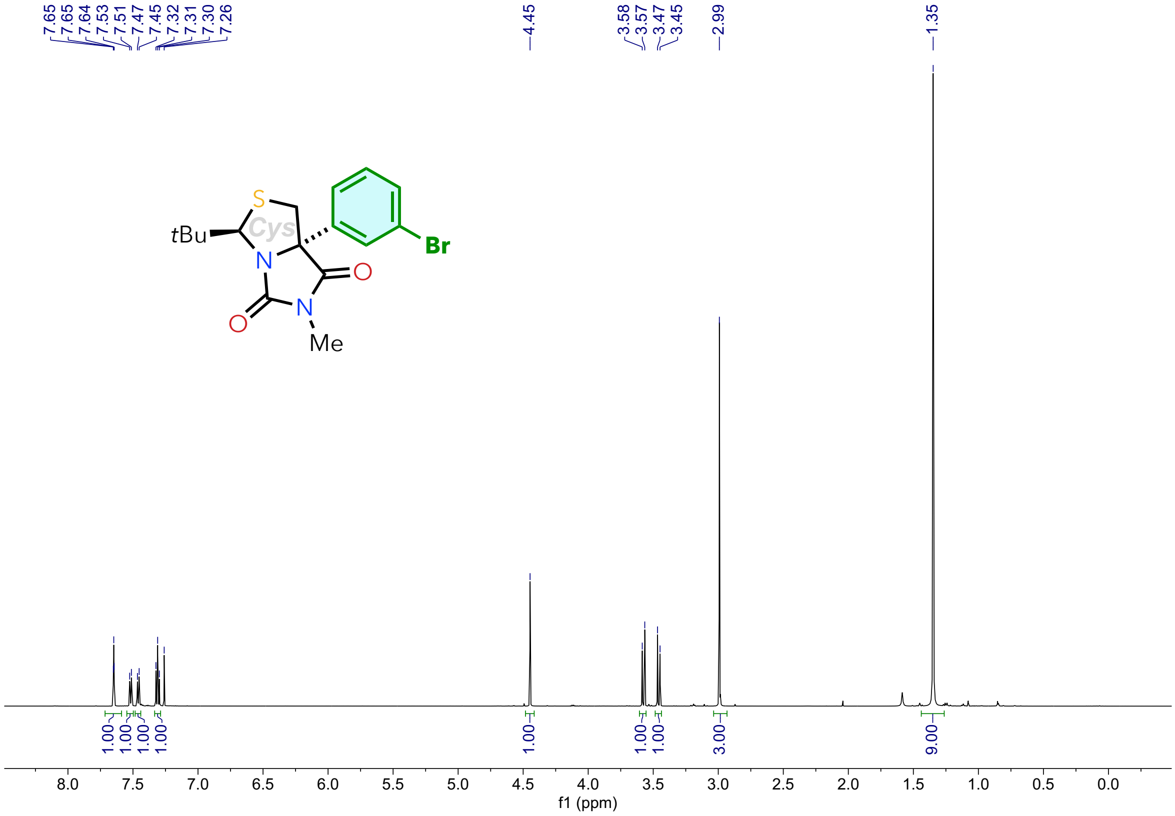
7.65
7.65
7.64
7.53
7.51
7.47
7.45
7.32
7.31
7.30
7.26
4.45
3.58
3.57
3.47
3.45
2.99
1.35
S
Cys
tBu
N
O
N
O
Me
Br
1.00
1.00
1.00
1.00
1.00
1.00
1.00
3.00
9.00
8.0 7.5 7.0 6.5 6.0 5.5 5.0 4.5 4.0 3.5 3.0 2.5 2.0 1.5 1.0 0.5 0.0
f1 (ppm)

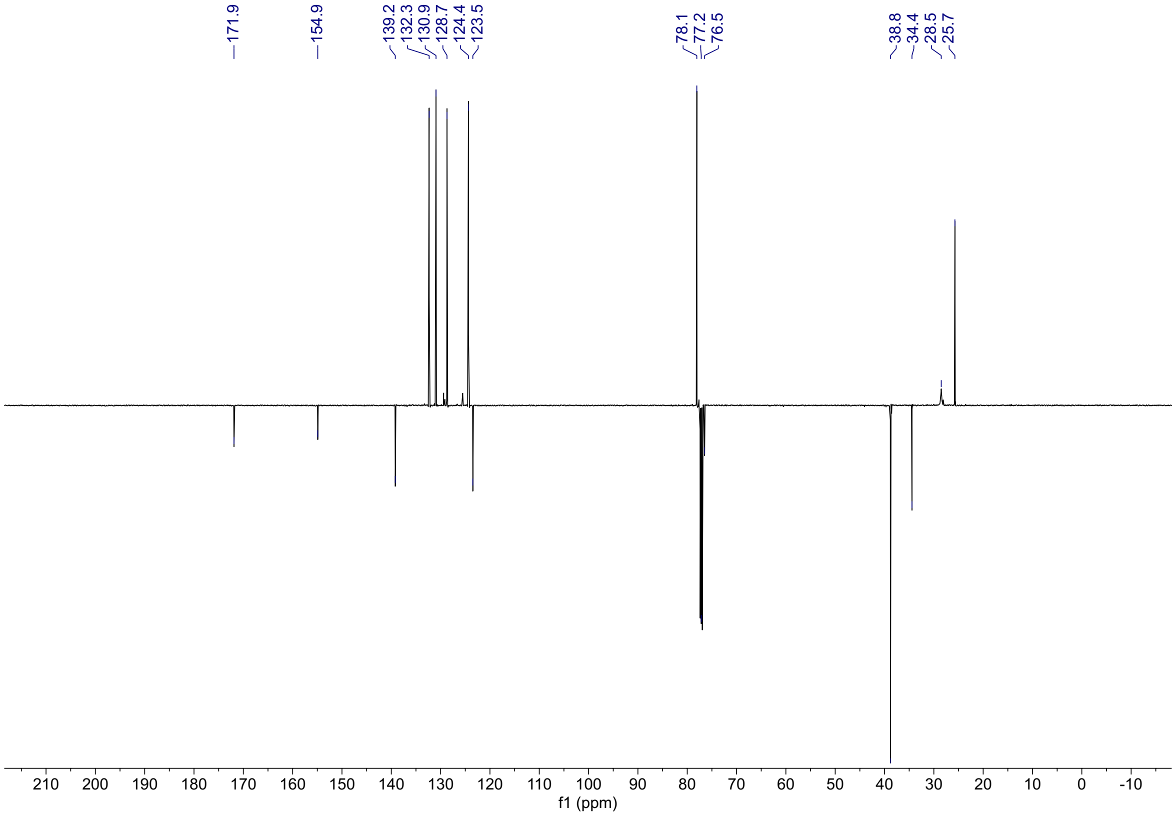
171.9
154.9
139.2
132.3
130.9
128.7
124.4
123.5
78.1
77.2
76.5
38.8
34.4
28.5
25.7
210 200 190 180 170 160 150 140 130 120 110 100 90 80 70 60 50 40 30 20 10 0 -10
f1 (ppm)

(3*R*,7a*R*)-7a-(2-Bromophenyl)-3-(*tert*-butyl)-6-methyldihydro-3*H*,5*H*-imidazo[1,5-*c*]thiazole-5,7(6*H*)-dione (23d)

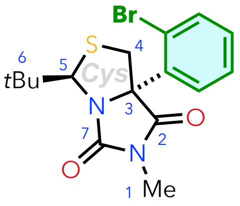

23d

Following **GP4**, to a solution of *N*-aryl urea **19d** (100 mg, 0.24 mmol, 1.0 equiv.) in dry THF (2.4 mL, 0.1 M) at –78 °C was added potassium bis(trimethylsilyl)amide solution (0.36 mL, 0.36 mmol, 1 M in THF, 1.5 equiv.) dropwise. The title compound **23d** was obtained as a white crystalline solid (48 mg, 0.13 mmol, 52%) after purification by silica gel column chromatography (gradient elution, PE/EA).

Formula: $C_{16}H_{19}BrN_2O_2S$, **MW:** 383.30 g/mol, **m.p.:** 183 – 186 °C. **TLC:** R_f = 0.60 (PE/EA 2:1), $KMnO_4$ stain. **^{1}H NMR** (600 MHz, $CDCl_3$): δ [ppm] = 7.70 (dd, *J* 8.0, 1.6 Hz, 1H, Ar), 7.68 (dd, *J* 7.9, 1.4 Hz, 1H, Ar), 7.42 (td, *J* 7.7, 1.4 Hz, 1H, Ar), 7.28 (td, *J* 7.7, 1.6 Hz, 1H, Ar), 4.14 (s, 1H, H-5), 3.82 (d, *J* 12.1 Hz, 1H, H-4a), 3.25 (d, *J* 12.1 Hz, 1H, H-4b), 3.05 (s, 3H, H-1, Me), 1.26 (s, 9H, *t*Bu). **^{13}C NMR** (150 MHz, $CDCl_3$): δ [ppm] = 170.9 (s, C-2), 154.1 (s, C-7), 135.9 (d, Ar), 132.2 (s, Ar), 131.2 (d, Ar), 130.3 (d, Ar), 127.9 (d, Ar), 122.7 (s, Ar), 77.1 (s, C-3), 76.0 (d, C-5), 35.9 (t, C-4), 34.3 (s, *t*Bu), 28.3 (q, C-6, *t*Bu), 25.5 (q, C-1, Me). **FT-IR (ATR):** $\tilde{\nu}$ [cm^{-1}] = 2964 (br w), 1777 (w), 1719 (vs), 1438 (m), 1388 (w), 1311 (w), 1288 (m), 1258 (w), 1225 (m), 1208 (w), 1122 (w), 1033 (w), 1025 (w), 994 (w), 750 (m), 640 (w), 575 (w), 455 (w). **HR-MS:** (ESI) = *m*/*z* calcd. for: $C_{16}H_{20}{}^{79}BrN_2O_2S$ $[M+H]^+$ 383.0429 u, found: 383.0418 u. **$[a]_\lambda^T$:** (c = 1.00 g/100 mL, $CHCl_3$) = $[a]_D^{20}$: –15.20°.

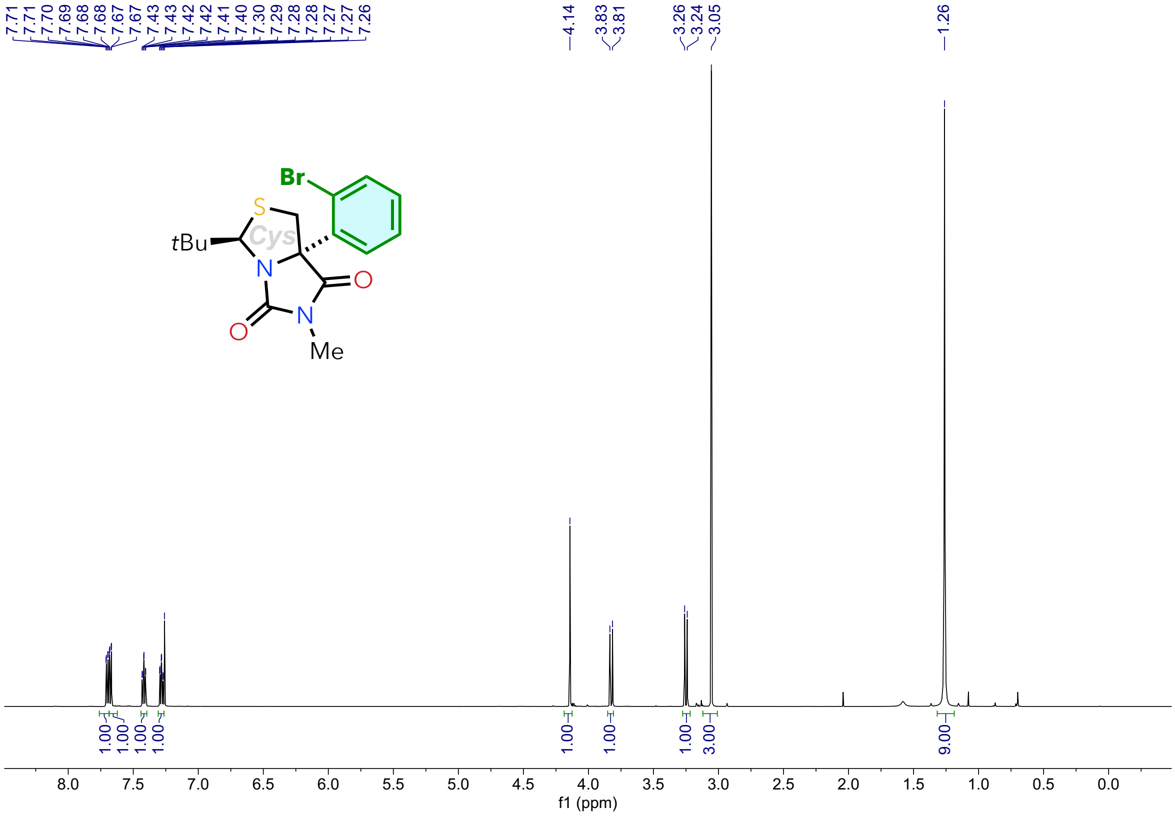
7.71 7.71 7.70 7.69 7.68 7.68 7.67 7.67 7.43 7.43 7.42 7.42 7.41 7.40 7.30 7.29 7.28 7.28 7.27 7.27 7.26
4.14
3.83 3.81
3.26 3.24 3.05
1.26
Br
S
Cys
tBu
N
O
N
O
Me
1.00 1.00 1.00 1.00
1.00
1.00
1.00 3.00
9.00
8.0 7.5 7.0 6.5 6.0 5.5 5.0 4.5 4.0 3.5 3.0 2.5 2.0 1.5 1.0 0.5 0.0
f1 (ppm)

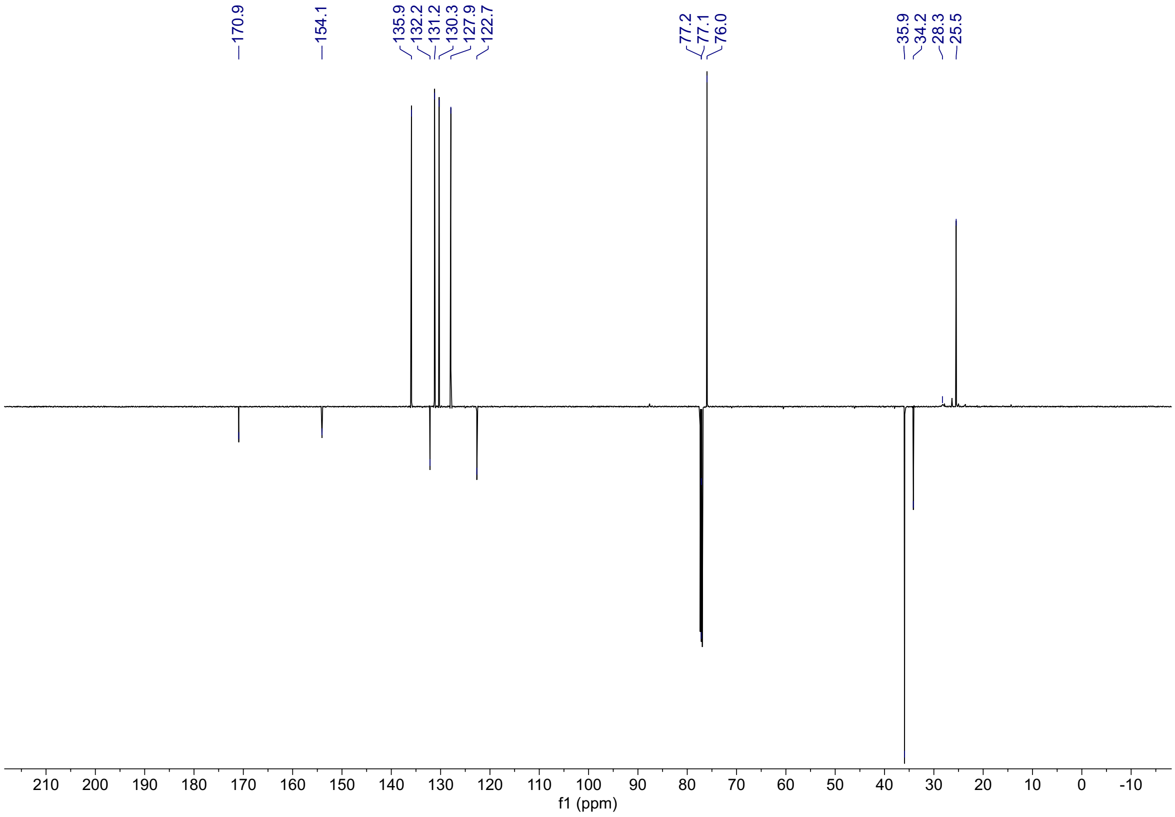
170.9
154.1
135.9 132.2 131.2 130.3 127.9 122.7
77.2 77.1 76.0
35.9 34.2 28.3 25.5
210 200 190 180 170 160 150 140 130 120 110 100 90 80 70 60 50 40 30 20 10 0 -10
f1 (ppm)

(3*R*,7a*R*)-3-(*tert*-Butyl)-6-methyl-7a-(4-(trifluoromethyl)phenyl)dihydro-3*H*,5*H*-imidazo[1,5-*c*]thiazole-5,7(6*H*)-dione (23e)

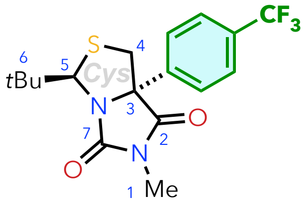

23e

Following **GP4**, to a solution of *N*-aryl urea **19e** (100 mg, 0.25 mmol, 1.0 equiv.) in dry THF (2.5 mL, 0.1 M) at –78 °C was added potassium bis(trimethylsilyl)amide solution (0.37 mL, 0.37 mmol, 1 M in THF, 1.5 equiv.) dropwise. The title compound **23e** was obtained as a colourless oil (62 mg, 0.17 mmol, 68%) after purification by silica gel column chromatography (gradient elution, PE/EA).

Formula: $C_{17}H_{19}F_3N_2O_2S$, **MW:** 372.41 g/mol. **TLC:** R_f = 0.71 (PE/EA 2:1), $KMnO_4$ stain. **^{1}H NMR** (500 MHz, $CDCl_3$): δ [ppm] = 7.71 (d, *J* 8.4 Hz, 2H, Ar), 7.66 (d, *J* 8.4 Hz, 2H, Ar), 4.45 (s, 1H, H-5), 3.64 (d, *J* 11.6 Hz, 1H, H-4a), 3.48 (d, *J* 11.6 Hz, 1H, H-4b), 3.00 (s, 3H, H-1, Me), 1.36 (s, 9H, *t*Bu). **^{13}C NMR** (125 MHz, $CDCl_3$): δ [ppm] = 171.8 (s, C-2, amide), 155.0 (s, C-7, urea), 131.5 (s, *q J* 32.8 Hz, Ar), 126.4 (d, *q J* 3.7 Hz, 2Ar), 126.2 (d, 2Ar), 123.9 (s, *q J* 271.2 Hz, Ar), 78.2 (d, C-5), 76.8 (s, C-3), 39.0 (t, C-4), 34.5 (s, *t*Bu), 28.5 (q, C-6, *t*Bu), 25.8 (q, C-1, Me). **^{19}F NMR** (471 MHz, $CDCl_3$, C_6F_6 ref.): δ [ppm] = –66.00 (s, 3F). **FT-IR (ATR):** $\tilde{\nu}$ [cm^{-1}] = 2962 (br w), 1783 (w), 1720 (vs), 1446 (m), 1410 (w), 1391 (w), 1367 (w), 1326 (s), 1167 (w), 1127 (m), 1069 (m), 1045 (w), 1018 (w), 848 (w), 734 (w), 511 (w). **HR-MS:** (ESI) = *m*/*z* calcd. for: $C_{16}H_{16}F_3N_2O_2S$ $[M–Me]^+$ 357.0885 u, found: 357.0874 u. **$[a]_\lambda^T$:** (c = 1.00 g/100 mL, $CHCl_3$) = $[a]_D^{20}$: –9.20°.

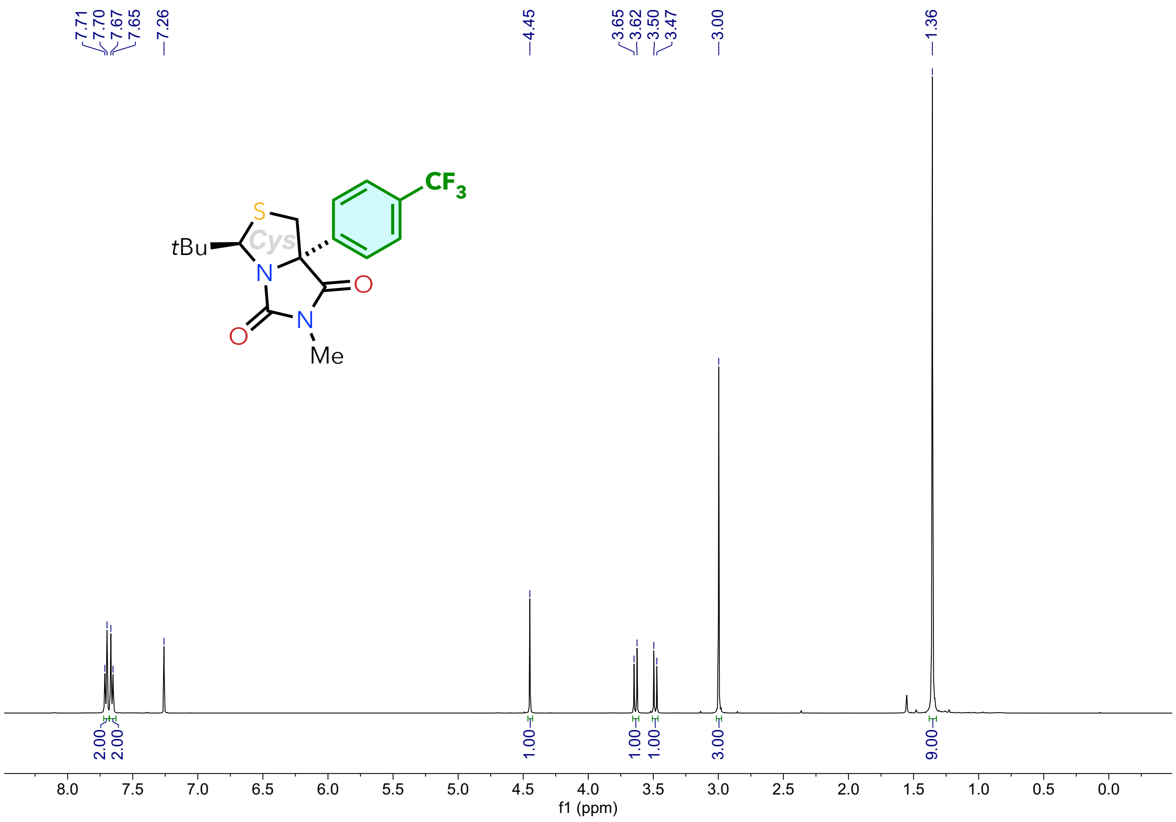

7.71
7.70
7.67
7.65
7.26
4.45
3.65
3.62
3.50
3.47
3.00
1.36
CF3
S
Cys
tBu
N
O
N
O
Me
2.00
2.00
1.00
1.00
1.00
3.00
9.00
8.0
7.5
7.0
6.5
6.0
5.5
5.0
4.5
4.0
3.5
3.0
2.5
2.0
1.5
1.0
0.5
0.0
f1 (ppm)

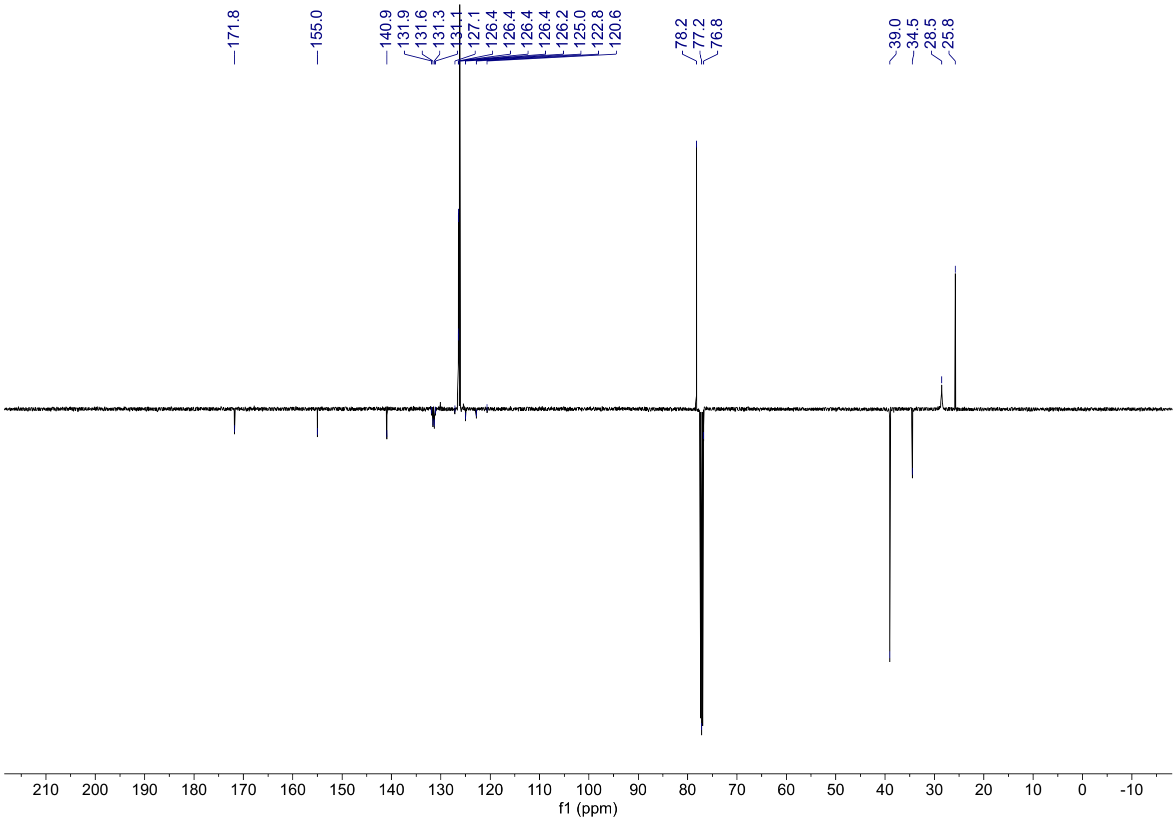

171.8
155.0
140.9
131.9
131.6
131.3
131.1
127.1
126.4
126.4
126.4
126.4
126.2
125.0
122.8
120.6
78.2
77.2
76.8
39.0
34.5
28.5
25.8
210
200
190
180
170
160
150
140
130
120
110
100
90
80
70
60
50
40
30
20
10
0
-10
f1 (ppm)

4-((3*R*,7a*R*)-3-(*tert*-Butyl)-6-methyl-5,7-dioxodihydro-1*H*,3*H*-imidazo[1,5-*c*]thiazol-7a(5*H*)-yl)benzonitrile (23f)

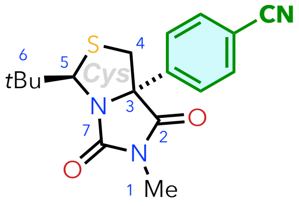

23f

Following **GP4**, to a solution of *N*-aryl urea **19f** (100 mg, 0.26 mmol, 1.0 equiv.) in dry THF (2.6 mL, 0.1 M) at –78 °C was added potassium bis(trimethylsilyl)amide solution (0.38 mL, 0.38 mmol, 1 M in THF, 1.5 equiv.) dropwise. The title compound **23f** was obtained as a yellowish oil (33 mg, 0.10 mmol, 40%) after purification by silica gel column chromatography (gradient elution, PE/EA).

Formula: $C_{17}H_{19}N_3O_2S$, **MW:** 329.42 g/mol. **TLC:** R_f = 0.56 (PE/EA 2:1), $KMnO_4$ stain. **^{1}H NMR** (500 MHz, $CDCl_3$): δ [ppm] = 7.74 (d, *J* 8.6 Hz, 2H, Ar), 7.66 (d, *J* 8.6 Hz, 2H, Ar), 4.41 (s, 1H, H-5), 3.64 (d, *J* 11.7 Hz, 1H, H-4a), 3.44 (d, *J* 11.7 Hz, 1H, H-4b), 2.99 (s, 3H, H-1, Me), 1.35 (s, 9H, *t*Bu). **^{13}C NMR** (125 MHz, $CDCl_3$): δ [ppm] = 171.4 (s, C-2, amide), 155.0 (s, C-7, urea), 142.2 (s, Ar), 133.1 (d, 2Ar), 126.5 (d, 2Ar), 118.2 (s, Ar), 113.3 (s, Ar), 78.5 (d, C-5), 76.7 (s, C-3), 39.2 (t, C-4), 34.5 (s, *t*Bu), 28.5 (q, C-6, *t*Bu), 25.8 (q, C-1, Me). **FT-IR (ATR):** $\tilde{\nu}$ [cm^{-1}] = 2980 (br w), 2230 (w), 1781 (w), 1717 (vs), 1444 (m), 1390 (m), 1270 (w), 1045 (w), 1000 (w), 913 (w), 846 (w), 755 (m), 733 (m), 556 (m). **HR-MS:** (ESI) = *m*/*z* calcd. for: $C_{16}H_{16}N_3O_2S$ [M–Me]$^+$ 314.0963 u, found: 314.0956 u. **$[a]_\lambda^T$:** (c = 1.00 g/100 mL, $CHCl_3$) = $[a]_D^{20}$: –9.20°.

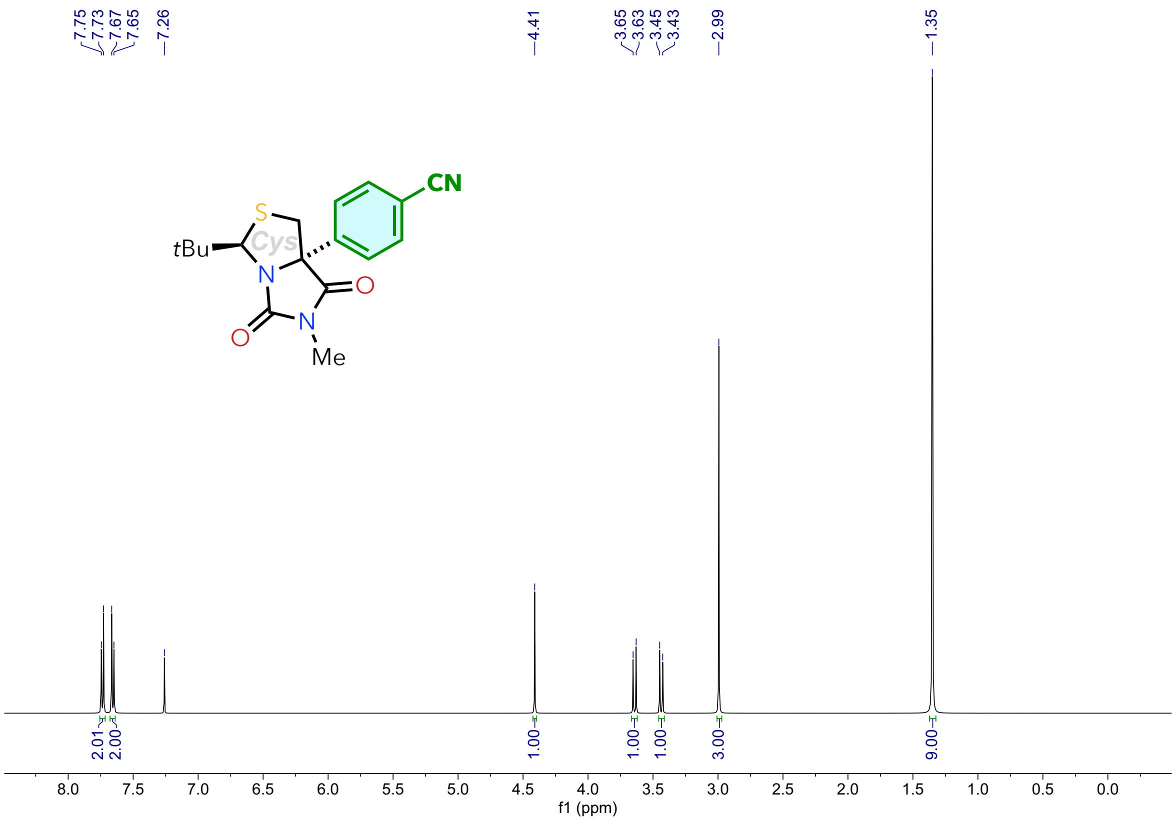
7.75
7.73
7.67
7.65
7.26
4.41
3.65
3.63
3.45
3.43
2.99
1.35
CN
S
tBu
Cys
N
O
O
N
Me
2.01
2.00
1.00
1.00
1.00
3.00
9.00
8.0 7.5 7.0 6.5 6.0 5.5 5.0 4.5 4.0 3.5 3.0 2.5 2.0 1.5 1.0 0.5 0.0
f1 (ppm)

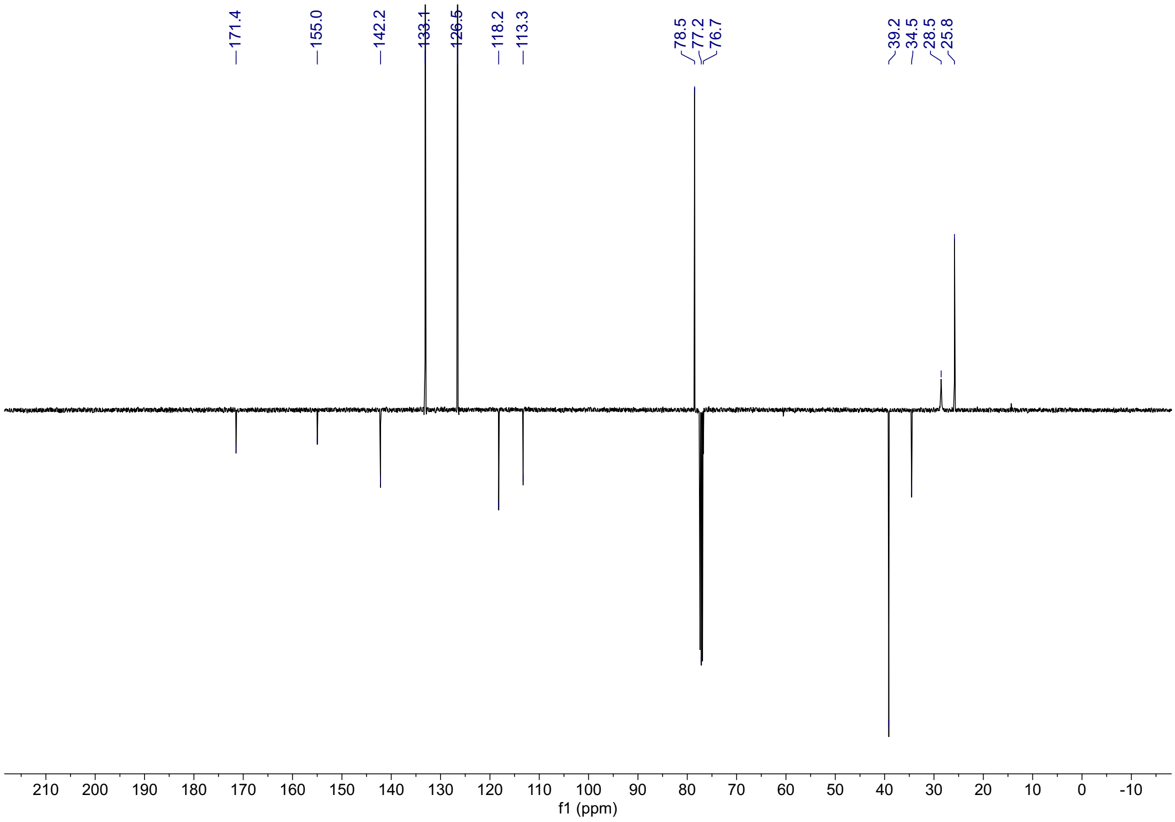
171.4
155.0
142.2
133.1
126.5
118.2
113.3
78.5
77.2
76.7
39.2
34.5
28.5
25.8
210 200 190 180 170 160 150 140 130 120 110 100 90 80 70 60 50 40 30 20 10 0 -10
f1 (ppm)

3-((3R,7aR)-3-(*tert*-Butyl)-6-methyl-5,7-dioxodihydro-1H,3H-imidazo[1,5-*c*]thiazol-7a(5H)-yl)benzonitrile (23g)

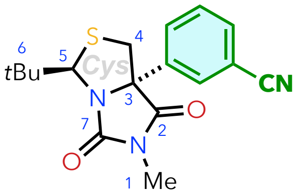

23g

Following **GP4**, to a solution of *N*-aryl urea **19g** (100 mg, 0.28 mmol, 1.0 equiv.) in dry THF (2.8 mL, 0.1 M) at –78 °C was added potassium bis(trimethylsilyl)amide solution (0.41 mL, 0.41 mmol, 1 M in THF, 1.5 equiv.) dropwise. The title compound **23g** was obtained as a white solid (80 mg, 0.24 mmol, 88%) after purification by silica gel column chromatography (gradient elution, PE/EA).

Formula: $C_{17}H_{19}N_3O_2S$, **MW:** 329.42 g/mol, **m.p.:** 65 – 67 °C. **TLC:** R_f = 0.65 (PE/EA 2:1), $KMnO_4$ stain. **^{1}H NMR** (600 MHz, $CDCl_3$): δ [ppm] = 7.88 – 7.77 (m, 2H, Ar), 7.71 (d, *J* 7.7 Hz, 1H, Ar), 7.59 (t, *J* 7.8 Hz, 1H, Ar), 4.44 (s, 1H, H-5), 3.65 (d, *J* 11.7 Hz, 1H, H-4a), 3.45 (d, *J* 11.7 Hz, 1H, H-4b), 3.02 (s, 3H, H-1, Me), 1.38 (s, 9H, *t*Bu). **^{13}C NMR** (150 MHz, $CDCl_3$): δ [ppm] = 171.5 (s, C-2, amide), 154.9 (s, C-7, urea), 138.8 (s, Ar), 132.8 (d, Ar), 130.3 (d, Ar), 130.2 (d, Ar), 129.3 (d, Ar), 118.3 (s, Ar), 113.7 (s, Ar), 78.5 (d, C-5), 76.4 (s, C-3), 39.1 (t, C-4), 34.5 (s, *t*Bu), 28.5 (q, C-6, *t*Bu), 25.8 (q, C-1, Me). **FT-IR (ATR):** $\tilde{\nu}$ [cm^{-1}] = 2980 (br w), 2231 (w), 1780 (w), 1716 (vs), 1441 (m), 1389 (m), 1293 (w), 1271 (w), 1233 (w), 1084 (w), 998 (w), 941 (w), 801 (w), 755 (m), 687 (w). **HR-MS:** (ESI) = *m/z* calcd. for: $C_{17}H_{19}N_3O_2S$ $[M]^+$ 329.1198 u, found: 329.1187 u. **$[a]_\lambda^T$:** (c = 1.00 g/100 mL, $CHCl_3$) = $[a]_D^{20}$: –9.60°.

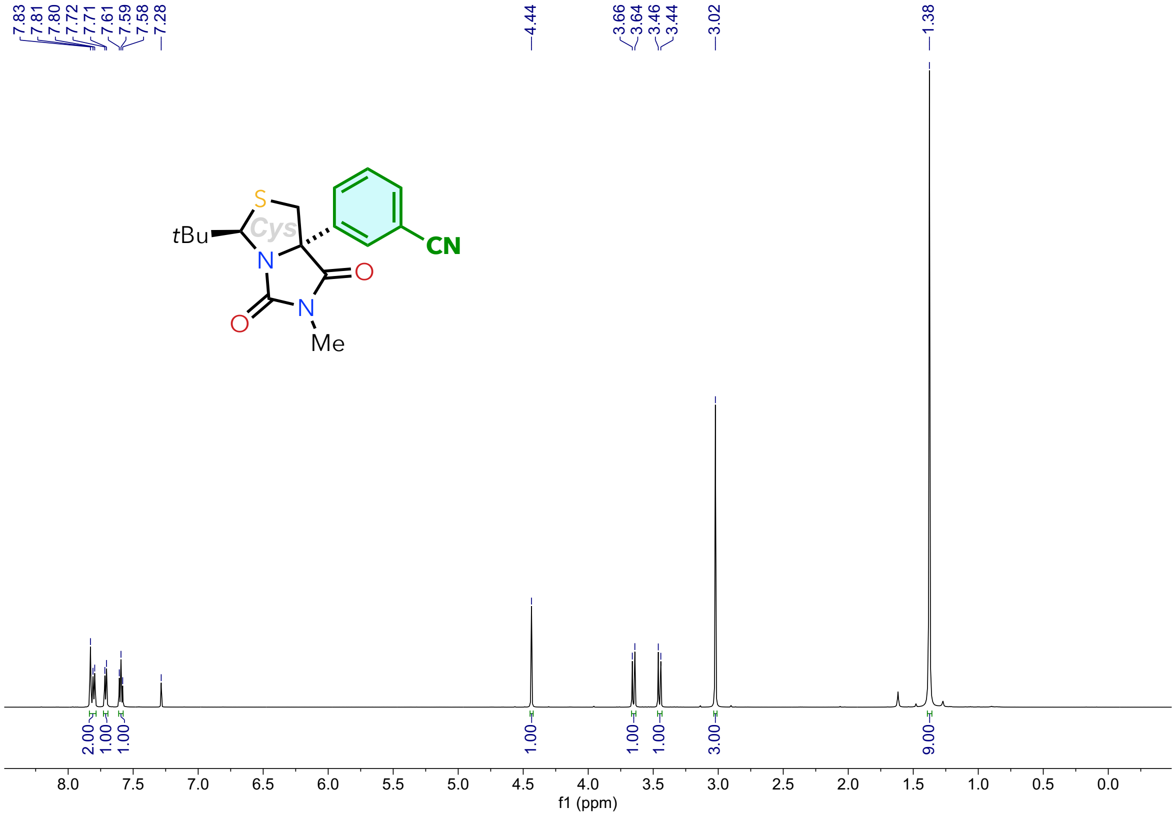
7.83 7.81 7.80 7.72 7.71 7.61 7.59 7.58 7.28
4.44
3.66 3.64 3.46 3.44
3.02
1.38
S
Cys
tBu
N
O
N
O
Me
CN
2.00 1.00 1.00
1.00
1.00 1.00
3.00
9.00
8.0 7.5 7.0 6.5 6.0 5.5 5.0 4.5 4.0 3.5 3.0 2.5 2.0 1.5 1.0 0.5 0.0
f1 (ppm)

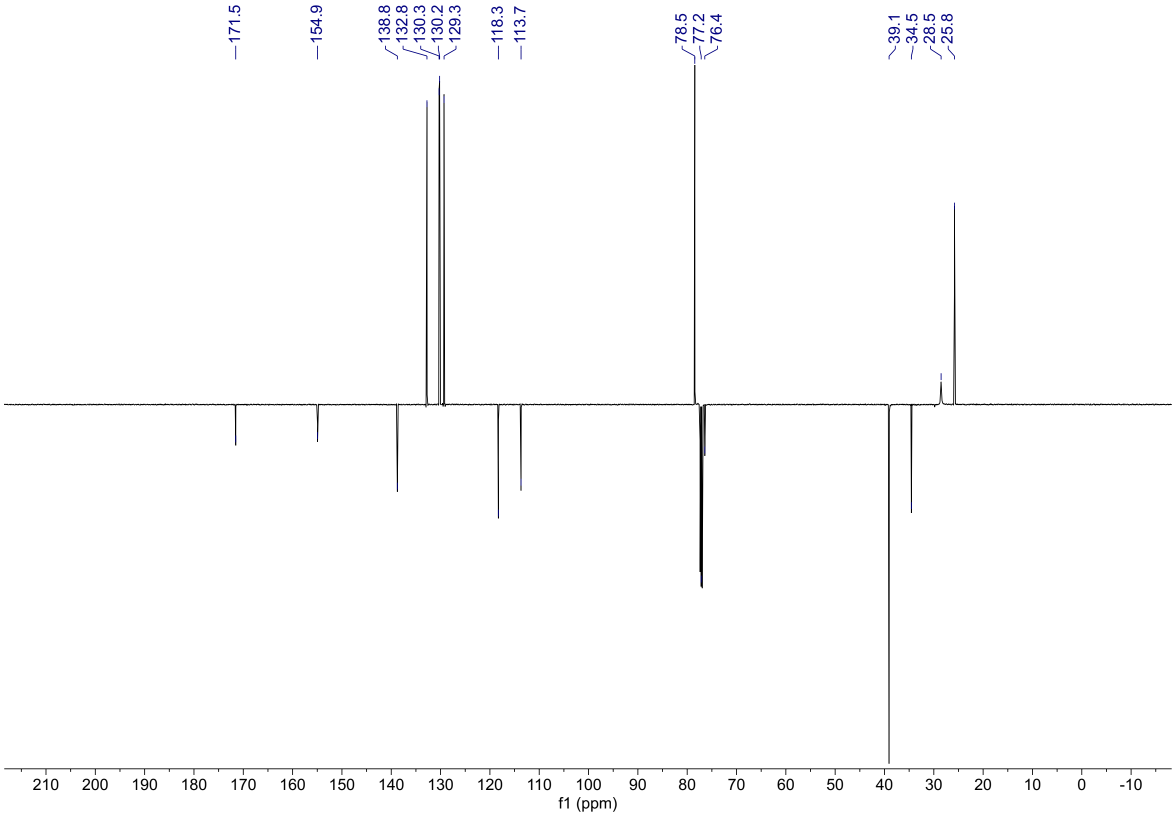
171.5
154.9
138.8 132.8 130.3 130.2 129.3
118.3
113.7
78.5 77.2 76.4
39.1 34.5 28.5 25.8
210 200 190 180 170 160 150 140 130 120 110 100 90 80 70 60 50 40 30 20 10 0 -10
f1 (ppm)

(3*R*,7a*R*)-3-(*tert*-butyl)-7a-(3-ethynylphenyl)-6-methyldihydro-3*H*,5*H*-imidazo[1,5-*c*]thiazole-5,7(6*H*)-dione (23h)

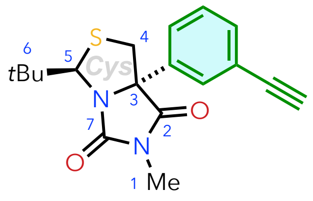

23h

Following **GP4**, to a solution of *N*-aryl urea **19h** (100 mg, 0./.728 mmol, 1.0 equiv.) in dry THF (2.8 mL, 0.1 M) at –78 °C was added potassium bis(trimethylsilyl)amide solution (0.42 mL, 0.42 mmol, 1 M in THF, 1.5 equiv.) dropwise. The title compound **23h** was obtained as a pale yellow solid (33 mg, 0.10 mmol, 36%) after purification by silica gel column chromatography (gradient elution, PE/EA).

Formula: $C_{18}H_{20}N_2O_2S$, **MW:** 328.43 g/mol, **m.p.:** 116 – 119 °C. **TLC:** R_f = 0.66 (PE/EA 2:1), $KMnO_4$ stain. **^{1}H NMR** (600 MHz, $CDCl_3$): δ [ppm] = 7.63 (s, 1H, Ar), 7.50 (d, *J* 7.5 Hz, 2H, Ar), 7.40 (t, *J* 7.8 Hz, 1H, Ar), 4.46 (s, 1H, H-5), 3.57 (d, *J* 11.6 Hz, 1H, H-4a), 3.48 (d, *J* 11.6 Hz, 1H, H-4b), 3.13 (s, 1H, Ar, ≡CH), 2.99 (s, 3H, H-1, Me), 1.35 (s, 9H, *t*Bu). **^{13}C NMR** (150 MHz, $CDCl_3$): δ [ppm] = 172.0 (s, C-2), 154.9 (s, Ar), 137.3 (s, Ar), 132.8 (d, Ar), 129.5 (d, Ar), 129.2 (d, Ar), 126.1 (d, Ar), 123.4 (s, Ar), 83.0 (s, Ar, C≡), 78.4 (s, Ar, ≡CH), 77.9 (d, C-5), 76.7 (s, C-3), 38.6 (t, C-4), 34.4 (s, *t*Bu), 28.5 (q, C-6, *t*Bu), 25.7 (q, C-1, Me). **FT-IR (ATR):** $\tilde{\nu}$ [cm^{-1}] = 2959 (br w), 1779 (w), 1713 (vs), 1439 (m), 1388 (m), 1366 (w), 1292 (w), 1234 (w), 1044 (w), 997 (w), 798 (w), 691 (w), 643 (m), 463 (w). **HR-MS:** (ESI) = *m*/*z* calcd. for: $C_{18}H_{21}N_2O_2S$ $[M+H]^+$ 329.1324 u, found: 329.1324 u. **$[a]_{\lambda}^{T}$:** (c = 0.99 g/100 mL, $CHCl_3$) = $[a]_D^{20}$: –13.74°.

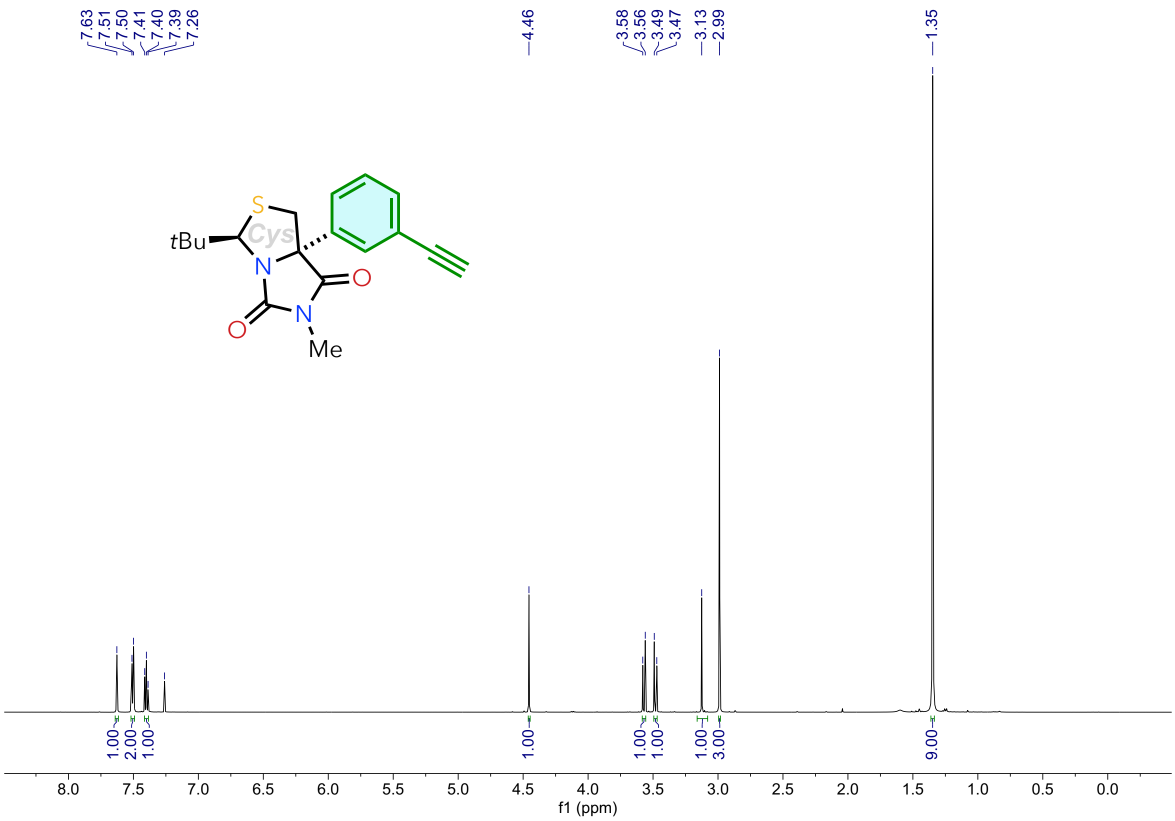
7.63
7.51
7.50
7.41
7.40
7.39
7.26
4.46
3.58
3.56
3.49
3.47
3.13
2.99
1.35
S
Cys
tBu
N
O
N
O
Me
1.00
2.00
1.00
1.00
1.00
1.00
1.00
3.00
9.00
8.0 7.5 7.0 6.5 6.0 5.5 5.0 4.5 4.0 3.5 3.0 2.5 2.0 1.5 1.0 0.5 0.0
f1 (ppm)

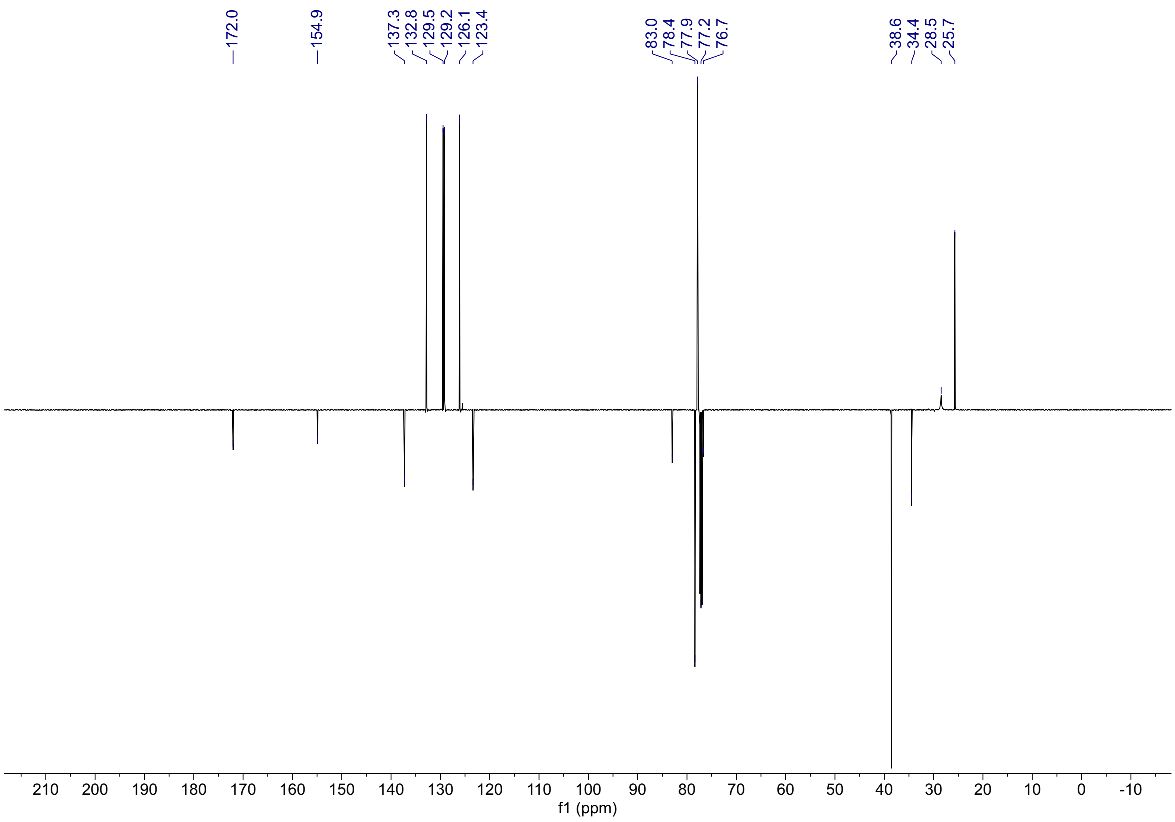
172.0
154.9
137.3
132.8
129.5
129.2
126.1
123.4
83.0
78.4
77.9
77.2
76.7
38.6
34.4
28.5
25.7
210 200 190 180 170 160 150 140 130 120 110 100 90 80 70 60 50 40 30 20 10 0 -10
f1 (ppm)

Methyl (3*R*,10b*R*)-3-(*tert*-butyl)-6-methyl-7-nitro-5-oxo-5,6-dihydro-3*H*-thiazolo[3,4-*c*]quinazoline-10b(1*H*)-carboxylate (23i)

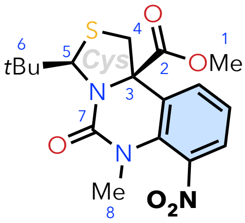

23i

Following **GP4**, to a solution of *N*-aryl urea **19i** (100 mg, 0.30 mmol, 1.0 equiv.) in dry THF (3.0 mL, 0.1 M) at –78 °C was added potassium bis(trimethylsilyl)amide solution (0.45 mL, 0.45 mmol, 1 M in THF, 1.5 equiv.) dropwise. The title compound **23i** was obtained as an orange solid (32 mg, 0.08 mmol, 28%) after purification by silica gel column chromatography (gradient elution, PE/EA).

Formula: $C_{17}H_{21}N_3O_5S$, **MW:** 379.43 g/mol. **TLC:** R_f = 0.57 (PE/EA 2:1), $KMnO_4$ stain. **^{1}H NMR** (400 MHz, $CDCl_3$): δ [ppm] = 7.88 (d, *J* 10.7 Hz, 1H, Ar), 7.70 (s, 1H, Ar), 7.49 (d, *J* 8.6 Hz, 1H, Ar), 5.52 (s, 1H, H-5), 4.26 (d, *J* 12.2 Hz, 1H, H-4a), 3.68 (s, 3H, H-1, OMe), 3.63 (d, *J* 12.2 Hz, 1H, H-4b), 3.41 (s, 3H, H-8, NMe), 1.06 (s, 9H, H-6, *t*Bu). **FT-IR (ATR):** $\tilde{\nu}$ [cm^{-1}] = 2957 (br w), 1740 (m), 1676 (m), 1621 (w), 1533 (s), 1479 (w), 1349 (vs), 1313 (m), 1260 (m), 1174 (w), 1118 (w), 738 (m). **HR-MS:** (ESI) = *m*/*z* calcd. for: $C_{17}H_{22}N_3O_5S$ $[M+H]^+$ 380.1280 u, found: 380.1269 u.

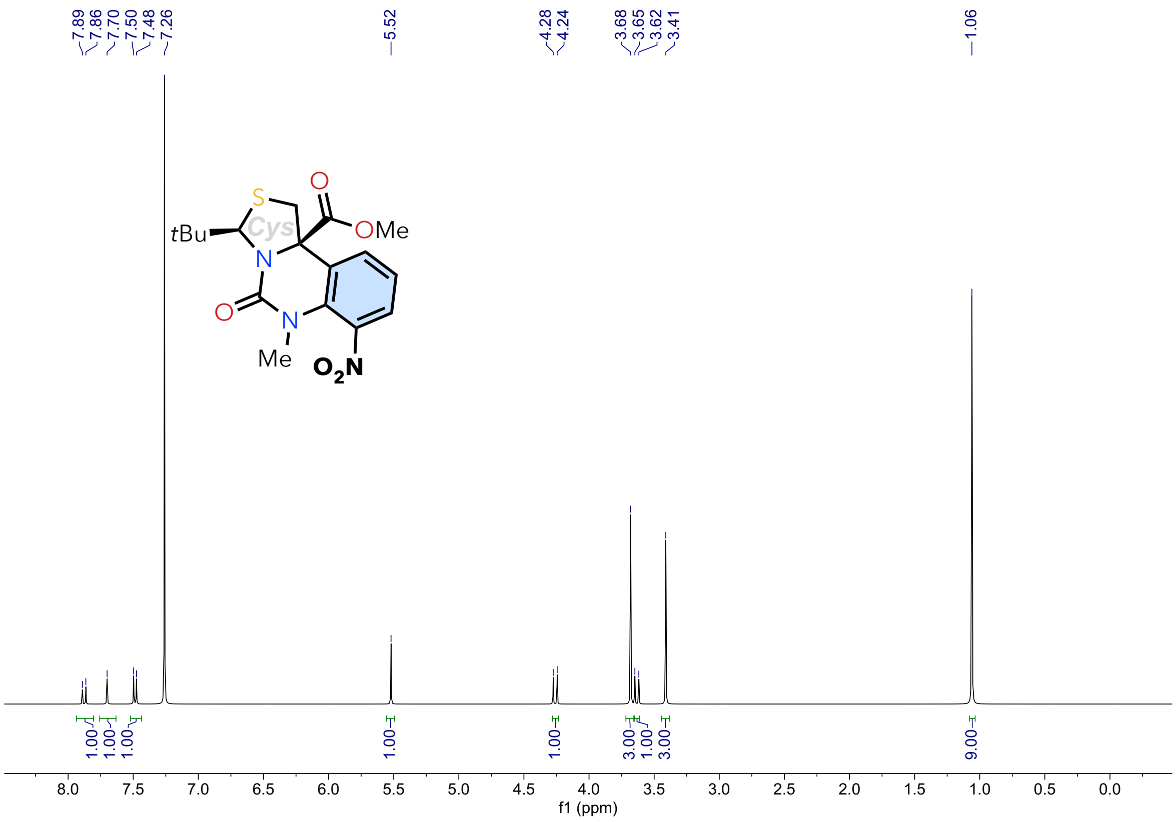
7.89
7.86
7.70
7.50
7.48
7.26
5.52
4.28
4.24
3.68
3.65
3.62
3.41
1.06
tBu
S
Cys
N
O
OMe
N
Me
O_2N
1.00
1.00
1.00
1.00
1.00
3.00
1.00
3.00
9.00
8.0
7.5
7.0
6.5
6.0
5.5
5.0
4.5
4.0
3.5
3.0
2.5
2.0
1.5
1.0
0.5
0.0
f1 (ppm)

(3R,7aR)-3-(*tert*-Butyl)-7a-(3-methoxyphenyl)-6-methyldihydro-3H,5H-imidazo[1,5-*c*]thiazole-5,7(6H)-dione (23j)

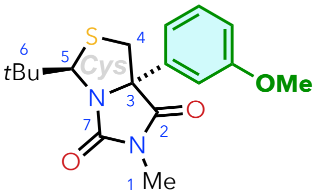

23j

Following **GP4**, to a solution of *N*-aryl urea **19j** (100 mg, 0.27 mmol, 1.0 equiv.) in dry THF (2.7 mL, 0.1 M) at –78 °C was added potassium bis(trimethylsilyl)amide solution (0.41 mL, 0.41 mmol, 1 M in THF, 1.5 equiv.) dropwise. The title compound **23j** was obtained as a colourless oil (86 mg, 0.26 mmol, 94%) after purification by silica gel column chromatography (gradient elution, PE/EA).

Formula: $C_{17}H_{22}N_2O_3S$, **MW:** 334.43 g/mol. **TLC:** R_f = 0.67 (PE/EA 2:1), $KMnO_4$ stain. **^{1}H NMR** (500 MHz, $CDCl_3$): δ [ppm] = 7.35 (t, *J* 8.0 Hz, 1H, Ar), 7.10 (ddd, *J* 7.8, 1.8, 0.8 Hz, 1H, Ar), 7.05 (t, *J* 2.2 Hz, 1H, Ar), 6.91 (ddd, *J* 8.3, 2.5, 0.8 Hz, 1H, Ar), 4.50 (s, 1H, H-5), 3.82 (s, 3H, Ar, OMe), 3.56 (d, *J* 11.5 Hz, 1H, H-4a), 3.51 (d, *J* 11.5 Hz, 1H, H-4b), 2.98 (s, 3H, H-1, Me), 1.35 (s, 9H, *t*Bu). **^{13}C NMR** (125 MHz, $CDCl_3$): δ [ppm] = 172.3 (s, C-2, amide), 160.3 (s, Ar), 154.9 (s, C-7, urea), 138.4 (s, Ar), 130.5 (d, Ar), 117.8 (d, Ar), 114.2 (d, Ar), 111.5 (d, Ar), 77.7 (d, C-5), 76.8 (s, C-3), 55.5 (q, Ar, OMe), 38.6 (t, C-4), 34.3 (s, *t*Bu), 28.5 (q, C-6, *t*Bu), 25.6 (q, C-1, Me). **FT-IR (ATR):** $\tilde{\nu}$ [cm^{-1}] = 2970 (br w), 1778 (w), 1714 (vs), 1600 (w), 1436 (m), 1388 (m), 1289 (m), 1269 (m), 1178 (w), 1151 (w), 1042 (m), 877 (w), 757 (m), 691 (m), 644 (w). **HR-MS:** (ESI) = *m*/*z* calcd. for: $C_{17}H_{23}N_2O_3S$ $[M+H]^+$ 335.1429 u, found: 335.1415 u. **$[a]_\lambda^T$:** (c = 1.00 g/100 mL, $CHCl_3$) = $[a]_D^{20}$: –30.80°.

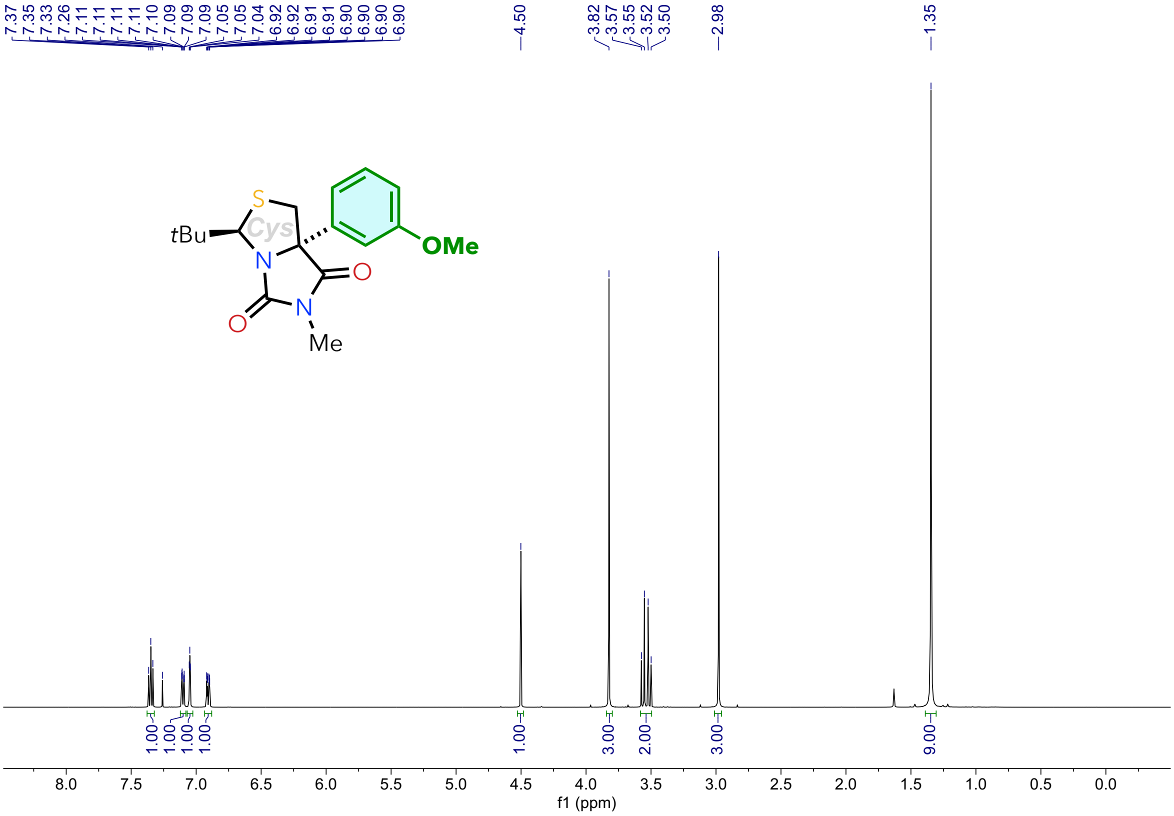
7.37
7.35
7.33
7.26
7.11
7.11
7.11
7.11
7.10
7.09
7.09
7.09
7.05
7.05
7.04
6.92
6.92
6.91
6.91
6.90
6.90
6.90
6.90
4.50
3.82
3.57
3.55
3.52
3.50
2.98
1.35
tBu
S
Cys
N
O
N
Me
O
OMe
1.00
1.00
1.00
1.00
1.00
3.00
2.00
3.00
9.00
f1 (ppm)

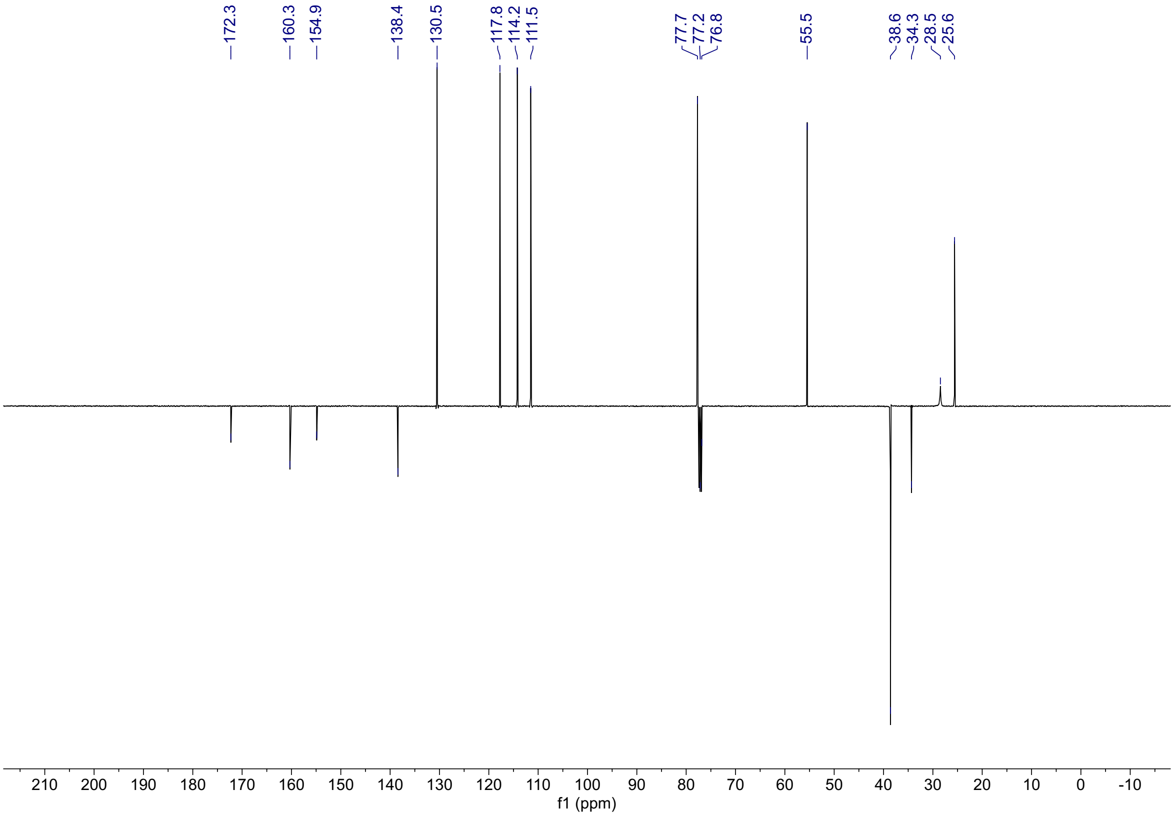
172.3
160.3
154.9
138.4
130.5
117.8
114.2
111.5
77.7
77.2
76.8
55.5
38.6
34.3
28.5
25.6
f1 (ppm)

(3*R*,7a*R*)-3-(*tert*-butyl)-6-methyl-7a-(*p*-tolyl)dihydro-3*H*,5*H*-imidazo[1,5-*c*]thiazole-5,7(6*H*)-dione (23k)

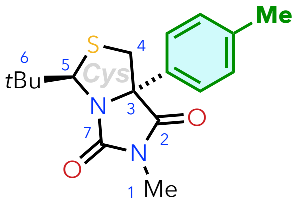

23k

Following **GP4**, to a solution of *N*-aryl urea **19k** (100 mg, 0.29 mmol, 1.0 equiv.) in dry THF (2.9 mL, 0.1 M) at –78 °C was added potassium bis(trimethylsilyl)amide solution (0.43 mL, 0.43 mmol, 1 M in THF, 1.5 equiv.) dropwise. The title compound **23k** was obtained as a colourless oil (63 mg, 0.20 mmol, 69%) after purification by silica gel column chromatography (gradient elution, PE/EA).

Formula: $C_{17}H_{22}N_2O_2S$, **MW:** 318.44 g/mol. **TLC:** R_f = 0.77 (PE/EA 2:1), $KMnO_4$ stain. **^{1}H NMR** (500 MHz, $CDCl_3$): δ [ppm] = 7.39 (d, *J* 8.3 Hz, 2H, Ar), 7.24 (d, *J* 8.0 Hz, 2H, Ar), 4.49 (s, 1H, H-5), 3.52 (s, 2H, H-4), 2.98 (s, 3H, H-1, Me), 2.36 (s, 3H, H-1, Ar, Me), 1.34 (s, 9H, *t*Bu). **^{13}C NMR** (125 MHz, $CDCl_3$): δ [ppm] = 172.5 (s, C-2, amide), 154.7 (s, C-7, urea), 139.2 (s, Ar), 133.7 (s, Ar), 130.1 (d, 2Ar), 125.4 (d, 2Ar), 77.3 (d, C-5), 76.9 (s, C-3), 38.2 (t, C-4), 34.3 (s, *t*Bu), 28.4 (q, C-6, *t*Bu), 25.6 (q, C-1, Me), 25.2 (q, Ar, Me). **FT-IR (ATR):** $\tilde{\nu}$ [cm^{-1}] = 2980 (br w), 1777 (w), 1714 (vs), 1439 (m), 1388 (m), 1294 (w), 1269 (w), 1231 (w), 1045 (w), 1000 (w), 758 (w), 739 (w), 661 (w), 510 (m). **HR-MS:** (ESI) = *m/z* calcd. for: $C_{17}H_{23}N_2O_2S$ $[M+H]^+$ 319.1480 u, found: 319.1464 u. **$[a]_\lambda^T$:** (c = 1.00 g/100 mL, $CHCl_3$) = $[a]_D^{20}$: –10.80°.

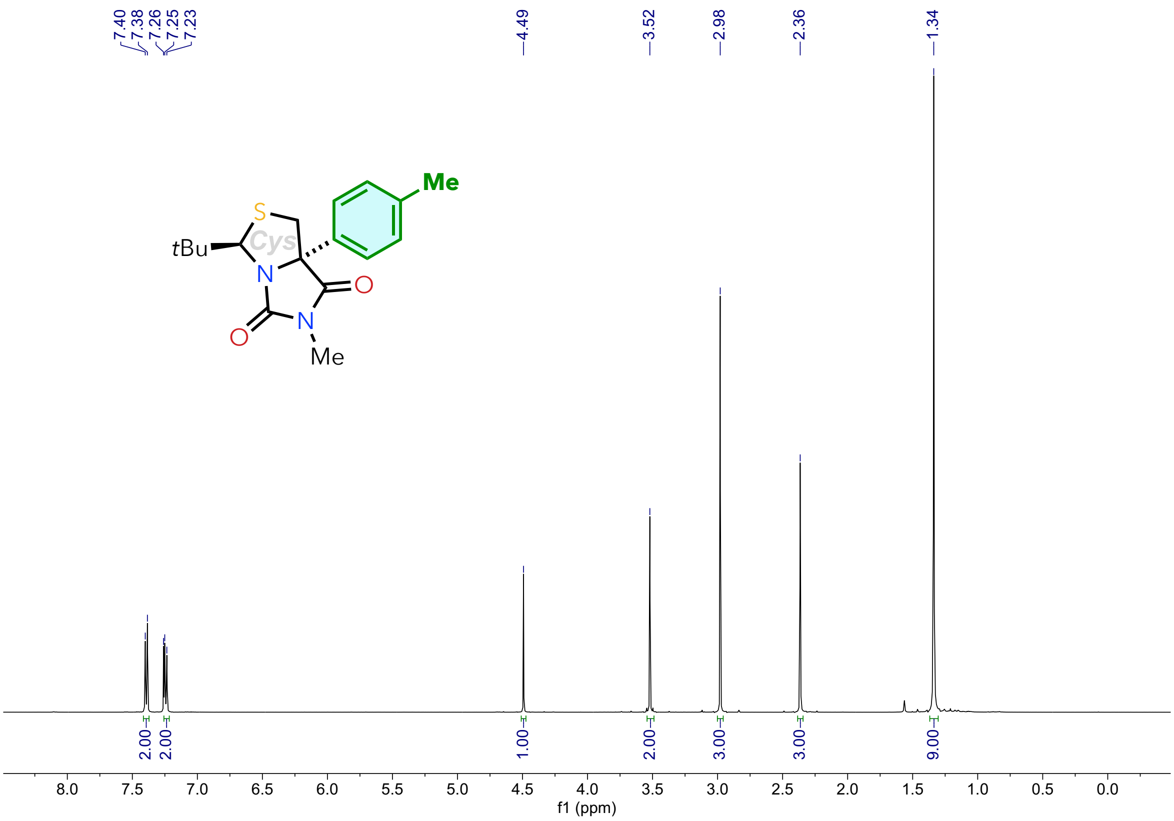
7.40
7.38
7.26
7.25
7.23
4.49
3.52
2.98
2.36
1.34
S
tBu
Cys
N
O
N
Me
O
Me
2.00
2.00
1.00
2.00
3.00
3.00
9.00
f1 (ppm)

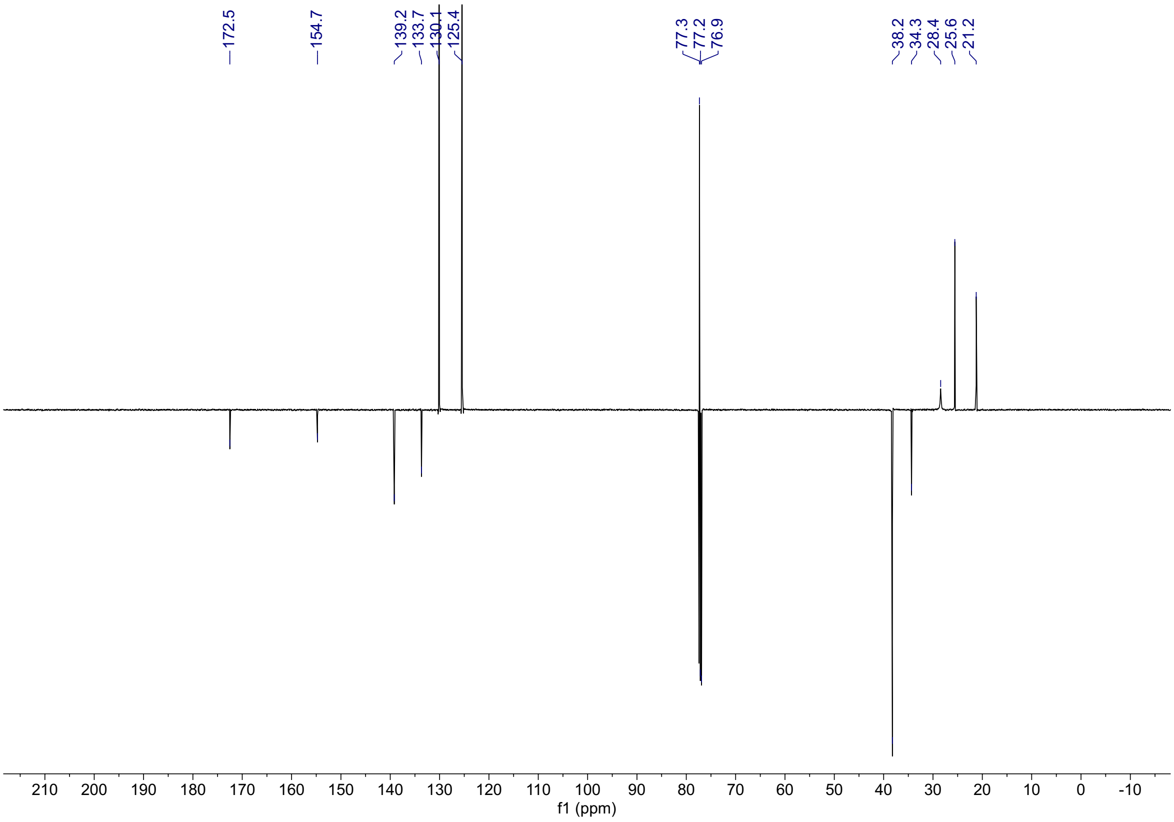
172.5
154.7
139.2
133.7
130.1
125.4
77.3
77.2
76.9
38.2
34.3
28.4
25.6
21.2
f1 (ppm)

(3*R*,7a*R*)-3-(*tert*-butyl)-7a-(3,5-difluorophenyl)-6-methyldihydro-3*H*,5*H*-imidazo[1,5-*c*]thiazole-5,7(6*H*)-dione (231)

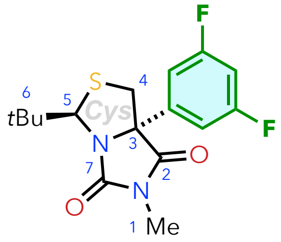

231

Following **GP4**, to a solution of *N*-aryl urea **191** (100 mg, 0.27 mmol, 1.0 equiv.) in dry THF (2.7 mL, 0.1 M) at –78 °C was added potassium bis(trimethylsilyl)amide solution (0.40 mL, 0.40 mmol, 1 M in THF, 1.5 equiv.) dropwise. The title compound **231** was obtained as a yellowish oil (37 mg, 0.11 mmol, 41%) after purification by silica gel column chromatography (gradient elution, PE/EA).

Formula: $C_{16}H_{18}F_2N_2O_2S$, **MW:** 340.39 g/mol. **TLC:** R_f = 0.74 (PE/EA 2:1), $KMnO_4$ stain. **^{1}H NMR** (600 MHz, $CDCl_3$): δ [ppm] = 7.06 (dd, *J* 8.1, 2.2 Hz, 2H, Ar), 6.83 (tt, *J* 8.6, 2.3 Hz, 1H, Ar), 4.43 (s, 1H, H-5), 3.59 (d, *J* 11.7 Hz, 1H, H-4a), 3.41 (d, *J* 11.7 Hz, 1H, H-4b), 2.99 (s, 3H, H-1, Me), 1.35 (s, 9H, *t*Bu). **^{13}C NMR** (150 MHz, $CDCl_3$): δ [ppm] = 171.4 (s, C-2, amide), 164.4 (s, *d J* 12.6 Hz, Ar), 162.7 (s, *d J* 12.6 Hz, Ar), 154.9 (s, C-7, urea), 141.0 (s, *t J* 8.9 Hz, Ar), 109.0 (d, *dd J* 21.3, 6.0 Hz, Ar), 78.4 (d, C-5), 76.5 (s, *t J* 2.2 Hz, C-3), 39.0 (t, C-4), 34.5 (s, *t*Bu), 28.5 (q, C-6, *t*Bu), 25.8 (q, C-1, Me). **^{19}F NMR** (565 MHz, $CDCl_3$, C_6F_6 ref.): δ [ppm] = –110.36 (t, *J* 7.9 Hz, 2F). **FT-IR (ATR):** $\tilde{\nu}$ [cm^{-1}] = 2966 (br w), 1775 (w), 1717 (m), 1623 (w), 1599 (w), 1455 (w), 1435 (w), 1324 (w), 1306 (w), 1115 (w), 1054 (s), 1033 (vs), 1014 (m), 848 (w), 660 (w). **HR-MS:** (ESI) = *m/z* calcd. for: $C_{15}H_{15}F_2N_2O_2S$ $[M–Me]^+$ 325.0822 u, found: 325.0811 u. **$[a]_\lambda^T$:** (c = 1.02 g/100 mL, $CHCl_3$) = $[a]_D^{20}$: –10.98°.

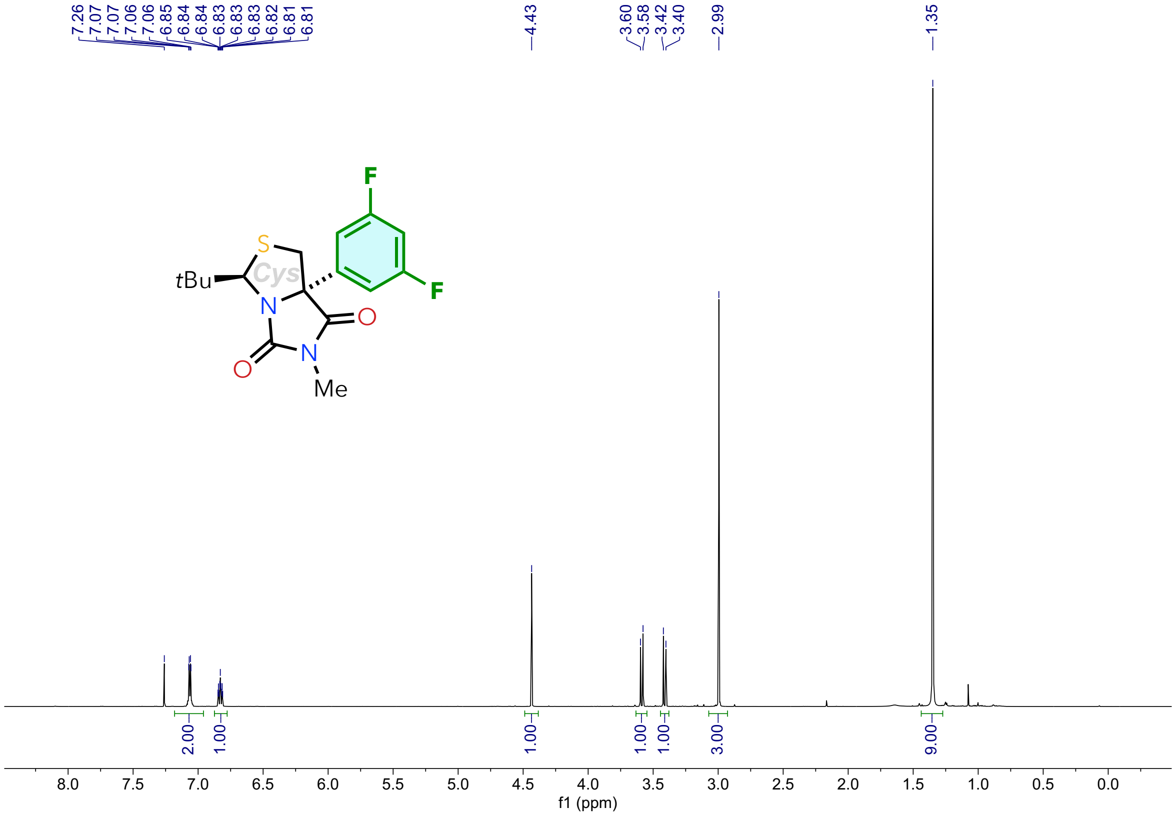

7.26
7.07
7.07
7.06
6.85
6.84
6.84
6.83
6.83
6.83
6.82
6.81
6.81
4.43
3.60
3.58
3.42
3.40
2.99
1.35
F
S
Cys
tBu
N
O
N
O
F
Me
2.00
1.00
1.00
1.00
1.00
3.00
9.00
8.0 7.5 7.0 6.5 6.0 5.5 5.0 4.5 4.0 3.5 3.0 2.5 2.0 1.5 1.0 0.5 0.0
f1 (ppm)

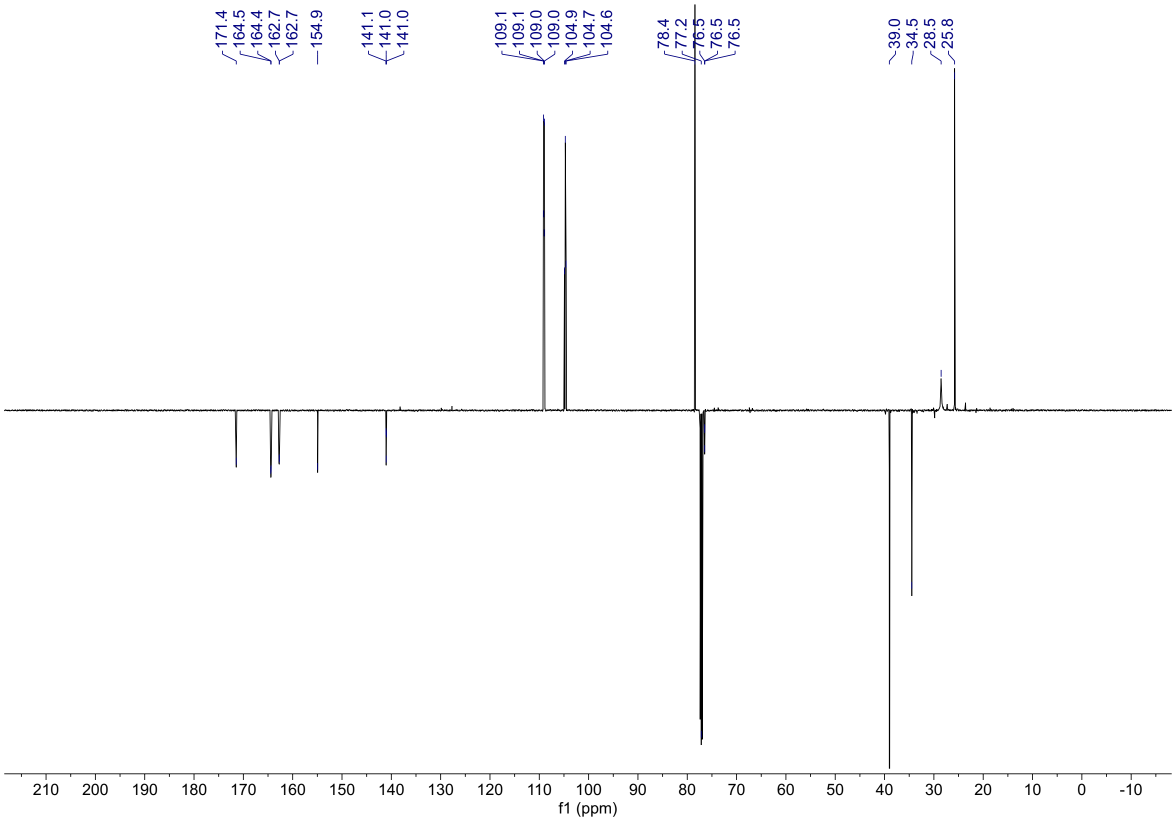

171.4
164.5
164.4
162.7
162.7
154.9
141.1
141.0
141.0
109.1
109.1
109.0
109.0
104.9
104.7
104.6
78.4
77.2
76.5
76.5
76.5
39.0
34.5
28.5
25.8
210 200 190 180 170 160 150 140 130 120 110 100 90 80 70 60 50 40 30 20 10 0 -10
f1 (ppm)

(3*R*,7a*R*)-3-(*tert*-butyl)-6-methyl-7a-(pyridin-2-yl)dihydro-3*H*,5*H*-imidazo[1,5-*c*]thiazole-5,7(6*H*)-dione (23m)

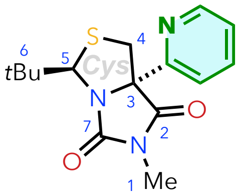

23m

Following **GP4**, to a solution of *N*-aryl urea **19m** (100 mg, 0.30 mmol, 1.0 equiv.) in dry THF (3.0 mL, 0.1 M) at –78 °C was added potassium bis(trimethylsilyl)amide solution (0.45 mL, 0.45 mmol, 1 M in THF, 1.5 equiv.) dropwise. The title compound **23m** was obtained as a yellow oil (32 mg, 0.10 mmol, 35%) after purification by silica gel column chromatography (gradient elution, PE/EA).

Formula: $C_{15}H_{19}N_3O_2S$, **MW:** 305.40 g/mol. **TLC:** R_f = 0.52 (PE/EA 2:1), $KMnO_4$ stain. **^{1}H NMR** (500 MHz, $CDCl_3$): δ [ppm] = 8.73 (ddd, *J* 4.8, 1.8, 0.9 Hz, 1H, Ar), 7.76 (td, *J* 7.8, 1.8 Hz, 1H, Ar), 7.50 (dt, *J* 7.9, 1.0 Hz, 1H, Ar), 7.33 (ddd, *J* 7.6, 4.8, 1.1 Hz, 1H, Ar), 4.47 (s, 1H, H-5), 4.13 (d, *J* 11.2 Hz, 1H, H-4a), 3.37 (d, *J* 11.2 Hz, 1H, H-4b), 3.02 (s, 3H, H-1, Me), 1.31 (s, 9H, *t*Bu). **^{13}C NMR** (125 MHz, $CDCl_3$): δ [ppm] = 170.9 (s, C-2, amide), 154.4 (s, C-7, urea), 154.2 (s, Ar), 150.5 (d, Ar), 137.6 (d, Ar), 124.0 (d, Ar), 119.9 (d, Ar), 78.7 (s, C-3), 76.6 (d, C-5), 35.2 (t, C-4), 34.4 (s, *t*Bu), 28.3 (q, C-6, *t*Bu), 25.7 (q, C-1, Me). **FT-IR (ATR):** $\tilde{\nu}$ [cm^{-1}] = 2980 (br m), 1776 (w), 1712 (vs), 1585 (w), 1434 (s), 1390 (m), 1292 (m), 1154 (m), 1131 (m), 1081 (m), 992 (w), 955 (w), 758 (m), 649 (w), 504 (w). **HR-MS:** (ESI) = *m*/*z* calcd. for: $C_{15}H_{20}N_3O_2S$ $[M+H]^+$ 306.1276 u, found: 306.1260 u. **$[a]_\lambda^T$:** (c = 1.00 g/100 mL, $CHCl_3$) = $[a]_D^{20}$: –8.80°.

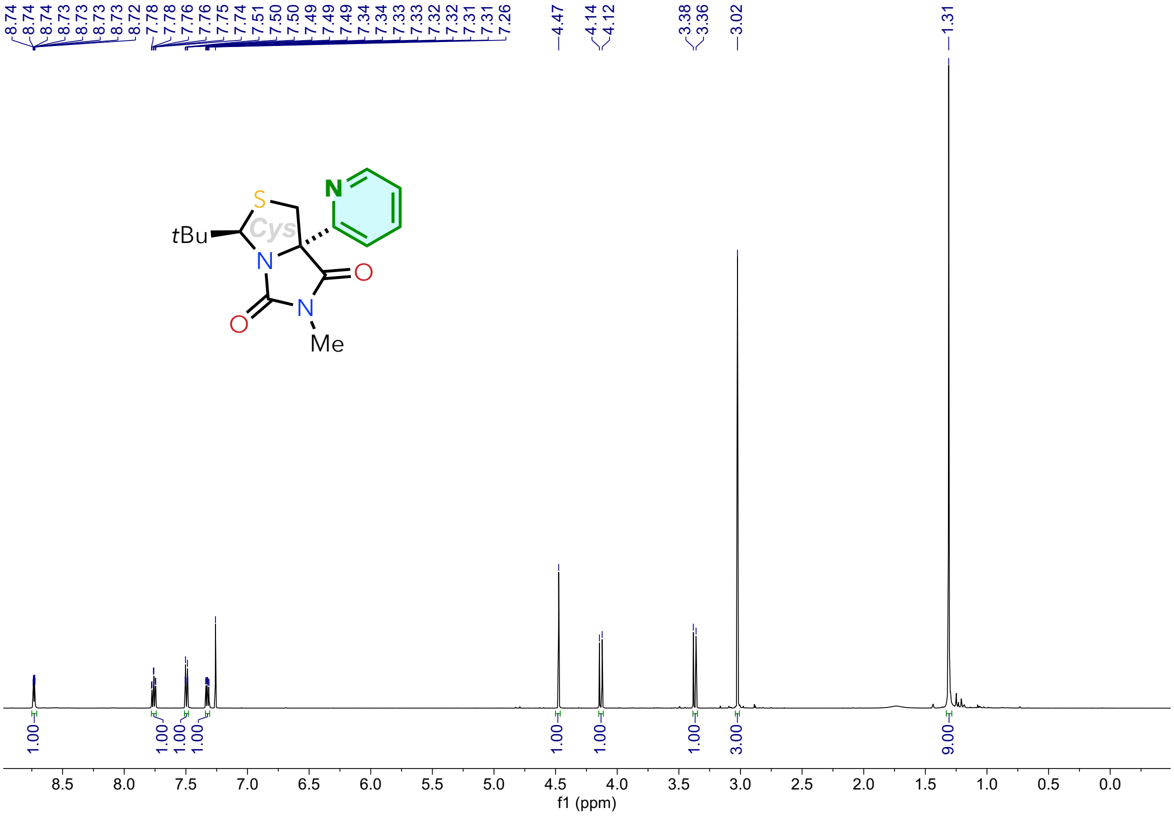
8.74
8.74
8.74
8.73
8.73
8.73
8.73
8.72
7.78
7.78
7.76
7.76
7.75
7.74
7.51
7.50
7.50
7.49
7.49
7.49
7.34
7.33
7.33
7.32
7.32
7.31
7.31
7.26
4.47
4.14
4.12
3.38
3.36
3.02
1.31
S
Cys
N
tBu
N
O
N
O
Me
1.00
1.00
1.00
1.00
1.00
1.00
1.00
3.00
9.00
8.5
8.0
7.5
7.0
6.5
6.0
5.5
5.0
4.5
4.0
3.5
3.0
2.5
2.0
1.5
1.0
0.5
0.0
f1 (ppm)

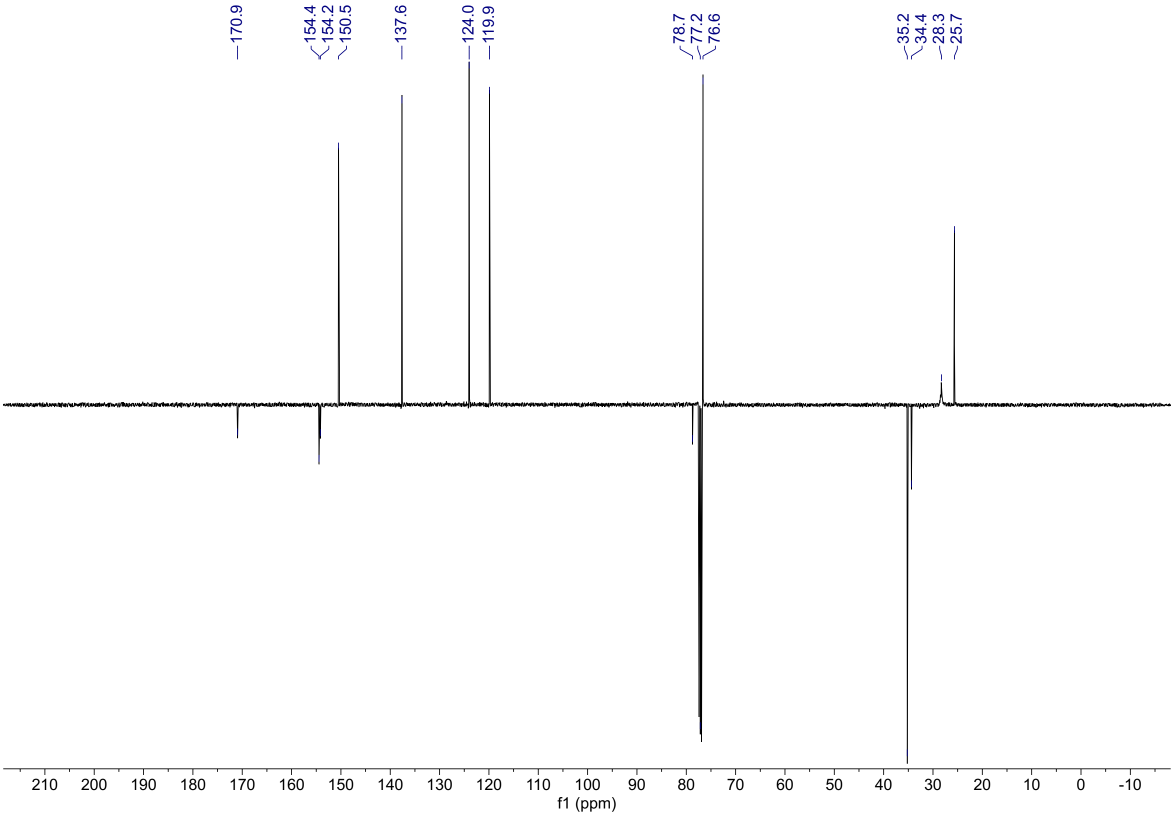
170.9
154.4
154.2
150.5
137.6
124.0
119.9
78.7
77.2
76.6
35.2
34.4
28.3
25.7
210
200
190
180
170
160
150
140
130
120
110
100
90
80
70
60
50
40
30
20
10
0
-10
f1 (ppm)

(3*R*,7a*R*)-3-(*tert*-Butyl)-6-methyl-7a-(naphthalen-2-yl)dihydro-3*H*,5*H*-imidazo[1,5-*c*]thiazole-5,7(6*H*)-dione (23n)

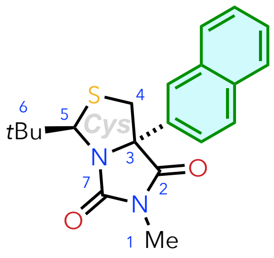

23n

Following **GP4**, to a solution of *N*-aryl urea **19n** (100 mg, 0.26 mmol, 1.0 equiv.) in dry THF (2.6 mL, 0.1 M) at –78 °C was added potassium bis(trimethylsilyl)amide solution (0.39 mL, 0.39 mmol, 1 M in THF, 1.5 equiv.) dropwise. The title compound **23n** was obtained as a white solid (76 mg, 0.21 mmol, 83%) after purification by silica gel column chromatography (gradient elution, PE/EA).

Formula: $C_{20}H_{22}N_2O_2S$, **MW:** 354.47 g/mol, **m.p.:** 176 – 179 °C. **TLC:** R_f = 0.76 (PE/EA 2:1), $KMnO_4$ stain. **^{1}H NMR** (500 MHz, $CDCl_3$): δ [ppm] = 8.52 (d, *J* 8.7 Hz, 1H, Ar), 7.89 (t, *J* 7.4 Hz, 2H, Ar), 7.75 (dd, *J* 7.4, 1.0 Hz, 1H, Ar), 7.60 (ddd, *J* 8.5, 6.8, 1.4 Hz, 1H, Ar), 7.53 (ddd, *J* 8.5, 6.8, 1.4 Hz, 1H, Ar), 7.45 (t, *J* 7.4 Hz, 1H, Ar), 4.45 (s, 1H, H-5), 4.00 (d, *J* 11.8 Hz, 1H, H-4a), 3.88 (d, *J* 11.8 Hz, 1H, H-4b), 3.02 (s, 3H, H-1, Me), 1.39 (s, 9H, *t*Bu). **^{13}C NMR** (125 MHz, $CDCl_3$): δ [ppm] = 172.6 (s, C-2, amide), 155.3 (s, C-7, urea), 135.5 (s, Ar), 133.4 (s, Ar), 130.7 (d, Ar), 130.6 (s, Ar), 129.3 (d, Ar), 126.9 (d, Ar), 126.4 (d, Ar), 126.2 (d, Ar), 125.1 (d, Ar), 123.2 (d, Ar), 77.6 (s, C-3), 77.2 (d, C-5), 39.9 (t, C-4), 34.3 (s, *t*Bu), 28.8 (q, C-6, *t*Bu), 25.6 (q, C-1, Me). **FT-IR (ATR):** $\tilde{\nu}$ [cm^{-1}] = 2981 (br w), 1777 (w), 1716 (vs), 1596 (w), 1509 (w), 1445 (m), 1385 (m), 1292 (m), 1271 (w), 1178 (w), 1124 (w), 1092 (w), 1016 (w), 1000 (w), 804 (m), 777 (s), 764 (m), 733 (w), 699 (w), 645 (w), 627 (w). **HR-MS:** (ESI) = *m*/*z* calcd. for: $C_{20}H_{22}N_2O_2S$ $[M]^+$ 354.1402 u, found: 354.1392 u. **$[a]_\lambda^T$:** (c = 1.00 g/100 mL, $CHCl_3$) = $[a]_D^{20}$: –30.80°.

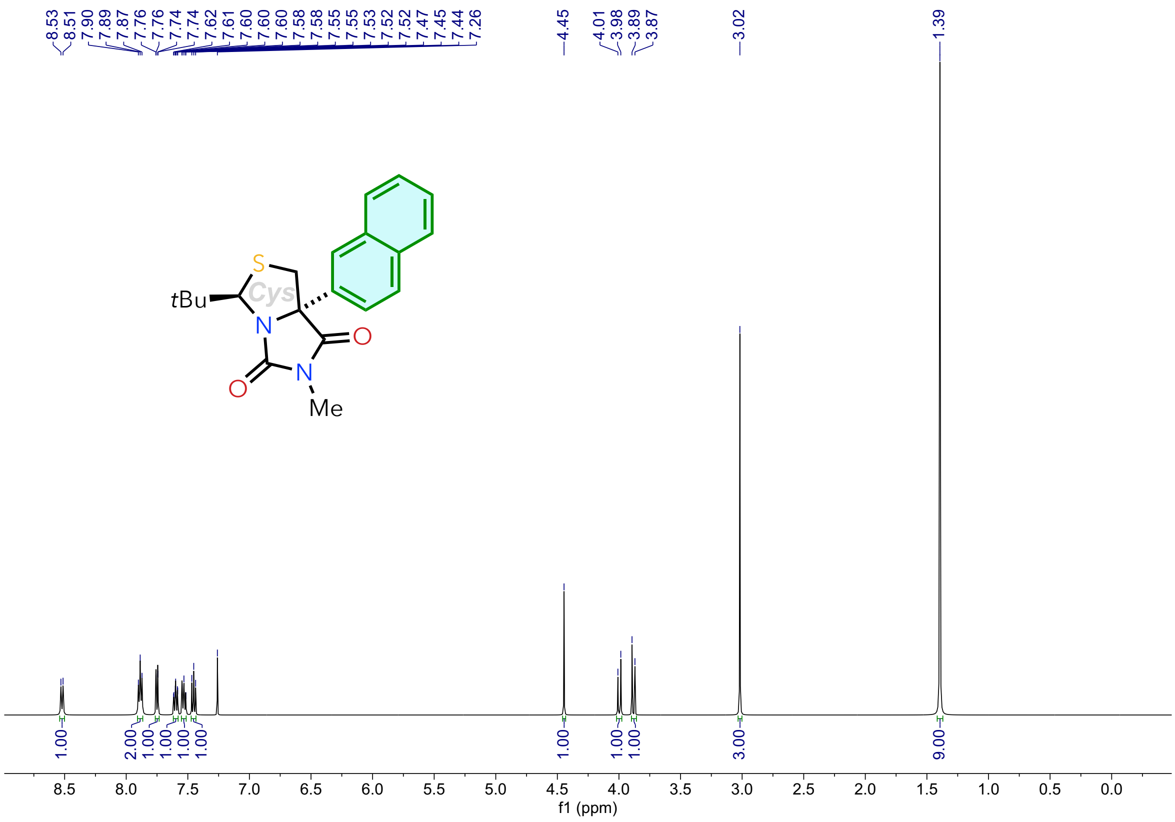
8.53 8.51 7.90 7.89 7.87 7.76 7.76 7.74 7.74 7.62 7.61 7.60 7.60 7.58 7.58 7.55 7.55 7.53 7.52 7.52 7.47 7.45 7.44 7.26
4.45
4.01 3.98 3.89 3.87
3.02
1.39
S
Cys
tBu
N
O
N
O
Me
1.00 2.00 1.00 1.00 1.00 1.00 1.00 1.00 1.00 3.00 9.00
f1 (ppm)

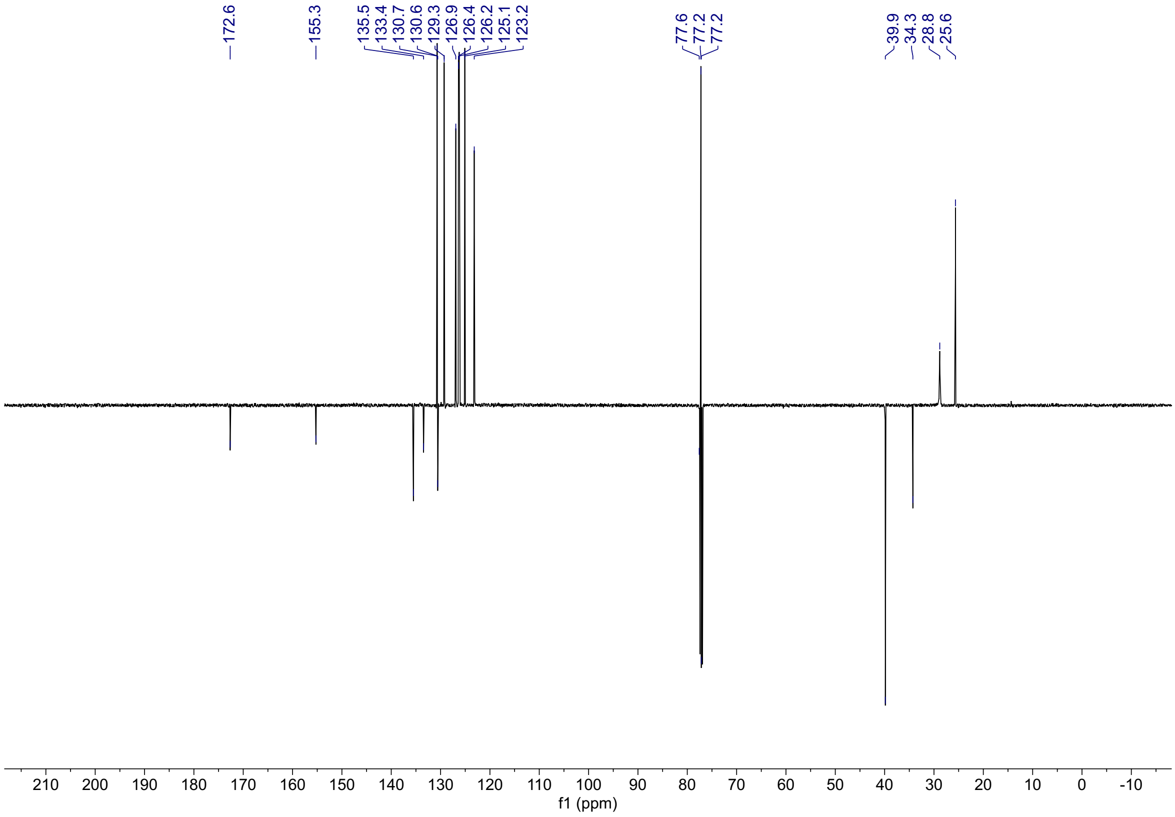
172.6
155.3
135.5 133.4 130.7 130.6 129.3 126.9 126.4 126.2 125.1 123.2
77.6 77.2 77.2
39.9 34.3 28.8 25.6
f1 (ppm)

***tert*-Butyl (3*R*,7a*S*)-3-(*tert*-butyl)-6-methyl-5,7-dioxo-7a-phenyltetrahydro-1*H*-imidazo[1,5-*c*]imidazole-2(3*H*)-carboxylate (24a)**

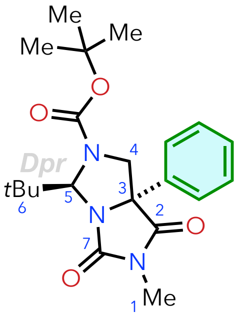

24a

Following **GP4**, to a solution of *N*-aryl urea **20a** (120 mg, 0.29 mmol, 1.0 equiv.) in dry THF (2.9 mL, 0.1 M) at –78 °C was added potassium bis(trimethylsilyl)amide solution (0.43 mL, 0.43 mmol, 1 M in THF, 1.5 equiv.) dropwise. The title compound **24a** was obtained as a white solid (74 mg, 0.19 mmol, 66%) after purification by silica gel column chromatography (gradient elution, PE/EA).

Formula: $C_{21}H_{29}N_3O_4$, **MW:** 387.48 g/mol, **m.p.:** 153 – 156 °C. **TLC:** R_f = 0.56 (PE/EA 2:1), $KMnO_4$ stain. **^{1}H NMR** (600 MHz, $CHCl_3$): δ [ppm] = 7.58 (d, *J* 7.4 Hz, 2H, Ar), 7.42 – 7.30 (m, 3H, Ar), 5.44 (br s, 1H, H-5), 4.42 (br s, 1H, H-4a), 3.47 (d, *J* 10.6 Hz, 1H, H-4b), 2.97 (s, 3H, H-1, Me), 1.16 (s, 9H, H-6, *t*Bu), 1.20 – 1.00 (m, 9H, *t*Bu, Boc). **^{13}C NMR** (150 MHz, $CHCl_3$): δ [ppm] = 171.1 (s, C-2, amide), 155.2 (s, Boc), 154.7 (s, C-7, urea), 136.3 (s, Ar), 129.1 (d, Ar), 128.9 (d, 2Ar), 125.8 (d, 2Ar), 81.3 (d, C-5), 74.4 (s, C-3), 53.9 (t, C-4), 37.8 (s, C-6, *t*Bu), 27.7 (q, Boc, *t*Bu, 3Me), 27.4 (q, C-6, *t*Bu, 3Me), 25.5 (q, C-1, Me). **FT-IR (ATR):** $\tilde{\nu}$ [cm^{-1}] = 2954 (br w), 1772 (w), 1714 (vs); 1691 (s), 1627 (w), 1436 (m), 1362 (s), 1294 (m), 1252 (m), 1160 (s), 1047 (m), 1012 (w), 977 (w), 912 (w), 864 (w), 835 (w), 753 (m), 723 (m), 695 (m), 662 (w), 595 (w), 501 (m). **HR-MS:** (ESI) = *m*/*z* calcd. for: $C_{21}H_{29}N_3O_4Na$ $[M+Na]^+$ 410.2056 u, found: 410.2049 u. **$[a]_\lambda^T$:** (c = 1.02 g/100 mL, CH_3Cl_3) = $[a]_D^{20}$: –1.18°.

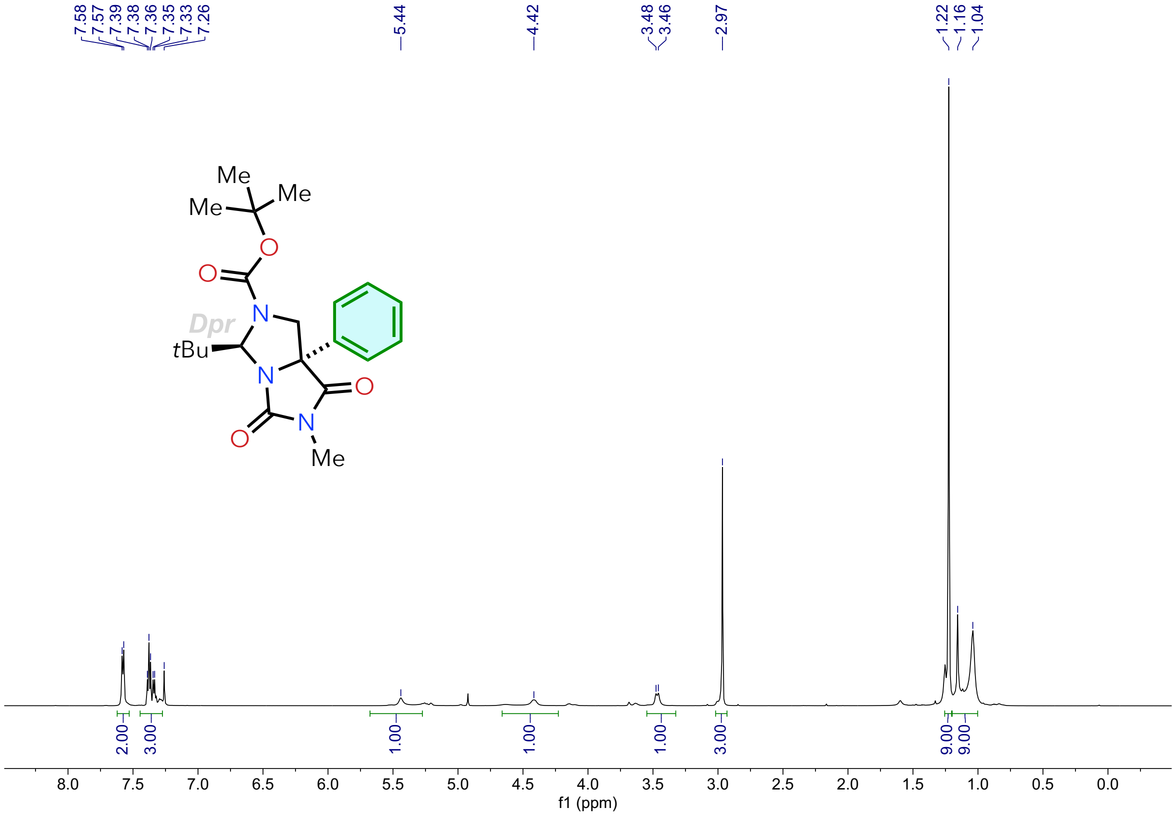
7.58 7.57 7.39 7.38 7.36 7.35 7.33 7.26
5.44
4.42
3.48 3.46
2.97
1.22 1.16 1.04
Me
Me
Me
O
O
N
Dpr
tBu
N
O
O
N
Me
2.00 3.00 1.00 1.00 1.00 3.00 9.00 9.00
f1 (ppm)

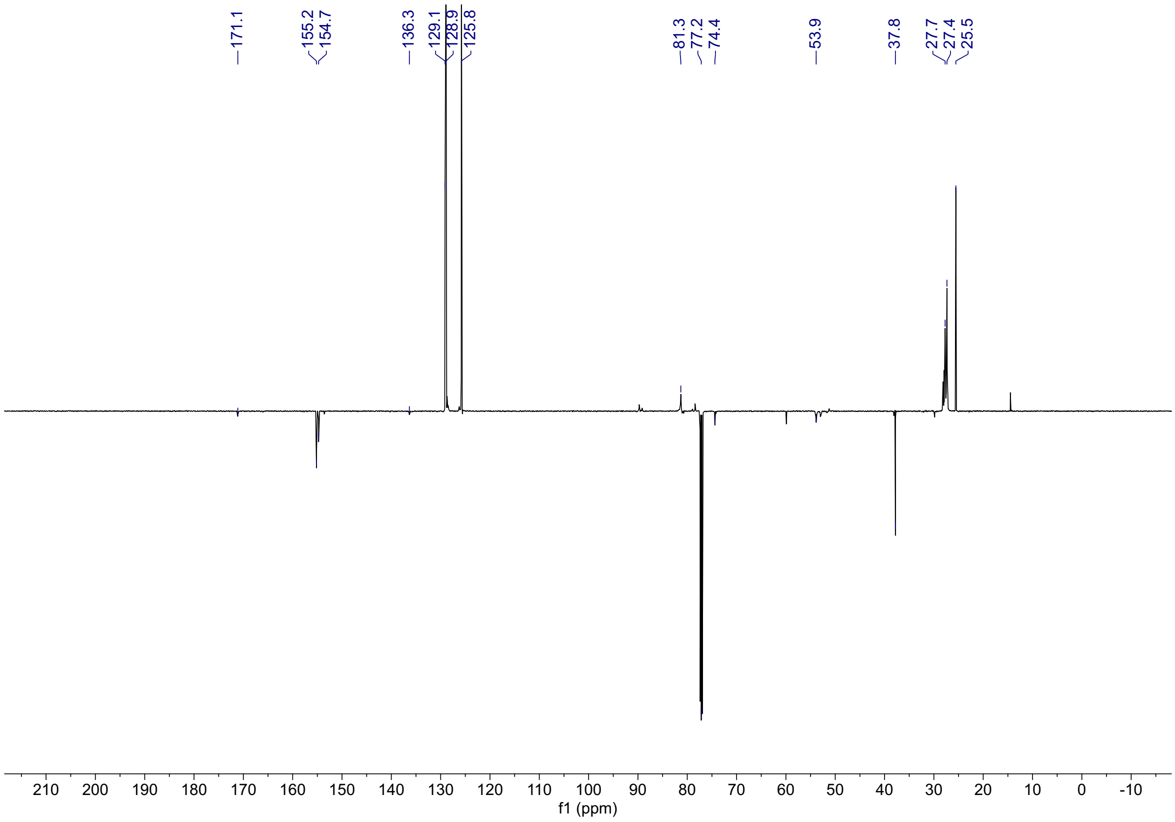
171.1
155.2 154.7
136.3 129.1 128.9 125.8
81.3 77.2 74.4
53.9
37.8
27.7 27.4 25.5
f1 (ppm)

Methyl (*E*)-2-(3-methyl-3-phenylureido)but-2-enoate (25)

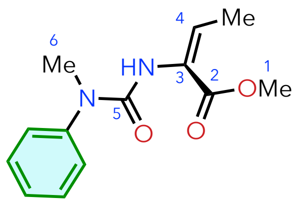

25

Following **GP4**, to a solution of *N*-aryl urea **21a** (100 mg, 0.30 mmol, 1.0 equiv.) in dry THF (3.0 mL, 0.1 M) at –78 °C was added potassium bis(trimethylsilyl)amide solution (0.45 mL, 0.45 mmol, 1 M in THF, 1.5 equiv.) dropwise. The title compound **25** was obtained as a white solid (51 mg, 0.21 mmol, 69%) after purification by silica gel column chromatography (gradient elution, PE/EA).

Formula: $C_{13}H_{16}N_2O_3$, **MW:** 248.28 g/mol. **TLC:** R_f = 0.25 (PE/EA 2:1), $KMnO_4$ stain. **^{1}H NMR** (600 MHz, $CDCl_3$): δ [ppm] = 7.45 (t, *J* 7.6 Hz, 2H, Ar), 7.34 (t, *J* 7.4 Hz, 1H, Ar), 7.30 (d, *J* 7.9 Hz, 2H, Ar), 6.73 (q, *J* 7.6 Hz, 1H, H-4), 6.38 (br s, 1H, NH, urea), 3.72 (s, 3H, H-1, OMe, ester), 3.28 (s, 3H, H-6, NMe, urea), 2.02 (d, *J* 7.7 Hz, 3H, H-4, Me). **^{13}C NMR** (150 MHz, $CDCl_3$): δ [ppm] = 165. 6 (s, C-2, ester), 155.3 (s, C-5, urea), 142.9 (s, Ar), 130.3 (d, 2Ar), 127.9 (d, Ar), 127.5 (d, 2Ar), 126.6 (d, C-4), 126.5 (s, C-3), 52.1 (q, C-1, OMe, ester), 37.3 (q, C-6, NMe, urea), 14.3 (q, C-4, Me). **HR-MS:** (ESI) = *m/z* calcd. for: $C_{13}H_{17}N_2O_3$ $[M+H]^+$ 249.1239 u, found: 249.1223 u.

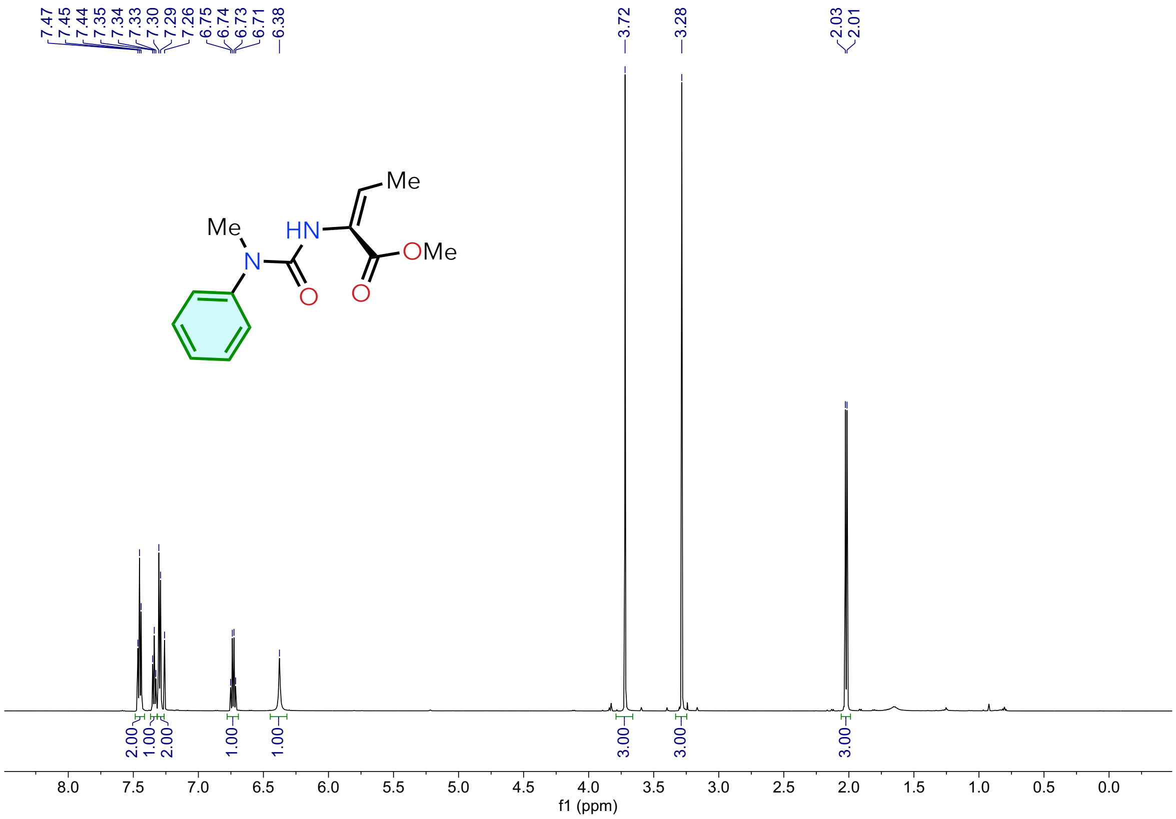
7.47
7.45
7.44
7.35
7.34
7.33
7.30
7.29
7.26
6.75
6.74
6.73
6.71
6.38
3.72
3.28
2.03
2.01
Me
HN
Me
N
O
O
OMe
2.00
1.00
2.00
1.00
1.00
3.00
3.00
3.00
8.0 7.5 7.0 6.5 6.0 5.5 5.0 4.5 4.0 3.5 3.0 2.5 2.0 1.5 1.0 0.5 0.0
f1 (ppm)

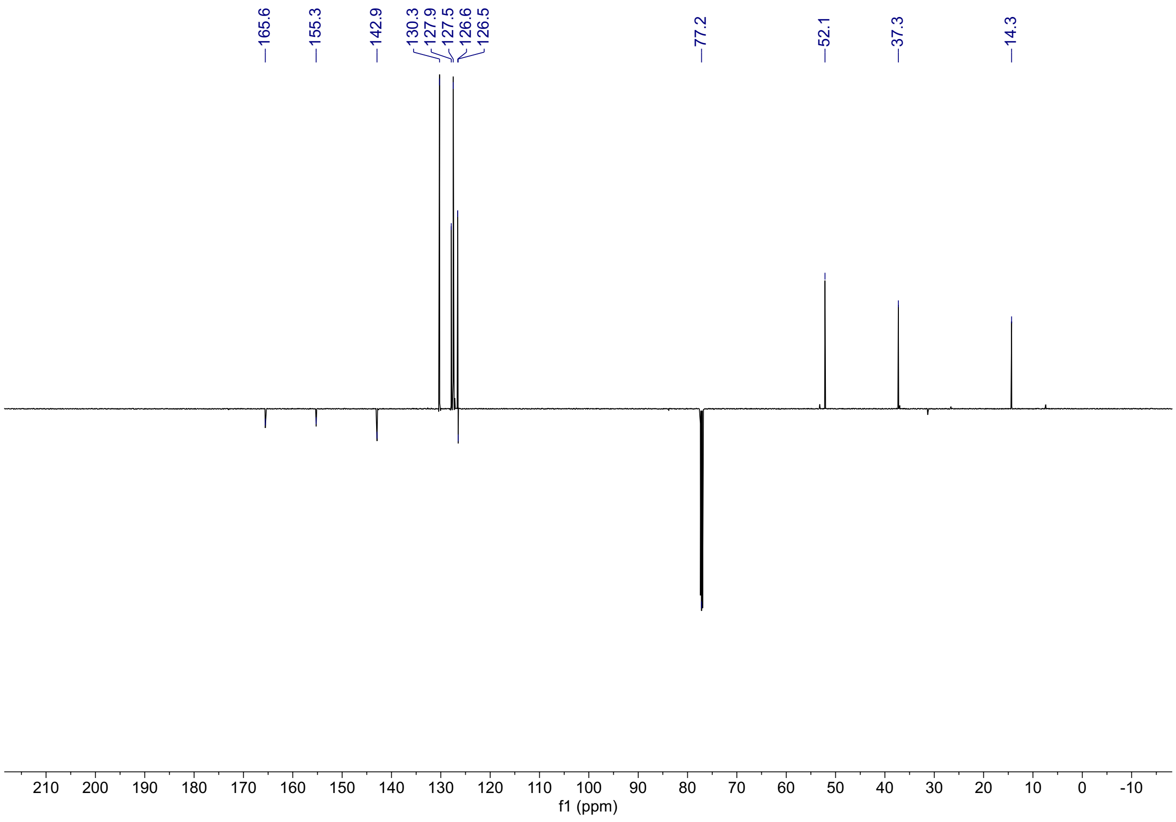
165.6
155.3
142.9
130.3
127.9
127.5
126.6
126.5
77.2
52.1
37.3
14.3
210 200 190 180 170 160 150 140 130 120 110 100 90 80 70 60 50 40 30 20 10 0 -10
f1 (ppm)

(1*S*,3*R*,7a*S*)-3-(*tert*-Butyl)-1,6-dimethyl-7a-phenyldihydro-3*H*,5*H*-imidazo[1,5-*c*]oxazole-5,7(6*H*)-dione (26a)

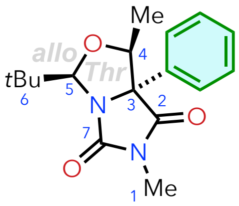

26a

Following **GP4**, to a solution of *N*-aryl urea **22a** (100 mg, 0.30 mmol, 1.0 equiv.) in dry THF (3.0 mL, 0.1 M) at –78 °C was added potassium bis(trimethylsilyl)amide solution (0.45 mL, 0.45 mmol, 1 M in THF, 1.5 equiv.) dropwise. The title compound **26a** was obtained as an incolour gel (51 mg, 0.17 mmol, 56%) after purification by silica gel column chromatography (gradient elution, PE/EA).

Formula: $C_{17}H_{22}N_2O_3$, **MW:** 302.37 g/mol. **TLC:** R_f = 0.60 (PE/EA 2:1), $KMnO_4$ stain. **^{1}H NMR** (600 MHz, $CDCl_3$): δ [ppm] = 7.50 (d, *J* 7.3 Hz, 2H, Ar), 7.42 (t, *J* 7.6 Hz, 2H, Ar), 7.36 (t, *J* 7.3 Hz, 1H, Ar), 4.27 (s, 1H, H-5), 4.25 (q, *J* 6.4 Hz, 1H, H-4), 2.97 (s, 3H, H-1, Me), 1.48 (d, *J* 6.4 Hz, 3H, H-4, Me) 1.26 (s, 9H, *t*Bu). **^{13}C NMR** (150 MHz, $CDCl_3$): δ [ppm] = 171.6 (s, C-2), 157.5 (s, C-7), 137.8 (s, Ar), 129.3 (d, 2Ar), 128.8 (d, Ar), 125.3 (d, 2Ar), 97.6 (d, C-5), 80.9 (t, C-4), 75.9 (s, C-3), 33.3 (s, *t*Bu), 25.5 (q, C-6, *t*Bu), 18.4 (q, C-1, Me). **FT-IR (ATR):** $\tilde{\nu}$ [cm^{-1}] = 2980 (br w), 1782 (w), 1716 (vs), 1441 (m), 1384 (m), 1279 (m), 1202 (w), 1147 (m), 1094 (m), 1049 (w), 1008 (m), 965 (w), 760 (m), 750 (m), 697 (m), 638 (w). **HR-MS:** (ESI) = *m*/*z* calcd. for: $C_{17}H_{23}N_2O_3$ [M+H]$^+$ 303.1709 u, found: 303.1693 u. **$[a]_\lambda^T$:** (c = 1.00 g/100 mL, CH_3Cl_3) = $[a]_D^{20}$: –10.00°.

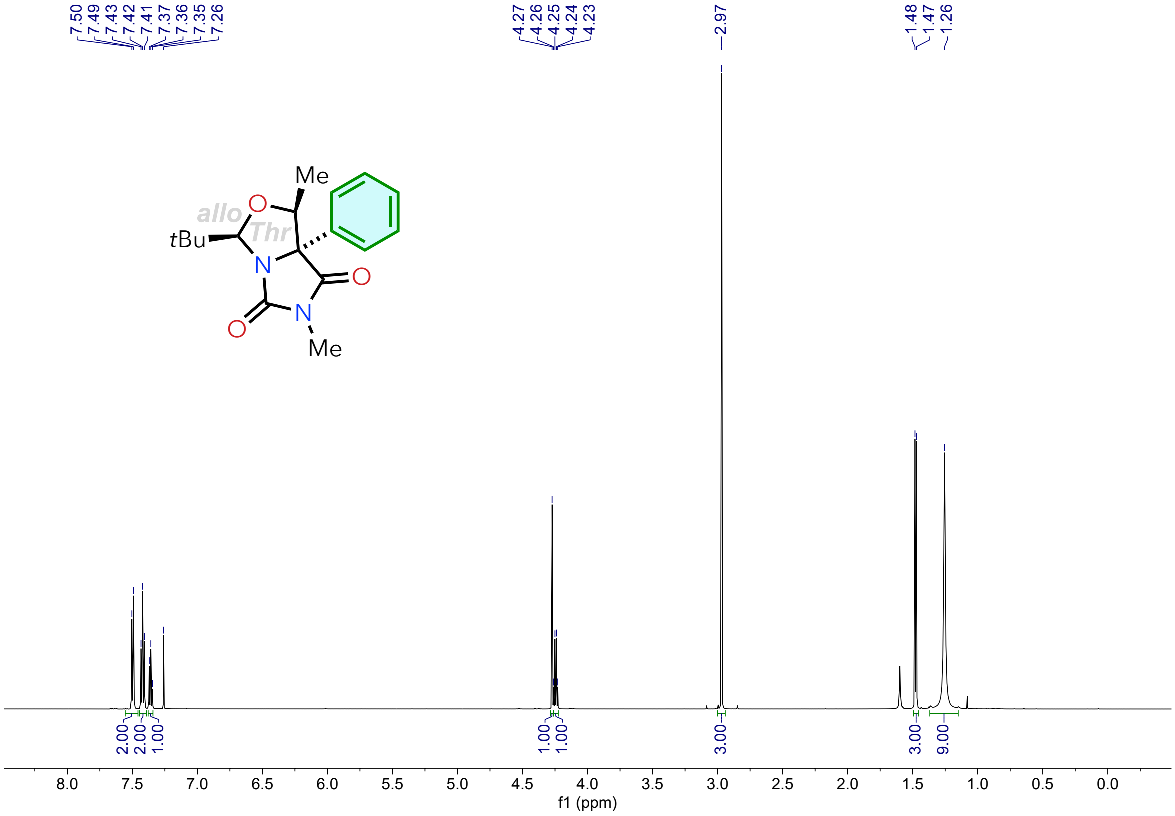
7.50 7.49 7.43 7.42 7.41 7.37 7.36 7.35 7.26
4.27 4.26 4.25 4.24 4.23
2.97
1.48 1.47 1.26
Me
O
allo
Thr
tBu
N
O
N
O
Me
2.00 2.00 1.00
1.00 1.00
3.00
3.00 9.00
8.0 7.5 7.0 6.5 6.0 5.5 5.0 4.5 4.0 3.5 3.0 2.5 2.0 1.5 1.0 0.5 0.0
f1 (ppm)

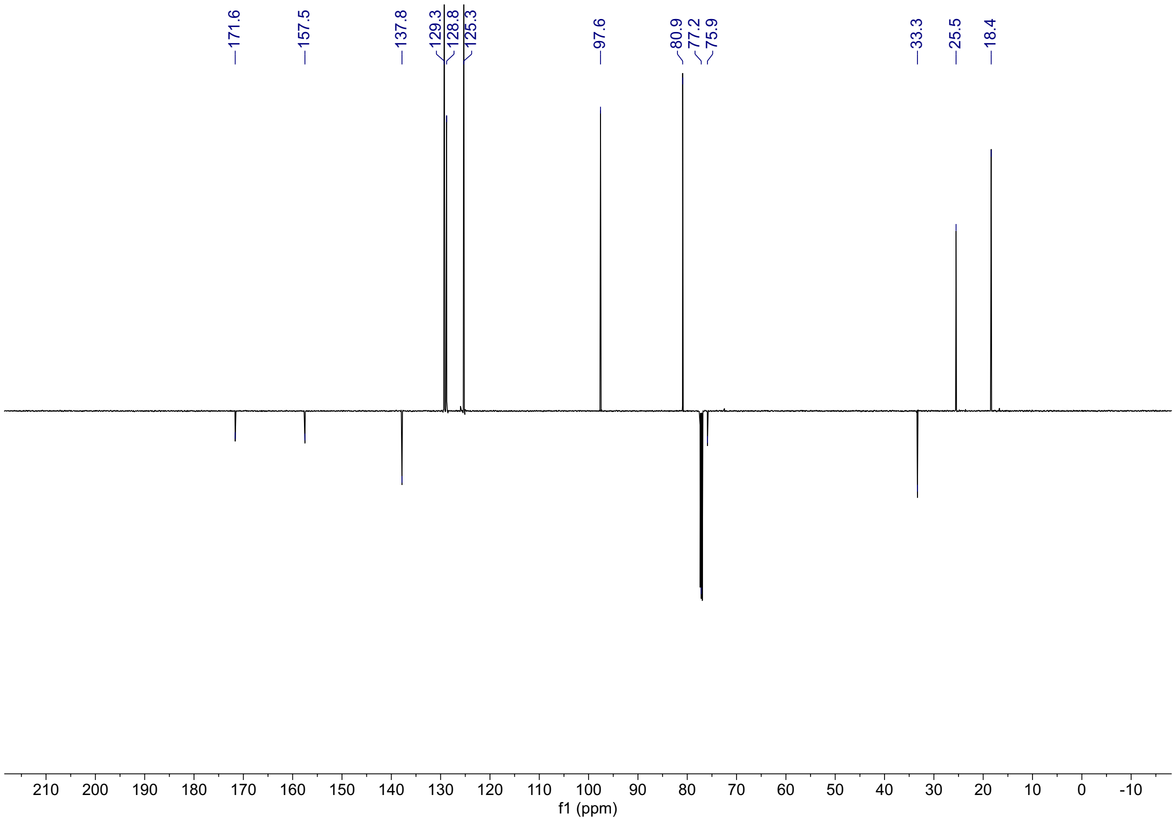
171.6
157.5
137.8 129.3 128.8 125.3
97.6
80.9 77.2 75.9
33.3 25.5 18.4
210 200 190 180 170 160 150 140 130 120 110 100 90 80 70 60 50 40 30 20 10 0 -10
f1 (ppm)

(1*S*,3*R*,7a*S*)-7a-(4-Bromophenyl)-3-(*tert*-butyl)-1,6-dimethyldihydro-3*H*,5*H*-imidazo[1,5-*c*]oxazole-5,7(6*H*)-dione (26b)

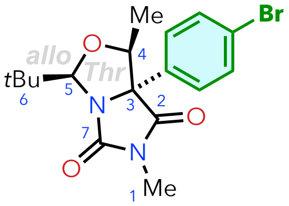

26b

Following **GP4**, to a solution of *N*-aryl urea **22b** (100 mg, 0.24 mmol, 1.0 equiv.) in dry THF (2.4 mL, 0.1 M) at –78 °C was added potassium bis(trimethylsilyl)amide solution (0.36 mL, 0.36 mmol, 1 M in THF, 1.5 equiv.) dropwise. The title compound **26b** was obtained as a sticky colourless oil (72 mg, 0.19 mmol, 78%) after purification by silica gel column chromatography (gradient elution, PE/EA).

Formula: $C_{17}H_{21}BrN_2O_3$, **MW:** 381.27 g/mol. **TLC:** R_f = 0.67 (PE/EA 2:1), $KMnO_4$ stain. **^{1}H NMR** (500 MHz, $CDCl_3$): δ [ppm] = 7.55 (d, *J* 8.5 Hz, 2H, Ar), 7.37 (d, *J* 8.5 Hz, 2H, Ar), 4.22 (s, 1H, H-5), 4.17 (q, *J* 6.4 Hz, 1H, H-4), 2.97 (s, 3H, H-1, Me), 1.46 (d, *J* 6.4 Hz, 3H, H-4, Me) 1.25 (s, 9H, *t*Bu). **^{13}C NMR** (125 MHz, $CDCl_3$): δ [ppm] = 171.2 (s, C-2), 157.5 (s, C-7), 136.9 (s, Ar), 132.4 (d, 2Ar), 127.1 (d, 2Ar), 123.1 (s, Ar), 97.8 (d, C-5), 80.9 (t, C-4), 75.6 (s, C-3), 33.4 (s, *t*Bu), 25.6 (q, C-6, *t*Bu), 18.3 (q, C-1, Me). **FT-IR (ATR):** $\tilde{\nu}$ [cm^{-1}] = 2980 (br m), 1783 (w), 1717 (vs), 1486 (w), 1440 (m), 1385 (m), 1340 (w), 11278 (m), 1204 (w), 1147 (m), 1091 (m), 1049 (w), 1009 (m), 965 (w), 896 (w), 871 (w), 806 (m), 778 (w), 705 (w), 573 (w), 511 (w). **HR-MS:** (ESI) = *m*/*z* calcd. for: $C_{16}H_{18}{}^{79}BrN_2O_3$ [M–Me]$^+$ 365.0501 u, found: 365.0490 u. **$[a]_{\lambda}^{T}$:** (c = 1.00 g/100 mL, $CHCl_3$) = $[a]_D^{20}$: –16.40°.

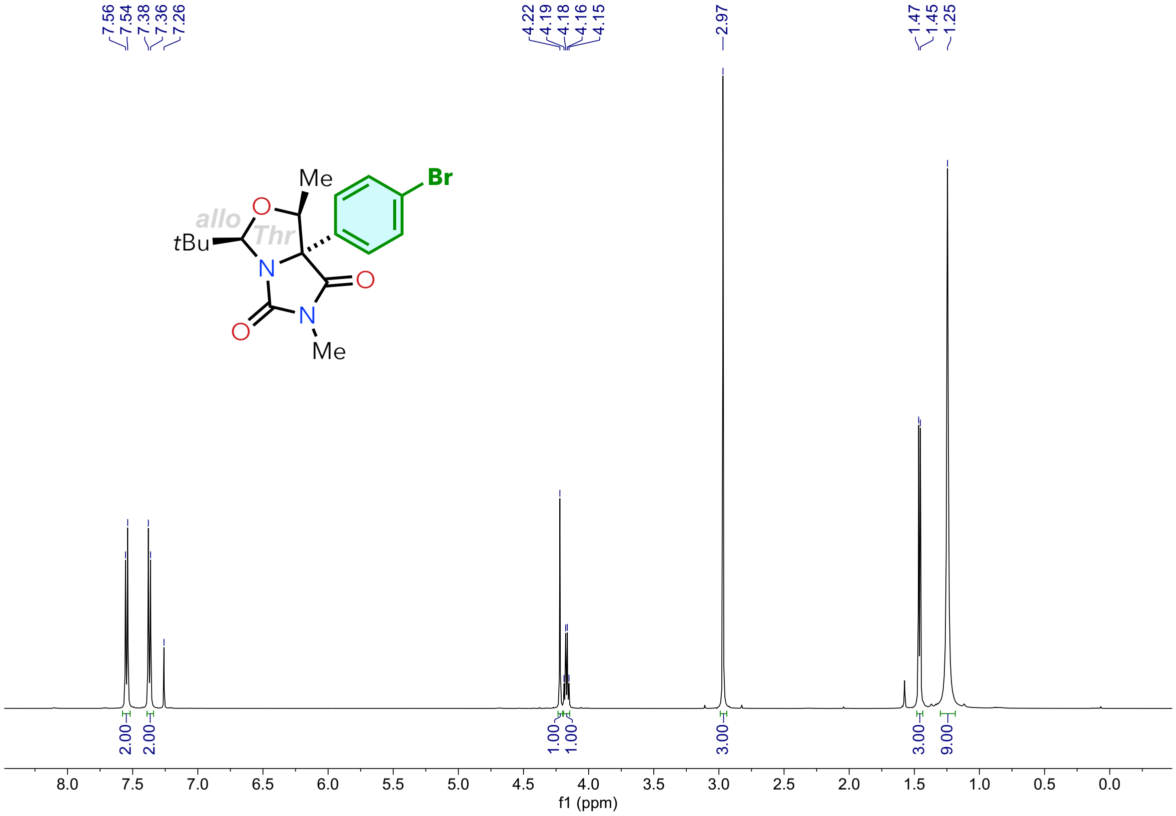
7.56
7.54
7.38
7.36
7.26
4.22
4.19
4.18
4.16
4.15
2.97
1.47
1.45
1.25
Me
Br
O
allo
Thr
tBu
N
O
N
O
Me
2.00
2.00
1.00
1.00
3.00
3.00
9.00
8.0 7.5 7.0 6.5 6.0 5.5 5.0 4.5 4.0 3.5 3.0 2.5 2.0 1.5 1.0 0.5 0.0
f1 (ppm)

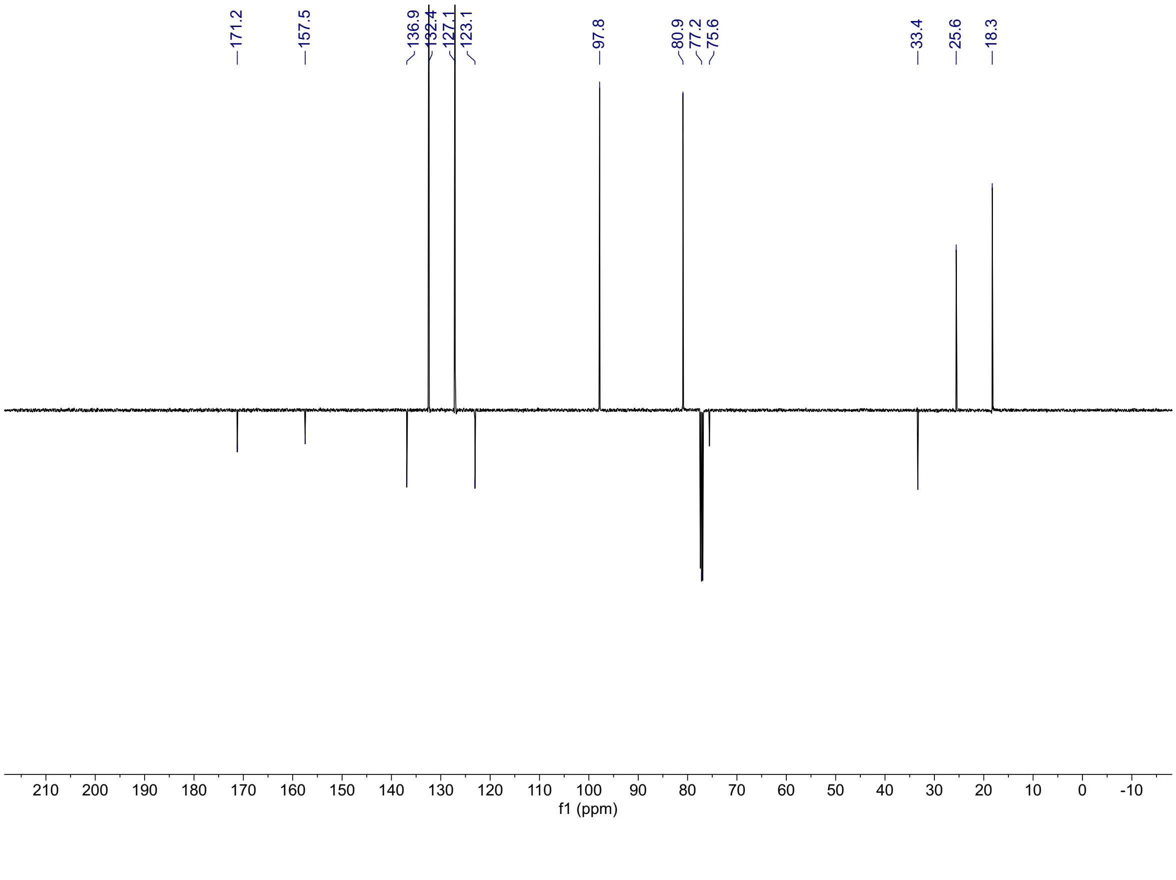
171.2
157.5
136.9
132.4
127.1
123.1
97.8
80.9
77.2
75.6
33.4
25.6
18.3
210 200 190 180 170 160 150 140 130 120 110 100 90 80 70 60 50 40 30 20 10 0 -10
f1 (ppm)

(1*S*,3*R*,7a*S*)-3-(*tert*-Butyl)-1,6-dimethyl-7a-(4-(trifluoromethyl)phenyl)dihydro-3*H*,5*H*-imidazo[1,5-*c*]oxazole-5,7(6*H*)-dione (26c)

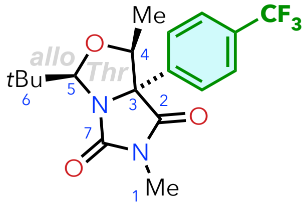

26c

Following **GP4**, to a solution of *N*-aryl urea **22c** (100 mg, 0.25 mmol, 1.0 equiv.) in dry THF (2.5 mL, 0.1 M) at –78 °C was added potassium bis(trimethylsilyl)amide solution (0.37 mL, 0.37 mmol, 1 M in THF, 1.5 equiv.) dropwise. The title compound **26c** was obtained as a white solid (66 mg, 0.18 mmol, 72%) after purification by silica gel column chromatography (gradient elution, PE/EA).

Formula: $C_{18}H_{21}F_3N_2O_3$, **MW:** 370.37 g/mol, **m.p.:** 106 – 109 °C. **TLC:** R_f = 0.81 (PE/EA 2:1), $KMnO_4$ stain. **^{1}H NMR** (600 MHz, $CDCl_3$): δ [ppm] = 7.71 (d, *J* 8.4 Hz, 2H, Ar), 7.66 (d, *J* 8.4 Hz, 2H, Ar), 4.26 (s, 1H, H-5), 4.21 (q, *J* 6.4 Hz, 1H, H-4), 3.01 (s, 3H, H-1, Me), 1.52 (d, *J* 6.4 Hz, 3H, H-4, Me) 1.28 (s, 9H, *t*Bu). **^{13}C NMR** (150 MHz, $CDCl_3$): δ [ppm] = 171.0 (s, C-2, amide), 157.5 (s, C-7, urea), 141.8 (s, Ar), 131.1 (s, *q J* 32.7 Hz, Ar), 126.3 (d, *q J* 3.7 Hz, 2Ar), 125.9 (d, 2Ar), 124.0 (s, *q J* 272.3 Hz, Ar), 98.0 (d, C-5), 81.0 (d, C-4), 75.8 (s, C-3), 33.4 (s, *t*Bu), 25.6 (q, C-6, *t*Bu), 18.3 (q, C-1, Me). **^{19}F NMR** (565 MHz, $CDCl_3$, C_6F_6 ref.): δ [ppm] = –66.00 (s, 3F). **FT-IR (ATR):** $\tilde{\nu}$ [cm^{-1}] = 2980 (br m), 1784 (w), 1721 (vs), 1436 (w), 1383 (m), 1325 (s), 1280 (m), 1159 (m), 1132 (s), 1116 (s), 1087 (m), 1069 (vs), 1016 (m), 948 (m), 896 (w), 844 (w), 816 (m), 760 (w), 704 (w), 601 (w), 571 (w). **HR-MS:** (ESI) = *m/z* calcd. for: $C_{18}H_{20}F_3N_2O_3$ [M–Me]$^+$ 369.1426 u, found: 369.1411 u. **$[a]_\lambda^T$:** (c = 1.00 g/100 mL, $CHCl_3$) = $[a]_D^{20}$: –21.20°.

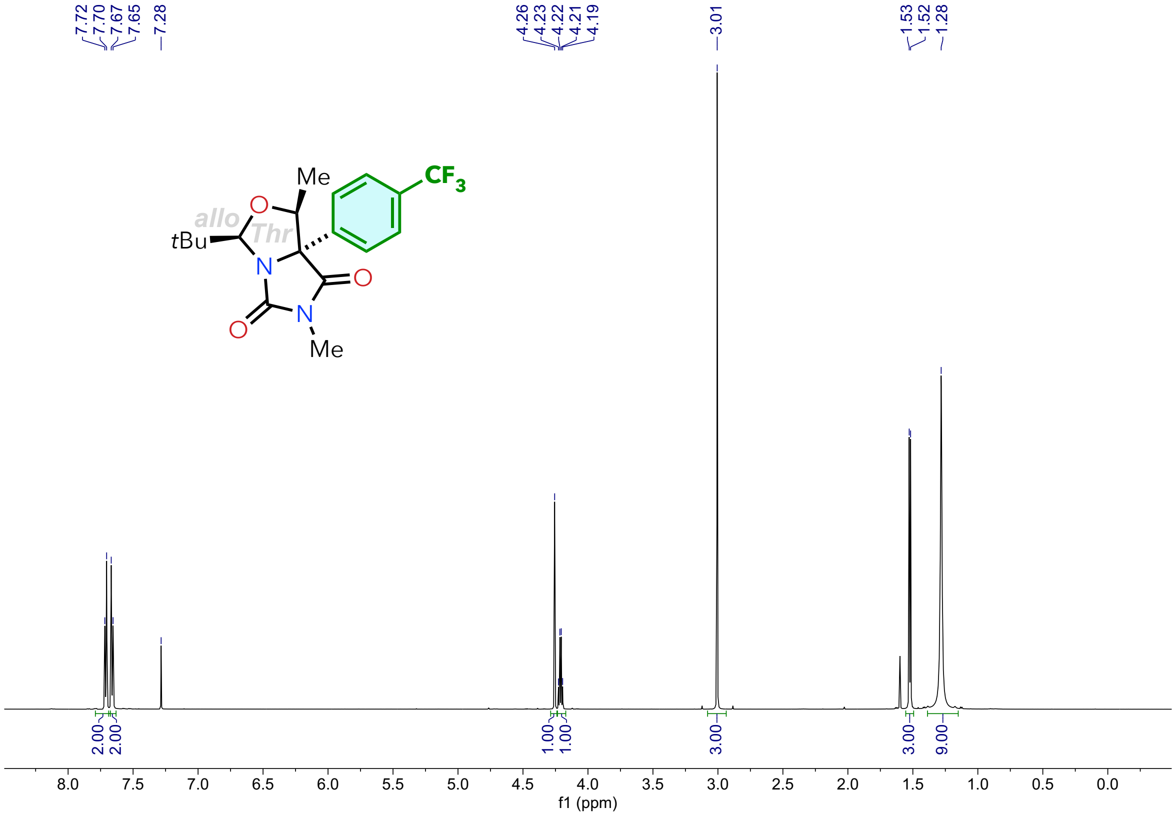
7.72
7.70
7.67
7.65
7.28
4.26
4.23
4.22
4.21
4.19
3.01
1.53
1.52
1.28
Me
CF3
O
allo
Thr
tBu
N
O
O
N
Me
2.00
2.00
1.00
1.00
3.00
3.00
9.00
8.0 7.5 7.0 6.5 6.0 5.5 5.0 4.5 4.0 3.5 3.0 2.5 2.0 1.5 1.0 0.5 0.0
f1 (ppm)

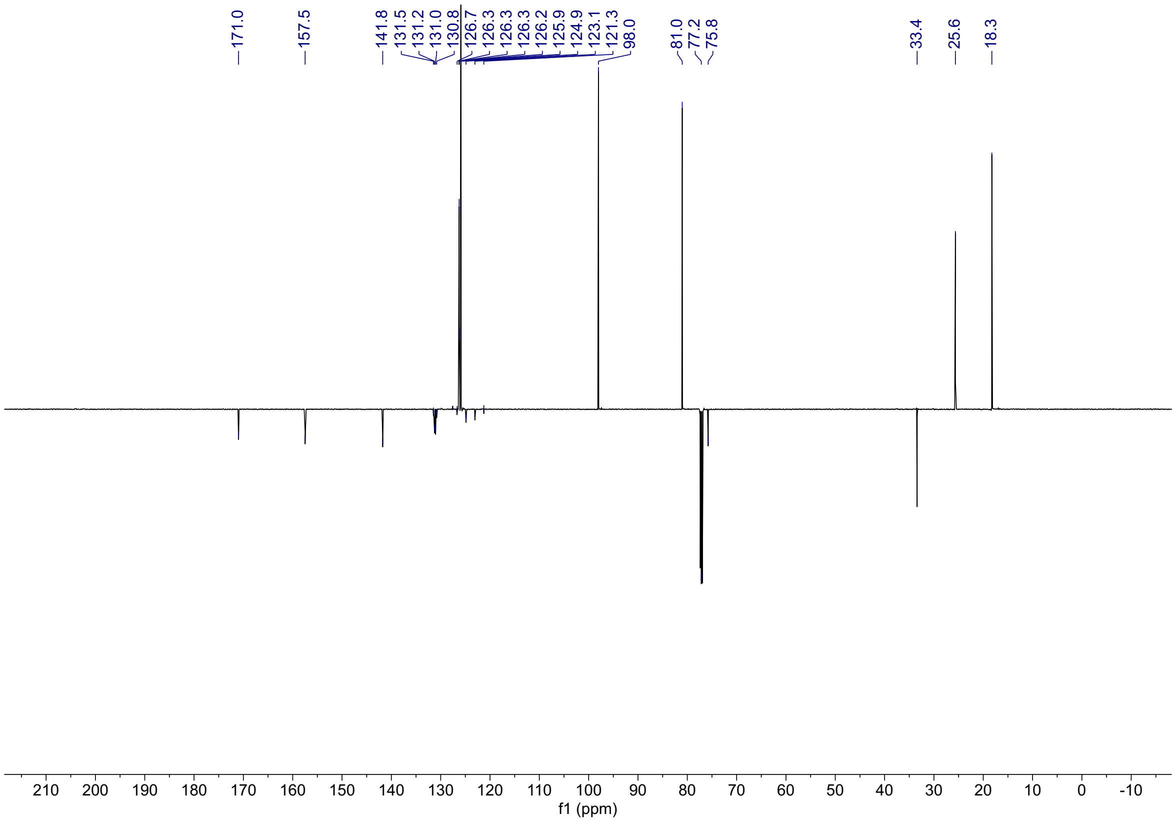
171.0
157.5
141.8
131.5
131.2
131.0
130.8
126.7
126.3
126.3
126.3
126.2
125.9
124.9
123.1
121.3
98.0
81.0
77.2
75.8
33.4
25.6
18.3
210 200 190 180 170 160 150 140 130 120 110 100 90 80 70 60 50 40 30 20 10 0 -10
f1 (ppm)

(1*S*,3*R*,7a*S*)-3-(*tert*-Butyl)-7a-(3-methoxyphenyl)-1,6-dimethyldihydro-3*H*,5*H*-imidazo[1,5-*c*]oxazole-5,7(6*H*)-dione (26d)

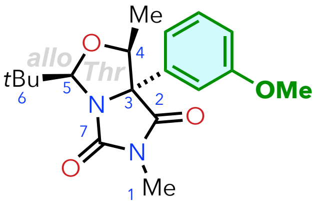

26d

Following **GP4**, to a solution of *N*-aryl urea **22d** (100 mg, 0.27 mmol, 1.0 equiv.) in dry THF (2.7 mL, 0.1 M) at –78 °C was added potassium bis(trimethylsilyl)amide solution (0.41 mL, 0.41 mmol, 1 M in THF, 1.5 equiv.) dropwise. The title compound **26d** was obtained as a colourless oil (64 mg, 0.19 mmol, 70%) after purification by silica gel column chromatography (gradient elution, PE/EA).

Formula: $C_{18}H_{24}N_2O_4$, **MW:** 332.40 g/mol. **TLC:** R_f = 0.58 (PE/EA 2:1), $KMnO_4$ stain. **^{1}H NMR** (500 MHz, $CDCl_3$): δ [ppm] = 7.34 (d, *J* 8.0 Hz, 1H, Ar), 7.08 (ddd, *J* 7.8, 1.6, 0.8 Hz, 1H, Ar), 7.05 – 7.03 (m, 1H, Ar), 6.92 – 6.87 (m, 1H, Ar), 4.27 (s, 1H, H-5), 4.23 (q, *J* 6.4 Hz, 1H, H-4), 3.82 (s, 3H, Ar, OMe), 2.97 (s, 3H, H-1, Me), 1.47 (d, *J* 6.4 Hz, 3H, H-4, Me) 1.25 (s, 9H, *t*Bu). **^{13}C NMR** (125 MHz, $CDCl_3$): δ [ppm] = 171.5 (s, C-2), 160.3 (s, Ar), 157.5 (s, C-7), 139.4 (s, Ar), 130.4 (d, 1Ar), 117.6 (d, Ar), 113.7 (d, Ar), 111.4 (d, Ar), 97.6 (d, C-5), 80.9 (t, C-4), 75.8 (s, C-3), 55.5 (q, Ar, OMe), 33.3 (s, *t*Bu), 25.5 (q, C-6, *t*Bu), 18.3 (q, C-1, Me). **FT-IR (ATR):** $\tilde{\nu}$ [cm^{-1}] = 2980 (br w), 1781 (w), 1715 (vs), 1600 (w), 1584 (w), 1436 (m), 1385 (m), 1279 (m), 1263 (m), 1200 (m), 1146 (m), 1085 (w), 1047 (m), 1009 (m), 963 (w), 808 (w), 776 (w), 723 (w), 692 (m). **HR-MS:** (ESI) = *m*/*z* calcd. for: $C_{18}H_{25}N_2O_4$ $[M+H]^+$ 333.1814 u, found: 333.1802 u. **$[a]_\lambda^T$:** (c = 1.00 g/100 mL, $CHCl_3$) = $[a]_D^{20}$: –13.60°.

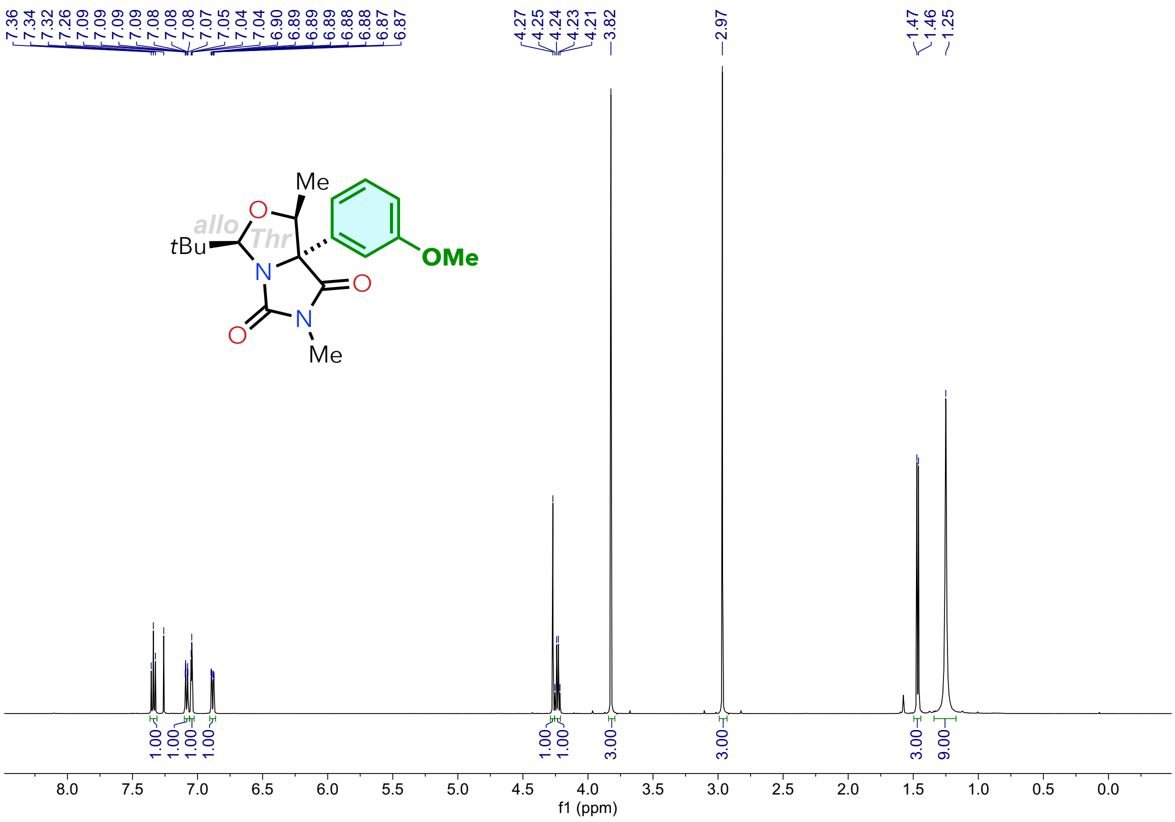
7.36
7.34
7.32
7.26
7.09
7.09
7.09
7.09
7.08
7.08
7.08
7.07
7.05
7.04
7.04
6.90
6.89
6.89
6.89
6.88
6.88
6.87
6.87
4.27
4.25
4.24
4.24
4.23
4.21
3.82
2.97
1.47
1.46
1.25
Me
O
allo
Thr
tBu
N
O
N
O
Me
OMe
1.00
1.00
1.00
1.00
1.00
1.00
3.00
3.00
3.00
9.00
8.0 7.5 7.0 6.5 6.0 5.5 5.0 4.5 4.0 3.5 3.0 2.5 2.0 1.5 1.0 0.5 0.0
f1 (ppm)

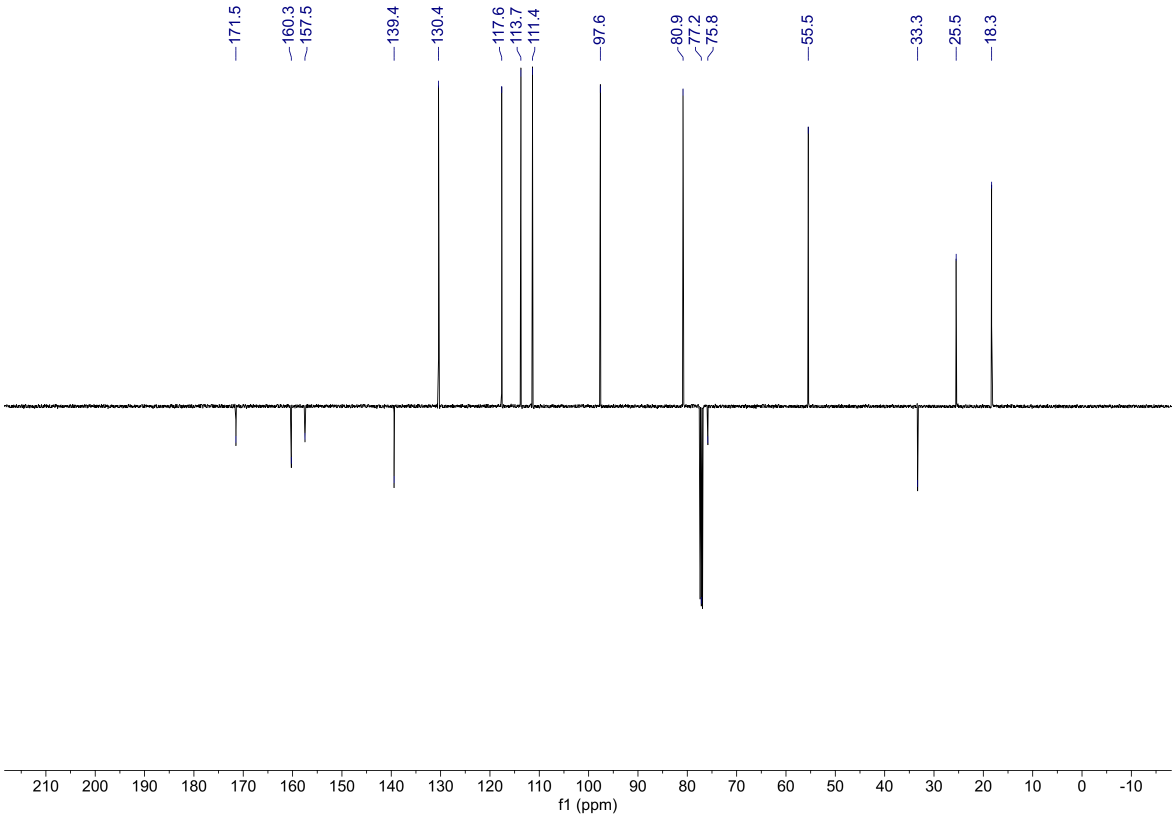
171.5
160.3
157.5
139.4
130.4
117.6
113.7
111.4
97.6
80.9
77.2
75.8
55.5
33.3
25.5
18.3
210 200 190 180 170 160 150 140 130 120 110 100 90 80 70 60 50 40 30 20 10 0 -10
f1 (ppm)

4.5 Acidic Hydrolysis according to General Procedure 5 (GP5)

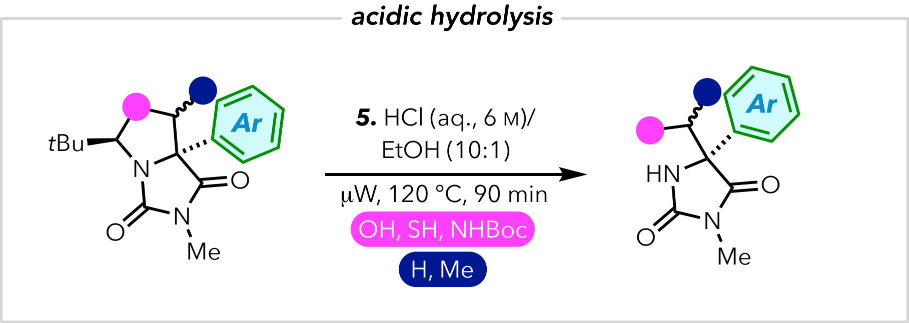

(*S*)-5-(Hydroxymethyl)-3-methyl-5-phenylimidazolidine-2,4-dione (27a)

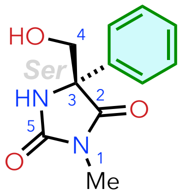

27a

Following **GP5**, a solution of rearranged urea **5a** (330 mg, 1.14 mmol, 1.0 equiv.) was suspended in a HCl/EtOH-solution mixture (11 mL, 6 M, aq.; 10:1). The reaction mixture was placed in a microwave reactor for 1.5 h at 120 °C. The title hydantoin **27a** was obtained as a white solid (214 mg, 0.97 mmol, 85%) after purification by silica gel column chromatography (PE/EA, gradient elution).

Formula: $C_{11}H_{12}N_2O_3$, **MW:** 220.23 g/mol, **m.p.:** 194 – 197 °C. **TLC:** R_f = 0.27 (PE/EA 1:2), $KMnO_4$ stain. **^{1}H NMR** (500 MHz, CD_3OD): δ [ppm] = 7.60 – 7.53 (m, 2H, Ar), 7.36 (dt, *J* 24.8, 7.2 Hz, 3H, Ar), 4.17 (d, *J* 11.3 Hz, 1H, H-4a), 3.69 (d, *J* 11.3 Hz, 1H, H-4b), 2.96 (s, 3H, H-1, Me). **^{13}C NMR** (125 MHz, CD_3OD): δ [ppm] = 176.0 (s, C-2), 159.4 (s, C-5, urea), 137.0 (s, Ar), 129.7 (d, 2Ar), 129.5 (d, Ar), 126.8 (d, Ar), 70.6 (t, C-4), 67.5 (s, C-3, *alpha*-carbon), 24.7 (q, C-1, Me). **FT-IR (ATR):** $\tilde{\nu}$ [cm^{-1}] = 3427 (br w), 3300 (br w), 1770 (w), 1695 (vs); 1465 (m), 1447 (m), 1390 (w), 1324 (w), 1313 (w), 1068 (m), 1029 (w), 901 (w), 777 (w), 732 (m), 718 (m), 703 (m), 654 (w), 626 (m), 614 (w), 571 (s), 506 (s). **HR-MS:** (ESI) = *m/z* calcd. for: $C_{11}H_{12}N_2O_3$ $[M+H]^+$ 221.0926 u, found: 221.0924 u. **$[a]_\lambda^T$:** (c = 1.00 g/100 mL, MeOH) = $[a]_D^{20}$: –10.80°.

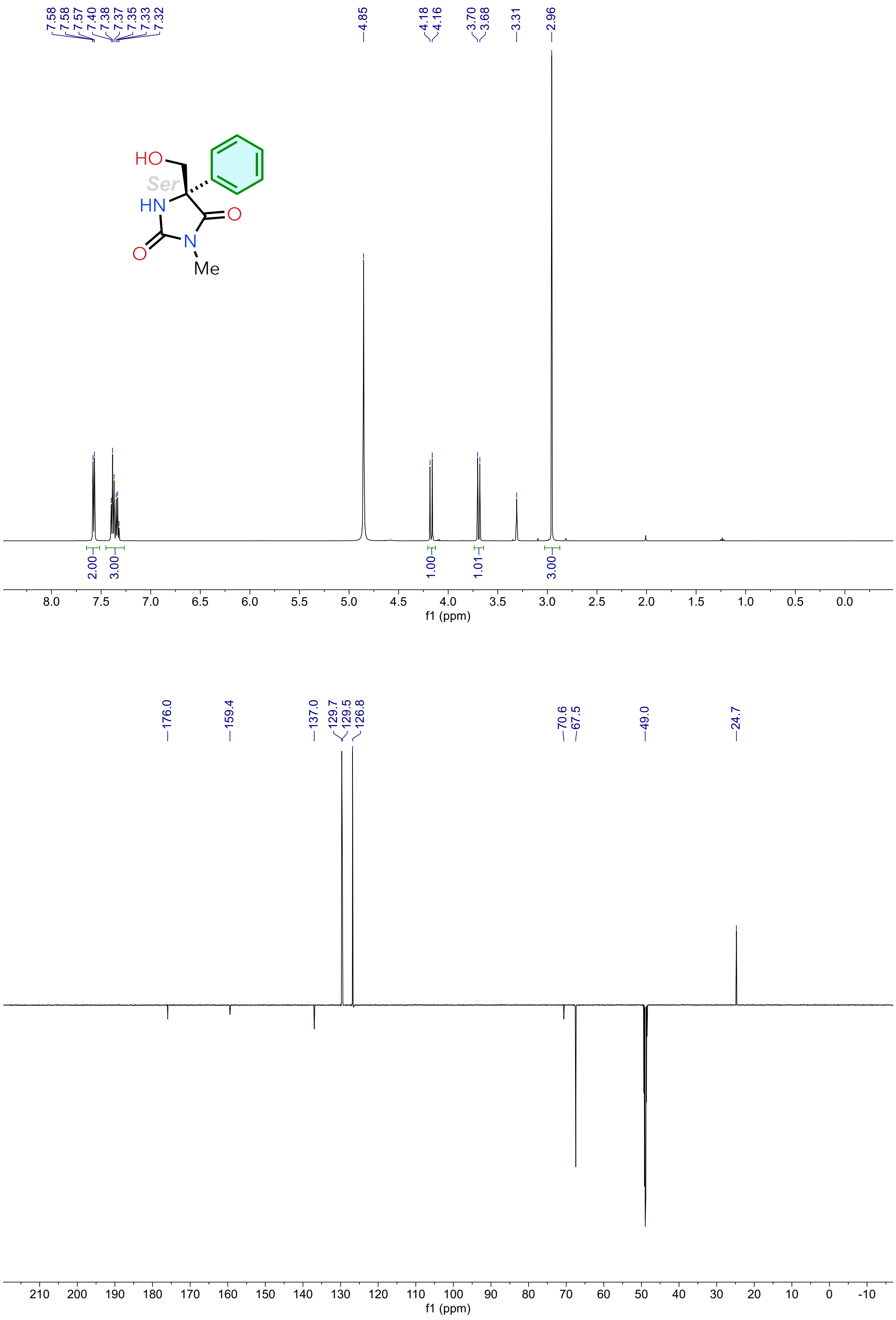

7.58
7.58
7.57
7.40
7.38
7.37
7.35
7.33
7.32
4.85
4.18
4.16
3.70
3.68
3.31
2.96
HO
Ser
HN
O
N
O
Me
2.00
3.00
1.00
1.01
3.00
f1 (ppm)
176.0
159.4
137.0
129.7
129.5
126.8
70.6
67.5
49.0
24.7
f1 (ppm)

(*S*)-5-(4-Chlorophenyl)-5-(hydroxymethyl)-3-methylimidazolidine-2,4-dione (27b)

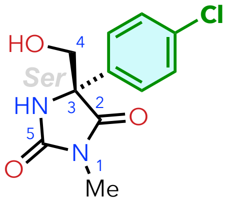

27b

Following **GP5**, a solution of rearranged urea **5c** (200 mg, 0.56 mmol, 1.0 equiv.) was suspended in a HCl/EtOH-solution mixture (11 mL, 6 M, aq.; 10:1). The title compound **27b** was obtained as a white solid (119 mg, 0.47 mmol, 83%) after purification by silica gel column chromatography (PE/EA, gradient elution).

Formula: $C_{11}H_{11}ClN_2O_3$, **MW:** 254.67 g/mol, **m.p.:** 183 – 186 °C. **TLC:** R_f = 0.36 (PE/EA 1:2), $KMnO_4$ stain. **^{1}H NMR** (500 MHz, CD_3OD): δ [ppm] = 7.58 (d, *J* 8.7 Hz, 2H, Ar), 7.40 (d, *J* 8.7 Hz, 2H, Ar), 4.13 (d, *J* 11.3 Hz, 1H, H-4a), 3.67 (d, *J* 11.3 Hz, 1H, H-4b), 2.96 (s, 3H, H-1, Me). **^{13}C NMR** (125 MHz, CD_3OD): δ [ppm] = 175.6 (s, C-2), 159.3 (s, C-5, urea), 135.9 (s, Ar), 135.6 (s, Ar), 129.7 (d, 2Ar), 128.6 (d, 2Ar), 126.8 (d, Ar), 70.2 (t, C-4), 67.5 (s, C-3, *alpha*-carbon), 24.8 (q, C-1, Me). **FT-IR (ATR):** $\tilde{\nu}$ [cm^{-1}] = 3504 (br w), 3257 (br w), 1759 (m), 1701 (vs), 1461 (m), 1396 (m), 1321 (w), 1252 (w), 1205 (w), 1186 (w), 1155 (w), 1078 (m), 1033 (m), 1014 (m), 979 (w), 947 (w), 897 (m), 825 (m), 728 (m), 715 (m), 692 (m), 560 (m), 511 (s), 483 (m). **HR-MS:** (ESI) = *m/z* calcd. for: $C_{11}H_{12}{}^{35}ClN_2O_3$ $[M+H]^+$ 255.0536 u, found: 255.0541 u. **$[a]_\lambda^T$:** (c = 1.04 g/100 mL, MeOH) = $[a]_D^{20}$: –10.38°.

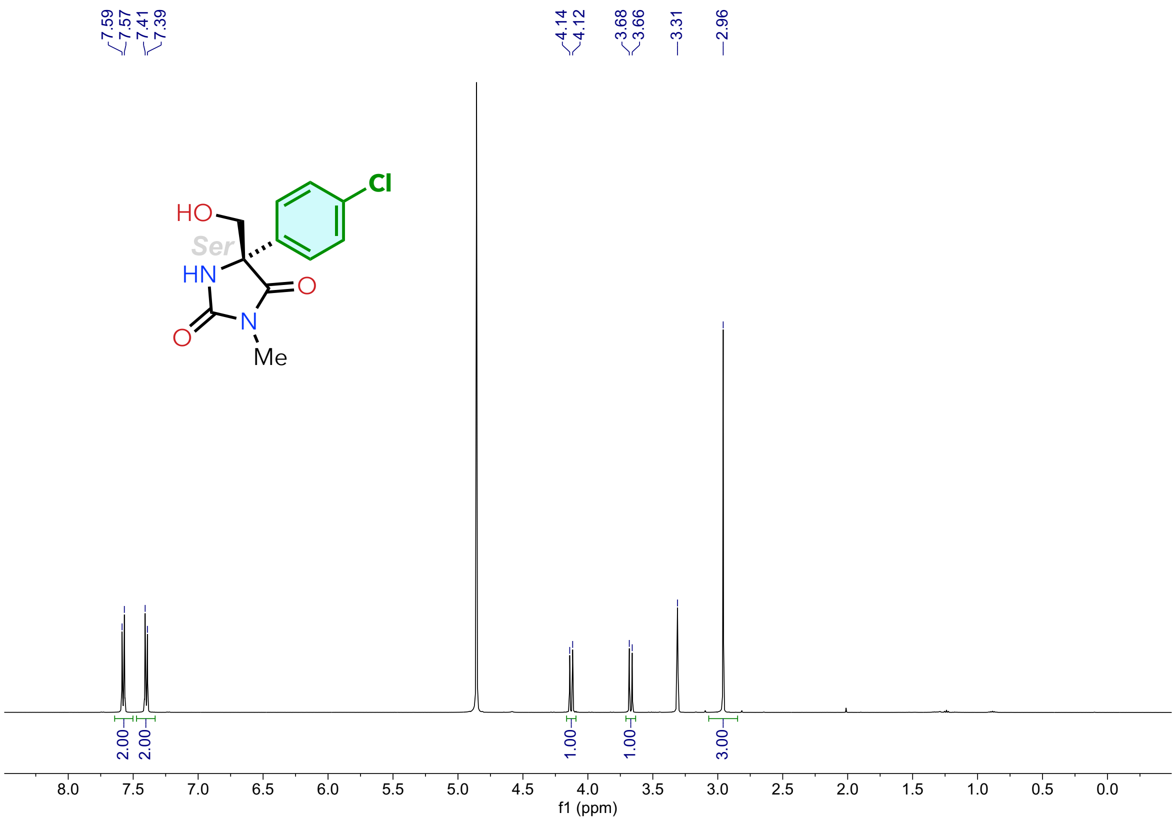
7.59
7.57
7.41
7.39
4.14
4.12
3.68
3.66
3.31
2.96
HO
Ser
HN
O
N
Me
Cl
2.00
2.00
1.00
1.00
3.00
8.0 7.5 7.0 6.5 6.0 5.5 5.0 4.5 4.0 3.5 3.0 2.5 2.0 1.5 1.0 0.5 0.0
f1 (ppm)

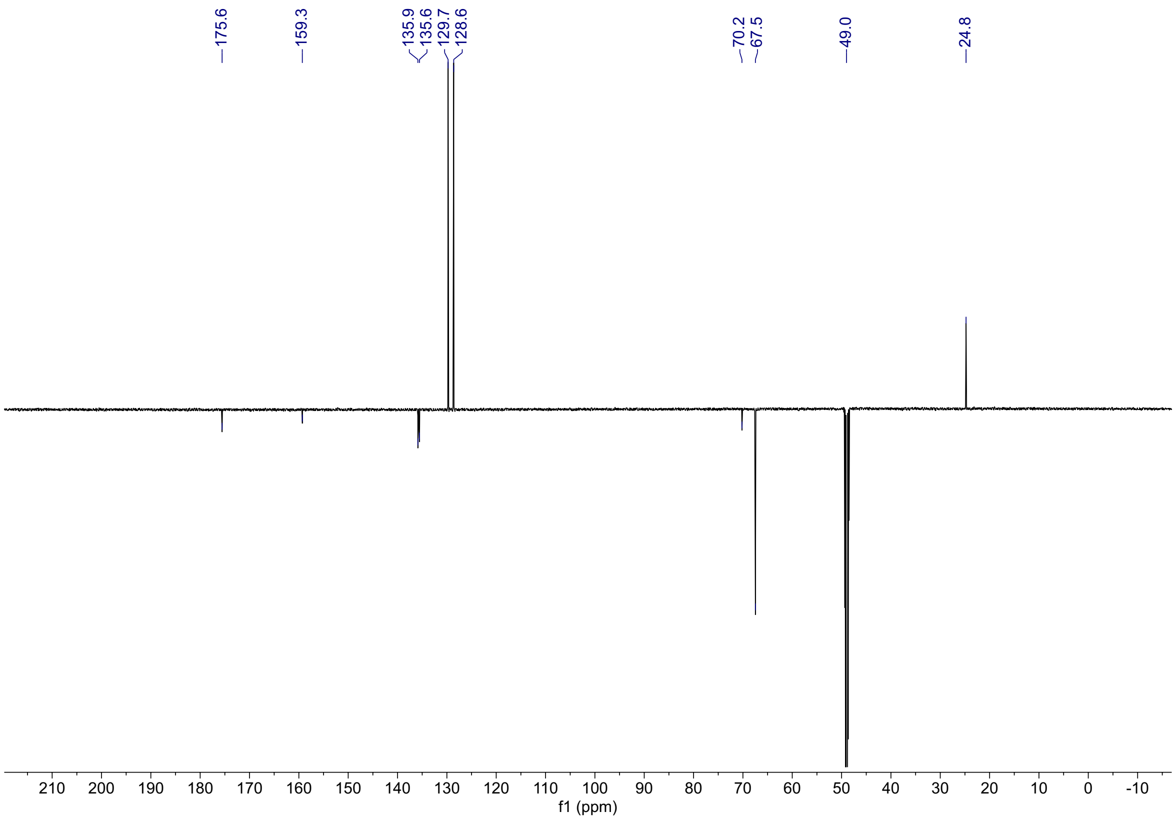
175.6
159.3
135.9
135.6
129.7
128.6
70.2
67.5
49.0
24.8
210 200 190 180 170 160 150 140 130 120 110 100 90 80 70 60 50 40 30 20 10 0 -10
f1 (ppm)

(*S*)-5-(hydroxymethyl)-3-methyl-5-(4-(trifluoromethyl)phenyl)imidazolidine-2,4-dione (27c)

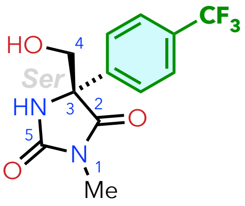

27c

Following **GP5**, a solution of rearranged urea **5f** (447 mg, 1.25 mmol, 1.0 equiv.) was suspended in a HCl/EtOH-solution mixture (11 mL, 6 M, aq.; 10:1). The title compound **27c** was obtained as a white solid (322 mg, 1.12 mmol, 89%) after purification by silica gel column chromatography (PE/EA, gradient elution).

Formula: $C_{12}H_{11}F_3N_2O_3$, **MW:** 288.23 g/mol, **m.p.:** 154 – 157 °C. **TLC:** R_f = 0.25 (PE/EA 1:2), $KMnO_4$ stain. **^{1}H NMR** (600 MHz, CD_3OD): δ [ppm] = 7.81 (d, *J* 8.3 Hz, 2H, Ar), 7.70 (d, *J* 8.4 Hz, 2H, Ar), 4.18 (d, *J* 11.3 Hz, 1H, H-4a), 3.72 (d, *J* 11.3 Hz, 1H, H-4b), 2.97 (s, 3H, H-1, Me). **^{13}C NMR** (150 MHz, CD_3OD): δ [ppm] = 175.2 (s, C-2), 159.3 (s, C-5, urea), 141.4 (s, Ar), 131.6 (s, *q J* 32.4 Hz, Ar), 127.8 (d, 2Ar), 126.5 (d, *q J* 3.8 Hz, 2Ar), 125.5 (s, *q J* 271.2 Hz, Ar), 70.5 (t, C-4), 67.6 (s, C-3, *alpha*-carbon), 24.8 (q, C-1, Me). **^{19}F NMR** (565 MHz, $CDCl_3$, C_6F_6 ref.): δ [ppm] = –63.80 (s, 3F). **FT-IR (ATR):** $\tilde{\nu}$ [cm^{-1}] = 2446 (br w), 1762 (m), 1703 (vs), 1617 (w), 1461 (m), 1414 (w), 1387 (w), 1324 (s), 1274 (w), 1161 (m), 1114 (s), 1063 (s), 1018 (m), 982 (w), 891 (w), 834 (m), 758 (w), 702 (m), 671 (w), 600 (m), 557 (m), 508 (w). **HR-MS:** (ESI) = *m/z* calcd. for: $C_{12}H_{12}F_3N_2O_3$ $[M+H]^+$ 289.0800 u, found: 289.0791 u. **$[a]_\lambda^T$:** (c = 1.12 g/100 mL, MeOH) = $[a]_D^{20}$: –10.00°.

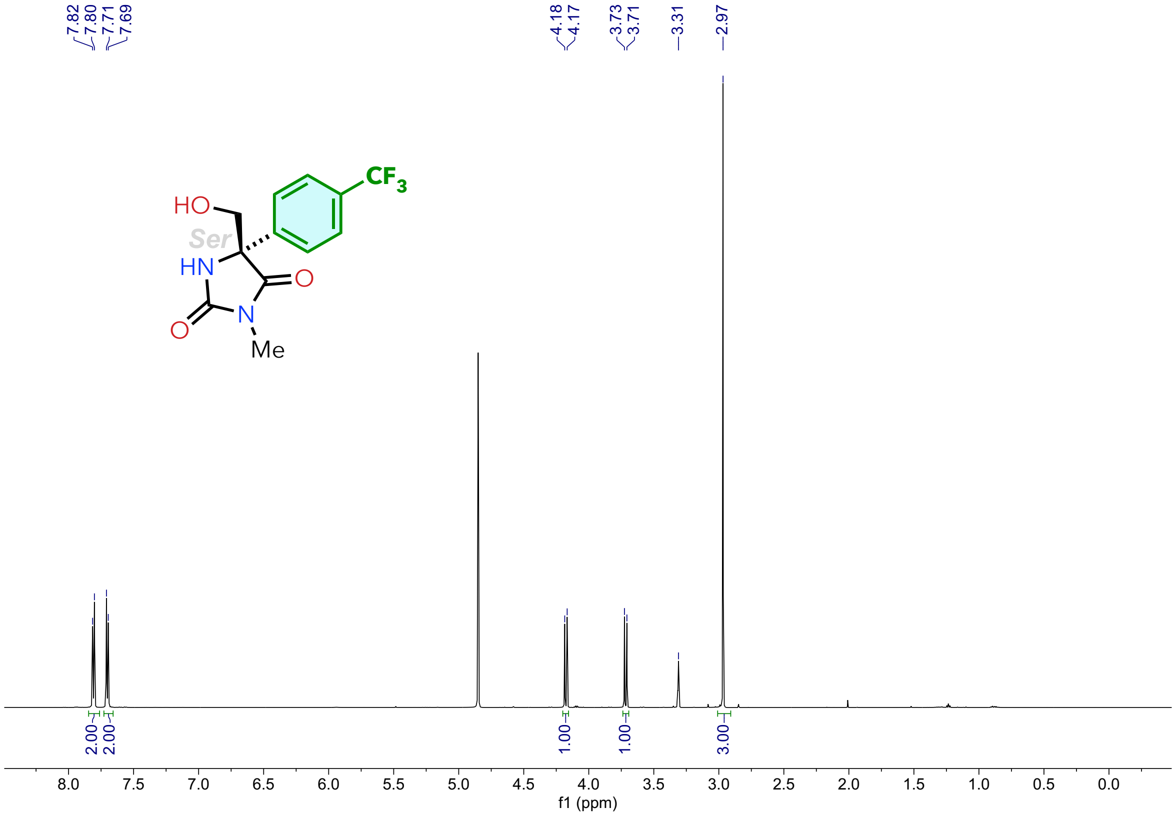
7.82
7.80
7.71
7.69
4.18
4.17
3.73
3.71
3.31
2.97
CF3
HO
Ser
HN
O
N
Me
O
2.00
2.00
1.00
1.00
3.00
8.0 7.5 7.0 6.5 6.0 5.5 5.0 4.5 4.0 3.5 3.0 2.5 2.0 1.5 1.0 0.5 0.0
f1 (ppm)

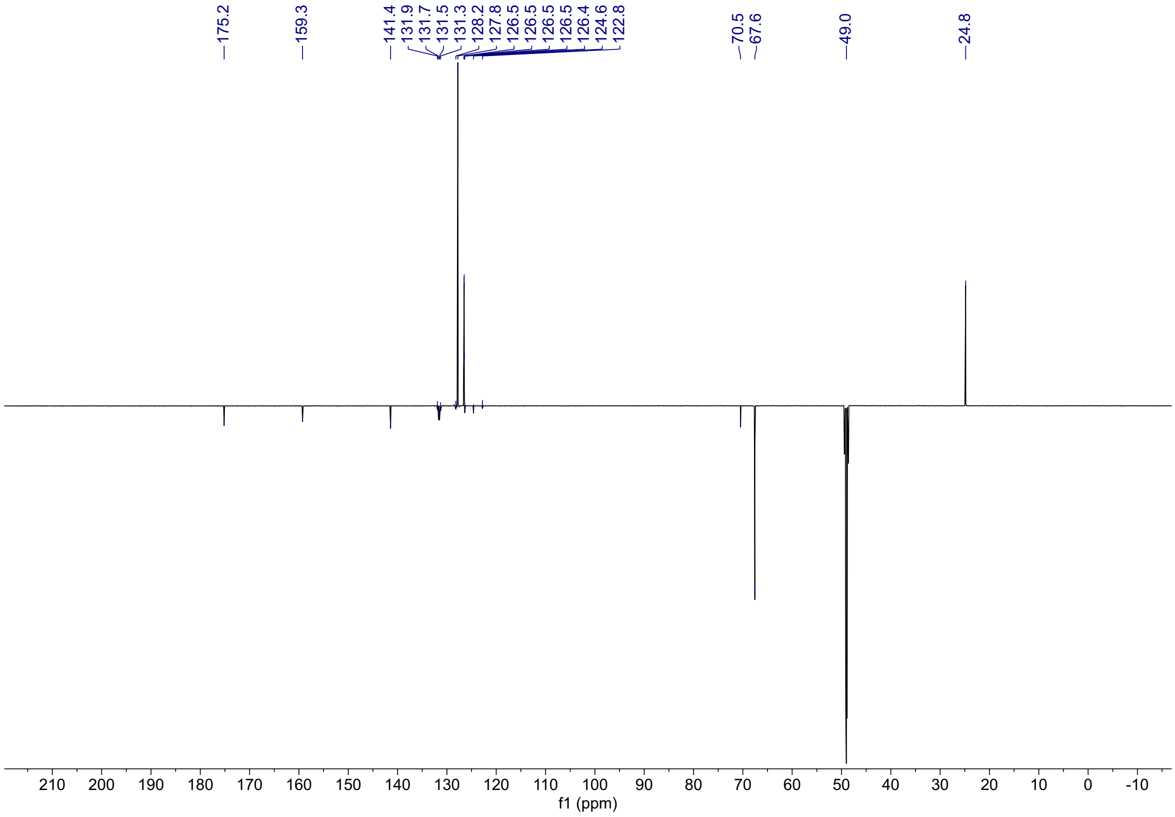
175.2
159.3
141.4
131.9
131.7
131.5
131.3
128.2
127.8
126.5
126.5
126.5
126.5
126.4
124.6
122.8
70.5
67.6
49.0
24.8
210 200 190 180 170 160 150 140 130 120 110 100 90 80 70 60 50 40 30 20 10 0 -10
f1 (ppm)

(*S*)-5-(bromo-3-fluorophenyl)-5-(hydroxymethyl)-3-methylimidazolidine-2,4-dione (27d)

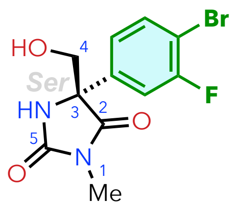

27d

Following **GP5**, a solution of rearranged urea **5o** (75 mg, 0.19 mmol, 1.0 equiv.) was suspended in a HCl/EtOH-solution mixture (5.5 mL, 6 M, aq.; 10:1). The title compound **27d** was obtained as a white solid (56 mg, 0.18 mmol, 91%) after purification by silica gel column chromatography (PE/EA, gradient elution).

Formula: $C_{11}H_{10}BrFN_2O_3$, **MW:** 317.11 g/mol, **m.p.:** 237 – 240 °C. **TLC:** R_f = 0.27 (PE/EA 1:2), $KMnO_4$ stain. **^{1}H NMR** (600 MHz, CD_3OD): δ [ppm] = 7.65 (dd, *J* 8.4, 7.2 Hz, 1H, Ar), 7.47 (dd, *J* 10.2, 2.2 Hz, 1H, Ar), 7.35 (dd, *J* 8.4, 2.1 Hz, 1H, Ar), 4.10 (d, *J* 11.3 Hz, 1H, H-4a), 3.68 (d, *J* 11.3 Hz, 1H, H-4b), 2.96 (s, 3H, H-1, Me). **^{13}C NMR** (150 MHz, CD_3OD): δ [ppm] = 175.0 (s, C-2), 161.1 (s, C-5, urea), 159.5 (s, Ar), 159.2 (s, Ar), 139.3 (s, *d J* 6.6 Hz, Ar), 134.9 (d, Ar), 124.3 (d, *d J* 3.7 Hz, Ar), 115.4 (d, *d J* 24.7 Hz, Ar), 109.8 (s, *d J* 20.9 Hz, Ar), 70.0 (s, C-3, *alpha*-carbon), 67.5 (t, C-4), 24.8 (q, C-1, Me). **^{19}F NMR** (565 MHz, $CDCl_3$, C_6F_6 ref.): δ [ppm] = –108.08 (dd, *J* 10.2, 7.4 Hz, F). **FT-IR (ATR):** $\tilde{\nu}$ [cm^{-1}] = 3465 (br w), 3233 (br w), 1780 (w), 1704 (vs), 1470 (m), 1412 (m), 1397 (m), 1307 (w), 1248 (w), 1063 (m), 1032 (m), 931 (w), 869 (w), 843 (m), 764 (w), 712 (w), 621 (w), 549 (m), 450 (w). **$[a]_\lambda^T$:** (c = 1.11 g/100 mL, MeOH) = $[a]_D^{20}$: –9.01°.

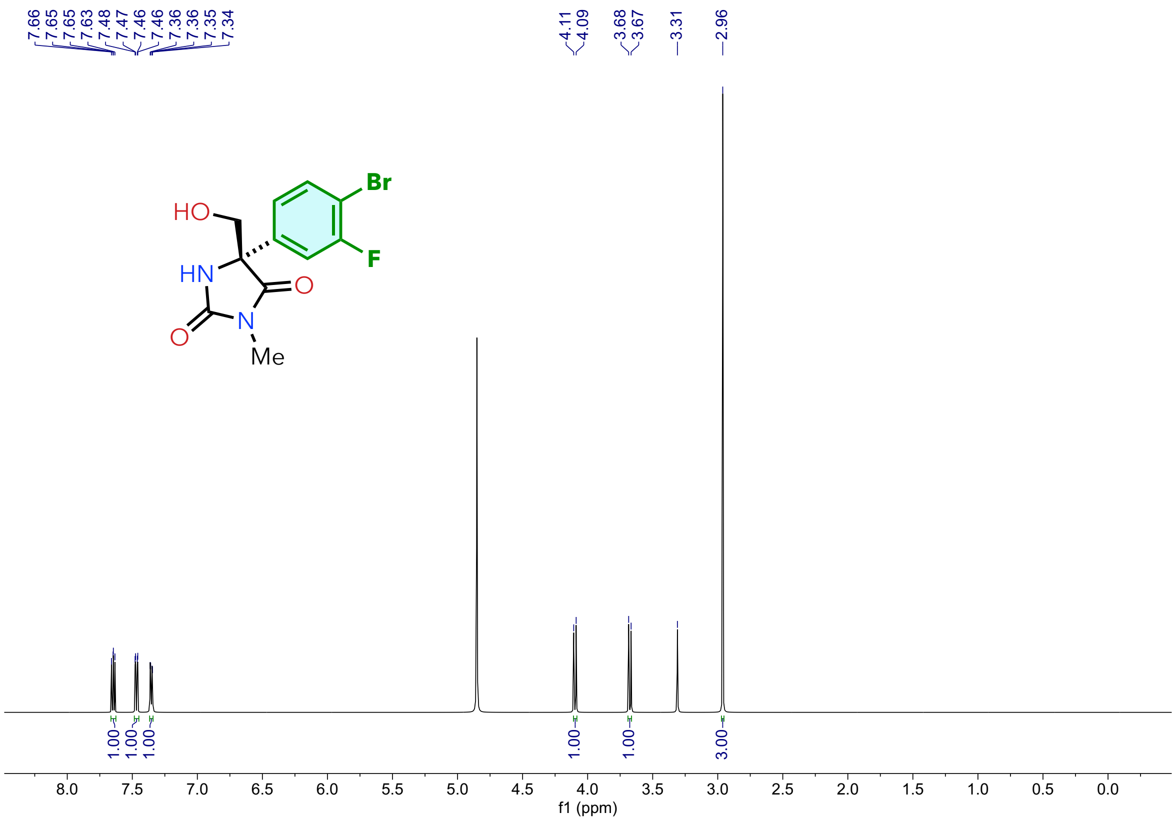
7.66
7.65
7.65
7.63
7.48
7.47
7.46
7.46
7.36
7.36
7.35
7.34
4.11
4.09
3.68
3.67
3.31
2.96
HO
Br
F
HN
O
O
N
Me
1.00
1.00
1.00
1.00
1.00
3.00
8.0 7.5 7.0 6.5 6.0 5.5 5.0 4.5 4.0 3.5 3.0 2.5 2.0 1.5 1.0 0.5 0.0
f1 (ppm)

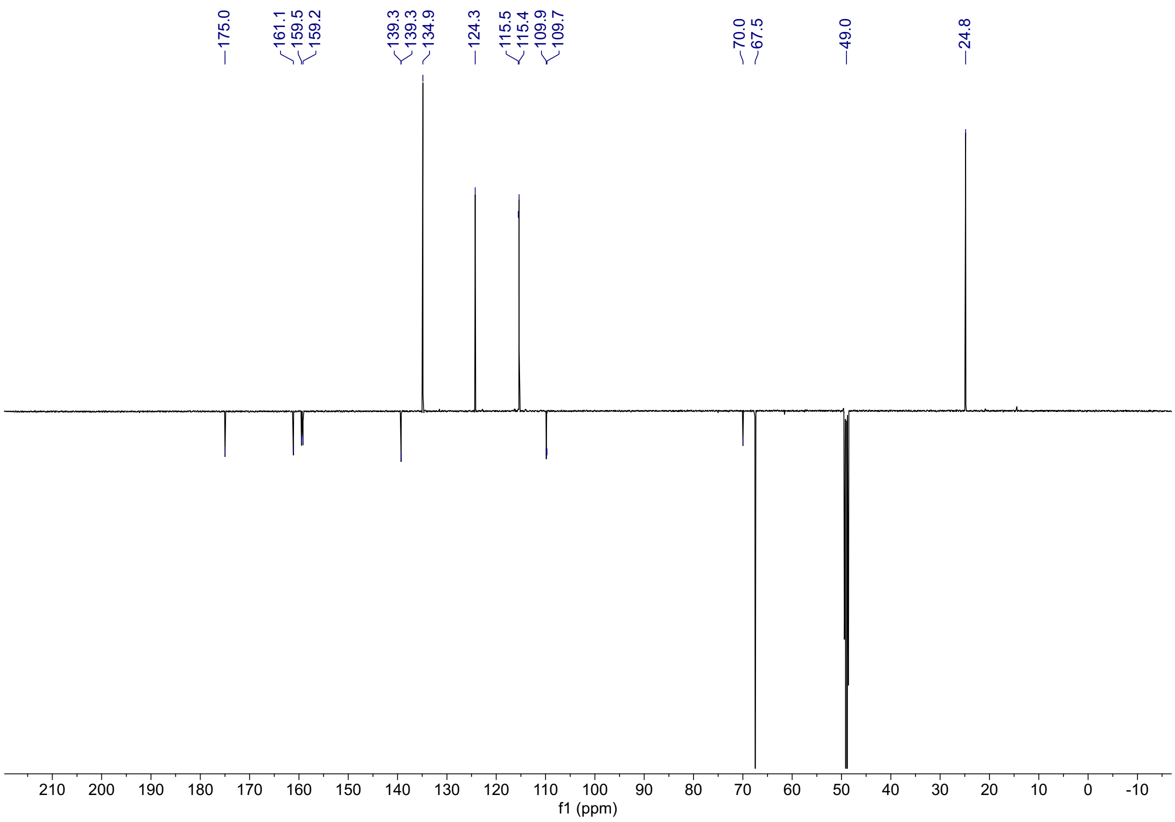
175.0
161.1
159.5
159.2
139.3
139.3
134.9
124.3
115.5
115.4
109.9
109.7
70.0
67.5
49.0
24.8
210 200 190 180 170 160 150 140 130 120 110 100 90 80 70 60 50 40 30 20 10 0 -10
f1 (ppm)

(*S*)-5-(hydroxymethyl)-5-(2-methoxyphenyl)-3-methylimidazolidine-2,4-dione (27e)

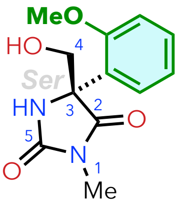

27e

Following **GP5**, a solution of rearranged urea **51** (120 mg, 0.38 mmol, 1.0 equiv.) was suspended in a HCl/EtOH-solution mixture (5.5 mL, 6 M, aq.; 10:1). The title compound **27e** was obtained as a white solid (73 mg, 0.29 mmol, 77%) after purification by silica gel column chromatography (PE/EA, gradient elution).

Formula: $C_{12}H_{14}N_2O_4$, **MW:** 250.25 g/mol, **m.p.:** 202 – 205 °C. **TLC:** R_f = 0.18 (PE/EA 1:2), $KMnO_4$ stain. **^{1}H NMR** (600 MHz, CD_3OD): δ [ppm] = 7.38 (dd, *J* 7.8, 1.6 Hz, 2H, Ar), 7.37 – 7.34 (m, 1H, Ar), 7.04 (d, *J* 8.0 Hz, 1H, Ar), 4.19 (d, *J* 10.9 Hz, 1H, H-4a), 4.07 (d, *J* 10.9 Hz, 1H, H-4b), 3.08 (s, 3H, Ar, OMe), 3.04 (s, 3H, H-1, Me). **^{13}C NMR** (150 MHz, CD_3OD): δ [ppm] = 177.5 (s, C-2), 160.1 (s, C-5, urea), 159.1 (s, Ar), 131.4 (d, Ar), 128.2 (d, Ar), 125.1 (s, Ar, OMe), 121.8 (d, Ar), 113.0 (d, Ar), 68.0 (s, C-3, *alpha*-carbon), 65.1 (t, C-4), 56.3 (q, Ar, OMe), 24.7 (q, C-1, Me). **FT-IR (ATR):** $\tilde{\nu}$ [cm^{-1}] = 3407 (br w), 3255 (br w), 1774 (m), 1694 (vs), 1492 (w), 1461 (m), 1401 (w), 1296 (w), 1248 (m), 1181 (w), 1168 (w), 1146 (w), 1063 (m), 1022 (w), 902 (w); 757 (m), 738 (w), 709 (w), 646 (w), 578 (m), 566 (m), 540 (m), 479 (w). **HR-MS:** (ESI) = *m*/*z* calcd. for: $C_{12}H_{15}N_2O_4$ [M+H]$^+$ 251.1024 u, found: 251.1026 u. **$[a]_\lambda^T$:** (c = 1.01 g/100 mL, MeOH) = $[a]_D^{20}$: –6.34°.

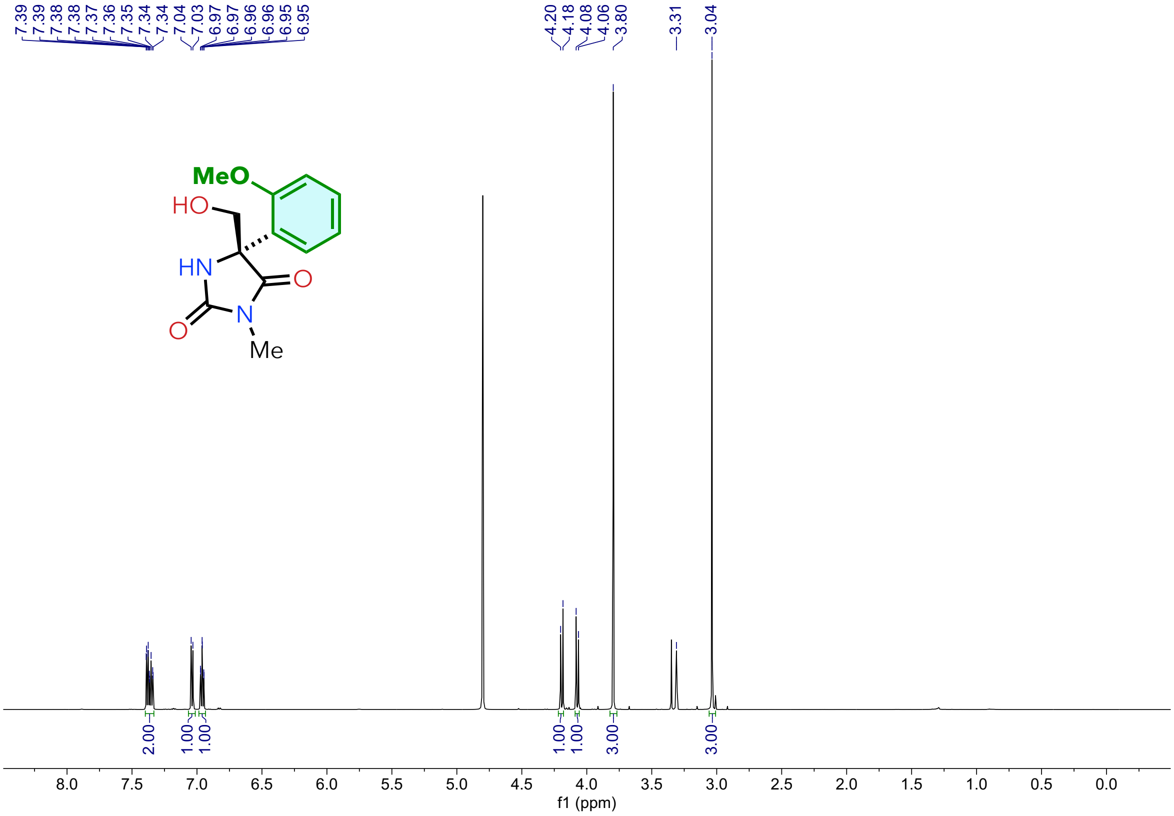
7.39
7.39
7.38
7.38
7.37
7.36
7.35
7.34
7.34
7.04
7.03
6.97
6.97
6.96
6.96
6.95
6.95
4.20
4.18
4.08
4.06
3.80
3.31
3.04
MeO
HO
HN
O
O
N
Me
2.00
1.00
1.00
1.00
1.00
3.00
3.00
f1 (ppm)

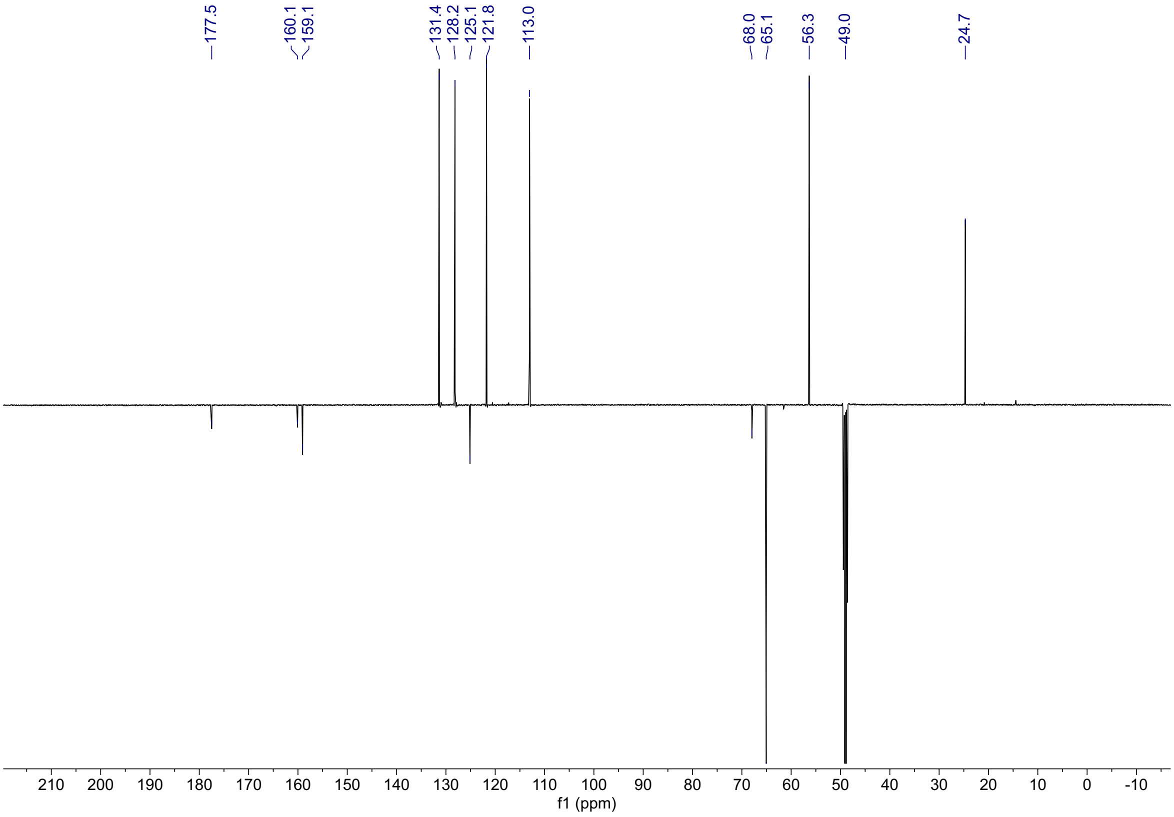
177.5
160.1
159.1
131.4
128.2
125.1
121.8
113.0
68.0
65.1
56.3
49.0
24.7
f1 (ppm)

(*S*)-3-(4-(hydroxymethyl)-1-methyl-2,5-dioxoimidazolidin-4-yl)benzoic acid (27f)

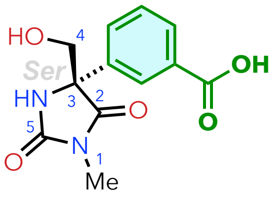

27f

Following **GP5**, a solution of rearranged urea **5h** (50 mg, 0.16 mmol, 1.0 equiv.) was suspended in a HCl/EtOH-solution mixture (1.1 mL, 6 M, aq.; 10:1). The title compound **27f** was obtained as a colourless oil (30 mg, 0.11 mmol, 71%) after purification by silica gel column chromatography (PE/EA, gradient elution).

Formula: $C_{12}H_{12}N_2O_5$, **MW:** 264.24 g/mol. **TLC:** R_f = 0.16 (PE/EA 1:2), $KMnO_4$ stain. **^{1}H NMR** (600 MHz, CD_3OD): δ [ppm] = 8.27 (s, 1H, Ar), 8.00 (d, *J* 7.7 Hz, 1H, Ar), 7.81 (d, *J* 7.9 Hz, 1H, Ar), 7.49 (d, *J* 7.8 Hz, 1H, Ar), 4.19 (d, *J* 11.3 Hz, 1H, H-4a), 3.72 (d, *J* 11.3 Hz, 1H, H-4b), 2.97 (s, 3H, H-1, Me). **^{13}C NMR** (150 MHz, CD_3OD): δ [ppm] = 175.6 (s, C-2), 170.7 (s, Ar, C=OOH), 159.4 (s, C-5, urea), 137.5 (s, Ar), 133.5 (s, Ar, –COOH), 131.1 (d, Ar), 130.7 (d, Ar), 129.8 (d, Ar), 128.1 (d, Ar), 70.4 (s, C-3, *alpha*-carbon), 67.4 (t, C-4), 24.8 (q, C-1, Me). **FT-IR (ATR):** $\tilde{\nu}$ [cm^{-1}] = 3271 (br w), 1772 (w), 1693 (br vs), 1548 (w), 1464 (m), 1393 (m), 1302 (m), 1258 (m), 1211 (m), 1065 (m), 1028 (m), 760 (w), 736 (w), 665 (w), 645 (w), 564 (w), 538 (w). **HR-MS:** (Nanospray) = *m/z* calcd. for: $C_{12}H_{11}N_2O_5$ $[M–H]^-$ 263.0668 u, found: 263.0667 u. **$[a]_\lambda^T$:** (c = 1.00 g/100 mL, MeOH) = $[a]_D^{20}$: –8.80°.

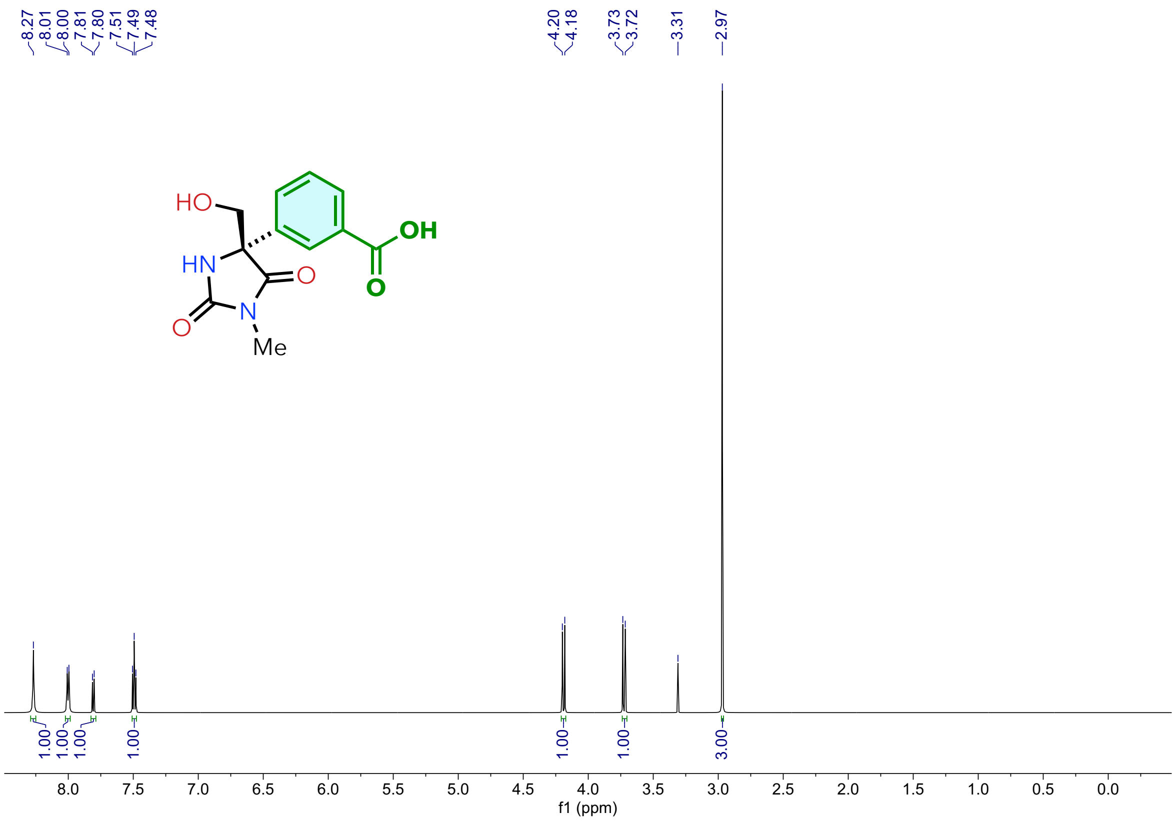
8.27
8.01
8.00
7.81
7.80
7.51
7.49
7.48
4.20
4.18
3.73
3.72
3.31
2.97
HO
HN
O
N
Me
O
OH
O
1.00
1.00
1.00
1.00
1.00
1.00
3.00
8.0 7.5 7.0 6.5 6.0 5.5 5.0 4.5 4.0 3.5 3.0 2.5 2.0 1.5 1.0 0.5 0.0
f1 (ppm)

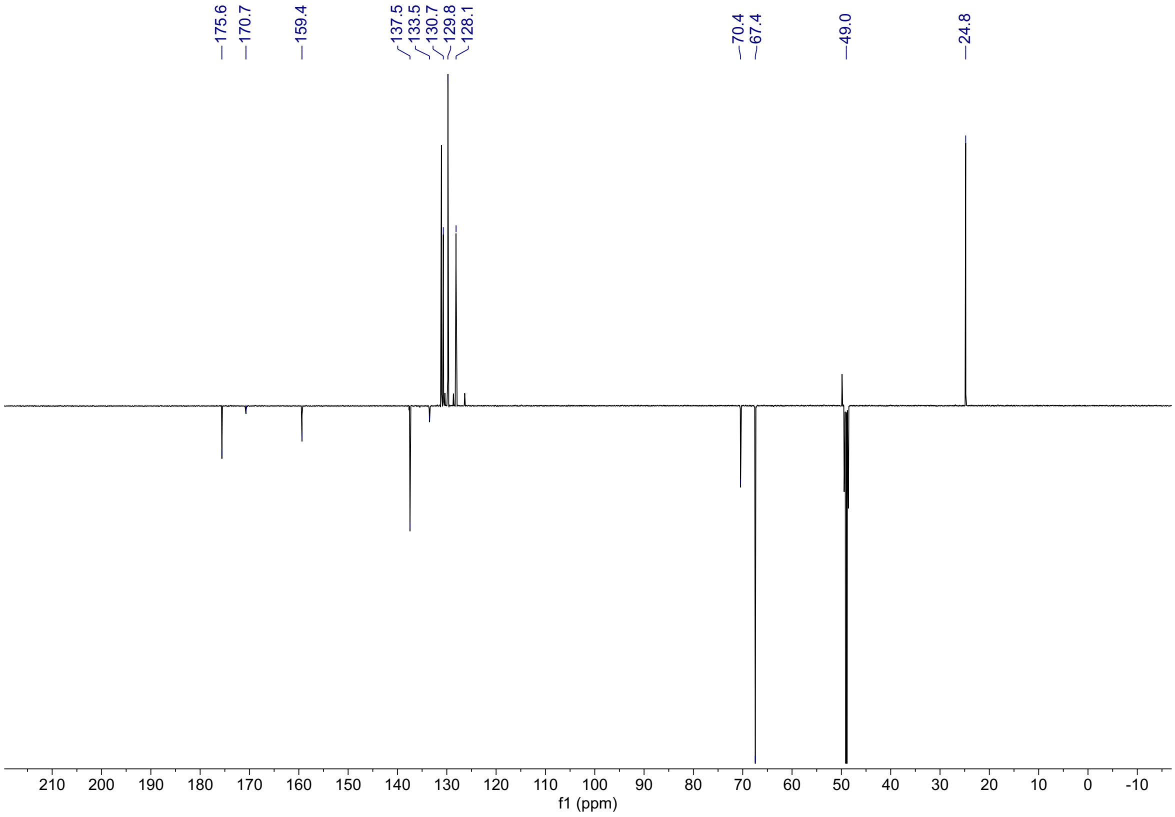
175.6
170.7
159.4
137.5
133.5
130.7
129.8
128.1
70.4
67.4
49.0
24.8
210 200 190 180 170 160 150 140 130 120 110 100 90 80 70 60 50 40 30 20 10 0 -10
f1 (ppm)

(*R*)-5-(Mercaptomethyl)-3-methyl-5-phenylimidazolidine-2,4-dione (28a)

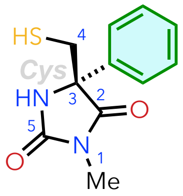

28a

Following **GP5**, a solution of rearranged urea **23a** (250 mg, 0.82 mmol, 1.0 equiv.) was suspended in a HCl/EtOH-solution mixture (6 M, aq.; 10:1). The title compound **28a** was obtained as a colourless oil (132 mg, 0.56 mmol, 68%) after purification by silica gel column chromatography (PE/EA, gradient elution).

Formula: $C_{11}H_{12}N_2O_2S$, **MW:** 236.29 g/mol. **TLC:** R_f = 0.32 (PE/EA 1:2), $KMnO_4$ stain. **^{1}H NMR** (600 MHz, CD_3OD): δ [ppm] = 7.61 – 7.54 (m, 2H, Ar), 7.40 (td, *J* 6.9, 1.7 Hz, 2H, Ar), 7.36 – 7.32 (m, 2H, Ar), 3.65 (d, *J* 14.3 Hz, 1H, H-4a), 3.36 (d, *J* 14.3 Hz, 1H, H-4b), 2.97 (s, 3H, H-1, Me). **^{13}C NMR** (150 MHz, CD_3OD): δ [ppm] = 175.4 (s, C-2), 158.6 (s, C-5, urea), 138.6 (s, Ar), 129.9 (d, 2Ar), 129.7 (d, Ar), 126.8 (d, 2Ar), 68.5 (t, C-4), 50.6 (s, C-3, *alpha*-carbon), 25.0 (q, C-1, Me). **FT-IR (ATR):** $\tilde{\nu}$ [cm^{-1}] = 3282 (br w), 1771 (m), 1701 (vs), 1448 (m), 1393 (m), 1307 (w), 1280 (w), 1099 (w), 1016 (w), 976 (w), 757 (w), 694 (m), 547 (w), 490 (w). **HR-MS:** (MALDI) = *m/z* calcd. for: $C_{22}H_{24}N_4O_4S_2Na$ $[2M+Na]^+$ 493.09 u, found: 493.1 u. **$[a]_\lambda^T$:** (c = 1.00 g/100 mL, MeOH) = $[a]_D^{20}$: –0.80°.

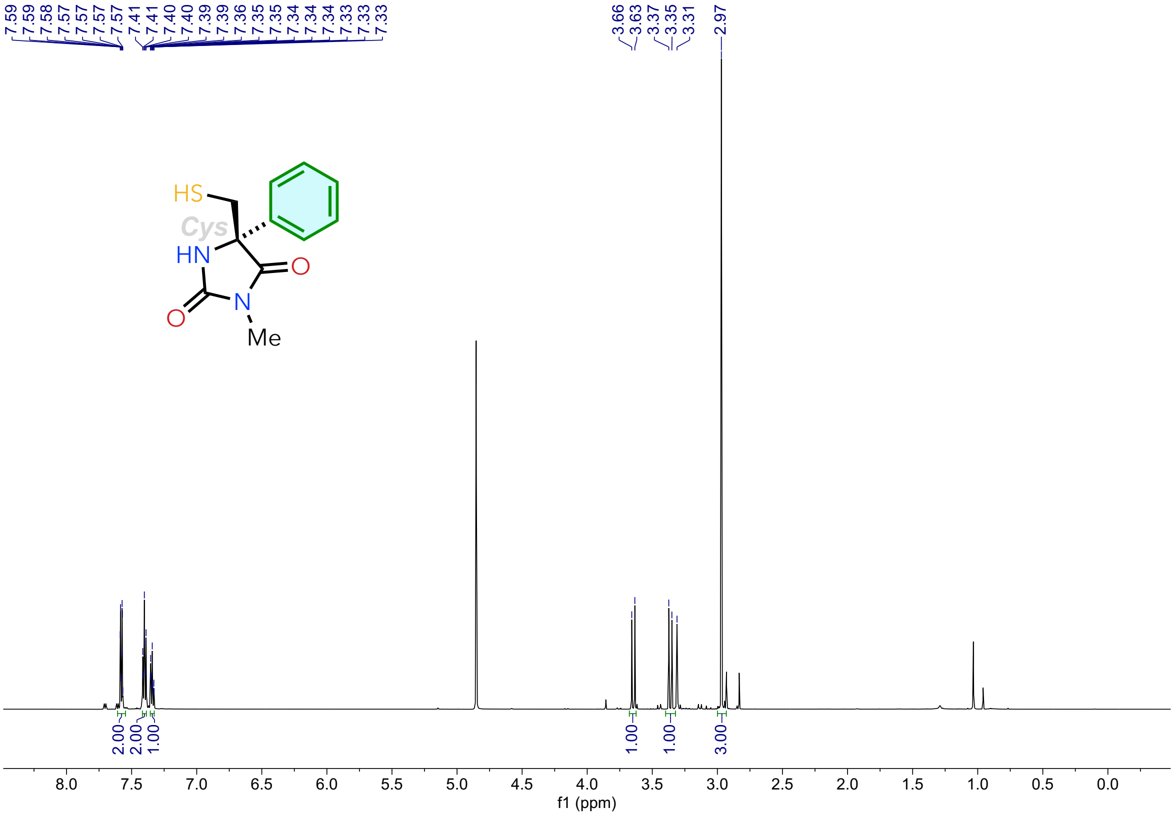
7.59 7.59 7.58 7.57 7.57 7.57 7.57 7.41 7.41 7.40 7.40 7.39 7.39 7.36 7.35 7.35 7.34 7.34 7.34 7.33 7.33 7.33
3.66 3.63 3.37 3.35 3.31 2.97
HS
Cys
HN
N
O
O
Me
2.00 2.00 1.00
1.00 1.00 3.00
f1 (ppm)

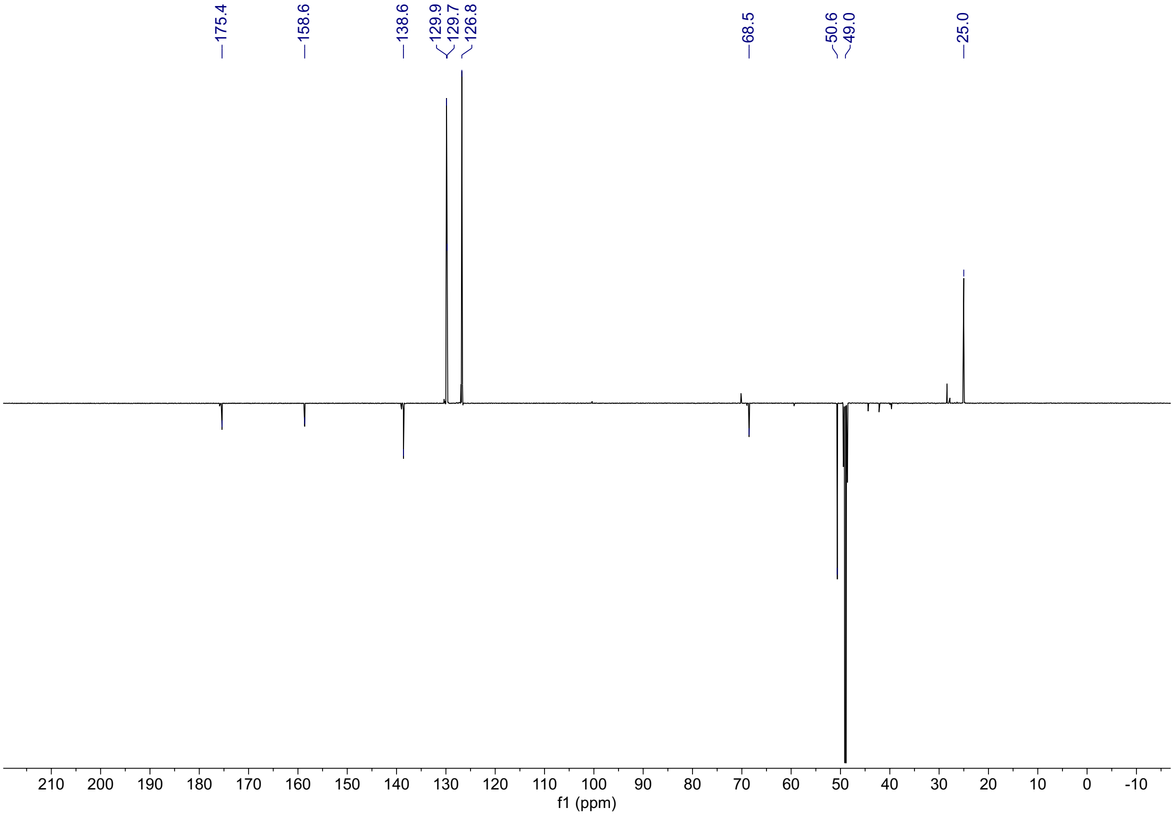
175.4 158.6 138.6 129.9 129.7 126.8 68.5 50.6 49.0 25.0
f1 (ppm)

(*R*)-5-(mercaptomethyl)-3-methyl-5-(naphthalen-2-yl)imidazolidine-2,4-dione (28b)

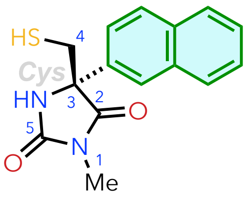

28b

Following **GP5**, a solution of rearranged urea **23n** (150 mg, 0.42 mmol, 1.0 equiv.) was suspended in a HCl/EtOH-solution mixture (6 M, aq.; 10:1). The title compound **28b** was obtained as a colourless oil (118 mg, 0.41 mmol, 98%) after purification by silica gel column chromatography (PE/EA, gradient elution).

Formula: $C_{15}H_{14}N_2O_2S$, **MW:** 286.35 g/mol. **TLC:** R_f = 0.60 (PE/EA 1:2), $KMnO_4$ stain. **^{1}H NMR** (600 MHz, CD_3OD): δ [ppm] = 8.21 (d, *J* 8.8 Hz, 1H, Ar), 7.90 (dd, *J* 8.1, 1.2 Hz, 1H, Ar), 7.87 (d, *J* 8.2 Hz, 1H, Ar), 7.74 (dd, *J* 7.4, 1.0 Hz, 1H, Ar), 7.54 (ddd, *J* 8.6, 6.8, 1.5 Hz, 1H, Ar), 7.49 (d, *J* 7.4, 6.9, 1.0 Hz, 1H, Ar), 7.45 – 7.40 (m, 1H, Ar), 3.54 (s, 2H, H-4), 3.07 (s, 3H, H-1, Me). **^{13}C NMR** (150 MHz, CD_3OD): δ [ppm] = 175.9 (s, C-2), 159.2 (s, C-5, urea), 136.4 (s, Ar), 133.9 (s, Ar),131.8 (s, Ar), 131.4 (d, Ar), 130.6 (d, Ar), 127.6 (d, Ar), 126.8 (d, Ar), 126.4 (d, Ar), 126.0 (d, Ar), 125.7 (d, Ar), 70.8 (s, C-3, *alpha*-carbon), 33.2 (t, C-4), 24.8 (q, C-1, Me). **FT-IR (ATR):** $\tilde{\nu}$ [cm^{-1}] = 3280 (br w), 2923 (br w), 1772 (w), 1699 (vs), 1511 (w), 1455 (m), 1392 (m), 1346 (w), 1306 (w), 1102 (w), 1021 (w), 804 (w), 775 (s), 759 (m), 704 (w), 640 (w), 533 (w), 505 (w), 425 (m). **$[a]_\lambda^T$:** (c = 1.05 g/100 mL, MeOH) = $[a]_D^{20}$: –0.76°.

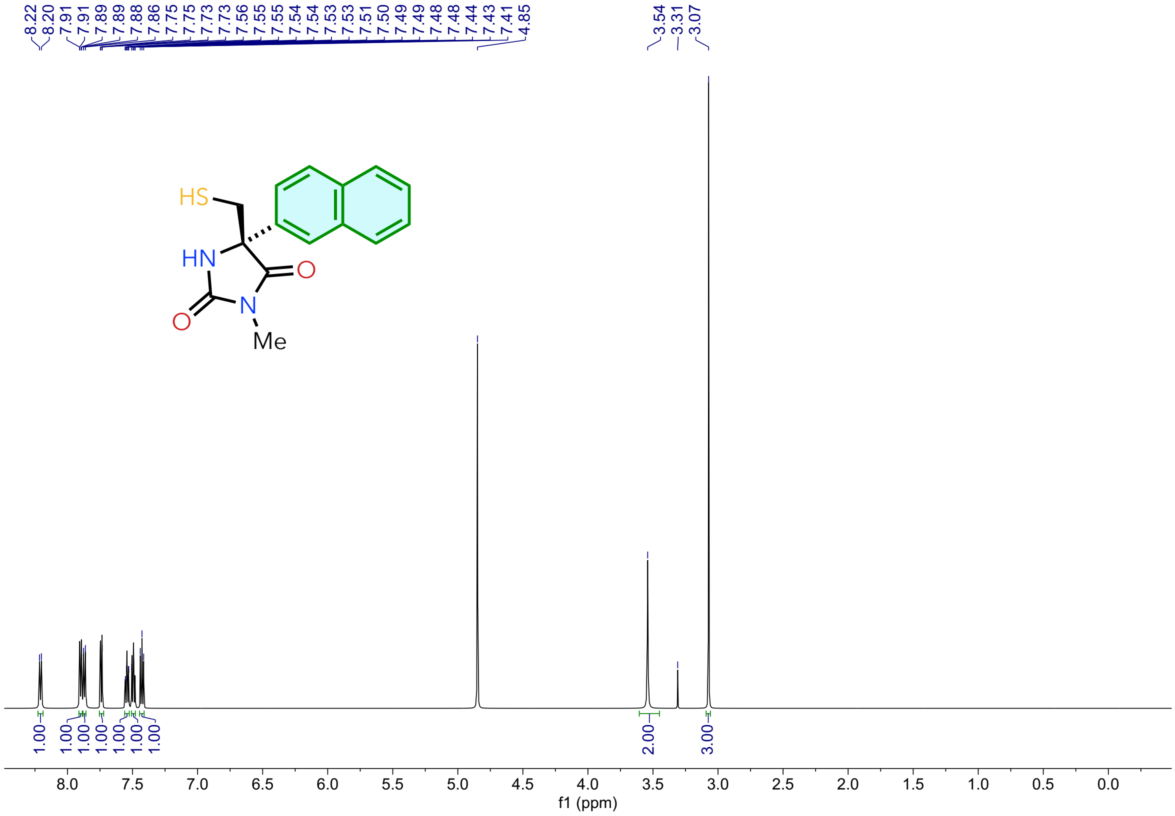
8.22
8.20
7.91
7.89
7.89
7.88
7.86
7.75
7.73
7.73
7.56
7.55
7.55
7.54
7.54
7.53
7.53
7.51
7.50
7.49
7.49
7.48
7.48
7.44
7.43
7.41
4.85
3.54
3.31
3.07
HS
HN
N
O
O
Me
1.00
1.00
1.00
1.00
1.00
1.00
1.00
2.00
3.00
f1 (ppm)

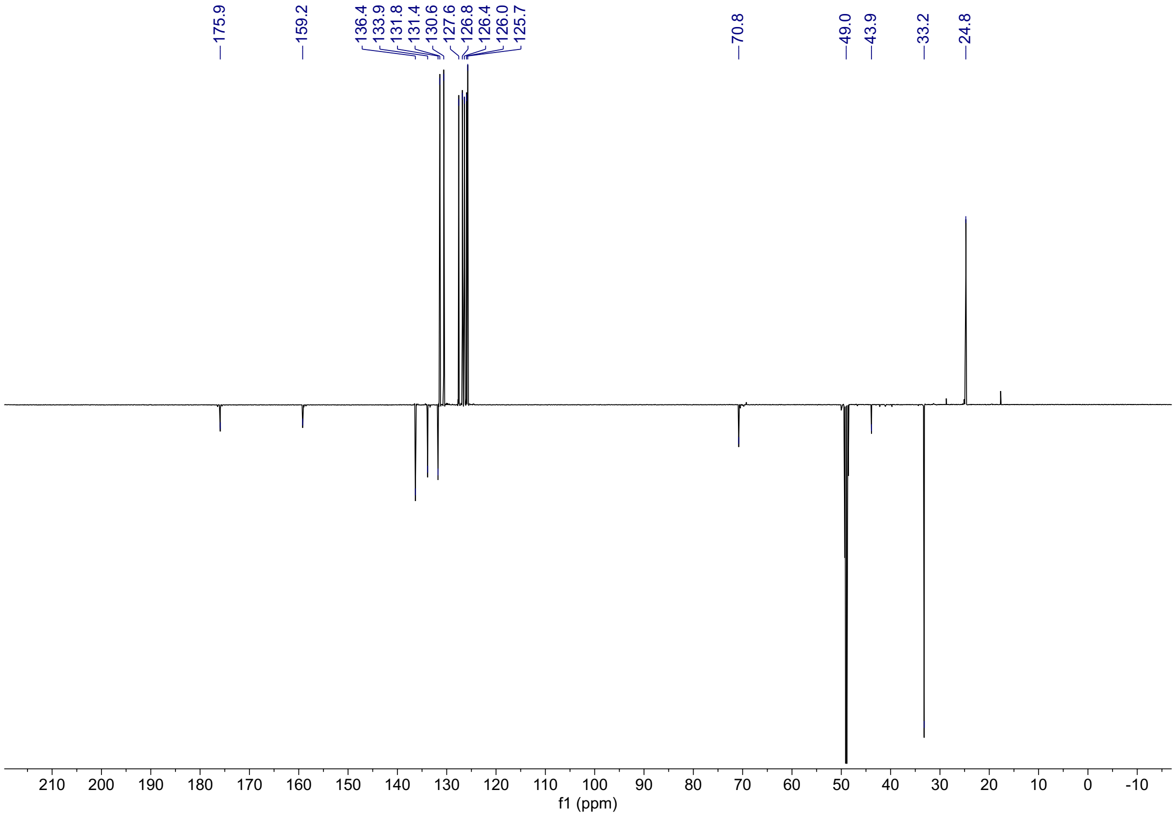
175.9
159.2
136.4
133.9
131.8
131.4
130.6
127.6
126.8
126.4
126.0
125.7
70.8
49.0
43.9
33.2
24.8
f1 (ppm)

(*S*)-5-(Aminomethyl)-3-methyl-5-phenylimidazolidine-2,4-dione (29a)

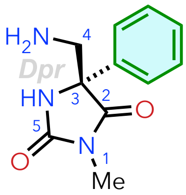

29a

Following **GP5**, a solution of rearranged urea **24a** (100 mg, 0.26 mmol, 1.0 equiv.) was suspended in a HCl/EtOH-solution mixture (6 M, aq.; 10:1). The title compound **29a** was obtained as a brown white solid (48 mg, 0.22 mmol, 84%) after purification by silica gel column chromatography (PE/EA, gradient elution).

Formula: $C_{11}H_{13}N_3O_2$, **MW:** 219.24 g/mol, **m.p.:** 242 – 245 °C. **TLC:** R_f = 0.15 (PE/EA 1:5), $KMnO_4$ stain. **^{1}H NMR** (600 MHz, CD_3OD): δ [ppm] = 7.56 (d, *J* 8.2 Hz, 2H, Ar), 7.40 (t, *J* 7.7 Hz, 2H, Ar), 7.35 (t, *J* 7.1 Hz, 1H, Ar), 3.41 (d, *J* 13.6 Hz, 1H, H-4a), 3.05 (d, *J* 13.6 Hz, 1H, H-4b), 2.96 (s, 3H, H-1, Me). **^{13}C NMR** (150 MHz, CD_3OD): δ [ppm] = 176.0 (s, C-2), 159.1 (s, C-5, urea), 138.0 (s, Ar), 129.9 (d, 2Ar), 129.6 (d, Ar), 126.6 (d, Ar), 70.2 (t, C-4), 49.6 (s, C-3, *alpha*-carbon), 24.8 (q, C-1, Me). **FT-IR (ATR):** $\tilde{\nu}$ [cm^{-1}] = 3296 (br w), 1771 (m), 1694 (vs), 1447 (m), 1392 (m), 1299 (w), 1283 (w), 1012 (w), 758 (m), 696 (m), 648 (w), 557 (m), 496 (w). **HR-MS:** (ESI) = *m*/*z* calcd. for: $C_{11}H_{14}N_3O_2$ $[M+H]^+$ 220.1086 u, found: 220.1071 u. **$[a]_\lambda^T$:** (c = 1.00 g/100 mL, $CHCl_3$) = $[a]_D^{20}$: –13.20°.

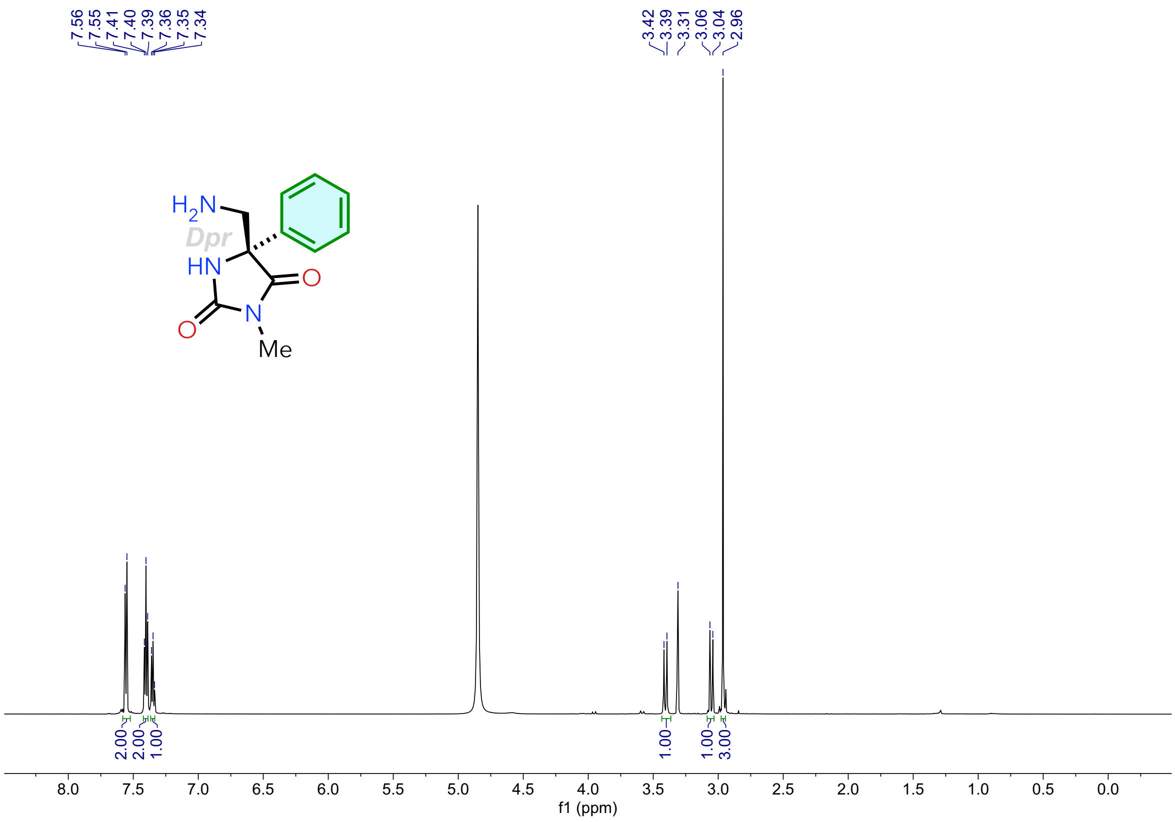
7.56
7.55
7.41
7.40
7.39
7.36
7.35
7.34
3.42
3.39
3.31
3.06
3.04
2.96
H2N
Dpr
HN
O
N
Me
O
2.00
2.00
1.00
1.00
1.00
3.00
8.0 7.5 7.0 6.5 6.0 5.5 5.0 4.5 4.0 3.5 3.0 2.5 2.0 1.5 1.0 0.5 0.0
f1 (ppm)

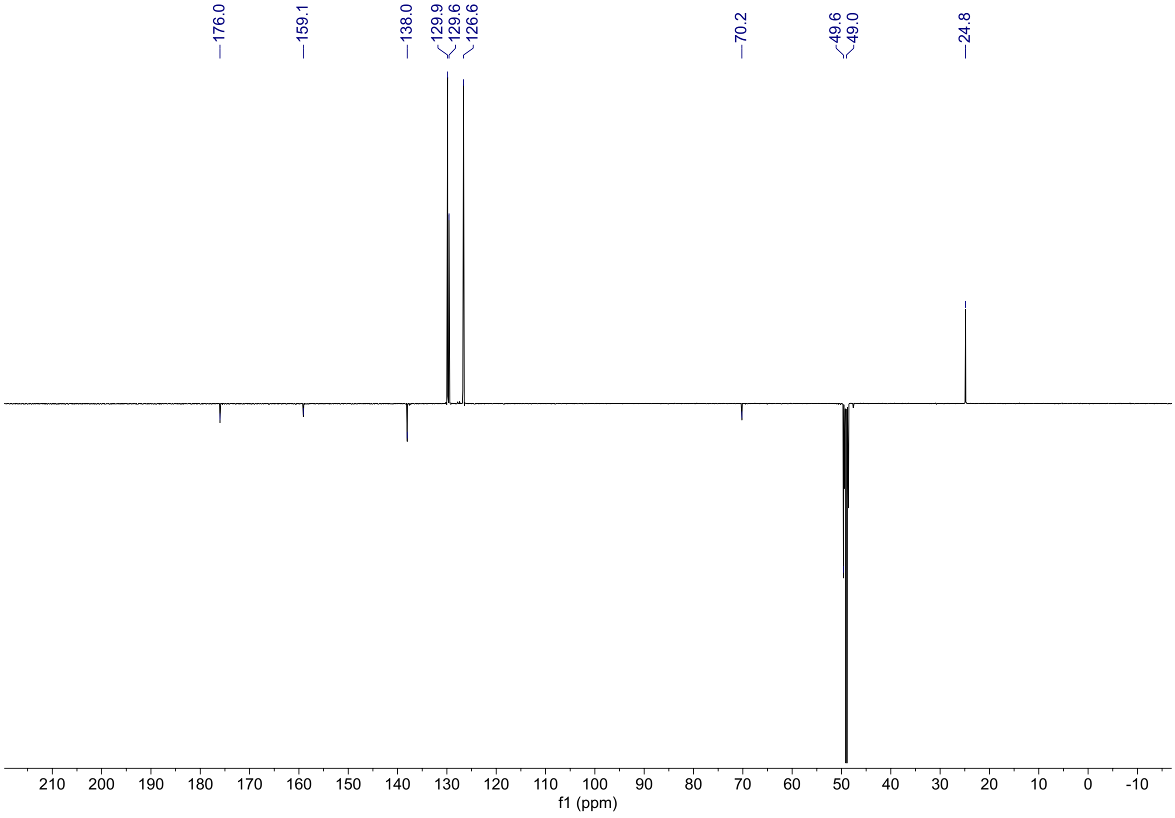
176.0
159.1
138.0
129.9
129.6
126.6
70.2
49.6
49.0
24.8
210 200 190 180 170 160 150 140 130 120 110 100 90 80 70 60 50 40 30 20 10 0 -10
f1 (ppm)

(*S*)-5-((*R*)-1-Hydroxyethyl)-3-methyl-5-phenylimidazolidine-2,4-dione (30a)

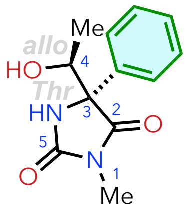

30a

Following **GP5**, a solution of rearranged urea **26a** (64 mg, 0.21 mmol, 1.0 equiv.) was suspended in a HCl/EtOH-solution mixture (3 mL, 6 M, aq.; 10:1). The title compound **30a** was obtained as a colourless oil (20 mg, 0.09 mmol, 40%) after purification by silica gel column chromatography (PE/EA, gradient elution).

Formula: $C_{12}H_{14}N_2O_3$, **MW:** 234.26 g/mol. **TLC:** R_f = 0.36 (PE/EA 1:2), $KMnO_4$ stain. **^{1}H NMR** (600 MHz, CD_3OD): δ [ppm] = 7.64 (d, *J* 7.5 Hz, 2H, Ar), 7.40 (t, *J* 7.5 Hz, 2H, Ar), 7.35 (t, *J* 7.2 Hz, 1H, Ar), 4.45 (q, *J* 6.2 Hz, 1H, H-4), 2.96 (s, 3H, H-1, Me), 1.20 (d, *J* 6.2 Hz, 3H, H-4, Me). **^{13}C NMR** (150 MHz, CD_3OD): δ [ppm] = 173.2 (s, C-2), 157.8 (s, C-5, urea), 135.9 (s, Ar), 129.0 (d, 2Ar), 128.9 (d, Ar), 126.0 (d, 2Ar), 72.6 (t, C-4), 71.2 (s, C-3, *alpha*-carbon), 24.8 (q, C-1, Me), 16.8 (q, C-4, Me). **FT-IR (ATR):** $\tilde{\nu}$ [cm^{-1}] = 3214 (br w), 1773 (m), 1701 (vs), 1471 (m), 1446 (m), 1396 (m), 1342 (w), 1308 (w), 1195 (w), 1102 (w), 1080 (w), 969 (w), 830 (w), 758 (w), 695 (m), 539 (w). **HR-MS:** (ESI) = *m/z* calcd. for: $C_{12}H_{15}N_2O_3$ $[M+H]^+$ 235.1083 u, found: 235.1075 u. **$[a]_\lambda^T$:** (c = 1.00 g/100 mL, $CHCl_3$) = $[a]_D^{20}$: –11.20°.

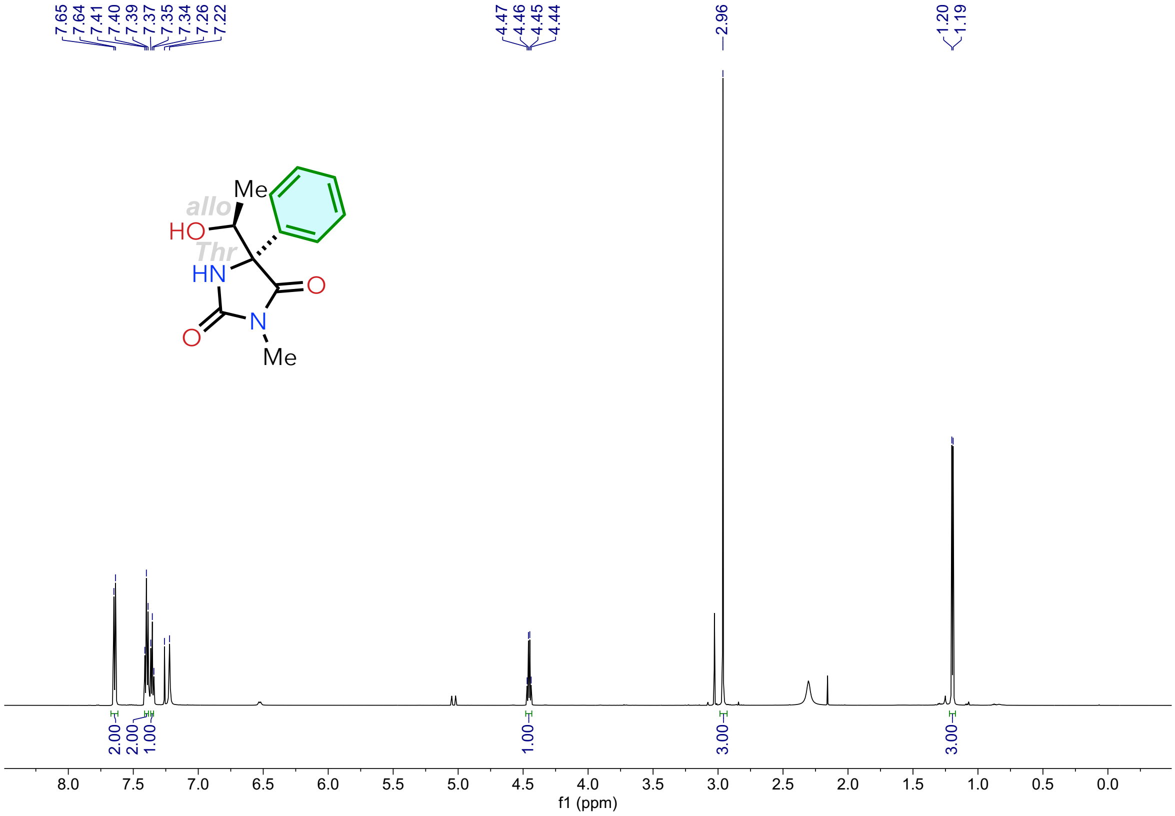
7.65
7.64
7.41
7.40
7.39
7.37
7.35
7.34
7.26
7.22
4.47
4.46
4.45
4.44
2.96
1.20
1.19
allo
Thr
Me
HO
HN
N
O
O
Me
2.00
2.00
1.00
1.00
3.00
3.00
8.0 7.5 7.0 6.5 6.0 5.5 5.0 4.5 4.0 3.5 3.0 2.5 2.0 1.5 1.0 0.5 0.0
f1 (ppm)

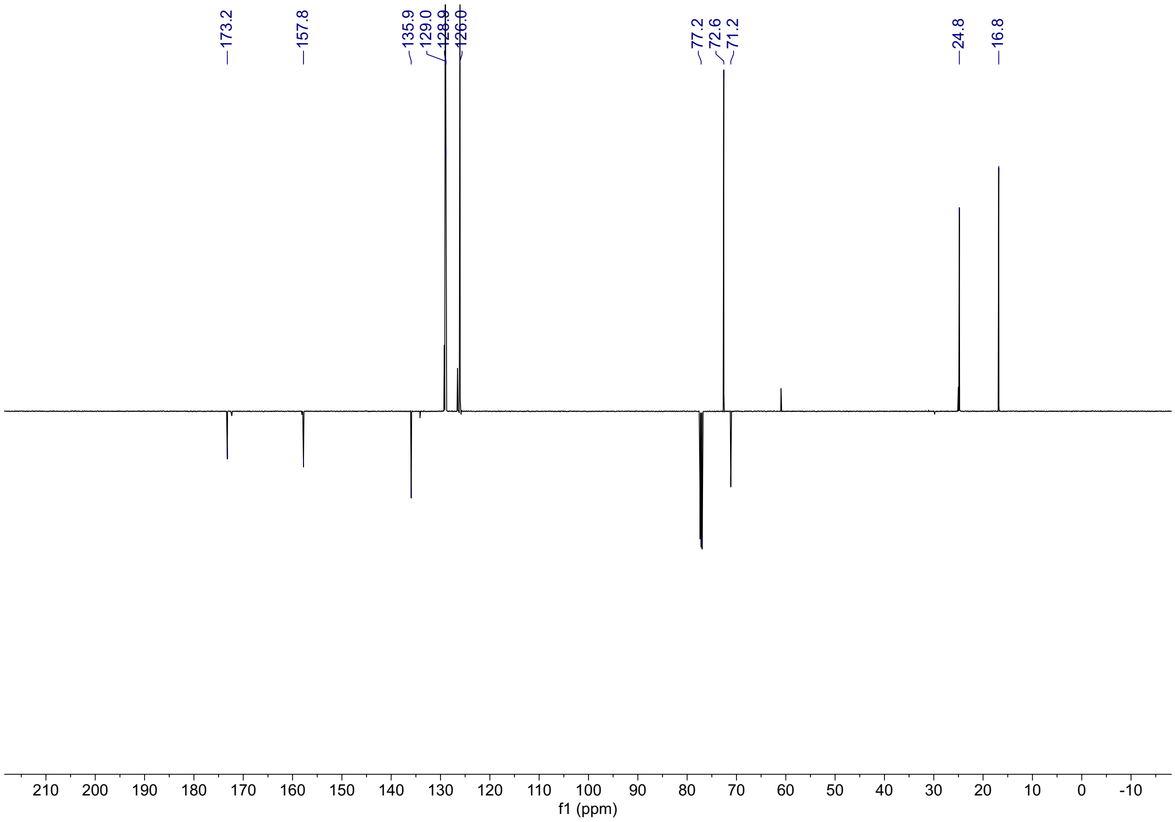
173.2
157.8
135.9
129.0
128.9
126.0
77.2
72.6
71.2
24.8
16.8
210 200 190 180 170 160 150 140 130 120 110 100 90 80 70 60 50 40 30 20 10 0 -10
f1 (ppm)

4.6 Basic Hydrolysis according to General Procedure 6 (GP6)

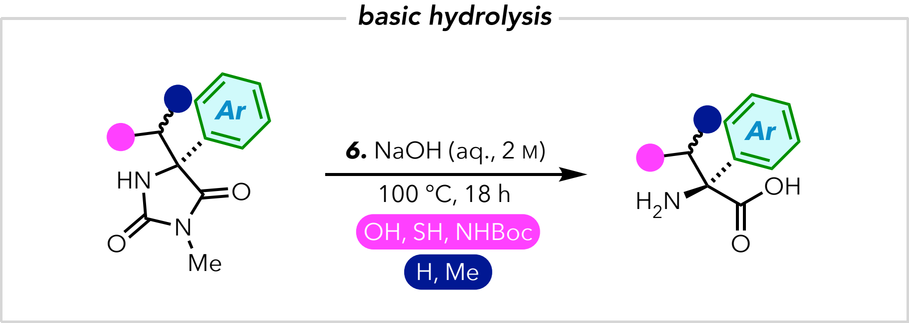

(*S*)-2-amino-3-hydroxy-2-phenylpropanoic acid (31)[2]

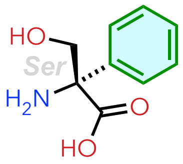

31

Following **GP6**, hydantoin **27a** (50 mg, 0.23 mmol, 1.0 equiv.) was dissolved in a water/dioxane mixture (0.6 mL, 5:1), and 0.5 mL of a 2 M aqueous sodium hydroxide solution was added. The title compound **31** was obtained as a white solid (38 mg, 0.21 mmol, 93%) after purification by silica gel column chromatography (CH_2Cl_2/MeOH, gradient elution).

Formula: $C_9H_{11}NO_3$, **MW:** 181.19 g/mol, **m.p.:** >225 °C (decomposition). **TLC:** R_f = 0.10 (CH_2Cl_2/MeOH 5:1), ninhydrin stain. **^{1}H NMR** (600 MHz, D_2O): δ [ppm] = 7.51 (m, 5H, Ar), 4.40 (d, *J* 11.8 Hz, 1H, CH_2), 4.29 (d, *J* 11.8 Hz, 1H, CH_2). **^{13}C NMR** (150 MHz, D_2O): δ [ppm] = 173.2 (s, CO_2H), 134.4 (s, Ar), 129.2 (d, Ar), 129.1 (d, Ar), 125.7 (d, Ar), 67.5 (s, αC), 63.4 (t, CH_2). **FT-IR (ATR):** $\tilde{\nu}$ [cm^{-1}] = 3335 (br w), 2473 (br w), 1615 (vs), 1451 (m), 1392 (s), 1356 (m), 1062 (m), 851 (w), 696 (s). **HR-MS:** (ESI) = *m*/*z* calcd. for: $C_9H_{12}NO_3$ $[M+H]^+$ 182.0812 u, found: 182.0804 u. **$[a]_\lambda^T$:** (c = 0.60 g/100 mL, H_2O) = $[a]_D^{20}$: –26.5°.
Data consistent with literature.[2]

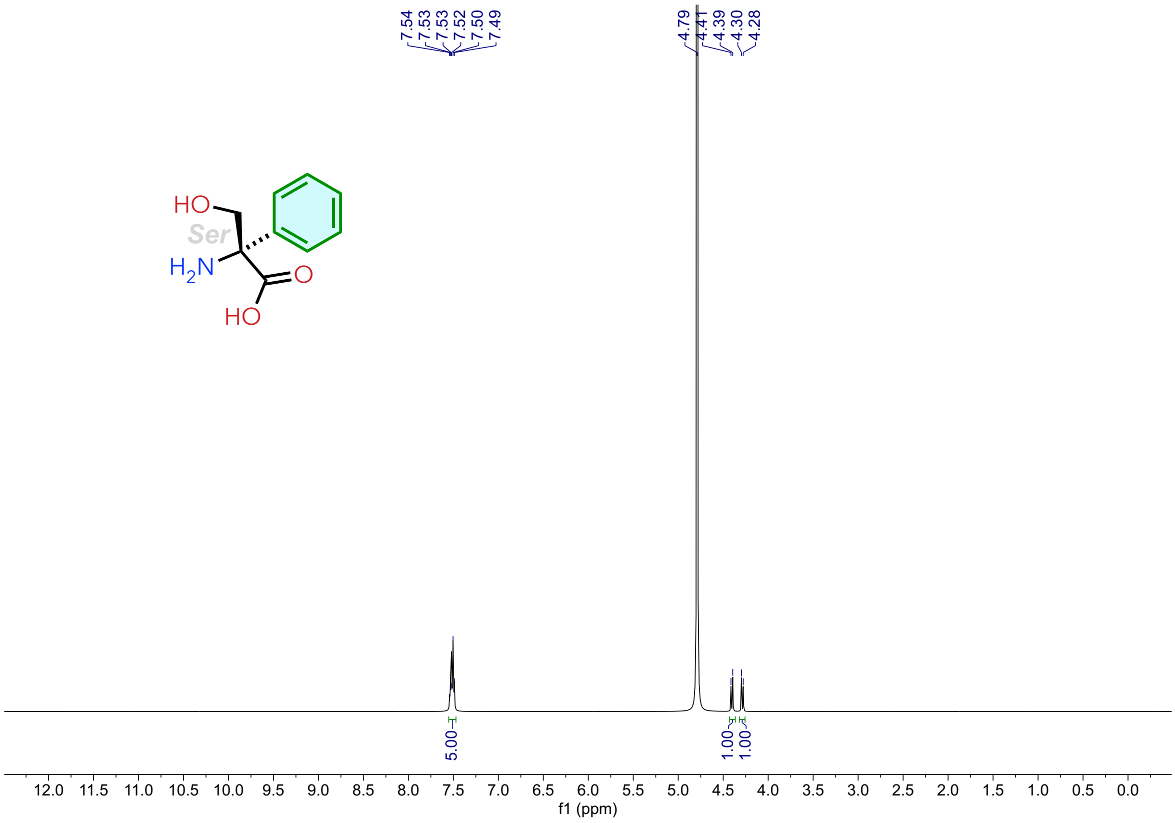
7.54
7.53
7.53
7.52
7.50
7.49
4.79
4.41
4.39
4.30
4.28
HO
Ser
H_2N
O
HO
5.00
1.00
1.00
12.0 11.5 11.0 10.5 10.0 9.5 9.0 8.5 8.0 7.5 7.0 6.5 6.0 5.5 5.0 4.5 4.0 3.5 3.0 2.5 2.0 1.5 1.0 0.5 0.0
f1 (ppm)

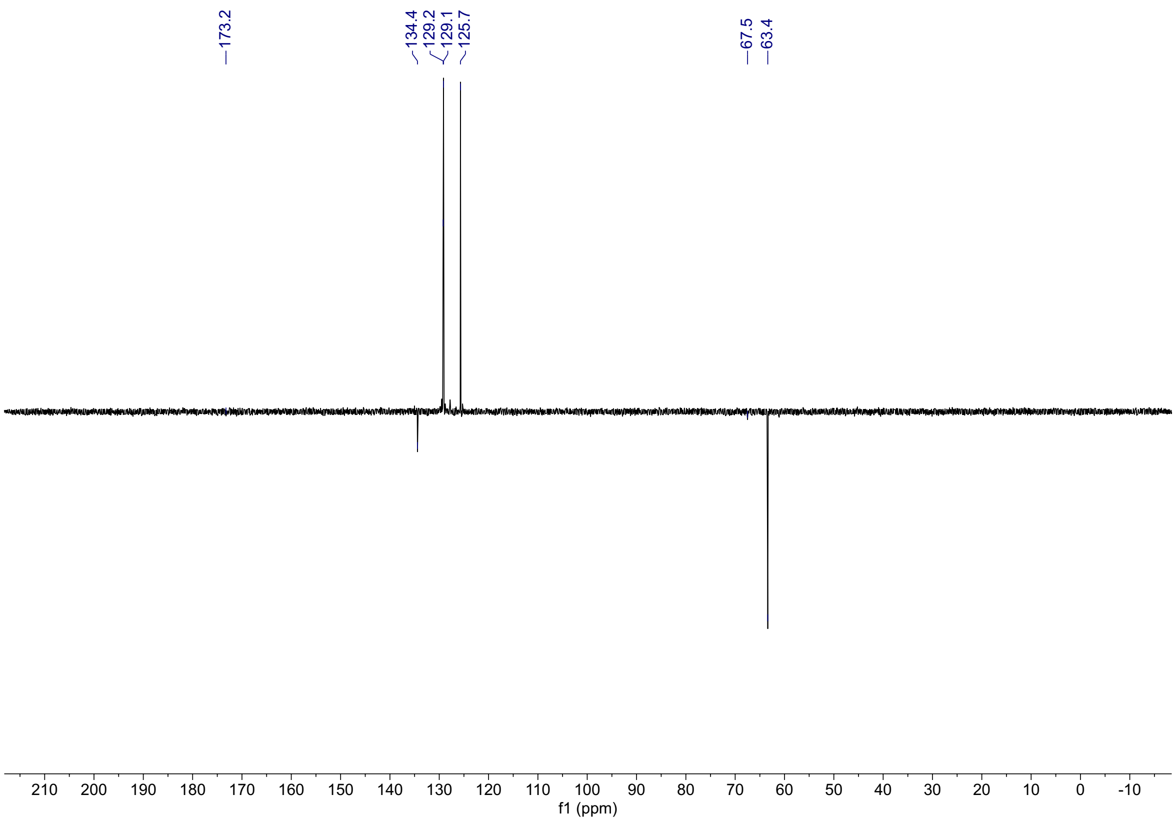
173.2
134.4
129.2
129.1
125.7
67.5
63.4
210 200 190 180 170 160 150 140 130 120 110 100 90 80 70 60 50 40 30 20 10 0 -10
f1 (ppm)

(*S*)-2-((((9*H*-fluoren-9-yl)methoxy)carbonyl)amino)-2-phenyl-3-(trityloxy)propanoic acid (32)[3]

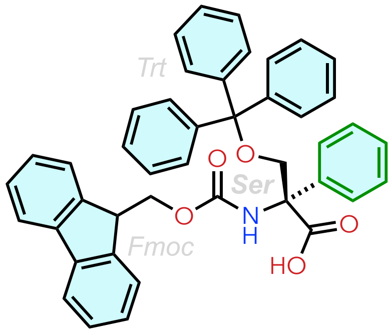

32

Following **GP6**, crude α-phenylserine **31** (obtained after basic hydrolysis starting with 50 mg of **27a**) and sodium carbonate (241 mg, 2.27 mmol, 10.0 equiv.) were suspended in a dioxane/water (4 mL, 1:1 v/v) at 0 °C. Fmoc-OSu (155 mg, 0.46 mmol, 2.0 equiv.) was added dropwise over 5 minutes. The reaction mixture was allowed to warm to ambient temperature and stirred overnight. The next day, the reaction mixture was diluted with H_2O and washed with Et_2O. The aqueous layer was acidified to pH 4 using 3 M aqueous HCl and extracted with EtOAc. The combined organic layers were dried over $MgSO_4$ and concentrated under reduced pressure, affording Fmoc-protected α-phenylserine as a white solid. The crude carbamate was dissolved in CH_2Cl_2 (3 mL), and DIPEA (60 µL, 0.35 mmol, 1.5 equiv.) and TrtCl (128 mg, 0.46 mmol, 2.0 equiv.) were added. The reaction mixture was stirred at ambient temperature overnight. The reaction was quenched with brine and extracted with EtOAc. The combined organic layers were dried over $MgSO_4$, and the solvent was removed under reduced pressure. After purification by silica gel column chromatography (PE/EA, gradient elution), the title compound **32** was obtained as a white solid (74 mg, 0.12 mmol, 50% over 3 steps).

Formula: $C_{43}H_{35}NO_5$, **MW:** 645.76 g/mol, **m.p.:** 195 – 197 °C. **TLC:** R_f = 0.54 (PE/EA 1:2), $KMnO_4$ stain. **^{1}H NMR** (600 MHz, CD_3OD): δ [ppm] = 7.80 (d, *J* 7.5 Hz, 2H, Ar), 7.64 (d, *J* 7.4 Hz, 2H, Ar), 7.48 – 7.09 (m, 24H, Ar), 4.19 (m, 2H, $CH_{2a}OH$, FmocCH), 4.15 – 4.09 (m, 2H, $FmocCH_2$), 4.03 (d, *J* 8.3 Hz, 1H, $CH_{2b}OH$). **^{13}C NMR** (150 MHz, CD_3OD): δ [ppm] = 174.6 (s, CO_2H), 156.0 (s, Fmoc carbonyl), 138.6 (s, Ar), 145.3 (s, FmocAr), 145.3 (s, FmocAr), 145.2 (s, TrtAr), 142.6 (s, Ar), 142.5 (s, FmocAr), 142.5 (s, FmocAr), 130.1 (d, Ar), 130.0 (d, 6Ar), 130.0 (d, Ar), 129.3 (d, Ar), 129.2 (d, Ar), 128.8 (d, 2Ar), 128.7 (d, 6Ar), 128.6 (d, Ar), 128.3 (d, Ar), 128.2 (d, Ar), 128.1 (d, Ar), 127.3 (d, Ar), 126.4 (d, Ar), 126.4 (d, Ar), 120.9 (d, 2Ar), 120.9 (d, Ar), 87.8 (s, TrtC), 68.1 (t, $FmocCH_2$), 64.6 (t, CH_2OH), 48.2 (d, FmocCH). **HR-MS:** (ESI) = *m/z* calcd. for: $C_{43}H_{35}NO_5Na$ $[M+Na]^+$ 668.2407 u, found: 668.2415 u. Data consistent with literature.[3]

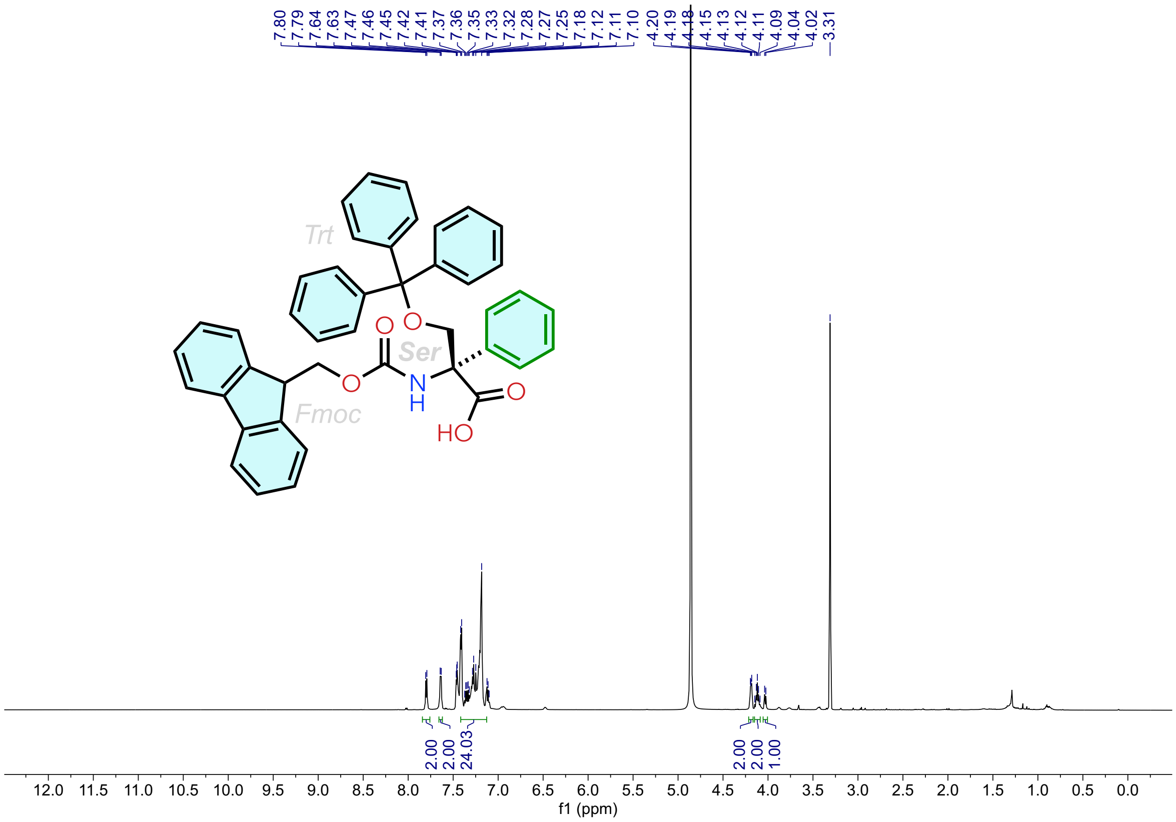
7.80 7.79 7.64 7.63 7.47 7.46 7.45 7.42 7.41 7.37 7.36 7.35 7.33 7.32 7.28 7.27 7.25 7.18 7.12 7.11 7.10 4.20 4.19 4.18 4.15 4.13 4.12 4.11 4.09 4.04 4.02 3.31
Trt
Ser
Fmoc
O
N
H
HO
2.00 2.00 24.03 2.00 2.00 1.00
f1 (ppm)

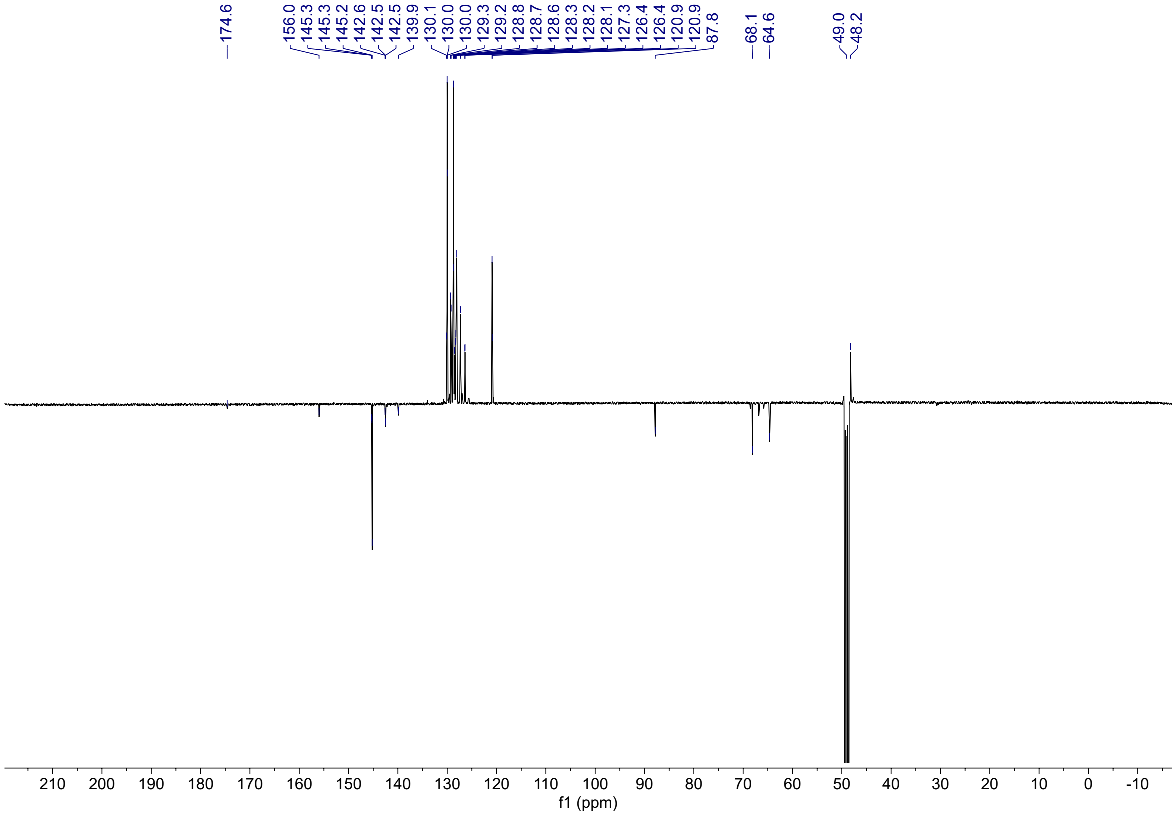
174.6 156.0 145.3 145.2 142.6 142.5 142.5 139.9 130.1 130.0 130.0 129.3 129.2 128.8 128.7 128.6 128.3 128.2 128.1 127.3 126.4 120.9 120.9 87.8 68.1 64.6 49.0 48.2
f1 (ppm)

(*S*)-2,3-diamino-2-phenylpropanoic acid (33)

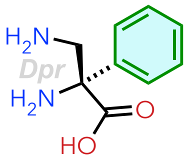

33

Following **GP6**, hydantoin **29a** (50 mg, 0.23 mmol, 1.0 equiv.) was dissolved in a water/dioxane mixture (0.6 mL, 5:1), and 0.5 mL of a 2 M aqueous sodium hydroxide solution was added. The title compound **33** was obtained as a white solid (36 mg, 0.20 mmol, 85%) after purification by silica gel column chromatography (CH_2Cl_2/MeOH, gradient elution).

Formula: $C_9H_{12}N_2O_2$, **MW:** 180.21 g/mol, **m.p.:** >225 °C (decomposition). **TLC:** R_f = 0.10 (CH_2Cl_2/MeOH 1:1), ninhydrin stain. **^{1}H NMR** (600 MHz, D_2O): δ [ppm] = 7.66 (m, 5H, Ar), 3.97 (d, *J* 13.2 Hz, 1H, CH_2), 3.88 (d, *J* 13.2 Hz, 1H, CH_2). **^{13}C NMR** (150 MHz, D_2O): δ [ppm] = 172.1 (s, CO_2H), 132.1 (s, Ar), 130.7 (d, Ar), 130.0 (d, Ar), 126.8 (d, Ar), 62.4 (s, αC), 42.9 (t, CH_2). **FT-IR (ATR):** $\tilde{\nu}$ [cm^{-1}] = 3282 (br w), 1771 (m), 1701 (vs), 1448 (m), 1393 (m), 1307 (w), 1280 (w), 1099 (w), 1016 (w), 976 (w). **HR-MS:** (ESI) = *m*/*z* calcd. for: $C_9H_{13}N_2O_2$ $[M+H]^+$ 181.0972 u, found: 181.0964. **$[a]_\lambda^T$:** (c = 0.55 g/100 mL, H_2O) = $[a]_D^{20}$: –24.5°.

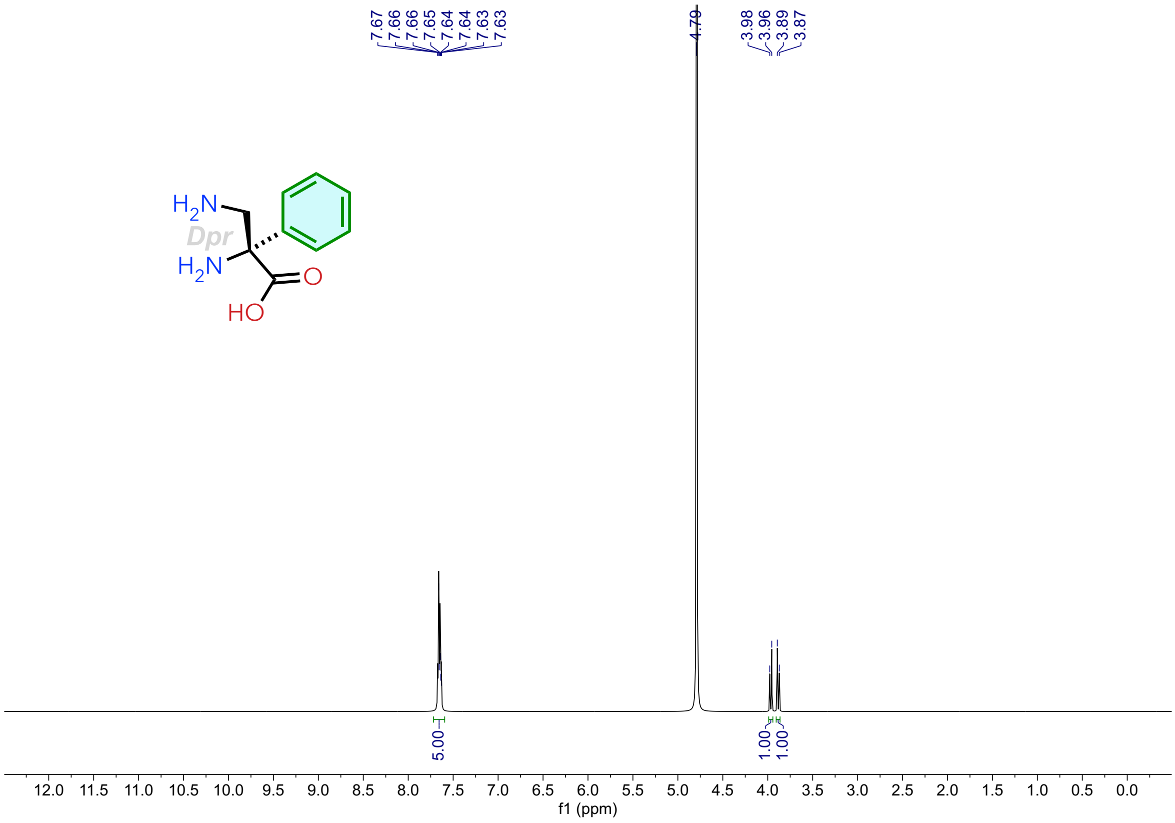
7.67
7.66
7.66
7.65
7.64
7.64
7.63
7.63
4.79
3.98
3.96
3.89
3.87
H2N
Dpr
H2N
O
HO
5.00
1.00
1.00
12.0 11.5 11.0 10.5 10.0 9.5 9.0 8.5 8.0 7.5 7.0 6.5 6.0 5.5 5.0 4.5 4.0 3.5 3.0 2.5 2.0 1.5 1.0 0.5 0.0
f1 (ppm)

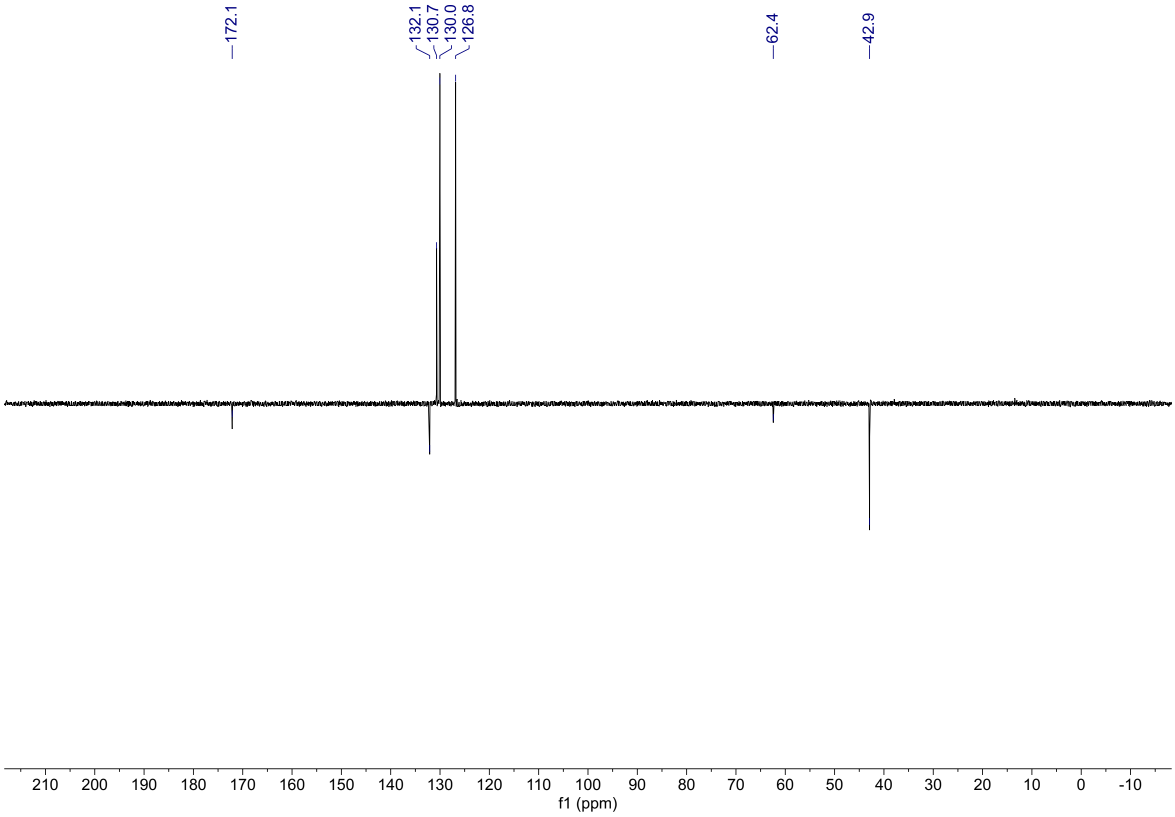
172.1
132.1
130.7
130.0
126.8
62.4
42.9
210 200 190 180 170 160 150 140 130 120 110 100 90 80 70 60 50 40 30 20 10 0 -10
f1 (ppm)

5. In situ IR Spectroscopy

Spectra were recorded using a Mettler Toledo ReactIR 15 spectrometer equipped with a DST series AgX fiber conduit and an integrated DiComp probe. Following **GP4**, to a solution of **4a** (100 mg, 0.31 mmol, 1.0 equiv.) in dry THF (3.1 mL, 0.1 M) was added to a three-neck Schlenk tube equipped with a stirrer bar, ReactIR probe and nitrogen inlet; the remaining neck was used for the addition of reagents. The solution was cooled to −78 °C and allowed to stabilise for at least 20 minutes prior to analysis. *In situ* IR analysis was initiated and the cooled KHMDS (0.47 mL, 0.47 mmol, 1 M in THF, 1.5 equiv.) was added rapidly in a single portion to the solution and immediate disappearance of the amide and urea carbonyl peaks (at ca. 1771 and 1667 cm^{-1}) was observed, along with formation of two peaks of **5a** at ca. 1784 and 1724 cm^{-1}, respectively.

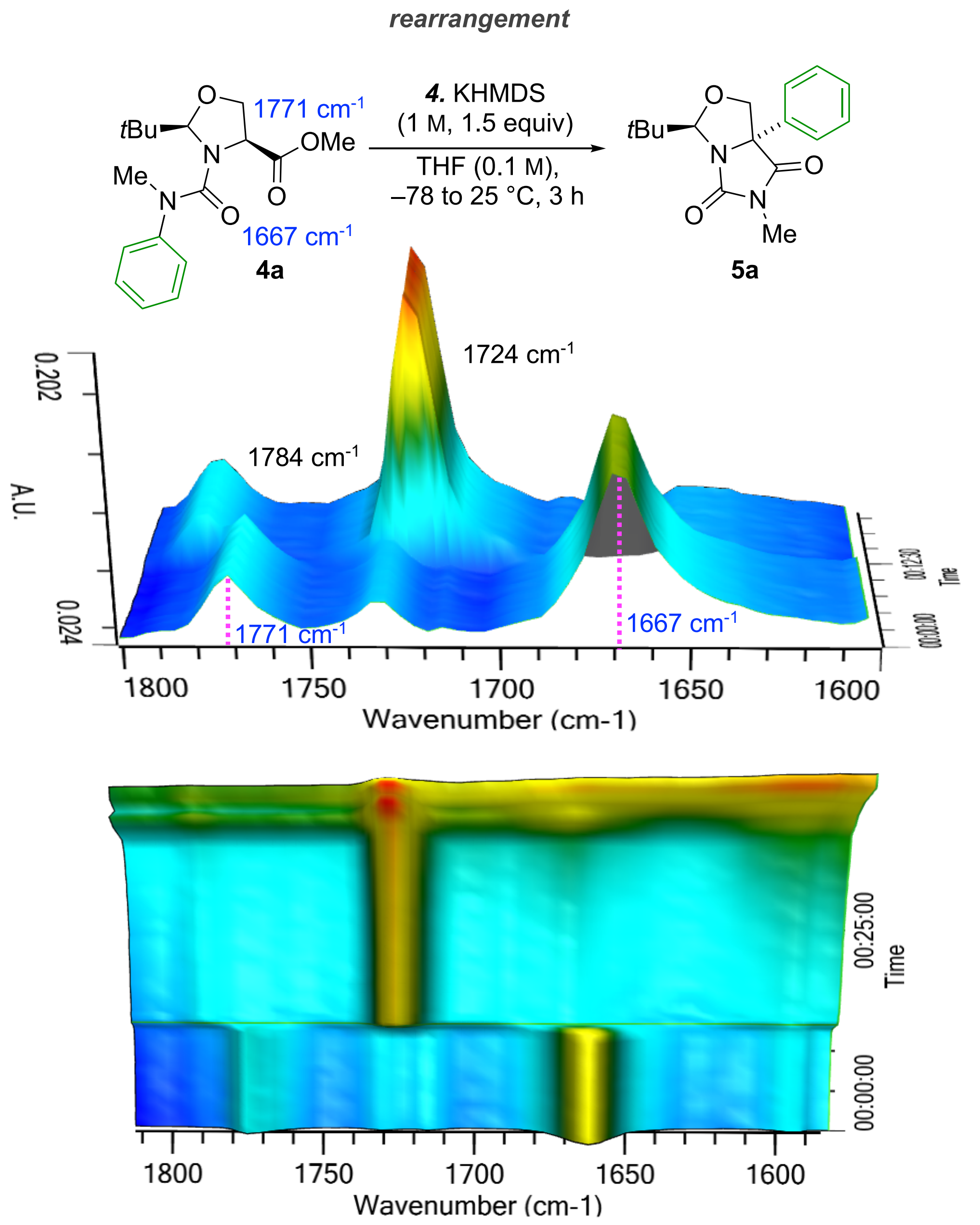

6. X-ray Crystallographic Data

X-ray diffraction experiments were all carried out at 100(2) K with the exception of **TAS_A50** which was collected at 200(2) K, data for **TAS_A133**, **TAS_A176** and **TAS_A35** were collected on a Bruker APEX II diffractometer using Mo-K_{α} radiation (λ = 0.71073 Å), while **CG38**, **NPI_M38**, **TAS_A7**, **TAS_A42_A43**, **TAS_A44**, **TAS_A116** and **TAS_A219** were collected on a Bruker D8 Venture using Mo-K_{α} radiation (λ = 0.71073 Å) while data for **TAS_A23**, **TAS_A48**, **TAS_A50**, **TAS_A109**, **TAS_A159**, **TAS_A202** were collected on a Bruker D8 Venture using Cu-K_{α} (λ = 1.54178 Å) radiation. Intensities were integrated in SAINT[4] and absorption corrections based on equivalent reflections were applied using SADABS.[5] Structures **CG38** and **TAS_A35** were solved using ShelXS[6], while **NPI_M38**, **TAS_A7**, **TAS_A23**, **TAS_A42_A43**, **TAS_A44**, **TAS_A48**, **TAS_A50**, **TAS_A109**, **TAS_A116**, **TAS_A133**, **TAS_A159**, **TAS_A176**, **TAS_A202**, **TAS_A219** were solved using ShelXT[7] all of the structures were refined by full matrix least squares against F^2 in ShelXL[6, 8] using Olex2[9]. All of the non-hydrogen atoms were refined anisotropically. While all of the hydrogen atoms were located geometrically and refined using a riding model. In the case of **NPI_M38** the molecule displayed disorder, the occupancies of the fragments were determined by refining them against a free variable with the sum of the two sites set to equal 1, restraints and constraints were used to maintain sensible geometries and thermal parameters. In the case of **TAS_A23** the structure displayed racemic twinning and the absolute structure was not able to be determined. For **TAS_A50**, **TAS_A109, TAS_A159**, **TAS_A176**, **TAS_A202** it was also not possible to reliably determined the absolute structure. Crystal structure and refinement data are given in the following section. Crystallographic data for compounds **3**, **4a/c**, **5a/c/n/o/q**, **23a/l/m**, **24a**, and **27a/b/e** have been deposited with the Cambridge Crystallographic Data Centre as supplementary publication CCDC 2385406-2385411, 2385413, 2385415-2385416, 2385418-2385424. Copies of the data can be obtained free of charge on application to CCDC, 12 Union Road, Cambridge CB2 1EZ, UK [fax(+44) 1223 336033, e-mail: deposit@ccdc.cam.ac.uk].

6.1 X-ray Crystallographic Data and Structure Refinement of 3 (CCDC 2385406)

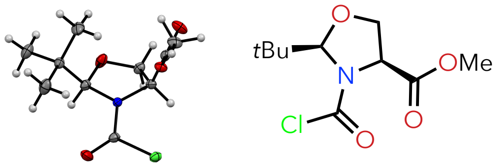

Identification code	**TAS_A7**
Empirical formula	$C_{10}H_{16}NO_4Cl$
Formula weight	249.69
Temperature/K	100(2)
Crystal system	orthorhombic
Space group	$P2_12_12_1$
a/Å	8.1845(3)
b/Å	11.8291(4)
c/Å	12.4218(4)
α/°	90
β/°	90
γ/°	90
Volume/Å^3	1202.62(7)
Z	4
ρ_{calc}g/cm^3	1.379
μ/mm^{-1}	0.317
F(000)	528.0
Crystal size/mm^3	0.46 × 0.34 × 0.16
Radiation	MoKα (λ = 0.71073)
2θ range for data collection/°	4.756 to 57.424
Index ranges	-11 ≤ h ≤ 10, -15 ≤ k ≤ 15, -16 ≤ l ≤ 16
Reflections collected	22948
Independent reflections	3096 [R_{int} = 0.0374, R_{sigma} = 0.0292]
Data/restraints/parameters	3096/0/149
Goodness-of-fit on F^2	1.039
Final R indexes [I>=2σ (I)]	R_1 = 0.0286, wR_2 = 0.0579
Final R indexes [all data]	R_1 = 0.0340, wR_2 = 0.0596
Largest diff. peak/hole / e Å^{-3}	0.24/-0.22
Flack parameter	-0.037(18)
CCDC number	2385406

6.2 X-ray Crystallographic Data and Structure Refinement of 15 (CCDC 2385421)

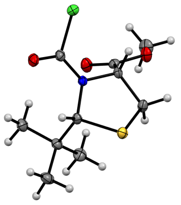

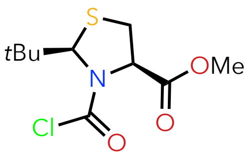

Identification code	**TAS_A219**
Empirical formula	$C_{10}H_{16}NO_3SCl$
Formula weight	265.75
Temperature/K	100(2)
Crystal system	orthorhombic
Space group	$P2_12_12_1$
a/Å	7.5398(3)
b/Å	15.0171(6)
c/Å	22.1606(10)
α/°	90
β/°	90
γ/°	90
Volume/Å^3	2509.15(18)
Z	8
ρ_{calc}g/cm^3	1.407
μ/mm^{-1}	0.463
F(000)	1120.0
Crystal size/mm^3	0.51 × 0.43 × 0.16
Radiation	MoKα (λ = 0.71073)
2θ range for data collection/°	3.676 to 55.89
Index ranges	-9 ≤ h ≤ 9, -19 ≤ k ≤ 19, -29 ≤ l ≤ 27
Reflections collected	74222
Independent reflections	5999 [R_{int} = 0.0614, R_{sigma} = 0.0298]
Data/restraints/parameters	5999/18/310
Goodness-of-fit on F^2	1.038
Final R indexes [I>=2σ (I)]	R_1 = 0.0265, wR_2 = 0.0579
Final R indexes [all data]	R_1 = 0.0303, wR_2 = 0.0591
Largest diff. peak/hole / e Å^{-3}	0.21/-0.24
Flack parameter	0.01(2)
CCDC number	2385421

6.3 X-ray Crystallographic Data and Structure Refinement of 4a (CCDC 2385407)

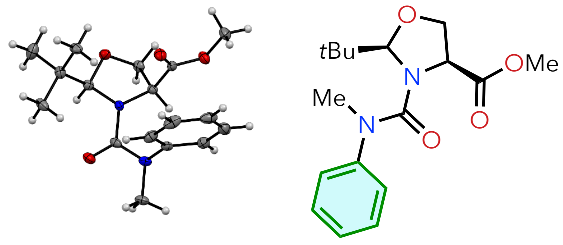

Identification code	**TAS_A23**
Empirical formula	$C_{17}H_{24}N_2O_4$
Formula weight	320.38
Temperature/K	100(2)
Crystal system	monoclinic
Space group	$P2_1$
a/Å	9.6944(4)
b/Å	17.3138(7)
c/Å	20.3159(9)
α/°	90
β/°	90.653(2)
γ/°	90
Volume/Å^3	3409.7(2)
Z	8
ρ_{calc}g/cm^3	1.248
μ/mm^{-1}	0.729
F(000)	1376.0
Crystal size/mm^3	0.33 × 0.15 × 0.10
Radiation	CuKα (λ = 1.54178)
2θ range for data collection/°	4.35 to 159.286
Index ranges	-12 ≤ h ≤ 11, -22 ≤ k ≤ 21, -25 ≤ l ≤ 25
Reflections collected	81361
Independent reflections	14581 [R_{int} = 0.0841, R_{sigma} = 0.0548]
Data/restraints/parameters	14581/1/850
Goodness-of-fit on F^2	1.056
Final R indexes [I>=2σ (I)]	R_1 = 0.0596, wR_2 = 0.1661
Final R indexes [all data]	R_1 = 0.0679, wR_2 = 0.1728
Largest diff. peak/hole / e Å^{-3}	0.26/-0.28
Flack parameter	undetermined
CCDC number	2385407

6.4 X-ray Crystallographic Data and Structure Refinement of 4c (CCDC 2385408)

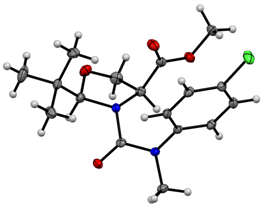

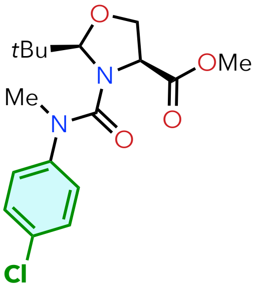

Identification code	**TAS_A35**
Empirical formula	$C_{17}H_{23}ClN_2O_4$
Formula weight	354.82
Temperature/K	100(2)
Crystal system	orthorhombic
Space group	$P2_12_12_1$
a/Å	11.2203(2)
b/Å	11.5068(2)
c/Å	13.9430(3)
α/°	90
β/°	90
γ/°	90
Volume/Å^3	1800.18(6)
Z	4
ρ_{calc}g/cm^3	1.309
μ/mm^{-1}	0.235
F(000)	752.0
Crystal size/mm^3	0.71 × 0.37 × 0.31
Radiation	MoKα (λ = 0.71073)
2θ range for data collection/°	4.59 to 55.958
Index ranges	-14 ≤ h ≤ 14, -15 ≤ k ≤ 15, -18 ≤ l ≤ 18
Reflections collected	16206
Independent reflections	4326 [R_{int} = 0.0410, R_{sigma} = 0.0400]
Data/restraints/parameters	4326/0/222
Goodness-of-fit on F^2	1.044
Final R indexes [I>=2σ (I)]	R_1 = 0.0332, wR_2 = 0.0719
Final R indexes [all data]	R_1 = 0.0398, wR_2 = 0.0752
Largest diff. peak/hole / e Å^{-3}	0.20/-0.20
Flack parameter	-0.04(3)
CCDC number	2385408

6.5 X-ray Crystallographic Data and Structure Refinement of 5a (CCDC 2385413)

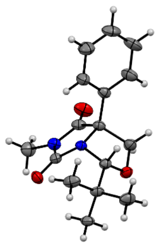

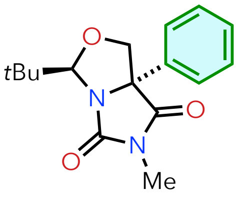

Identification code	**TAS_A50**
Empirical formula	$C_{16}H_{20}N_2O_3$
Formula weight	288.34
Temperature/K	200(2)
Crystal system	monoclinic
Space group	$P2_1$
a/Å	6.13708(8)
b/Å	8.61648(12)
c/Å	14.5004(2)
α/°	90
β/°	97.3376(7)
γ/°	90
Volume/Å^3	760.501(18)
Z	2
ϱ_{calc}g/cm^3	1.259
μ/mm^{-1}	0.713
F(000)	308.0
Crystal size/mm^3	0.47 × 0.39 × 0.29
Radiation	CuKα (λ = 1.54178)
2θ range for data collection/°	6.146 to 136.448
Index ranges	$-7 \leq h \leq 7$, $-10 \leq k \leq 10$, $-15 \leq l \leq 17$
Reflections collected	24068
Independent reflections	2773 [R_{int} = 0.0417, R_{sigma} = 0.0239]
Data/restraints/parameters	2773/1/195
Goodness-of-fit on F^2	1.146
Final R indexes [I>=2σ (I)]	R_1 = 0.0449, wR_2 = 0.1196
Final R indexes [all data]	R_1 = 0.0452, wR_2 = 0.1204
Largest diff. peak/hole / e Å^{-3}	0.25/-0.27
Flack parameter	undetermined
CCDC number	2385413

6.6 X-ray Crystallographic Data and Structure Refinement of 5c (CCDC 2385409)

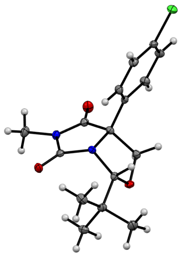

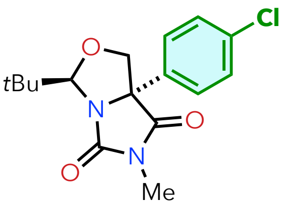

Identification code	**TAS_A42_43**
Empirical formula	$C_{16}H_{19}N_2O_3Cl$
Formula weight	322.78
Temperature/K	100(2)
Crystal system	orthorhombic
Space group	$P2_12_12_1$
a/Å	7.8016(5)
b/Å	11.1570(7)
c/Å	18.2200(11)
α/°	90
β/°	90
γ/°	90
Volume/Å^3	1585.91(17)
Z	4
ρ_{calc}g/cm^3	1.352
μ/mm^{-1}	0.255
F(000)	680.0
Crystal size/mm^3	0.59 × 0.43 × 0.28
Radiation	MoKα (λ = 0.71073)
2θ range for data collection/°	4.28 to 55.72
Index ranges	-10 ≤ h ≤ 10, -14 ≤ k ≤ 14, -23 ≤ l ≤ 23
Reflections collected	21524
Independent reflections	3762 [R_{int} = 0.0269, R_{sigma} = 0.0214]
Data/restraints/parameters	3762/0/203
Goodness-of-fit on F^2	1.055
Final R indexes [I>=2σ (I)]	R_1 = 0.0247, wR_2 = 0.0595
Final R indexes [all data]	R_1 = 0.0257, wR_2 = 0.0600
Largest diff. peak/hole / e Å^{-3}	0.20/-0.15
Flack parameter	0.003(14)
CCDC number	2385409

6.7 X-ray Crystallographic Data and Structure Refinement of 5n (CCDC 2385418)

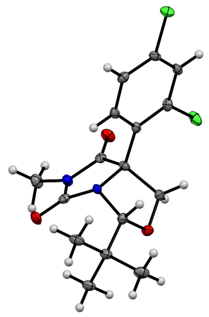

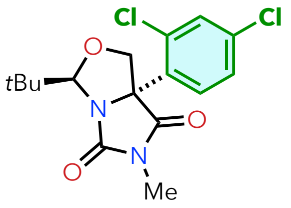

Identification code	**TAS_A116**
Empirical formula	$C_{16}H_{18}N_2O_3Cl_2$
Formula weight	357.22
Temperature/K	100(2)
Crystal system	orthorhombic
Space group	$P2_12_12_1$
a/Å	10.0630(4)
b/Å	11.7020(5)
c/Å	13.7495(5)
α/°	90
β/°	90
γ/°	90
Volume/Å^3	1619.10(11)
Z	4
ρ_{calc}g/cm^3	1.465
μ/mm^{-1}	0.417
F(000)	744.0
Crystal size/mm^3	0.39 × 0.23 × 0.11
Radiation	MoKα (λ = 0.71073)
2θ range for data collection/°	4.57 to 54.24
Index ranges	$-12 \leq h \leq 12$, $-15 \leq k \leq 14$, $-17 \leq l \leq 17$
Reflections collected	42303
Independent reflections	3574 [R_{int} = 0.0834, R_{sigma} = 0.0551]
Data/restraints/parameters	3574/0/212
Goodness-of-fit on F^2	1.030
Final R indexes [I>=2σ (I)]	R_1 = 0.0365, wR_2 = 0.0658
Final R indexes [all data]	R_1 = 0.0585, wR_2 = 0.0717
Largest diff. peak/hole / e Å^{-3}	0.22/-0.27
Flack parameter	-0.03(3)
CCDC number	2385418

6.8 X-ray Crystallographic Data and Structure Refinement of 5o (CCDC 2385424)

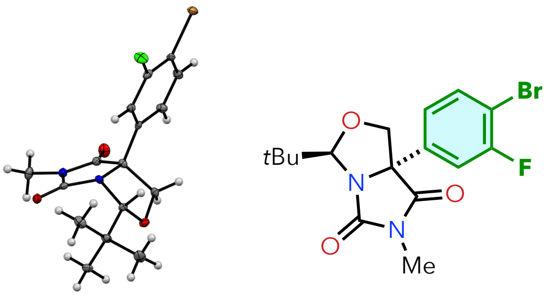

Identification code	**TAS_A133**
Empirical formula	$C_{16}H_{18}N_2O_3FBr$
Formula weight	385.23
Temperature/K	100(2)
Crystal system	orthorhombic
Space group	$P2_12_12_1$
a/Å	7.8561(2)
b/Å	11.2132(3)
c/Å	18.4399(4)
α/°	90
β/°	90
γ/°	90
Volume/Å^3	1624.41(7)
Z	4
ρ_{calc}g/cm^3	1.575
μ/mm^{-1}	2.557
F(000)	784.0
Crystal size/mm^3	0.39 × 0.36 × 0.28
Radiation	MoKα (λ = 0.71073)
2θ range for data collection/°	5.72 to 55.942
Index ranges	-10 ≤ h ≤ 10, -14 ≤ k ≤ 14, -24 ≤ l ≤ 24
Reflections collected	22531
Independent reflections	3909 [R_{int} = 0.0487, R_{sigma} = 0.0345]
Data/restraints/parameters	3909/0/212
Goodness-of-fit on F^2	1.039
Final R indexes [I>=2σ (I)]	R_1 = 0.0246, wR_2 = 0.0513
Final R indexes [all data]	R_1 = 0.0290, wR_2 = 0.0525
Largest diff. peak/hole / e Å^{-3}	0.27/-0.28
Flack parameter	0.005(4)
CCDC number	2385424

6.9 X-ray Crystallographic Data and Structure Refinement of 5q (CCDC 2385419)

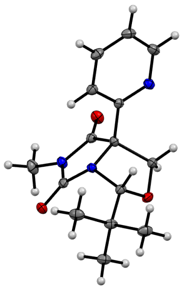

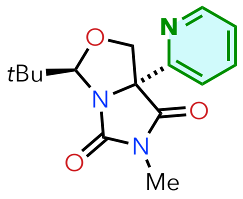

Identification code	**TAS_A159**
Empirical formula	$C_{15}H_{19}N_3O_3$
Formula weight	289.33
Temperature/K	100(2)
Crystal system	orthorhombic
Space group	$P2_12_12_1$
a/Å	10.0248(2)
b/Å	10.3656(2)
c/Å	14.0719(3)
α/°	90
β/°	90
γ/°	90
Volume/Å^3	1462.25(5)
Z	4
ρ_{calc}g/cm^3	1.314
μ/mm^{-1}	0.764
F(000)	616.0
Crystal size/mm^3	0.40 × 0.15 × 0.06
Radiation	CuKα (λ = 1.54178)
2θ range for data collection/°	10.6 to 150.354
Index ranges	-11 ≤ h ≤ 12, -12 ≤ k ≤ 12, -17 ≤ l ≤ 17
Reflections collected	29292
Independent reflections	3011 [R_{int} = 0.0443, R_{sigma} = 0.0204]
Data/restraints/parameters	3011/0/194
Goodness-of-fit on F^2	1.034
Final R indexes [I>=2σ (I)]	R_1 = 0.0287, wR_2 = 0.0715
Final R indexes [all data]	R_1 = 0.0299, wR_2 = 0.0725
Largest diff. peak/hole / e Å^{-3}	0.17/-0.25
Flack parameter	undetermined
CCDC number	2385419

6.10 X-ray Crystallographic Data and Structure Refinement of 23a (CCDC 2385415)

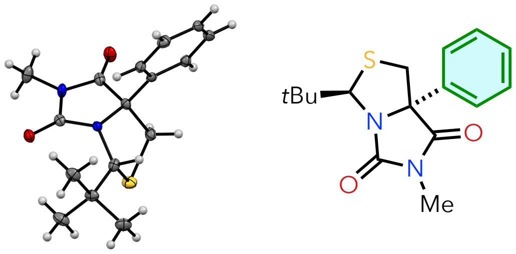

Identification code	**CG38**
Empirical formula	$C_{16}H_{20}N_2O_2S$
Formula weight	304.40
Temperature/K	100(2)
Crystal system	orthorhombic
Space group	$P2_12_12_1$
a/Å	10.3162(4)
b/Å	10.6719(4)
c/Å	14.1946(5)
α/°	90
β/°	90
γ/°	90
Volume/Å^3	1562.73(10)
Z	4
ϱ_{calc}g/cm^3	1.294
μ/mm^{-1}	0.213
F(000)	648.0
Crystal size/mm^3	0.32 × 0.25 × 0.23
Radiation	MoKα (λ = 0.71073)
2θ range for data collection/°	4.776 to 55.774
Index ranges	$-13 \leq h \leq 13$, $-14 \leq k \leq 14$, $-18 \leq l \leq 18$
Reflections collected	41796
Independent reflections	3725 [R_{int} = 0.0906, R_{sigma} = 0.0606]
Data/restraints/parameters	3725/0/194
Goodness-of-fit on F^2	1.031
Final R indexes [I>=2σ (I)]	R_1 = 0.0459, wR_2 = 0.1003
Final R indexes [all data]	R_1 = 0.0696, wR_2 = 0.1093
Largest diff. peak/hole / e Å^{-3}	0.92/-0.33
Flack parameter	0.04(4)
CCDC number	2385415

6.11 X-ray Crystallographic Data and Structure Refinement of 23l (CCDC 2385423)

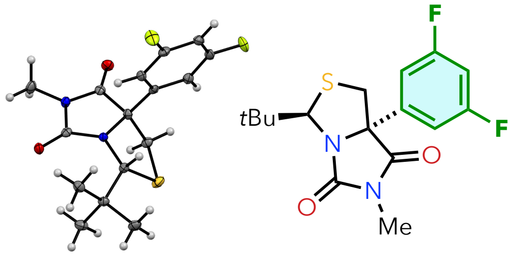

Identification code	**TAS_A176**
Empirical formula	$C_{16}H_{18}N_2O_2F_2S$
Formula weight	340.38
Temperature/K	100(2)
Crystal system	orthorhombic
Space group	$P2_12_12_1$
a/Å	8.8771(2)
b/Å	12.1239(3)
c/Å	14.7560(4)
α/°	90
β/°	90
γ/°	90
Volume/Å^3	1588.12(7)
Z	4
ρ_{calc}g/cm^3	1.424
μ/mm^{-1}	0.235
F(000)	712.0
Crystal size/mm^3	0.37 × 0.24 × 0.19
Radiation	MoKα (λ = 0.71073)
2θ range for data collection/°	4.348 to 55.89
Index ranges	$-11 \leq h \leq 11$, $-15 \leq k \leq 15$, $-19 \leq l \leq 19$
Reflections collected	21676
Independent reflections	3804 [R_{int} = 0.0524, R_{sigma} = 0.0362]
Data/restraints/parameters	3804/0/212
Goodness-of-fit on F^2	1.050
Final R indexes [I>=2σ (I)]	R_1 = 0.0316, wR_2 = 0.0728
Final R indexes [all data]	R_1 = 0.0358, wR_2 = 0.0752
Largest diff. peak/hole / e Å^{-3}	0.25/-0.22
Flack parameter	undetermined
CCDC number	2385423

6.12 X-ray Crystallographic Data and Structure Refinement of 23m (CCDC 2385422)

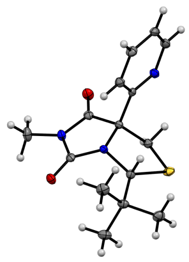

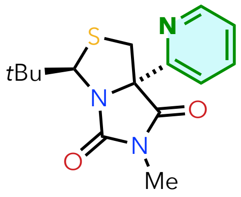

Identification code	**NPI_M38**
Empirical formula	$C_{15}H_{19}N_3O_2S$
Formula weight	305.39
Temperature/K	100(2)
Crystal system	orthorhombic
Space group	$P2_12_12_1$
a/Å	10.3712(4)
b/Å	10.5056(4)
c/Å	13.9365(6)
α/°	90
β/°	90
γ/°	90
Volume/Å^3	1518.46(11)
Z	4
ρ_{calc}g/cm^3	1.336
μ/mm^{-1}	0.221
F(000)	648.0
Crystal size/mm^3	0.50 × 0.45 × 0.38
Radiation	MoKα (λ = 0.71073)
2θ range for data collection/°	4.856 to 55.85
Index ranges	-13 ≤ h ≤ 13, -13 ≤ k ≤ 13, -18 ≤ l ≤ 18
Reflections collected	46005
Independent reflections	3632 [R_{int} = 0.0594, R_{sigma} = 0.0282]
Data/restraints/parameters	3632/2/198
Goodness-of-fit on F^2	1.056
Final R indexes [I>=2σ (I)]	R_1 = 0.0301, wR_2 = 0.0705
Final R indexes [all data]	R_1 = 0.0336, wR_2 = 0.0722
Largest diff. peak/hole / e Å^{-3}	0.29/-0.31
Flack parameter	-0.03(3)
CCDC number	2385422

6.13 X-ray Crystallographic Data and Structure Refinement of 24a (CCDC 2385420)

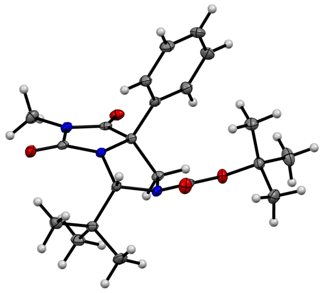

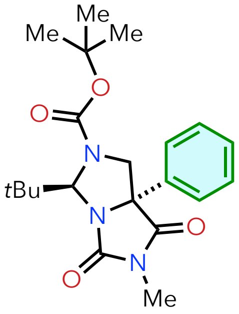

Identification code	**TAS_A202**
Empirical formula	$C_{21}H_{29}N_3O_4$
Formula weight	387.47
Temperature/K	100(2)
Crystal system	orthorhombic
Space group	$P2_12_12_1$
a/Å	6.29970(10)
b/Å	15.4749(3)
c/Å	21.2616(4)
α/°	90
β/°	90
γ/°	90
Volume/Å^3	2072.73(6)
Z	4
ρ_{calc}g/cm^3	1.242
μ/mm^{-1}	0.703
F(000)	832.0
Crystal size/mm^3	0.52 × 0.24 × 0.20
Radiation	CuKα (λ = 1.54178)
2θ range for data collection/°	7.064 to 136.478
Index ranges	-7 ≤ h ≤ 7, -18 ≤ k ≤ 18, -25 ≤ l ≤ 25
Reflections collected	39880
Independent reflections	3802 [R_{int} = 0.0542, R_{sigma} = 0.0249]
Data/restraints/parameters	3802/0/260
Goodness-of-fit on F^2	1.066
Final R indexes [I>=2σ (I)]	R_1 = 0.0320, wR_2 = 0.0856
Final R indexes [all data]	R_1 = 0.0328, wR_2 = 0.0864
Largest diff. peak/hole / e Å^{-3}	0.17/-0.23
Flack parameter	undetermined
CCDC number	2385420

6.14 X-ray Crystallographic Data and Structure Refinement of 27a (CCDC 2385411)

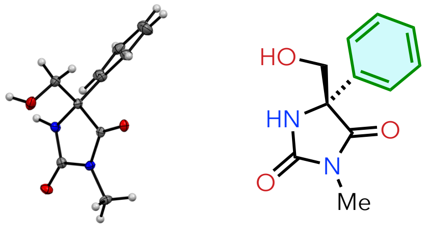

Identification code	**TAS_A48**
Empirical formula	$C_{11}H_{12}N_2O_3$
Formula weight	220.23
Temperature/K	100(2)
Crystal system	orthorhombic
Space group	$P2_12_12_1$
a/Å	8.7442(3)
b/Å	10.2577(3)
c/Å	11.5787(4)
α/°	90
β/°	90
γ/°	90
Volume/Å^3	1038.56(6)
Z	4
ρ_{calc}g/cm^3	1.408
μ/mm^{-1}	0.868
F(000)	464.0
Crystal size/mm^3	0.34 × 0.23 × 0.21
Radiation	CuKα (λ = 1.54178)
2θ range for data collection/°	11.524 to 144.972
Index ranges	$-10 \le h \le 10$, $-12 \le k \le 12$, $-13 \le l \le 14$
Reflections collected	16685
Independent reflections	2039 [R_{int} = 0.0474, R_{sigma} = 0.0281]
Data/restraints/parameters	2039/0/154
Goodness-of-fit on F^2	1.179
Final R indexes [I>=2σ (I)]	R_1 = 0.0367, wR_2 = 0.0917
Final R indexes [all data]	R_1 = 0.0367, wR_2 = 0.0917
Largest diff. peak/hole / e Å^{-3}	0.30/-0.40
Flack parameter	0.00(8)
CCDC number	2385411

6.15 X-ray Crystallographic Data and Structure Refinement of 27b (CCDC 2385410)

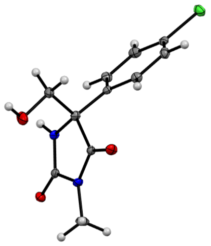

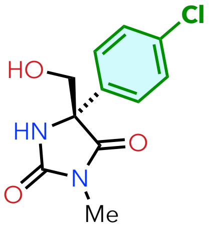

Identification code	**TAS_A44**
Empirical formula	$C_{11}H_{11}ClN_2O_3$
Formula weight	254.67
Temperature/K	100(2)
Crystal system	monoclinic
Space group	$P2_1$
a/Å	6.1431(3)
b/Å	26.6893(12)
c/Å	6.9816(3)
α/°	90
β/°	91.431(2)
γ/°	90
Volume/Å^3	1144.31(9)
Z	4
ρ_{calc}g/cm^3	1.478
μ/mm^{-1}	0.331
F(000)	528.0
Crystal size/mm^3	0.40 × 0.12 × 0.08
Radiation	MoKα (λ = 0.71073)
2θ range for data collection/°	3.052 to 54.964
Index ranges	-7 ≤ h ≤ 7, -34 ≤ k ≤ 34, -9 ≤ l ≤ 9
Reflections collected	29718
Independent reflections	5205 [R_{int} = 0.0405, R_{sigma} = 0.0354]
Data/restraints/parameters	5205/1/321
Goodness-of-fit on F^2	1.034
Final R indexes [I>=2σ (I)]	R_1 = 0.0321, wR_2 = 0.0717
Final R indexes [all data]	R_1 = 0.0381, wR_2 = 0.0747
Largest diff. peak/hole / e Å^{-3}	0.24/-0.23
Flack parameter	-0.002(19)
CCDC number	2385410

6.16 X-ray Crystallographic Data and Structure Refinement of 27e (CCDC 2385416)

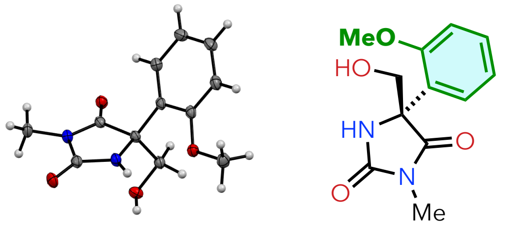

Identification code	**TAS_A109**
Empirical formula	$C_{12}H_{14}N_2O_4$
Formula weight	250.25
Temperature/K	100(2)
Crystal system	orthorhombic
Space group	$P2_12_12_1$
a/Å	7.4308(2)
b/Å	8.5849(2)
c/Å	18.9087(5)
α/°	90
β/°	90
γ/°	90
Volume/Å^3	1206.24(5)
Z	4
ρ_{calc}g/cm^3	1.378
μ/mm^{-1}	0.879
F(000)	528.0
Crystal size/mm^3	0.38 × 0.28 × 0.14
Radiation	CuKα (λ = 1.54178)
2θ range for data collection/°	9.354 to 144.228
Index ranges	$-9 \le h \le 9$, $-10 \le k \le 10$, $-23 \le l \le 23$
Reflections collected	43261
Independent reflections	2379 [R_{int} = 0.1302, R_{sigma} = 0.0370]
Data/restraints/parameters	2379/0/173
Goodness-of-fit on F^2	1.107
Final R indexes [I>=2σ (I)]	R_1 = 0.0517, wR_2 = 0.1454
Final R indexes [all data]	R_1 = 0.0517, wR_2 = 0.1454
Largest diff. peak/hole / e Å^{-3}	0.33/-0.34
Flack parameter	undetermined
CCDC number	2385416

7. Computational Studies

7.1 Computational Details

7.1.1 Geometry optimization Density functional theory (DFT) calculations were performed with ORCA 5.0.2.[10] The geometries were optimized using the hybrid B3LYP functional[11, 12] with D3(BJ) dispersion correction[13] and the minimally augmented triple-zeta *ma*-def2-TZVP basis set.[14] All DFT calculations were conducted with the *Defgrid2* integration grid, TightSCF convergence criteria, and Rijcosx approximation. Restricted KS was used throughout; unrestricted KS gave the same single point energies. Frequency calculations were performed at the same level of theory as geometry optimizations to verify the stationary points as minima (no imaginary frequencies); or transition states (one imaginary frequency) as well as to obtain thermal Gibbs free energy corrections at 298 or 195 K. Grimme's entropy corrections using quasi-rigid rotor harmonic approximation (qRRHO)[15] were applied to all frequencies below 100 cm^{-1}. Relaxed potential energy scans were done by RI-PBE-D3(BJ)/*ma*-def2-SVP.[16] Noncovalent interaction (NCI) plot was generated by NCIplot 4.0[17, 18] and visualized by VMD 1.9.3.[19]

7.1.2 Single-point calculations To refine the computed energy, single point calculations were performed in ORCA 5.0.2 at PBE0-D4/*ma*-def2-TZVP/SMD(THF)[20-22] level of theory using D4 dispersion correction,[21] Rijcosx approximation, and the corresponding auxiliary basis sets. TightSCF convergence criteria were used.

7.1.3 Gibbs free energies The ΔG value was obtained by adding the corresponding free energy corrections at 298 K calculated at the B3LYP-D3(BJ)/ma-def2-TZVP level, to ΔE, calculated at the single-point calculation at PBE0-D4/ma-def2-TZVP/SMD(THF) level of theory including solvation correction, if not stated otherwise.

7.2 Investigated Reactions

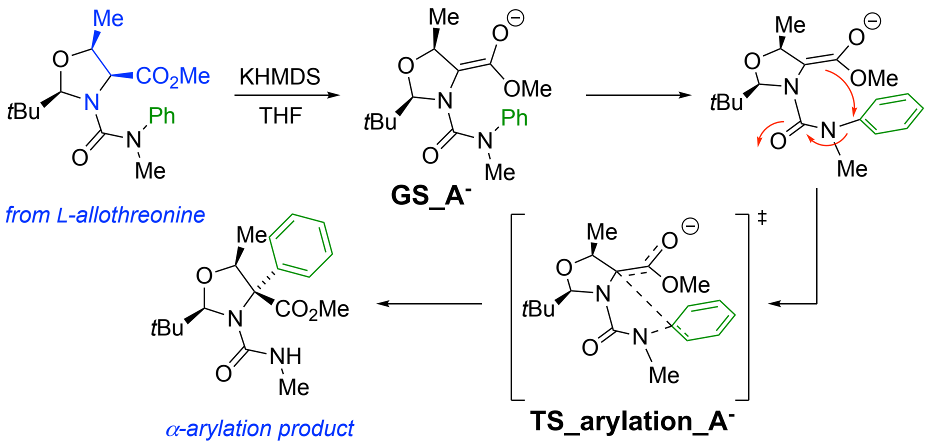

Figure S1. Computationally investigated **reaction A** of L-allothreonine to give the α-arylation product.

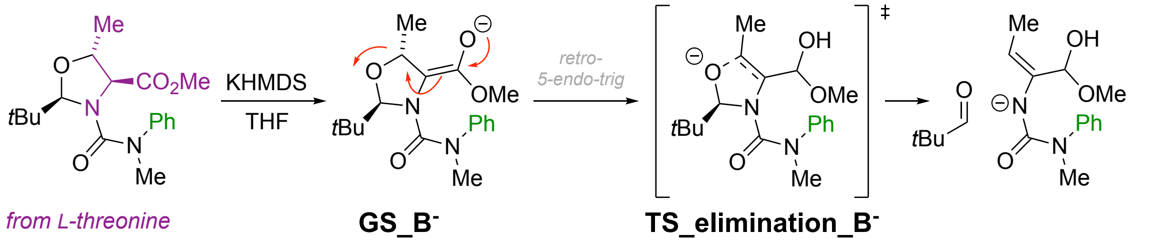

Figure S2. Computationally investigated **reaction B** of L-threonine, which proceeds to give a retro-5-endo-trig ring cyclisation-ring-opening.

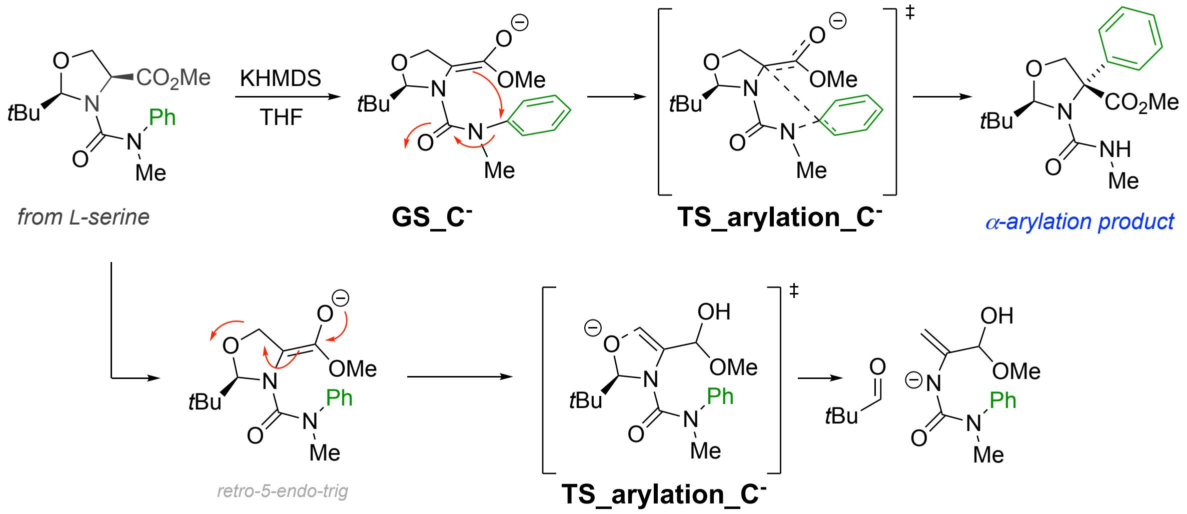

Figure S3. Computationally investigated **reaction C** of L-serine as a control.

7.3 Energy Profiles

Table S1. Comparison of reaction barriers of **reaction A** (via deprotonated L-allothreonine) and **reaction B** (via deprotonated L-threonine): as naked enolates (no countercation) of the ground state, the transition state of the α-arylation (**TS_arylation**), and ring-opening (**TS_elimination**). **Reaction C** (via deprotonated L-serine) as potassium enolate is included for comparison. The values are Δ*G*(sol) for anions (or neutral species) at PBE0-D4/ma-def2-TZVP//B3LYP-D3(BJ)/ma-def2-TZVP with SMD(THF) solvation correction calculated at 298 K and 195 K.

Structure	**Δ*G*(sol) at 298 K** [kJ·mol^{-1}]	**Δ*G*(sol) at 195 K** [kJ·mol^{-1}]
GS_**A**$^-$	0.0	0.0
TS_arylation_**A**$^-$	39.3	35.1
TS_elimination_**A**$^-$	58.6	58.9
GS_**B**$^-$	+0.3	+0.7
TS_arylation_**B**$^-$	46.5	45.5
TS_elimination_**B**$^-$	68.8	68.8
GS_ **C**$^-$_K$^+$•2Me$_2$O	0.0	n.a.
TS_arylation_ **C**$^-$_K$^+$•2Me$_2$O	90.2	n.a.
TS_elim_ **C**$^-$_K$^+$•2Me$_2$O	113.5	n.a.

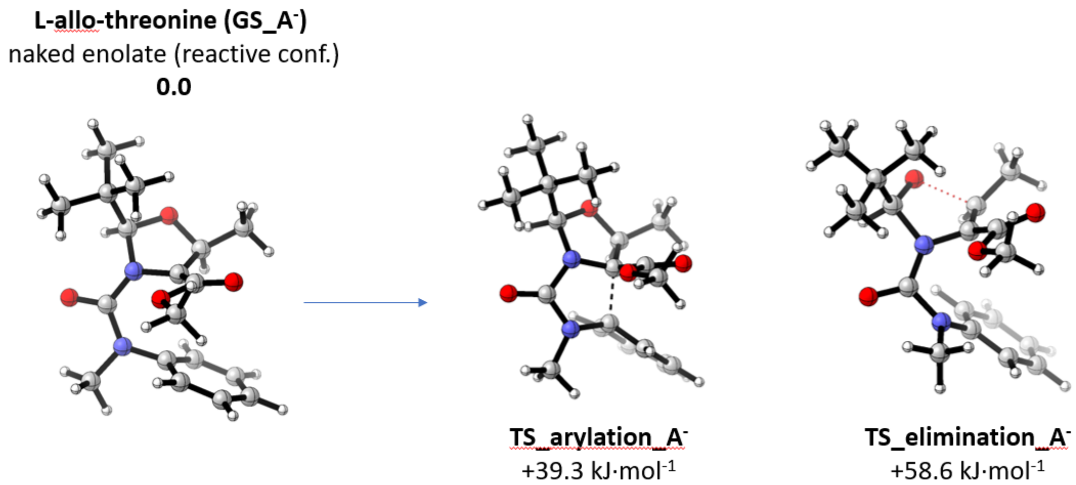

Figure S4. Reaction of deprotonated of L-allo-threonine **GS_A**$^-$ (naked anion; left) via **TS_arylation_A**$^-$ or **TS_elimination_A**$^-$. Lower barrier of **TS_arylation_A**$^-$ compared to the elimination favours the arylation process over the ring opening side reaction in agreement with the experiment.

Table S2. Comparison of reaction barriers of **reaction A**$^-$ (deprotonated L-allo-threonine), as naked enolate (no countercation); and in the presence of K$^+$ with two explicit dimethylether molecules. The values are Δ*G*(sol) for anions at PBE0-D4/ma-def2-TZVP//B3LYP-D3(BJ)/ma-def2-TZVP with SMD(THF) solvation correction calculated at 298 K.

Structure	**A**$^-$ [kJ·mol^{-1}]	**A$^-$·K$^+$·2Me$_2$O** [kJ·mol^{-1}]
**GS**	0.0	0.0
**TS_arylation**	39.3	85.0
**TS_elimination**	58.6	106.6

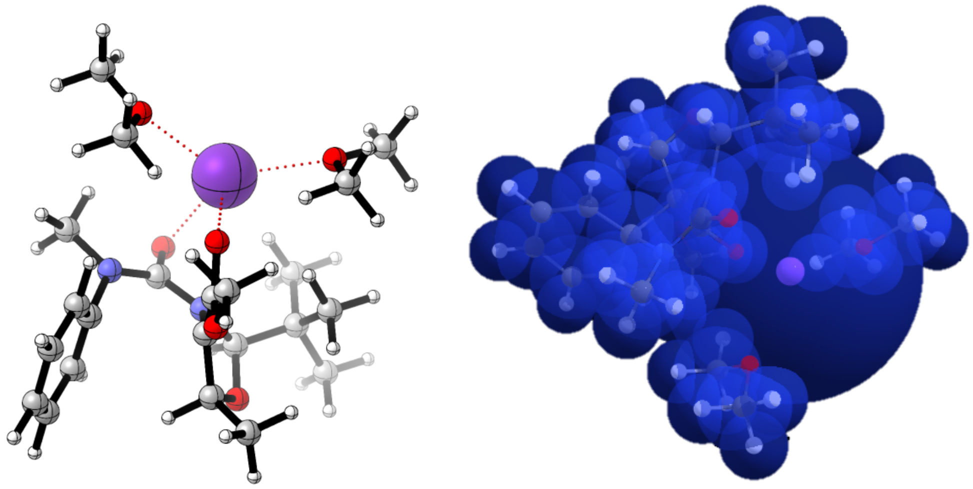

Figure S5. Computed structure of L-allothreonine **GS_ A^{-}·K^{+}·$2Me_2O$_conf8** (left) and the atomic van der Waals radii (right). The potassium cation is chelated by both ester enolate and urea oxygens, which stabilises the ground state and lowers its reactivity by raising the reaction barriers to both arylation and elimination.

Table S3. Comparison of reaction barriers of **reaction A^{-}** (deprotonated L-allo-threonine), as naked enolate (no countercation); in the presence of K^{+} with two explicit dimethylether molecules; and in the presence of two K^{+} cations with two explicit dimethylether molecules for each of the cations. The values are ΔG(sol) for anions at PBE0-D4/ma-def2-TZVP//B3LYP-D3(BJ)/ma-def2-TZVP with SMD(THF) solvation correction calculated at 298 K.

Structure	A^{-} [kJ·mol^{-1}]	A^{-}·K^{+}·$2Me_2O$ [kJ·mol^{-1}]	A^{-}·$2K^{+}$·$4Me_2O$ [kJ·mol^{-1}]
**GS**	0.0	0.0	0.0
**TS_arylation**	39.3	85.0	30.0
**TS_elimination**	58.6	106.6	66.10

The coordination of a single K^{+} cation by the enolate oxygen and urea stabilises the GS, thus leading to a higher arylation barrier. In the presence of two cations, the additional K^{+} polarises the aromatic ring, which favours the arylation TS even more than in the case of naked anion. In summary, either removal of K^{+} by a crown ether or excess K^{+} lower arylation barriers, which can then proceed at very low temperatures.

The computations based on the B3LYP-D3(BJ)/ma-def2-TZVP energies could provide the reactivity explanation: the arylation is preferential for the allo-threonine **A^{-}·$2K^{+}$·$4Me_2O$** due to lower energy barrier, while the elimination is preferred for the threonine system **B^{-}·$2K^{+}$·$4Me_2O$** due to K^{+} migration in the latter in the process leading up to the elimination transition state. This was not observed in the allo-threonine system **A^{-}·$2K^{+}$·$4Me_2O$.** The two ground states differ in energy by ~5 kJ/mol.

Table S4. Gibbs free energies of potassium enolates $\mathbf{X^{-}\cdot 2K^{+}\cdot 4Me_2O}$ (cationic species) at B3LYP-D3(BJ)/ma-def2-TZVP with Gibbs correction calculated at 298 K.

Structure	Δ*G*solv [kJ·mol^{-1}]
**GS_A-_2K^{+}**	-4.54
**TS_arylation_A-_2K+**	65.40
**TS_elimination_A-_2K+**	97.05
**GS_B-_2K^{+}**	0.00
**TS_arylation_B-_2K+**	79.22
**TS_elimination_B-_2K+**	32.57

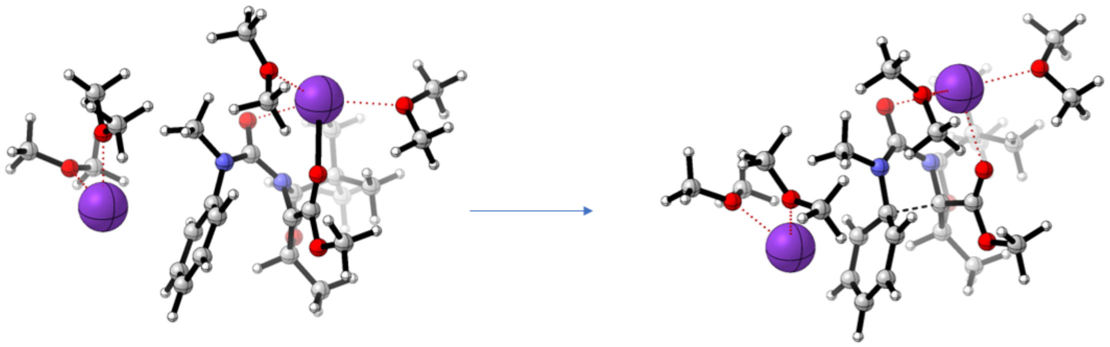

GS_A^{-}_2K^{+}_4Me$_2$O

TS_arylation_A^{-}_2K^{+}_4Me$_2$O

Figure S6. Ground state and TS_arylation of $\mathbf{GS\_A^{-}\cdot 2K^{+}\cdot 4Me_2O}$ including two potassium cations. One of them is chelated by the enolate oxygen and the other one polarises the aryl ring towards the C-C bond formation.

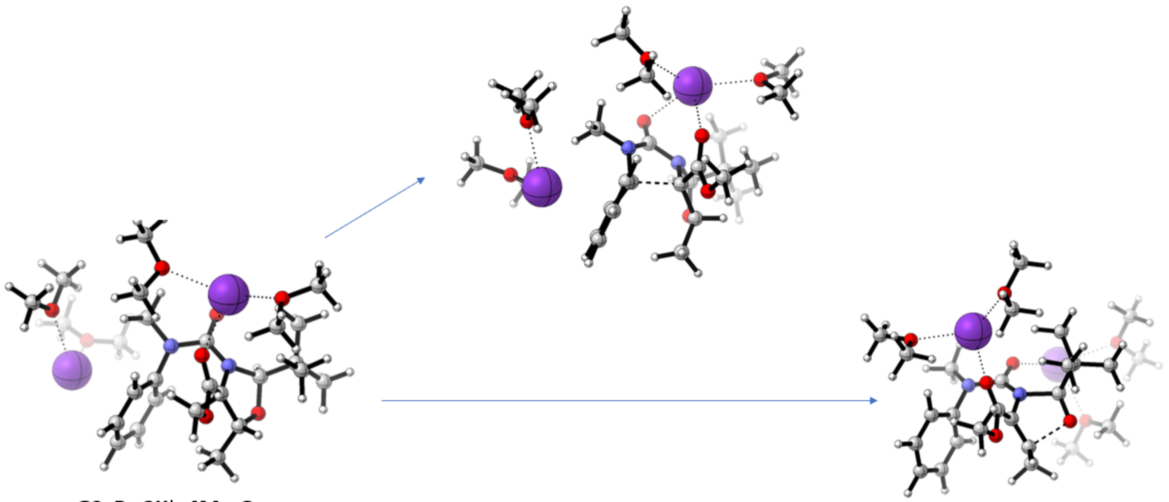

GS_B^{-}_2K^{+}_4Me$_2$O

TS_ B^{-}_ elimination_2K^{+}_4Me$_2$O

Figure S7. Ground state and TS_arylation and elimination of $\mathbf{GS\_B^{-}\cdot 2K^{+}\cdot 4Me_2O}$ including two potassium cations. The elimination has lower energy barrier due to potassium migration before the TS.

Table S5. Atomic distances of the two carbon atoms that are bound during the intramolecular C-C arylation reaction. This distance is shortened in the ground state with each added potassium cation (values highlighted in blue).

Structure	C-C (Å)
**GS_A$^-$**	2.97
**TS_arylation_A$^-$**	1.86
**GS_B$^-$**	2.89
**TS_arylation_B$^-$**	1.88
**GS_C$^-$·K$^+$·2Me$_2$O**	2.78
**TS_arylation_C$^-$·K$^+$·2Me$_2$O**	1.77
**GS_A$^-$·K$^+$·2Me$_2$O_conf8**	2.79
**TS_arylation_A$^-$·K$^+$·2Me$_2$O_conf8**	1.76
**GS_A$^-$·2K$^+$·4Me$_2$O**	2.67
**TS_arylation_A$^-$·2K$^+$·4Me$_2$O**	1.94

7.4 Computed Energies

Reaction A and B (naked enolate)

Table S6. Gibbs free energies of naked enolates **A**$^-$ and **B**$^-$ (**anionic species)** at PBE0-D4/ma-def2-TZVP/SMD(THF)//B3LYP-D3(BJ)/ma-def2-TZVP with Gibbs correction calculated at 298 K (correction at 195 K also given for comparison). The other potential conformations of both ground states (conf2) have high reaction barriers towards arylation and elimination.

Structure	B3LYP/ma-TZVP	$G_{corr\ 298K}$	$G_{corr\ 195K}$	PBE0-D4	ΔG_{corr}	ΔGsolv [kJ·mol^{-1}]
**GS_A-**	-1111.2911756	0.357180	0.379673	-1110.689209	-1110.332029	0
**TS_arylation_A-**	-1111.2698676	0.358607	0.379496	-1110.675669	-1110.317062	39.30
**TS_elimination_A-**	-1111.2736211	0.354511	0.377134	-1110.664230	-1110.309719	58.58
**GS_A-_conf2**	-1111.2925976	0.357779		-1110.688543	-1110.330764	3.32
**TS_arylation_A-_conf2**	-1111.2601916	0.358406		-1110.661740	-1110.303334	75.34
**TS_elimination_A-_conf2**	-1111.2639872	0.355365		-1110.655141	-1110.299776	84.68
**GS_B-**	-1111.2895968	0.356322	0.378982	-1110.688236	-1110.331914	0.30
**TS_arylation_B-**	-1111.2655445	0.357072	0.379179	-1110.671375	-1110.314303	46.54
**TS_elimination_B-**	-1111.2682138	0.354566	0.377050	-1110.660394	-1110.305828	68.79
**GS_B-_conf2**	-1111.2908971	0.356561		-1110.689163	-1110.332602	-1.51
**TS_arylation_B-_conf2**	-1111.2601916	0.358406		-1110.661740	-1110.303334	75.34
**TS_elimination_B-_conf2**	-1111.2639872	0.355365		-1110.655141	-1110.299776	84.68

Table 7. Gibbs free energies of enolate **C**$^-$_K^+·$2Me_2O$ (**neutral species)** and its transition states at PBE0-D4/ma-def2-TZVP/SMD(THF)//B3LYP-D3(BJ)/ma-def2-TZVP with Gibbs correction calculated at 298 K.

Structure	B3LYP/ma-TZVP	$G_{corr\ 298K}$	$G_{corr\ 195K}$	PBE0-D4	ΔG_{corr}	ΔGsolv [kJ·mol^{-1}]
**GS_C-**	-1981.912081	0.47779981		-1980.966352	-1980.488552	0.00
**TS_arylation_C-**	-1981.871069	0.48081119		-1980.935009	-1980.454197	90.20
**TS_elimination_C-**	-1981.871493	0.47843543		-1980.923749	-1980.445313	113.52

Reactions of A and B ($A^{-}{\cdot}K^{+}{\cdot}2Me_2O$)

Table S8. Gibbs free energies of potassium enolates **A-·K+·2Me2O** and **B-·K+·2Me2O (neutral species)** at PBE0-D4/ma-def2-TZVP/SMD(THF)//B3LYP-D3(BJ)/ma-def2-TZVP with Gibbs correction calculated at 298 K.

Structure	B3LYP/ma-TZVP	$G_{corr\ 298K}$	PBE0-D4	ΔG_{corr}	ΔGsolv [kJ·mol^{-1}]
**GS_A-_K+_conf8**	-2021.221089	0.504516	-2020.251624	-2019.747108	0.00
**TS_arylation_A-_K+_conf8**	-2021.180948	0.507498	-2020.222239	-2019.714741	84.98
**TS_elimination_A-_K+_conf8**	-2021.185199	0.505177	-2020.211696	-2019.706519	106.57
**product_arylation**	-2021.221214	0.504516	-2020.257754	-2019.753238	-16.09
**GS_A-_K+_conf3**	-2021.215198	0.505199	-2020.251601	-2019.746402	1.85
**TS_arylation_A-_K+_conf3**	-2021.175246	0.508431	-2020.220393	-2019.711962	92.27
**TS_elimination_A-_K+_conf3**	-2021.175632	0.505694	-2020.211493	-2019.705800	108.46
**GS_A-_K+_conf4**	-2021.188414	0.505038	-2020.234919	-2019.729881	45.23

Reactions of enolates with two potassium cations ($A/B^{-}{\cdot}2K^{+}{\cdot}4Me_2O$)

Table 9. Gibbs free energies of potassium enolates **$A^{-}{\cdot}2K^{+}{\cdot}4Me_2O$ (cationic species**) at PBE0-D4/ma-def2-TZVP/SMD(THF)//B3LYP-D3(BJ)/ma-def2-TZVP with Gibbs correction calculated at 298 K.

Structure	B3LYP/ma-TZVP	$G_{corr\ 298K}$	PBE0-D4	ΔG_{corr}	ΔGsolv [kJ·mol^{-1}]
**GS_A-_2K+**	-2931.019090	0.651963	-2929.766964	-2929.115002	0.00
**TS_arylation_A-_2K+**	-2930.996371	0.655885	-2929.759446	-2929.103561	30.04
**TS_elimination_A-_2K+**	-2930.981485	0.653052	-2929.742879	-2929.089828	66.10
**GS_B-_2K+**	-2931.016235	0.650838	-2929.779294	-2929.128457	-35.33
**TS_arylation_B-_2K+**	-2930.991411	0.656186	-2929.753613	-2929.097426	46.14
**TS_elimination_B-_2K+**	-2931.003162	0.650171	-2929.744490	-2929.094319	54.30

Table 10. Gibbs free energies of potassium enolates **$A^{-}{\cdot}2K^{+}{\cdot}4Me_2O$** (**cationic species)** at B3LYP-D3(BJ)/ma-def2-TZVP with Gibbs correction calculated at 298 K.

Structure	B3LYP/ma-TZVP	$G_{corr\ 298K}$	ΔG_{corr}	ΔGsolv [kJ·mol^{-1}]
**GS_A-_2K+**	-2931.019090	0.651963	-2930.367128	-4.54
**TS_arylation_A-_2K+**	-2930.996371	0.655885	-2930.340486	65.40
**TS_elimination_A-_2K+**	-2930.981485	0.653052	-2930.328434	97.05
**GS_B-_2K+**	-2931.016235	0.650838	-2930.365397	0.00
**TS_arylation_B-_2K+**	-2930.991411	0.656186	-2930.335224	79.22
**TS_elimination_B-_2K+**	-2931.003162	0.650171	-2930.352991	32.57

7.5 Coordinates of Computed Structures

All structures were optimised at B3LYP-D3(BJ)/ma-def2-TZVP level of theory.

7.5.1 Naked Enolates (Anionic; Charge -1)

GS_A-

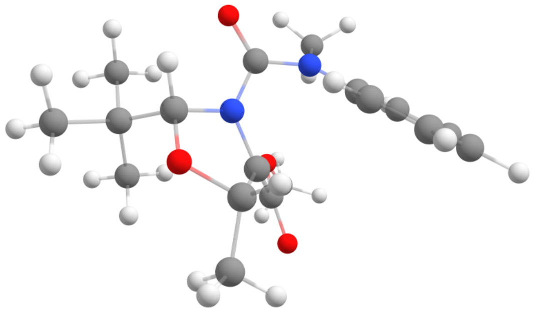

O	-0.193318000000	1.602002000000	-0.948833000000
C	0.228211000000	2.433922000000	0.195986000000
C	-1.304721000000	0.794512000000	-0.582771000000
C	-0.891118000000	2.291819000000	1.159031000000
C	0.567647000000	3.825255000000	-0.293409000000
H	1.157545000000	1.956146000000	0.551640000000
N	-1.459642000000	1.015628000000	0.857541000000
C	-1.526676000000	3.289886000000	1.875985000000
H	-1.065516000000	-0.260285000000	-0.748169000000
C	-2.544445000000	1.150509000000	-1.446908000000
C	-1.788634000000	-0.015338000000	1.671585000000
O	-2.305122000000	-1.056900000000	1.260798000000
N	-1.618831000000	0.180840000000	3.066184000000
C	-2.763410000000	-0.145992000000	3.892163000000
C	-0.480899000000	0.722516000000	3.645509000000
H	-2.441814000000	-0.650901000000	4.806242000000
H	-3.401521000000	-0.818988000000	3.325997000000
H	-3.333490000000	0.750120000000	4.161538000000
C	0.762746000000	0.642160000000	2.999828000000
C	-0.525235000000	1.309278000000	4.916516000000
H	-0.321076000000	4.338101000000	-0.659257000000
H	1.304521000000	3.759440000000	-1.098263000000
H	0.973564000000	4.415053000000	0.527627000000
C	-2.978500000000	2.605941000000	-1.249708000000
C	-3.716095000000	0.227628000000	-1.094415000000
C	-2.151710000000	0.924926000000	-2.913948000000
H	-3.269691000000	2.794396000000	-0.217330000000
H	-3.835973000000	2.819188000000	-1.896414000000
H	-2.177392000000	3.296954000000	-1.508380000000
H	-4.048203000000	0.387823000000	-0.069827000000
H	-3.440468000000	-0.823728000000	-1.183274000000
H	-4.554793000000	0.429225000000	-1.768273000000
H	-1.318969000000	1.569274000000	-3.195944000000
H	-3.000734000000	1.140653000000	-3.569128000000
H	-1.851907000000	-0.113592000000	-3.083408000000
O	-1.151635000000	4.451637000000	2.094548000000

O	-2.748572000000	2.852498000000	2.446754000000
C	-3.377745000000	3.801837000000	3.281574000000
H	-4.283941000000	3.321025000000	3.655685000000
H	-3.642819000000	4.713271000000	2.738636000000
H	-2.737509000000	4.088169000000	4.120771000000
C	0.630736000000	1.806331000000	5.509067000000
C	1.904566000000	1.143341000000	3.595017000000
C	1.853846000000	1.732169000000	4.858551000000
H	0.563078000000	2.270712000000	6.486099000000
H	2.749921000000	2.128797000000	5.318926000000
H	0.819932000000	0.177952000000	2.025571000000
H	2.846939000000	1.077857000000	3.063938000000
H	-1.470651000000	1.412904000000	5.428412000000

TS_arylation_A-

Imaginary frequency -184.04 cm^{-1}

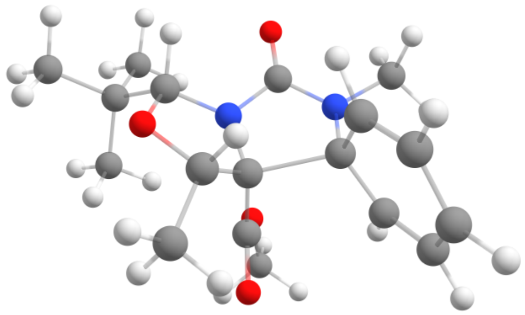

O	-0.391715000000	1.667560000000	-0.686987000000
C	0.139566000000	2.147478000000	0.572124000000
C	-1.533718000000	0.851739000000	-0.476690000000
C	-1.027924000000	2.028195000000	1.545118000000
C	0.806093000000	3.488848000000	0.351329000000
H	0.909503000000	1.429554000000	0.912915000000
N	-1.846645000000	1.009675000000	0.935217000000
C	-1.737197000000	3.249896000000	1.965149000000
H	-1.296771000000	-0.213310000000	-0.668548000000
C	-2.660444000000	1.278286000000	-1.453527000000
C	-2.130424000000	-0.063649000000	1.765223000000
O	-2.712179000000	-1.087088000000	1.401198000000
N	-1.682692000000	0.207474000000	3.027632000000
C	-1.821123000000	-0.793047000000	4.058772000000
C	-0.532601000000	1.160590000000	3.114040000000
H	-1.020063000000	-1.555004000000	4.015663000000
H	-2.789901000000	-1.297369000000	3.938498000000
H	-1.768831000000	-0.301517000000	5.041649000000
C	0.757663000000	0.478925000000	2.962809000000
C	-0.559944000000	2.041245000000	4.283844000000
H	0.094361000000	4.245828000000	-0.006456000000
H	1.609481000000	3.371366000000	-0.393963000000
H	1.239782000000	3.847310000000	1.295012000000
C	-3.063884000000	2.738044000000	-1.202586000000
C	-3.874908000000	0.362139000000	-1.243901000000
C	-2.128660000000	1.117248000000	-2.887674000000

H	-3.490106000000	2.854563000000	-0.196672000000
H	-3.821006000000	3.055835000000	-1.939561000000
H	-2.194344000000	3.406448000000	-1.290280000000
H	-4.266476000000	0.453959000000	-0.221076000000
H	-3.611082000000	-0.696904000000	-1.391883000000
H	-4.675273000000	0.626613000000	-1.956589000000
H	-1.264838000000	1.775982000000	-3.061894000000
H	-2.914780000000	1.367109000000	-3.620836000000
H	-1.809022000000	0.077991000000	-3.077811000000
O	-1.220944000000	4.318819000000	2.240845000000
O	-3.064994000000	3.028405000000	2.200850000000
C	-3.779047000000	4.102369000000	2.784712000000
H	-4.807372000000	3.743630000000	2.929974000000
H	-3.778170000000	4.988357000000	2.127817000000
H	-3.338425000000	4.392584000000	3.752008000000
C	0.587454000000	2.407578000000	4.960524000000
C	1.892596000000	0.862888000000	3.666069000000
C	1.853820000000	1.862919000000	4.651537000000
H	0.497467000000	3.129473000000	5.780464000000
H	2.749012000000	2.153861000000	5.205859000000
H	0.810312000000	-0.358968000000	2.259952000000
H	2.833960000000	0.337686000000	3.463948000000
H	-1.525089000000	2.464356000000	4.576241000000

TS_elimination_A-

Imaginary frequency -227.43 cm^{-1}

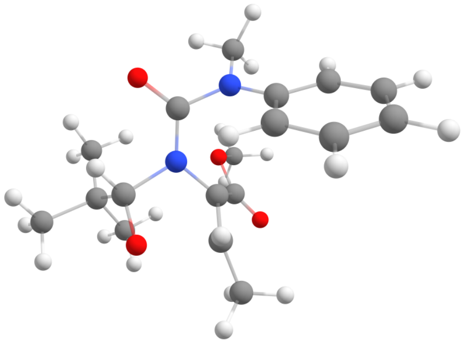

O	-0.199050000000	1.615146000000	-1.103044000000
C	0.240665000000	2.707774000000	0.562463000000
C	-1.222148000000	0.862124000000	-0.662614000000
C	-0.943575000000	2.386502000000	1.213013000000
C	0.673131000000	4.084615000000	0.190820000000
H	1.038568000000	1.982550000000	0.603644000000
N	-1.344826000000	1.073209000000	0.876270000000
C	-1.875777000000	3.338470000000	1.725018000000
H	-1.049927000000	-0.222030000000	-0.750028000000
C	-2.569853000000	1.177156000000	-1.406496000000
C	-1.661857000000	0.063526000000	1.701387000000
O	-2.127206000000	-1.015545000000	1.345990000000
N	-1.484828000000	0.320216000000	3.105337000000
C	-2.635120000000	0.069105000000	3.946053000000
C	-0.278577000000	0.690350000000	3.663235000000
H	-2.364374000000	-0.548490000000	4.807881000000

H	-3.371355000000	-0.467321000000	3.353907000000
H	-3.082667000000	1.002011000000	4.306878000000
C	0.929503000000	0.523748000000	2.962581000000
C	-0.211027000000	1.199633000000	4.970731000000
H	-0.170830000000	4.727861000000	-0.045025000000
H	1.349176000000	4.027847000000	-0.664430000000
H	1.213995000000	4.546009000000	1.024260000000
C	-2.841482000000	2.684344000000	-1.444313000000
C	-3.766621000000	0.453000000000	-0.785962000000
C	-2.368362000000	0.678648000000	-2.845128000000
H	-3.146427000000	3.060144000000	-0.469201000000
H	-3.648971000000	2.901618000000	-2.151618000000
H	-1.945077000000	3.218732000000	-1.757894000000
H	-3.965294000000	0.822641000000	0.220274000000
H	-3.590352000000	-0.620314000000	-0.713894000000
H	-4.662005000000	0.621833000000	-1.394867000000
H	-1.516812000000	1.182366000000	-3.304182000000
H	-3.262091000000	0.871077000000	-3.447778000000
H	-2.176091000000	-0.398383000000	-2.860279000000
O	-1.693120000000	4.534126000000	1.920698000000
O	-3.083636000000	2.757054000000	2.081326000000
C	-4.032931000000	3.641457000000	2.651693000000
H	-4.900273000000	3.029129000000	2.896893000000
H	-4.318786000000	4.427199000000	1.948632000000
H	-3.640931000000	4.118029000000	3.552952000000
C	1.010271000000	1.543945000000	5.537502000000
C	2.136517000000	0.875645000000	3.536583000000
C	2.195530000000	1.392509000000	4.830590000000
H	1.026912000000	1.946677000000	6.543861000000
H	3.144025000000	1.668431000000	5.273464000000
H	0.905857000000	0.116640000000	1.962040000000
H	3.047426000000	0.742007000000	2.964808000000
H	-1.118842000000	1.352952000000	5.536028000000

GS_A-_conf2

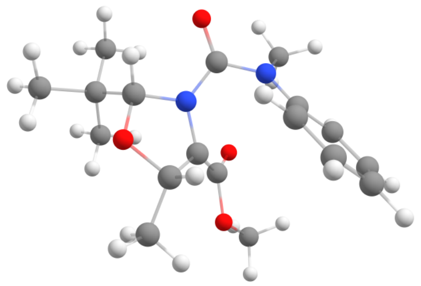

O	-0.166701000000	1.566818000000	-0.963651000000
C	0.256221000000	2.396714000000	0.177472000000
C	-1.293687000000	0.773767000000	-0.604854000000
C	-0.875085000000	2.270452000000	1.132701000000
C	0.626573000000	3.773149000000	-0.336092000000
H	1.175832000000	1.911448000000	0.548972000000
N	-1.454866000000	1.001639000000	0.828263000000

C	-1.520432000000	3.169176000000	1.958104000000
H	-1.066113000000	-0.283811000000	-0.768641000000
C	-2.523687000000	1.147068000000	-1.474735000000
C	-1.803566000000	-0.013712000000	1.656502000000
O	-2.325705000000	-1.055475000000	1.256181000000
N	-1.640037000000	0.209513000000	3.047043000000
C	-2.836784000000	0.030081000000	3.850221000000
C	-0.504331000000	0.775896000000	3.605460000000
H	-2.573161000000	-0.375512000000	4.829617000000
H	-3.475528000000	-0.684612000000	3.337083000000
H	-3.370566000000	0.977184000000	3.973348000000
C	0.751254000000	0.592320000000	3.006251000000
C	-0.563584000000	1.501338000000	4.802220000000
H	-0.254892000000	4.309069000000	-0.687706000000
H	1.336674000000	3.670954000000	-1.160589000000
H	1.082463000000	4.357217000000	0.462031000000
C	-2.952738000000	2.601346000000	-1.257748000000
C	-3.700011000000	0.223753000000	-1.138227000000
C	-2.125092000000	0.938174000000	-2.942336000000
H	-3.228956000000	2.781448000000	-0.219186000000
H	-3.818493000000	2.822449000000	-1.890599000000
H	-2.153680000000	3.292819000000	-1.522946000000
H	-4.037152000000	0.373496000000	-0.113949000000
H	-3.426332000000	-0.827401000000	-1.237546000000
H	-4.534302000000	0.434580000000	-1.814639000000
H	-1.289730000000	1.583846000000	-3.213574000000
H	-2.970749000000	1.163327000000	-3.598679000000
H	-1.826900000000	-0.098969000000	-3.122947000000
O	-2.575731000000	3.004395000000	2.595731000000
O	-0.820111000000	4.385249000000	2.105300000000
C	-1.306609000000	5.224881000000	3.130954000000
H	-0.690828000000	6.125810000000	3.100855000000
H	-1.213351000000	4.751772000000	4.113557000000
H	-2.356603000000	5.488609000000	2.980820000000
C	0.590672000000	2.036074000000	5.362488000000
C	1.892757000000	1.133950000000	3.567794000000
C	1.827056000000	1.863774000000	4.754181000000
H	0.512536000000	2.611277000000	6.278124000000
H	2.721847000000	2.290651000000	5.189756000000
H	0.816021000000	0.025575000000	2.087243000000
H	2.845013000000	0.989010000000	3.070981000000
H	-1.521072000000	1.698361000000	5.259828000000

GS_B-

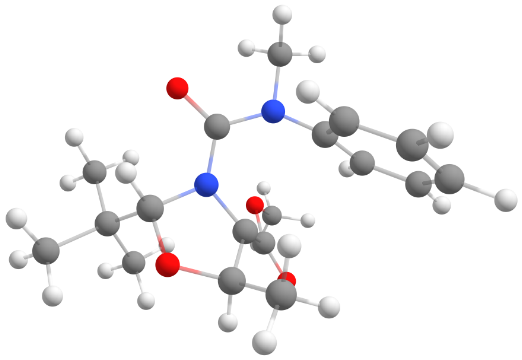

O	-0.262854000000	1.538643000000	-0.798895000000
C	0.018899000000	2.512310000000	0.259490000000
C	-1.442421000000	0.800869000000	-0.489807000000
C	-1.069878000000	2.295027000000	1.257359000000
N	-1.644747000000	1.025899000000	0.935774000000
C	-1.755131000000	3.347076000000	1.838458000000
H	-1.247791000000	-0.261365000000	-0.657017000000
C	-2.621764000000	1.220682000000	-1.406826000000
C	-1.969723000000	-0.009621000000	1.753818000000
O	-2.502633000000	-1.045416000000	1.341175000000
N	-1.763314000000	0.185434000000	3.125197000000
C	-2.381548000000	-0.798361000000	3.999240000000
C	-0.531378000000	0.717631000000	3.615000000000
H	-1.838893000000	-1.752789000000	4.018319000000
H	-3.395082000000	-0.995405000000	3.657721000000
H	-2.407878000000	-0.391585000000	5.011285000000
C	0.656957000000	0.025213000000	3.396720000000
C	-0.522301000000	1.861244000000	4.410141000000
C	-2.956872000000	2.709312000000	-1.266769000000
C	-3.861787000000	0.389385000000	-1.062191000000
C	-2.197638000000	0.926007000000	-2.852117000000
H	-3.322540000000	2.937979000000	-0.267271000000
H	-3.735866000000	2.976788000000	-1.988436000000
H	-2.085268000000	3.334122000000	-1.461900000000
H	-4.194650000000	0.591833000000	-0.044903000000
H	-3.658124000000	-0.680211000000	-1.128412000000
H	-4.675239000000	0.636164000000	-1.751876000000
H	-1.311477000000	1.501929000000	-3.120848000000
H	-3.005151000000	1.181895000000	-3.544367000000
H	-1.966245000000	-0.135313000000	-2.983449000000
O	-1.369168000000	4.522402000000	1.961747000000
O	-3.003347000000	2.975095000000	2.383503000000
C	-3.694068000000	4.015238000000	3.042880000000
H	-4.616976000000	3.572347000000	3.421501000000
H	-3.930846000000	4.841735000000	2.366774000000
H	-3.113481000000	4.426751000000	3.874124000000
C	0.665650000000	2.320968000000	4.956613000000
C	1.850715000000	0.490152000000	3.939507000000
C	1.858864000000	1.639154000000	4.719210000000
H	0.667858000000	3.230200000000	5.544940000000
H	2.788342000000	2.008542000000	5.135349000000

H	0.639198000000	-0.864683000000	2.779507000000
H	2.774255000000	-0.042528000000	3.745888000000
H	-1.449361000000	2.398932000000	4.551004000000
H	-0.055502000000	3.521061000000	-0.163584000000
C	1.452577000000	2.285884000000	0.721922000000
H	1.569488000000	1.283798000000	1.132615000000
H	1.695330000000	3.008557000000	1.502946000000
H	2.153943000000	2.410265000000	-0.110027000000

TS_arylation_B-_

Imaginary frequency -261.38 cm^{-1}

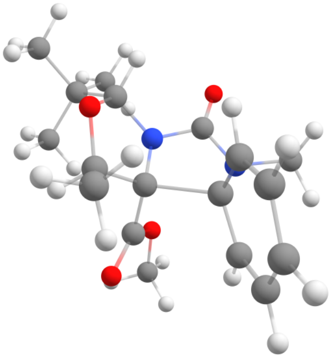

O	-0.277072000000	1.279743000000	-0.471992000000
C	-0.130022000000	2.338832000000	0.495234000000
C	-1.571077000000	0.693629000000	-0.357877000000
C	-1.164457000000	2.009576000000	1.583545000000
N	-1.995316000000	0.987805000000	0.997127000000
C	-1.848029000000	3.214729000000	2.059609000000
H	-1.461481000000	-0.388668000000	-0.472808000000
C	-2.552592000000	1.181161000000	-1.451636000000
C	-2.331724000000	-0.031609000000	1.877653000000
O	-2.964595000000	-1.034295000000	1.554530000000
N	-1.876012000000	0.277609000000	3.122414000000
C	-2.056406000000	-0.685638000000	4.184242000000
C	-0.610259000000	1.094147000000	3.133592000000
H	-1.344947000000	-1.516284000000	4.119938000000
H	-3.067140000000	-1.086487000000	4.125411000000
H	-1.906521000000	-0.180592000000	5.138907000000
C	0.556389000000	0.250128000000	2.910433000000
C	-0.454370000000	1.969698000000	4.283508000000
C	-2.815557000000	2.686772000000	-1.352969000000
C	-3.873811000000	0.425254000000	-1.272367000000
C	-1.946459000000	0.847325000000	-2.819472000000
H	-3.220398000000	2.946599000000	-0.374407000000
H	-3.545849000000	2.986125000000	-2.110289000000
H	-1.906616000000	3.265273000000	-1.519887000000
H	-4.320394000000	0.650548000000	-0.304402000000
H	-3.722054000000	-0.654937000000	-1.311906000000
H	-4.576403000000	0.707829000000	-2.061879000000
H	-0.996384000000	1.363855000000	-2.960808000000
H	-2.628078000000	1.143526000000	-3.621722000000
H	-1.764146000000	-0.226882000000	-2.914390000000
O	-1.295373000000	4.284125000000	2.241902000000

O	-3.151254000000	3.013129000000	2.410008000000
C	-3.789747000000	4.119057000000	3.036059000000
H	-4.794707000000	3.781042000000	3.282541000000
H	-3.834800000000	4.979792000000	2.365192000000
H	-3.257085000000	4.415949000000	3.941035000000
C	0.752394000000	2.139702000000	4.919541000000
C	1.743672000000	0.429122000000	3.583208000000
C	1.894894000000	1.401413000000	4.577165000000
H	0.806131000000	2.859130000000	5.731678000000
H	2.830092000000	1.532072000000	5.105101000000
H	0.475084000000	-0.524029000000	2.154602000000
H	2.580910000000	-0.217941000000	3.335284000000
H	-1.315145000000	2.543018000000	4.607673000000
H	-0.437585000000	3.281628000000	0.030187000000
C	1.330388000000	2.494355000000	0.873354000000
H	1.774318000000	1.551753000000	1.170489000000
H	1.410624000000	3.190686000000	1.708102000000
H	1.880104000000	2.900473000000	0.020573000000

TS_elimination_B-

Imaginary frequency -293.73 cm^{-1}

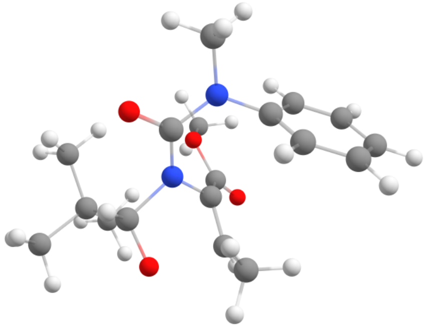

O	-0.330479000000	1.655202000000	-1.168101000000
C	0.385977000000	2.506885000000	0.582946000000
C	-1.357764000000	0.926775000000	-0.710540000000
C	-0.793143000000	2.241679000000	1.256926000000
N	-1.373124000000	1.021800000000	0.859212000000
C	-1.581909000000	3.319894000000	1.781547000000
H	-1.253509000000	-0.155656000000	-0.882081000000
C	-2.732800000000	1.364327000000	-1.338313000000
C	-1.728869000000	-0.044137000000	1.608360000000
O	-2.228773000000	-1.068353000000	1.136602000000
N	-1.583999000000	0.050888000000	3.009431000000
C	-2.109212000000	-1.072543000000	3.771395000000
C	-0.529548000000	0.753920000000	3.659743000000
H	-1.436608000000	-1.940851000000	3.760325000000
H	-3.061508000000	-1.381432000000	3.348881000000
H	-2.246961000000	-0.754778000000	4.806036000000
C	0.794428000000	0.346034000000	3.512135000000
C	-0.827121000000	1.789522000000	4.544305000000
C	-2.906187000000	2.884939000000	-1.277832000000
C	-3.933792000000	0.683298000000	-0.679980000000
C	-2.658386000000	0.937297000000	-2.812313000000

H	-3.140426000000	3.217110000000	-0.267912000000
H	-3.730833000000	3.195537000000	-1.928707000000
H	-1.991033000000	3.378785000000	-1.603121000000
H	-4.038161000000	0.998786000000	0.358698000000
H	-3.830726000000	-0.401670000000	-0.684945000000
H	-4.853143000000	0.952934000000	-1.212845000000
H	-1.809349000000	1.416017000000	-3.302064000000
H	-3.576407000000	1.213928000000	-3.341872000000
H	-2.533768000000	-0.146571000000	-2.896794000000
O	-1.223348000000	4.475034000000	1.969213000000
O	-2.843273000000	2.910896000000	2.168575000000
C	-3.661800000000	3.922861000000	2.729100000000
H	-4.594117000000	3.432498000000	3.007213000000
H	-3.860016000000	4.718272000000	2.006863000000
H	-3.194360000000	4.372757000000	3.608280000000
C	0.188042000000	2.440484000000	5.229217000000
C	1.811695000000	0.999999000000	4.194720000000
C	1.513424000000	2.052950000000	5.052182000000
H	-0.051525000000	3.268115000000	5.885565000000
H	2.307360000000	2.571862000000	5.575138000000
H	1.018647000000	-0.465555000000	2.832887000000
H	2.840374000000	0.692637000000	4.049022000000
H	-1.857919000000	2.100924000000	4.643670000000
H	0.610929000000	3.561590000000	0.446737000000
C	1.573645000000	1.602385000000	0.512023000000
H	1.288717000000	0.555635000000	0.590393000000
H	2.254597000000	1.845530000000	1.339213000000
H	2.107196000000	1.740810000000	-0.428101000000

GS_B-_conf2

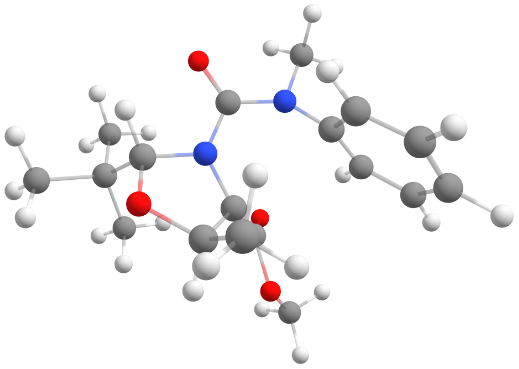

O	-0.289284000000	1.501512000000	-0.952403000000
C	0.140208000000	2.382500000000	0.137331000000
C	-1.463417000000	0.784129000000	-0.575388000000
C	-0.919558000000	2.220568000000	1.176792000000
N	-1.563961000000	0.982943000000	0.864974000000
C	-1.594543000000	3.243788000000	1.815975000000
H	-1.312665000000	-0.278558000000	-0.778941000000
C	-2.689603000000	1.262858000000	-1.397169000000
C	-1.888794000000	-0.060080000000	1.674865000000
O	-2.424234000000	-1.088003000000	1.243593000000
N	-1.690142000000	0.100308000000	3.045521000000
C	-2.280333000000	-0.918956000000	3.897471000000

C	-0.578400000000	0.784541000000	3.622256000000
H	-1.663006000000	-1.825302000000	3.959187000000
H	-3.252975000000	-1.199610000000	3.501861000000
H	-2.393506000000	-0.507794000000	4.901472000000
C	0.712343000000	0.286447000000	3.458902000000
C	-0.795294000000	1.862487000000	4.479566000000
C	-2.987766000000	2.747686000000	-1.165791000000
C	-3.921562000000	0.436844000000	-1.011776000000
C	-2.364558000000	1.026924000000	-2.878556000000
H	-3.278723000000	2.934530000000	-0.132823000000
H	-3.810565000000	3.059517000000	-1.817857000000
H	-2.119709000000	3.366431000000	-1.396247000000
H	-4.191031000000	0.601917000000	0.030625000000
H	-3.742097000000	-0.632101000000	-1.135650000000
H	-4.769772000000	0.726223000000	-1.640299000000
H	-1.490752000000	1.605259000000	-3.180261000000
H	-3.213001000000	1.320176000000	-3.503939000000
H	-2.155767000000	-0.029983000000	-3.069974000000
O	-2.678499000000	3.219645000000	2.421230000000
O	-0.845946000000	4.448006000000	1.800070000000
C	-1.447470000000	5.525557000000	2.484261000000
H	-0.766876000000	6.372190000000	2.374831000000
H	-1.588668000000	5.303931000000	3.546221000000
H	-2.425155000000	5.782326000000	2.066047000000
C	0.277855000000	2.468552000000	5.114664000000
C	1.786327000000	0.896590000000	4.094245000000
C	1.573615000000	1.993507000000	4.921497000000
H	0.107366000000	3.330833000000	5.748325000000
H	2.410481000000	2.476990000000	5.411125000000
H	0.866207000000	-0.562852000000	2.805019000000
H	2.790381000000	0.521256000000	3.935516000000
H	-1.799392000000	2.254382000000	4.564534000000
H	0.158823000000	3.408978000000	-0.241333000000
C	1.558672000000	1.971464000000	0.515974000000
H	1.571187000000	0.943244000000	0.877646000000
H	1.928637000000	2.620893000000	1.311549000000
H	2.227426000000	2.045595000000	-0.347883000000

TS_arylation_B-_conf2

Imaginary frequency -291.34 cm^{-1}

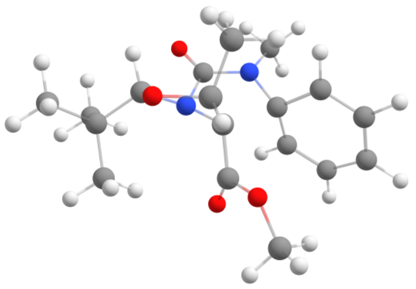

O	-0.573860000000	1.718553000000	-0.938033000000
C	0.251463000000	1.866449000000	0.256246000000
C	-1.735894000000	0.952850000000	-0.601963000000
C	-0.809657000000	1.921558000000	1.365193000000
N	-1.805627000000	0.992637000000	0.868993000000
C	-1.282314000000	3.259531000000	1.734495000000
H	-1.612951000000	-0.116019000000	-0.900998000000
C	-2.973848000000	1.506589000000	-1.354113000000
C	-1.771354000000	-0.248288000000	1.527076000000
O	-2.327003000000	-1.268866000000	1.091895000000
N	-1.022621000000	-0.178031000000	2.673889000000
C	-1.016072000000	-1.280825000000	3.607113000000
C	-0.511773000000	1.129636000000	3.133164000000
H	0.022852000000	-1.519080000000	3.916362000000
H	-1.473145000000	-2.155718000000	3.107889000000
H	-1.589427000000	-1.029037000000	4.528075000000
C	0.892006000000	1.144656000000	3.536564000000
C	-1.384241000000	1.768396000000	4.125869000000
C	-3.231877000000	2.979321000000	-1.003650000000
C	-4.191375000000	0.654168000000	-0.957888000000
C	-2.701518000000	1.362747000000	-2.863466000000
H	-3.454628000000	3.099745000000	0.073987000000
H	-4.092962000000	3.364669000000	-1.590236000000
H	-2.344515000000	3.601142000000	-1.237082000000
H	-4.396017000000	0.748709000000	0.126464000000
H	-4.021537000000	-0.421778000000	-1.166667000000
H	-5.090608000000	0.986977000000	-1.518673000000
H	-1.816567000000	1.958793000000	-3.163512000000
H	-3.577107000000	1.713317000000	-3.450365000000
H	-2.510860000000	0.303205000000	-3.139016000000
O	-2.410080000000	3.566629000000	2.114789000000
O	-0.221602000000	4.143270000000	1.816060000000
C	-0.462035000000	5.329035000000	2.563479000000
H	0.391840000000	6.003794000000	2.361382000000
H	-0.500754000000	5.089782000000	3.649189000000
H	-1.416124000000	5.810254000000	2.264354000000
C	-0.888777000000	2.602579000000	5.122835000000
C	1.367852000000	1.991660000000	4.537669000000
C	0.500328000000	2.779559000000	5.326642000000
H	-1.608841000000	3.111860000000	5.786722000000
H	0.884881000000	3.430190000000	6.126590000000
H	1.597375000000	0.496682000000	2.997267000000
H	2.453402000000	2.007813000000	4.739074000000
H	-2.470363000000	1.640636000000	4.004077000000
H	0.772398000000	2.836627000000	0.150295000000
C	1.270761000000	0.728705000000	0.303805000000
H	0.795438000000	-0.243673000000	0.539677000000
H	2.043390000000	0.928829000000	1.070072000000
H	1.766449000000	0.648056000000	-0.684733000000

TS_elimination_B-_conf2

Imaginary frequency -374.26 cm^{-1}

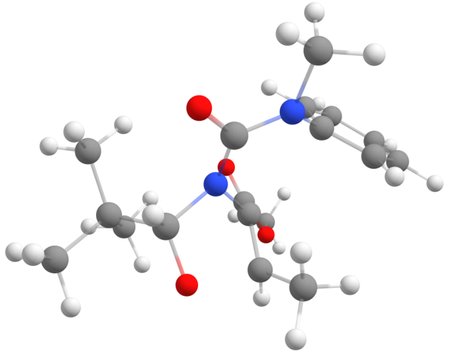

O	-0.482662000000	1.497675000000	-1.301592000000
C	0.474878000000	2.056775000000	0.191768000000
C	-1.600415000000	0.885779000000	-0.749678000000
C	-0.641733000000	2.076311000000	1.096654000000
N	-1.493540000000	0.982593000000	0.768075000000
C	-1.185990000000	3.263783000000	1.668231000000
H	-1.625259000000	-0.209514000000	-0.967366000000
C	-2.935603000000	1.488514000000	-1.309137000000
C	-1.633910000000	-0.186175000000	1.484210000000
O	-2.209553000000	-1.187837000000	1.019740000000
N	-1.069013000000	-0.244049000000	2.774487000000
C	-1.247916000000	-1.478487000000	3.522497000000
C	-0.550854000000	0.837693000000	3.558672000000
H	-0.280393000000	-1.820045000000	3.949600000000
H	-1.645743000000	-2.243185000000	2.830511000000
H	-1.962521000000	-1.342993000000	4.368014000000
C	0.834519000000	0.895822000000	3.839153000000
C	-1.427741000000	1.690041000000	4.265028000000
C	-3.010348000000	3.004790000000	-1.074254000000
C	-4.138739000000	0.794526000000	-0.651185000000
C	-2.937272000000	1.205100000000	-2.823819000000
H	-3.147297000000	3.240916000000	-0.001552000000
H	-3.866268000000	3.438148000000	-1.636627000000
H	-2.077073000000	3.491378000000	-1.423225000000
H	-4.168366000000	1.012576000000	0.434723000000
H	-4.083877000000	-0.307104000000	-0.766157000000
H	-5.088791000000	1.153150000000	-1.104252000000
H	-2.066725000000	1.691645000000	-3.308206000000
H	-3.869149000000	1.588149000000	-3.294473000000
H	-2.875365000000	0.114125000000	-3.027932000000
O	-2.300942000000	3.424088000000	2.191995000000
O	-0.250619000000	4.313440000000	1.686783000000
C	-0.672688000000	5.474632000000	2.375898000000
H	0.130852000000	6.225939000000	2.241353000000
H	-0.814509000000	5.274049000000	3.461407000000
H	-1.631568000000	5.871655000000	1.977191000000
C	-0.916605000000	2.638341000000	5.163454000000
C	1.339050000000	1.838714000000	4.741093000000
C	0.464641000000	2.723863000000	5.403375000000
H	-1.610148000000	3.324437000000	5.674677000000

H	0.861506000000	3.473497000000	6.105962000000
H	1.508316000000	0.207615000000	3.307164000000
H	2.424050000000	1.893853000000	4.924870000000
H	-2.504680000000	1.635150000000	4.058463000000
H	0.885625000000	3.045456000000	-0.081795000000
C	1.499635000000	0.951267000000	0.236268000000
H	1.030325000000	-0.022925000000	0.474687000000
H	2.246867000000	1.177322000000	1.031197000000
H	2.036111000000	0.857797000000	-0.729098000000

7.5.2 Potassium Enolates (Neutral; Charge 0)

GS_A-_K+_conf8

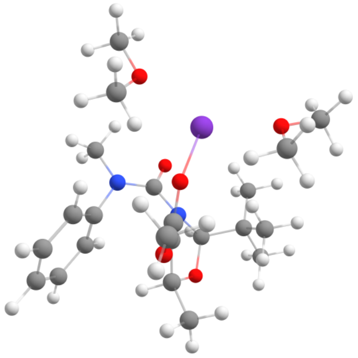

O	2.629199000000	0.585737000000	3.194991000000
C	2.028107000000	0.919401000000	1.892095000000
C	1.629930000000	0.251244000000	4.154331000000
C	0.609948000000	0.472135000000	2.057413000000
C	2.861742000000	0.303975000000	0.780764000000
H	2.096121000000	2.034175000000	1.813630000000
N	0.354421000000	0.523233000000	3.461129000000
C	-0.294121000000	-0.167292000000	1.213455000000
H	1.703001000000	0.932869000000	5.033472000000
C	1.836494000000	-1.211050000000	4.657051000000
C	-0.644020000000	1.271960000000	4.069588000000
O	-0.982276000000	1.065428000000	5.258525000000
N	-1.372456000000	2.137757000000	3.259872000000
C	-2.545908000000	2.773994000000	3.846090000000
C	-0.791419000000	2.832901000000	2.143309000000
H	-2.283218000000	3.700543000000	4.404410000000
H	-3.030846000000	2.077904000000	4.553337000000
H	-3.254692000000	3.043561000000	3.039348000000
C	0.275603000000	3.728663000000	2.356561000000
C	-1.402245000000	2.767324000000	0.874385000000
H	3.922044000000	0.601391000000	0.901575000000
H	2.499407000000	0.658989000000	-0.202337000000
H	2.795965000000	-0.801554000000	0.793330000000
C	3.302102000000	-1.347624000000	5.112621000000
C	1.545883000000	-2.221095000000	3.536603000000
C	0.918841000000	-1.475717000000	5.862070000000
H	3.994544000000	-1.179055000000	4.266256000000
H	3.484352000000	-2.361688000000	5.525691000000
H	3.547019000000	-0.610140000000	5.905696000000

H	2.209866000000	-2.051368000000	2.667778000000
H	0.502360000000	-2.142112000000	3.177501000000
H	1.713136000000	-3.255958000000	3.902535000000
H	-0.144923000000	-1.300306000000	5.617345000000
H	1.150984000000	-0.795498000000	6.706468000000
H	1.037847000000	-2.520632000000	6.216162000000
O	-1.407704000000	-0.691910000000	1.553675000000
O	0.047566000000	-0.130098000000	-0.136188000000
C	-0.825174000000	-0.808016000000	-1.021689000000
H	-1.860197000000	-0.406378000000	-0.976220000000
H	-0.873049000000	-1.899168000000	-0.809241000000
H	-0.412719000000	-0.653033000000	-2.036861000000
C	-0.916327000000	3.547940000000	-0.179281000000
C	0.760390000000	4.509930000000	1.294641000000
C	0.170007000000	4.421457000000	0.025123000000
H	-1.380252000000	3.469394000000	-1.174270000000
H	0.550162000000	5.035168000000	-0.805375000000
H	0.724075000000	3.800729000000	3.359019000000
H	1.604014000000	5.196304000000	1.465541000000
H	-2.232478000000	2.063685000000	0.721118000000
K	-2.299098000000	-1.138958000000	3.982017000000
O	-4.593473000000	-0.022378000000	3.081741000000
C	-5.779821000000	0.455594000000	3.685919000000
H	-6.687482000000	0.013100000000	3.211861000000
H	-5.851584000000	1.566536000000	3.615352000000
H	-5.759055000000	0.168021000000	4.755191000000
C	-4.504914000000	0.291757000000	1.694358000000
H	-3.508873000000	-0.045270000000	1.340874000000
H	-4.598162000000	1.390925000000	1.533317000000
H	-5.313127000000	-0.218062000000	1.119675000000
H	-2.108908000000	-4.733849000000	1.619800000000
H	-0.396041000000	-4.249221000000	1.959892000000
C	-1.462206000000	-3.944652000000	2.068967000000
H	-1.604217000000	-2.977231000000	1.546565000000
O	-1.790933000000	-3.739687000000	3.441346000000
H	-0.381388000000	-5.074732000000	4.226833000000
C	-1.472706000000	-4.848494000000	4.260895000000
H	-2.034394000000	-5.761296000000	3.951529000000
H	-1.750686000000	-4.593844000000	5.302269000000

TS_arylation_ A-_K+_conf8
Imaginary frequency -295.40 cm^{-1}

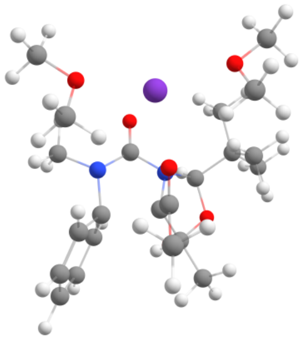

O	2.496824000000	0.223857000000	2.988586000000
C	1.935523000000	1.059112000000	1.959746000000
C	1.586543000000	0.080792000000	4.057230000000
C	0.430268000000	0.812820000000	2.069253000000
C	2.677818000000	0.799111000000	0.671430000000
H	2.084959000000	2.109904000000	2.234808000000
N	0.278650000000	0.389637000000	3.448339000000
C	-0.240498000000	-0.145784000000	1.160382000000
H	1.786538000000	0.815290000000	4.850331000000
C	1.753019000000	-1.342972000000	4.635021000000
C	-0.712846000000	1.117537000000	4.096050000000
O	-1.048226000000	0.913969000000	5.269584000000
N	-1.324806000000	1.939180000000	3.219188000000
C	-2.341715000000	2.862076000000	3.670454000000
C	-0.483856000000	2.319689000000	2.012169000000
H	-1.906345000000	3.794494000000	4.039058000000
H	-2.910398000000	2.395330000000	4.472397000000
H	-2.990589000000	3.104580000000	2.828534000000
C	0.331873000000	3.496587000000	2.314462000000
C	-1.268778000000	2.504822000000	0.792169000000
H	3.731752000000	1.037942000000	0.823663000000
H	2.286443000000	1.437242000000	-0.119335000000
H	2.597145000000	-0.241540000000	0.358505000000
C	3.215660000000	-1.505245000000	5.074600000000
C	1.423753000000	-2.383834000000	3.561483000000
C	0.852884000000	-1.531386000000	5.859313000000
H	3.893240000000	-1.411050000000	4.227140000000
H	3.362242000000	-2.487623000000	5.530314000000
H	3.488733000000	-0.749284000000	5.815331000000
H	2.082995000000	-2.268966000000	2.701419000000
H	0.397859000000	-2.283627000000	3.211036000000
H	1.554320000000	-3.391648000000	3.963064000000
H	-0.190726000000	-1.326048000000	5.632936000000
H	1.124570000000	-0.843873000000	6.662425000000
H	0.947074000000	-2.552773000000	6.235513000000
O	-1.130898000000	-0.928211000000	1.495499000000
O	0.084077000000	0.032085000000	-0.123719000000
C	-0.693047000000	-0.671941000000	-1.096235000000
H	-1.743433000000	-0.391219000000	-1.020318000000
H	-0.597596000000	-1.749663000000	-0.960321000000
H	-0.293237000000	-0.372029000000	-2.061022000000
C	-1.052596000000	3.545511000000	-0.078278000000
C	0.520306000000	4.527447000000	1.416390000000
C	-0.129112000000	4.566530000000	0.182115000000
H	-1.639197000000	3.577146000000	-0.991708000000
H	0.020984000000	5.381745000000	-0.511563000000
H	0.806884000000	3.542003000000	3.289264000000
H	1.173672000000	5.345137000000	1.705280000000
H	-2.004685000000	1.748611000000	0.545105000000
K	-2.305209000000	-1.232915000000	3.906626000000
O	-4.468725000000	-0.010738000000	2.916923000000
C	-5.512346000000	0.678277000000	3.584424000000

H	-6.488780000000	0.413706000000	3.162219000000
H	-5.374284000000	1.762197000000	3.511735000000
H	-5.485189000000	0.385807000000	4.633810000000
C	-4.388424000000	0.327327000000	1.536877000000
H	-3.521660000000	-0.189022000000	1.128943000000
H	-4.250038000000	1.404893000000	1.410034000000
H	-5.295766000000	0.011897000000	1.009272000000
H	-2.128621000000	-5.142029000000	2.006559000000
H	-0.462235000000	-4.522038000000	2.179902000000
C	-1.523025000000	-4.270996000000	2.280759000000
H	-1.747187000000	-3.435475000000	1.620029000000
O	-1.816263000000	-3.854585000000	3.608469000000
H	-0.368871000000	-5.008885000000	4.545847000000
C	-1.446248000000	-4.815235000000	4.581741000000
H	-1.985436000000	-5.757247000000	4.429785000000
H	-1.701677000000	-4.410055000000	5.560402000000

TS_elimination_ A-_K+_conf8
Imaginary frequency -234.43 cm^{-1}

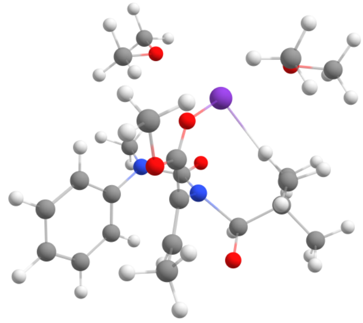

O	2.765054000000	0.392627000000	3.266924000000
C	1.794320000000	1.120044000000	1.493309000000
C	1.780368000000	0.171962000000	4.092321000000
C	0.601700000000	0.547668000000	1.876883000000
C	2.613494000000	0.819874000000	0.289066000000
H	2.078849000000	2.017066000000	2.016050000000
N	0.361033000000	0.603071000000	3.260389000000
C	-0.237408000000	-0.313276000000	1.098371000000
H	1.693716000000	0.856838000000	4.955612000000
C	1.717616000000	-1.306851000000	4.598809000000
C	-0.666309000000	1.237277000000	3.860241000000
O	-1.043156000000	0.969419000000	5.016180000000
N	-1.410922000000	2.165208000000	3.118130000000
C	-2.568781000000	2.750221000000	3.787759000000
C	-0.791955000000	3.017932000000	2.152935000000
H	-2.278939000000	3.476491000000	4.554132000000
H	-3.154029000000	1.971010000000	4.268169000000
H	-3.178034000000	3.256313000000	3.039749000000
C	0.247283000000	3.864705000000	2.525845000000
C	-1.266266000000	3.044445000000	0.844953000000
H	3.658637000000	1.036310000000	0.513338000000
H	2.309509000000	1.465062000000	-0.542206000000
H	2.521312000000	-0.212756000000	-0.036628000000

C	3.126924000000	-1.640524000000	5.115697000000
C	1.385749000000	-2.251770000000	3.445365000000
C	0.743591000000	-1.487375000000	5.763968000000
H	3.855777000000	-1.564772000000	4.311184000000
H	3.147044000000	-2.653998000000	5.528551000000
H	3.426908000000	-0.948387000000	5.906511000000
H	2.053144000000	-2.059124000000	2.604350000000
H	0.363488000000	-2.124207000000	3.094994000000
H	1.515996000000	-3.293059000000	3.753927000000
H	-0.258380000000	-1.150393000000	5.519520000000
H	1.053117000000	-0.888018000000	6.622886000000
H	0.713398000000	-2.534184000000	6.079588000000
O	-1.236796000000	-0.917663000000	1.512124000000
O	0.075846000000	-0.358322000000	-0.223491000000
C	-0.755551000000	-1.171944000000	-1.046399000000
H	-1.788727000000	-0.822240000000	-1.029364000000
H	-0.732593000000	-2.213272000000	-0.721890000000
H	-0.348141000000	-1.084775000000	-2.050885000000
C	-0.687993000000	3.889219000000	-0.091421000000
C	0.832607000000	4.704600000000	1.585600000000
C	0.369056000000	4.717380000000	0.275512000000
H	-1.049993000000	3.891207000000	-1.111758000000
H	0.828295000000	5.368720000000	-0.456995000000
H	0.610625000000	3.840826000000	3.545308000000
H	1.653958000000	5.346844000000	1.877015000000
H	-2.066225000000	2.371776000000	0.564881000000
K	-2.259688000000	-1.219852000000	3.918372000000
O	-4.554533000000	-0.118327000000	2.977503000000
C	-5.719527000000	0.377868000000	3.611023000000
H	-6.623854000000	0.047971000000	3.087278000000
H	-5.709701000000	1.472942000000	3.646808000000
H	-5.733457000000	-0.011151000000	4.628318000000
C	-4.412648000000	0.349786000000	1.641262000000
H	-3.471968000000	-0.041335000000	1.258022000000
H	-4.384975000000	1.444110000000	1.617737000000
H	-5.245762000000	0.000316000000	1.021212000000
H	-1.966232000000	-5.246257000000	2.162601000000
H	-0.390668000000	-4.412456000000	2.286199000000
C	-1.473036000000	-4.295567000000	2.393952000000
H	-1.809376000000	-3.523375000000	1.704607000000
O	-1.802641000000	-3.867964000000	3.709030000000
H	-0.219765000000	-4.820712000000	4.654691000000
C	-1.310156000000	-4.744021000000	4.708140000000
H	-1.747646000000	-5.743370000000	4.603627000000
H	-1.588554000000	-4.329452000000	5.675951000000

Product_ A-_K+_conf8

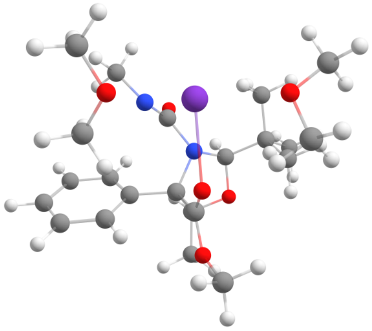

O	2.540864000000	-0.012900000000	2.626802000000
C	2.079840000000	1.054133000000	1.823509000000
C	1.691906000000	-0.006884000000	3.769643000000
C	0.531043000000	0.849568000000	1.869320000000
C	2.858820000000	1.132204000000	0.529790000000
H	2.229291000000	1.998094000000	2.362169000000
N	0.368511000000	0.459068000000	3.293071000000
C	0.022158000000	-0.329602000000	1.006235000000
H	2.054793000000	0.725998000000	4.494769000000
C	1.699665000000	-1.397501000000	4.424860000000
C	-0.298685000000	1.399278000000	4.228562000000
O	0.410535000000	1.986387000000	5.065924000000
N	-1.596871000000	1.474822000000	4.042425000000
C	-2.231513000000	2.489374000000	4.866112000000
C	-0.168433000000	2.126663000000	1.411084000000
H	-1.846770000000	3.496619000000	4.657508000000
H	-2.081218000000	2.322490000000	5.940238000000
H	-3.307284000000	2.498825000000	4.668601000000
C	0.018927000000	3.319566000000	2.115313000000
C	-0.937362000000	2.164913000000	0.250010000000
H	3.854385000000	1.523134000000	0.744655000000
H	2.367169000000	1.811711000000	-0.167033000000
H	2.960383000000	0.158595000000	0.060373000000
C	3.132680000000	-1.692198000000	4.889872000000
C	1.244632000000	-2.483061000000	3.446630000000
C	0.773830000000	-1.366177000000	5.646381000000
H	3.819307000000	-1.724138000000	4.043882000000
H	3.175056000000	-2.654292000000	5.406117000000
H	3.482460000000	-0.922812000000	5.581746000000
H	1.893558000000	-2.513802000000	2.570450000000
H	0.225966000000	-2.309236000000	3.106063000000
H	1.283063000000	-3.462386000000	3.930272000000
H	-0.259435000000	-1.164831000000	5.360787000000
H	1.067819000000	-0.581699000000	6.344397000000
H	0.804647000000	-2.326809000000	6.165990000000
O	-0.951585000000	-1.001198000000	1.272656000000
O	0.726172000000	-0.539804000000	-0.105991000000
C	0.283478000000	-1.609501000000	-0.955896000000
H	-0.732895000000	-1.424972000000	-1.302480000000
H	0.312880000000	-2.555365000000	-0.416747000000
H	0.977472000000	-1.621734000000	-1.791255000000

C	-1.521589000000	3.348598000000	-0.189982000000
C	-0.566557000000	4.499229000000	1.682019000000
C	-1.342732000000	4.521853000000	0.527465000000
H	-2.118575000000	3.345841000000	-1.093433000000
H	-1.799897000000	5.444188000000	0.192407000000
H	0.591740000000	3.325093000000	3.032201000000
H	-0.420084000000	5.404628000000	2.257073000000
H	-1.101337000000	1.268616000000	-0.332133000000
K	-2.422630000000	-0.947676000000	3.461543000000
O	-4.632891000000	0.255826000000	2.316743000000
C	-5.625094000000	0.820508000000	3.154379000000
H	-6.410882000000	1.301413000000	2.560060000000
H	-5.196264000000	1.562833000000	3.836249000000
H	-6.066585000000	0.009584000000	3.733389000000
C	-3.979683000000	1.234441000000	1.510256000000
H	-3.319660000000	0.697769000000	0.832765000000
H	-3.376346000000	1.907897000000	2.123435000000
H	-4.711169000000	1.803071000000	0.925782000000
H	-2.430820000000	-5.246663000000	2.241007000000
H	-0.789489000000	-4.550217000000	2.336426000000
C	-1.859533000000	-4.320759000000	2.370586000000
H	-2.096650000000	-3.628033000000	1.565145000000
O	-2.199083000000	-3.685431000000	3.593506000000
H	-0.773041000000	-4.665455000000	4.742138000000
C	-1.848674000000	-4.461632000000	4.726651000000
H	-2.394231000000	-5.411961000000	4.735508000000
H	-2.113463000000	-3.887739000000	5.613438000000

GS_ A-_K+_conf3

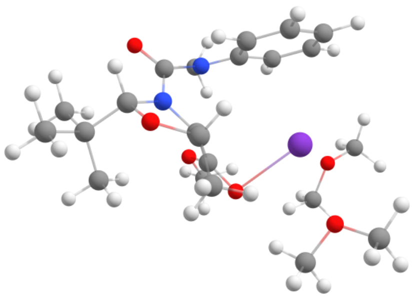

O	-0.292136000000	1.762699000000	-0.755895000000
C	0.160861000000	2.551428000000	0.381033000000
C	-1.378506000000	0.914351000000	-0.392801000000
C	-0.915041000000	2.350818000000	1.404769000000
C	0.448170000000	3.961216000000	-0.099400000000
H	1.115897000000	2.087845000000	0.686529000000
N	-1.487955000000	1.086862000000	1.059018000000
C	-1.600300000000	3.308996000000	2.124636000000
H	-1.112611000000	-0.127008000000	-0.593472000000
C	-2.640440000000	1.264964000000	-1.217581000000
C	-1.769306000000	0.028532000000	1.854846000000
O	-2.275950000000	-1.007658000000	1.453611000000
N	-1.513035000000	0.210627000000	3.251096000000

C	-2.627857000000	-0.047109000000	4.145852000000
C	-0.246634000000	0.409017000000	3.746955000000
H	-2.355139000000	-0.778999000000	4.909834000000
H	-3.445129000000	-0.454115000000	3.558032000000
H	-2.966112000000	0.871693000000	4.633309000000
C	0.895400000000	0.254668000000	2.931969000000
C	-0.041187000000	0.739463000000	5.101200000000
H	-0.472968000000	4.502359000000	-0.306167000000
H	1.045280000000	3.904323000000	-1.010724000000
H	1.013841000000	4.528229000000	0.639975000000
C	-3.081716000000	2.714548000000	-0.994985000000
C	-3.789937000000	0.324150000000	-0.840190000000
C	-2.282077000000	1.054795000000	-2.695782000000
H	-3.353402000000	2.890974000000	0.045031000000
H	-3.956788000000	2.926018000000	-1.614684000000
H	-2.296364000000	3.416110000000	-1.274269000000
H	-4.104351000000	0.475839000000	0.191750000000
H	-3.504870000000	-0.722711000000	-0.944625000000
H	-4.645856000000	0.517875000000	-1.491585000000
H	-1.467025000000	1.711408000000	-3.000080000000
H	-3.150028000000	1.264191000000	-3.325121000000
H	-1.975813000000	0.021923000000	-2.881799000000
O	-1.185747000000	4.458856000000	2.445474000000
O	-2.834180000000	2.890012000000	2.596403000000
C	-3.467370000000	3.743175000000	3.534985000000
H	-4.446527000000	3.304821000000	3.721359000000
H	-3.581403000000	4.756800000000	3.149110000000
H	-2.905238000000	3.789427000000	4.472347000000
C	1.243751000000	0.911412000000	5.605677000000
C	2.168321000000	0.434058000000	3.447628000000
C	2.363023000000	0.770373000000	4.790887000000
H	1.365760000000	1.151877000000	6.655067000000
H	3.360701000000	0.883668000000	5.194130000000
H	0.773992000000	-0.030296000000	1.897030000000
H	3.022092000000	0.288797000000	2.796426000000
H	-0.888940000000	0.869358000000	5.757370000000
K	1.071430000000	3.751898000000	3.444806000000
O	1.929027000000	6.192805000000	2.565801000000
C	3.189996000000	6.830030000000	2.502131000000
H	3.184659000000	7.773696000000	3.060116000000
H	3.474457000000	7.036178000000	1.463925000000
H	3.924665000000	6.158502000000	2.945837000000
C	0.879181000000	6.984298000000	2.012535000000
H	-0.029717000000	6.386906000000	2.049785000000
H	1.102983000000	7.241472000000	0.971708000000
H	0.754610000000	7.907508000000	2.589301000000
H	-1.702210000000	5.564825000000	6.192928000000
H	-1.086776000000	5.683478000000	4.527557000000
C	-0.808306000000	5.722751000000	5.579505000000
H	-0.377465000000	6.700121000000	5.825777000000
O	0.139499000000	4.685323000000	5.801646000000
H	-0.385032000000	4.300015000000	7.772279000000

C	0.494235000000	4.535087000000	7.161533000000
H	0.961707000000	5.446530000000	7.553140000000
H	1.202978000000	3.710879000000	7.225182000000

TS_arylation_ A-_K+_conf3
Imaginary frequency -201.64 cm^{-1}

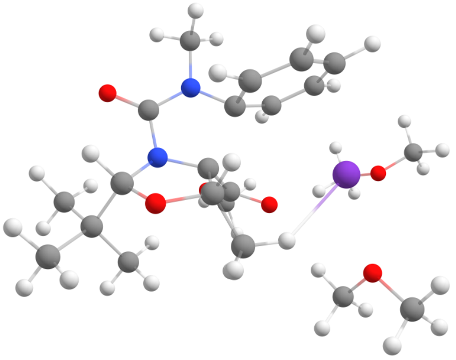

O	-0.360825000000	1.502500000000	-0.507414000000
C	0.089591000000	2.122076000000	0.713361000000
C	-1.646519000000	0.888520000000	-0.360119000000
C	-1.060806000000	1.909664000000	1.722603000000
C	0.547741000000	3.537051000000	0.366078000000
H	0.974052000000	1.546166000000	1.086397000000
N	-1.943871000000	0.984873000000	1.062501000000
C	-1.669951000000	3.099078000000	2.332156000000
H	-1.573855000000	-0.190711000000	-0.625925000000
C	-2.698482000000	1.529752000000	-1.306884000000
C	-2.194780000000	-0.194529000000	1.781315000000
O	-2.835560000000	-1.147482000000	1.332071000000
N	-1.629425000000	-0.103031000000	3.027856000000
C	-1.729695000000	-1.215342000000	3.948586000000
C	-0.526485000000	0.872870000000	3.222456000000
H	-0.814052000000	-1.847227000000	3.932441000000
H	-2.594278000000	-1.834470000000	3.646294000000
H	-1.871021000000	-0.843842000000	4.983547000000
C	0.810726000000	0.296700000000	2.991160000000
C	-0.620365000000	1.562147000000	4.523043000000
H	-0.301596000000	4.215404000000	0.161337000000
H	1.171512000000	3.473148000000	-0.546663000000
H	1.168673000000	4.000346000000	1.153953000000
C	-2.878597000000	3.027735000000	-1.019106000000
C	-4.034118000000	0.797311000000	-1.089885000000
C	-2.213007000000	1.325123000000	-2.752963000000
H	-3.223583000000	3.191466000000	0.020117000000
H	-3.640475000000	3.457612000000	-1.701419000000
H	-1.934350000000	3.584682000000	-1.177156000000
H	-4.399044000000	0.940209000000	-0.054178000000
H	-3.930504000000	-0.294402000000	-1.250221000000
H	-4.800756000000	1.183706000000	-1.792849000000
H	-1.238474000000	1.824952000000	-2.922356000000
H	-2.948753000000	1.740677000000	-3.472099000000
H	-2.089119000000	0.246396000000	-2.984888000000
O	-1.009675000000	4.017489000000	2.853325000000

O	-3.017719000000	3.029935000000	2.468438000000
C	-3.641731000000	4.073268000000	3.216057000000
H	-4.728201000000	3.887163000000	3.146957000000
H	-3.397029000000	5.070210000000	2.795372000000
H	-3.323412000000	4.045728000000	4.278418000000
C	0.438147000000	1.636052000000	5.424288000000
C	1.868807000000	0.436633000000	3.890539000000
C	1.724888000000	1.119703000000	5.123934000000
H	0.257437000000	2.111274000000	6.403864000000
H	2.539613000000	1.145131000000	5.862413000000
H	0.936736000000	-0.348676000000	2.106286000000
H	2.828259000000	-0.051258000000	3.648259000000
H	-1.613745000000	1.930172000000	4.826518000000
K	1.507911000000	3.822893000000	3.778124000000
O	2.026036000000	6.105116000000	2.393317000000
C	3.184566000000	6.744387000000	1.889239000000
H	3.307891000000	7.765276000000	2.320942000000
H	3.148138000000	6.832051000000	0.778307000000
H	4.061339000000	6.129478000000	2.169932000000
C	0.824059000000	6.802996000000	2.086240000000
H	-0.022908000000	6.188497000000	2.446285000000
H	0.715325000000	6.945464000000	0.986209000000
H	0.804691000000	7.801366000000	2.581229000000
H	-1.150387000000	4.384183000000	6.675048000000
H	-1.143481000000	5.051940000000	4.999115000000
C	-0.764732000000	5.205941000000	6.027343000000
H	-1.135350000000	6.178867000000	6.426722000000
O	0.655611000000	5.187539000000	5.967544000000
H	0.984492000000	4.456925000000	7.906414000000
C	1.266553000000	5.304982000000	7.238703000000
H	0.983224000000	6.259752000000	7.740573000000
H	2.363771000000	5.293360000000	7.090578000000

TS_elimination_ A-_K+_conf3
Imaginary frequency -286.83 cm^{-1}

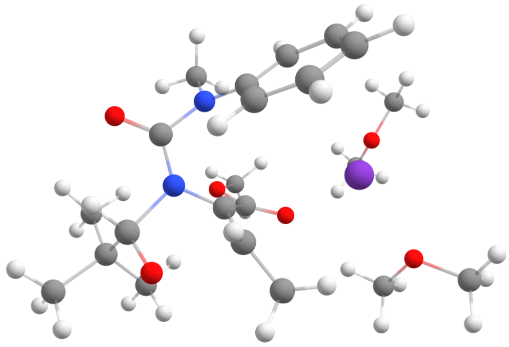

O	-0.404149000000	1.397809000000	-1.227151000000
C	0.360659000000	2.529937000000	0.503293000000
C	-1.425391000000	0.772664000000	-0.672082000000
C	-0.778503000000	2.245688000000	1.228248000000
C	0.847474000000	3.883298000000	0.111850000000
H	1.032358000000	1.717064000000	0.286840000000
N	-1.328338000000	0.994148000000	0.931991000000

C	-1.527787000000	3.211576000000	1.986238000000
H	-1.391570000000	-0.327325000000	-0.723705000000
C	-2.822322000000	1.242339000000	-1.205942000000
C	-1.667053000000	0.033781000000	1.805819000000
O	-2.251199000000	-0.999888000000	1.529457000000
N	-1.353069000000	0.306284000000	3.201513000000
C	-2.446653000000	0.073567000000	4.130821000000
C	-0.073349000000	0.455477000000	3.679150000000
H	-2.194749000000	-0.702503000000	4.858538000000
H	-3.308519000000	-0.259125000000	3.561892000000
H	-2.707659000000	0.989858000000	4.669715000000
C	1.056799000000	0.288847000000	2.849294000000
C	0.160108000000	0.764893000000	5.037370000000
H	0.120555000000	4.665870000000	0.310516000000
H	1.059292000000	3.858210000000	-0.959449000000
H	1.791790000000	4.143333000000	0.606608000000
C	-2.923623000000	2.771492000000	-1.212964000000
C	-3.993811000000	0.643954000000	-0.425538000000
C	-2.876147000000	0.741206000000	-2.657990000000
H	-3.077373000000	3.167284000000	-0.209515000000
H	-3.774458000000	3.088417000000	-1.822729000000
H	-2.014424000000	3.204828000000	-1.630009000000
H	-4.023271000000	1.025154000000	0.595623000000
H	-3.925274000000	-0.442623000000	-0.372907000000
H	-4.938320000000	0.907862000000	-0.911082000000
H	-2.060284000000	1.171501000000	-3.239012000000
H	-3.826620000000	1.017661000000	-3.122793000000
H	-2.784871000000	-0.347607000000	-2.699011000000
O	-1.064068000000	4.256950000000	2.469543000000
O	-2.800516000000	2.847398000000	2.252888000000
C	-3.542626000000	3.693454000000	3.127249000000
H	-4.513065000000	3.216968000000	3.239935000000
H	-3.659958000000	4.690225000000	2.701145000000
H	-3.048966000000	3.777408000000	4.096419000000
C	1.454379000000	0.907174000000	5.527998000000
C	2.338250000000	0.443679000000	3.350672000000
C	2.559738000000	0.762410000000	4.694195000000
H	1.595133000000	1.119226000000	6.581366000000
H	3.565285000000	0.850331000000	5.084230000000
H	0.914199000000	0.015455000000	1.814546000000
H	3.179985000000	0.290404000000	2.686022000000
H	-0.673257000000	0.887117000000	5.712822000000
K	1.287510000000	3.836894000000	3.640327000000
O	1.986013000000	6.295143000000	2.820473000000
C	3.185003000000	7.047533000000	2.876287000000
H	3.049921000000	7.959529000000	3.468575000000
H	3.520291000000	7.324502000000	1.870897000000
H	3.945586000000	6.425557000000	3.346987000000
C	0.920149000000	7.004770000000	2.194879000000
H	0.055556000000	6.344997000000	2.174539000000
H	1.190727000000	7.278286000000	1.170062000000
H	0.683063000000	7.914383000000	2.757579000000

H	-1.972223000000	5.364593000000	6.085080000000
H	-1.265552000000	5.699673000000	4.488002000000
C	-1.068779000000	5.690092000000	5.557722000000
H	-0.799119000000	6.694114000000	5.904010000000
O	-0.002978000000	4.777208000000	5.784842000000
H	-0.622427000000	4.192069000000	7.679319000000
C	0.264155000000	4.572844000000	7.160496000000
H	0.585770000000	5.502429000000	7.644070000000
H	1.061642000000	3.835215000000	7.233595000000

GS_ A-_K+_conf4

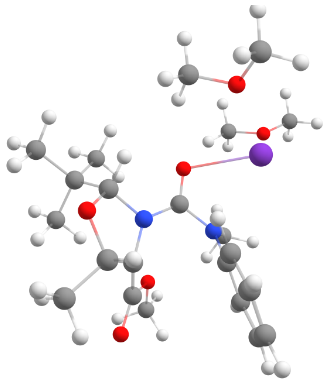

O	-0.138283000000	0.304172000000	0.088075000000
C	0.311528000000	1.622309000000	0.583818000000
C	-0.935979000000	-0.348582000000	1.052573000000
C	-0.507584000000	1.826615000000	1.805125000000
C	0.195230000000	2.644215000000	-0.524325000000
H	1.379717000000	1.474282000000	0.813632000000
N	-0.824585000000	0.510587000000	2.249175000000
C	-1.170586000000	2.977353000000	2.208831000000
H	-0.499861000000	-1.323154000000	1.291378000000
C	-2.376304000000	-0.576701000000	0.532547000000
C	-0.716135000000	-0.022314000000	3.465431000000
O	-1.051222000000	-1.201260000000	3.742369000000
N	-0.210610000000	0.780449000000	4.524932000000
C	-1.129299000000	1.007481000000	5.636744000000
C	0.859959000000	1.676933000000	4.318487000000
H	-0.563432000000	1.174828000000	6.555737000000
H	-1.745254000000	0.118698000000	5.754933000000
H	-1.782561000000	1.861394000000	5.454246000000
C	1.961779000000	1.260745000000	3.559050000000
C	0.904004000000	2.926373000000	4.936858000000
H	-0.846625000000	2.818157000000	-0.789286000000
H	0.737133000000	2.290965000000	-1.404114000000
H	0.615141000000	3.593561000000	-0.195199000000
C	-3.098678000000	0.744828000000	0.254405000000
C	-3.183555000000	-1.389357000000	1.550879000000
C	-2.258334000000	-1.381073000000	-0.770391000000
H	-3.216905000000	1.334000000000	1.163015000000
H	-4.091902000000	0.535445000000	-0.151607000000
H	-2.555910000000	1.346719000000	-0.472427000000
H	-3.313995000000	-0.842158000000	2.483541000000
H	-2.692698000000	-2.335032000000	1.788886000000

H	-4.171502000000	-1.610875000000	1.139945000000
H	-1.691638000000	-0.828442000000	-1.518490000000
H	-3.252732000000	-1.592434000000	-1.170400000000
H	-1.755824000000	-2.337956000000	-0.599932000000
O	-1.021160000000	4.130663000000	1.800754000000
O	-2.094118000000	2.732053000000	3.250940000000
C	-2.827188000000	3.874861000000	3.662639000000
H	-3.477892000000	3.541003000000	4.472256000000
H	-3.433608000000	4.277797000000	2.849046000000
H	-2.167044000000	4.669806000000	4.015694000000
C	2.015884000000	3.745903000000	4.777857000000
C	3.060076000000	2.087448000000	3.396912000000
C	3.097271000000	3.338035000000	4.009134000000
H	2.021488000000	4.722567000000	5.244783000000
H	3.953788000000	3.986058000000	3.879766000000
H	1.929672000000	0.301534000000	3.054373000000
H	3.887705000000	1.757813000000	2.781178000000
H	0.054609000000	3.280793000000	5.501428000000
K	0.904357000000	-1.765701000000	5.381326000000
O	-0.920245000000	-2.794872000000	7.038165000000
C	-0.898490000000	-3.436086000000	8.301353000000
H	-1.118086000000	-4.505153000000	8.202419000000
H	-1.629755000000	-2.986412000000	8.981683000000
H	0.100036000000	-3.313640000000	8.719705000000
C	-2.192077000000	-2.894101000000	6.394533000000
H	-2.116738000000	-2.380968000000	5.437038000000
H	-2.966936000000	-2.421967000000	7.007188000000
H	-2.456261000000	-3.944560000000	6.232492000000
H	1.156085000000	-3.404967000000	1.563924000000
H	-0.113290000000	-3.223847000000	2.794400000000
C	0.788984000000	-3.770482000000	2.528072000000
H	0.574576000000	-4.841363000000	2.448175000000
O	1.753462000000	-3.530665000000	3.553565000000
H	3.426187000000	-3.749214000000	2.340410000000
C	3.003845000000	-4.136920000000	3.273743000000
H	2.902913000000	-5.224812000000	3.191978000000
H	3.679150000000	-3.902049000000	4.095927000000

INT_ A-_K+_conf4

Imaginary frequency -14.52 cm^{-1} (solvent molecules vibration)

E_{el} = -2021.171683950438 Eh

G_{corr} = 0.50690405 Eh

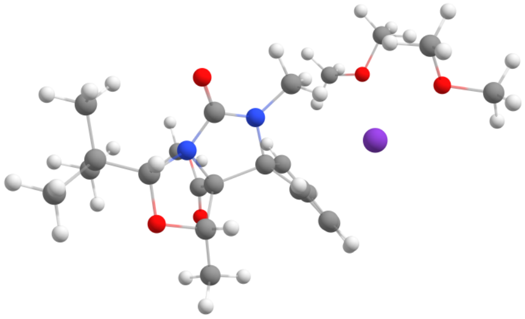

O	-0.602494000000	2.185755000000	-0.344570000000
C	0.112597000000	2.074179000000	0.892682000000
C	-1.615220000000	1.194306000000	-0.371150000000
C	-0.999132000000	1.725778000000	1.898934000000
C	0.990665000000	3.286269000000	1.084911000000
H	0.753369000000	1.188261000000	0.842460000000
N	-1.878212000000	0.931917000000	1.049999000000
C	-1.718623000000	2.915217000000	2.511114000000
H	-1.243305000000	0.266530000000	-0.828997000000
C	-2.811137000000	1.705219000000	-1.193830000000
C	-2.056684000000	-0.340242000000	1.550867000000
O	-2.588464000000	-1.269507000000	0.962469000000
N	-1.553860000000	-0.372334000000	2.827335000000
C	-1.568631000000	-1.605308000000	3.559352000000
C	-0.551553000000	0.699432000000	3.113692000000
H	-0.606616000000	-2.132935000000	3.493063000000
H	-2.331371000000	-2.246401000000	3.121580000000
H	-1.806989000000	-1.427764000000	4.611015000000
C	0.851764000000	0.180951000000	2.980260000000
C	-0.709636000000	1.277294000000	4.485715000000
H	0.417968000000	4.207526000000	1.032189000000
H	1.756718000000	3.292149000000	0.307236000000
H	1.478595000000	3.238135000000	2.058083000000
C	-3.406046000000	2.967133000000	-0.562032000000
C	-3.880537000000	0.610963000000	-1.264129000000
C	-2.303258000000	2.015297000000	-2.608842000000
H	-3.813702000000	2.748057000000	0.424112000000
H	-4.214897000000	3.348603000000	-1.189123000000
H	-2.654056000000	3.750523000000	-0.461584000000
H	-4.254578000000	0.355924000000	-0.273626000000
H	-3.485454000000	-0.305214000000	-1.706308000000
H	-4.716694000000	0.956327000000	-1.876777000000
H	-1.554941000000	2.807358000000	-2.593944000000
H	-3.133189000000	2.333767000000	-3.243345000000
H	-1.853438000000	1.130195000000	-3.066599000000
O	-1.223785000000	3.976214000000	2.791545000000
O	-3.003567000000	2.616211000000	2.794947000000
C	-3.742627000000	3.652374000000	3.447028000000
H	-4.737685000000	3.245851000000	3.609150000000
H	-3.791512000000	4.541433000000	2.818244000000
H	-3.274464000000	3.918212000000	4.395227000000
C	0.356896000000	1.771756000000	5.195790000000
C	1.894649000000	0.691958000000	3.718168000000
C	1.696105000000	1.536065000000	4.826378000000
H	0.150116000000	2.329556000000	6.105163000000
H	2.522415000000	1.975442000000	5.365930000000
H	1.055496000000	-0.502939000000	2.164549000000
H	2.904344000000	0.383316000000	3.459130000000
H	-1.721407000000	1.477435000000	4.816269000000
K	0.797603000000	-1.121217000000	5.782979000000
O	-1.442380000000	-1.637037000000	7.225937000000
C	-2.456431000000	-0.646288000000	7.337358000000

H	-2.761992000000	-0.521253000000	8.381757000000
H	-3.331512000000	-0.917159000000	6.736981000000
H	-2.041133000000	0.284313000000	6.956947000000
C	-1.882241000000	-2.920848000000	7.632276000000
H	-1.040949000000	-3.601966000000	7.516697000000
H	-2.716566000000	-3.263681000000	7.009553000000
H	-2.203665000000	-2.912788000000	8.679798000000
H	1.111788000000	-5.138825000000	4.368448000000
H	-0.331587000000	-4.155170000000	4.704986000000
C	0.480660000000	-4.719572000000	5.158676000000
H	0.063904000000	-5.538407000000	5.755330000000
O	1.221619000000	-3.825480000000	5.978364000000
H	3.011566000000	-4.882750000000	5.905869000000
C	2.307742000000	-4.462352000000	6.632214000000
H	1.953579000000	-5.264697000000	7.288915000000
H	2.818779000000	-3.710618000000	7.232349000000

GS_C-_K+

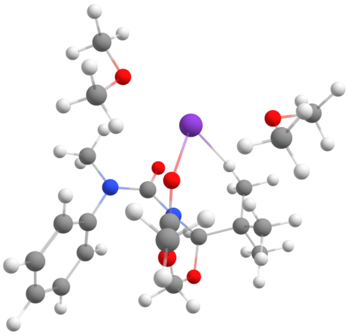

O	2.550265000000	0.668787000000	3.149678000000
C	1.952781000000	0.829306000000	1.831381000000
C	1.568149000000	0.283381000000	4.108401000000
C	0.528153000000	0.463738000000	2.031962000000
N	0.290468000000	0.548746000000	3.435676000000
C	-0.328900000000	-0.225972000000	1.219257000000
H	1.650384000000	0.938913000000	4.979887000000
C	1.803941000000	-1.173932000000	4.576455000000
C	-0.690158000000	1.294135000000	4.044669000000
O	-1.019499000000	1.087526000000	5.222460000000
N	-1.414323000000	2.164832000000	3.256239000000
C	-2.586959000000	2.784334000000	3.864059000000
C	-0.812818000000	2.899698000000	2.185556000000
H	-2.312674000000	3.579011000000	4.564668000000
H	-3.163384000000	2.038193000000	4.404483000000
H	-3.198653000000	3.210716000000	3.071122000000
C	0.292581000000	3.708603000000	2.430415000000
C	-1.402171000000	2.896459000000	0.923327000000
C	3.258262000000	-1.279696000000	5.056228000000
C	1.566364000000	-2.175588000000	3.443410000000
C	0.878502000000	-1.489718000000	5.755602000000
H	3.951643000000	-1.084334000000	4.239961000000
H	3.452680000000	-2.280052000000	5.450834000000
H	3.462348000000	-0.559660000000	5.853008000000

H	2.214702000000	-1.970276000000	2.592037000000
H	0.536067000000	-2.148989000000	3.092806000000
H	1.783841000000	-3.187422000000	3.796843000000
H	-0.168364000000	-1.361660000000	5.490181000000
H	1.065024000000	-0.821978000000	6.598394000000
H	1.032605000000	-2.518173000000	6.089592000000
O	-1.458934000000	-0.710315000000	1.533585000000
O	0.110328000000	-0.331118000000	-0.093188000000
C	-0.756042000000	-0.997422000000	-0.994380000000
H	-1.735542000000	-0.516561000000	-1.041223000000
H	-0.900938000000	-2.045782000000	-0.719981000000
H	-0.272047000000	-0.941253000000	-1.968322000000
C	-0.868173000000	3.671213000000	-0.093281000000
C	0.830653000000	4.480093000000	1.405961000000
C	0.253702000000	4.464092000000	0.143224000000
H	-1.316034000000	3.646855000000	-1.078585000000
H	0.672895000000	5.064295000000	-0.653944000000
H	0.736630000000	3.717957000000	3.417689000000
H	1.700801000000	5.094715000000	1.599096000000
H	-2.247865000000	2.247333000000	0.742804000000
K	-2.335386000000	-1.125430000000	3.944691000000
O	-4.636349000000	-0.006658000000	3.068875000000
C	-5.846121000000	0.415305000000	3.667858000000
H	-6.713198000000	0.001825000000	3.139878000000
H	-5.922456000000	1.508853000000	3.668842000000
H	-5.850622000000	0.057636000000	4.696973000000
C	-4.520078000000	0.410093000000	1.709959000000
H	-3.536784000000	0.098645000000	1.358015000000
H	-4.609979000000	1.499630000000	1.637722000000
H	-5.306125000000	-0.050458000000	1.101040000000
H	-2.059823000000	-4.837445000000	1.738373000000
H	-0.409490000000	-4.194868000000	1.965275000000
C	-1.475233000000	-3.984833000000	2.101385000000
H	-1.724728000000	-3.080909000000	1.548386000000
O	-1.761046000000	-3.733427000000	3.474910000000
H	-0.226956000000	-4.900366000000	4.241687000000
C	-1.311223000000	-4.769755000000	4.326522000000
H	-1.804194000000	-5.719191000000	4.087184000000
H	-1.556099000000	-4.487839000000	5.349986000000
H	2.123034000000	1.872773000000	1.543017000000
H	2.461127000000	0.180999000000	1.114265000000

TS_arylation_ C-_K+
Imaginary frequency -294.11 cm^{-1}

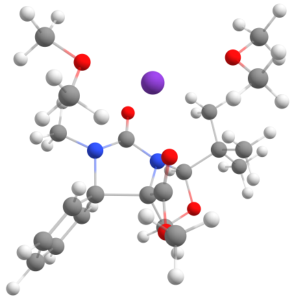

O	2.516610000000	0.151431000000	2.872721000000
C	1.922989000000	1.040868000000	1.919740000000
C	1.657198000000	0.021609000000	3.990547000000
C	0.435434000000	0.807167000000	2.073485000000
N	0.321114000000	0.351817000000	3.446788000000
C	-0.191193000000	-0.138157000000	1.126075000000
H	1.909541000000	0.760288000000	4.764245000000
C	1.829747000000	-1.399181000000	4.565136000000
C	-0.631784000000	1.082682000000	4.151056000000
O	-0.891230000000	0.894059000000	5.343345000000
N	-1.317074000000	1.877057000000	3.294678000000
C	-2.314947000000	2.794640000000	3.800330000000
C	-0.521439000000	2.301205000000	2.058342000000
H	-1.865949000000	3.711159000000	4.191384000000
H	-2.875827000000	2.310842000000	4.598802000000
H	-2.976815000000	3.066122000000	2.977959000000
C	0.284581000000	3.483938000000	2.351648000000
C	-1.344498000000	2.478355000000	0.864158000000
C	3.300470000000	-1.565317000000	4.974251000000
C	1.470725000000	-2.442689000000	3.503558000000
C	0.948618000000	-1.579212000000	5.804018000000
H	3.961828000000	-1.478770000000	4.112916000000
H	3.453170000000	-2.545379000000	5.432584000000
H	3.591076000000	-0.806356000000	5.705044000000
H	2.121400000000	-2.353568000000	2.634131000000
H	0.441602000000	-2.326082000000	3.167338000000
H	1.585811000000	-3.447301000000	3.917627000000
H	-0.094120000000	-1.353738000000	5.594420000000
H	1.247008000000	-0.898247000000	6.603271000000
H	1.033606000000	-2.602725000000	6.175890000000
O	-1.106839000000	-0.913304000000	1.396864000000
O	0.240815000000	0.039375000000	-0.127825000000
C	-0.463582000000	-0.654529000000	-1.162628000000
H	-1.514253000000	-0.365800000000	-1.164596000000
H	-0.384997000000	-1.733186000000	-1.024159000000
H	0.011283000000	-0.353266000000	-2.092150000000
C	-1.222248000000	3.567277000000	0.033785000000
C	0.376358000000	4.559904000000	1.493470000000
C	-0.347413000000	4.626753000000	0.302417000000
H	-1.840344000000	3.600641000000	-0.858596000000

H	-0.269284000000	5.476746000000	-0.360498000000
H	0.823588000000	3.504447000000	3.293581000000
H	1.018559000000	5.387582000000	1.778461000000
H	-2.038889000000	1.688182000000	0.604388000000
K	-2.369267000000	-1.091719000000	3.758117000000
O	-4.604595000000	-0.013849000000	2.751428000000
C	-5.625158000000	0.712017000000	3.416404000000
H	-6.608176000000	0.489450000000	2.986019000000
H	-5.442741000000	1.789910000000	3.353083000000
H	-5.617461000000	0.409381000000	4.463150000000
C	-4.491278000000	0.343483000000	1.378115000000
H	-3.642140000000	-0.201323000000	0.968457000000
H	-4.307416000000	1.416363000000	1.270503000000
H	-5.403358000000	0.073120000000	0.834462000000
H	-2.197943000000	-5.113917000000	2.219319000000
H	-0.523004000000	-4.507724000000	2.349650000000
C	-1.579640000000	-4.235079000000	2.433053000000
H	-1.792177000000	-3.446273000000	1.713341000000
O	-1.865850000000	-3.722156000000	3.728754000000
H	-0.442552000000	-4.838604000000	4.745578000000
C	-1.516515000000	-4.626051000000	4.763044000000
H	-2.070344000000	-5.566511000000	4.663846000000
H	-1.770454000000	-4.157406000000	5.712812000000
H	2.158658000000	2.081866000000	2.160390000000
H	2.313786000000	0.800484000000	0.936752000000

TS_elimination_ C-_K+
Imaginary frequency -332.42 cm^{-1}

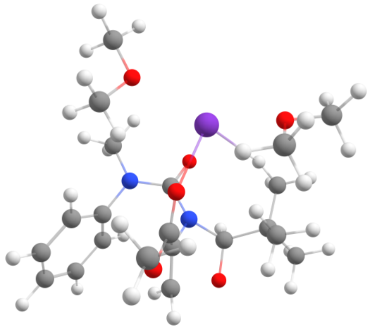

O	2.776624000000	0.529456000000	3.222344000000
C	1.871017000000	1.117574000000	1.574651000000
C	1.744466000000	0.241962000000	4.068053000000
C	0.607055000000	0.607699000000	1.948438000000
N	0.426019000000	0.657199000000	3.353419000000
C	-0.236823000000	-0.223915000000	1.162720000000
H	1.754451000000	0.868477000000	4.998835000000
C	1.790297000000	-1.269590000000	4.514578000000
C	-0.603750000000	1.277897000000	4.014125000000
O	-0.917984000000	1.004762000000	5.196198000000
N	-1.396646000000	2.165958000000	3.268210000000
C	-2.568891000000	2.731407000000	3.925869000000
C	-0.843887000000	2.973542000000	2.219124000000
H	-2.314826000000	3.619367000000	4.548138000000

H	-3.025903000000	1.977529000000	4.591492000000
H	-3.301730000000	3.042675000000	3.156308000000
C	0.215334000000	3.858110000000	2.497194000000
C	-1.423754000000	2.960990000000	0.936421000000
C	3.231303000000	-1.535467000000	4.995228000000
C	1.475729000000	-2.189024000000	3.326356000000
C	0.844018000000	-1.556318000000	5.689256000000
H	3.951890000000	-1.345972000000	4.177558000000
H	3.337580000000	-2.586556000000	5.339466000000
H	3.500061000000	-0.871483000000	5.843894000000
H	2.137892000000	-1.952003000000	2.470180000000
H	0.428854000000	-2.076066000000	2.985893000000
H	1.638430000000	-3.252169000000	3.604490000000
H	-0.217577000000	-1.408956000000	5.424901000000
H	1.041561000000	-0.876078000000	6.542226000000
H	0.972225000000	-2.601454000000	6.042944000000
O	-1.282861000000	-0.810310000000	1.550866000000
O	0.129741000000	-0.282265000000	-0.164705000000
C	-0.667155000000	-1.108817000000	-1.001649000000
H	-1.727486000000	-0.779128000000	-1.012381000000
H	-0.638041000000	-2.171306000000	-0.677664000000
H	-0.238626000000	-1.019462000000	-2.017034000000
C	-0.926142000000	3.799818000000	-0.068356000000
C	0.717564000000	4.691535000000	1.485586000000
C	0.150896000000	4.663837000000	0.201434000000
H	-1.369831000000	3.769861000000	-1.075150000000
H	0.546742000000	5.316849000000	-0.590983000000
H	0.652853000000	3.870675000000	3.506827000000
H	1.557336000000	5.368798000000	1.704274000000
H	-2.242850000000	2.257201000000	0.730922000000
K	-2.309722000000	-1.103214000000	3.945789000000
O	-4.587169000000	-0.043447000000	2.947231000000
C	-5.783571000000	0.447577000000	3.522996000000
H	-6.682882000000	0.046932000000	2.999160000000
H	-5.822254000000	1.561688000000	3.490012000000
H	-5.811868000000	0.121278000000	4.580708000000
C	-4.439541000000	0.311644000000	1.576546000000
H	-3.452144000000	-0.062307000000	1.240258000000
H	-4.473013000000	1.418341000000	1.448260000000
H	-5.249297000000	-0.141042000000	0.958104000000
H	-2.165532000000	-5.058025000000	2.037810000000
H	-0.514791000000	-4.312699000000	2.138041000000
C	-1.601489000000	-4.133496000000	2.302638000000
H	-1.914319000000	-3.296122000000	1.650464000000
O	-1.855684000000	-3.748903000000	3.648590000000
H	-0.257975000000	-4.807711000000	4.495190000000
C	-1.360389000000	-4.680666000000	4.594585000000
H	-1.849084000000	-5.676460000000	4.478998000000
H	-1.580997000000	-4.287489000000	5.605687000000
H	2.159977000000	2.140975000000	1.851836000000
H	2.369620000000	0.709098000000	0.680965000000

7.5.3 Potassium Enolates with Additional K^+ (Cationic; Charge +1)

GS_A$^-$·2K$^+$·4Me$_2$O

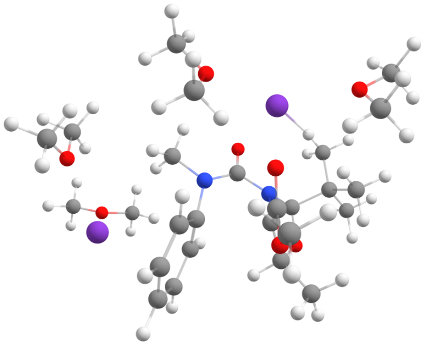

O	2.646896000000	0.805441000000	2.995385000000
C	1.989096000000	1.157386000000	1.737322000000
C	1.708751000000	0.459935000000	4.009745000000
C	0.550082000000	0.816289000000	1.988681000000
C	2.711385000000	0.493703000000	0.575365000000
H	2.112079000000	2.267583000000	1.634774000000
N	0.394799000000	0.776472000000	3.407289000000
C	-0.421652000000	0.187526000000	1.184577000000
H	1.851024000000	1.119389000000	4.896525000000
C	1.909199000000	-1.014620000000	4.464781000000
C	-0.519025000000	1.586162000000	4.062363000000
O	-0.803999000000	1.443127000000	5.267048000000
N	-1.216104000000	2.477012000000	3.246437000000
C	-2.307252000000	3.225366000000	3.842260000000
C	-0.656794000000	3.010851000000	2.031749000000
H	-1.980115000000	4.221458000000	4.223011000000
H	-2.700668000000	2.658862000000	4.704931000000
H	-3.115932000000	3.376717000000	3.099039000000
C	0.446304000000	3.913283000000	2.107087000000
C	-1.442168000000	3.006602000000	0.842412000000
H	3.792926000000	0.721468000000	0.638238000000
H	2.321602000000	0.878940000000	-0.385249000000
H	2.578225000000	-0.605043000000	0.586703000000
C	3.392902000000	-1.186725000000	4.843811000000
C	1.543239000000	-1.991289000000	3.336259000000
C	1.049366000000	-1.285970000000	5.710524000000
H	4.048589000000	-1.004914000000	3.971584000000
H	3.576901000000	-2.215257000000	5.215567000000
H	3.689326000000	-0.480461000000	5.647234000000
H	2.176595000000	-1.821577000000	2.444878000000
H	0.485821000000	-1.887454000000	3.026053000000
H	1.702633000000	-3.036849000000	3.670002000000
H	-0.028710000000	-1.136223000000	5.515442000000
H	1.311364000000	-0.604100000000	6.544236000000
H	1.195559000000	-2.328308000000	6.058903000000
O	-1.511215000000	-0.311293000000	1.594270000000
O	-0.170383000000	0.271863000000	-0.167654000000
C	-1.098619000000	-0.378459000000	-1.028580000000
H	-2.123668000000	0.031946000000	-0.915448000000

H	-1.139113000000	-1.470824000000	-0.833640000000
H	-0.735613000000	-0.200873000000	-2.057399000000
C	-1.044526000000	3.747835000000	-0.274332000000
C	0.836536000000	4.656419000000	0.978996000000
C	0.102765000000	4.578398000000	-0.221195000000
H	-1.623525000000	3.672111000000	-1.207856000000
H	0.425517000000	5.141142000000	-1.109580000000
H	1.022139000000	3.974278000000	3.043620000000
H	1.728234000000	5.300396000000	1.039015000000
H	-2.321371000000	2.347701000000	0.796729000000
K	-2.239542000000	-0.824711000000	4.095303000000
O	-4.494631000000	0.547759000000	3.453777000000
C	-5.585700000000	1.072860000000	4.187348000000
H	-6.547371000000	0.590898000000	3.895843000000
H	-5.682956000000	2.174298000000	4.031941000000
H	-5.399303000000	0.881992000000	5.261647000000
C	-4.624800000000	0.722349000000	2.047758000000
H	-3.699507000000	0.332582000000	1.578600000000
H	-4.735811000000	1.802861000000	1.790157000000
H	-5.513385000000	0.176989000000	1.654555000000
H	-2.367921000000	-4.467567000000	1.800255000000
H	-0.612761000000	-4.036123000000	1.907529000000
C	-1.646473000000	-3.684647000000	2.128678000000
H	-1.826066000000	-2.743445000000	1.573687000000
O	-1.798860000000	-3.398730000000	3.516220000000
H	-0.408274000000	-4.812961000000	4.196277000000
C	-1.466231000000	-4.498508000000	4.349193000000
H	-2.129147000000	-5.372654000000	4.153640000000
H	-1.594811000000	-4.179659000000	5.401590000000
H	-5.133116000000	5.261321000000	0.595925000000
H	-6.356308000000	5.988965000000	1.709355000000
H	0.554690000000	7.368808000000	5.070439000000
H	-1.062065000000	9.204387000000	4.672469000000
C	-5.486465000000	5.297215000000	1.644201000000
H	-5.818854000000	4.277201000000	1.945904000000
C	-0.292117000000	6.656964000000	4.947581000000
C	-1.843356000000	8.415666000000	4.588442000000
O	-1.297994000000	7.197613000000	4.092294000000
H	0.083291000000	5.727178000000	4.480422000000
H	-2.621444000000	8.754932000000	3.877291000000
O	-4.406529000000	5.746141000000	2.454267000000
H	-0.707290000000	6.414753000000	5.951692000000
H	-2.310407000000	8.271021000000	5.589343000000
K	-1.874816000000	6.132948000000	1.692314000000
C	-4.745278000000	5.832152000000	3.834236000000
H	-5.573497000000	6.556815000000	4.002507000000
H	-5.052794000000	4.839201000000	4.235387000000
H	-3.844199000000	6.173388000000	4.378130000000

TS_arylation_A^{-}·2K^{+}·4Me$_2$O

Imaginary frequency -289.59 cm^{-1}

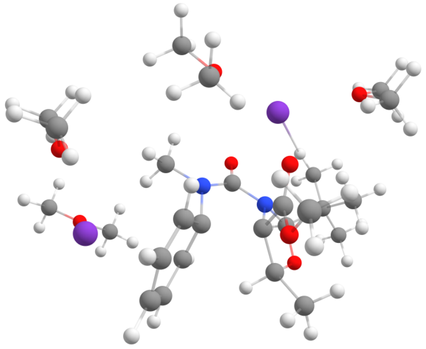

O	2.572078000000	0.617647000000	2.782569000000
C	1.907070000000	1.295704000000	1.696173000000
C	1.715551000000	0.390597000000	3.882981000000
C	0.428668000000	1.100469000000	1.983988000000
C	2.503779000000	0.806455000000	0.394904000000
H	2.122674000000	2.365992000000	1.792703000000
N	0.381326000000	0.779432000000	3.385295000000
C	-0.453394000000	0.261366000000	1.175397000000
H	1.970924000000	1.048698000000	4.723247000000
C	1.876303000000	-1.077860000000	4.337468000000
C	-0.499461000000	1.554438000000	4.112396000000
O	-0.752890000000	1.376691000000	5.304798000000
N	-1.148979000000	2.443136000000	3.306704000000
C	-2.151379000000	3.315651000000	3.870631000000
C	-0.490201000000	2.806661000000	2.039794000000
H	-1.732596000000	4.268680000000	4.213754000000
H	-2.597678000000	2.825043000000	4.732184000000
H	-2.920652000000	3.517921000000	3.124305000000
C	0.491619000000	3.875997000000	2.165796000000
C	-1.383741000000	2.999681000000	0.907447000000
H	3.583331000000	0.958114000000	0.429998000000
H	2.100075000000	1.371691000000	-0.443562000000
H	2.307285000000	-0.252812000000	0.230457000000
C	3.367443000000	-1.313963000000	4.621443000000
C	1.406004000000	-2.028112000000	3.233504000000
C	1.093153000000	-1.315694000000	5.631033000000
H	3.965256000000	-1.178180000000	3.721743000000
H	3.521809000000	-2.330050000000	4.990949000000
H	3.738203000000	-0.623230000000	5.383240000000
H	1.964466000000	-1.855036000000	2.313200000000
H	0.345993000000	-1.903846000000	3.014882000000
H	1.571524000000	-3.064533000000	3.535076000000
H	0.030409000000	-1.107481000000	5.521131000000
H	1.449686000000	-0.669600000000	6.435320000000
H	1.209417000000	-2.352557000000	5.952305000000
O	-1.399864000000	-0.389356000000	1.621112000000
O	-0.263302000000	0.409641000000	-0.140060000000
C	-1.180583000000	-0.257829000000	-1.014321000000
H	-2.197523000000	0.097541000000	-0.848635000000
H	-1.148017000000	-1.335642000000	-0.856042000000

H	-0.854908000000	-0.013367000000	-2.021490000000
C	-1.046909000000	3.826283000000	-0.140891000000
C	0.815880000000	4.693135000000	1.096539000000
C	0.080069000000	4.668668000000	-0.093473000000
H	-1.683252000000	3.837501000000	-1.019985000000
H	0.356790000000	5.283528000000	-0.938384000000
H	1.062068000000	3.934367000000	3.085762000000
H	1.646462000000	5.384062000000	1.201437000000
H	-2.264478000000	2.372678000000	0.842451000000
K	-2.226928000000	-0.791446000000	4.137332000000
O	-4.418338000000	0.636124000000	3.470479000000
C	-5.441681000000	1.258143000000	4.227336000000
H	-6.424007000000	0.837761000000	3.985928000000
H	-5.462857000000	2.338388000000	4.040558000000
H	-5.227160000000	1.086201000000	5.281232000000
C	-4.593506000000	0.791181000000	2.068968000000
H	-3.738350000000	0.326566000000	1.580884000000
H	-4.624218000000	1.852501000000	1.797526000000
H	-5.520797000000	0.313229000000	1.734858000000
H	-2.427249000000	-4.741645000000	2.285077000000
H	-0.746507000000	-4.155238000000	2.163375000000
C	-1.771216000000	-3.877076000000	2.430570000000
H	-2.090797000000	-3.058228000000	1.787464000000
O	-1.833616000000	-3.415377000000	3.774589000000
H	-0.304409000000	-4.624110000000	4.495302000000
C	-1.349079000000	-4.372316000000	4.705036000000
H	-1.950209000000	-5.287219000000	4.677315000000
H	-1.414782000000	-3.930372000000	5.698543000000
H	-4.433013000000	4.779816000000	0.341095000000
H	-5.912301000000	5.661400000000	0.808532000000
H	0.556596000000	7.386261000000	5.287580000000
H	-1.324353000000	8.952153000000	5.246076000000
C	-5.115007000000	4.999496000000	1.161648000000
H	-5.561682000000	4.062951000000	1.512125000000
C	-0.158570000000	6.588615000000	5.063131000000
C	-1.974728000000	8.103853000000	5.009602000000
O	-1.260298000000	7.092460000000	4.312367000000
H	0.328978000000	5.827057000000	4.456576000000
H	-2.781819000000	8.445972000000	4.362077000000
O	-4.356362000000	5.615627000000	2.193441000000
H	-0.500029000000	6.138150000000	6.000749000000
H	-2.399474000000	7.712234000000	5.940334000000
K	-1.736877000000	6.107984000000	1.861608000000
C	-5.119064000000	5.870480000000	3.363825000000
H	-5.948562000000	6.553605000000	3.154136000000
H	-5.521900000000	4.938638000000	3.775784000000
H	-4.454034000000	6.323536000000	4.097775000000

TS_elimination_A$^-$·2K$^+$·4Me$_2$O

Imaginary frequency -308.92 cm^{-1}; -27.92 cm^{-1} (could not be removed by further optimization)

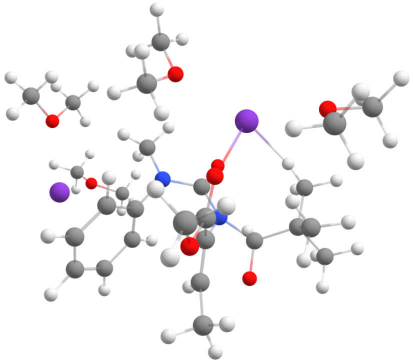

O	2.923713000000	0.656606000000	3.081863000000
C	1.892022000000	1.276090000000	1.288229000000
C	1.951083000000	0.452798000000	3.937102000000
C	0.687400000000	0.830265000000	1.793758000000
C	2.604085000000	0.805367000000	0.071182000000
H	2.301811000000	2.168703000000	1.732946000000
N	0.567493000000	0.965874000000	3.190096000000
C	-0.293288000000	0.032195000000	1.121092000000
H	1.946031000000	1.111125000000	4.825586000000
C	1.857171000000	-1.034184000000	4.422937000000
C	-0.402781000000	1.626012000000	3.840360000000
O	-0.699348000000	1.430672000000	5.029101000000
N	-1.189769000000	2.538805000000	3.098643000000
C	-2.303239000000	3.180365000000	3.775407000000
C	-0.690713000000	3.235813000000	1.968265000000
H	-2.011158000000	4.132433000000	4.241320000000
H	-2.663771000000	2.532124000000	4.567307000000
H	-3.109829000000	3.372141000000	3.065007000000
C	0.447512000000	4.044300000000	2.064981000000
C	-1.390500000000	3.186645000000	0.758203000000
H	3.677544000000	0.909145000000	0.236329000000
H	2.340960000000	1.425214000000	-0.793637000000
H	2.376406000000	-0.228394000000	-0.176179000000
C	3.286220000000	-1.417950000000	4.845928000000
C	1.417825000000	-1.961725000000	3.289776000000
C	0.963404000000	-1.204705000000	5.653318000000
H	3.968707000000	-1.335432000000	4.002394000000
H	3.303467000000	-2.443253000000	5.227172000000
H	3.650907000000	-0.757776000000	5.637556000000
H	2.036873000000	-1.793292000000	2.406894000000
H	0.376171000000	-1.806805000000	3.007785000000
H	1.532876000000	-3.007581000000	3.588710000000
H	-0.087101000000	-1.008300000000	5.455166000000
H	1.257672000000	-0.518528000000	6.449935000000
H	1.046278000000	-2.225173000000	6.037398000000
O	-1.313349000000	-0.439659000000	1.642810000000
O	-0.095838000000	-0.083475000000	-0.215103000000
C	-1.074606000000	-0.818679000000	-0.949135000000
H	-2.047074000000	-0.323598000000	-0.904336000000
H	-1.175807000000	-1.834104000000	-0.564784000000

H	-0.715399000000	-0.840707000000	-1.975399000000
C	-0.939491000000	3.899476000000	-0.347521000000
C	0.904187000000	4.750018000000	0.953631000000
C	0.212638000000	4.682838000000	-0.254975000000
H	-1.465602000000	3.816374000000	-1.291077000000
H	0.580985000000	5.214471000000	-1.123596000000
H	1.004375000000	4.061427000000	2.993857000000
H	1.812403000000	5.336469000000	1.026179000000
H	-2.255455000000	2.539947000000	0.684800000000
K	-2.172477000000	-0.750683000000	4.103539000000
O	-4.383022000000	0.579199000000	3.250631000000
C	-5.517515000000	1.093483000000	3.922836000000
H	-6.434323000000	0.588336000000	3.598633000000
H	-5.624567000000	2.170544000000	3.739426000000
H	-5.375291000000	0.928281000000	4.990356000000
C	-4.459409000000	0.730705000000	1.840374000000
H	-3.534000000000	0.336194000000	1.422694000000
H	-4.561264000000	1.790757000000	1.572021000000
H	-5.318015000000	0.183851000000	1.434647000000
H	-2.401934000000	-4.736152000000	2.298836000000
H	-0.719734000000	-4.136488000000	2.265996000000
C	-1.760258000000	-3.865254000000	2.472977000000
H	-2.049701000000	-3.052394000000	1.808025000000
O	-1.900916000000	-3.394787000000	3.807844000000
H	-0.373593000000	-4.558527000000	4.600974000000
C	-1.431859000000	-4.331452000000	4.766934000000
H	-2.011194000000	-5.260371000000	4.722286000000
H	-1.548291000000	-3.881868000000	5.752553000000
H	-5.162613000000	5.389711000000	0.653290000000
H	-6.384224000000	6.050106000000	1.767938000000
H	0.414069000000	7.223163000000	5.347595000000
H	-1.131738000000	9.120939000000	5.087762000000
C	-5.518691000000	5.385537000000	1.682990000000
H	-5.821301000000	4.368372000000	1.954866000000
C	-0.433659000000	6.558555000000	5.155673000000
C	-1.924384000000	8.384786000000	4.921233000000
O	-1.401030000000	7.201627000000	4.324563000000
H	-0.085754000000	5.670762000000	4.630320000000
H	-2.664200000000	8.802020000000	4.237756000000
O	-4.451867000000	5.832628000000	2.512261000000
H	-0.881578000000	6.259039000000	6.108031000000
H	-2.407240000000	8.157784000000	5.877438000000
K	-1.927713000000	6.358439000000	1.872369000000
C	-4.806592000000	5.854110000000	3.892151000000
H	-5.642882000000	6.538238000000	4.067284000000
H	-5.083871000000	4.852485000000	4.237172000000
H	-3.934169000000	6.193618000000	4.448548000000

GS_B$^-$·2K$^+$·4Me$_2$O

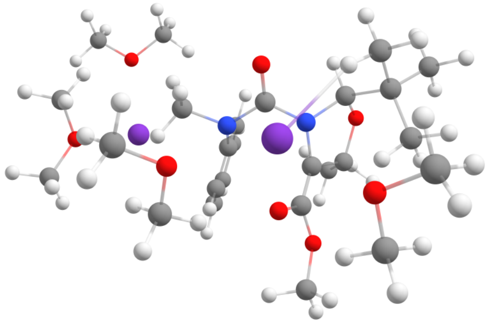

O	2.689808000000	0.791388000000	2.909727000000
C	2.083708000000	0.673414000000	1.597734000000
C	1.749092000000	0.508350000000	3.932845000000
C	0.608448000000	0.693301000000	1.893172000000
C	2.658091000000	1.762274000000	0.710070000000
N	0.465562000000	0.863106000000	3.303953000000
C	-0.398208000000	0.115966000000	1.156757000000
H	1.934427000000	1.193055000000	4.763380000000
C	1.887645000000	-0.939478000000	4.467644000000
C	-0.451377000000	1.648514000000	3.934555000000
O	-0.732000000000	1.518364000000	5.134351000000
N	-1.191218000000	2.519813000000	3.135774000000
C	-2.311636000000	3.203330000000	3.759379000000
C	-0.609466000000	3.210850000000	2.031254000000
H	-2.002868000000	4.116126000000	4.286672000000
H	-2.782559000000	2.548549000000	4.484789000000
H	-3.041836000000	3.465359000000	2.991815000000
C	0.445881000000	4.102577000000	2.244825000000
C	-1.200002000000	3.132406000000	0.767038000000
C	3.355736000000	-1.139836000000	4.870650000000
C	1.499213000000	-1.984814000000	3.417914000000
C	1.017771000000	-1.111166000000	5.717862000000
H	4.015705000000	-1.034038000000	4.011479000000
H	3.494589000000	-2.135085000000	5.297641000000
H	3.661530000000	-0.408314000000	5.622721000000
H	2.164825000000	-1.951625000000	2.556259000000
H	0.480390000000	-1.845181000000	3.060375000000
H	1.573572000000	-2.983508000000	3.854276000000
H	-0.040302000000	-0.975282000000	5.503671000000
H	1.272917000000	-0.378758000000	6.485101000000
H	1.158282000000	-2.109227000000	6.136775000000
O	-1.570394000000	-0.165120000000	1.544258000000
O	-0.047461000000	-0.096246000000	-0.165882000000
C	-0.999749000000	-0.772363000000	-0.974497000000
H	-1.954711000000	-0.244440000000	-0.998177000000
H	-1.177486000000	-1.790167000000	-0.619606000000
H	-0.569619000000	-0.806739000000	-1.973347000000
C	-0.717400000000	3.909657000000	-0.278630000000
C	0.932694000000	4.878858000000	1.191498000000
C	0.353820000000	4.786328000000	-0.071301000000
H	-1.150176000000	3.808518000000	-1.265834000000

H	0.747384000000	5.369123000000	-0.894117000000
H	0.909901000000	4.146128000000	3.222267000000
H	1.776549000000	5.537645000000	1.355074000000
H	-1.999281000000	2.419245000000	0.614749000000
K	-2.232329000000	-0.744361000000	4.032364000000
O	-4.503752000000	0.646167000000	3.497031000000
C	-5.644235000000	1.042216000000	4.234426000000
H	-6.544869000000	0.530575000000	3.878466000000
H	-5.802446000000	2.125301000000	4.157274000000
H	-5.472651000000	0.783004000000	5.278083000000
C	-4.611633000000	0.930114000000	2.106469000000
H	-3.678584000000	0.618234000000	1.637976000000
H	-4.762744000000	2.005459000000	1.947858000000
H	-5.455830000000	0.389314000000	1.665878000000
H	-2.301453000000	-4.444992000000	1.798151000000
H	-0.607418000000	-3.898083000000	1.918195000000
C	-1.648736000000	-3.626252000000	2.118046000000
H	-1.888964000000	-2.717062000000	1.569237000000
O	-1.835734000000	-3.347337000000	3.502341000000
H	-0.391532000000	-4.670627000000	4.192190000000
C	-1.449574000000	-4.428504000000	4.335526000000
H	-2.051058000000	-5.319380000000	4.126468000000
H	-1.608805000000	-4.123402000000	5.368932000000
H	-5.351536000000	5.606144000000	0.683594000000
H	-6.522982000000	6.175307000000	1.896370000000
H	0.196460000000	7.583462000000	5.314031000000
H	-1.543460000000	9.298393000000	5.040753000000
C	-5.672104000000	5.510968000000	1.719799000000
H	-5.979499000000	4.477556000000	1.907958000000
C	-0.569718000000	6.827009000000	5.122127000000
C	-2.251139000000	8.478469000000	4.888375000000
O	-1.605405000000	7.355985000000	4.294429000000
H	-0.123124000000	5.987132000000	4.593352000000
H	-3.039106000000	8.804153000000	4.210504000000
O	-4.570500000000	5.867785000000	2.549060000000
H	-0.977642000000	6.476344000000	6.074445000000
H	-2.695041000000	8.206282000000	5.850800000000
K	-2.072398000000	6.412115000000	1.868303000000
C	-4.883128000000	5.772927000000	3.936091000000
H	-5.703303000000	6.448895000000	4.195087000000
H	-5.165238000000	4.749084000000	4.199721000000
H	-3.990885000000	6.053182000000	4.492511000000
H	2.189892000000	1.707843000000	-0.273199000000
H	3.735252000000	1.626863000000	0.592355000000
H	2.476405000000	2.745675000000	1.136369000000
H	2.364472000000	-0.293333000000	1.161142000000

TS_arylation_B^{-}·2K^{+}·4Me$_2$O
Imaginary frequency -339.44 cm^{-1}.

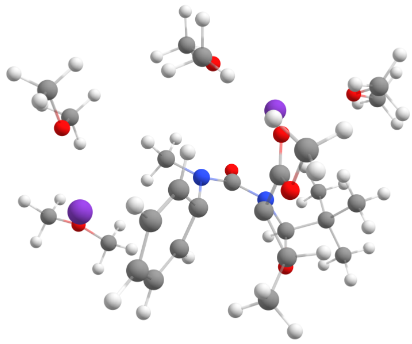

O	2.501394000000	1.070023000000	3.031642000000
C	1.894515000000	0.959675000000	1.729046000000
C	1.625002000000	0.537676000000	4.016701000000
C	0.384561000000	1.031408000000	2.037006000000
C	2.539636000000	1.951403000000	0.773572000000
N	0.279214000000	0.777167000000	3.453858000000
C	-0.514929000000	0.226552000000	1.214290000000
H	1.747718000000	1.150103000000	4.936189000000
C	1.916828000000	-0.944694000000	4.398243000000
C	-0.657773000000	1.572046000000	4.111278000000
O	-0.976739000000	1.420902000000	5.305422000000
N	-1.271684000000	2.438321000000	3.239384000000
C	-2.304263000000	3.313544000000	3.742676000000
C	-0.508882000000	2.831912000000	2.030081000000
H	-1.896740000000	4.261367000000	4.164151000000
H	-2.841842000000	2.797972000000	4.559096000000
H	-3.016514000000	3.557792000000	2.929598000000
C	0.452163000000	3.906211000000	2.279611000000
C	-1.301328000000	3.035485000000	0.820487000000
C	3.414073000000	-1.057455000000	4.738137000000
C	1.560798000000	-1.923814000000	3.265119000000
C	1.088969000000	-1.283107000000	5.651071000000
H	4.041193000000	-0.826777000000	3.855784000000
H	3.655365000000	-2.083025000000	5.083508000000
H	3.698955000000	-0.353902000000	5.547640000000
H	2.209658000000	-1.782132000000	2.379900000000
H	0.505876000000	-1.815683000000	2.945377000000
H	1.705388000000	-2.968077000000	3.609216000000
H	0.005036000000	-1.143309000000	5.478561000000
H	1.356759000000	-0.630905000000	6.506355000000
H	1.260060000000	-2.335624000000	5.954254000000
O	-1.562959000000	-0.328277000000	1.598408000000
O	-0.162991000000	0.259836000000	-0.094390000000
C	-1.033935000000	-0.399323000000	-1.019421000000
H	-2.053456000000	0.033579000000	-0.982420000000
H	-1.099135000000	-1.483484000000	-0.797290000000
H	-0.590088000000	-0.242807000000	-2.018072000000
C	-0.898647000000	3.928091000000	-0.172669000000
C	0.832284000000	4.803878000000	1.277904000000
C	0.192688000000	4.816060000000	0.016711000000

H	-1.461789000000	3.954617000000	-1.120276000000
H	0.520535000000	5.494119000000	-0.784062000000
H	0.985177000000	3.899431000000	3.244169000000
H	1.645193000000	5.519903000000	1.485677000000
H	-2.165903000000	2.374878000000	0.651179000000
K	-2.345062000000	-0.791063000000	4.138582000000
O	-4.569777000000	0.591727000000	3.420983000000
C	-5.641972000000	1.127037000000	4.177128000000
H	-6.623960000000	0.725955000000	3.835680000000
H	-5.670399000000	2.240146000000	4.103297000000
H	-5.487808000000	0.846332000000	5.236631000000
C	-4.663055000000	0.889594000000	2.033073000000
H	-3.774962000000	0.452562000000	1.538542000000
H	-4.664793000000	1.991546000000	1.861181000000
H	-5.591784000000	0.460875000000	1.591478000000
H	-2.336295000000	-4.679281000000	2.144163000000
H	-0.650427000000	-4.024700000000	2.052616000000
C	-1.701341000000	-3.783970000000	2.333697000000
H	-2.049296000000	-2.940223000000	1.707660000000
O	-1.784926000000	-3.373986000000	3.694093000000
H	-0.215962000000	-4.575367000000	4.397044000000
C	-1.288114000000	-4.350317000000	4.598366000000
H	-1.871019000000	-5.297411000000	4.533676000000
H	-1.379080000000	-3.941702000000	5.623014000000
H	-5.040069000000	5.496720000000	0.429246000000
H	-6.317235000000	6.176624000000	1.512623000000
H	0.403323000000	7.437145000000	5.275582000000
H	-1.438728000000	9.069758000000	5.085645000000
C	-5.476369000000	5.449827000000	1.445230000000
H	-5.876883000000	4.423972000000	1.616419000000
C	-0.343920000000	6.635160000000	5.081079000000
C	-2.116141000000	8.202425000000	4.915646000000
O	-1.428750000000	7.123314000000	4.294303000000
H	0.140876000000	5.818147000000	4.513367000000
H	-2.937052000000	8.515530000000	4.241943000000
O	-4.447302000000	5.760116000000	2.376412000000
H	-0.703482000000	6.235272000000	6.056255000000
H	-2.549447000000	7.895108000000	5.895150000000
K	-1.831439000000	6.135766000000	1.820899000000
C	-4.893479000000	5.731588000000	3.726324000000
H	-5.685332000000	6.493293000000	3.908058000000
H	-5.298267000000	4.728395000000	3.995022000000
H	-4.021900000000	5.948136000000	4.372430000000
H	1.966774000000	1.983337000000	-0.171766000000
H	3.571579000000	1.621216000000	0.546945000000
H	2.582172000000	2.966329000000	1.197728000000
H	2.095378000000	-0.057013000000	1.317659000000

TS_elimination_B⁻·2K⁺·4Me₂O

Imaginary frequency -304.69 cm^{-1}; -14.3 (could not be removed by further optimization)

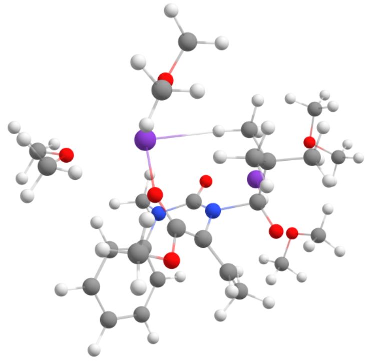

O	1.423124000000	2.558685000000	3.497861000000
C	1.185207000000	2.534256000000	1.351143000000
C	0.181734000000	2.376636000000	3.916832000000
C	0.039446000000	1.758399000000	1.536623000000
C	1.244471000000	4.026094000000	1.336289000000
N	-0.762756000000	2.176616000000	2.621155000000
C	-0.111659000000	0.420334000000	1.012854000000
H	-0.279310000000	3.285145000000	4.402857000000
C	0.043321000000	1.175102000000	4.932912000000
C	-2.016502000000	2.696641000000	2.580793000000
O	-2.601569000000	3.115004000000	3.619368000000
N	-2.746481000000	2.672477000000	1.378916000000
C	-4.119242000000	3.180932000000	1.434826000000
C	-2.158418000000	2.756684000000	0.063542000000
H	-4.163225000000	4.292392000000	1.386976000000
H	-4.606454000000	2.866326000000	2.376222000000
H	-4.676129000000	2.779189000000	0.567140000000
C	-1.510756000000	3.936692000000	-0.342654000000
C	-2.363457000000	1.716744000000	-0.861170000000
C	1.011751000000	1.497151000000	6.088764000000
C	0.467108000000	-0.134041000000	4.254731000000
C	-1.367052000000	1.032969000000	5.523006000000
H	2.045061000000	1.600796000000	5.708177000000
H	0.987477000000	0.694283000000	6.855275000000
H	0.733504000000	2.450218000000	6.588584000000
H	1.441072000000	0.003510000000	3.745467000000
H	-0.274872000000	-0.457281000000	3.501197000000
H	0.575225000000	-0.946908000000	5.002779000000
H	-2.130533000000	0.830950000000	4.749645000000
H	-1.687516000000	1.954323000000	6.050116000000
H	-1.397687000000	0.203666000000	6.260053000000
O	-1.062691000000	-0.353527000000	1.257524000000
O	0.851350000000	0.073605000000	0.116489000000
C	0.730842000000	-1.210564000000	-0.493675000000
H	-0.194446000000	-1.274810000000	-1.102503000000
H	0.719043000000	-2.019436000000	0.264965000000
H	1.614609000000	-1.320232000000	-1.146525000000
C	-1.857358000000	1.830472000000	-2.163574000000
C	-1.003630000000	4.045962000000	-1.644786000000

C	-1.166282000000	2.989689000000	-2.555347000000
H	-2.002984000000	1.008451000000	-2.880825000000
H	-0.766991000000	3.075760000000	-3.576073000000
H	-1.394562000000	4.763415000000	0.372931000000
H	-0.482303000000	4.964541000000	-1.953658000000
H	-2.904490000000	0.816674000000	-0.540832000000
K	-3.262594000000	-0.192343000000	2.573146000000
O	-4.427234000000	-0.983783000000	0.279715000000
C	-5.734215000000	-0.807126000000	-0.249226000000
H	-6.294944000000	-1.769227000000	-0.277994000000
H	-5.696923000000	-0.384735000000	-1.279291000000
H	-6.272539000000	-0.097328000000	0.408065000000
C	-3.632259000000	-1.886545000000	-0.485151000000
H	-2.611944000000	-1.873104000000	-0.056691000000
H	-3.576233000000	-1.563599000000	-1.550144000000
H	-4.048979000000	-2.918783000000	-0.449709000000
H	-1.767578000000	-4.201947000000	3.108368000000
H	-0.492803000000	-3.116290000000	3.801543000000
C	-1.448999000000	-3.141822000000	3.231933000000
H	-1.274657000000	-2.679955000000	2.241787000000
O	-2.455347000000	-2.378861000000	3.891975000000
H	-1.728439000000	-2.666801000000	5.836678000000
C	-2.659808000000	-2.784943000000	5.236485000000
H	-2.990374000000	-3.847303000000	5.295522000000
H	-3.448986000000	-2.140925000000	5.670330000000
H	-2.280100000000	2.653588000000	8.360051000000
H	-1.857701000000	3.601703000000	9.839523000000
H	1.722950000000	4.734397000000	4.425518000000
H	0.707703000000	6.513717000000	2.884775000000
C	-1.498770000000	3.274635000000	8.837426000000
H	-0.581494000000	2.656330000000	8.965952000000
C	1.394525000000	5.438917000000	5.222902000000
C	0.340557000000	7.023846000000	3.804279000000
O	0.173404000000	6.106738000000	4.876217000000
H	1.198683000000	4.847150000000	6.136068000000
H	-0.643440000000	7.487224000000	3.593400000000
O	-1.250798000000	4.391266000000	7.990709000000
H	2.196098000000	6.179662000000	5.441689000000
H	1.061294000000	7.829795000000	4.072231000000
K	-2.113584000000	4.813487000000	5.477326000000
C	-0.274439000000	5.279231000000	8.526339000000
H	-0.610746000000	5.708847000000	9.497062000000
H	0.697918000000	4.759297000000	8.684196000000
H	-0.127698000000	6.098831000000	7.797189000000
H	1.178711000000	4.372974000000	0.278776000000
H	2.204372000000	4.389449000000	1.748464000000
H	0.415468000000	4.483700000000	1.906685000000
H	2.030674000000	2.026137000000	0.857294000000

8. References

[1] D. Seebach, G. Stucki, P. Renaud, *Chimia* **1988**, *42*, 176–178.

[2] A. Avenoza, J. H. Busto, F. Corzana, J. M. Peregrina, D. Sucunza, M. M. Zurban, *Synthesis* **2005**, *4*, 575–578.

[3] K. Barlos, P. Mamos, D. Papaioannou, S. Patrianakou, C. Sanida, W. Schäfer, *Liebigs Ann. Chem.* **1987**, 1025–1030.

[4] Bruker. *SAINT+ v8.38A Integration Engine, Data Reduction Software, Bruker Analytical X-ray Instruments Inc., Madison, WI, USA* **2015**.

[5] Bruker. *SADABS 2014/5, Bruker AXS area detector scaling and absorption correction, Bruker Analytical X-ray Instruments Inc., Madison, Wisconsin, USA* **2014/5**.

[6] Sheldrick, G. M. A short history of SHELX. *Acta Crystallographica Section A* **2008**, *64*, 112-122.

[7] Sheldrick, G. M. SHELXT - Integrated space-group and crystal-structure determination. *Acta Crystallographica a-Foundation and Advances* **2015**, *71*, 3-8.

[8] Sheldrick, G. M. Crystal structure refinement with SHELXL. *Acta Crystallographica Section C-Structural Chemistry* **2015**, *71*, 3-8.

[9] Dolomanov, O. V.; Bourhis, L. J.; Gildea, R. J.; Howard, J. A. K.; Puschmann, H. OLEX2: a complete structure solution, refinement and analysis program. *Journal of Applied Crystallography* **2009**, *42*, 339-341.

[10] F. Neese, F. Wennmohs, U. Becker, C. Riplinger, *J. Chem. Phys.* **2020**, *152*, 224108.

[11] A. D. Becke, *J. Chem. Phys.* **1993**, *98*, 5648–5652.

[12] C. Lee, W. Yang, R. G. Parr, *Phys. Rev. B* **1988**, *37*, 785–789.

[13] S. Grimme, *Wiley Interdiscip. Rev. Comput. Mol. Sci.* **2011**, *1*, 211–228.

[14] J. Zheng, X. Xu, D. G. Truhlar, *Theor. Chem. Acc.* **2011**, *128*, 295–305.

[15] S. Grimme, *Chem. Eur. J.* **2012**, *18*, 9955–9964.

[16] J. P. Perdew, K. Burke, M. Ernzerhof, *Phys. Rev. Lett.* **1996**, *77*, 3865–3868.

[17] J. Contreras-García, E. R. Johnson, S. Keinan, R. Chaudret, J.-P. Piquemal, D. N. Beratan, W. Yang, *J. Chem. Theory Comput.* **2011**, *7*, 625–632.

[18] R. Laplaza, F. Peccati, R. A. Boto, C. Quan, A. Carbone, J.-P. Piquemal, Y. Maday, J. Contreras-García, *Wiley Interdiscip. Rev. Comput. Mol. Sci.* **2021**, 11, e1497.

[19] W. Humphrey, A. Dalke, K. Schulten, *J. Mol. Graph.* **1996**, *14*, 33–38.

[20] C. Adamo, V. Barone, *J. Chem. Phys.* **1999**, *110*, 6158–6170.

[21] E. Caldeweyher, S. Ehlert, A. Hansen, H. Neugebauer, S. Spicher, C. Bannwarth, S. Grimme, *J. Chem. Phys.* **2019**, *150*, 154122.

[22] A. V. Marenich, C. J. Cramer, D. G. Truhlar, *J. Phys. Chem. B* **2009**, *113*, 6378–6396.
